# Deciphering miR-520c-3p as a probable target for immunometabolism in non-small cell lung cancer using systems biology approach

**DOI:** 10.18632/oncotarget.28233

**Published:** 2022-05-24

**Authors:** Pooja Gulhane, Prajakta Nimsarkar, Komal Kharat, Shailza Singh

**Affiliations:** ^1^National Centre for Cell Science, NCCS Complex, Ganeshkhind, SP Pune University Campus, Pune 411007, India

**Keywords:** non-small cell lung cancer, miRNAs, miR-520c-3p, PI3K/AKT signaling pathway, therapeutic targeting

## Abstract

Background: Non-small cell lung cancer (NSCLC) is considered to have more than 80% of all lung cancer cases, making it the leading cause of cancer-related deaths globally. MicroRNA (miRNA) deregulation has been seen often in NSCLC and has been linked to the disease’s genesis, progression, and metastasis via affecting their target genes.

Materials and Methods: Our study focused on the functionality of down-regulated miRNAs in NSCLC. For this study, we used 91 miRNAs reported to be down-regulated in NSCLC. The targets of these miRNAs were chosen from miRNA databases with functionality in NSCLC, including miRBase, miRDB, miRTV, and others. Inter-regulatory miRNA-NSCLC networks were generated. Simulated annealing was used to improve the network’s resilience and understandability. GSEA was used to examine 24607 genes reported experimentally in order to gain physiologically relevant information about the target miR-520c-3p.

Results: The study revealed functional prominence on miR-520c-3p, down-regulated during NSCLC. The involvement of miR-520c-3p in the PI3K/AKT/mTOR signaling pathway was recognized.

Conclusions: The therapeutic usage by designing a synthetic circuit of miR-520c-3p was explored, which may help in suppressing tumors in NSCLC. Our study holds promise for the successful deployment of currently proposed miRNA-based therapies for malignant disorders, which are still in the early pre-clinical stages of development.

## INTRODUCTION

Lung cancer is the most frequent cancer globally, accounting for more than 1.6 million deaths per year [1, 2]. For therapeutic reasons, lung cancer is classified into Small Cell Lung Cancer (SCLC) and Non-Small Cell Lung Cancer (NSCLC). NSCLC is the most prevalent cancer, contributing to around 85% of all cases. It is subdivided into three histological subtypes: adenocarcinoma (LUAD), squamous cell carcinoma (LUSC), and large-cell carcinoma [3, 4]. The life expectancy for advanced stages with malignant tumors is only 4%. The absence of practical techniques and methods for early detection of NSCLC and its resistance to most presently available medications are serious issues [5].

In the case of NSCLC, much of the recent research has focused on epidermal growth factor receptor (EGFR) mutations and aberrant fusions of the anaplastic lymphoma kinase (ALK) or c-ros oncogene 1 (ROS1) genes [6, 7]. In contrast, chemotherapy remains the gold standard in treating advanced NSCLC patients with no druggable genetic abnormalities. The PI3K/AKT/mTOR pathway and signaling cascade regulates cellular growth and metabolism. Increased activation of the phosphatidylinositol-3-kinase (PI3K)/AKT/mTOR pathway results in many cancer hallmarks such as acquired growth signal autonomy, apoptosis inhibition, sustained angiogenesis, increased tissue invasion, metastasis, and anti-growth signal insensitivity. As a result, this route is a promising target for new anticancer medicines [8].

The acknowledgment of NSCLC as a disease with intricate genetics has made significant progress in the last two decades in research on the underlying molecular pathways of lung carcinogenesis. This offered reason to believe that the notion of personalized medicine might be successfully implemented in the treatment of lung cancer in the future.

**Figure 1:**
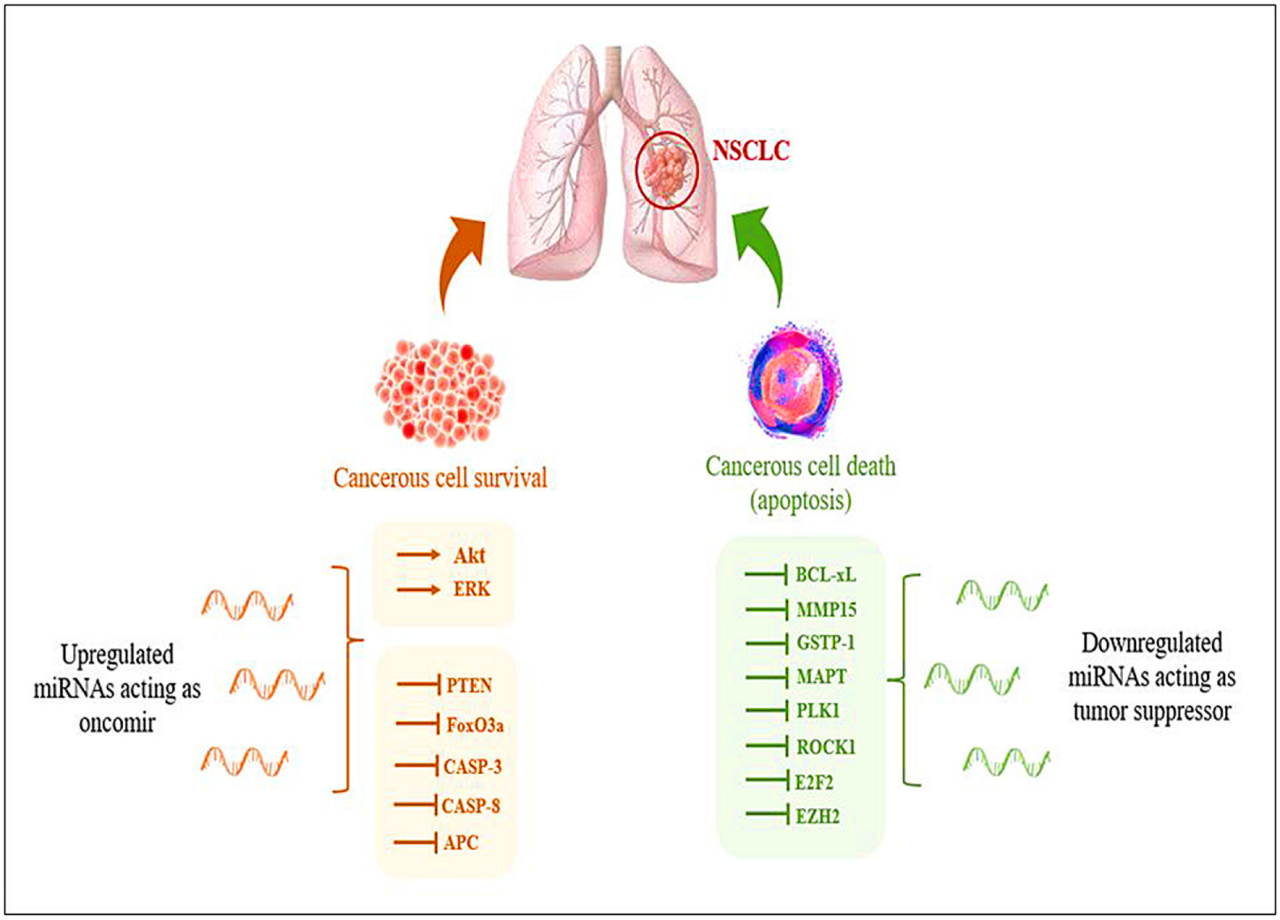
Representation of deregulation of miRNAs during NSCLC aiding in tumor progression.

Identifying reliable biomarkers to guide clinical decision-making is one of the prerequisites for developing personalized medicine [9]. Small, non-coding microRNA (miRNA) molecules’ potential quickly becomes evident in this environment. The potential role of miRNA-based biomarkers to complement radiographic modalities and boosting the total sensitivity and specificity of the lung cancer screening process is now being studied in the context of personalized medicine [10]. Deregulation in the expression of various miRNAs has been observed during NSCLC, which aids in tumor growth and progression (Figure 1).

Because of the widespread role of miRNAs in human diseases, including all cancer types, new therapeutic strategies have been developed based on the identification and validation of miRNAs that are causally involved in the disease process and the effective regulation of target-miRNA function by drugs. Many researchers have used cell lines, matched tissue samples, and blood samples to conduct miRNA profiling investigations in NSCLC. miRNA profiling assays based on micro-arrays offer a reliable way to screen hundreds of miRNAs. Well-documented miRNA signatures have been identified in the literature [11]. Several miRNAs have recently been discovered to target critical cancer-related immunological pathways and immune cell secretion of immunosuppressive or immune-stimulatory substances [12, 13]. The clinical trial of a synthetic oligonucleotide “MiRavirsen” (SPC3649), homologous to miR-122 that can sequester and limit the functionality of this miRNA, has recently been expanded to a long-term phase 2 study for patients with chronic hepatitis C virus genotype-1 infection [14].

Our research focuses on identifying down-regulated miRNAs in NSCLC and their role in tumor progression inhibition. Due to down-regulation in the expression of specific miRNAs, targets such as PI3K/AKT, PDK1, PDK2, CDK1, and others are enhanced. It is critical to recognize these significant miRNAs if they are to be used as therapeutic interventions in the future.

## RESULTS

### Inter-regulatory miRNA-NSCLC associated network construction

**Figure 2:**
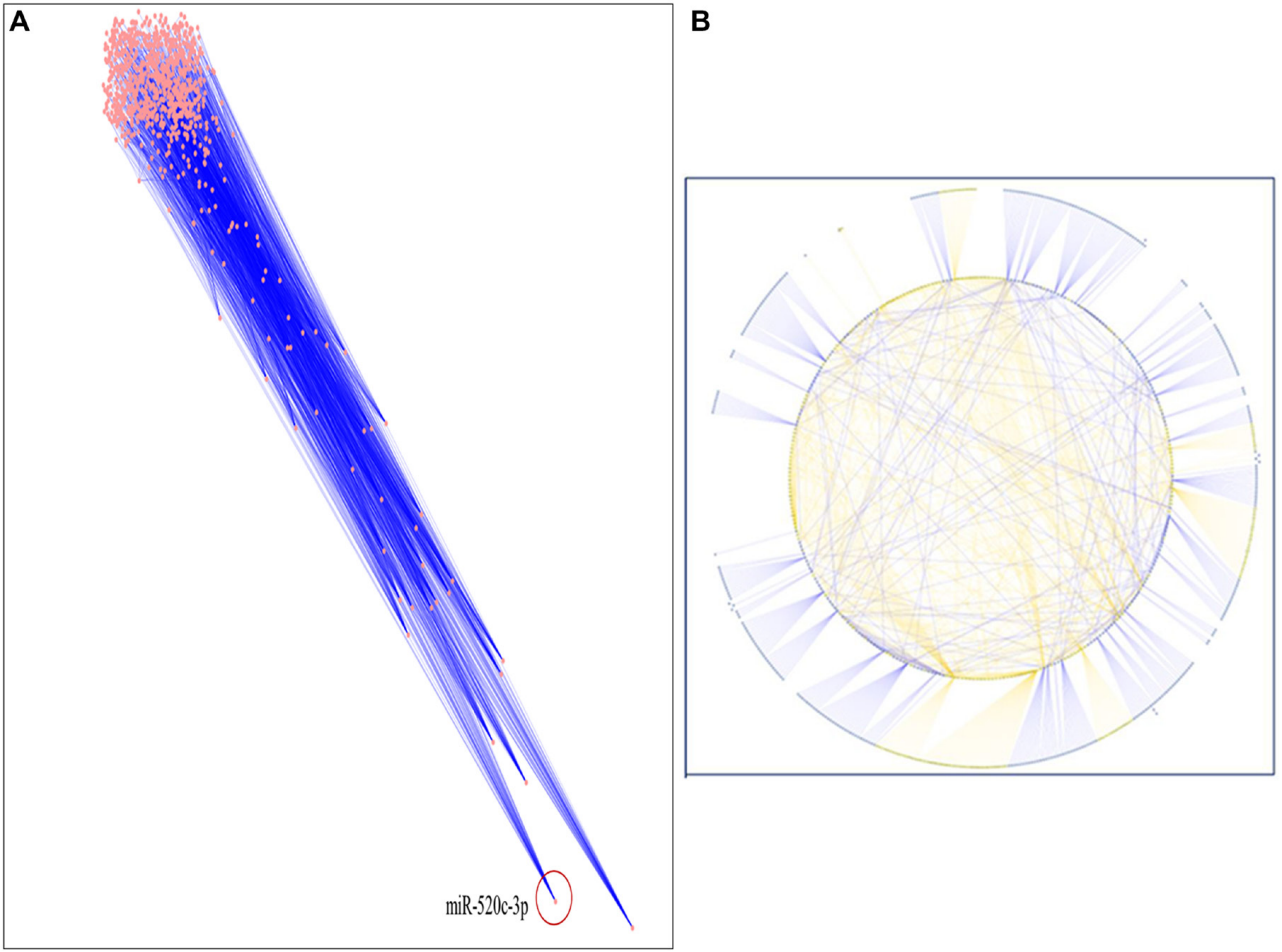
(**A**) The inter-regulatory miRNA network after running simulated annealing algorithm showing placement of highly clustered miR-520c-3p. (**B**) The simulated network in circular layout demonstrating the strength of chosen miRNAs over the whole network.

A thorough literature review was conducted to accumulate a list of chosen miRNAs based on scientific literature searches/published miRNA data or a collection of differentially expressed miRNAs discovered using array or sequencing methods [15–17]. In this study, we used an enlisted 91 miRNAs reported to be down regulated in NSCLC. The targets of these miRNAs were chosen from miRNA databases with functionality in NSCLC, and subsequently, inter-regulatory miRNA-NSCLC networks were generated. Simulated annealing was used to improve the network’s resilience and comprehensibility. The simulated annealing algorithm analyses and positions each node such that the most clustered or weighted nodes on the entire network are on the bottom. In contrast, nodes with fewer clusters are placed in the upper section in descending order. let-7a-2 was placed first and miR-520c-3p was placed second most bottom in the simulated annealing layout network. The entire network was analyzed using parameters like Betweenness Centrality, Degree of Nodes, Edge Betweenness, Closeness Centrality, Clustering Coefficient, and others to present a condensed and robust nature of the inter-regulatory miRNAs network with their putative targets (Figure 2A, 2B and Table 1).

**Table 1:** Network analysis depicts the network’s resilience depending on various parameters

Statistical Summary	
Number of nodes	804
Number of edges	1278
Avg. number of neighbors	3.354
Network diameter	10
Network radius	5
Characteristic path length	4.453
Clustering Coefficient	0.005
Network Density	0.005
Connected Components	28
Network heterogeneity	1.920
Network centralization	0.069

### Identification of significantly linked regions in the miRNAs-target network

**Figure 3:**
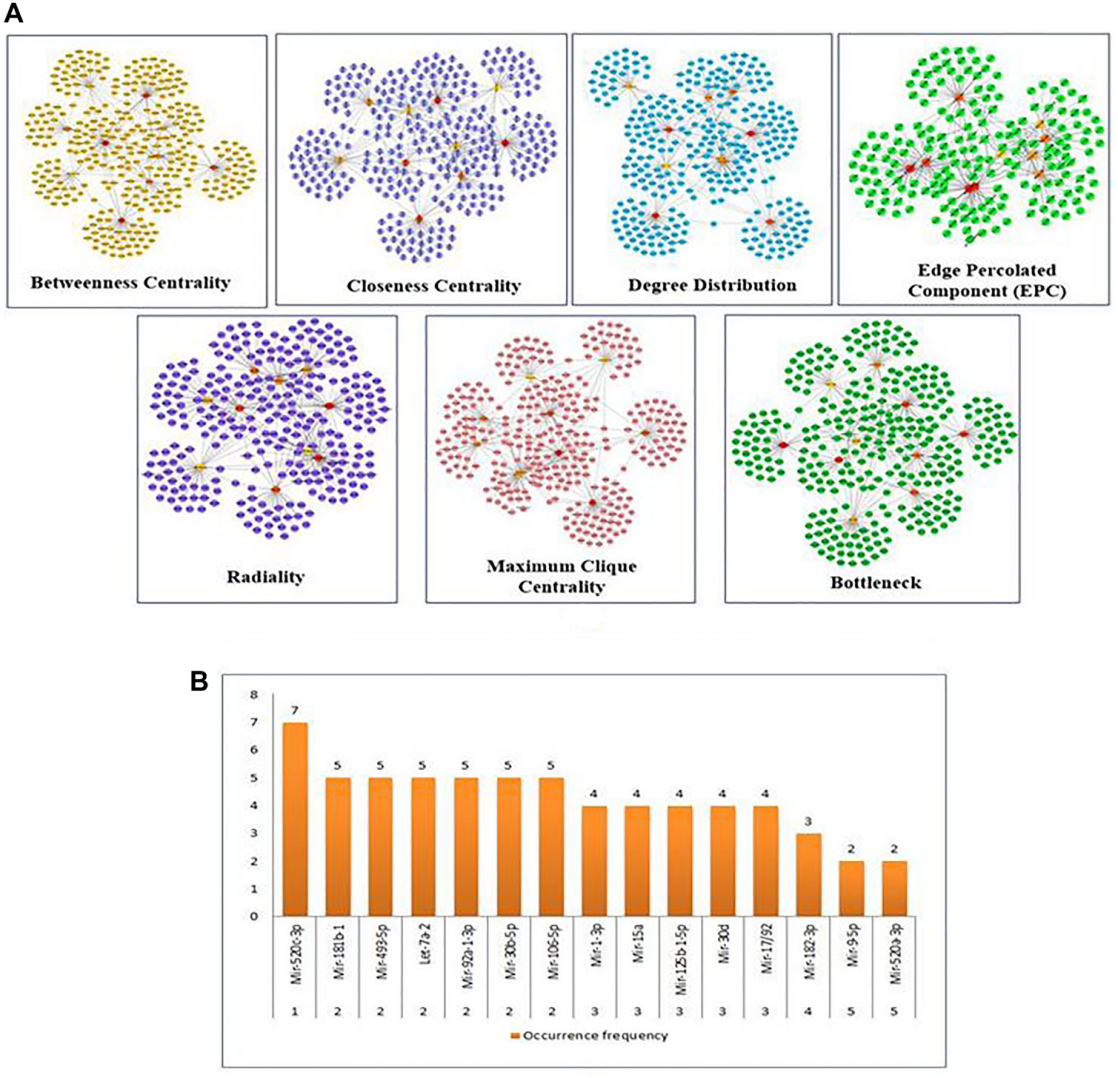
(**A**) Substantial modules based on Betweenness centrality; Closeness centrality; Degree Distribution; Edge percolated Component (EPC); Radiality; Maximum Clique Centrality (MCC); and Bottleneck were identified from the leading network. From top to bottom, red to light yellow in modules denotes the rank. (**B**) The top 5 ranked miRNAs are graphically represented based on their frequency of occurrence in the 11-scoring method of CytoHubba.

The significantly linked regions in the miRNAs-target network were identified using Cytoscape filters, where 1-56 out degree nodes were inclusive and the CytoHubba plugin. In Figure 3, the CytoHubba was used to find the most valuable modules and top-ranked nodes in the entire network. MCC, DMNC, MNC, EPC, Radiality, Degree centrality, Closeness centrality, Betweenness centrality, Edge Betweenness, Clustering Coefficient, and Stress centrality are the 11 scoring methods that were utilized to identify the main functional modules and top-ranking miRNAs/targets in the network using the CytoHubba. Each scoring technique yielded the top 10 nodes in the network. Based on their occurrence in each of the top 10 scoring methods, the top 5 miRNAs are represented in Figure 3A and 3B. miR-520c-3p was the most prominent in the analysis, as mentioned above. The CytoHubba rank table has been provided in Supplementary Table 1.

**Figure 4:**
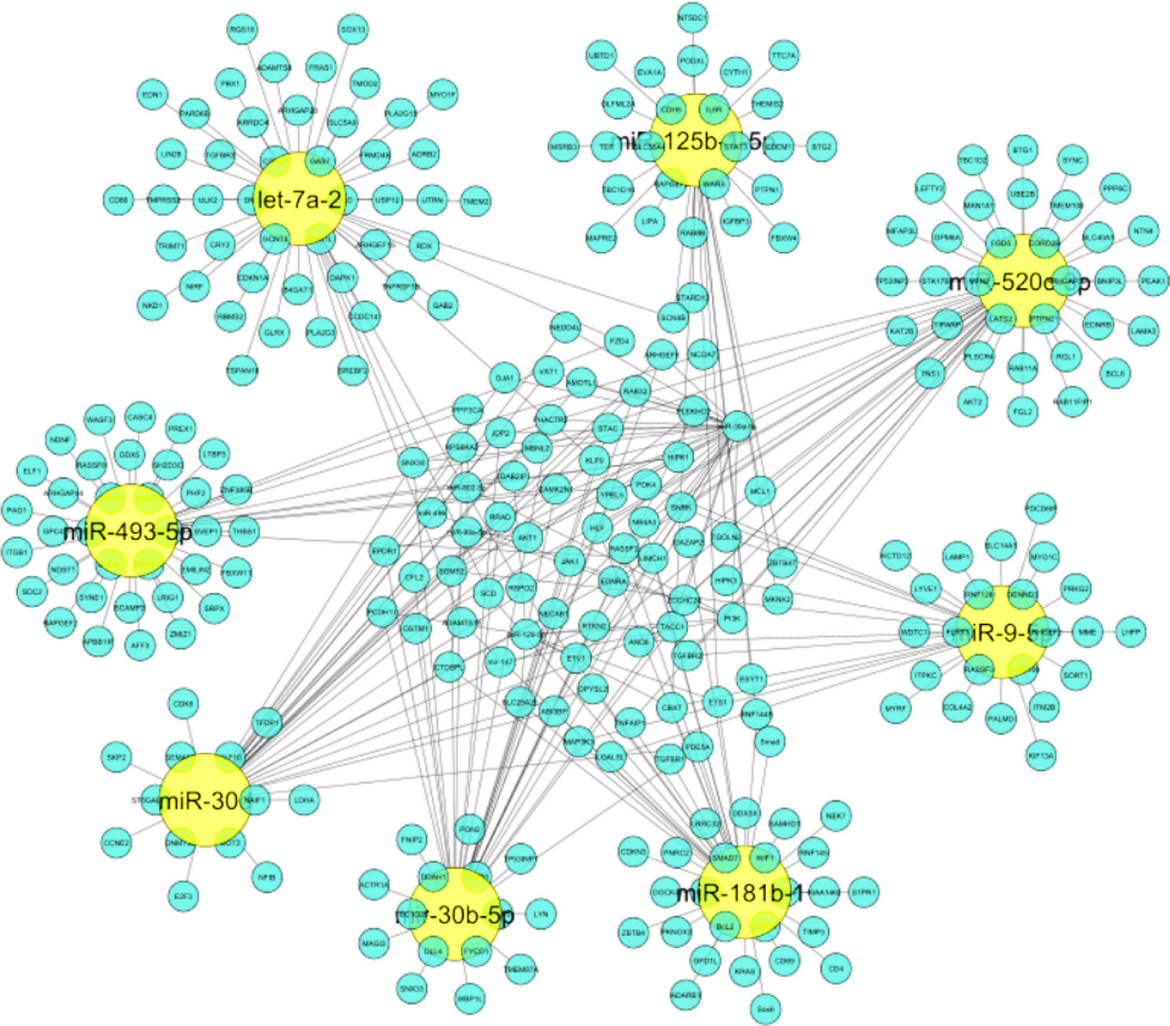
Inter-regulatory miRNA hub network generated which represents significance of identified miRNAs on entire network. Yellow colored nodes depict identified miRNAs and Cyan blue colored nodes are its respective targets.

The importance of identified miRNAs on the overall network was calculated using an inter-regulatory miRNAs hub network (Figure 4). The identified miRNAs were miR-520c-3p, miR-493-5p, miR-181b-1, miR-30b-5p, miR-30d, miR-125b-15p, miR-9-5p, let-7a-2. MiR-520c-3p was chosen because it has the most prevalence across the entire inter-regulatory miRNAs network generated and filtered by the Cytoscape filters and the CytoHubba analysis. To be considered for use as a therapeutic intervention, miRNAs must be unique and have low participation in the cells’ conventional metabolic processes.

The various analyses show that the identified miRNAs were not chosen at random.

**Figure 5:**
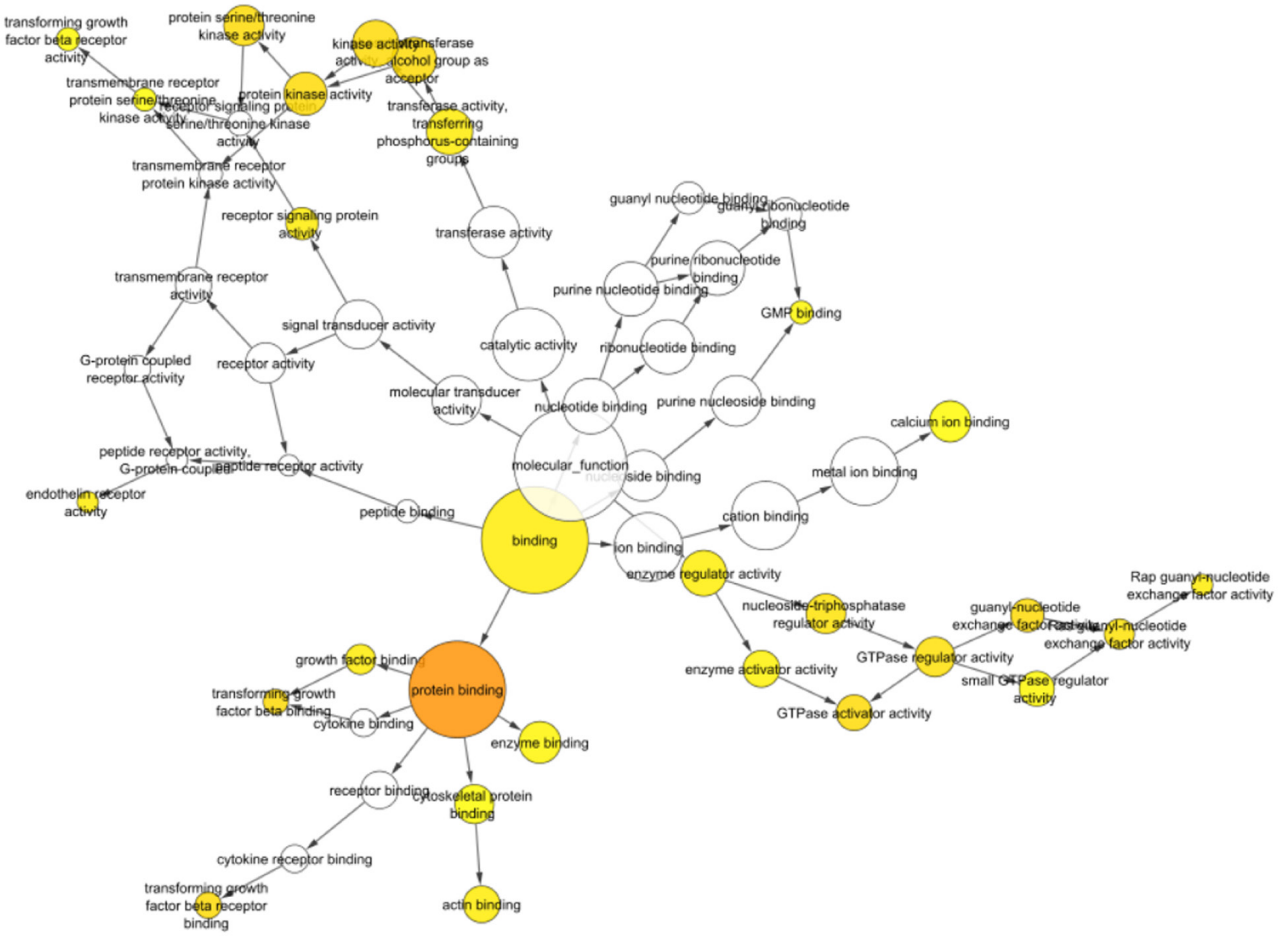
Bingo visualization of identified miRNAs shows significant involvement in protein binding activity, signal transduction, GTPase activity, cytokine binding, transforming growth factor beta binding and more which are involved in various biological and immunological processes. The darker and larger the size indicates more involvement of identified miRNAs.

The BiNGO plugin helped to find the GO categories significantly over-represented in a collection of genes or a biological network sub-graph [18]. The Biological Networks Gene Ontology tool (BiNGO, version 3.0.3; http://apps.cytoscape.org/apps/bingo) was used to investigate and display the biological processes and cellular components of identified hub miRNAs and targets. As an outcome, a graph depicting the gene ontology words overrepresented in the network (Figure 5 and Table 2) and a table including the data, including the *p*-value, corrected *p*-value, and cluster frequency, were generated (Supplementary Table 2), where the identified miRNAs show involvement in various biological pathways which are shown in Table 1.

**Table 2:** Identified miRNAs with their respective targets retrieved via BINGO analysis in tabulated manner showing involvement of identified miRNAs in inflammatory and immune mechanisms

GO-ID	*p*-value	Description
5515	8.97E-11	protein binding
50794	1.01E-10	regulation of cellular process
50789	9.07E-10	regulation of biological process
65007	1.32E-09	biological regulation
51270	3.28E-09	regulation of cellular component movement
1944	8.72E-09	vasculature development
1568	1.03E-07	blood vessel development
30154	5.26E-07	cell differentiation
10646	6.86E-07	regulation of cell communication
2520	7.67E-07	immune system development
35466	9.90E-07	regulation of signaling pathway
30334	1.08E-06	regulation of cell migration
48514	1.44E-06	blood vessel morphogenesis
43068	4.76E-06	positive regulation of programmed cell death
43065	4.43E-06	positive regulation of apoptosis
10942	5.49E-06	positive regulation of cell death
51271	9.87E-06	negative regulation of cellular component movement
70482	3.26E-05	response to oxygen levels
30336	3.88E-05	negative regulation of cell migration
8285	7.93E-05	negative regulation of cell proliferation
7166	9.04E-05	cell surface receptor linked signaling pathway
6917	1.70E-04	induction of apoptosis
12502	1.77E-04	induction of programmed cell death
82	2.56E-04	G1/S transition of mitotic cell cycle
1666	2.78E-04	response to hypoxia
1525	3.42E-04	angiogenesis
10746	2.64E-03	regulation of plasma membrane long-chain fatty acid transport
51329	3.01E-03	interphase of mitotic cell cycle
30308	3.01E-03	negative regulation of cell growth
43029	5.29E-03	T-cell homeostasis
1569	6.09E-03	patterning of blood vessels
31323	6.75E-03	regulation of cellular metabolic process
51726	6.96E-03	regulation of cell cycle

Enrichment map analysis conducted shows the pathways sharing several genes are represented as circles (nodes) linked by lines (edges). Enrichment Score (ES) colors the nodes and the number of genes shared by the associated pathways determines the size of the edges. The nodes were organized so that substantially comparable gene sets were clustered together; these clusters were recognized and linked to biological activities. The Enrichment Map visualized the gene-set enrichment results. Edge thickness was proportional to the overlap between gene sets, computed using the Jaccard or overlap coefficients. Node size reflected the number of genes in the gene set; edge thickness is proportional to the overlap between gene sets, determined using the Jaccard or overlap coefficients.

**Figure 6:**
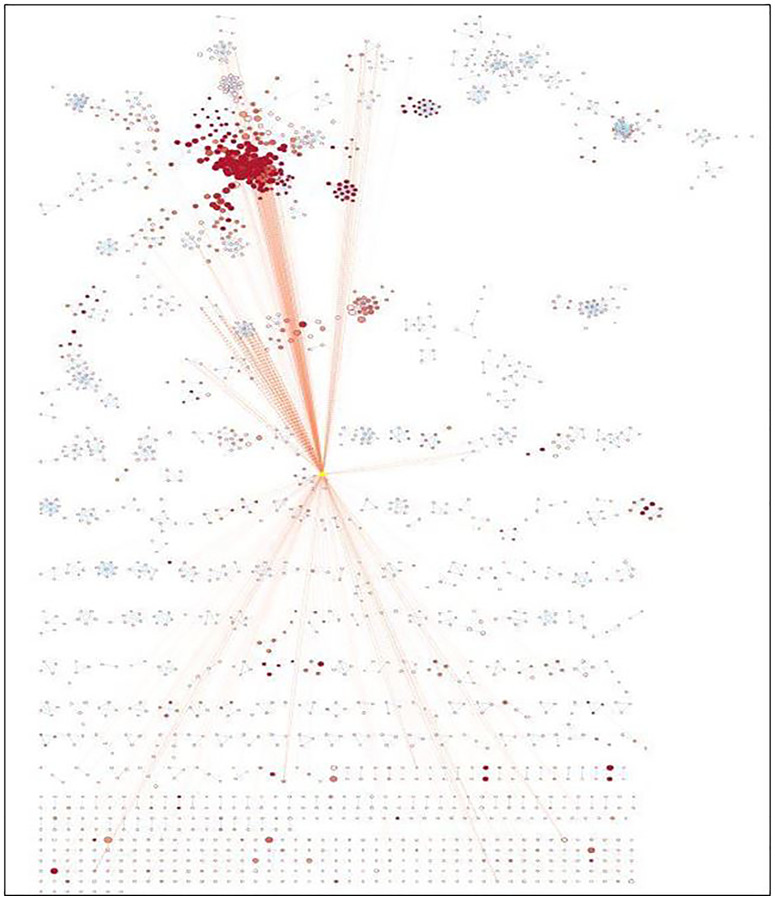
Enrichment map generated for identified miRNAs.

The enrichment map acknowledged the significant involvement of identified miRNAs in vital pathways such as immune response, cell signaling, cell growth, cell proliferation, etc. (Supplementary Table 3). The enrichment map is represented in Figure 6 and Table 3 with their respective *p*-value validating the contribution of identified miRNAs down-regulated during NSCLC aiding in tumor progression.

**Table 3:** Immune and inflammatory pathways affected due to down-regulation of identified miRNAs tabulated

Enrichment Map: Name	Enrichment Map: GS_DESCR	*p*-value
GO.0045089	positive regulation of innate immune response	0.0021
GO.0045088	regulation of innate immune response	0.0000103
GO.0090087	regulation of peptide transport	6.91E-06
GO.0071347	cellular response to interleukin 1	0.005
GO.0071345	cellular response to cytokine stimulus	0.000000324
GO.0070498	interleukin-1-mediated signaling pathway	0.00082
GO.0002376	immune system process	4.52E-08
GO.2001020	regulation of response to DNA damage stimulus	0.0015
GO.1902882	regulation of response to oxidative stress	4.80E-05
HSA-176408	Regulation of APC/C activators between G1/S and early anaphase	1.55E-05
HSA-176407	Conversion from APC/C: Cdc20 to APC/C:Cdh1 in late anaphase	1.15E-05
GO.0090092	regulation of transmembrane receptor protein serine/threonine kinase signaling pathway	8.12E-06
GO.0070482	response to oxygen levels	4.38E-09
GO.0090090	negative regulation of canonical Wnt signaling pathway	5.60E-04
HSA-198693	AKT phosphorylates targets in the nucleus	0.006
HSA-1257604	PIP3 activates AKT signaling	3.04E-13
GO.1901992	positive regulation of mitotic cell cycle phase transition	9.80E-04
GO.0090068	positive regulation of cell cycle process	3.18E-05
KW-0072	Autophagy	0.0059
GO.0045937	positive regulation of phosphate metabolic process	3.25E-05
GO.0031325	positive regulation of cellular metabolic process	1.43E-39
KW-0043	Tumor suppressor	5.20E-05
KW-0053	Apoptosis	1.52E-08

### Enrichment analysis of miR-520c-3p via gene-set enrichment analysis (GSEA)

Gene Set Enrichment Analysis (GSEA) method was used for analysing microarray data at the gene set level [19]. Prior biological knowledge, such as published information on metabolic pathways or co-expression in previous studies, was used to identify the gene sets. We considered the GSEA’s capability where 24607 genes were analysed to give physiologically relevant insights for miR-520c-3p for proportionate background information (Supplementary Table 4). We explored the enrichment/expression of miR-520c-3p in BEAS-2B vs. A549 cells. Gene set enrichment analysis for BEAS-2B vs. A549 cells shows gradual decrease in expression of miR-520c-3p indicating negative correlation with tumor gene expression (A549 cells) as it was differentially expressed during NSCLC.

**Figure 7:**
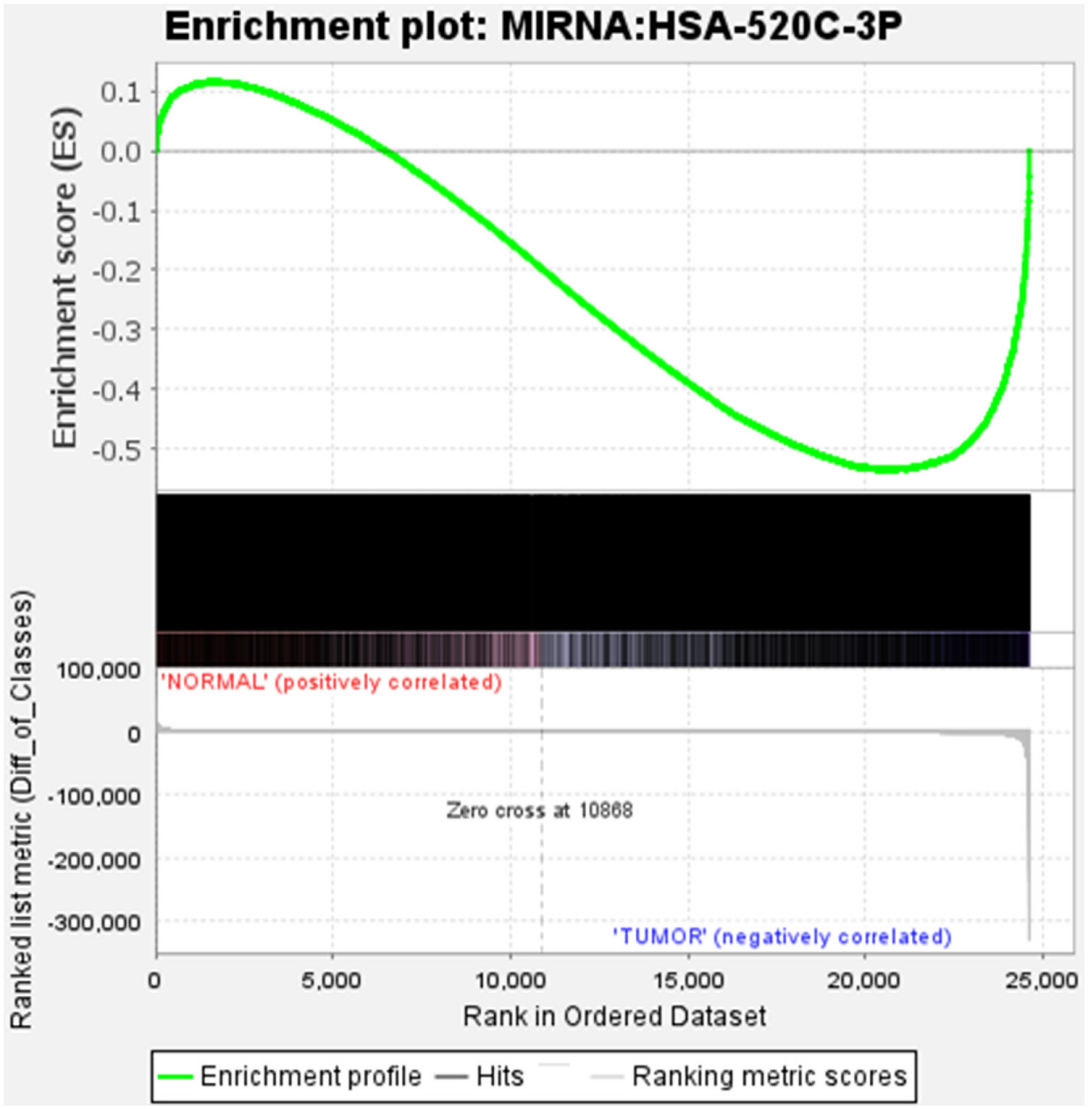
The position of the maximum enrichment score (ES) and the leading-edge subset are plotted in the running sum for S in the data set. Enrichment analysis of miR-520c-3p in BEAS-2B vs. A549 cells is represented.

The plot’s middle section displayed where the gene set’s members appeared in the ranked list of genes. The gene set data was only for miR-520c-3p; it appears in only one color (black). The ranking metric assesses the relationship between a gene and a phenotype—the ranking metric’s value shifts from positive to negative as we move down the ranked list. A positive value correlates with the first phenotype, whereas a negative value indicates a correlation with the second phenotype. miR-520c-3p appears to be connected in both positive and negative correlation as it is involved in both the first and second phenotype of genes playing a role in NSCLC (Figure 7).

### Pathway enrichment analysis of identified miRNAs

**Figure 8:**
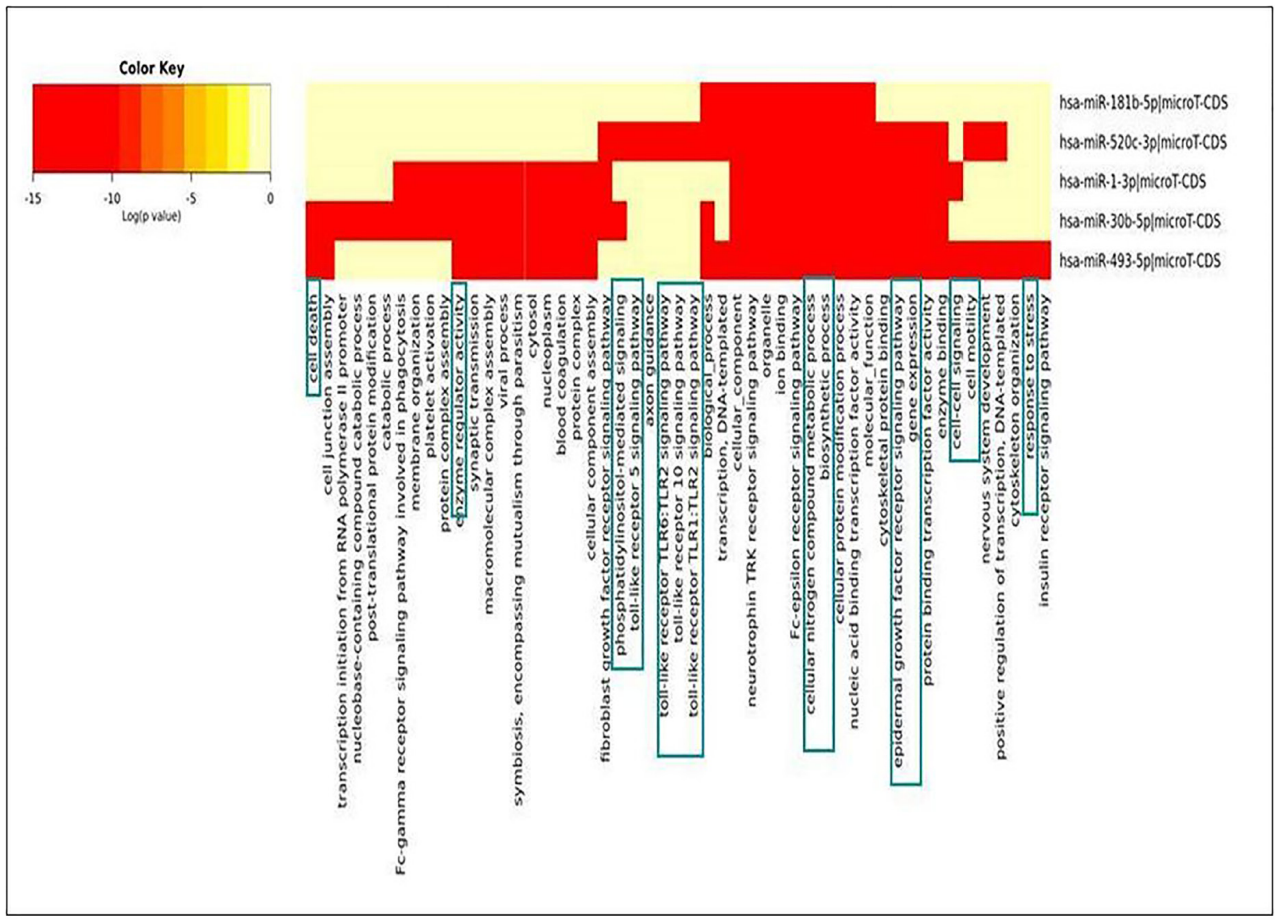
Heat-map generated for identified down-regulated miRNAs during NSCLC.

Pathway enrichment analysis is critical in using a current understanding of genes and biological processes to analyse high-throughput data [20]. The heatmap was generated for seeking relationships of identified miRNAs with various cellular pathways via DIANA Tools. The database utilized for generating the heat map was Gene Ontology and micro-TDS. The resulting heatmap depicted that the down-regulated miRNAs during NSCLC acted primarily on cell death, TLR-Signaling, response towards stress, cell cycle regulation, immune response process, etc., (Figure 8). The pathway enrichment analysis results made the picture clear that differential expression of miRNAs plays a significant role in tumor progression in NSCLC. If down-regulated miRNAs were expressed in normal conditions, cell-cycle regulation, cellular growth progression, cell proliferation, etc., would have been supervised wisely.

### Mutagenesis during NSCLC

**Figure 9:**
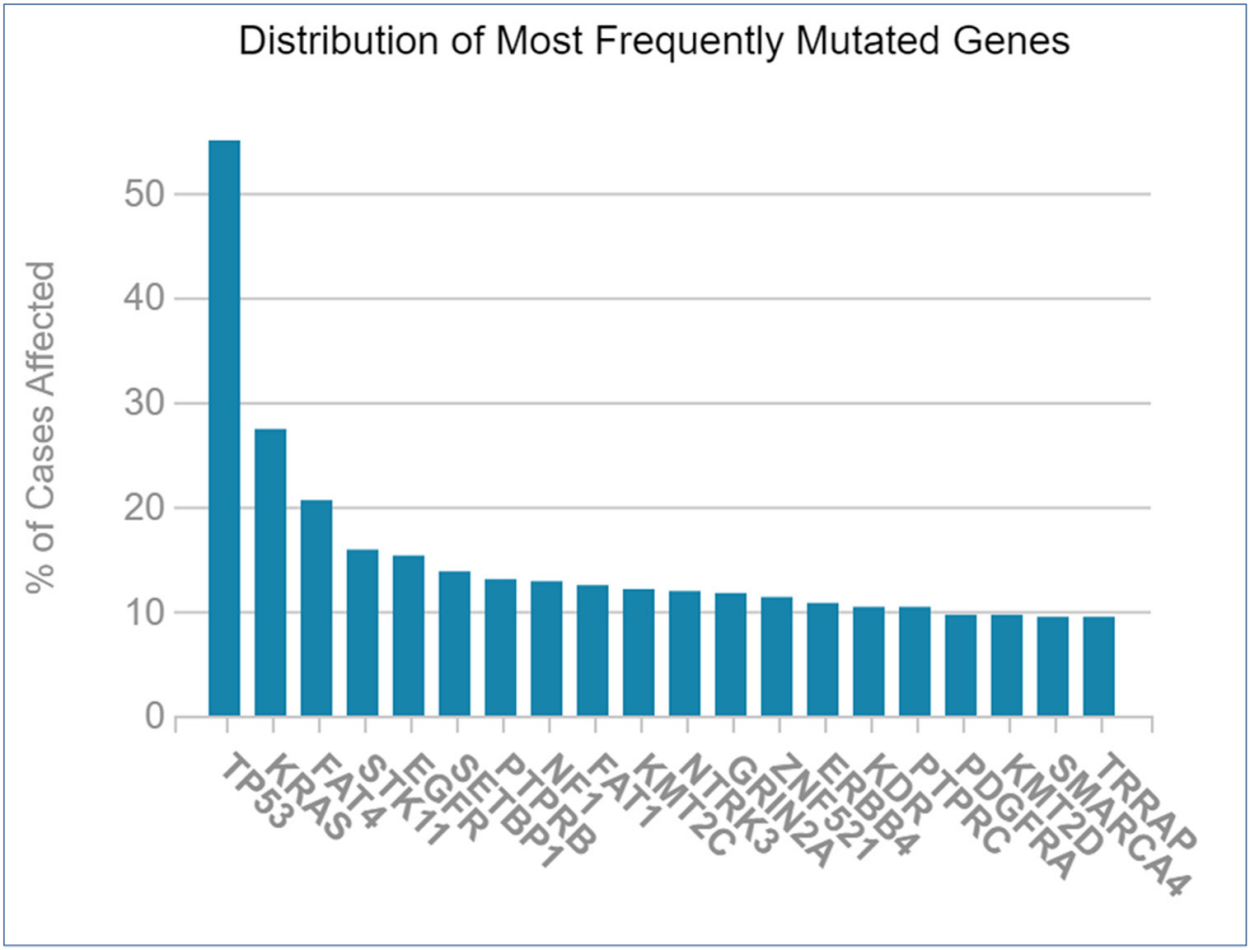
Frequently mutated genes during NSCLC vs. percentage of cases affected.

The genes that were mutated the most were crucified from TCGA database for NSCLC. The resulting graph shows that TP53 (p53) has the most mutations; followed by KRAS, FAT4, STK11, EGFR, and others (Figure 9). Mutagenesis research was essential for selecting therapeutic targets since mutations in targeted genes via miRNAs would impair the function of identified miRNAs.

### Sequence-based miR-520c-3p target prediction analysis

MiR-520c-3p was identified as the most prominent down-regulated miRNA in NSCLC based on the studies mentioned above. It was essential to search its targets since it significantly impacted several immunometabolic pathways, which were down-regulated, resulting in tumor growth. It was achieved using tools4miR; further, TargetScan provided the complementary seed sequences of miR-520c-3p and their respective targets, and the KMP algorithm validated the results from TargetScan. Tools4miR gave comprised assessment of miR-520c-3p possible targets via eight databases, including miRanda, PITA, MiRmap, MicroTar, etc. (Supplementary Table 5).

The complementary seed sequences of miRNAs with their respective targets and the location where complementary base pairing occurred were given by TargetScan. The properties of original context scores (i.e., site type, 3′-supplementary pairing, local AU content, and distance from the nearest 3′-UTR end) are taken into account by TargetScan. mRNAs were ranked with conventional 7–8nt miRNA sites in their 3′ UTRs. TargetScan predictions included both the context++ scores and the current isoform information (Table 4).

**Table 4:** TargetScan results depicting association of miR-520c-3p with PI3K/AKT signaling pathway

Targets of miR-520c-3p	Predicted Consequential pairing of Target region (top) and miRNA (bottom)	Site type	Context++ score
Position 768-774 of AKT 3′ UTR hsa-miR-520c-3p	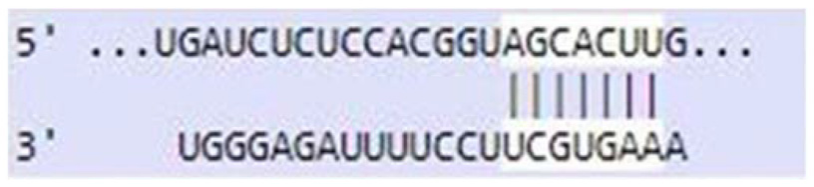	7 mer-m8	-0.15
Position 415-421 of AKTP 3′ UTR hsa-miR-520c-3p	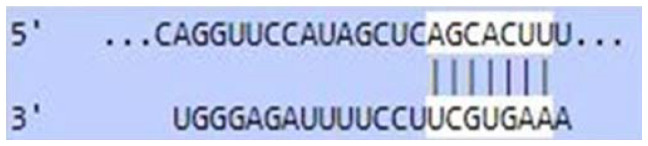	7 mer-m8	-0.2
Position 205-211 of CDK2 3′ UTR hsa-miR-520c-3p	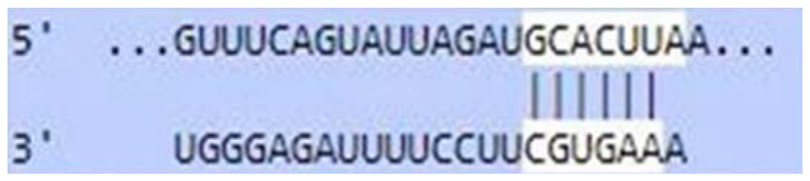	7 mer-A1	-0.22
Position 4574-4580 of EGFR 3′ UTR hsa-miR-520c-3p	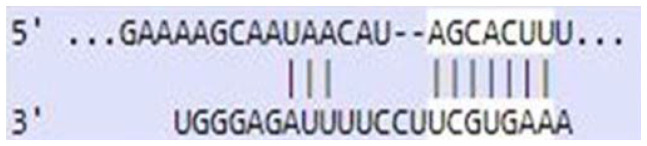	7 mer-m8	-0.11
Position 191-198 of FOXO3 3′ UTR hsa-miR-520c-3p	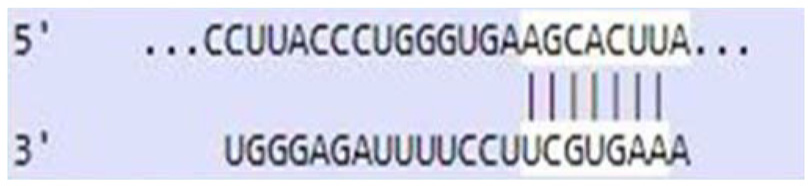	8mer	-0.03
Position 264-270 of CDK19 3′ UTR hsa-miR-520c-3p	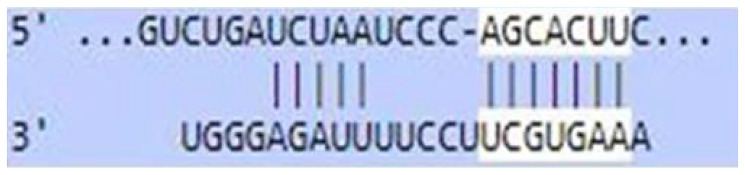	7 mer-m8	-0.19
Position 9154-9160 of TLR4 3′ UTR hsa-miR-520c-3p	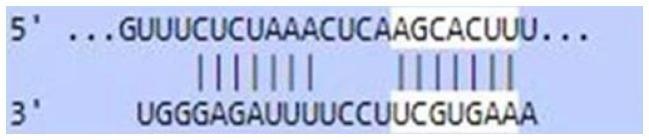	7 mer-m8	-0.15

KMP algorithm gave positive relation of miR-520c-3p with respective targets from the AKT/PI3K signaling pathway. It was confirmed as in every mRNA transcript variant of AKT1, AKT1P, CDK2, FOXO3, CDK19, and EGFR, the seed sequence for complementary base pairing with miR-520c-3p was present (Supplementary Material 1).

The findings strongly suggested miR-520c-3p’s participation in the AKT/PI3K signaling pathway. The PI3K/AKT/MTOR pathway has been implicated in carcinogenesis and disease progression in NSCLC. Up-regulation of the AKT pathway has also been seen in a substantial number of NSCLC patients. In a study of 110 NSCLC tumors, immunohistochemistry indicated that 51% had elevated AKT activation. There was also a link between AKT activation and enhanced mTOR and forkhead activity, key AKT downstream targets.

### Survival assessment of down-regulated miRNAs’ identified targets

**Figure 10:**
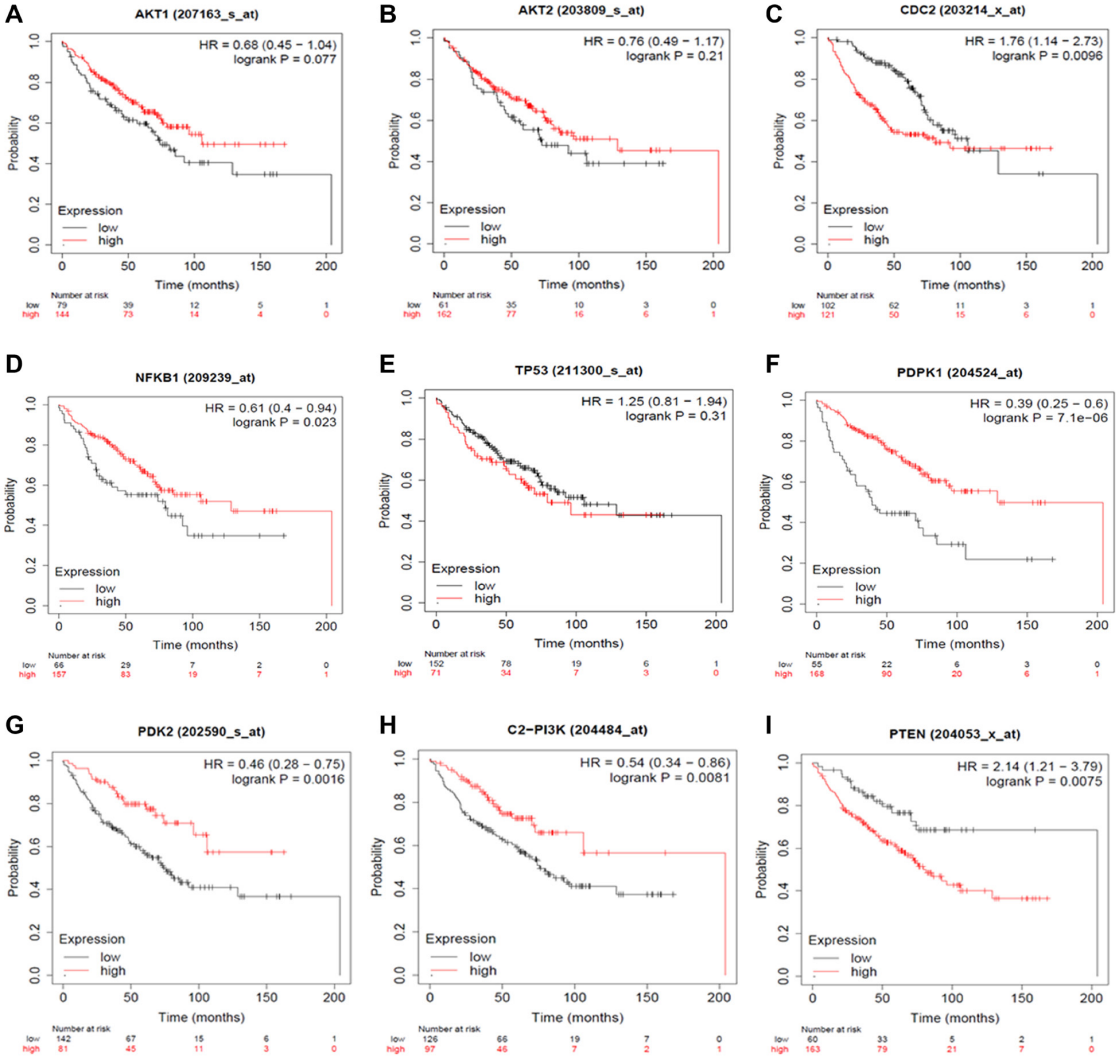
Expression Plots of (**A**) AKT1, (**B**) AKT2, (**C**) CDC2, (**D**) NFKB1, (**E**) TP53, (**F**) PDK1, (**G**) PDK2, (**H**) PI3K and (**I**) PTEN, respectively. The Kaplan Meier Plotter database evaluated the predictive impact of AKT1, AKT2, CDC2, NF-κB, TP53, PDPK1, PDK2, PI3K and PTEN expression NSCLC development. The red line indicates patients with expressions above the median, whereas the black line indicates patients below.

The Kaplan Meier (KM) Plotter was used to considerably evaluate the specific gene expression in Non-Small Cell Lung Cancer progression to validate our results. Briefly, eight genes (AKT1, AKT2, PDPK1, PDK2, NF-κB, PI3K, PTEN, and TP53) were uploaded into the database, and samples were separated into two cohorts based on median gene expression (high vs. low expression) to generate Kaplan-Meier survival graphs, with the number-at-risk (Figure 10).

High mRNA levels of AKT1, AKT2, PDPK1, PDK2, NFKB, and PI3K predicted poor overall survival (OS) in all NSCLC patients, whereas low mRNA levels of PTEN and TP53 were found to indicate worse overall survival (OS) in all NSCLC patients. With the use of HR ratios of certain genes, this may be better understood. The hazard ratios of AKT1 = 0.68, AKT2 = 0.76, PDPK1 = 0.39, PDK2 = 0.46, NFKB = 0.61, and PI3K = 0.54, respectively, indicate lower overall survival, but the hazard ratios of PTEN and TP53 are 2.14 and 1.25, respectively, suggesting worse overall survival.

In Non-Small Cell Lung Cancer, it was discovered that the PI3K/AKT signaling pathway promotes cell survival, proliferation, and angiogenesis in response to extracellular signals. AKT1, AKT2, PDPK1, PDK2, NF-κB, PI3K, PTEN, and TP53 are all critical components in this pathway. The Kaplan Meier Plotter may be used to connect the mRNA expression patterns of these genes to the course of Non-Small Cell Lung Cancer.

#### Survival analysis

**Figure 11:**
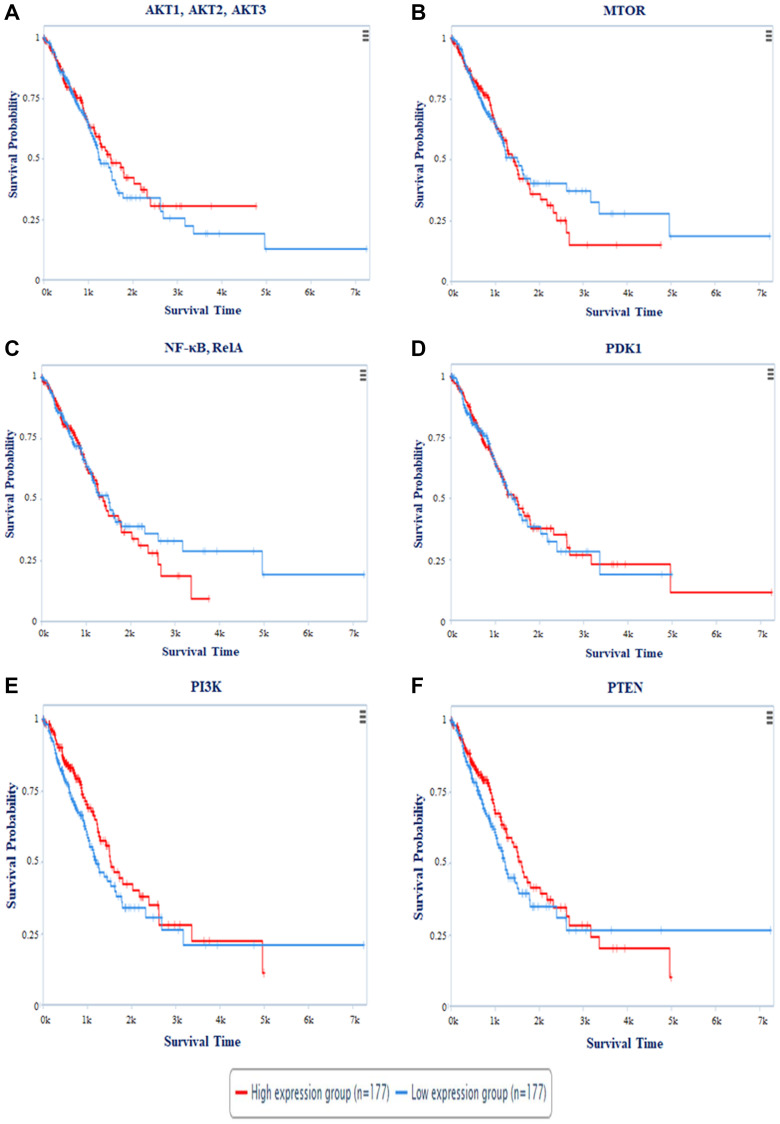
The plot represents survival probability vs. survival time of patients in days. Survival Analysis of (**A**) AKT1, AKT2, AKT3; (**B**) MTOR; (**C**) NF-κB, RelA; (**D**) PDK1; (**E**) PI3K; (**F**) PTEN with survival time period. Red colored line represents the high expression and Blue colored line depicts low expression of the respective genes during NSCLC.

The Cancer Proteome Atlas (TCPA) created a Kaplan Meier (KM) plot for miR-520c-3p targets AKT1, AKT2, AKT3, MTOR, NF-κB (RelA), PDK1, PI3K, and PTEN. The survival plot analysis revealed that patients with high expression of AKT1, AKT2, AKT3, MTOR, PTEN, PI3K, and NF-κB (RelA) have a worse survival rate. Patients with high PDK1 expression had a higher survival rate (Figure 11). Cox′s proportional hazards (Cox P) value model is similar to a multiple regression model. It allows for comparing the survival times of different patients while taking other factors into account. The log-rank test is used to perceive if there is a difference in survival times across groups. However, it does not take into account the other explanatory variables. A positive coefficient in a Cox regression implies a worse prognosis, whereas a negative coefficient suggests a protective impact of the linked variable. The Cox-P value of PTEN, PI3K, PDK1, AKT1, AKT2 & AKT3 showed low hazard value (Table 5). Thus, survival analysis validates PI3K/AKT signaling pathway as targets.

**Table 5:** Cox P value and Log-Rank P value of targeted proteins

Target Proteins	Cox *P* value	log-Rank *P*
AKT1, AKT2, AKT3	0.35081	0.49181
MTOR	0.57039	0.64953
NF-κB, Rel A	0.46215	0.60575
PDK1	0.33433	0.87617
PI3K	0.25909	0.093684
PTEN	0.1277	0.17828

#### Comparative analysis (normal vs. tumor) of targeted genes

**Figure 12:**
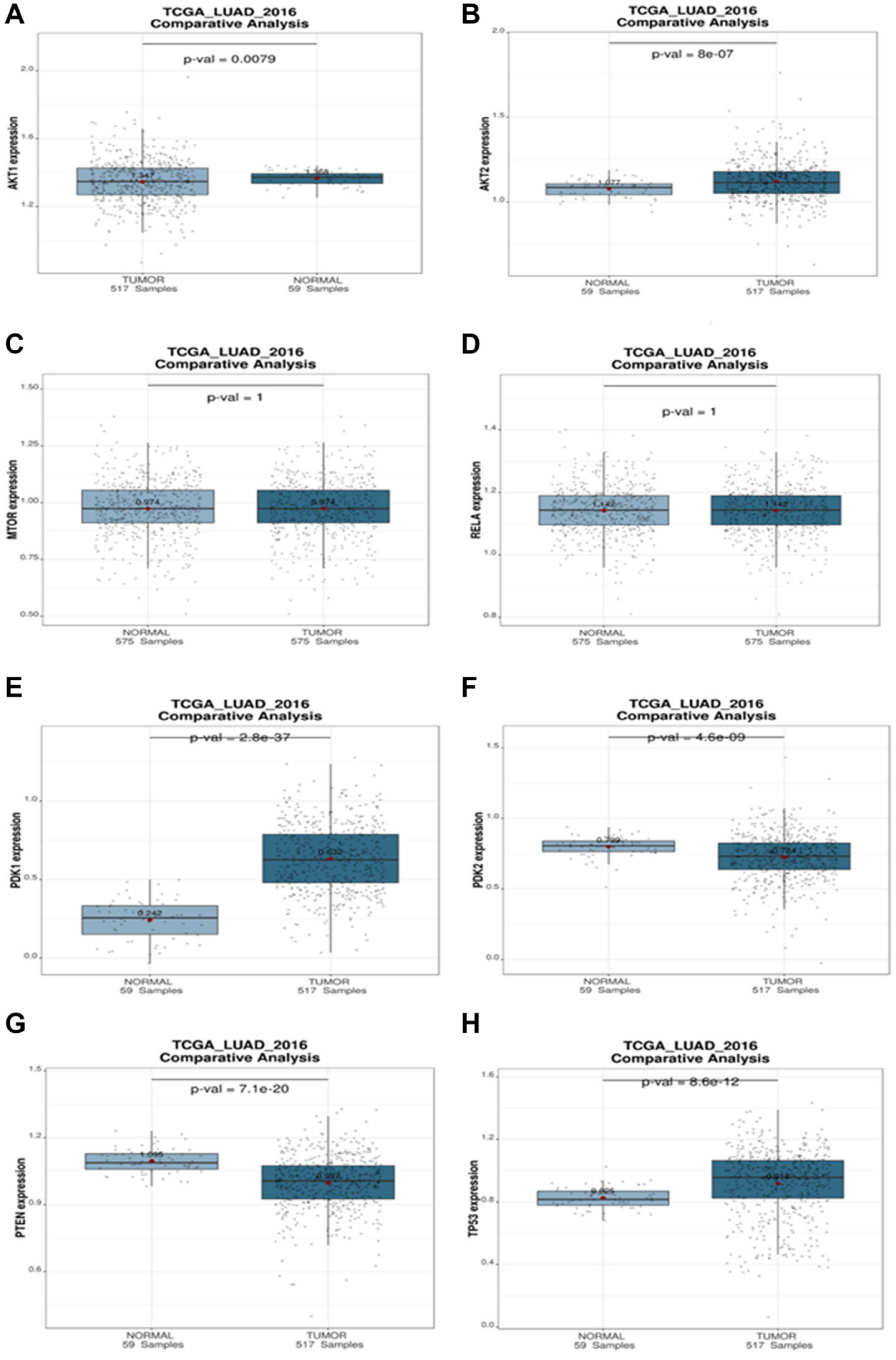
Comparative analysis plot (Normal vs. Tumor). The differential expression of (**A**) AKT1, (**B**) AKT2, (**C**) mTOR, (**D**) RELA, (**E**) PDK1, (**F**) PDK2, (**G**) PTEN, (**H**) TP53 targeted genes during NSCLC in different progression states ranging from tumors to normal. The statistical significance of differential expression of genes is shown as *p*-value.

A comparative analysis was used to investigate the connections between a targeted gene and clinical variables within a given dataset. Boxplots depict the expression levels of the targeted genes of miRNA in the patient groups, with *p* values for expression differences indicated. According to the boxplots analysis, tumor expression of AKT1, AKT2, and PDK1 is higher than the normal. There are no substantial changes in MTOR, NF-κB (RELA) expression. The expression of PDK2 and PTEN is lower in tumor than in normal. Because TP53 is mutated, it has a high expression level in NSCLC tumors (Figure 12).

### Synthetic circuit designed for the controlled elevation of expression of miR-520c-3p

**Figure 13:**
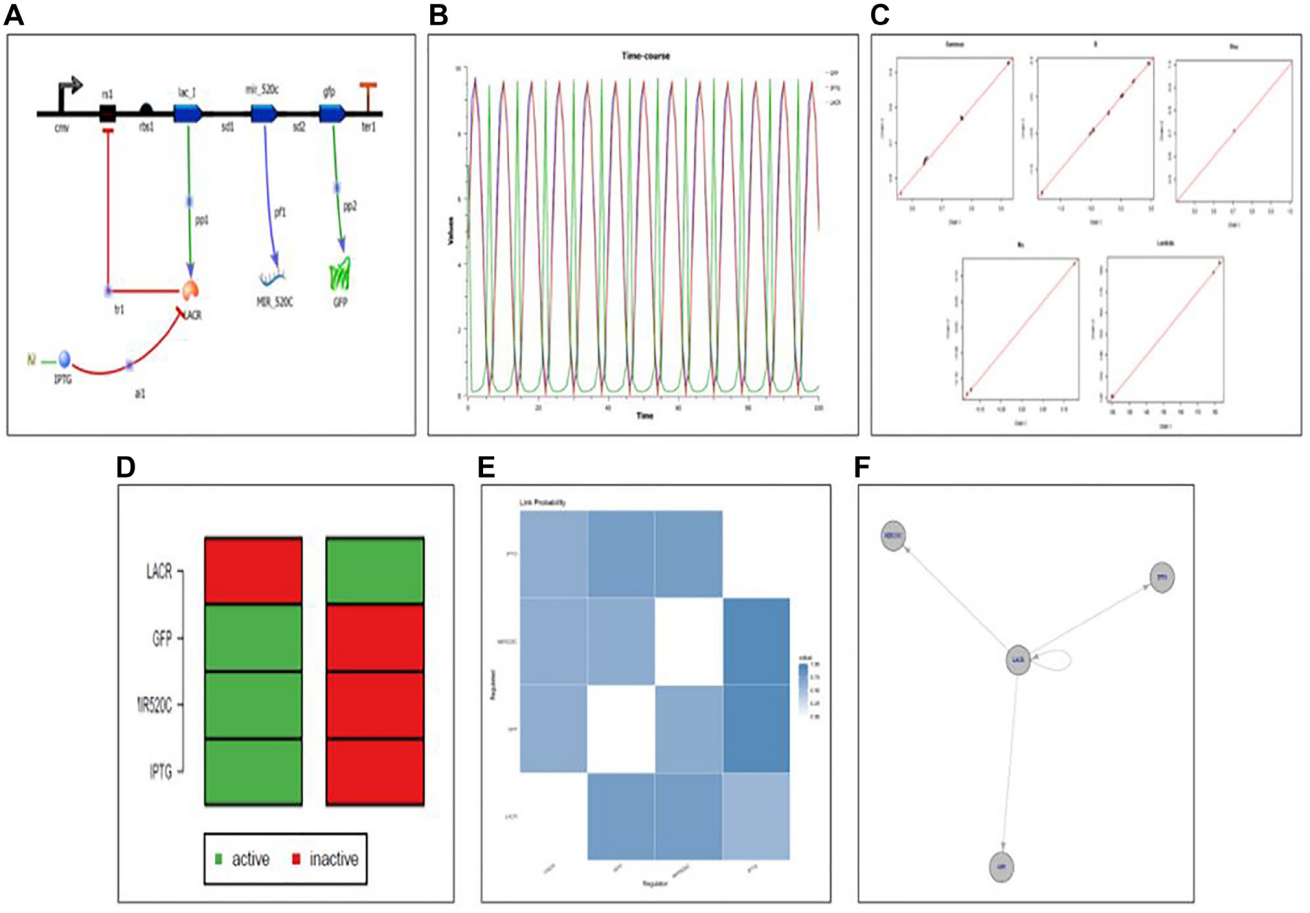
(**A**) Designed synthetic genetic circuit for miR-520c-3p. (**B**) Deterministic simulation of the designed genetic synthetic circuit. (**C**) Convergence plot of designed genetic synthetic circuit indicating all the parts in the pool is in working mechanistic aspect as all variables are on the line; Gamma: indicator variable of Gibbs variable selection, B: coefficient of linear regression, Lambda: precision of each regression, Mu: intercept of each regression. (**D**) Attractor state depicting the ON and OFF state of the synthetic circuit (**E**) Network uncertainty plot of the synthetic circuit depicting connections between the relation between repressor (Lac R) and expression of the genes ahead (miR-520c-3p and GFP). (**F**) Designed genetic synthetic circuit wired in a manner that makes Lac R the central switch.

The program Tinker cell was used to design a synthetic circuit for miR-520c-3p (Figure 13). A genetic circuit is made up of a genetic toggle switch and a repressilator. The toggle switch’s repressing genes encoded the repressor for the gene. The working mechanism of the designed circuit was based on the Lac operon system, where Lac R remains bonded to the operator region in the OFF state, inhibiting miR-520c-3p expression. Lac R binds to the inducer in the presence of an inducer (IPTG), putting the circuit in the ON state, expressing miR-520c-3p with GFP as the reporter protein (Figure 10). The orthogonality and modularity of the designed synthetic circuit were examined via Complex Pathway Simulator (COPASI), Gene Regulatory Network Inference Using Time Series (GRENITS). The Boolean technique was used to model qualitative networks using BoolNet [21].

The genetic circuit was simulated for 100-time points at different concentrations using deterministic simulation. The resulting variations in the protein level concerning change in dissociation constant (Kd) are addressed in two situations based on the Kd values (cases 1 and 2). In case 1, where the Kd value of Lac R was increased, which decreased the Kd value of miR-520c-3p and GFP protein showcasing the OFF state of the system. Case 2 is where the Kd value of IPTG was elevated/incorporated, which increased the Kd value of miR-520c-3p, GFP proteins and decreased the Kd value of Lac R, showing the ON state of the system. These findings suggest that the toggle switch is flipping; indicating that the built toggle switch made up of miR-520c-3p is bistable and that there is a direct connection between the miR-520c-3p and the toggle switch repressilator’s LacI.

#### Validation of the designed synthetic circuit

The model was validated using both qualitative and quantitative methods. The genetic circuit’s ODE model was created, providing insight into the circuit’s regulation process. The Bioconductor software created a gene regulatory network for the genetic circuit [22]. The time-series data for qualitative and quantitative network modeling was acquired and simulated using COPASI. The circuit model was stable; therefore, the linear network was built. The GRENITS software was used to do qualitative network modeling, which provided the likelihood of each regulator in the regulatory network circuit. Using default settings, the posterior probability was calculated using a Monte Carlo–Markov chain simulation. Two Markov chains were created during the simulation, and the network’s connection probability is calculated based on their convergence. The regulatory network circuit gave a probability matrix, analysis, and a convergence graphic. Network inference was observed for 10 and 100-time points to understand the regulatory mechanism in the circuit, where a probability of 1 indicates that there is regulation between the respective regulators. In contrast, a probability of 0 indicates that there is no regulation.

The connection probability between the Lac I belonging to the repressilator and miR-520c-3p of the genetic toggle switch was shown in the analysis plot of the genetic circuit, which offers insight into the switching behaviour. The color blue in the figure represents a probability of one. The probability of 1 between the two genes miR-520c-3p and Lac R is shown in the analysis plot. The Lac R and miR-520c-3p genes were likewise found to be related since the probability between them was also 1. The marginal network uncertainty graphic shows the top regulators in the circuit. The network uncertainty plot provides network uncertainty. miR-520c-3p and Lac R were the major regulators in the network model (Figure 13).

The best-fit method was used to create a Boolean network from time-series data. The Boolean network developed for miR-520c-3p has a total of two states. 1 (active state) and 0 (inactive state) symbolize the 2 states (inactive state). After observing state transitions, the network circuit reaches stable states (attractors). The bistability of the circuit created was shown by two attractors in the genetic circuit. The two stable states produced for each gene in the circuit reflects the ON and OFF states, with 0 indicating the OFF state and 1 indicating the ON state. The attractor plot manifests that during the OFF state, Lac R presence deprives the expression of miR-520c-3p and GFP; however, in the ON state, after incorporating IPTG, Lac R binds to IPTG, leaving the operator open for RNA polymerase to move forward and transcribe the genes ahead, i.e., miR-520c-3p and GFP (Figure 13). It shows each gene’s active and inactive states at various times. Further, the therapeutic aspect of the designed genetic circuit can be explored *in-vitro* and *in-vivo*.

## DISCUSSION

Several studies have reported on the involvement of miRNAs in cancer development or regression. The results have been acclimatized to the clinical application after much effort. The use of miRNA as a therapeutic intervention in the treatment of NSCLC might lead to innovative therapies for the disease. Therapy based on miRNA mimics or miRNA-mediated suppression of carcinogenic mRNA should yield promising outcomes with minimal side effects. Our study’s significant findings suggest and clarify the significance of miR-520c-3p as a possible innovative treatment for NSCLC.

The main advantage of miRNA as a therapeutic agent is that it may target many genes in redundant pathways to develop Non-Small Cell Lung Cancer. This result suggests that miRNA might be utilized to suppress pro-tumoral pathways, making it more attractive than a combination of siRNAs, which is already being employed as a treatment.

Furthermore, because of the small size of the miRNA sequence, mutations are sporadic, and resistance to miRNA treatments would almost certainly need many mutations in various genes. Furthermore, multiple studies have shown that even minor changes in the expression of miRNAs and their related targets may cause phenotypic changes, bolstering the hypothesis that correcting a small number of miRNAs might reverse the malignant phenotype.

**Figure 14:**
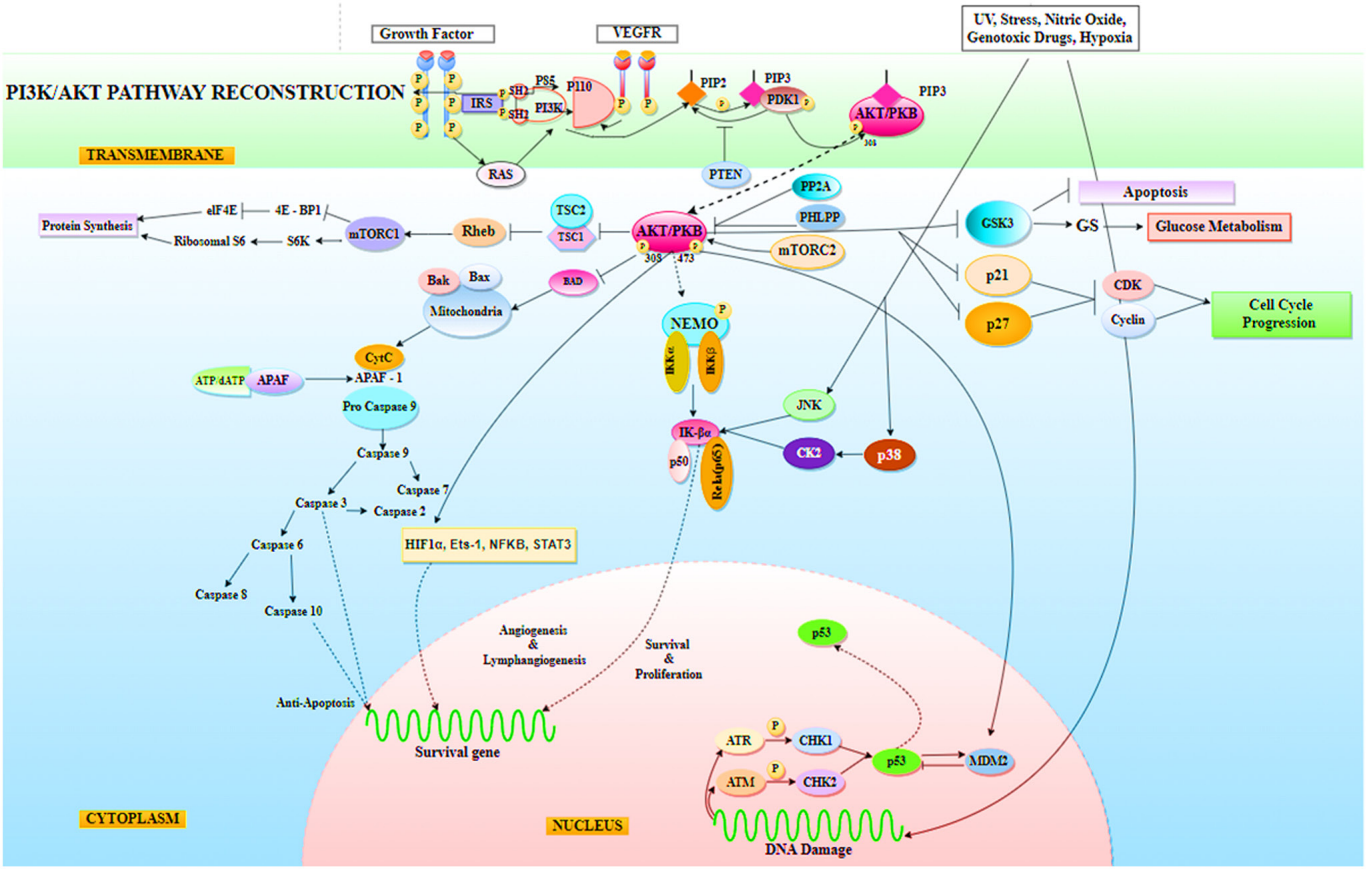
Representation of PI3K/AKT signaling pathway in Non-Small Cell Lung Cancer (NSCLC), where AKT has been found to mediate a variety of cellular processes required by tumor cells for survival, including protein synthesis, glucose metabolism, cell cycle progression, anti-apoptosis, tumor cell angiogenesis and lymphangiogenesis activity for survival, proliferation, metastasis, and invasion. Indirectly, AKT has been found to affect the tumor suppressor function of p53.

The purpose of the study was to look into the functioning of the miRNAs that were down-regulated during NSCLC. miR-520c-3p, miR-181b-1, miR-493-5p, let-7a-2, miR-92a-1-3p, miR-30b-5p, miR-106-5p, miR-1-3p, miR-15a, miR-125b-1-5p, miR-30d, miR-17/92, miR-182-3p, miR-9-5p, and miR-520a-3p were determined to be the key miRNAs after generating an inter-regulatory miRNA network for NSCLC. Furthermore, literature evidence and network processing revealed miR-520c-3p (downregulated) as the most prominent candidate. In other kinds of cancer, miR-520c-3p serves as an oncomiR, but it supports tumor suppression in NSCLC. Seed sequence analysis confirmed its participation in the PI3K/AKT/mTOR signaling pathway, which was predicted by pathway enrichment studies. The PI3K/AKT/mTOR pathway has been implicated in carcinogenesis and disease progression in NSCLC (Li X et al., 2019) (Figure 14). Another way to treat cancer is to provide tumor-suppressive miRNAs to cancer cells. To target tumor-promoting mRNAs, synthetic double-stranded miRNA mimics, pre-miR, or plasmid-encoded miRNA genes substitute for missing tumor-suppressor miRNAs. miR-520c-3p, which is aberrant in NSCLC, plays an important role in tumor suppression. The administration of miR-520c-3p mimics to cancer cells may have a growth inhibitory impact; making miRNA mimics a possible cancer therapeutic option.

Several miRNA therapeutic techniques, such as intratumoral injections and viral vector-mediated modulation of miRNA expression as delivery channels, are unlikely to be used in a clinical setting. Intratumoral injections could only be utilized for a small number of easily accessible tumors, as accessing the lungs with a tumor in NSCLC may appear hard. Similarly, using viral vectors to modulate miRNA expression might have some drawbacks to gene therapy, such as low infectivity and issues with gene product transcription. Furthermore, cancer cells cannot usually mature miRNA precursors, making viral vector expression as a less desirable option.

However, using mimics as therapy might be inconvenient in the future since there will be no way to control the expression of the desired miR-520c-3p. We attempted to leverage the functionality of miR-520c-3p as a therapeutic intervention by constructing a synthetic genetic circuit of miR-520c-3p that will act in a regulated manner. In the future, the developed genetic synthetic circuit may aid in tumor suppression by increasing miR-520c-3p expression in a controllable way, which will then target and block the PI3K/AKT/mTOR signaling pathway. The problems of miRNA treatment, on the other hand, must be addressed. miRNAs are unstable in the body due to numerous ribonucleases and RES clearance, as previously stated. Furthermore, their negative charges make it difficult for them to cross the cell membrane or the vascular endothelium. Even if they reach the interior of a cell, they are degraded by endolysosomes. It is critical not to damage healthy tissue to enable optimal cancer cell-specific delivery. The tumor micro-environment acts as a barrier and inhibits effective miRNA transport, whereas impaired blood perfusion in tumors reduces systemic delivery of miRNAs. Tumor-associated immune cells (macrophages, neutrophils, and monocytes) can absorb miRNAs encapsulated in the delivery system non-specifically in the tumor micro-environment. More studies will be needed to attempt new techniques in delivering therapeutic miRNA-built genetic synthetic circuits to solve these issues.

The obstacles to future miRNA therapeutic development, such as improving miRNA stability, delivery, and regulation of off-target effects, must be addressed. We expect some of the miRNA techniques mentioned above to be further improved to increase specificity and efficacy. These will eventually be utilized to treat lung cancer patients, either alone or in conjunction with chemotherapy.

## MATERIALS AND METHODS

### Collection of data

Data on miRNAs linked to NSCLC was gathered from publicly available databases such as miRTV, miRBase, and scientific literature searches and then confirmed using the TCGA database. The literature review dataset was created for downregulated miRNAs in NSCLC and their corresponding targets [23–25].

#### Construction of an inter-regulatory miRNA-NSCLC associated network

We built the bipartite network in this study by mapping pairs of miRNA-NSCLC interactions and visualizing it with Cytoscape v3.8.2. The miRNAs with their respective target attributes to the nodes and their interaction is represented as edges. Consequently, the miRNA-NSCLC association network that was created offers information on the role of miRNA in NSCLC. The network was constructed based on a dataset for downregulated miRNAs with targets in NSCLC. Cytoscape is modular, and applications may extend networks with new features (known as plugins) [26].

### Gene ontology, StringApp, enrichment map, and CytoHubba plugins

CytoHubba was used to evaluate the created networks [27]. The top ten nodes in CytoHubba were selected depending on the metrics (local and global). The Cytoscape plugin program BiNGO v3.0.3 collected GO annotations for the targeted genes identified [28]. BiNGO retrieved the overrepresentation of GO categories in a subgraph of a biological network, which is displayed on Cytoscape, using the input list of targeted genes. The Benjamini and Hochberg correction was used to give tight control over the false discovery rate under positive regression dependence of the test statistics, and the hypergeometric test *P*-value was adjusted at 0.05. The GO hierarchy was displayed as overrepresented GO categories after statistical analysis. StringApp plugin in Cytoscape was used to obtain miRNA–gene interaction data [29]. We considered connections with an edge interaction confidence cut-off of > 0.4 (medium confidence), as indicated by the StringApp, with 1 being the highest possible confidence and 0 being the lowest.

The Enrichment Map Cytoscape plugin loaded gene-set definition and enrichment table files, and then filtered for significance using the user-defined *p*-value and FDR criteria [30, 31]. The Jaccard coefficient and overlap coefficient was used to calculate the overlap between important gene sets and miRNAs.

### Cell-line

The BEAS-2B (normal human bronchial epithelial cell line) obtained from American Type Culture Collection (ATCC) was used in this study. It was maintained in BEGM^™^ Bronchial Epithelial Cell Growth Medium BulletKit^™^. A549 human non-small cell lung carcinoma (NSCLC) cell lines obtained from Cell Repository, National Centre for Cell Science, Pune. It was cultured in Ham’s F-12K (Kaighn’s) Medium, supplemented with 10% fetal bovine serum (FBS), penicillin (100 U/mL), and streptomycin (100 mg/mL). Both the cells were maintained and cultured in a humidified incubator containing 5% CO_2_ at 37°C conditions.

### Transcriptome sequencing

A549 cells (lung adenoma-carcinoma) and BEAS-2B cells were used to extract RNA, build a cDNA library, and perform whole transcriptome sequencing. For the RNA samples’ preparation, 3 μg of RNA per sample was utilized as the input material. Illumina Novoseq was used to generate the sequencing data. FastQC and MultiQC applications were used to assess the data quality.

### Gene-set enrichment analysis

GSEA was used to quantify the significance of multiple perturbations [32], cell transcriptional responses or perturbations, and massive gene expression profiling datasets for miR-520c-3p in NSCLC (BEAS-2B vs. A549 cells). GMT, CLS, and GCT files were created as input files for GSEA analysis using gene expression profiles of miR-520c-3p in NSCLC (BEAS-2B vs. A549 cells), gave information about the expression of miR-520c-3p in NSCLC.

### Pathway enrichment analysis

DIANA-miRPath v3.0 package analyzed miRNA regulatory functions and identified regulated pathways. The DIANA-miRPath v3.0 database and capabilities have been greatly expanded to include all KEGG molecular pathway studies (http://www.microrna.gr/miRPathv3) [33]. The selected miRNAs from network analysis were loaded for analyzing their enrichment in the particular molecular/signaling pathways. The webserver used absolute *P*-values (option: ‘Significance Clusters’). A heat map of miRNAs vs. pathways was generated, representing the relationship and pattern between selected miRNAs with their involvement in particular pathways examined regarding NSCLC.

### Sequence-based miR-520c-3p target prediction analysis via TargetScan, KMP algorithm, and Tools4miRs

Tools4miRs helped in exploring the provided data using designated target prediction algorithms. The server was used to check the involvement of miR-520c-3p in particular pathways chosen [34]. The TargetScan was utilized for the target prediction of miR-520c-3p. The locations with a more significant and lower chance of targeting miRNAs are shown for each transcript [35]. This likelihood was calculated by combining all methods and factors for each miRNA candidate (site type, context++ score, context++ score percentile, weighted context++ score, conserved branch length, and PCT). TargetScan was used to calculate the expected frequency of matching to the 3′ end of the miRNA, the expected frequency of seed matches in the 3′ UTR dataset. An optimized base pairing of the remaining 3′ portion of the miRNA to the 35 bases of the UTR immediately 5′ from the seed match, the predicted free energy of a seed: seed match duplex (kcal/mol), and the observed count of seed matches in the 3′ UTR dataset were also calculated. Complementary base-pairing of miR-520c-3p seed sequence with targets selected from pathway enrichment was done with TargetScan.

The KMP (Knuth Morris Pratt) matching method reduces the worst-case complexity to O(n) by utilizing the pattern’s degenerating property (pattern with the same sub-patterns appearing more than once in the pattern). KMP’s method is based on detecting a mismatch (after some matches) [21]. For evaluation of the existing sequence for base-pairing with seed sequence of miR-520c-3p in various variants of transcripts of the targets selected based on pathway enrichment analysis, the KMP algorithm was utilized.

### Estimation of survival via Kaplan-Meier plot

Considering censored data, it is critical in clinical trials to robustly and adequately assess the proportion of patients that survive after a particular period of therapy. The prognostic value of mRNA expression of topoisomerase family genes in NSCLC was assessed using the Kaplan-Meier plotter (https://www.kmplot.com/), an online database incorporating gene expression and clinical data. This database contains lung cancer, ovarian cancer, stomach cancer, and breast cancer statistics. The Kaplan-Meier estimate is the simplest and most successful method for calculating survival rates in this situation. The log-rank test determines the significance of differences in survival distributions between two or more groups of participants. If the *p*-value for the log-rank test is less than 0.05, the survival outcomes of the two groups are regarded as substantially different [36]. To summarize, each of the eight genes (AKT1, AKT2, PDK1, PDK2, NF-κB, PI3K, PTEN & TP53) was entered into the database Kaplan-Meier survival graphs were created, with the number of persons at risk given underneath the prominent figure. The hazard ratio (HR), log-rank *p*-value, and 95% confidence intervals were computed and shown on the website.

#### Survival analysis

Survival analysis plot was retrieved from online database The Cancer Proteome Atlas (TCPA, http://tcpaportal.org) which consists of independent patient cohort dataset. The Cancer Genome Atlas (TCGA) describes approximately 8,000 patient samples, and the current RPPA platform comprises over 300 protein markers that span all main cancer signaling pathways [37].

#### Comparative analysis

Lung Cancer Explorer (LCE), a web application powered by a consolidated lung cancer database (http://lce.biohpc.swmed.edu/), was used to get the normal vs. tumor comparison. The TCGA LUAD 2016 cohort was used to examine the expression of miRNA-targeted genes in both normal and tumor circumstances during NSCLC [38].

### Designing the genetic synthetic circuit for PI3K/AKT signaling network rewiring aiding in tumor suppression

Tinker Cell, the computer-aided design software for synthetic biology (http://www.tinkercell.com), created the genetic circuit. FASTA or Genbank sequences were used as inputs. Parts came from the Registry of Biological Parts (http://parts.igem.org/Main_Page), a database of genetic components that may be combined matched to create synthetic biology devices and systems. A mature miR-520c-3p sequence was retrieved from miRBase. Each of the three genes that make up the repressilator was given a transcription repression response. Protein degradation, promoter strength, transcription rate, dissociation constants, Hill’s coefficient, and reaction rates were set for the reaction. To examine the behaviour of the designed circuit, it was subjected to deterministic simulation [39].

### Validation of model

The model validation was carried out using the Bioconductor package, which comprises two packages: (a) Gene Regulatory Network Inference Using Time Series (GRENITS) and (b) Boolnet. The validation time-series data was collected from the Complex Pathway Simulator utilizing the time course approach for deterministic simulation. The Boolean technique was used to model qualitative networks using BoolNet. BoolNet is a tool for integrating synchronous, asynchronous, and probabilistic Boolean network techniques. Aside from reconstructing networks using time series data, robustness analysis may be performed using perturbation and Markov chain simulations, identifying and visualizing attractors. The Bayesian method was used for quantitative network modeling, and the GRENITS package was used to exploit the time-series data provided by COPASI. The likelihood of the genes included in the circuit, the regulators of the circuit, and the network uncertainty were calculated using ODE time-series data [40].

### Statistical analysis

R 3.5.2 (https://www.r-project.org/) and GraphPad Prism version 8 for Windows, GraphPad Software (San Diego, California USA, https://www.graphpad.com/) were used for the statistical analysis. The Kaplan-Meier survival analysis was used to determine the prognostic value. Both univariate and multivariate Cox regression analyses were used to verify independent prognostic factors. The differential expression was examined using the Student’s *t*-test.

## CONCLUSIONS

In this study, miR-520c-3p was a new tumor suppressor miRNA identified in NSCLC. The heatmap analysis revealed that it might have a functional role in NSCLC by targeting the defective EGFR and PI3K/AKT-driven signaling pathway. AKT1 and AKT2 were identified as possible targets of miR-520c-3p based on target prediction and seed sequence analysis. AKT’s promote biological activities such as protein synthesis, glucose metabolism, cell cycle progression, anti-apoptosis, angiogenesis, and lymphangiogenesis, which are necessary for tumor cell survival, proliferation, metastasis, and invasion. AKT has also been discovered to affect the tumor suppressor function of p53 indirectly. miR-520c-3p can regulate the overexpression of AKT genes and their downstream processing in the EGFR pathway, triggering death in tumor cells, restoring the function of tumor suppressor p53, and decreasing protein synthesis for cell survival. The Cancer Genome Atlas (TCGA) database data revealed a negative association between AKT1, AKT2, and miR-520c-3p mRNA levels. Elevating the expression of miR-520c-3p in a regulated manner using a developed genetic synthetic circuit is novel as an RNA therapy might help suppress tumors in NSCLC in the future by blocking PI3K/AKT/MTOR signaling pathway in a controlled manner.

## SUPPLEMENTARY MATERIALS











## Abbreviations

ALK: Anaplastic lymphoma kinase
COPASI: Complex Pathway Simulator
ROS1: c-ros oncogene 1
ES: Enrichment score
EGFR: Epidermal growth factor receptor
GRENITS: Gene Regulatory Network Inference Using Time Series
mTOR: Mechanistic target of rapamycin
NSCLC: Non-small cell lung cancer
PI3K: Phosphatidylinositol-3-kinase
