# Supplementary material for: Deciphering miR-520c-3p as a probable target for immunometabolism in non-small cell lung cancer using systems biology approach
Source: Oncotarget. 2022 May 24;13:725–46. doi: 10.18632/oncotarget.28233 (PMC9131939; doi:10.18632/oncotarget.28233)
Supplement: Supplementary file 1 [file oncotarget-13-28233-s001.pdf]

Deciphering miR-520c-3p as a probable target for immunometabolism in non-small cell lung cancer using systems biology approach

SUPPLEMENTARY MATERIALS

Supplementary Material 1: KMP algorithm

```
# Python program for KMP Algorithm
import sys
fileNameTranscript = "Transcript.txt" # Name of the
transcript file
fileNamePrefix = "trs" #Name of transcript files without
number
startIndex = 1 # Start number of transcript file
EndIndex = 36 # End number of transcript file
#Read transcript from file
f Transcript = open(fileName Transcript, 'r')
txt = fTranscript.readline()
#print(txt)
fTranscript.close()
EndIndex += 1 # Increment the end index by 1 to cover
last index
for x in range(startIndex, EndIndex):
 inputFileName = fileNamePrefix + str(x) + ".txt"
 #print("Input file name : " + inputFileName)
 fInput = open(inputFileName, 'r')
 searchHeader = fInput.readline()
 searchString = fInput.readline()
 fInput.close()
 #print("Input search String : " + searchString)
 #print(searchString)
 pat = searchString
   outputFileName = "SequenceFoundFor-" +
inputFileName
 #print("Output file name : " + outputFileName)
 f = open(outputFileName,'w')
 # write the search string in output file
 f.write("Found pattern for - \n" + searchHeader + "\n")

 def KMPSearch(pat, txt):
 M = len(pat)
 N = len(txt)

 # create lps[] that will hold the longest prefix suffix
S2
 # values for pattern
 lps = [0]*M
 j = 0 # index for pat[]

# Preprocess the pattern (calculate lps[] array)
computeLPSArray(pat, M, lps)

i = 0 # index for txt[]
while i < N:
if pat[j] == txt[i]:
i += 1
j += 1

if j == M:
#print >> f, "Found pattern AAAGTGCA at index " +
str(i-j)
f.write("at index " + str(i-j) + "\n")
j = lps[j-1]

# mismatch after j matches
elif i < N and pat[j] != txt[i]:
# Do not match lps[0..lps[j-1]] characters,
# they will match anyway
if j != 0:
j = lps[j-1]
else:
i += 1

def computeLPSArray(pat, M, lps):
len = 0 # length of the previous longest prefix suffix

lps[0] # lps[0] is always 0
i = 1

# the loop calculates lps[i] for i = 1 to M-1
while i < M:
if pat[i]==pat[len]:
len += 1
lps[i] = len
i += 1
else:
# This is tricky. Consider the example.
# AAACAAAA and i = 7. The idea is similar
# to search step.
if len != 0:
len = lps[len-1]
```

```
# Also, note that we do not increment i here
else:
lps[i] = 0
i += 1
KMPSearch(pat, txt)
f.close()
#
```

Supplementary Table 1: CytoHubba rank table. See Supplementary Table 1

Supplementary Table 2: Bingo analysis table of identified miRNAs with their putative targets. See Supplementary Table 2

Supplementary Table 3: String app analysis of identified miRNAs with their respective target. See Supplementary Table 3

Supplementary Table 4: Normalized_Expression_Counts. See Supplementary Table 4

Supplementary Table 5: Results of Tools4miR for identified miRNAs

AKT1

	mrna	mirna	microtar	miranda	mirmap	pita	rna22	targetspy	rnahybrid	guugle	binding_sites	no_tools
0	ENST00000349310.7	hsa-let-7a-2-3p	FALSE	FALSE	FALSE	TRUE	TRUE	FALSE	FALSE	FALSE	8	2
1	ENST00000349310.7	hsa-let-7a-5p	TRUE	FALSE	FALSE	TRUE	TRUE	FALSE	FALSE	FALSE	7	3
2	ENST00000349310.7	hsa-miR-1-3p	TRUE	FALSE	FALSE	FALSE	FALSE	FALSE	FALSE	FALSE	1	1
3	ENST00000349310.7	hsa-miR-1-5p	FALSE	FALSE	FALSE	TRUE	FALSE	FALSE	FALSE	FALSE	2	1
4	ENST00000349310.7	hsa-miR-15a-3p	FALSE	FALSE	TRUE	TRUE	TRUE	FALSE	FALSE	FALSE	5	3
5	ENST00000349310.7	hsa-miR-15a-5p	FALSE	FALSE	TRUE	TRUE	FALSE	FALSE	FALSE	FALSE	4	2
6	ENST00000349310.7	hsa-miR-17-3p	FALSE	FALSE	FALSE	TRUE	FALSE	FALSE	FALSE	FALSE	9	1
7	ENST00000349310.7	hsa-miR-17-5p	TRUE	TRUE	FALSE	TRUE	TRUE	FALSE	FALSE	FALSE	8	4
8	ENST00000349310.7	hsa-miR-181b-3p	FALSE	FALSE	FALSE	TRUE	FALSE	FALSE	FALSE	FALSE	4	1
9	ENST00000349310.7	hsa-miR-181b-5p	FALSE	FALSE	FALSE	TRUE	TRUE	TRUE	FALSE	FALSE	3	3
10	ENST00000349310.7	hsa-miR-30b-3p	TRUE	FALSE	FALSE	TRUE	FALSE	FALSE	FALSE	FALSE	6	2
11	ENST00000349310.7	hsa-miR-30b-5p	FALSE	FALSE	FALSE	TRUE	FALSE	FALSE	FALSE	FALSE	2	1
12	ENST00000349310.7	hsa-miR-493-3p	TRUE	TRUE	FALSE	TRUE	TRUE	FALSE	FALSE	FALSE	11	4
13	ENST00000349310.7	hsa-miR-493-5p	FALSE	FALSE	FALSE	TRUE	TRUE	FALSE	FALSE	FALSE	3	2
14	ENST00000349310.7	hsa-miR-520c-3p	TRUE	TRUE	FALSE	TRUE	TRUE	FALSE	FALSE	FALSE	11	4
15	ENST00000349310.7	hsa-miR-520c-5p	FALSE	FALSE	FALSE	TRUE	TRUE	FALSE	FALSE	FALSE	6	2
16	ENST00000349310.7	hsa-miR-92a-1-5p	TRUE	FALSE	FALSE	TRUE	TRUE	FALSE	FALSE	FALSE	12	3
17	ENST00000349310.7	hsa-miR-92a-2-5p	TRUE	FALSE	FALSE	TRUE	TRUE	FALSE	FALSE	FALSE	17	3
18	ENST00000349310.7	hsa-miR-92a-3p	FALSE	FALSE	FALSE	TRUE	FALSE	FALSE	FALSE	FALSE	1	1

PI3K

	mrna	mirna	microtar	miranda	mirmap	pita	rna22	targetspy	rnahybrid	guugle	binding_sites	no_tools
0	ENST00000263967.4	hsa-let-7a-2-3p	FALSE	TRUE	FALSE	TRUE	TRUE	FALSE	FALSE	FALSE	19	3
1	ENST00000263967.4	hsa-let-7a-5p	TRUE	TRUE	FALSE	TRUE	TRUE	TRUE	FALSE	FALSE	25	5
2	ENST00000263967.4	hsa-miR-1-3p	TRUE	TRUE	FALSE	TRUE	FALSE	TRUE	FALSE	FALSE	20	4
3	ENST00000263967.4	hsa-miR-1-5p	FALSE	TRUE	FALSE	TRUE	FALSE	TRUE	FALSE	FALSE	23	3
4	ENST00000263967.4	hsa-miR-15a-3p	TRUE	TRUE	TRUE	TRUE	FALSE	TRUE	FALSE	FALSE	16	5
5	ENST00000263967.4	hsa-miR-15a-5p	TRUE	TRUE	TRUE	TRUE	FALSE	FALSE	FALSE	FALSE	22	4
6	ENST00000263967.4	hsa-miR-17-3p	TRUE	TRUE	FALSE	TRUE	TRUE	TRUE	FALSE	FALSE	13	5
7	ENST00000263967.4	hsa-miR-17-5p	FALSE	TRUE	FALSE	TRUE	FALSE	TRUE	FALSE	FALSE	23	3
8	ENST00000263967.4	hsa-miR-181b-3p	TRUE	TRUE	FALSE	TRUE	FALSE	TRUE	FALSE	FALSE	18	4
9	ENST00000263967.4	hsa-miR-181b-5p	TRUE	TRUE	FALSE	TRUE	FALSE	TRUE	FALSE	FALSE	28	4
10	ENST00000263967.4	hsa-miR-30b-3p	TRUE	FALSE	FALSE	TRUE	TRUE	TRUE	FALSE	FALSE	22	4
11	ENST00000263967.4	hsa-miR-30b-5p	TRUE	TRUE	FALSE	TRUE	FALSE	TRUE	FALSE	FALSE	28	4
12	ENST00000263967.4	hsa-miR-493-3p	TRUE	TRUE	FALSE	TRUE	TRUE	TRUE	FALSE	FALSE	24	5
13	ENST00000263967.4	hsa-miR-493-5p	TRUE	FALSE	FALSE	TRUE	FALSE	TRUE	FALSE	FALSE	17	3
14	ENST00000263967.4	hsa-miR-520c-3p	FALSE	TRUE	FALSE	TRUE	FALSE	TRUE	FALSE	FALSE	23	3
15	ENST00000263967.4	hsa-miR-520c-5p	TRUE	TRUE	FALSE	TRUE	TRUE	TRUE	FALSE	FALSE	19	5
16	ENST00000263967.4	hsa-miR-92a-1-5p	TRUE	TRUE	FALSE	TRUE	TRUE	FALSE	FALSE	FALSE	19	4
17	ENST00000263967.4	hsa-miR-92a-2-5p	TRUE	FALSE	FALSE	TRUE	TRUE	FALSE	FALSE	FALSE	19	3
18	ENST00000263967.4	hsa-miR-92a-3p	TRUE	TRUE	FALSE	TRUE	TRUE	TRUE	FALSE	FALSE	17	5
