# Supplementary material for: Deciphering miR-520c-3p as a probable target for immunometabolism in non-small cell lung cancer using systems biology approach
Source: Oncotarget. 2022 May 24;13:725–46. doi: 10.18632/oncotarget.28233 (PMC9131939; doi:10.18632/oncotarget.28233)
Supplement: Supplementary file 3 [file oncotarget-13-28233-s003.docx]

**Supplementary Table 2: Bingo analysis table of identified miRNAs with their putative targets**

| GO-ID | p-value | corr p-value | x | n | X2 | N3 | Description | Genes in test set |
| --- | --- | --- | --- | --- | --- | --- | --- | --- |
| 9653 | 1.38E-10 | 1.81E-07 | 52 | 1217 | 237 | 14294 | anatomical structure morphogenesis | SEMA5A\|CDKN1A\|BMPR2\|CITED2\|HHIP\|PTEN\|SLC40A1\|TCF21\|NDST1\|EDNRA\|GJA1\|EDNRB\|ZMIZ1\|TIMP3\|SOX6\|EDN1\|ANXA2\|SOX13\|TGFBR1\|DKK3\|TGFBR2\|TGFBR3\|ULK2\|PDE5A\|GAS7\|HLF\|SDC2\|TGFB\|GATA6\|PDGFA\|ADRB1\|ADRB2\|THBS1\|DLL4\|PARD6B\|BCL2L11\|MFN2\|DMD\|GPC4\|SLIT2\|EGR2\|UBE2B\|TIPARP\|KLF4\|PBX1\|SMAD7\|DAB2\|NR4A3\|BCL6\|GNAQ\|BCL2\|PNPLA6 |
| 23052 | 1.45E-10 | 1.81E-07 | 95 | 3127 | 237 | 14294 | signaling | SEMA5A\|CD86\|ITGB1\|CDKN1A\|BMPR2\|MAML2\|RTKN2\|PTEN\|PTPRG\|SLC6A4\|AKAP12\|NDST1\|RPS6KA3\|PPP3CA\|EDNRA\|GJA1\|EDNRB\|RASSF2\|RASSF3\|DPYSL2\|AKT2\|RPS6KA2\|PDK4\|RSPO2\|AKT1\|SOSTDC1\|JAK1\|KLF10\|EDN1\|DAPK1\|FBXW11\|PRKCE\|ANXA4\|MAGI3\|TMOD2\|SH2D3C\|GAB2\|TNFRSF1B\|SREBF2\|TGFBR1\|TGFBR2\|APBB1IP\|RAB32\|TGFBR3\|LATS2\|DDAH1\|ULK2\|RAPGEF2\|PDE5A\|ARHGEF6\|PTGER4\|HLF\|RRAD\|FGL2\|TGFB\|NTN4\|PDGFA\|ADRB1\|ADRB2\|LTBP3\|THBS1\|NKD1\|CORO1C\|DLL4\|RASD1\|WIF1\|MKNK2\|S1PR1\|DMD\|SLIT2\|LEFTY2\|PAG1\|LYN\|MAP3K3\|PTPN1\|EGR2\|UBE2B\|STAC\|FZD4\|TIPARP\|DAB2IP\|RASSF8\|ARHGAP28\|HIPK1\|RAB11A\|SMAD7\|KLF6\|CD4\|ARHGAP30\|RAB14\|STK17B\|GNAQ\|KLF9\|KRAS\|RGL1 |
| 50794 | 2.20E-10 | 1.82E-07 | 151 | 6218 | 237 | 14294 | regulation of cellular process | CD86\|MAML2\|HHIP\|RTKN2\|NIRF\|ETS1\|NDST1\|PREX1\|RPS6KA3\|EDNRA\|GJA1\|EDNRB\|RASSF2\|RASSF3\|ZMIZ1\|DPYSL2\|AKT2\|RPS6KA2\|PDK4\|AKT1\|SOX6\|PKNOX2\|BNIP3L\|MBNL2\|DAPK1\|LIN28\|PRKCE\|TMOD2\|LMO2\|SOX13\|TBC1D2B\|DKK3\|RAB32\|ULK2\|UTRN\|DDX5\|RRAD\|ABHD2\|TGFB\|GATA6\|NEDD4L\|PDGFA\|ADRB1\|ADRB2\|GATA2\|ZBTB4\|CORO1C\|BCL2L11\|RAD21\|TPPP\|PAG1\|MCL1\|LYN\|CBX7\|EGR2\|UBE2B\|STAC\|RDX\|RASSF8\|ARHGAP28\|PBX1\|SMAD7\|DAB2\|ARHGAP30\|NR4A3\|BCL6\|GNAQ\|BCL2\|RGL1\|FERMT2\|ITM2B\|BCL2L2\|PHF2\|CDKN1A\|BTG2\|BTG1\|BMPR2\|CITED2\|PTEN\|TCF21\|TNFAIP1\|AFF3\|PTPRG\|AKAP12\|PPP3CA\|TMSB4X\|TIMP3\|ADAMTS8\|JAK1\|KLF10\|EDN1\|ANXA2\|ARHGEF15\|ANXA4\|SH2D3C\|ETV1\|GAB2\|TNFRSF1B\|SREBF2\|TGFBR1\|TGFBR2\|APBB1IP\|TGFBR3\|KAT2B\|ELF1\|TBC1D2\|LATS2\|CRY2\|RAPGEF2\|NCOA7\|PDE5A\|JDP2\|ARHGEF6\|GAS7\|PTGER4\|HLF\|ZBTB47\|RGS16\|FGL2\|LAMA3\|GLRX\|THBS1\|DLL4\|FGD5\|PARD6B\|RASD1\|WIF1\|MKNK2\|TP53INP1\|MFN2\|DMD\|SLIT2\|MAP3K3\|PTPN1\|PCGF5\|SPRY4\|DAB2IP\|HIPK1\|KLF4\|HIPK3\|RAB11A\|PNRC2\|KLF6\|CD4\|TEF\|RAB14\|STK17B\|KLF9\|KRAS\|CHPT1\|CDKN3 |
| 32502 | 3.87E-10 | 1.85E-07 | 96 | 3231 | 237 | 14294 | developmental process | SEMA5A\|CD86\|TRIM71\|ITGB1\|CDKN1A\|BTG2\|BMPR2\|CITED2\|HHIP\|RTKN2\|PTEN\|SLC40A1\|TCF21\|NIRF\|TNFAIP1\|AFF3\|PTPRG\|SLC6A4\|SYNE1\|NDST1\|RPS6KA3\|PPP3CA\|EDNRA\|GJA1\|EDNRB\|ZMIZ1\|DPYSL2\|AKT1\|TIMP3\|SOX6\|KLF10\|EDN1\|ANXA2\|LIN28\|SEMA6D\|TMOD2\|LMO2\|SOX13\|GAB2\|NPNT\|SREBF2\|TGFBR1\|DKK3\|TGFBR2\|TGFBR3\|ULK2\|PDE5A\|UTRN\|GAS7\|HLF\|AHNAK\|SDC2\|LAMA3\|TGFB\|GATA6\|NTN4\|PDGFA\|ADRB1\|ADRB2\|LTBP3\|GATA2\|THBS1\|PAPSS2\|DLL4\|PARD6B\|BCL2L11\|WIF1\|MFN2\|DMD\|GPC4\|SLIT2\|LEFTY2\|MCL1\|LYN\|EGR2\|UBE2B\|FZD4\|TIPARP\|SPRY4\|HIPK1\|KLF4\|PBX1\|SMAD7\|DAB2\|SNRK\|KLF6\|CD4\|NR4A3\|OSTM1\|BCL6\|GNAQ\|KLF9\|BCL2\|KRAS\|PNPLA6\|ITM2B |
| 48856 | 4.37E-10 | 1.85E-07 | 84 | 2654 | 237 | 14294 | anatomical structure development | SEMA5A\|ITGB1\|CDKN1A\|BTG2\|BMPR2\|CITED2\|HHIP\|RTKN2\|PTEN\|SLC40A1\|TCF21\|PTPRG\|SLC6A4\|SYNE1\|NDST1\|RPS6KA3\|PPP3CA\|EDNRA\|GJA1\|EDNRB\|ZMIZ1\|DPYSL2\|AKT1\|TIMP3\|SOX6\|KLF10\|EDN1\|ANXA2\|LIN28\|SEMA6D\|TMOD2\|LMO2\|SOX13\|GAB2\|TGFBR1\|DKK3\|TGFBR2\|TGFBR3\|ULK2\|PDE5A\|UTRN\|GAS7\|HLF\|AHNAK\|SDC2\|LAMA3\|TGFB\|GATA6\|NTN4\|PDGFA\|ADRB1\|ADRB2\|LTBP3\|GATA2\|THBS1\|PAPSS2\|DLL4\|PARD6B\|BCL2L11\|MFN2\|DMD\|GPC4\|SLIT2\|LEFTY2\|LYN\|EGR2\|UBE2B\|FZD4\|TIPARP\|KLF4\|PBX1\|SMAD7\|DAB2\|SNRK\|KLF6\|CD4\|NR4A3\|OSTM1\|BCL6\|GNAQ\|BCL2\|KRAS\|PNPLA6\|ITM2B |
| 51270 | 4.47E-10 | 1.85E-07 | 21 | 238 | 237 | 14294 | regulation of cellular component movement | LYN\|EDN1\|BMPR2\|CITED2\|ABHD2\|LAMA3\|TGFB\|PTEN\|PDGFA\|ETS1\|THBS1\|TGFBR1\|SMAD7\|TGFBR3\|DLL4\|PARD6B\|BCL6\|AKT2\|BCL2\|AKT1\|SLIT2 |
| 35295 | 8.49E-10 | 2.97E-07 | 23 | 297 | 237 | 14294 | tube development | SEMA5A\|EDN1\|BMPR2\|HLF\|HHIP\|TGFB\|GATA6\|PDGFA\|TCF21\|ADRB1\|ADRB2\|PBX1\|TGFBR1\|SMAD7\|TGFBR2\|EDNRA\|GJA1\|EDNRB\|BCL2L11\|NR4A3\|BCL2\|PDE5A\|SLIT2 |
| 1944 | 9.53E-10 | 2.97E-07 | 22 | 273 | 237 | 14294 | vasculature development | SEMA5A\|EDN1\|HLF\|ANXA2\|TIPARP\|CITED2\|LMO2\|LAMA3\|PTEN\|PDGFA\|TCF21\|THBS1\|TGFBR1\|SMAD7\|TGFBR2\|DLL4\|EDNRA\|GJA1\|ZMIZ1\|AKT1\|PNPLA6\|SLIT2 |
| 48534 | 2.09E-09 | 5.78E-07 | 21 | 259 | 237 | 14294 | hemopoietic or lymphoid organ development | LYN\|ITGB1\|HLF\|TIPARP\|RTKN2\|LMO2\|TGFB\|TCF21\|GAB2\|PBX1\|TGFBR1\|TGFBR2\|TGFBR3\|SNRK\|KLF6\|CD4\|BCL2L11\|OSTM1\|BCL6\|BCL2\|SOX6 |
| 1568 | 3.16E-09 | 7.86E-07 | 21 | 265 | 237 | 14294 | blood vessel development | SEMA5A\|EDN1\|HLF\|ANXA2\|TIPARP\|CITED2\|LMO2\|LAMA3\|PTEN\|PDGFA\|THBS1\|TGFBR1\|SMAD7\|TGFBR2\|DLL4\|EDNRA\|GJA1\|ZMIZ1\|AKT1\|PNPLA6\|SLIT2 |
| 50789 | 3.69E-09 | 8.34E-07 | 153 | 6548 | 237 | 14294 | regulation of biological process | CD86\|MAML2\|HHIP\|RTKN2\|NIRF\|ETS1\|NDST1\|PREX1\|RPS6KA3\|EDNRA\|GJA1\|EDNRB\|RASSF2\|RASSF3\|ZMIZ1\|DPYSL2\|AKT2\|RPS6KA2\|PDK4\|AKT1\|SOX6\|PKNOX2\|BNIP3L\|MBNL2\|DAPK1\|LIN28\|PRKCE\|TMOD2\|LMO2\|SOX13\|TBC1D2B\|DKK3\|RAB32\|ULK2\|UTRN\|DDX5\|RRAD\|ABHD2\|TGFB\|GATA6\|NEDD4L\|PDGFA\|ADRB1\|ADRB2\|GATA2\|ZBTB4\|CORO1C\|BCL2L11\|RAD21\|TPPP\|PAG1\|MCL1\|LYN\|CBX7\|EGR2\|UBE2B\|STAC\|RDX\|RASSF8\|ARHGAP28\|PBX1\|SMAD7\|DAB2\|ARHGAP30\|NR4A3\|BCL6\|GNAQ\|BCL2\|RGL1\|FERMT2\|ITM2B\|BCL2L2\|PHF2\|CDKN1A\|BTG2\|BTG1\|BMPR2\|CITED2\|PTEN\|TCF21\|TNFAIP1\|AFF3\|PTPRG\|AKAP12\|PPP3CA\|TMSB4X\|TIMP3\|ADAMTS8\|JAK1\|KLF10\|EDN1\|ANXA2\|ARHGEF15\|ANXA4\|SH2D3C\|ETV1\|GAB2\|TNFRSF1B\|SREBF2\|TGFBR1\|TGFBR2\|APBB1IP\|TGFBR3\|KAT2B\|ELF1\|TBC1D2\|LATS2\|CRY2\|RAPGEF2\|NCOA7\|PDE5A\|JDP2\|ARHGEF6\|GAS7\|PTGER4\|HLF\|ZBTB47\|RGS16\|FGL2\|LAMA3\|NTN4\|GLRX\|SAMHD1\|THBS1\|DLL4\|FGD5\|PARD6B\|RASD1\|WIF1\|MKNK2\|TP53INP1\|MFN2\|DMD\|SLIT2\|MAP3K3\|PTPN1\|PCGF5\|SPRY4\|DAB2IP\|HIPK1\|KLF4\|HIPK3\|RAB11A\|PNRC2\|KLF6\|CD4\|TEF\|RAB14\|STK17B\|KLF9\|KRAS\|CHPT1\|CDKN3 |
| 65007 | 4.67E-09 | 9.69E-07 | 159 | 6937 | 237 | 14294 | biological regulation | CD86\|MAML2\|HHIP\|RTKN2\|NIRF\|ETS1\|NDST1\|PREX1\|RPS6KA3\|EDNRA\|GJA1\|EDNRB\|RASSF2\|RASSF3\|ZMIZ1\|DPYSL2\|AKT2\|RPS6KA2\|PDK4\|AKT1\|SOX6\|PKNOX2\|BNIP3L\|MBNL2\|DAPK1\|LIN28\|PRKCE\|TMOD2\|LMO2\|SOX13\|TBC1D2B\|DKK3\|RAB32\|ULK2\|UTRN\|DDX5\|RRAD\|ABHD2\|TGFB\|GATA6\|NEDD4L\|PDGFA\|ADRB1\|ADRB2\|GATA2\|ZBTB4\|CORO1C\|BCL2L11\|RAD21\|TPPP\|PAG1\|MCL1\|LYN\|CBX7\|EGR2\|UBE2B\|STAC\|RDX\|RASSF8\|ARHGAP28\|PBX1\|SMAD7\|DAB2\|ARHGAP30\|NR4A3\|BCL6\|GNAQ\|BCL2\|RGL1\|FERMT2\|ITM2B\|BCL2L2\|PHF2\|CDKN1A\|BTG2\|BTG1\|BMPR2\|CITED2\|PTEN\|SLC40A1\|TCF21\|TNFAIP1\|AFF3\|PTPRG\|SLC6A4\|SYNE1\|AKAP12\|PPP3CA\|TMSB4X\|TIMP3\|ADAMTS8\|JAK1\|KLF10\|EDN1\|ANXA2\|ARHGEF15\|ANXA4\|SH2D3C\|ETV1\|GAB2\|TNFRSF1B\|SREBF2\|TGFBR1\|TGFBR2\|APBB1IP\|TGFBR3\|KAT2B\|ELF1\|TBC1D2\|LATS2\|PLSCR4\|CRY2\|RAPGEF2\|NCOA7\|PDE5A\|JDP2\|ARHGEF6\|GAS7\|PTGER4\|HLF\|ZBTB47\|RGS16\|FGL2\|LAMA3\|NTN4\|GLRX\|SAMHD1\|THBS1\|DLL4\|FGD5\|PARD6B\|RASD1\|WIF1\|MKNK2\|TP53INP1\|MFN2\|DMD\|SLIT2\|LDLR\|LEFTY2\|MAP3K3\|PTPN1\|PCGF5\|SPRY4\|DAB2IP\|HIPK1\|KLF4\|HIPK3\|RAB11A\|PNRC2\|KLF6\|CD4\|TEF\|RAB14\|STK17B\|KLF9\|KRAS\|CHPT1\|CDKN3 |
| 2520 | 6.12E-09 | 1.17E-06 | 21 | 275 | 237 | 14294 | immune system development | LYN\|ITGB1\|HLF\|TIPARP\|RTKN2\|LMO2\|TGFB\|TCF21\|GAB2\|PBX1\|TGFBR1\|TGFBR2\|TGFBR3\|SNRK\|KLF6\|CD4\|BCL2L11\|OSTM1\|BCL6\|BCL2\|SOX6 |
| 23033 | 6.90E-09 | 1.23E-06 | 69 | 2098 | 237 | 14294 | signaling pathway | CD86\|ITGB1\|CDKN1A\|BMPR2\|MAML2\|PTEN\|PTPRG\|AKAP12\|NDST1\|RPS6KA3\|EDNRA\|EDNRB\|AKT2\|RPS6KA2\|RSPO2\|AKT1\|SOSTDC1\|JAK1\|KLF10\|EDN1\|DAPK1\|FBXW11\|PRKCE\|MAGI3\|SH2D3C\|GAB2\|TNFRSF1B\|SREBF2\|TGFBR1\|TGFBR2\|RAB32\|TGFBR3\|LATS2\|DDAH1\|RAPGEF2\|ARHGEF6\|PTGER4\|RRAD\|TGFB\|PDGFA\|ADRB1\|ADRB2\|LTBP3\|THBS1\|NKD1\|DLL4\|RASD1\|WIF1\|MKNK2\|S1PR1\|SLIT2\|LEFTY2\|PAG1\|MAP3K3\|UBE2B\|STAC\|FZD4\|TIPARP\|HIPK1\|RAB11A\|SMAD7\|KLF6\|CD4\|RAB14\|STK17B\|GNAQ\|KLF9\|KRAS\|RGL1 |
| 7275 | 9.03E-09 | 1.40E-06 | 87 | 2968 | 237 | 14294 | multicellular organismal development | SEMA5A\|TRIM71\|ITGB1\|CDKN1A\|BTG2\|BMPR2\|CITED2\|HHIP\|RTKN2\|PTEN\|TCF21\|TNFAIP1\|AFF3\|PTPRG\|SLC6A4\|NDST1\|RPS6KA3\|PPP3CA\|EDNRA\|GJA1\|EDNRB\|ZMIZ1\|DPYSL2\|AKT1\|TIMP3\|SOX6\|KLF10\|EDN1\|ANXA2\|SEMA6D\|TMOD2\|LMO2\|GAB2\|NPNT\|TGFBR1\|DKK3\|TGFBR2\|TGFBR3\|ULK2\|PDE5A\|UTRN\|GAS7\|HLF\|AHNAK\|SDC2\|LAMA3\|TGFB\|GATA6\|NTN4\|PDGFA\|ADRB1\|ADRB2\|LTBP3\|GATA2\|THBS1\|PAPSS2\|DLL4\|PARD6B\|BCL2L11\|WIF1\|MFN2\|DMD\|SLIT2\|LEFTY2\|MCL1\|LYN\|EGR2\|UBE2B\|FZD4\|TIPARP\|SPRY4\|HIPK1\|KLF4\|PBX1\|SMAD7\|DAB2\|SNRK\|KLF6\|CD4\|NR4A3\|OSTM1\|BCL6\|GNAQ\|KLF9\|BCL2\|PNPLA6\|ITM2B |
| 30154 | 9.05E-09 | 1.40E-06 | 59 | 1667 | 237 | 14294 | cell differentiation | SEMA5A\|ITGB1\|BTG2\|CITED2\|HHIP\|PTEN\|TCF21\|NIRF\|PTPRG\|SYNE1\|PPP3CA\|EDNRA\|GJA1\|EDNRB\|ZMIZ1\|DPYSL2\|AKT1\|SOX6\|EDN1\|LIN28\|SEMA6D\|GAB2\|NPNT\|TGFBR1\|TGFBR2\|TGFBR3\|ULK2\|GAS7\|HLF\|SDC2\|LAMA3\|TGFB\|GATA6\|NTN4\|ADRB1\|ADRB2\|GATA2\|DLL4\|PARD6B\|DMD\|SLIT2\|LEFTY2\|MCL1\|LYN\|EGR2\|UBE2B\|FZD4\|TIPARP\|KLF4\|PBX1\|DAB2\|SNRK\|KLF6\|CD4\|OSTM1\|BCL6\|GNAQ\|BCL2\|KRAS |
| 48869 | 9.59E-09 | 1.40E-06 | 60 | 1713 | 237 | 14294 | cellular developmental process | SEMA5A\|ITGB1\|BTG2\|CITED2\|HHIP\|PTEN\|TCF21\|NIRF\|PTPRG\|SYNE1\|PPP3CA\|EDNRA\|GJA1\|EDNRB\|ZMIZ1\|DPYSL2\|AKT1\|SOX6\|EDN1\|LIN28\|SEMA6D\|GAB2\|NPNT\|TGFBR1\|TGFBR2\|TGFBR3\|ULK2\|GAS7\|HLF\|SDC2\|LAMA3\|TGFB\|GATA6\|NTN4\|ADRB1\|ADRB2\|GATA2\|DLL4\|PARD6B\|MFN2\|DMD\|SLIT2\|LEFTY2\|MCL1\|LYN\|EGR2\|UBE2B\|FZD4\|TIPARP\|KLF4\|PBX1\|DAB2\|SNRK\|KLF6\|CD4\|OSTM1\|BCL6\|GNAQ\|BCL2\|KRAS |
| 9719 | 1.22E-08 | 1.69E-06 | 28 | 492 | 237 | 14294 | response to endogenous stimulus | CDKN1A\|BTG2\|BTG1\|TGFB\|GATA6\|PTEN\|PDGFA\|THBS1\|PPP3CA\|GJA1\|DPYSL2\|AKT2\|AKT1\|TIMP3\|SLIT2\|LDLR\|LYN\|EGR2\|UBE2B\|SREBF2\|TGFBR1\|TGFBR3\|KAT2B\|NR4A3\|LATS2\|BCL2\|PDE5A\|KRAS |
| 90066 | 1.43E-08 | 1.87E-06 | 22 | 316 | 237 | 14294 | regulation of anatomical structure size | CDKN1A\|EDN1\|DDX5\|BTG1\|BMPR2\|RRAD\|RDX\|TGFB\|ADRB1\|ADRB2\|TGFBR1\|TGFBR3\|EDNRA\|DAB2\|EDNRB\|BCL6\|TMSB4X\|BCL2\|AKT1\|PDE5A\|SLIT2\|LEFTY2 |
| 9725 | 1.97E-08 | 2.46E-06 | 26 | 440 | 237 | 14294 | response to hormone stimulus | CDKN1A\|BTG2\|BTG1\|TGFB\|GATA6\|PTEN\|PDGFA\|THBS1\|GJA1\|AKT2\|AKT1\|TIMP3\|SLIT2\|LDLR\|LYN\|EGR2\|UBE2B\|SREBF2\|TGFBR1\|TGFBR3\|KAT2B\|NR4A3\|LATS2\|BCL2\|PDE5A\|KRAS |
| 48514 | 2.69E-08 | 3.19E-06 | 18 | 220 | 237 | 14294 | blood vessel morphogenesis | SEMA5A\|EDN1\|HLF\|ANXA2\|TIPARP\|CITED2\|PTEN\|PDGFA\|THBS1\|TGFBR1\|SMAD7\|TGFBR2\|DLL4\|EDNRA\|GJA1\|ZMIZ1\|PNPLA6\|SLIT2 |
| 43687 | 3.91E-08 | 4.42E-06 | 48 | 1266 | 237 | 14294 | post-translational protein modification | CDKN1A\|BTG2\|BTG1\|BMPR2\|USP12\|TGFB\|PTEN\|NEDD4L\|PTPN21\|NIRF\|TNFAIP1\|THBS1\|PTPRG\|RPS6KA3\|PPP3CA\|PPP6C\|RPS6KA2\|MKNK2\|PDK4\|AKT1\|JAK1\|LYN\|ATG3\|MAP3K3\|PTPN1\|UBE2B\|DAPK1\|TIPARP\|FBXW11\|PRKCE\|NEK7\|HIPK1\|HIPK3\|TGFBR1\|SMAD7\|TGFBR2\|TGFBR3\|KAT2B\|RNF144B\|SNRK\|LATS2\|KCTD10\|STK17B\|GNAQ\|CRY2\|BCL2\|ULK2\|SGK1 |
| 6464 | 4.70E-08 | 5.08E-06 | 54 | 1526 | 237 | 14294 | protein modification process | CDKN1A\|BTG2\|BTG1\|BMPR2\|PTEN\|PTPN21\|NIRF\|TNFAIP1\|PTPRG\|RPS6KA3\|PPP3CA\|PPP6C\|AKT2\|RPS6KA2\|PDK4\|AKT1\|JAK1\|ATG3\|DAPK1\|FBXW11\|PRKCE\|TGFBR1\|TGFBR2\|TGFBR3\|KAT2B\|RNF144B\|LATS2\|KCTD10\|CRY2\|ULK2\|SGK1\|ST6GALNAC6\|USP12\|TGFB\|NEDD4L\|THBS1\|MKNK2\|MGAT3\|GCNT4\|LDLR\|LYN\|MAP3K3\|PTPN1\|UBE2B\|TIPARP\|NEK7\|HIPK1\|HIPK3\|SMAD7\|SNRK\|CD4\|STK17B\|GNAQ\|BCL2 |
| 48523 | 5.74E-08 | 5.95E-06 | 61 | 1843 | 237 | 14294 | negative regulation of cellular process | CDKN1A\|BTG2\|BTG1\|BMPR2\|CITED2\|HHIP\|PTEN\|TCF21\|TNFAIP1\|ETS1\|PTPRG\|EDNRA\|GJA1\|AKT2\|TMSB4X\|AKT1\|TIMP3\|SOX6\|ADAMTS8\|KLF10\|BNIP3L\|EDN1\|DAPK1\|LIN28\|ANXA4\|LMO2\|TGFBR1\|DKK3\|TGFBR3\|KAT2B\|ELF1\|LATS2\|JDP2\|GAS7\|RRAD\|ABHD2\|RGS16\|TGFB\|PDGFA\|ADRB2\|THBS1\|DLL4\|RASD1\|TP53INP1\|MFN2\|SLIT2\|PAG1\|MCL1\|PTPN1\|UBE2B\|RDX\|KLF4\|PBX1\|HIPK3\|SMAD7\|DAB2\|BCL6\|BCL2\|KRAS\|BCL2L2\|CDKN3 |
| 30097 | 6.51E-08 | 6.47E-06 | 18 | 233 | 237 | 14294 | hemopoiesis | LYN\|ITGB1\|HLF\|TIPARP\|RTKN2\|LMO2\|TGFB\|GAB2\|PBX1\|TGFBR2\|TGFBR3\|SNRK\|KLF6\|CD4\|OSTM1\|BCL6\|BCL2\|SOX6 |
| 48731 | 8.41E-08 | 8.05E-06 | 73 | 2420 | 237 | 14294 | system development | SEMA5A\|ITGB1\|CDKN1A\|BTG2\|BMPR2\|CITED2\|HHIP\|RTKN2\|PTEN\|TCF21\|PTPRG\|SLC6A4\|NDST1\|RPS6KA3\|PPP3CA\|EDNRA\|GJA1\|EDNRB\|ZMIZ1\|DPYSL2\|AKT1\|TIMP3\|SOX6\|KLF10\|EDN1\|ANXA2\|SEMA6D\|TMOD2\|LMO2\|GAB2\|TGFBR1\|DKK3\|TGFBR2\|TGFBR3\|ULK2\|PDE5A\|UTRN\|GAS7\|HLF\|AHNAK\|SDC2\|LAMA3\|TGFB\|GATA6\|NTN4\|PDGFA\|LTBP3\|GATA2\|THBS1\|PAPSS2\|DLL4\|PARD6B\|BCL2L11\|MFN2\|DMD\|SLIT2\|LYN\|EGR2\|FZD4\|TIPARP\|KLF4\|PBX1\|SMAD7\|SNRK\|KLF6\|CD4\|NR4A3\|OSTM1\|BCL6\|GNAQ\|BCL2\|PNPLA6\|ITM2B |
| 48522 | 9.27E-08 | 8.54E-06 | 64 | 2004 | 237 | 14294 | positive regulation of cellular process | CD86\|CDKN1A\|BTG1\|BMPR2\|MAML2\|CITED2\|RTKN2\|PTEN\|TCF21\|ETS1\|AKAP12\|PREX1\|EDNRA\|GJA1\|ZMIZ1\|DPYSL2\|AKT2\|AKT1\|SOX6\|KLF10\|BNIP3L\|EDN1\|ANXA2\|DAPK1\|LIN28\|PRKCE\|TMOD2\|GAB2\|SREBF2\|TGFBR1\|TGFBR2\|ELF1\|PDE5A\|UTRN\|ARHGEF6\|DDX5\|HLF\|TGFB\|GATA6\|NEDD4L\|PDGFA\|ADRB1\|ADRB2\|GATA2\|THBS1\|BCL2L11\|TP53INP1\|TPPP\|SLIT2\|LYN\|MAP3K3\|EGR2\|UBE2B\|HIPK1\|PBX1\|SMAD7\|KLF6\|CD4\|NR4A3\|BCL6\|STK17B\|BCL2\|KRAS\|ITM2B |
| 50793 | 9.62E-08 | 8.55E-06 | 35 | 790 | 237 | 14294 | regulation of developmental process | PTGER4\|CD86\|BTG1\|BMPR2\|LAMA3\|TGFB\|GATA6\|PTEN\|NTN4\|PDGFA\|ADRB2\|GATA2\|ETS1\|THBS1\|PTPRG\|GJA1\|FGD5\|BCL2L11\|DPYSL2\|AKT1\|SLIT2\|KLF10\|EGR2\|EDN1\|LIN28\|LMO2\|PBX1\|TGFBR1\|SMAD7\|TGFBR2\|BCL6\|GNAQ\|BCL2\|FERMT2\|GAS7 |
| 43412 | 1.05E-07 | 9.03E-06 | 55 | 1607 | 237 | 14294 | macromolecule modification | CDKN1A\|BTG2\|BTG1\|BMPR2\|PTEN\|PTPN21\|ADARB1\|NIRF\|TNFAIP1\|PTPRG\|RPS6KA3\|PPP3CA\|PPP6C\|AKT2\|RPS6KA2\|PDK4\|AKT1\|JAK1\|ATG3\|DAPK1\|FBXW11\|PRKCE\|TGFBR1\|TGFBR2\|TGFBR3\|KAT2B\|RNF144B\|LATS2\|KCTD10\|CRY2\|ULK2\|SGK1\|ST6GALNAC6\|USP12\|TGFB\|NEDD4L\|THBS1\|MKNK2\|MGAT3\|GCNT4\|LDLR\|LYN\|MAP3K3\|PTPN1\|UBE2B\|TIPARP\|NEK7\|HIPK1\|HIPK3\|SMAD7\|SNRK\|CD4\|STK17B\|GNAQ\|BCL2 |
| 7165 | 1.09E-07 | 9.06E-06 | 61 | 1876 | 237 | 14294 | signal transduction | CDKN1A\|RTKN2\|AKAP12\|NDST1\|RPS6KA3\|EDNRA\|EDNRB\|RASSF2\|RASSF3\|DPYSL2\|AKT2\|RPS6KA2\|PDK4\|AKT1\|JAK1\|EDN1\|DAPK1\|PRKCE\|ANXA4\|SH2D3C\|GAB2\|TGFBR1\|APBB1IP\|RAB32\|TGFBR3\|LATS2\|ULK2\|RAPGEF2\|PDE5A\|ARHGEF6\|PTGER4\|HLF\|RRAD\|FGL2\|TGFB\|ADRB1\|ADRB2\|THBS1\|CORO1C\|DLL4\|RASD1\|WIF1\|MKNK2\|SLIT2\|PAG1\|LYN\|MAP3K3\|PTPN1\|STAC\|DAB2IP\|RASSF8\|ARHGAP28\|RAB11A\|KLF6\|CD4\|ARHGAP30\|RAB14\|STK17B\|GNAQ\|KRAS\|RGL1 |
| 48519 | 1.22E-07 | 9.83E-06 | 64 | 2019 | 237 | 14294 | negative regulation of biological process | CDKN1A\|BTG2\|BTG1\|BMPR2\|CITED2\|HHIP\|PTEN\|TCF21\|TNFAIP1\|ETS1\|PTPRG\|EDNRA\|GJA1\|AKT2\|TMSB4X\|AKT1\|TIMP3\|SOX6\|ADAMTS8\|KLF10\|BNIP3L\|EDN1\|ANXA2\|DAPK1\|LIN28\|ANXA4\|LMO2\|TNFRSF1B\|TGFBR1\|DKK3\|TGFBR3\|KAT2B\|ELF1\|LATS2\|JDP2\|GAS7\|RRAD\|ABHD2\|RGS16\|TGFB\|PDGFA\|ADRB1\|ADRB2\|THBS1\|DLL4\|RASD1\|TP53INP1\|MFN2\|SLIT2\|PAG1\|MCL1\|PTPN1\|UBE2B\|RDX\|KLF4\|PBX1\|HIPK3\|SMAD7\|DAB2\|BCL6\|BCL2\|KRAS\|BCL2L2\|CDKN3 |
| 10646 | 1.35E-07 | 1.05E-05 | 44 | 1153 | 237 | 14294 | regulation of cell communication | BMPR2\|CITED2\|HHIP\|RGS16\|TGFB\|PTEN\|ADRB2\|TNFAIP1\|THBS1\|AKAP12\|PREX1\|PPP3CA\|GJA1\|FGD5\|DPYSL2\|AKT2\|AKT1\|MFN2\|SLIT2\|LYN\|MAP3K3\|PTPN1\|EGR2\|EDN1\|UBE2B\|ARHGEF15\|TMOD2\|SPRY4\|DAB2IP\|TBC1D2B\|HIPK3\|RAB11A\|TGFBR1\|DKK3\|SMAD7\|ELF1\|TBC1D2\|CD4\|LATS2\|BCL6\|RAPGEF2\|KRAS\|RGL1\|ARHGEF6 |
| 35466 | 1.72E-07 | 1.30E-05 | 40 | 1002 | 237 | 14294 | regulation of signaling pathway | BMPR2\|CITED2\|HHIP\|RGS16\|TGFB\|PTEN\|ADRB2\|TNFAIP1\|THBS1\|AKAP12\|PREX1\|GJA1\|FGD5\|AKT2\|AKT1\|MFN2\|SLIT2\|LYN\|MAP3K3\|PTPN1\|EDN1\|UBE2B\|ARHGEF15\|TMOD2\|SPRY4\|DAB2IP\|TBC1D2B\|HIPK3\|TGFBR1\|DKK3\|SMAD7\|ELF1\|TBC1D2\|CD4\|LATS2\|BCL6\|RAPGEF2\|KRAS\|RGL1\|ARHGEF6 |
| 35239 | 2.38E-07 | 1.74E-05 | 16 | 201 | 237 | 14294 | tube morphogenesis | SEMA5A\|EDN1\|TGFB\|TCF21\|ADRB1\|ADRB2\|PBX1\|TGFBR2\|EDNRA\|GJA1\|EDNRB\|BCL2L11\|NR4A3\|BCL2\|PDE5A\|SLIT2 |
| 23046 | 2.64E-07 | 1.83E-05 | 66 | 2155 | 237 | 14294 | signaling process | CDKN1A\|RTKN2\|SLC6A4\|AKAP12\|NDST1\|RPS6KA3\|PPP3CA\|EDNRA\|EDNRB\|RASSF2\|RASSF3\|DPYSL2\|AKT2\|RPS6KA2\|PDK4\|AKT1\|JAK1\|EDN1\|DAPK1\|PRKCE\|ANXA4\|TMOD2\|SH2D3C\|GAB2\|TGFBR1\|APBB1IP\|RAB32\|TGFBR3\|LATS2\|ULK2\|RAPGEF2\|PDE5A\|ARHGEF6\|PTGER4\|HLF\|RRAD\|FGL2\|TGFB\|ADRB1\|ADRB2\|THBS1\|CORO1C\|DLL4\|RASD1\|WIF1\|MKNK2\|DMD\|SLIT2\|PAG1\|LYN\|MAP3K3\|PTPN1\|EGR2\|STAC\|DAB2IP\|RASSF8\|ARHGAP28\|RAB11A\|KLF6\|CD4\|ARHGAP30\|RAB14\|STK17B\|GNAQ\|KRAS\|RGL1 |
| 23060 | 2.64E-07 | 1.83E-05 | 66 | 2155 | 237 | 14294 | signal transmission | CDKN1A\|RTKN2\|SLC6A4\|AKAP12\|NDST1\|RPS6KA3\|PPP3CA\|EDNRA\|EDNRB\|RASSF2\|RASSF3\|DPYSL2\|AKT2\|RPS6KA2\|PDK4\|AKT1\|JAK1\|EDN1\|DAPK1\|PRKCE\|ANXA4\|TMOD2\|SH2D3C\|GAB2\|TGFBR1\|APBB1IP\|RAB32\|TGFBR3\|LATS2\|ULK2\|RAPGEF2\|PDE5A\|ARHGEF6\|PTGER4\|HLF\|RRAD\|FGL2\|TGFB\|ADRB1\|ADRB2\|THBS1\|CORO1C\|DLL4\|RASD1\|WIF1\|MKNK2\|DMD\|SLIT2\|PAG1\|LYN\|MAP3K3\|PTPN1\|EGR2\|STAC\|DAB2IP\|RASSF8\|ARHGAP28\|RAB11A\|KLF6\|CD4\|ARHGAP30\|RAB14\|STK17B\|GNAQ\|KRAS\|RGL1 |
| 48513 | 2.82E-07 | 1.85E-05 | 58 | 1790 | 237 | 14294 | organ development | SEMA5A\|ITGB1\|CDKN1A\|BMPR2\|CITED2\|HHIP\|RTKN2\|PTEN\|TCF21\|PTPRG\|SLC6A4\|NDST1\|PPP3CA\|EDNRA\|GJA1\|EDNRB\|ZMIZ1\|DPYSL2\|AKT1\|TIMP3\|SOX6\|KLF10\|EDN1\|ANXA2\|LMO2\|GAB2\|TGFBR1\|DKK3\|TGFBR2\|TGFBR3\|UTRN\|HLF\|LAMA3\|TGFB\|GATA6\|PDGFA\|GATA2\|THBS1\|DLL4\|BCL2L11\|MFN2\|DMD\|SLIT2\|LYN\|EGR2\|TIPARP\|KLF4\|PBX1\|SMAD7\|SNRK\|KLF6\|CD4\|NR4A3\|OSTM1\|BCL6\|GNAQ\|BCL2\|PNPLA6 |
| 51271 | 2.83E-07 | 1.85E-05 | 10 | 72 | 237 | 14294 | negative regulation of cellular component movement | TGFBR3\|DLL4\|CITED2\|ABHD2\|TGFB\|BCL2\|PTEN\|SLIT2\|THBS1\|SMAD7 |
| 48646 | 2.94E-07 | 1.87E-05 | 22 | 375 | 237 | 14294 | anatomical structure formation involved in morphogenesis | SEMA5A\|EGR2\|EDN1\|BMPR2\|HLF\|ANXA2\|UBE2B\|CITED2\|GATA6\|PTEN\|PDGFA\|KLF4\|THBS1\|TGFBR2\|TGFBR3\|DLL4\|EDNRA\|BCL2L11\|NR4A3\|MFN2\|PNPLA6\|SLIT2 |
| 10033 | 3.13E-07 | 1.95E-05 | 36 | 868 | 237 | 14294 | response to organic substance | CD86\|CDKN1A\|BTG2\|BTG1\|SDC2\|TGFB\|GATA6\|PTEN\|PDGFA\|THBS1\|PPP3CA\|EDNRA\|GJA1\|DPYSL2\|AKT2\|AKT1\|TIMP3\|SLIT2\|LDLR\|MCL1\|LYN\|KLF10\|EGR2\|UBE2B\|LIN28\|SREBF2\|TGFBR1\|TGFBR2\|TGFBR3\|KAT2B\|CD4\|NR4A3\|LATS2\|BCL2\|PDE5A\|KRAS |
| 32879 | 4.08E-07 | 2.47E-05 | 32 | 726 | 237 | 14294 | regulation of localization | BMPR2\|CITED2\|ABHD2\|LAMA3\|TGFB\|PTEN\|NEDD4L\|PDGFA\|ADRB2\|GATA2\|ETS1\|THBS1\|DLL4\|EDNRA\|GJA1\|PARD6B\|DPYSL2\|AKT2\|AKT1\|SLIT2\|LYN\|EDN1\|ANXA2\|GAB2\|RAB11A\|TGFBR1\|SMAD7\|TGFBR3\|BCL6\|RAB14\|GNAQ\|BCL2 |
| 42325 | 4.93E-07 | 2.92E-05 | 26 | 518 | 237 | 14294 | regulation of phosphorylation | CDKN1A\|BMPR2\|TGFB\|PTEN\|PDGFA\|ADRB2\|THBS1\|EDNRA\|AKT2\|AKT1\|SLIT2\|LYN\|EDN1\|UBE2B\|PRKCE\|SPRY4\|HIPK3\|TGFBR1\|SMAD7\|TGFBR2\|CD4\|LATS2\|GNAQ\|BCL2\|KRAS\|CDKN3 |
| 30334 | 5.93E-07 | 3.43E-05 | 16 | 215 | 237 | 14294 | regulation of cell migration | EDN1\|BMPR2\|CITED2\|ABHD2\|LAMA3\|TGFB\|PTEN\|PDGFA\|THBS1\|SMAD7\|DLL4\|PARD6B\|AKT2\|BCL2\|AKT1\|SLIT2 |
| 1701 | 6.31E-07 | 3.57E-05 | 16 | 216 | 237 | 14294 | in utero embryonic development | EDN1\|HLF\|UBE2B\|CITED2\|GATA6\|GATA2\|TGFBR1\|EDNRA\|GJA1\|DAB2\|BCL2L11\|ZMIZ1\|AKT1\|MFN2\|SLIT2\|SOX6 |
| 1932 | 6.71E-07 | 3.71E-05 | 16 | 217 | 237 | 14294 | regulation of protein amino acid phosphorylation | LYN\|EDN1\|BMPR2\|UBE2B\|PRKCE\|TGFB\|PTEN\|PDGFA\|HIPK3\|TGFBR1\|SMAD7\|EDNRA\|CD4\|AKT2\|BCL2\|SLIT2 |
| 9790 | 7.81E-07 | 4.22E-05 | 28 | 601 | 237 | 14294 | embryonic development | BMPR2\|HLF\|CITED2\|TGFB\|GATA6\|PDGFA\|TCF21\|TNFAIP1\|GATA2\|NDST1\|EDNRA\|GJA1\|BCL2L11\|ZMIZ1\|AKT1\|MFN2\|SLIT2\|SOX6\|EDN1\|UBE2B\|LMO2\|KLF4\|PBX1\|TGFBR1\|TGFBR2\|DAB2\|NR4A3\|GNAQ |
| 51174 | 1.16E-06 | 6.01E-05 | 26 | 542 | 237 | 14294 | regulation of phosphorus metabolic process | CDKN1A\|BMPR2\|TGFB\|PTEN\|PDGFA\|ADRB2\|THBS1\|EDNRA\|AKT2\|AKT1\|SLIT2\|LYN\|EDN1\|UBE2B\|PRKCE\|SPRY4\|HIPK3\|TGFBR1\|SMAD7\|TGFBR2\|CD4\|LATS2\|GNAQ\|BCL2\|KRAS\|CDKN3 |
| 19220 | 1.16E-06 | 6.01E-05 | 26 | 542 | 237 | 14294 | regulation of phosphate metabolic process | CDKN1A\|BMPR2\|TGFB\|PTEN\|PDGFA\|ADRB2\|THBS1\|EDNRA\|AKT2\|AKT1\|SLIT2\|LYN\|EDN1\|UBE2B\|PRKCE\|SPRY4\|HIPK3\|TGFBR1\|SMAD7\|TGFBR2\|CD4\|LATS2\|GNAQ\|BCL2\|KRAS\|CDKN3 |
| 43065 | 1.27E-06 | 6.45E-05 | 23 | 442 | 237 | 14294 | positive regulation of apoptosis | BNIP3L\|CDKN1A\|DAPK1\|PRKCE\|TGFB\|GATA6\|PTEN\|ADRB2\|HIPK1\|ETS1\|THBS1\|TGFBR1\|PREX1\|BCL2L11\|BCL6\|STK17B\|TP53INP1\|BCL2\|AKT1\|PDE5A\|SLIT2\|ITM2B\|ARHGEF6 |
| 48518 | 1.41E-06 | 6.96E-05 | 65 | 2208 | 237 | 14294 | positive regulation of biological process | CD86\|CDKN1A\|BTG2\|BTG1\|BMPR2\|MAML2\|CITED2\|RTKN2\|PTEN\|TCF21\|ETS1\|AKAP12\|PREX1\|EDNRA\|GJA1\|ZMIZ1\|DPYSL2\|AKT2\|AKT1\|SOX6\|KLF10\|BNIP3L\|EDN1\|ANXA2\|DAPK1\|LIN28\|PRKCE\|TMOD2\|GAB2\|SREBF2\|TGFBR1\|TGFBR2\|ELF1\|PDE5A\|UTRN\|ARHGEF6\|DDX5\|HLF\|TGFB\|GATA6\|NEDD4L\|PDGFA\|ADRB1\|ADRB2\|GATA2\|THBS1\|BCL2L11\|TP53INP1\|TPPP\|SLIT2\|LYN\|MAP3K3\|EGR2\|UBE2B\|HIPK1\|PBX1\|SMAD7\|KLF6\|CD4\|NR4A3\|BCL6\|STK17B\|BCL2\|KRAS\|ITM2B |
| 43068 | 1.43E-06 | 6.96E-05 | 23 | 445 | 237 | 14294 | positive regulation of programmed cell death | BNIP3L\|CDKN1A\|DAPK1\|PRKCE\|TGFB\|GATA6\|PTEN\|ADRB2\|HIPK1\|ETS1\|THBS1\|TGFBR1\|PREX1\|BCL2L11\|BCL6\|STK17B\|TP53INP1\|BCL2\|AKT1\|PDE5A\|SLIT2\|ITM2B\|ARHGEF6 |
| 30336 | 1.51E-06 | 7.21E-05 | 9 | 67 | 237 | 14294 | negative regulation of cell migration | DLL4\|CITED2\|ABHD2\|TGFB\|BCL2\|PTEN\|SLIT2\|THBS1\|SMAD7 |
| 9966 | 1.56E-06 | 7.30E-05 | 32 | 772 | 237 | 14294 | regulation of signal transduction | HHIP\|RGS16\|TGFB\|PTEN\|ADRB2\|TNFAIP1\|THBS1\|AKAP12\|PREX1\|GJA1\|FGD5\|AKT2\|AKT1\|MFN2\|SLIT2\|LYN\|MAP3K3\|EDN1\|UBE2B\|ARHGEF15\|SPRY4\|DAB2IP\|TBC1D2B\|HIPK3\|TGFBR1\|TBC1D2\|CD4\|BCL6\|RAPGEF2\|KRAS\|RGL1\|ARHGEF6 |
| 10942 | 1.66E-06 | 7.66E-05 | 23 | 449 | 237 | 14294 | positive regulation of cell death | BNIP3L\|CDKN1A\|DAPK1\|PRKCE\|TGFB\|GATA6\|PTEN\|ADRB2\|HIPK1\|ETS1\|THBS1\|TGFBR1\|PREX1\|BCL2L11\|BCL6\|STK17B\|TP53INP1\|BCL2\|AKT1\|PDE5A\|SLIT2\|ITM2B\|ARHGEF6 |
| 23051 | 1.79E-06 | 8.08E-05 | 32 | 777 | 237 | 14294 | regulation of signaling process | HHIP\|RGS16\|TGFB\|PTEN\|ADRB2\|TNFAIP1\|THBS1\|AKAP12\|PREX1\|GJA1\|FGD5\|AKT2\|AKT1\|MFN2\|SLIT2\|LYN\|MAP3K3\|EDN1\|UBE2B\|ARHGEF15\|SPRY4\|DAB2IP\|TBC1D2B\|HIPK3\|TGFBR1\|TBC1D2\|CD4\|BCL6\|RAPGEF2\|KRAS\|RGL1\|ARHGEF6 |
| 60389 | 2.13E-06 | 9.46E-05 | 5 | 14 | 237 | 14294 | pathway-restricted SMAD protein phosphorylation | TGFBR3\|TGFB\|TGFBR1\|SMAD7\|TGFBR2 |
| 70482 | 2.27E-06 | 9.77E-05 | 13 | 158 | 237 | 14294 | response to oxygen levels | CDKN1A\|EDN1\|HLF\|UBE2B\|CITED2\|SDC2\|TGFB\|PDGFA\|THBS1\|TGFBR1\|EDNRA\|BCL2\|PDE5A |
| 7166 | 2.28E-06 | 9.77E-05 | 44 | 1278 | 237 | 14294 | cell surface receptor linked signaling pathway | PTGER4\|ITGB1\|BMPR2\|MAML2\|TGFB\|PTEN\|PDGFA\|ADRB1\|ADRB2\|LTBP3\|NKD1\|PTPRG\|AKAP12\|DLL4\|NDST1\|EDNRA\|EDNRB\|RASD1\|WIF1\|AKT2\|MKNK2\|RSPO2\|S1PR1\|AKT1\|SOSTDC1\|SLIT2\|LEFTY2\|JAK1\|KLF10\|EDN1\|UBE2B\|FZD4\|TIPARP\|FBXW11\|HIPK1\|TNFRSF1B\|TGFBR1\|SMAD7\|TGFBR2\|TGFBR3\|KLF6\|CD4\|GNAQ\|KRAS |
| 40012 | 2.54E-06 | 1.07E-04 | 16 | 240 | 237 | 14294 | regulation of locomotion | EDN1\|BMPR2\|CITED2\|ABHD2\|LAMA3\|TGFB\|PTEN\|PDGFA\|THBS1\|SMAD7\|DLL4\|PARD6B\|AKT2\|BCL2\|AKT1\|SLIT2 |
| 40013 | 2.79E-06 | 1.15E-04 | 9 | 72 | 237 | 14294 | negative regulation of locomotion | DLL4\|CITED2\|ABHD2\|TGFB\|BCL2\|PTEN\|SLIT2\|THBS1\|SMAD7 |
| 35556 | 2.81E-06 | 1.15E-04 | 33 | 833 | 237 | 14294 | intracellular signal transduction | PTGER4\|CDKN1A\|RRAD\|ADRB1\|ADRB2\|THBS1\|NDST1\|RPS6KA3\|EDNRA\|EDNRB\|RASD1\|AKT2\|RPS6KA2\|MKNK2\|AKT1\|JAK1\|MAP3K3\|EDN1\|DAPK1\|SH2D3C\|GAB2\|RAB11A\|TGFBR1\|RAB32\|TGFBR3\|LATS2\|RAB14\|STK17B\|GNAQ\|RAPGEF2\|KRAS\|RGL1\|ARHGEF6 |
| 8361 | 3.57E-06 | 1.43E-04 | 15 | 218 | 237 | 14294 | regulation of cell size | CDKN1A\|EDN1\|DDX5\|BTG1\|BMPR2\|RRAD\|TGFB\|TGFBR1\|TGFBR3\|DAB2\|BCL6\|BCL2\|AKT1\|SLIT2\|LEFTY2 |
| 7179 | 3.63E-06 | 1.43E-04 | 8 | 56 | 237 | 14294 | transforming growth factor beta receptor signaling pathway | KLF10\|TGFBR3\|TGFB\|PDGFA\|LTBP3\|LEFTY2\|TGFBR1\|TGFBR2 |
| 30099 | 3.82E-06 | 1.49E-04 | 10 | 95 | 237 | 14294 | myeloid cell differentiation | LYN\|TGFBR3\|SNRK\|HLF\|OSTM1\|BCL6\|TGFB\|GAB2\|SOX6\|TGFBR2 |
| 42127 | 4.02E-06 | 1.54E-04 | 33 | 847 | 237 | 14294 | regulation of cell proliferation | CD86\|CDKN1A\|BTG2\|BTG1\|BMPR2\|RTKN2\|TGFB\|PTEN\|PDGFA\|ADRB2\|ETS1\|THBS1\|EDNRA\|GJA1\|ZMIZ1\|MFN2\|SLIT2\|ADAMTS8\|LYN\|KLF10\|EDN1\|GAB2\|HIPK1\|KLF4\|PBX1\|TGFBR1\|TGFBR2\|TGFBR3\|KAT2B\|BCL6\|BCL2\|KRAS\|CDKN3 |
| 42981 | 4.56E-06 | 1.72E-04 | 33 | 852 | 237 | 14294 | regulation of apoptosis | CDKN1A\|BTG2\|BTG1\|CITED2\|TGFB\|GATA6\|PTEN\|ADRB2\|ETS1\|THBS1\|PREX1\|BCL2L11\|AKT2\|TP53INP1\|AKT1\|SLIT2\|MCL1\|BNIP3L\|UBE2B\|DAPK1\|PRKCE\|ANXA4\|HIPK1\|HIPK3\|TGFBR1\|BCL6\|STK17B\|BCL2\|PDE5A\|KRAS\|ITM2B\|BCL2L2\|ARHGEF6 |
| 32535 | 4.80E-06 | 1.78E-04 | 17 | 282 | 237 | 14294 | regulation of cellular component size | CDKN1A\|EDN1\|DDX5\|BTG1\|BMPR2\|RRAD\|RDX\|TGFB\|TGFBR1\|TGFBR3\|DAB2\|BCL6\|TMSB4X\|BCL2\|AKT1\|SLIT2\|LEFTY2 |
| 7167 | 4.93E-06 | 1.80E-04 | 19 | 345 | 237 | 14294 | enzyme linked receptor protein signaling pathway | KLF10\|BMPR2\|TIPARP\|TGFB\|PTEN\|PDGFA\|ADRB2\|LTBP3\|TGFBR1\|PTPRG\|SMAD7\|TGFBR2\|TGFBR3\|NDST1\|CD4\|AKT2\|AKT1\|LEFTY2\|JAK1 |
| 65008 | 5.17E-06 | 1.86E-04 | 49 | 1541 | 237 | 14294 | regulation of biological quality | CDKN1A\|BTG1\|BMPR2\|PTEN\|SLC40A1\|SLC6A4\|SYNE1\|PPP3CA\|EDNRA\|EDNRB\|TMSB4X\|AKT1\|SOX6\|EDN1\|ANXA2\|TGFBR1\|TGFBR3\|PLSCR4\|PDE5A\|GAS7\|DDX5\|HLF\|RRAD\|TGFB\|NEDD4L\|ADRB1\|GLRX\|ADRB2\|FGD5\|BCL2L11\|DMD\|SLIT2\|LDLR\|LEFTY2\|MCL1\|LYN\|EGR2\|UBE2B\|RDX\|RAB11A\|SMAD7\|DAB2\|CD4\|BCL6\|RAB14\|GNAQ\|BCL2\|KRAS\|FERMT2 |
| 43067 | 5.55E-06 | 1.97E-04 | 33 | 860 | 237 | 14294 | regulation of programmed cell death | CDKN1A\|BTG2\|BTG1\|CITED2\|TGFB\|GATA6\|PTEN\|ADRB2\|ETS1\|THBS1\|PREX1\|BCL2L11\|AKT2\|TP53INP1\|AKT1\|SLIT2\|MCL1\|BNIP3L\|UBE2B\|DAPK1\|PRKCE\|ANXA4\|HIPK1\|HIPK3\|TGFBR1\|BCL6\|STK17B\|BCL2\|PDE5A\|KRAS\|ITM2B\|BCL2L2\|ARHGEF6 |
| 51239 | 5.66E-06 | 1.99E-04 | 38 | 1066 | 237 | 14294 | regulation of multicellular organismal process | PTGER4\|CD86\|BTG1\|BMPR2\|HLF\|LAMA3\|TGFB\|GATA6\|PTEN\|NTN4\|PDGFA\|ADRB1\|ADRB2\|GATA2\|ETS1\|THBS1\|PTPRG\|PPP3CA\|GJA1\|DPYSL2\|AKT1\|SLIT2\|KLF10\|EGR2\|EDN1\|ANXA2\|LMO2\|PBX1\|RAB11A\|TGFBR1\|SMAD7\|TGFBR2\|ELF1\|CD4\|BCL6\|BCL2\|PDE5A\|KRAS |
| 10941 | 6.59E-06 | 2.25E-04 | 33 | 867 | 237 | 14294 | regulation of cell death | CDKN1A\|BTG2\|BTG1\|CITED2\|TGFB\|GATA6\|PTEN\|ADRB2\|ETS1\|THBS1\|PREX1\|BCL2L11\|AKT2\|TP53INP1\|AKT1\|SLIT2\|MCL1\|BNIP3L\|UBE2B\|DAPK1\|PRKCE\|ANXA4\|HIPK1\|HIPK3\|TGFBR1\|BCL6\|STK17B\|BCL2\|PDE5A\|KRAS\|ITM2B\|BCL2L2\|ARHGEF6 |
| 23034 | 6.61E-06 | 2.25E-04 | 40 | 1158 | 237 | 14294 | intracellular signaling pathway | PTGER4\|CDKN1A\|RRAD\|ADRB1\|ADRB2\|THBS1\|NDST1\|RPS6KA3\|EDNRA\|EDNRB\|RASD1\|AKT2\|RPS6KA2\|MKNK2\|AKT1\|PAG1\|JAK1\|MAP3K3\|EDN1\|DAPK1\|STAC\|PRKCE\|MAGI3\|SH2D3C\|GAB2\|HIPK1\|SREBF2\|RAB11A\|TGFBR1\|RAB32\|TGFBR3\|LATS2\|RAB14\|STK17B\|GNAQ\|KLF9\|RAPGEF2\|KRAS\|RGL1\|ARHGEF6 |
| 1666 | 7.00E-06 | 2.35E-04 | 12 | 149 | 237 | 14294 | response to hypoxia | EDNRA\|EDN1\|HLF\|UBE2B\|CITED2\|SDC2\|TGFB\|BCL2\|PDGFA\|PDE5A\|THBS1\|TGFBR1 |
| 45926 | 7.27E-06 | 2.41E-04 | 11 | 125 | 237 | 14294 | negative regulation of growth | CDKN1A\|DAB2\|BTG1\|BMPR2\|BCL6\|RRAD\|TGFB\|BCL2\|ADRB1\|ADRB2\|SLIT2 |
| 1525 | 8.59E-06 | 2.81E-04 | 12 | 152 | 237 | 14294 | angiogenesis | SEMA5A\|DLL4\|EDNRA\|EDN1\|HLF\|ANXA2\|PTEN\|PDGFA\|PNPLA6\|SLIT2\|THBS1\|TGFBR2 |
| 31328 | 8.75E-06 | 2.83E-04 | 29 | 720 | 237 | 14294 | positive regulation of cellular biosynthetic process | CD86\|DDX5\|HLF\|MAML2\|CITED2\|TGFB\|GATA6\|PDGFA\|TCF21\|ADRB1\|ADRB2\|GATA2\|ETS1\|THBS1\|AKAP12\|ZMIZ1\|AKT2\|AKT1\|SOX6\|EGR2\|EDN1\|LIN28\|PBX1\|SREBF2\|TGFBR1\|ELF1\|KLF6\|CD4\|NR4A3 |
| 43009 | 9.07E-06 | 2.89E-04 | 19 | 360 | 237 | 14294 | chordate embryonic development | EDN1\|HLF\|UBE2B\|CITED2\|GATA6\|GATA2\|PBX1\|TGFBR1\|TGFBR2\|NDST1\|EDNRA\|GJA1\|DAB2\|BCL2L11\|ZMIZ1\|AKT1\|MFN2\|SLIT2\|SOX6 |
| 31325 | 9.85E-06 | 3.10E-04 | 35 | 966 | 237 | 14294 | positive regulation of cellular metabolic process | CD86\|DDX5\|BMPR2\|HLF\|MAML2\|CITED2\|TGFB\|GATA6\|PDGFA\|TCF21\|ADRB1\|ADRB2\|GATA2\|ETS1\|THBS1\|AKAP12\|EDNRA\|ZMIZ1\|AKT2\|AKT1\|SOX6\|LYN\|EGR2\|EDN1\|UBE2B\|LIN28\|PBX1\|SREBF2\|TGFBR1\|SMAD7\|ELF1\|KLF6\|CD4\|NR4A3\|BCL2 |
| 9792 | 1.06E-05 | 3.30E-04 | 19 | 364 | 237 | 14294 | embryonic development ending in birth or egg hatching | EDN1\|HLF\|UBE2B\|CITED2\|GATA6\|GATA2\|PBX1\|TGFBR1\|TGFBR2\|NDST1\|EDNRA\|GJA1\|DAB2\|BCL2L11\|ZMIZ1\|AKT1\|MFN2\|SLIT2\|SOX6 |
| 7178 | 1.12E-05 | 3.43E-04 | 10 | 107 | 237 | 14294 | transmembrane receptor protein serine/threonine kinase signaling pathway | KLF10\|TGFBR3\|BMPR2\|TGFB\|PDGFA\|LTBP3\|LEFTY2\|TGFBR1\|SMAD7\|TGFBR2 |
| 9891 | 1.17E-05 | 3.54E-04 | 29 | 731 | 237 | 14294 | positive regulation of biosynthetic process | CD86\|DDX5\|HLF\|MAML2\|CITED2\|TGFB\|GATA6\|PDGFA\|TCF21\|ADRB1\|ADRB2\|GATA2\|ETS1\|THBS1\|AKAP12\|ZMIZ1\|AKT2\|AKT1\|SOX6\|EGR2\|EDN1\|LIN28\|PBX1\|SREBF2\|TGFBR1\|ELF1\|KLF6\|CD4\|NR4A3 |
| 9893 | 1.23E-05 | 3.69E-04 | 36 | 1018 | 237 | 14294 | positive regulation of metabolic process | CD86\|DDX5\|BMPR2\|HLF\|MAML2\|CITED2\|TGFB\|GATA6\|PDGFA\|TCF21\|ADRB1\|ADRB2\|GATA2\|ETS1\|THBS1\|AKAP12\|EDNRA\|GJA1\|ZMIZ1\|AKT2\|AKT1\|SOX6\|LYN\|EGR2\|EDN1\|UBE2B\|LIN28\|PBX1\|SREBF2\|TGFBR1\|SMAD7\|ELF1\|KLF6\|CD4\|NR4A3\|BCL2 |
| 6468 | 1.28E-05 | 3.79E-04 | 27 | 657 | 237 | 14294 | protein amino acid phosphorylation | CDKN1A\|BMPR2\|TGFB\|THBS1\|RPS6KA3\|RPS6KA2\|MKNK2\|PDK4\|AKT1\|JAK1\|LYN\|MAP3K3\|DAPK1\|PRKCE\|NEK7\|HIPK1\|HIPK3\|TGFBR1\|SMAD7\|TGFBR2\|TGFBR3\|SNRK\|LATS2\|STK17B\|BCL2\|ULK2\|SGK1 |
| 45792 | 1.32E-05 | 3.86E-04 | 10 | 109 | 237 | 14294 | negative regulation of cell size | CDKN1A\|DAB2\|BTG1\|BMPR2\|BCL6\|RRAD\|TGFB\|BCL2\|AKT1\|SLIT2 |
| 42221 | 1.46E-05 | 4.23E-04 | 46 | 1464 | 237 | 14294 | response to chemical stimulus | CD86\|CDKN1A\|BTG2\|BTG1\|HLF\|CITED2\|SDC2\|TGFB\|GATA6\|PTEN\|NEDD4L\|PDGFA\|ETS1\|THBS1\|SLC6A4\|PPP3CA\|EDNRA\|GJA1\|EDNRB\|DPYSL2\|AKT2\|AKT1\|TIMP3\|SLIT2\|LDLR\|JAK1\|MCL1\|LYN\|KLF10\|EGR2\|EDN1\|UBE2B\|FZD4\|LIN28\|CYBRD1\|SREBF2\|TGFBR1\|TGFBR2\|TGFBR3\|KAT2B\|CD4\|NR4A3\|LATS2\|BCL2\|PDE5A\|KRAS |
| 6793 | 1.69E-05 | 4.79E-04 | 34 | 949 | 237 | 14294 | phosphorus metabolic process | CDKN1A\|BMPR2\|TGFB\|PTEN\|PTPN21\|THBS1\|PTPRG\|RPS6KA3\|PPP3CA\|PPP6C\|RPS6KA2\|MKNK2\|PDK4\|AKT1\|JAK1\|LYN\|MAP3K3\|PTPN1\|DAPK1\|PRKCE\|NEK7\|HIPK1\|HIPK3\|TGFBR1\|SMAD7\|TGFBR2\|TGFBR3\|SNRK\|LATS2\|STK17B\|BCL2\|ULK2\|GPD1L\|SGK1 |
| 6796 | 1.69E-05 | 4.79E-04 | 34 | 949 | 237 | 14294 | phosphate metabolic process | CDKN1A\|BMPR2\|TGFB\|PTEN\|PTPN21\|THBS1\|PTPRG\|RPS6KA3\|PPP3CA\|PPP6C\|RPS6KA2\|MKNK2\|PDK4\|AKT1\|JAK1\|LYN\|MAP3K3\|PTPN1\|DAPK1\|PRKCE\|NEK7\|HIPK1\|HIPK3\|TGFBR1\|SMAD7\|TGFBR2\|TGFBR3\|SNRK\|LATS2\|STK17B\|BCL2\|ULK2\|GPD1L\|SGK1 |
| 51272 | 1.74E-05 | 4.88E-04 | 11 | 137 | 237 | 14294 | positive regulation of cellular component movement | LYN\|EDN1\|BMPR2\|BCL6\|AKT2\|TGFB\|BCL2\|PDGFA\|ETS1\|THBS1\|TGFBR1 |
| 10748 | 1.78E-05 | 4.89E-04 | 3 | 4 | 237 | 14294 | negative regulation of plasma membrane long-chain fatty acid transport | AKT2\|AKT1\|THBS1 |
| 48754 | 1.79E-05 | 4.89E-04 | 9 | 90 | 237 | 14294 | branching morphogenesis of a tube | SEMA5A\|EDNRA\|EDN1\|TGFB\|BCL2\|TCF21\|SLIT2\|PBX1\|TGFBR2 |
| 9887 | 1.94E-05 | 5.25E-04 | 26 | 634 | 237 | 14294 | organ morphogenesis | SEMA5A\|CDKN1A\|BMPR2\|HHIP\|TGFB\|GATA6\|PDGFA\|TCF21\|NDST1\|EDNRA\|GJA1\|BCL2L11\|ZMIZ1\|MFN2\|SLIT2\|EDN1\|TIPARP\|KLF4\|PBX1\|TGFBR1\|SMAD7\|TGFBR2\|TGFBR3\|NR4A3\|BCL2\|PNPLA6 |
| 3018 | 1.96E-05 | 5.25E-04 | 7 | 51 | 237 | 14294 | vascular process in circulatory system | EDNRA\|EDN1\|EDNRB\|ADRB1\|PDE5A\|ADRB2\|SLIT2 |
| 51173 | 2.18E-05 | 5.76E-04 | 27 | 677 | 237 | 14294 | positive regulation of nitrogen compound metabolic process | CD86\|DDX5\|HLF\|MAML2\|CITED2\|TGFB\|GATA6\|PDGFA\|TCF21\|ADRB1\|ADRB2\|GATA2\|ETS1\|AKAP12\|ZMIZ1\|AKT2\|AKT1\|SOX6\|EGR2\|EDN1\|UBE2B\|PBX1\|SREBF2\|TGFBR1\|ELF1\|KLF6\|NR4A3 |
| 34405 | 2.22E-05 | 5.79E-04 | 4 | 11 | 237 | 14294 | response to fluid shear stress | CITED2\|TGFB\|AKT1\|SMAD7 |
| 82 | 2.23E-05 | 5.79E-04 | 7 | 52 | 237 | 14294 | G1/S transition of mitotic cell cycle | PPP3CA\|PPP6C\|CDKN1A\|LATS2\|BCL2\|AKT1\|CDKN3 |
| 48872 | 2.77E-05 | 7.11E-04 | 9 | 95 | 237 | 14294 | homeostasis of number of cells | LYN\|TGFBR3\|BCL2L11\|HLF\|BCL6\|TGFB\|BCL2\|AKT1\|SOX6 |
| 35467 | 3.07E-05 | 7.80E-04 | 15 | 261 | 237 | 14294 | negative regulation of signaling pathway | PTPN1\|UBE2B\|HHIP\|PTEN\|ADRB2\|TNFAIP1\|THBS1\|DKK3\|SMAD7\|ELF1\|LATS2\|BCL6\|AKT1\|MFN2\|SLIT2 |
| 9968 | 3.29E-05 | 8.27E-04 | 10 | 121 | 237 | 14294 | negative regulation of signal transduction | UBE2B\|BCL6\|HHIP\|RGS16\|PTEN\|AKT1\|MFN2\|TNFAIP1\|SLIT2\|THBS1 |
| 10604 | 3.50E-05 | 8.72E-04 | 33 | 941 | 237 | 14294 | positive regulation of macromolecule metabolic process | CD86\|DDX5\|BMPR2\|HLF\|MAML2\|CITED2\|TGFB\|GATA6\|PDGFA\|TCF21\|ADRB2\|GATA2\|ETS1\|THBS1\|EDNRA\|GJA1\|ZMIZ1\|AKT2\|AKT1\|SOX6\|LYN\|EGR2\|UBE2B\|LIN28\|PBX1\|SREBF2\|TGFBR1\|SMAD7\|ELF1\|KLF6\|CD4\|NR4A3\|BCL2 |
| 23014 | 3.73E-05 | 9.09E-04 | 18 | 364 | 237 | 14294 | signal transmission via phosphorylation event | MAP3K3\|EDN1\|DAPK1\|SH2D3C\|THBS1\|TGFBR1\|TGFBR3\|NDST1\|RPS6KA3\|LATS2\|AKT2\|RPS6KA2\|STK17B\|MKNK2\|AKT1\|RAPGEF2\|JAK1\|ARHGEF6 |
| 7243 | 3.73E-05 | 9.09E-04 | 18 | 364 | 237 | 14294 | intracellular protein kinase cascade | MAP3K3\|EDN1\|DAPK1\|SH2D3C\|THBS1\|TGFBR1\|TGFBR3\|NDST1\|RPS6KA3\|LATS2\|AKT2\|RPS6KA2\|STK17B\|MKNK2\|AKT1\|RAPGEF2\|JAK1\|ARHGEF6 |
| 23057 | 3.79E-05 | 9.15E-04 | 10 | 123 | 237 | 14294 | negative regulation of signaling process | UBE2B\|BCL6\|HHIP\|RGS16\|PTEN\|AKT1\|MFN2\|TNFAIP1\|SLIT2\|THBS1 |
| 2376 | 4.06E-05 | 9.71E-04 | 33 | 948 | 237 | 14294 | immune system process | PTGER4\|CD86\|ITGB1\|HLF\|RTKN2\|TGFB\|TCF21\|TNFAIP1\|SAMHD1\|ETS1\|THBS1\|PREX1\|EDNRB\|BCL2L11\|AKT1\|SOX6\|LYN\|BNIP3L\|EDN1\|TIPARP\|LMO2\|GAB2\|TNFRSF1B\|PBX1\|TGFBR1\|TGFBR2\|TGFBR3\|SNRK\|KLF6\|CD4\|OSTM1\|BCL6\|BCL2 |
| 10765 | 4.39E-05 | 1.03E-03 | 3 | 5 | 237 | 14294 | positive regulation of sodium ion transport | AKT2\|AKT1\|ADRB2 |
| 3085 | 4.39E-05 | 1.03E-03 | 3 | 5 | 237 | 14294 | negative regulation of systemic arterial blood pressure | BMPR2\|ADRB1\|ADRB2 |
| 30308 | 4.88E-05 | 1.14E-03 | 9 | 102 | 237 | 14294 | negative regulation of cell growth | CDKN1A\|DAB2\|BTG1\|BMPR2\|BCL6\|RRAD\|TGFB\|BCL2\|SLIT2 |
| 9888 | 4.94E-05 | 1.14E-03 | 28 | 750 | 237 | 14294 | tissue development | SEMA5A\|BMPR2\|CITED2\|LAMA3\|TGFB\|GATA6\|PTEN\|PDGFA\|TCF21\|PPP3CA\|EDNRA\|GJA1\|EDNRB\|AKT1\|TIMP3\|DMD\|SLIT2\|SOX6\|KLF10\|EDN1\|TIPARP\|KLF4\|PBX1\|SMAD7\|TGFBR2\|TGFBR3\|NR4A3\|BCL2 |
| 48468 | 4.98E-05 | 1.14E-03 | 25 | 631 | 237 | 14294 | cell development | HLF\|SDC2\|NTN4\|GATA2\|PPP3CA\|EDNRA\|GJA1\|PARD6B\|EDNRB\|AKT1\|DMD\|SLIT2\|SOX6\|LEFTY2\|EGR2\|EDN1\|UBE2B\|LIN28\|TGFBR3\|DAB2\|BCL6\|GNAQ\|BCL2\|ULK2\|GAS7 |
| 50880 | 5.39E-05 | 1.21E-03 | 6 | 41 | 237 | 14294 | regulation of blood vessel size | EDNRA\|EDN1\|EDNRB\|ADRB1\|PDE5A\|ADRB2 |
| 35150 | 5.39E-05 | 1.21E-03 | 6 | 41 | 237 | 14294 | regulation of tube size | EDNRA\|EDN1\|EDNRB\|ADRB1\|PDE5A\|ADRB2 |
| 10557 | 5.59E-05 | 1.23E-03 | 26 | 675 | 237 | 14294 | positive regulation of macromolecule biosynthetic process | CD86\|DDX5\|HLF\|MAML2\|CITED2\|TGFB\|GATA6\|PDGFA\|TCF21\|ADRB2\|GATA2\|ETS1\|THBS1\|ZMIZ1\|AKT2\|AKT1\|SOX6\|EGR2\|LIN28\|PBX1\|SREBF2\|TGFBR1\|ELF1\|KLF6\|CD4\|NR4A3 |
| 48729 | 5.60E-05 | 1.23E-03 | 15 | 275 | 237 | 14294 | tissue morphogenesis | SEMA5A\|EDN1\|BMPR2\|TGFB\|TCF21\|KLF4\|PBX1\|SMAD7\|TGFBR2\|TGFBR3\|EDNRA\|GJA1\|NR4A3\|BCL2\|SLIT2 |
| 43066 | 5.68E-05 | 1.24E-03 | 18 | 376 | 237 | 14294 | negative regulation of apoptosis | BNIP3L\|CDKN1A\|BTG2\|UBE2B\|DAPK1\|CITED2\|ANXA4\|PTEN\|THBS1\|HIPK3\|TGFBR1\|BCL6\|AKT2\|BCL2\|AKT1\|KRAS\|BCL2L2\|MCL1 |
| 8285 | 6.08E-05 | 1.32E-03 | 18 | 378 | 237 | 14294 | negative regulation of cell proliferation | KLF10\|CDKN1A\|BTG2\|BTG1\|TGFB\|PTEN\|KLF4\|ETS1\|THBS1\|TGFBR3\|KAT2B\|GJA1\|BCL6\|BCL2\|MFN2\|SLIT2\|ADAMTS8\|CDKN3 |
| 10594 | 6.21E-05 | 1.33E-03 | 6 | 42 | 237 | 14294 | regulation of endothelial cell migration | DLL4\|EDN1\|BMPR2\|TGFB\|SLIT2\|THBS1 |
| 43069 | 6.73E-05 | 1.43E-03 | 18 | 381 | 237 | 14294 | negative regulation of programmed cell death | BNIP3L\|CDKN1A\|BTG2\|UBE2B\|DAPK1\|CITED2\|ANXA4\|PTEN\|THBS1\|HIPK3\|TGFBR1\|BCL6\|AKT2\|BCL2\|AKT1\|KRAS\|BCL2L2\|MCL1 |
| 6917 | 7.40E-05 | 1.56E-03 | 16 | 315 | 237 | 14294 | induction of apoptosis | BNIP3L\|CDKN1A\|DAPK1\|PRKCE\|TGFB\|PTEN\|HIPK1\|ETS1\|THBS1\|TGFBR1\|PREX1\|BCL2L11\|STK17B\|TP53INP1\|ITM2B\|ARHGEF6 |
| 12502 | 7.69E-05 | 1.61E-03 | 16 | 316 | 237 | 14294 | induction of programmed cell death | BNIP3L\|CDKN1A\|DAPK1\|PRKCE\|TGFB\|PTEN\|HIPK1\|ETS1\|THBS1\|TGFBR1\|PREX1\|BCL2L11\|STK17B\|TP53INP1\|ITM2B\|ARHGEF6 |
| 7507 | 8.24E-05 | 1.70E-03 | 13 | 221 | 237 | 14294 | heart development | EDN1\|CITED2\|GATA6\|PTEN\|TGFBR1\|SMAD7\|TGFBR2\|TGFBR3\|EDNRA\|GJA1\|ZMIZ1\|GNAQ\|SOX6 |
| 32101 | 8.28E-05 | 1.70E-03 | 12 | 191 | 237 | 14294 | regulation of response to external stimulus | EDNRA\|EDN1\|ANXA2\|BCL6\|AKT2\|TGFB\|PDGFA\|PDE5A\|ADRB2\|SLIT2\|TNFRSF1B\|THBS1 |
| 45844 | 8.68E-05 | 1.74E-03 | 3 | 6 | 237 | 14294 | positive regulation of striated muscle tissue development | GJA1\|BCL2\|ADRB2 |
| 10746 | 8.68E-05 | 1.74E-03 | 3 | 6 | 237 | 14294 | regulation of plasma membrane long-chain fatty acid transport | AKT2\|AKT1\|THBS1 |
| 48636 | 8.68E-05 | 1.74E-03 | 3 | 6 | 237 | 14294 | positive regulation of muscle organ development | GJA1\|BCL2\|ADRB2 |
| 60548 | 8.78E-05 | 1.75E-03 | 18 | 389 | 237 | 14294 | negative regulation of cell death | BNIP3L\|CDKN1A\|BTG2\|UBE2B\|DAPK1\|CITED2\|ANXA4\|PTEN\|THBS1\|HIPK3\|TGFBR1\|BCL6\|AKT2\|BCL2\|AKT1\|KRAS\|BCL2L2\|MCL1 |
| 45935 | 9.28E-05 | 1.83E-03 | 25 | 656 | 237 | 14294 | positive regulation of nucleobase, nucleoside, nucleotide and nucleic acid metabolic process | CD86\|DDX5\|HLF\|MAML2\|CITED2\|TGFB\|GATA6\|PDGFA\|TCF21\|ADRB1\|ADRB2\|GATA2\|ETS1\|AKAP12\|ZMIZ1\|AKT2\|SOX6\|EGR2\|UBE2B\|PBX1\|SREBF2\|TGFBR1\|ELF1\|KLF6\|NR4A3 |
| 48545 | 9.44E-05 | 1.85E-03 | 13 | 224 | 237 | 14294 | response to steroid hormone stimulus | CDKN1A\|TGFB\|GATA6\|PTEN\|PDGFA\|THBS1\|TGFBR1\|BCL2\|TIMP3\|PDE5A\|KRAS\|SLIT2\|LDLR |
| 10648 | 1.15E-04 | 2.21E-03 | 16 | 327 | 237 | 14294 | negative regulation of cell communication | PTPN1\|UBE2B\|HHIP\|RGS16\|PTEN\|ADRB2\|TNFAIP1\|THBS1\|DKK3\|SMAD7\|ELF1\|LATS2\|BCL6\|AKT1\|MFN2\|SLIT2 |
| 51058 | 1.15E-04 | 2.21E-03 | 4 | 16 | 237 | 14294 | negative regulation of small GTPase mediated signal transduction | BCL6\|MFN2\|TNFAIP1\|SLIT2 |
| 45595 | 1.35E-04 | 2.59E-03 | 22 | 553 | 237 | 14294 | regulation of cell differentiation | CD86\|KLF10\|EDN1\|BTG1\|BMPR2\|LIN28\|LMO2\|TGFB\|GATA6\|PTEN\|ETS1\|PBX1\|TGFBR1\|PTPRG\|SMAD7\|TGFBR2\|BCL6\|DPYSL2\|GNAQ\|BCL2\|AKT1\|SLIT2 |
| 45859 | 1.45E-04 | 2.76E-03 | 17 | 369 | 237 | 14294 | regulation of protein kinase activity | CDKN1A\|EDN1\|SPRY4\|TGFB\|PTEN\|ADRB2\|THBS1\|HIPK3\|TGFBR1\|TGFBR2\|EDNRA\|CD4\|LATS2\|GNAQ\|AKT1\|KRAS\|CDKN3 |
| 10596 | 1.48E-04 | 2.78E-03 | 4 | 17 | 237 | 14294 | negative regulation of endothelial cell migration | DLL4\|TGFB\|SLIT2\|THBS1 |
| 70723 | 1.50E-04 | 2.78E-03 | 3 | 7 | 237 | 14294 | response to cholesterol | TGFB\|TGFBR1\|TGFBR2 |
| 14829 | 1.50E-04 | 2.78E-03 | 3 | 7 | 237 | 14294 | vascular smooth muscle contraction | EDNRA\|EDN1\|EDNRB |
| 7568 | 1.52E-04 | 2.79E-03 | 10 | 145 | 237 | 14294 | aging | CD86\|CDKN1A\|ZMIZ1\|TGFB\|BCL2\|PTEN\|TIMP3\|KRAS\|SREBF2\|TGFBR1 |
| 7399 | 1.67E-04 | 3.05E-03 | 36 | 1154 | 237 | 14294 | nervous system development | SEMA5A\|BTG2\|AHNAK\|CITED2\|HHIP\|SDC2\|TGFB\|PTEN\|NTN4\|GATA2\|PTPRG\|SLC6A4\|NDST1\|RPS6KA3\|GJA1\|PARD6B\|EDNRB\|DPYSL2\|AKT1\|TIMP3\|DMD\|SLIT2\|SOX6\|EGR2\|FZD4\|SEMA6D\|TMOD2\|PBX1\|TGFBR1\|TGFBR2\|GNAQ\|BCL2\|ULK2\|PDE5A\|ITM2B\|GAS7 |
| 45786 | 1.71E-04 | 3.11E-03 | 11 | 176 | 237 | 14294 | negative regulation of cell cycle | KAT2B\|CDKN1A\|LATS2\|BCL6\|TP53INP1\|TGFB\|BCL2\|ETS1\|THBS1\|GAS7\|CDKN3 |
| 51056 | 1.82E-04 | 3.28E-03 | 14 | 272 | 237 | 14294 | regulation of small GTPase mediated signal transduction | ARHGEF15\|DAB2IP\|TBC1D2B\|TNFAIP1\|PREX1\|FGD5\|TBC1D2\|BCL6\|RAPGEF2\|MFN2\|KRAS\|SLIT2\|RGL1\|ARHGEF6 |
| 1763 | 1.84E-04 | 3.29E-03 | 9 | 121 | 237 | 14294 | morphogenesis of a branching structure | SEMA5A\|EDNRA\|EDN1\|TGFB\|BCL2\|TCF21\|SLIT2\|PBX1\|TGFBR2 |
| 43029 | 1.88E-04 | 3.34E-03 | 4 | 18 | 237 | 14294 | T cell homeostasis | BCL2L11\|TGFB\|BCL2\|AKT1 |
| 14070 | 1.90E-04 | 3.34E-03 | 10 | 149 | 237 | 14294 | response to organic cyclic substance | EDNRA\|CDKN1A\|BTG2\|DPYSL2\|SDC2\|TGFB\|BCL2\|PTEN\|TIMP3\|TGFBR1 |
| 1952 | 1.95E-04 | 3.42E-03 | 5 | 33 | 237 | 14294 | regulation of cell-matrix adhesion | BCL6\|BCL2\|PTEN\|UTRN\|THBS1 |
| 1558 | 2.12E-04 | 3.68E-03 | 12 | 211 | 237 | 14294 | regulation of cell growth | CDKN1A\|DAB2\|BTG1\|BMPR2\|BCL6\|RRAD\|TGFB\|BCL2\|AKT1\|CHPT1\|SLIT2\|TGFBR1 |
| 43549 | 2.26E-04 | 3.91E-03 | 17 | 383 | 237 | 14294 | regulation of kinase activity | CDKN1A\|EDN1\|SPRY4\|TGFB\|PTEN\|ADRB2\|THBS1\|HIPK3\|TGFBR1\|TGFBR2\|EDNRA\|CD4\|LATS2\|GNAQ\|AKT1\|KRAS\|CDKN3 |
| 1569 | 2.35E-04 | 4.00E-03 | 4 | 19 | 237 | 14294 | patterning of blood vessels | SEMA5A\|EDNRA\|EDN1\|TGFBR2 |
| 48536 | 2.35E-04 | 4.00E-03 | 4 | 19 | 237 | 14294 | spleen development | BCL2L11\|BCL2\|TCF21\|PBX1 |
| 32891 | 2.37E-04 | 4.01E-03 | 3 | 8 | 237 | 14294 | negative regulation of organic acid transport | AKT2\|AKT1\|THBS1 |
| 48568 | 2.52E-04 | 4.23E-03 | 12 | 215 | 237 | 14294 | embryonic organ development | NDST1\|EDN1\|NR4A3\|HLF\|CITED2\|LMO2\|AKT1\|TCF21\|GATA2\|PBX1\|TGFBR1\|TGFBR2 |
| 61138 | 2.54E-04 | 4.25E-03 | 8 | 100 | 237 | 14294 | morphogenesis of a branching epithelium | SEMA5A\|EDNRA\|EDN1\|TGFB\|BCL2\|TCF21\|PBX1\|TGFBR2 |
| 42326 | 2.59E-04 | 4.28E-03 | 6 | 54 | 237 | 14294 | negative regulation of phosphorylation | CDKN1A\|UBE2B\|TGFB\|PTEN\|SLIT2\|SMAD7 |
| 33993 | 2.60E-04 | 4.28E-03 | 5 | 35 | 237 | 14294 | response to lipid | TGFBR3\|TGFB\|SREBF2\|TGFBR1\|TGFBR2 |
| 45944 | 2.63E-04 | 4.30E-03 | 17 | 388 | 237 | 14294 | positive regulation of transcription from RNA polymerase II promoter | EGR2\|HLF\|MAML2\|CITED2\|TGFB\|GATA6\|ADRB2\|GATA2\|ETS1\|PBX1\|SREBF2\|ELF1\|KLF6\|NR4A3\|ZMIZ1\|AKT2\|SOX6 |
| 2521 | 2.64E-04 | 4.30E-03 | 9 | 127 | 237 | 14294 | leukocyte differentiation | ITGB1\|KLF6\|CD4\|OSTM1\|BCL6\|TGFB\|BCL2\|GAB2\|TGFBR2 |
| 16310 | 2.66E-04 | 4.30E-03 | 27 | 786 | 237 | 14294 | phosphorylation | CDKN1A\|BMPR2\|TGFB\|THBS1\|RPS6KA3\|RPS6KA2\|MKNK2\|PDK4\|AKT1\|JAK1\|LYN\|MAP3K3\|DAPK1\|PRKCE\|NEK7\|HIPK1\|HIPK3\|TGFBR1\|SMAD7\|TGFBR2\|TGFBR3\|SNRK\|LATS2\|STK17B\|BCL2\|ULK2\|SGK1 |
| 2025 | 2.74E-04 | 4.34E-03 | 2 | 2 | 237 | 14294 | vasodilation by norepinephrine-epinephrine involved in regulation of systemic arterial blood pressure | ADRB1\|ADRB2 |
| 10762 | 2.74E-04 | 4.34E-03 | 2 | 2 | 237 | 14294 | regulation of fibroblast migration | TGFB\|THBS1 |
| 10763 | 2.74E-04 | 4.34E-03 | 2 | 2 | 237 | 14294 | positive regulation of fibroblast migration | TGFB\|THBS1 |
| 80134 | 2.88E-04 | 4.50E-03 | 15 | 319 | 237 | 14294 | regulation of response to stress | LYN\|CD86\|EDN1\|ANXA2\|PDGFA\|ADRB2\|TNFRSF1B\|SAMHD1\|THBS1\|HIPK3\|EDNRA\|BCL6\|AKT2\|AKT1\|PDE5A |
| 33135 | 2.90E-04 | 4.50E-03 | 4 | 20 | 237 | 14294 | regulation of peptidyl-serine phosphorylation | AKT2\|TGFB\|BCL2\|SMAD7 |
| 18107 | 2.90E-04 | 4.50E-03 | 4 | 20 | 237 | 14294 | peptidyl-threonine phosphorylation | BCL2\|HIPK3\|TGFBR1\|TGFBR2 |
| 31099 | 3.31E-04 | 5.11E-03 | 7 | 79 | 237 | 14294 | regeneration | GJA1\|CDKN1A\|NR4A3\|TGFB\|BCL2\|TIMP3\|TGFBR1 |
| 42327 | 3.33E-04 | 5.11E-03 | 9 | 131 | 237 | 14294 | positive regulation of phosphorylation | LYN\|EDNRA\|CD4\|BMPR2\|AKT2\|TGFB\|BCL2\|THBS1\|TGFBR1 |
| 90100 | 3.40E-04 | 5.18E-03 | 5 | 37 | 237 | 14294 | positive regulation of transmembrane receptor protein serine/threonine kinase signaling pathway | BMPR2\|CITED2\|TGFB\|THBS1\|TGFBR1 |
| 35162 | 3.51E-04 | 5.30E-03 | 3 | 9 | 237 | 14294 | embryonic hemopoiesis | LMO2\|PBX1\|TGFBR2 |
| 60393 | 3.53E-04 | 5.30E-03 | 4 | 21 | 237 | 14294 | regulation of pathway-restricted SMAD protein phosphorylation | BMPR2\|TGFB\|TGFBR1\|SMAD7 |
| 51338 | 3.54E-04 | 5.30E-03 | 17 | 398 | 237 | 14294 | regulation of transferase activity | CDKN1A\|EDN1\|SPRY4\|TGFB\|PTEN\|ADRB2\|THBS1\|HIPK3\|TGFBR1\|TGFBR2\|EDNRA\|CD4\|LATS2\|GNAQ\|AKT1\|KRAS\|CDKN3 |
| 48699 | 3.59E-04 | 5.35E-03 | 22 | 593 | 237 | 14294 | generation of neurons | EGR2\|BTG2\|FZD4\|HHIP\|SDC2\|TGFB\|PTEN\|NTN4\|GATA2\|PBX1\|TGFBR1\|PTPRG\|GJA1\|PARD6B\|DPYSL2\|GNAQ\|BCL2\|AKT1\|ULK2\|DMD\|SLIT2\|GAS7 |
| 51246 | 3.70E-04 | 5.48E-03 | 23 | 635 | 237 | 14294 | regulation of protein metabolic process | LYN\|EDN1\|BMPR2\|UBE2B\|LIN28\|PRKCE\|TGFB\|PTEN\|NEDD4L\|PDGFA\|THBS1\|HIPK3\|TGFBR1\|SMAD7\|EDNRA\|GJA1\|CD4\|AKT2\|MKNK2\|BCL2\|AKT1\|TIMP3\|SLIT2 |
| 22008 | 3.79E-04 | 5.56E-03 | 23 | 636 | 237 | 14294 | neurogenesis | EGR2\|BTG2\|FZD4\|HHIP\|SDC2\|TGFB\|PTEN\|NTN4\|GATA2\|PBX1\|TGFBR1\|PTPRG\|GJA1\|PARD6B\|DPYSL2\|GNAQ\|BCL2\|AKT1\|ULK2\|DMD\|SLIT2\|SOX6\|GAS7 |
| 48732 | 3.80E-04 | 5.56E-03 | 11 | 193 | 237 | 14294 | gland development | BCL2L11\|CITED2\|TGFB\|BCL2\|PTEN\|TCF21\|SLIT2\|GATA2\|PBX1\|TGFBR1\|DKK3 |
| 45936 | 3.84E-04 | 5.56E-03 | 6 | 58 | 237 | 14294 | negative regulation of phosphate metabolic process | CDKN1A\|UBE2B\|TGFB\|PTEN\|SLIT2\|SMAD7 |
| 10563 | 3.84E-04 | 5.56E-03 | 6 | 58 | 237 | 14294 | negative regulation of phosphorus metabolic process | CDKN1A\|UBE2B\|TGFB\|PTEN\|SLIT2\|SMAD7 |
| 45937 | 3.94E-04 | 5.63E-03 | 9 | 134 | 237 | 14294 | positive regulation of phosphate metabolic process | LYN\|EDNRA\|CD4\|BMPR2\|AKT2\|TGFB\|BCL2\|THBS1\|TGFBR1 |
| 10562 | 3.94E-04 | 5.63E-03 | 9 | 134 | 237 | 14294 | positive regulation of phosphorus metabolic process | LYN\|EDNRA\|CD4\|BMPR2\|AKT2\|TGFB\|BCL2\|THBS1\|TGFBR1 |
| 31399 | 4.09E-04 | 5.81E-03 | 16 | 366 | 237 | 14294 | regulation of protein modification process | LYN\|EDN1\|BMPR2\|UBE2B\|PRKCE\|TGFB\|PTEN\|PDGFA\|HIPK3\|TGFBR1\|SMAD7\|EDNRA\|CD4\|AKT2\|BCL2\|SLIT2 |
| 32268 | 4.16E-04 | 5.86E-03 | 21 | 559 | 237 | 14294 | regulation of cellular protein metabolic process | LYN\|EDN1\|BMPR2\|UBE2B\|LIN28\|PRKCE\|TGFB\|PTEN\|PDGFA\|THBS1\|HIPK3\|TGFBR1\|SMAD7\|EDNRA\|CD4\|AKT2\|MKNK2\|BCL2\|AKT1\|TIMP3\|SLIT2 |
| 8016 | 4.17E-04 | 5.86E-03 | 7 | 82 | 237 | 14294 | regulation of heart contraction | GJA1\|EDN1\|HLF\|ADRB1\|PDE5A\|ADRB2\|SMAD7 |
| 1657 | 4.62E-04 | 6.46E-03 | 6 | 60 | 237 | 14294 | ureteric bud development | TGFB\|BCL2\|TCF21\|SLIT2\|PBX1\|SMAD7 |
| 6916 | 4.92E-04 | 6.80E-03 | 11 | 199 | 237 | 14294 | anti-apoptosis | BNIP3L\|DAPK1\|CITED2\|ANXA4\|BCL2\|AKT1\|THBS1\|HIPK3\|BCL2L2\|TGFBR1\|MCL1 |
| 1933 | 4.92E-04 | 6.80E-03 | 5 | 40 | 237 | 14294 | negative regulation of protein amino acid phosphorylation | UBE2B\|TGFB\|PTEN\|SLIT2\|SMAD7 |
| 43537 | 4.96E-04 | 6.81E-03 | 3 | 10 | 237 | 14294 | negative regulation of blood vessel endothelial cell migration | DLL4\|TGFB\|THBS1 |
| 18210 | 5.09E-04 | 6.92E-03 | 4 | 23 | 237 | 14294 | peptidyl-threonine modification | BCL2\|HIPK3\|TGFBR1\|TGFBR2 |
| 48538 | 5.09E-04 | 6.92E-03 | 4 | 23 | 237 | 14294 | thymus development | BCL2L11\|BCL2\|PBX1\|TGFBR1 |
| 48589 | 5.49E-04 | 7.42E-03 | 8 | 112 | 237 | 14294 | developmental growth | GJA1\|ZMIZ1\|TGFB\|BCL2\|PTEN\|AKT1\|TIMP3\|SLIT2 |
| 30278 | 5.57E-04 | 7.49E-03 | 7 | 86 | 237 | 14294 | regulation of ossification | PTGER4\|EGR2\|BMPR2\|TGFB\|BCL2\|ADRB2\|PBX1 |
| 30036 | 5.62E-04 | 7.52E-03 | 12 | 235 | 237 | 14294 | actin cytoskeleton organization | PREX1\|FGD5\|BCL6\|LIMCH1\|TMSB4X\|BCL2\|KRAS\|TNFAIP1\|CORO2B\|FERMT2\|WASF3\|GAS7 |
| 45941 | 6.01E-04 | 7.98E-03 | 21 | 575 | 237 | 14294 | positive regulation of transcription | CD86\|EGR2\|DDX5\|HLF\|MAML2\|CITED2\|TGFB\|GATA6\|TCF21\|ADRB2\|GATA2\|ETS1\|PBX1\|SREBF2\|TGFBR1\|ELF1\|KLF6\|NR4A3\|ZMIZ1\|AKT2\|SOX6 |
| 7389 | 6.03E-04 | 7.98E-03 | 13 | 271 | 237 | 14294 | pattern specification process | SEMA5A\|EGR2\|BTG2\|EDN1\|BMPR2\|CITED2\|HHIP\|HIPK1\|PBX1\|TGFBR1\|TGFBR2\|EDNRA\|LEFTY2 |
| 43627 | 6.17E-04 | 8.13E-03 | 8 | 114 | 237 | 14294 | response to estrogen stimulus | TGFB\|GATA6\|BCL2\|PTEN\|PDGFA\|TIMP3\|LDLR\|TGFBR1 |
| 51128 | 6.50E-04 | 8.52E-03 | 20 | 538 | 237 | 14294 | regulation of cellular component organization | EDN1\|ANXA2\|UBE2B\|RDX\|TGFB\|PTEN\|NEDD4L\|PDGFA\|GATA2\|THBS1\|TGFBR1\|PTPRG\|SMAD7\|FGD5\|TMSB4X\|AKT1\|TPPP\|SLIT2\|FERMT2\|GAS7 |
| 8284 | 6.56E-04 | 8.55E-03 | 18 | 459 | 237 | 14294 | positive regulation of cell proliferation | LYN\|CD86\|CDKN1A\|EDN1\|BMPR2\|RTKN2\|TGFB\|PDGFA\|GAB2\|ADRB2\|HIPK1\|PBX1\|TGFBR1\|TGFBR2\|BCL6\|ZMIZ1\|BCL2\|KRAS |
| 6357 | 6.62E-04 | 8.56E-03 | 25 | 747 | 237 | 14294 | regulation of transcription from RNA polymerase II promoter | HLF\|MAML2\|CITED2\|TGFB\|GATA6\|ADRB2\|GATA2\|ETS1\|ZMIZ1\|AKT2\|RAD21\|SOX6\|PKNOX2\|KLF10\|EGR2\|PBX1\|SREBF2\|SMAD7\|ELF1\|KLF6\|NR4A3\|BCL6\|TEF\|KLF9\|JDP2 |
| 30041 | 6.73E-04 | 8.56E-03 | 3 | 11 | 237 | 14294 | actin filament polymerization | PREX1\|WASF3\|GAS7 |
| 1953 | 6.73E-04 | 8.56E-03 | 3 | 11 | 237 | 14294 | negative regulation of cell-matrix adhesion | BCL6\|PTEN\|THBS1 |
| 48070 | 6.73E-04 | 8.56E-03 | 3 | 11 | 237 | 14294 | regulation of developmental pigmentation | BCL2L11\|GNAQ\|BCL2 |
| 40007 | 6.84E-04 | 8.56E-03 | 11 | 207 | 237 | 14294 | growth | TGFBR3\|GJA1\|DDX5\|ZMIZ1\|TGFB\|BCL2\|PTEN\|AKT1\|TIMP3\|SLIT2\|LEFTY2 |
| 10959 | 6.85E-04 | 8.56E-03 | 7 | 89 | 237 | 14294 | regulation of metal ion transport | EDNRA\|GJA1\|AKT2\|TGFB\|BCL2\|AKT1\|ADRB2 |
| 32501 | 6.87E-04 | 8.56E-03 | 96 | 4372 | 237 | 14294 | multicellular organismal process | SEMA5A\|TRIM71\|ITGB1\|CDKN1A\|BTG2\|BTG1\|BMPR2\|CITED2\|HHIP\|RTKN2\|PTEN\|TCF21\|TNFAIP1\|AFF3\|PTPRG\|SLC6A4\|NDST1\|RPS6KA3\|PPP3CA\|EDNRA\|GJA1\|EDNRB\|ZMIZ1\|DPYSL2\|AKT1\|TIMP3\|SOX6\|KLF10\|EDN1\|ANXA2\|SEMA6D\|TMOD2\|LMO2\|GAB2\|NPNT\|SREBF2\|TGFBR1\|DKK3\|TGFBR2\|TGFBR3\|PLSCR4\|ULK2\|PDE5A\|UTRN\|GAS7\|HLF\|AHNAK\|SDC2\|RGS16\|LAMA3\|TGFB\|GATA6\|NTN4\|NEDD4L\|PDGFA\|ADRB1\|ADRB2\|LTBP3\|GATA2\|THBS1\|PAPSS2\|DLL4\|PARD6B\|BCL2L11\|WIF1\|MFN2\|DMD\|SLIT2\|LDLR\|LEFTY2\|MCL1\|LYN\|EGR2\|UBE2B\|FZD4\|TIPARP\|SPRY4\|HIPK1\|KLF4\|PBX1\|SMAD7\|DAB2\|SNRK\|KLF6\|CD4\|NR4A3\|OSTM1\|BCL6\|RAB14\|GNAQ\|KLF9\|BCL2\|KRAS\|PNPLA6\|ITM2B\|BCL2L2 |
| 30218 | 6.92E-04 | 8.56E-03 | 5 | 43 | 237 | 14294 | erythrocyte differentiation | LYN\|TGFBR3\|HLF\|BCL6\|SOX6 |
| 2696 | 6.93E-04 | 8.56E-03 | 8 | 116 | 237 | 14294 | positive regulation of leukocyte activation | CD86\|CDKN1A\|CD4\|BCL6\|TGFB\|GAB2\|THBS1\|TGFBR2 |
| 51094 | 6.93E-04 | 8.56E-03 | 15 | 347 | 237 | 14294 | positive regulation of developmental process | CD86\|KLF10\|BTG1\|BMPR2\|TGFB\|GATA6\|ADRB2\|GATA2\|ETS1\|THBS1\|TGFBR2\|GJA1\|BCL2\|AKT1\|SLIT2 |
| 6950 | 6.95E-04 | 8.56E-03 | 47 | 1773 | 237 | 14294 | response to stress | CD86\|ITGB1\|CDKN1A\|BTG2\|BTG1\|BMPR2\|HLF\|CITED2\|SDC2\|ABHD2\|TGFB\|NTN4\|PDGFA\|ADRB1\|ADRB2\|HIGD1A\|SAMHD1\|THBS1\|NDST1\|PPP3CA\|EDNRA\|GJA1\|AKT2\|RAD21\|MKNK2\|TP53INP1\|AKT1\|TIMP3\|LYN\|ATG3\|BNIP3L\|EDN1\|UBE2B\|STAC\|SH2D3C\|HIPK1\|TGFBR1\|SMAD7\|KLF6\|BCL6\|GNAQ\|PLSCR4\|CRY2\|BCL2\|PDE5A\|SGK1\|ARHGEF6 |
| 2260 | 7.09E-04 | 8.64E-03 | 4 | 25 | 237 | 14294 | lymphocyte homeostasis | BCL2L11\|TGFB\|BCL2\|AKT1 |
| 10595 | 7.09E-04 | 8.64E-03 | 4 | 25 | 237 | 14294 | positive regulation of endothelial cell migration | EDN1\|BMPR2\|TGFB\|THBS1 |
| 9791 | 7.12E-04 | 8.64E-03 | 6 | 65 | 237 | 14294 | post-embryonic development | BCL2L11\|TIPARP\|GNAQ\|BCL2\|SOX6\|TGFBR1 |
| 30155 | 7.71E-04 | 9.32E-03 | 9 | 147 | 237 | 14294 | regulation of cell adhesion | BCL6\|CITED2\|LAMA3\|TGFB\|BCL2\|PTEN\|UTRN\|THBS1\|SMAD7 |
| 32989 | 7.79E-04 | 9.37E-03 | 15 | 351 | 237 | 14294 | cellular component morphogenesis | EGR2\|UBE2B\|SDC2\|TGFBR3\|GJA1\|DAB2\|PARD6B\|BCL6\|BCL2\|ULK2\|MFN2\|DMD\|SLIT2\|SOX6\|GAS7 |
| 50765 | 8.12E-04 | 9.67E-03 | 2 | 3 | 237 | 14294 | negative regulation of phagocytosis | TGFB\|PTEN |
| 2024 | 8.12E-04 | 9.67E-03 | 2 | 3 | 237 | 14294 | diet induced thermogenesis | ADRB1\|ADRB2 |
| 51258 | 8.55E-04 | 1.01E-02 | 5 | 45 | 237 | 14294 | protein polymerization | PREX1\|TUBA1A\|TPPP\|WASF3\|GAS7 |
| 1934 | 8.66E-04 | 1.02E-02 | 8 | 120 | 237 | 14294 | positive regulation of protein amino acid phosphorylation | LYN\|EDNRA\|CD4\|BMPR2\|AKT2\|TGFB\|BCL2\|TGFBR1 |
| 42310 | 8.86E-04 | 1.04E-02 | 3 | 12 | 237 | 14294 | vasoconstriction | EDNRA\|EDN1\|EDNRB |
| 50867 | 9.14E-04 | 1.07E-02 | 8 | 121 | 237 | 14294 | positive regulation of cell activation | CD86\|CDKN1A\|CD4\|BCL6\|TGFB\|GAB2\|THBS1\|TGFBR2 |
| 40008 | 9.52E-04 | 1.11E-02 | 15 | 358 | 237 | 14294 | regulation of growth | CDKN1A\|BTG1\|BMPR2\|RRAD\|TGFB\|ADRB1\|ADRB2\|TGFBR1\|DAB2\|BCL2L11\|BCL6\|BCL2\|AKT1\|CHPT1\|SLIT2 |
| 30029 | 9.68E-04 | 1.12E-02 | 12 | 250 | 237 | 14294 | actin filament-based process | PREX1\|FGD5\|BCL6\|LIMCH1\|TMSB4X\|BCL2\|KRAS\|TNFAIP1\|CORO2B\|FERMT2\|WASF3\|GAS7 |
| 44267 | 9.72E-04 | 1.12E-02 | 54 | 2153 | 237 | 14294 | cellular protein metabolic process | CDKN1A\|BTG2\|BTG1\|BMPR2\|PTEN\|PTPN21\|NIRF\|TNFAIP1\|PTPRG\|RPS6KA3\|PPP3CA\|PPP6C\|AKT2\|RPS6KA2\|PDK4\|AKT1\|JAK1\|ATG3\|DAPK1\|FBXW11\|PRKCE\|TGFBR1\|TGFBR2\|TGFBR3\|KAT2B\|RNF144B\|LATS2\|KCTD10\|CRY2\|ULK2\|SGK1\|ST6GALNAC6\|USP12\|TGFB\|NEDD4L\|THBS1\|MKNK2\|MGAT3\|GCNT4\|LDLR\|LYN\|MAP3K3\|PTPN1\|UBE2B\|TIPARP\|NEK7\|HIPK1\|HIPK3\|SMAD7\|SNRK\|CD4\|STK17B\|GNAQ\|BCL2 |
| 7169 | 1.09E-03 | 1.24E-02 | 11 | 219 | 237 | 14294 | transmembrane receptor protein tyrosine kinase signaling pathway | NDST1\|CD4\|BMPR2\|TIPARP\|AKT2\|TGFB\|PTEN\|AKT1\|PDGFA\|ADRB2\|PTPRG |
| 10628 | 1.10E-03 | 1.25E-02 | 21 | 603 | 237 | 14294 | positive regulation of gene expression | CD86\|EGR2\|DDX5\|HLF\|MAML2\|CITED2\|TGFB\|GATA6\|TCF21\|ADRB2\|GATA2\|ETS1\|PBX1\|SREBF2\|TGFBR1\|ELF1\|KLF6\|NR4A3\|ZMIZ1\|AKT2\|SOX6 |
| 48286 | 1.10E-03 | 1.25E-02 | 4 | 28 | 237 | 14294 | lung alveolus development | BMPR2\|GATA6\|PDGFA\|TCF21 |
| 43434 | 1.14E-03 | 1.28E-02 | 10 | 187 | 237 | 14294 | response to peptide hormone stimulus | KAT2B\|GJA1\|EGR2\|BTG2\|BTG1\|NR4A3\|UBE2B\|AKT2\|BCL2\|AKT1 |
| 48583 | 1.19E-03 | 1.34E-02 | 19 | 524 | 237 | 14294 | regulation of response to stimulus | LYN\|CD86\|PTPN1\|EDN1\|ANXA2\|TGFB\|PDGFA\|GAB2\|ADRB2\|TNFRSF1B\|SAMHD1\|THBS1\|HIPK3\|EDNRA\|BCL6\|AKT2\|AKT1\|PDE5A\|SLIT2 |
| 21700 | 1.21E-03 | 1.36E-02 | 7 | 98 | 237 | 14294 | developmental maturation | GJA1\|HLF\|GNAQ\|NTN4\|AKT1\|GATA2\|LEFTY2 |
| 34330 | 1.22E-03 | 1.36E-02 | 6 | 72 | 237 | 14294 | cell junction organization | GJA1\|PARD6B\|LAMA3\|TGFB\|BCL2\|SMAD7 |
| 16567 | 1.23E-03 | 1.37E-02 | 10 | 189 | 237 | 14294 | protein ubiquitination | ATG3\|RNF144B\|UBE2B\|KCTD10\|FBXW11\|BCL2\|AKT1\|NEDD4L\|NIRF\|TNFAIP1 |
| 51726 | 1.26E-03 | 1.39E-02 | 17 | 446 | 237 | 14294 | regulation of cell cycle | CDKN1A\|EDN1\|UBE2B\|CITED2\|TGFB\|PTEN\|NIRF\|ETS1\|THBS1\|KAT2B\|LATS2\|BCL6\|TP53INP1\|BCL2\|AKT1\|GAS7\|CDKN3 |
| 34101 | 1.26E-03 | 1.39E-02 | 5 | 49 | 237 | 14294 | erythrocyte homeostasis | LYN\|TGFBR3\|HLF\|BCL6\|SOX6 |
| 18193 | 1.29E-03 | 1.41E-02 | 9 | 158 | 237 | 14294 | peptidyl-amino acid modification | LYN\|KAT2B\|PDK4\|BCL2\|AKT1\|HIPK3\|TGFBR1\|JAK1\|TGFBR2 |
| 1501 | 1.32E-03 | 1.44E-02 | 14 | 332 | 237 | 14294 | skeletal system development | KLF10\|EDN1\|ANXA2\|TIPARP\|TGFB\|LTBP3\|PBX1\|PAPSS2\|TGFBR1\|TGFBR2\|NDST1\|RPS6KA3\|GNAQ\|SOX6 |
| 50865 | 1.33E-03 | 1.45E-02 | 10 | 191 | 237 | 14294 | regulation of cell activation | CD86\|CDKN1A\|CD4\|BCL6\|TGFB\|PDGFA\|GAB2\|THBS1\|PAG1\|TGFBR2 |
| 16202 | 1.39E-03 | 1.50E-02 | 5 | 50 | 237 | 14294 | regulation of striated muscle tissue development | GJA1\|BTG1\|TGFB\|BCL2\|ADRB2 |
| 33138 | 1.43E-03 | 1.53E-02 | 3 | 14 | 237 | 14294 | positive regulation of peptidyl-serine phosphorylation | AKT2\|TGFB\|BCL2 |
| 2028 | 1.43E-03 | 1.53E-02 | 3 | 14 | 237 | 14294 | regulation of sodium ion transport | AKT2\|AKT1\|ADRB2 |
| 18105 | 1.44E-03 | 1.54E-02 | 4 | 30 | 237 | 14294 | peptidyl-serine phosphorylation | AKT1\|HIPK3\|TGFBR1\|TGFBR2 |
| 16055 | 1.45E-03 | 1.55E-02 | 8 | 130 | 237 | 14294 | Wnt receptor signaling pathway | UBE2B\|FZD4\|WIF1\|FBXW11\|RSPO2\|PTEN\|SOSTDC1\|NKD1 |
| 48634 | 1.52E-03 | 1.61E-02 | 5 | 51 | 237 | 14294 | regulation of muscle organ development | GJA1\|BTG1\|TGFB\|BCL2\|ADRB2 |
| 30324 | 1.53E-03 | 1.61E-02 | 7 | 102 | 237 | 14294 | lung development | BMPR2\|HLF\|HHIP\|GATA6\|PDGFA\|TCF21\|TGFBR1 |
| 51329 | 1.53E-03 | 1.61E-02 | 7 | 102 | 237 | 14294 | interphase of mitotic cell cycle | PPP3CA\|PPP6C\|CDKN1A\|LATS2\|BCL2\|AKT1\|CDKN3 |
| 22603 | 1.57E-03 | 1.63E-02 | 13 | 301 | 237 | 14294 | regulation of anatomical structure morphogenesis | EDN1\|BTG1\|TGFB\|NTN4\|PDGFA\|GATA2\|THBS1\|SMAD7\|FGD5\|BCL2\|SLIT2\|FERMT2\|GAS7 |
| 10523 | 1.61E-03 | 1.63E-02 | 2 | 4 | 237 | 14294 | negative regulation of calcium ion transport into cytosol | TGFB\|BCL2 |
| 14820 | 1.61E-03 | 1.63E-02 | 2 | 4 | 237 | 14294 | tonic smooth muscle contraction | EDNRA\|EDN1 |
| 14824 | 1.61E-03 | 1.63E-02 | 2 | 4 | 237 | 14294 | artery smooth muscle contraction | EDNRA\|EDN1 |
| 14826 | 1.61E-03 | 1.63E-02 | 2 | 4 | 237 | 14294 | vein smooth muscle contraction | EDN1\|EDNRB |
| 10799 | 1.61E-03 | 1.63E-02 | 2 | 4 | 237 | 14294 | regulation of peptidyl-threonine phosphorylation | TGFB\|SMAD7 |
| 31649 | 1.61E-03 | 1.63E-02 | 2 | 4 | 237 | 14294 | heat generation | ADRB1\|ADRB2 |
| 48643 | 1.61E-03 | 1.63E-02 | 2 | 4 | 237 | 14294 | positive regulation of skeletal muscle tissue development | BCL2\|ADRB2 |
| 48469 | 1.62E-03 | 1.64E-02 | 6 | 76 | 237 | 14294 | cell maturation | GJA1\|HLF\|GNAQ\|NTN4\|GATA2\|LEFTY2 |
| 45893 | 1.73E-03 | 1.74E-02 | 18 | 500 | 237 | 14294 | positive regulation of transcription, DNA-dependent | EGR2\|HLF\|MAML2\|CITED2\|TGFB\|GATA6\|TCF21\|ADRB2\|GATA2\|ETS1\|PBX1\|SREBF2\|ELF1\|KLF6\|NR4A3\|ZMIZ1\|AKT2\|SOX6 |
| 35270 | 1.73E-03 | 1.74E-02 | 6 | 77 | 237 | 14294 | endocrine system development | CITED2\|GATA6\|GATA2\|PBX1\|TGFBR1\|DKK3 |
| 46580 | 1.77E-03 | 1.74E-02 | 3 | 15 | 237 | 14294 | negative regulation of Ras protein signal transduction | BCL6\|MFN2\|TNFAIP1 |
| 30325 | 1.77E-03 | 1.74E-02 | 3 | 15 | 237 | 14294 | adrenal gland development | CITED2\|PBX1\|DKK3 |
| 44253 | 1.77E-03 | 1.74E-02 | 3 | 15 | 237 | 14294 | positive regulation of multicellular organismal metabolic process | TGFB\|ADRB1\|ADRB2 |
| 32369 | 1.77E-03 | 1.74E-02 | 3 | 15 | 237 | 14294 | negative regulation of lipid transport | AKT2\|AKT1\|THBS1 |
| 14031 | 1.80E-03 | 1.77E-02 | 5 | 53 | 237 | 14294 | mesenchymal cell development | TGFBR3\|EDNRA\|EDN1\|EDNRB\|BCL2 |
| 30323 | 1.81E-03 | 1.77E-02 | 7 | 105 | 237 | 14294 | respiratory tube development | BMPR2\|HLF\|HHIP\|GATA6\|PDGFA\|TCF21\|TGFBR1 |
| 1776 | 1.84E-03 | 1.79E-02 | 4 | 32 | 237 | 14294 | leukocyte homeostasis | BCL2L11\|TGFB\|BCL2\|AKT1 |
| 51896 | 1.84E-03 | 1.79E-02 | 4 | 32 | 237 | 14294 | regulation of protein kinase B signaling cascade | TGFB\|PTEN\|THBS1\|TGFBR1 |
| 51050 | 1.86E-03 | 1.80E-02 | 12 | 270 | 237 | 14294 | positive regulation of transport | EDNRA\|EDN1\|ANXA2\|DPYSL2\|AKT2\|TGFB\|AKT1\|NEDD4L\|GAB2\|ADRB2\|GATA2\|TGFBR1 |
| 51247 | 1.87E-03 | 1.81E-02 | 13 | 307 | 237 | 14294 | positive regulation of protein metabolic process | LYN\|BMPR2\|LIN28\|TGFB\|THBS1\|TGFBR1\|SMAD7\|EDNRA\|GJA1\|CD4\|AKT2\|BCL2\|AKT1 |
| 51325 | 1.91E-03 | 1.83E-02 | 7 | 106 | 237 | 14294 | interphase | PPP3CA\|PPP6C\|CDKN1A\|LATS2\|BCL2\|AKT1\|CDKN3 |
| 43269 | 1.91E-03 | 1.83E-02 | 7 | 106 | 237 | 14294 | regulation of ion transport | EDNRA\|GJA1\|AKT2\|TGFB\|BCL2\|AKT1\|ADRB2 |
| 34329 | 1.96E-03 | 1.85E-02 | 5 | 54 | 237 | 14294 | cell junction assembly | GJA1\|PARD6B\|LAMA3\|BCL2\|SMAD7 |
| 10810 | 1.96E-03 | 1.85E-02 | 5 | 54 | 237 | 14294 | regulation of cell-substrate adhesion | BCL6\|BCL2\|PTEN\|UTRN\|THBS1 |
| 48565 | 1.96E-03 | 1.85E-02 | 5 | 54 | 237 | 14294 | digestive tract development | EDNRB\|TGFB\|GATA6\|TCF21\|TGFBR1 |
| 51254 | 1.97E-03 | 1.85E-02 | 18 | 506 | 237 | 14294 | positive regulation of RNA metabolic process | EGR2\|HLF\|MAML2\|CITED2\|TGFB\|GATA6\|TCF21\|ADRB2\|GATA2\|ETS1\|PBX1\|SREBF2\|ELF1\|KLF6\|NR4A3\|ZMIZ1\|AKT2\|SOX6 |
| 40017 | 2.03E-03 | 1.90E-02 | 8 | 137 | 237 | 14294 | positive regulation of locomotion | EDN1\|BMPR2\|AKT2\|TGFB\|BCL2\|PDGFA\|SLIT2\|THBS1 |
| 7050 | 2.13E-03 | 1.99E-02 | 7 | 108 | 237 | 14294 | cell cycle arrest | KAT2B\|CDKN1A\|TP53INP1\|TGFB\|THBS1\|GAS7\|CDKN3 |
| 45823 | 2.15E-03 | 1.99E-02 | 3 | 16 | 237 | 14294 | positive regulation of heart contraction | EDN1\|ADRB1\|ADRB2 |
| 10812 | 2.15E-03 | 1.99E-02 | 3 | 16 | 237 | 14294 | negative regulation of cell-substrate adhesion | BCL6\|PTEN\|THBS1 |
| 48844 | 2.15E-03 | 1.99E-02 | 3 | 16 | 237 | 14294 | artery morphogenesis | ZMIZ1\|TGFBR1\|SMAD7 |
| 902 | 2.22E-03 | 2.05E-02 | 13 | 313 | 237 | 14294 | cell morphogenesis | EGR2\|SDC2\|TGFBR3\|GJA1\|DAB2\|PARD6B\|BCL6\|BCL2\|ULK2\|DMD\|SLIT2\|SOX6\|GAS7 |
| 51049 | 2.28E-03 | 2.08E-02 | 18 | 513 | 237 | 14294 | regulation of transport | EDN1\|ANXA2\|TGFB\|PTEN\|NEDD4L\|GAB2\|ADRB2\|GATA2\|THBS1\|RAB11A\|TGFBR1\|EDNRA\|GJA1\|DPYSL2\|AKT2\|GNAQ\|BCL2\|AKT1 |
| 51674 | 2.28E-03 | 2.08E-02 | 13 | 314 | 237 | 14294 | localization of cell | ITGB1\|BTG1\|TGFB\|PTEN\|TNFAIP1\|ETS1\|THBS1\|TGFBR1\|TGFBR3\|GJA1\|EDNRB\|AKT1\|SLIT2 |
| 48870 | 2.28E-03 | 2.08E-02 | 13 | 314 | 237 | 14294 | cell motility | ITGB1\|BTG1\|TGFB\|PTEN\|TNFAIP1\|ETS1\|THBS1\|TGFBR1\|TGFBR3\|GJA1\|EDNRB\|AKT1\|SLIT2 |
| 48762 | 2.31E-03 | 2.09E-02 | 5 | 56 | 237 | 14294 | mesenchymal cell differentiation | TGFBR3\|EDNRA\|EDN1\|EDNRB\|BCL2 |
| 2009 | 2.33E-03 | 2.10E-02 | 10 | 206 | 237 | 14294 | morphogenesis of an epithelium | SEMA5A\|EDNRA\|GJA1\|EDN1\|TGFB\|BCL2\|TCF21\|SLIT2\|PBX1\|TGFBR2 |
| 32446 | 2.33E-03 | 2.10E-02 | 10 | 206 | 237 | 14294 | protein modification by small protein conjugation | ATG3\|RNF144B\|UBE2B\|KCTD10\|FBXW11\|BCL2\|AKT1\|NEDD4L\|NIRF\|TNFAIP1 |
| 1655 | 2.54E-03 | 2.26E-02 | 8 | 142 | 237 | 14294 | urogenital system development | BCL2L11\|TIPARP\|BCL2\|PTEN\|TCF21\|SLIT2\|PBX1\|TGFBR1 |
| 42311 | 2.58E-03 | 2.26E-02 | 3 | 17 | 237 | 14294 | vasodilation | ADRB1\|PDE5A\|ADRB2 |
| 30279 | 2.58E-03 | 2.26E-02 | 3 | 17 | 237 | 14294 | negative regulation of ossification | TGFB\|BCL2\|ADRB2 |
| 10862 | 2.58E-03 | 2.26E-02 | 3 | 17 | 237 | 14294 | positive regulation of pathway-restricted SMAD protein phosphorylation | BMPR2\|TGFB\|TGFBR1 |
| 51926 | 2.58E-03 | 2.26E-02 | 3 | 17 | 237 | 14294 | negative regulation of calcium ion transport | TGFB\|BCL2\|ADRB2 |
| 16477 | 2.59E-03 | 2.26E-02 | 12 | 281 | 237 | 14294 | cell migration | ITGB1\|TGFBR3\|GJA1\|EDNRB\|BTG1\|TGFB\|PTEN\|AKT1\|TNFAIP1\|SLIT2\|THBS1\|TGFBR1 |
| 45634 | 2.65E-03 | 2.26E-02 | 2 | 5 | 237 | 14294 | regulation of melanocyte differentiation | GNAQ\|BCL2 |
| 21604 | 2.65E-03 | 2.26E-02 | 2 | 5 | 237 | 14294 | cranial nerve structural organization | EGR2\|DMD |
| 50932 | 2.65E-03 | 2.26E-02 | 2 | 5 | 237 | 14294 | regulation of pigment cell differentiation | GNAQ\|BCL2 |
| 34616 | 2.65E-03 | 2.26E-02 | 2 | 5 | 237 | 14294 | response to laminar fluid shear stress | TGFB\|SMAD7 |
| 34694 | 2.65E-03 | 2.26E-02 | 2 | 5 | 237 | 14294 | response to prostaglandin stimulus | TGFBR3\|TGFBR1 |
| 34695 | 2.65E-03 | 2.26E-02 | 2 | 5 | 237 | 14294 | response to prostaglandin E stimulus | TGFBR3\|TGFBR1 |
| 1993 | 2.65E-03 | 2.26E-02 | 2 | 5 | 237 | 14294 | regulation of systemic arterial blood pressure by norepinephrine-epinephrine | ADRB1\|ADRB2 |
| 71675 | 2.65E-03 | 2.26E-02 | 2 | 5 | 237 | 14294 | regulation of mononuclear cell migration | SLIT2\|THBS1 |
| 51895 | 2.65E-03 | 2.26E-02 | 2 | 5 | 237 | 14294 | negative regulation of focal adhesion assembly | PTEN\|THBS1 |
| 16322 | 2.65E-03 | 2.26E-02 | 2 | 5 | 237 | 14294 | neuron remodeling | GNAQ\|NTN4 |
| 31647 | 2.69E-03 | 2.29E-02 | 5 | 58 | 237 | 14294 | regulation of protein stability | UBE2B\|GNAQ\|BCL2\|PTEN\|SMAD7 |
| 30817 | 2.75E-03 | 2.32E-02 | 7 | 113 | 237 | 14294 | regulation of cAMP biosynthetic process | AKAP12\|EDNRA\|EDN1\|EDNRB\|GNAQ\|ADRB1\|ADRB2 |
| 60541 | 2.75E-03 | 2.32E-02 | 7 | 113 | 237 | 14294 | respiratory system development | BMPR2\|HLF\|HHIP\|GATA6\|PDGFA\|TCF21\|TGFBR1 |
| 51129 | 2.80E-03 | 2.35E-02 | 9 | 177 | 237 | 14294 | negative regulation of cellular component organization | UBE2B\|TMSB4X\|RDX\|TGFB\|PTEN\|SLIT2\|THBS1\|PTPRG\|SMAD7 |
| 31100 | 2.86E-03 | 2.40E-02 | 4 | 36 | 237 | 14294 | organ regeneration | CDKN1A\|NR4A3\|TGFB\|TGFBR1 |
| 6915 | 2.90E-03 | 2.42E-02 | 19 | 567 | 237 | 14294 | apoptosis | MAGI3\|TNFAIP1\|TNFRSF1B\|THBS1\|HIPK3\|PREX1\|RNF144B\|GJA1\|BCL2L11\|RASSF3\|RAD21\|STK17B\|TP53INP1\|BCL2\|AKT1\|SGK1\|ITM2B\|ARHGEF6\|MCL1 |
| 10627 | 2.91E-03 | 2.42E-02 | 13 | 323 | 237 | 14294 | regulation of intracellular protein kinase cascade | LYN\|MAP3K3\|EDN1\|TGFB\|PTEN\|ADRB2\|THBS1\|HIPK3\|TGFBR1\|AKAP12\|GJA1\|AKT2\|AKT1 |
| 30030 | 2.92E-03 | 2.43E-02 | 14 | 362 | 237 | 14294 | cell projection organization | EGR2\|UBE2B\|SDC2\|RDX\|PDGFA\|GJA1\|FGD5\|PARD6B\|BCL2\|AKT1\|ULK2\|DMD\|SLIT2\|GAS7 |
| 8219 | 2.94E-03 | 2.43E-02 | 22 | 698 | 237 | 14294 | cell death | MAGI3\|TGFB\|TNFAIP1\|TNFRSF1B\|THBS1\|HIPK3\|SYNE1\|PREX1\|RNF144B\|GJA1\|BCL2L11\|RASSF3\|RAD21\|STK17B\|TP53INP1\|BCL2\|AKT1\|PNPLA6\|SGK1\|ITM2B\|ARHGEF6\|MCL1 |
| 2694 | 3.01E-03 | 2.48E-02 | 9 | 179 | 237 | 14294 | regulation of leukocyte activation | CD86\|CDKN1A\|CD4\|BCL6\|TGFB\|GAB2\|THBS1\|PAG1\|TGFBR2 |
| 30814 | 3.03E-03 | 2.49E-02 | 7 | 115 | 237 | 14294 | regulation of cAMP metabolic process | AKAP12\|EDNRA\|EDN1\|EDNRB\|GNAQ\|ADRB1\|ADRB2 |
| 48008 | 3.05E-03 | 2.49E-02 | 3 | 18 | 237 | 14294 | platelet-derived growth factor receptor signaling pathway | TIPARP\|PTEN\|PDGFA |
| 60840 | 3.05E-03 | 2.49E-02 | 3 | 18 | 237 | 14294 | artery development | ZMIZ1\|TGFBR1\|SMAD7 |
| 60284 | 3.08E-03 | 2.50E-02 | 11 | 250 | 237 | 14294 | regulation of cell development | EDN1\|BTG1\|DPYSL2\|TGFB\|BCL2\|PTEN\|AKT1\|SLIT2\|PBX1\|PTPRG\|SMAD7 |
| 9628 | 3.09E-03 | 2.50E-02 | 15 | 404 | 237 | 14294 | response to abiotic stimulus | CDKN1A\|BTG2\|UBE2B\|STAC\|TGFB\|ADRB1\|ADRB2\|THBS1\|TGFBR1\|GJA1\|AKT2\|BCL2\|AKT1\|TIMP3\|KRAS |
| 79 | 3.13E-03 | 2.52E-02 | 5 | 60 | 237 | 14294 | regulation of cyclin-dependent protein kinase activity | CDKN1A\|LATS2\|PTEN\|AKT1\|CDKN3 |
| 16265 | 3.20E-03 | 2.58E-02 | 22 | 703 | 237 | 14294 | death | MAGI3\|TGFB\|TNFAIP1\|TNFRSF1B\|THBS1\|HIPK3\|SYNE1\|PREX1\|RNF144B\|GJA1\|BCL2L11\|RASSF3\|RAD21\|STK17B\|TP53INP1\|BCL2\|AKT1\|PNPLA6\|SGK1\|ITM2B\|ARHGEF6\|MCL1 |
| 32103 | 3.22E-03 | 2.58E-02 | 6 | 87 | 237 | 14294 | positive regulation of response to external stimulus | EDNRA\|AKT2\|TGFB\|PDE5A\|SLIT2\|THBS1 |
| 3013 | 3.25E-03 | 2.59E-02 | 9 | 181 | 237 | 14294 | circulatory system process | DLL4\|EDNRA\|EDN1\|EDNRB\|BMPR2\|ADRB1\|PDE5A\|ADRB2\|SLIT2 |
| 8015 | 3.25E-03 | 2.59E-02 | 9 | 181 | 237 | 14294 | blood circulation | DLL4\|EDNRA\|EDN1\|EDNRB\|BMPR2\|ADRB1\|PDE5A\|ADRB2\|SLIT2 |
| 32270 | 3.35E-03 | 2.66E-02 | 12 | 290 | 237 | 14294 | positive regulation of cellular protein metabolic process | LYN\|EDNRA\|CD4\|BMPR2\|AKT2\|LIN28\|TGFB\|BCL2\|AKT1\|THBS1\|TGFBR1\|SMAD7 |
| 31323 | 3.40E-03 | 2.70E-02 | 81 | 3734 | 237 | 14294 | regulation of cellular metabolic process | CD86\|PHF2\|CDKN1A\|BTG2\|BTG1\|BMPR2\|MAML2\|CITED2\|PTEN\|TCF21\|AFF3\|ETS1\|AKAP12\|EDNRA\|EDNRB\|ZMIZ1\|AKT2\|PDK4\|AKT1\|TIMP3\|SOX6\|PKNOX2\|KLF10\|EDN1\|MBNL2\|LIN28\|PRKCE\|SOX13\|TBC1D2B\|ETV1\|TNFRSF1B\|SREBF2\|TGFBR1\|DKK3\|TGFBR2\|KAT2B\|ELF1\|TBC1D2\|LATS2\|CRY2\|NCOA7\|JDP2\|DDX5\|HLF\|ZBTB47\|TGFB\|GATA6\|PDGFA\|ADRB1\|ADRB2\|GATA2\|ZBTB4\|THBS1\|FGD5\|RASD1\|RAD21\|MKNK2\|DMD\|SLIT2\|LYN\|CBX7\|EGR2\|UBE2B\|PCGF5\|SPRY4\|HIPK1\|KLF4\|PBX1\|HIPK3\|SMAD7\|PNRC2\|KLF6\|CD4\|NR4A3\|BCL6\|TEF\|GNAQ\|KLF9\|BCL2\|KRAS\|CDKN3 |
| 2573 | 3.49E-03 | 2.75E-02 | 4 | 38 | 237 | 14294 | myeloid leukocyte differentiation | OSTM1\|TGFB\|GAB2\|TGFBR2 |
| 3073 | 3.49E-03 | 2.75E-02 | 4 | 38 | 237 | 14294 | regulation of systemic arterial blood pressure | EDN1\|BMPR2\|ADRB1\|ADRB2 |
| 12501 | 3.51E-03 | 2.75E-02 | 19 | 577 | 237 | 14294 | programmed cell death | MAGI3\|TNFAIP1\|TNFRSF1B\|THBS1\|HIPK3\|PREX1\|RNF144B\|GJA1\|BCL2L11\|RASSF3\|RAD21\|STK17B\|TP53INP1\|BCL2\|AKT1\|SGK1\|ITM2B\|ARHGEF6\|MCL1 |
| 45597 | 3.58E-03 | 2.79E-02 | 11 | 255 | 237 | 14294 | positive regulation of cell differentiation | CD86\|KLF10\|BTG1\|BMPR2\|TGFB\|GATA6\|BCL2\|AKT1\|SLIT2\|ETS1\|TGFBR2 |
| 70647 | 3.58E-03 | 2.79E-02 | 11 | 255 | 237 | 14294 | protein modification by small protein conjugation or removal | ATG3\|RNF144B\|UBE2B\|KCTD10\|FBXW11\|USP12\|BCL2\|AKT1\|NEDD4L\|NIRF\|TNFAIP1 |
| 42493 | 3.69E-03 | 2.87E-02 | 11 | 256 | 237 | 14294 | response to drug | CDKN1A\|UBE2B\|DPYSL2\|TGFB\|GATA6\|BCL2\|PTEN\|PDGFA\|THBS1\|SREBF2\|TGFBR2 |
| 3006 | 3.73E-03 | 2.89E-02 | 12 | 294 | 237 | 14294 | reproductive developmental process | BCL2L11\|UBE2B\|CITED2\|TGFB\|GATA6\|BCL2\|PTEN\|AKT1\|TCF21\|SLIT2\|LEFTY2\|PBX1 |
| 19222 | 3.81E-03 | 2.94E-02 | 84 | 3916 | 237 | 14294 | regulation of metabolic process | CD86\|PHF2\|CDKN1A\|BTG2\|BTG1\|BMPR2\|MAML2\|CITED2\|PTEN\|TCF21\|AFF3\|ETS1\|AKAP12\|EDNRA\|GJA1\|EDNRB\|ZMIZ1\|AKT2\|PDK4\|AKT1\|TIMP3\|SOX6\|PKNOX2\|KLF10\|BNIP3L\|EDN1\|MBNL2\|LIN28\|PRKCE\|SOX13\|TBC1D2B\|ETV1\|TNFRSF1B\|SREBF2\|TGFBR1\|DKK3\|TGFBR2\|KAT2B\|ELF1\|TBC1D2\|LATS2\|CRY2\|NCOA7\|JDP2\|DDX5\|HLF\|ZBTB47\|TGFB\|GATA6\|NEDD4L\|PDGFA\|ADRB1\|ADRB2\|GATA2\|ZBTB4\|THBS1\|FGD5\|RASD1\|RAD21\|MKNK2\|DMD\|SLIT2\|LYN\|CBX7\|EGR2\|UBE2B\|PCGF5\|SPRY4\|HIPK1\|KLF4\|PBX1\|HIPK3\|SMAD7\|PNRC2\|KLF6\|CD4\|NR4A3\|BCL6\|TEF\|GNAQ\|KLF9\|BCL2\|KRAS\|CDKN3 |
| 30193 | 3.84E-03 | 2.95E-02 | 4 | 39 | 237 | 14294 | regulation of blood coagulation | EDN1\|ANXA2\|PDGFA\|THBS1 |
| 50821 | 3.84E-03 | 2.95E-02 | 4 | 39 | 237 | 14294 | protein stabilization | UBE2B\|GNAQ\|PTEN\|SMAD7 |
| 55123 | 3.86E-03 | 2.95E-02 | 5 | 63 | 237 | 14294 | digestive system development | EDNRB\|TGFB\|GATA6\|TCF21\|TGFBR1 |
| 45601 | 3.93E-03 | 2.95E-02 | 2 | 6 | 237 | 14294 | regulation of endothelial cell differentiation | BTG1\|TGFBR1 |
| 45628 | 3.93E-03 | 2.95E-02 | 2 | 6 | 237 | 14294 | regulation of T-helper 2 cell differentiation | CD86\|BCL6 |
| 50919 | 3.93E-03 | 2.95E-02 | 2 | 6 | 237 | 14294 | negative chemotaxis | PDGFA\|SLIT2 |
| 34698 | 3.93E-03 | 2.95E-02 | 2 | 6 | 237 | 14294 | response to gonadotropin stimulus | TGFBR3\|GATA6 |
| 51414 | 3.93E-03 | 2.95E-02 | 2 | 6 | 237 | 14294 | response to cortisol stimulus | TGFB\|SLIT2 |
| 51593 | 3.93E-03 | 2.95E-02 | 2 | 6 | 237 | 14294 | response to folic acid | BCL2\|TIMP3 |
| 30802 | 4.02E-03 | 3.00E-02 | 7 | 121 | 237 | 14294 | regulation of cyclic nucleotide biosynthetic process | AKAP12\|EDNRA\|EDN1\|EDNRB\|GNAQ\|ADRB1\|ADRB2 |
| 30808 | 4.02E-03 | 3.00E-02 | 7 | 121 | 237 | 14294 | regulation of nucleotide biosynthetic process | AKAP12\|EDNRA\|EDN1\|EDNRB\|GNAQ\|ADRB1\|ADRB2 |
| 2683 | 4.02E-03 | 3.00E-02 | 6 | 91 | 237 | 14294 | negative regulation of immune system process | ELF1\|BCL6\|TGFB\|SLIT2\|THBS1\|PAG1 |
| 60485 | 4.14E-03 | 3.02E-02 | 5 | 64 | 237 | 14294 | mesenchyme development | TGFBR3\|EDNRA\|EDN1\|EDNRB\|BCL2 |
| 32890 | 4.17E-03 | 3.02E-02 | 3 | 20 | 237 | 14294 | regulation of organic acid transport | AKT2\|AKT1\|THBS1 |
| 33158 | 4.17E-03 | 3.02E-02 | 3 | 20 | 237 | 14294 | regulation of protein import into nucleus, translocation | GNAQ\|TGFB\|TGFBR1 |
| 45884 | 4.17E-03 | 3.02E-02 | 3 | 20 | 237 | 14294 | regulation of survival gene product expression | BNIP3L\|AKT1\|TGFBR1 |
| 50764 | 4.17E-03 | 3.02E-02 | 3 | 20 | 237 | 14294 | regulation of phagocytosis | TGFB\|PTEN\|GATA2 |
| 14910 | 4.17E-03 | 3.02E-02 | 3 | 20 | 237 | 14294 | regulation of smooth muscle cell migration | BCL2\|PDGFA\|SLIT2 |
| 44246 | 4.17E-03 | 3.02E-02 | 3 | 20 | 237 | 14294 | regulation of multicellular organismal metabolic process | TGFB\|ADRB1\|ADRB2 |
| 8154 | 4.17E-03 | 3.02E-02 | 3 | 20 | 237 | 14294 | actin polymerization or depolymerization | PREX1\|WASF3\|GAS7 |
| 10551 | 4.17E-03 | 3.02E-02 | 8 | 154 | 237 | 14294 | regulation of gene-specific transcription from RNA polymerase II promoter | CITED2\|RAD21\|TGFB\|GATA6\|GATA2\|ETS1\|SREBF2\|SMAD7 |
| 18209 | 4.22E-03 | 3.05E-02 | 4 | 40 | 237 | 14294 | peptidyl-serine modification | AKT1\|HIPK3\|TGFBR1\|TGFBR2 |
| 90092 | 4.25E-03 | 3.06E-02 | 6 | 92 | 237 | 14294 | regulation of transmembrane receptor protein serine/threonine kinase signaling pathway | BMPR2\|CITED2\|TGFB\|THBS1\|TGFBR1\|SMAD7 |
| 9636 | 4.42E-03 | 3.18E-02 | 5 | 65 | 237 | 14294 | response to toxin | CDKN1A\|GATA6\|BCL2\|TGFBR1\|SLC6A4 |
| 34504 | 4.48E-03 | 3.21E-02 | 6 | 93 | 237 | 14294 | protein localization in nucleus | PPP3CA\|BCL6\|CRY2\|TGFB\|AKT1\|SYNE1 |
| 50790 | 4.52E-03 | 3.23E-02 | 26 | 907 | 237 | 14294 | regulation of catalytic activity | CDKN1A\|TGFB\|PTEN\|ADRB1\|ADRB2\|THBS1\|EDNRA\|FGD5\|EDNRB\|AKT2\|AKT1\|EDN1\|SPRY4\|TBC1D2B\|HIPK3\|TGFBR1\|SMAD7\|TGFBR2\|TBC1D2\|CD4\|LATS2\|BCL6\|GNAQ\|BCL2\|KRAS\|CDKN3 |
| 30799 | 4.60E-03 | 3.27E-02 | 7 | 124 | 237 | 14294 | regulation of cyclic nucleotide metabolic process | AKAP12\|EDNRA\|EDN1\|EDNRB\|GNAQ\|ADRB1\|ADRB2 |
| 80090 | 4.60E-03 | 3.27E-02 | 77 | 3552 | 237 | 14294 | regulation of primary metabolic process | CD86\|PHF2\|BTG2\|BTG1\|BMPR2\|MAML2\|CITED2\|PTEN\|TCF21\|AFF3\|ETS1\|AKAP12\|EDNRA\|GJA1\|EDNRB\|ZMIZ1\|AKT2\|AKT1\|TIMP3\|SOX6\|PKNOX2\|KLF10\|EDN1\|MBNL2\|LIN28\|PRKCE\|SOX13\|TBC1D2B\|ETV1\|TNFRSF1B\|SREBF2\|TGFBR1\|DKK3\|KAT2B\|ELF1\|TBC1D2\|CRY2\|NCOA7\|JDP2\|DDX5\|HLF\|ZBTB47\|TGFB\|GATA6\|NEDD4L\|PDGFA\|ADRB1\|ADRB2\|GATA2\|ZBTB4\|THBS1\|FGD5\|RASD1\|RAD21\|MKNK2\|DMD\|SLIT2\|LYN\|CBX7\|EGR2\|UBE2B\|PCGF5\|HIPK1\|KLF4\|PBX1\|HIPK3\|SMAD7\|PNRC2\|KLF6\|CD4\|NR4A3\|BCL6\|TEF\|GNAQ\|KLF9\|BCL2\|KRAS |
| 50921 | 4.61E-03 | 3.27E-02 | 4 | 41 | 237 | 14294 | positive regulation of chemotaxis | AKT2\|TGFB\|SLIT2\|THBS1 |
| 61061 | 4.77E-03 | 3.36E-02 | 11 | 265 | 237 | 14294 | muscle structure development | TGFBR3\|PPP3CA\|TIPARP\|GATA6\|PTEN\|DMD\|KRAS\|SOX6\|UTRN\|SMAD7\|SYNE1 |
| 45776 | 4.80E-03 | 3.36E-02 | 3 | 21 | 237 | 14294 | negative regulation of blood pressure | BMPR2\|ADRB1\|ADRB2 |
| 46470 | 4.80E-03 | 3.36E-02 | 3 | 21 | 237 | 14294 | phosphatidylcholine metabolic process | PLA2G15\|CHPT1\|PNPLA6 |
| 30501 | 4.80E-03 | 3.36E-02 | 3 | 21 | 237 | 14294 | positive regulation of bone mineralization | BMPR2\|TGFB\|ADRB2 |
| 2682 | 4.82E-03 | 3.37E-02 | 15 | 424 | 237 | 14294 | regulation of immune system process | CD86\|KLF10\|CDKN1A\|LMO2\|TGFB\|GAB2\|SAMHD1\|ETS1\|THBS1\|TGFBR2\|ELF1\|CD4\|BCL6\|SLIT2\|PAG1 |
| 44087 | 4.86E-03 | 3.39E-02 | 8 | 158 | 237 | 14294 | regulation of cellular component biogenesis | EDN1\|TMSB4X\|RDX\|PTEN\|TPPP\|SLIT2\|THBS1\|TGFBR1 |
| 42592 | 4.88E-03 | 3.39E-02 | 23 | 774 | 237 | 14294 | homeostatic process | LYN\|EGR2\|EDN1\|HLF\|TGFB\|SLC40A1\|NEDD4L\|ADRB1\|GLRX\|ADRB2\|TGFBR3\|PPP3CA\|EDNRA\|EDNRB\|BCL2L11\|BCL6\|GNAQ\|BCL2\|AKT1\|DMD\|SOX6\|LDLR\|MCL1 |
| 30098 | 4.97E-03 | 3.44E-02 | 6 | 95 | 237 | 14294 | lymphocyte differentiation | ITGB1\|KLF6\|CD4\|BCL6\|TGFB\|BCL2 |
| 48585 | 5.02E-03 | 3.47E-02 | 7 | 126 | 237 | 14294 | negative regulation of response to stimulus | PTPN1\|BCL6\|TGFB\|AKT1\|ADRB2\|SLIT2\|TNFRSF1B |
| 32870 | 5.24E-03 | 3.61E-02 | 8 | 160 | 237 | 14294 | cellular response to hormone stimulus | KAT2B\|LATS2\|UBE2B\|AKT2\|TGFB\|GATA6\|AKT1\|SLIT2 |
| 9987 | 5.31E-03 | 3.62E-02 | 174 | 9365 | 237 | 14294 | cellular process | SEMA5A\|CD86\|HHIP\|ADARB1\|NIRF\|ETS1\|SAT1\|NDST1\|PREX1\|RPS6KA3\|EDNRA\|GJA1\|EDNRB\|RASSF2\|RASSF3\|ZMIZ1\|DPYSL2\|AKT2\|RPS6KA2\|PDK4\|AKT1\|SVEP1\|SOX6\|ATG3\|MBNL2\|LIMCH1\|DAPK1\|FBXW11\|LIN28\|PRKCE\|SEMA6D\|MAGI3\|TMOD2\|EPDR1\|NPNT\|SCAMP2\|PLA2G15\|SLC5A9\|KCTD10\|ULK2\|NECAB1\|DDX5\|SDC2\|TGFB\|GATA6\|PLA2G3\|NEDD4L\|PDGFA\|ADRB1\|ADRB2\|LTBP3\|GATA2\|PCDH17\|CORO1C\|SNX2\|BCL2L11\|PDZD2\|RAD21\|TPPP\|MCL1\|LYN\|CBX7\|EGR2\|UBE2B\|STAC\|FZD4\|TIPARP\|RDX\|NEK7\|CYBRD1\|CORO2B\|PBX1\|SMAD7\|DAB2\|BCL6\|GNAQ\|BCL2\|FERMT2\|ITM2B\|ITGB1\|PHF2\|SRPX\|CDKN1A\|BTG2\|BTG1\|BMPR2\|CITED2\|PTEN\|SLC40A1\|TCF21\|PTPN21\|TNFAIP1\|PTPRG\|SLC6A4\|SYNE1\|AKAP12\|PPP3CA\|PPP6C\|ACTR1A\|TUBA1A\|TMSB4X\|EMILIN2\|JAK1\|KLF10\|EDN1\|ANXA2\|SH2D3C\|ETV1\|GAB2\|TNFRSF1B\|TGFBR1\|TGFBR2\|TGFBR3\|KAT2B\|RNF144B\|LATS2\|DDAH1\|PLSCR4\|CRY2\|PDE5A\|SYNC\|SGK1\|RBMS2\|ARHGEF6\|GAS7\|ST6GALNAC6\|HLF\|PON2\|USP12\|LAMA3\|NTN4\|GLRX\|SNX33\|SGMS2\|SNX30\|THBS1\|PAPSS2\|DLL4\|FGD5\|PARD6B\|MKNK2\|TP53INP1\|MGAT3\|S1PR1\|MFN2\|DMD\|GCNT4\|SLIT2\|LDLR\|LEFTY2\|WASF3\|SLC25A25\|MAP3K3\|PTPN1\|HIPK1\|KLF4\|HIPK3\|RAB11A\|PNRC2\|GALC\|SNRK\|KLF6\|CD4\|FRAS1\|OSTM1\|RAB14\|SCD\|STK17B\|TACC1\|KRAS\|CHPT1\|PNPLA6\|GPD1L\|CDKN3 |
| 51051 | 5.43E-03 | 3.62E-02 | 8 | 161 | 237 | 14294 | negative regulation of transport | EDN1\|AKT2\|TGFB\|BCL2\|PTEN\|AKT1\|ADRB2\|THBS1 |
| 31000 | 5.44E-03 | 3.62E-02 | 2 | 7 | 237 | 14294 | response to caffeine | SDC2\|BCL2 |
| 2902 | 5.44E-03 | 3.62E-02 | 2 | 7 | 237 | 14294 | regulation of B cell apoptosis | BCL6\|PTEN |
| 40015 | 5.44E-03 | 3.62E-02 | 2 | 7 | 237 | 14294 | negative regulation of multicellular organism growth | ADRB1\|ADRB2 |
| 30335 | 5.47E-03 | 3.62E-02 | 7 | 128 | 237 | 14294 | positive regulation of cell migration | EDN1\|BMPR2\|AKT2\|TGFB\|BCL2\|PDGFA\|THBS1 |
| 50818 | 5.48E-03 | 3.62E-02 | 4 | 43 | 237 | 14294 | regulation of coagulation | EDN1\|ANXA2\|PDGFA\|THBS1 |
| 60021 | 5.48E-03 | 3.62E-02 | 4 | 43 | 237 | 14294 | palate development | TGFBR3\|TIPARP\|TGFBR1\|TGFBR2 |
| 61041 | 5.48E-03 | 3.62E-02 | 4 | 43 | 237 | 14294 | regulation of wound healing | EDN1\|ANXA2\|PDGFA\|THBS1 |
| 90068 | 5.48E-03 | 3.62E-02 | 4 | 43 | 237 | 14294 | positive regulation of cell cycle process | CDKN1A\|EDN1\|UBE2B\|TGFB |
| 8633 | 5.49E-03 | 3.62E-02 | 3 | 22 | 237 | 14294 | activation of pro-apoptotic gene products | BCL2L11\|BCL2\|AKT1 |
| 45930 | 5.49E-03 | 3.62E-02 | 3 | 22 | 237 | 14294 | negative regulation of mitotic cell cycle | BCL6\|TGFB\|BCL2 |
| 2027 | 5.49E-03 | 3.62E-02 | 3 | 22 | 237 | 14294 | regulation of heart rate | EDN1\|HLF\|ADRB1 |
| 51385 | 5.49E-03 | 3.62E-02 | 3 | 22 | 237 | 14294 | response to mineralocorticoid stimulus | CDKN1A\|BCL2\|KRAS |
| 51607 | 5.49E-03 | 3.62E-02 | 3 | 22 | 237 | 14294 | defense response to virus | BNIP3L\|BCL2\|SAMHD1 |
| 43535 | 5.49E-03 | 3.62E-02 | 3 | 22 | 237 | 14294 | regulation of blood vessel endothelial cell migration | DLL4\|TGFB\|THBS1 |
| 45321 | 5.57E-03 | 3.67E-02 | 10 | 233 | 237 | 14294 | leukocyte activation | CD86\|ITGB1\|PREX1\|EDN1\|KLF6\|CD4\|BCL6\|TGFB\|BCL2\|TGFBR2 |
| 46578 | 5.74E-03 | 3.77E-02 | 10 | 234 | 237 | 14294 | regulation of Ras protein signal transduction | PREX1\|FGD5\|TBC1D2\|BCL6\|ARHGEF15\|MFN2\|TBC1D2B\|KRAS\|TNFAIP1\|ARHGEF6 |
| 9266 | 5.78E-03 | 3.78E-02 | 6 | 98 | 237 | 14294 | response to temperature stimulus | STAC\|BCL2\|AKT1\|ADRB1\|ADRB2\|THBS1 |
| 48812 | 5.83E-03 | 3.81E-02 | 9 | 198 | 237 | 14294 | neuron projection morphogenesis | GJA1\|EGR2\|PARD6B\|SDC2\|BCL2\|ULK2\|DMD\|SLIT2\|GAS7 |
| 45216 | 5.95E-03 | 3.86E-02 | 4 | 44 | 237 | 14294 | cell-cell junction organization | GJA1\|PARD6B\|TGFB\|SMAD7 |
| 30183 | 5.95E-03 | 3.86E-02 | 4 | 44 | 237 | 14294 | B cell differentiation | ITGB1\|KLF6\|BCL6\|BCL2 |
| 45860 | 6.08E-03 | 3.94E-02 | 10 | 236 | 237 | 14294 | positive regulation of protein kinase activity | EDNRA\|EDN1\|CD4\|TGFB\|AKT1\|KRAS\|ADRB2\|THBS1\|TGFBR1\|TGFBR2 |
| 44057 | 6.11E-03 | 3.95E-02 | 12 | 313 | 237 | 14294 | regulation of system process | PPP3CA\|GJA1\|EGR2\|EDN1\|BMPR2\|HLF\|ADRB1\|PDE5A\|KRAS\|ADRB2\|RAB11A\|SMAD7 |
| 60537 | 6.19E-03 | 3.98E-02 | 7 | 131 | 237 | 14294 | muscle tissue development | TGFBR3\|PPP3CA\|TIPARP\|GATA6\|PTEN\|DMD\|SMAD7 |
| 70169 | 6.24E-03 | 3.98E-02 | 3 | 23 | 237 | 14294 | positive regulation of biomineral formation | BMPR2\|TGFB\|ADRB2 |
| 46637 | 6.24E-03 | 3.98E-02 | 3 | 23 | 237 | 14294 | regulation of alpha-beta T cell differentiation | CD86\|BCL6\|TGFBR2 |
| 51897 | 6.24E-03 | 3.98E-02 | 3 | 23 | 237 | 14294 | positive regulation of protein kinase B signaling cascade | TGFB\|THBS1\|TGFBR1 |
| 48701 | 6.24E-03 | 3.98E-02 | 3 | 23 | 237 | 14294 | embryonic cranial skeleton morphogenesis | NDST1\|TGFBR1\|TGFBR2 |
| 1775 | 6.27E-03 | 3.99E-02 | 11 | 275 | 237 | 14294 | cell activation | CD86\|ITGB1\|PREX1\|EDN1\|KLF6\|CD4\|BCL6\|TGFB\|BCL2\|PDGFA\|TGFBR2 |
| 1822 | 6.37E-03 | 4.03E-02 | 6 | 100 | 237 | 14294 | kidney development | BCL2L11\|TIPARP\|BCL2\|TCF21\|SLIT2\|TGFBR1 |
| 6469 | 6.37E-03 | 4.03E-02 | 6 | 100 | 237 | 14294 | negative regulation of protein kinase activity | CDKN1A\|LATS2\|GNAQ\|SPRY4\|AKT1\|HIPK3 |
| 6605 | 6.41E-03 | 4.04E-02 | 9 | 201 | 237 | 14294 | protein targeting | AKAP12\|ATG3\|PPP3CA\|BCL6\|CRY2\|TGFB\|AKT1\|MFN2\|LTBP3 |
| 9967 | 6.43E-03 | 4.04E-02 | 11 | 276 | 237 | 14294 | positive regulation of signal transduction | LYN\|AKAP12\|MAP3K3\|GJA1\|CD4\|AKT2\|TGFB\|KRAS\|ADRB2\|THBS1\|TGFBR1 |
| 1570 | 6.45E-03 | 4.04E-02 | 4 | 45 | 237 | 14294 | vasculogenesis | ZMIZ1\|TIPARP\|CITED2\|TGFBR2 |
| 6470 | 6.45E-03 | 4.04E-02 | 7 | 132 | 237 | 14294 | protein amino acid dephosphorylation | PPP3CA\|PPP6C\|PTPN1\|BCL2\|PTEN\|PTPN21\|PTPRG |
| 10552 | 6.68E-03 | 4.16E-02 | 6 | 101 | 237 | 14294 | positive regulation of gene-specific transcription from RNA polymerase II promoter | CITED2\|TGFB\|GATA6\|GATA2\|ETS1\|SREBF2 |
| 43583 | 6.68E-03 | 4.16E-02 | 6 | 101 | 237 | 14294 | ear development | EDN1\|BCL2L11\|NR4A3\|TGFB\|BCL2\|PDGFA |
| 30182 | 6.86E-03 | 4.27E-02 | 15 | 441 | 237 | 14294 | neuron differentiation | EGR2\|BTG2\|FZD4\|SDC2\|NTN4\|GATA2\|TGFBR1\|GJA1\|PARD6B\|GNAQ\|BCL2\|ULK2\|DMD\|SLIT2\|GAS7 |
| 7200 | 6.97E-03 | 4.32E-02 | 4 | 46 | 237 | 14294 | activation of phospholipase C activity by G-protein coupled receptor protein signaling pathway coupled to IP3 second messenger | EDNRA\|EDN1\|EDNRB\|GNAQ |
| 71495 | 6.98E-03 | 4.32E-02 | 8 | 168 | 237 | 14294 | cellular response to endogenous stimulus | KAT2B\|LATS2\|UBE2B\|AKT2\|TGFB\|GATA6\|AKT1\|SLIT2 |
| 7417 | 7.00E-03 | 4.32E-02 | 15 | 442 | 237 | 14294 | central nervous system development | EGR2\|CITED2\|PTEN\|GATA2\|PTPRG\|SLC6A4\|TGFBR2\|NDST1\|RPS6KA3\|DPYSL2\|GNAQ\|BCL2\|TIMP3\|SLIT2\|SOX6 |
| 10608 | 7.02E-03 | 4.32E-02 | 10 | 241 | 237 | 14294 | posttranscriptional regulation of gene expression | UBE2B\|LIN28\|GNAQ\|MKNK2\|BCL2\|PTEN\|AKT1\|TNFRSF1B\|THBS1\|SMAD7 |
| 30195 | 7.04E-03 | 4.33E-02 | 3 | 24 | 237 | 14294 | negative regulation of blood coagulation | EDN1\|ANXA2\|PDGFA |
| 6140 | 7.14E-03 | 4.35E-02 | 11 | 280 | 237 | 14294 | regulation of nucleotide metabolic process | AKAP12\|EDNRA\|EDN1\|FGD5\|TBC1D2\|EDNRB\|BCL6\|GNAQ\|TBC1D2B\|ADRB1\|ADRB2 |
| 70227 | 7.18E-03 | 4.35E-02 | 2 | 8 | 237 | 14294 | lymphocyte apoptosis | BCL2L11\|AKT1 |
| 35024 | 7.18E-03 | 4.35E-02 | 2 | 8 | 237 | 14294 | negative regulation of Rho protein signal transduction | BCL6\|TNFAIP1 |
| 60391 | 7.18E-03 | 4.35E-02 | 2 | 8 | 237 | 14294 | positive regulation of SMAD protein nuclear translocation | TGFB\|TGFBR1 |
| 48532 | 7.18E-03 | 4.35E-02 | 2 | 8 | 237 | 14294 | anatomical structure arrangement | EGR2\|DMD |
| 23056 | 7.32E-03 | 4.43E-02 | 11 | 281 | 237 | 14294 | positive regulation of signaling process | LYN\|AKAP12\|MAP3K3\|GJA1\|CD4\|AKT2\|TGFB\|KRAS\|ADRB2\|THBS1\|TGFBR1 |
| 72001 | 7.33E-03 | 4.43E-02 | 6 | 103 | 237 | 14294 | renal system development | BCL2L11\|TIPARP\|BCL2\|TCF21\|SLIT2\|TGFBR1 |
| 48520 | 7.52E-03 | 4.53E-02 | 4 | 47 | 237 | 14294 | positive regulation of behavior | AKT2\|TGFB\|SLIT2\|THBS1 |
| 32990 | 7.63E-03 | 4.56E-02 | 10 | 244 | 237 | 14294 | cell part morphogenesis | GJA1\|EGR2\|PARD6B\|SDC2\|BCL2\|ULK2\|MFN2\|DMD\|SLIT2\|GAS7 |
| 42113 | 7.65E-03 | 4.56E-02 | 5 | 74 | 237 | 14294 | B cell activation | CD86\|ITGB1\|KLF6\|BCL6\|BCL2 |
| 42176 | 7.65E-03 | 4.56E-02 | 5 | 74 | 237 | 14294 | regulation of protein catabolic process | GJA1\|AKT1\|NEDD4L\|TIMP3\|SMAD7 |
| 6606 | 7.65E-03 | 4.56E-02 | 5 | 74 | 237 | 14294 | protein import into nucleus | PPP3CA\|BCL6\|CRY2\|TGFB\|AKT1 |
| 33674 | 7.84E-03 | 4.67E-02 | 10 | 245 | 237 | 14294 | positive regulation of kinase activity | EDNRA\|EDN1\|CD4\|TGFB\|AKT1\|KRAS\|ADRB2\|THBS1\|TGFBR1\|TGFBR2 |
| 7010 | 7.88E-03 | 4.67E-02 | 15 | 448 | 237 | 14294 | cytoskeleton organization | UBE2B\|LIMCH1\|TNFAIP1\|CORO2B\|SYNE1\|PREX1\|FGD5\|BCL6\|TMSB4X\|BCL2\|KRAS\|TPPP\|FERMT2\|WASF3\|GAS7 |
| 45429 | 7.91E-03 | 4.67E-02 | 3 | 25 | 237 | 14294 | positive regulation of nitric oxide biosynthetic process | EDN1\|AKT2\|AKT1 |
| 50873 | 7.91E-03 | 4.67E-02 | 3 | 25 | 237 | 14294 | brown fat cell differentiation | LAMA3\|ADRB1\|ADRB2 |
| 31326 | 7.93E-03 | 4.67E-02 | 66 | 3019 | 237 | 14294 | regulation of cellular biosynthetic process | CD86\|PHF2\|BTG2\|BTG1\|MAML2\|CITED2\|PTEN\|TCF21\|AFF3\|ETS1\|AKAP12\|EDNRA\|EDNRB\|ZMIZ1\|AKT2\|PDK4\|AKT1\|SOX6\|PKNOX2\|KLF10\|EDN1\|LIN28\|SOX13\|ETV1\|SREBF2\|TGFBR1\|DKK3\|KAT2B\|ELF1\|CRY2\|NCOA7\|JDP2\|DDX5\|HLF\|ZBTB47\|TGFB\|GATA6\|PDGFA\|ADRB1\|ADRB2\|GATA2\|ZBTB4\|THBS1\|RASD1\|RAD21\|MKNK2\|DMD\|CBX7\|EGR2\|UBE2B\|PCGF5\|HIPK1\|KLF4\|PBX1\|HIPK3\|SMAD7\|PNRC2\|KLF6\|CD4\|NR4A3\|BCL6\|TEF\|GNAQ\|KLF9\|BCL2\|KRAS |
| 33673 | 8.03E-03 | 4.70E-02 | 6 | 105 | 237 | 14294 | negative regulation of kinase activity | CDKN1A\|LATS2\|GNAQ\|SPRY4\|AKT1\|HIPK3 |
| 51251 | 8.03E-03 | 4.70E-02 | 6 | 105 | 237 | 14294 | positive regulation of lymphocyte activation | CD86\|CDKN1A\|CD4\|BCL6\|TGFB\|TGFBR2 |
| 35023 | 8.03E-03 | 4.70E-02 | 6 | 105 | 237 | 14294 | regulation of Rho protein signal transduction | PREX1\|FGD5\|BCL6\|ARHGEF15\|TNFAIP1\|ARHGEF6 |
| 7162 | 8.10E-03 | 4.73E-02 | 4 | 48 | 237 | 14294 | negative regulation of cell adhesion | BCL6\|TGFB\|PTEN\|THBS1 |
| 44419 | 8.52E-03 | 4.96E-02 | 12 | 327 | 237 | 14294 | interspecies interaction between organisms | LYN\|CD86\|ITGB1\|KAT2B\|BNIP3L\|CD4\|DDX3X\|ZC3H7B\|MAGI3\|TGFB\|NEDD4L\|LDLR |
| 7154 | 8.54E-03 | 4.96E-02 | 23 | 812 | 237 | 14294 | cell communication | SEMA5A\|CD86\|KLF10\|ATG3\|EGR2\|CDKN1A\|EDN1\|BMPR2\|FZD4\|TMOD2\|TGFB\|NTN4\|PDGFA\|SNX33\|SNX30\|SLC6A4\|DLL4\|PPP3CA\|GJA1\|SNX2\|FRAS1\|RAB14\|DMD |
| 51241 | 8.54E-03 | 4.96E-02 | 8 | 174 | 237 | 14294 | negative regulation of multicellular organismal process | EDN1\|BMPR2\|ANXA2\|BCL6\|PDGFA\|ADRB1\|ADRB2\|THBS1 |
