# Supplementary material for: Deciphering miR-520c-3p as a probable target for immunometabolism in non-small cell lung cancer using systems biology approach
Source: Oncotarget. 2022 May 24;13:725–46. doi: 10.18632/oncotarget.28233 (PMC9131939; doi:10.18632/oncotarget.28233)
Supplement: Supplementary file 4 [file oncotarget-13-28233-s004.docx]

**Supplementary Table 3: String App analysis of identified miRNAs with their respective targets**

| # background genes | # genes | category | chart color | description | FDR value | genes | network.SUID | nodes.SUID | p-value | PMID | term name | transferred  FDR value | year |
| --- | --- | --- | --- | --- | --- | --- | --- | --- | --- | --- | --- | --- | --- |
| 655 | 127 | GO Process |  | cytokine-mediated signaling pathway | 1.56E-69 | CX3CL1|RELT|TNFRSF1A|TGFB1|CCL1|CCL13|CXCL6|NFKB1|IL1RL1|TNFSF9|CCR7|TRAF2|LIF|TXNDC17|IRAK2|IL36G|IL36B|CCL21|MAP3K8|IL1A|IL1B|IL7|IL11|STAT3|IRF8|SOD1|IL22RA1|ACKR3|IL9|TNFRSF10B|PDCD4|TNFSF4|CSF1R|BAG4|IL17RB|TNFRSF13C|CXCR5|CXCL3|CXCL5|IL17RD|TNFRSF21|IL22RA2|TNFRSF11B|BCL2L1|IL16|IL12A|IL13|SOCS5|IL7R|CXCL11|IL1RAP|TNFRSF12A|CSF1|IFNLR1|IL25|SOCS1|SOCS3|CCR4|IL17F|IL21R|SMAD4|GPR29|IFNAR2|IRF5|CCRL2|CCR9|CCL20|CXCL9|MAP3K3|IRF6|IL20|TAB2|IL6R|MCL1|MAP3K7|IRAK1|TNFRSF6B|IFIT3|IFIT2|IL1RAPL2|CXCR3|IL2RG|HLA-DQB1|TNFSF13B|TNFRSF1B|CCL7|TAB3|IRF4|IL6ST|IL33|JAK2|TNFRSF19|IL17RE|IL17REL|IL36RN|SOCS4|CXCL12|IRF9|SOCS6|BCL2|TNFSF11|CMKBR6|CSF2RB|BCL6|IL6|IL1R1|CXCR4|IL18R1|IRAK4|CXCR6|HLA-B|IL10|ACKR2|IKBKB|TRAF6|HIF1A|IL22|SOD2|TRAF3|TNFRSF11A|CCL5|CCL16|TNFRSF9|SOCS2|SOCS7|CCL4|MAP3K14 | 77 | 325|532|351|350|484|485|531|529|336|120|243|444|432|140|258|561|558|500|165|322|345|266|126|334|398|281|211|247|295|410|311|383|153|409|497|224|306|356|355|136|513|272|359|372|385|519|418|226|176|321|199|340|149|341|478|460|246|502|367|217|470|448|452|160|134|456|308|283|299|487|161|184|348|278|358|434|481|404|394|252|230|563|109|326|276|289|206|169|391|346|309|256|465|479|536|523|181|492|244|475|430|431|524|310|218|274|450|287|371|435|114|425|173|526|315|495|534|381|557|163|135|501|473|419|186|118|171 | 2.98E-73 |  | GO.0019221 | 6.88068754 | 0 |
| 1035 | 142 | GO Process |  | response to cytokine | 1.05E-61 | CX3CL1|RELT|TNFRSF1A|MAPK1|TGFB1|CCL1|CCL13|CXCL6|NFKB1|IL1RL1|TNFSF9|CCR7|TRAF2|LIF|TXNDC17|IRAK2|IL36G|IL36B|CCL21|SMAD7|MAP3K8|IL1A|IL1B|IL7|MAP4K3|IL11|STAT3|LAMP3|IRF8|SOD1|IL22RA1|ACKR3|IL9|TNFRSF10B|PDCD4|TNFSF4|CSF1R|BAG4|IL17RB|TNFRSF13C|CXCR5|CXCL3|CXCL5|IL17RD|TNFRSF21|IL22RA2|TNFRSF11B|BCL2L1|IL16|IL12A|IL13|SOCS5|IL7R|CXCL11|IL1RAP|TNFRSF12A|CSF1|IFNLR1|IL25|SOCS1|SOCS3|CCR4|SMAD3|IL17F|IL21R|SMAD4|GPR29|IFNAR2|IRF5|CCRL2|CCR9|CCL20|MAP3K5|CXCL9|MAP3K3|IRF6|IL20|MAPKAPK2|TAB2|IL6R|MCL1|MAP3K7|IRAK1|TNFRSF6B|IFIT3|IFIT2|IL1RAPL2|CXCR3|TLR4|IL2RG|HLA-DQB1|TNFSF13B|TNFRSF1B|CCL7|TAB3|IRF4|IL6ST|IL33|JAK2|TNFRSF19|IL17RE|IL17REL|TANK|PDCD10|IL36RN|SOCS4|MAPK8|CXCL12|IRF9|SOCS6|MAP2K7|BCL2|TNFSF11|CMKBR6|CSF2RB|BCL6|IL6|IL1R1|CXCR4|IL18R1|IRAK4|CXCR6|HLA-B|IL10|TICAM2|ACKR2|IKBKB|TRAF6|BCLAF1|HIF1A|IL22|SOD2|TRAF3|IKBKE|TNFRSF11A|CCL5|CCL16|TNFRSF9|SOCS2|SOCS7|CCL4|MAP3K14 | 77 | 325|532|351|469|350|484|485|531|529|336|120|243|444|432|140|258|561|558|500|195|165|322|345|266|141|126|334|377|398|281|211|247|295|410|311|383|153|409|497|224|306|356|355|136|513|272|359|372|385|519|418|226|176|321|199|340|149|341|478|460|246|502|241|367|217|470|448|452|160|134|456|308|335|283|299|487|161|268|184|348|278|358|434|481|404|394|252|230|342|563|109|326|276|289|206|169|391|346|309|256|465|479|314|316|536|523|282|181|492|244|236|475|430|431|524|310|218|274|450|287|371|435|114|425|329|173|526|315|180|495|534|381|557|548|163|135|501|473|419|186|118|171 | 4.03E-65 |  | GO.0034097 | 6.09788107 | 0 |
| 953 | 136 | GO Process |  | cellular response to cytokine stimulus | 9.60E-61 | CX3CL1|RELT|TNFRSF1A|MAPK1|TGFB1|CCL1|CCL13|CXCL6|NFKB1|IL1RL1|TNFSF9|CCR7|TRAF2|LIF|TXNDC17|IRAK2|IL36G|IL36B|CCL21|SMAD7|MAP3K8|IL1A|IL1B|IL7|IL11|STAT3|IRF8|SOD1|IL22RA1|ACKR3|IL9|TNFRSF10B|PDCD4|TNFSF4|CSF1R|BAG4|IL17RB|TNFRSF13C|CXCR5|CXCL3|CXCL5|IL17RD|TNFRSF21|IL22RA2|TNFRSF11B|BCL2L1|IL16|IL12A|IL13|SOCS5|IL7R|CXCL11|IL1RAP|TNFRSF12A|CSF1|IFNLR1|IL25|SOCS1|SOCS3|CCR4|SMAD3|IL17F|IL21R|SMAD4|GPR29|IFNAR2|IRF5|CCRL2|CCR9|CCL20|MAP3K5|CXCL9|MAP3K3|IRF6|IL20|TAB2|IL6R|MCL1|MAP3K7|IRAK1|TNFRSF6B|IFIT3|IFIT2|IL1RAPL2|CXCR3|TLR4|IL2RG|HLA-DQB1|TNFSF13B|TNFRSF1B|CCL7|TAB3|IRF4|IL6ST|IL33|JAK2|TNFRSF19|IL17RE|IL17REL|TANK|PDCD10|IL36RN|SOCS4|MAPK8|CXCL12|IRF9|SOCS6|BCL2|TNFSF11|CMKBR6|CSF2RB|BCL6|IL6|IL1R1|CXCR4|IL18R1|IRAK4|CXCR6|HLA-B|IL10|ACKR2|IKBKB|TRAF6|BCLAF1|HIF1A|IL22|SOD2|TRAF3|TNFRSF11A|CCL5|CCL16|TNFRSF9|SOCS2|SOCS7|CCL4|MAP3K14 | 77 | 325|532|351|469|350|484|485|531|529|336|120|243|444|432|140|258|561|558|500|195|165|322|345|266|126|334|398|281|211|247|295|410|311|383|153|409|497|224|306|356|355|136|513|272|359|372|385|519|418|226|176|321|199|340|149|341|478|460|246|502|241|367|217|470|448|452|160|134|456|308|335|283|299|487|161|184|348|278|358|434|481|404|394|252|230|342|563|109|326|276|289|206|169|391|346|309|256|465|479|314|316|536|523|282|181|492|244|475|430|431|524|310|218|274|450|287|371|435|114|425|173|526|315|180|495|534|381|557|163|135|501|473|419|186|118|171 | 5.50E-64 |  | GO.0071345 | 6.001772877 | 0 |
| 2219 | 189 | GO Process |  | cellular response to organic substance | 2.23E-55 | CX3CL1|RELT|TNFRSF1A|TGFBR3|MAPK1|TGFB1|CCL1|CCL13|CXCL6|NFKB1|CDKN1B|IL1RL1|SOGA1|TGFB3|RHOQ|TNFSF9|CCR7|TRAF2|LIF|PIAS1|TXNDC17|IRAK2|PDGFRA|IL36G|IL36B|CCL21|FDX1|PDGFRB|MAPK6|SMAD7|SMAD2|SMURF2|MAP3K8|NOX4|IL1A|IL1B|IL7|IL11|STAT3|IRF8|SOD1|IL22RA1|ACKR3|IL9|TNFRSF10B|PDCD4|TNFSF4|CSF1R|AIFM1|BAG4|IL17RB|SMAD6|TNFRSF13C|CXCR5|CXCL3|CXCL5|SNIP1|IL17RD|TNFRSF21|CREBRF|IL22RA2|TNFRSF11B|AVPR1A|BCL2L1|IL16|IL12A|IL13|SOCS5|IL7R|CXCL11|PPARD|CFLAR|IL1RAP|TNIP1|PRKRA|TNFRSF12A|CSF1|IFNLR1|TGIF1|IL25|SOCS1|SOCS3|CCR4|SMAD3|IL17F|IL21R|SMAD4|LAMTOR4|GPR29|IFNAR2|MAP2K3|ATG7|IRF5|CCRL2|CCR9|CCL20|TGFBR2|MAP3K5|SMURF1|CXCL9|MAP3K3|TGFB2|IRF6|IL20|MAPKAPK2|TAB2|IL6R|MCL1|MAP3K7|IRAK1|TNFRSF6B|IFIT3|IFIT2|IL1RAPL2|AGO3|AGO1|AGO4|CXCR3|TLR4|IL2RG|HLA-DQB1|TGFBR1|TNFSF13B|TNFRSF1B|CCL7|TAB3|IRF4|IL6ST|IL33|JAK2|TNFRSF19|IL17RE|IL17REL|TANK|PDCD10|IL36RN|BCL2L11|SOCS4|MAPK8|CXCL12|IRF9|SOCS6|BCL2|TNFSF11|MAPK4|CMKBR6|CSF2RB|GATSL3|BCL6|PPARA|IL6|IL1R1|CXCR4|IL18R1|NFATC4|TLR6|IRAK4|MAPK9|CXCR6|HLA-B|NCOA2|MAP2K4|IL10|TICAM2|ACKR2|SMAD1|IKBKB|TRAF6|BCLAF1|DICER1|HIF1A|SMAD5|IL22|SOD2|APAF1|TRAF3|LITAF|TNFRSF11A|PIAS2|MAP2K6|CCL5|NOS1|CCL16|TNFRSF9|SOCS2|TNFAIP3|SOCS7|CCL4|MAP3K14 | 77 | 325|532|351|447|469|350|484|485|531|529|285|336|317|265|552|120|243|444|432|554|140|258|516|561|558|500|353|506|235|195|200|362|165|364|322|345|266|126|334|398|281|211|247|295|410|311|383|153|482|409|497|369|224|306|356|355|337|136|513|296|272|359|222|372|385|519|418|226|176|321|227|133|199|538|196|340|149|341|143|478|460|246|502|241|367|217|470|188|448|452|528|119|160|134|456|308|332|335|464|283|299|376|487|161|268|184|348|278|358|434|481|404|394|252|520|239|240|230|342|563|109|368|326|276|289|206|169|391|346|309|256|465|479|314|316|536|234|523|282|181|492|244|475|430|231|431|524|288|310|491|218|274|450|287|249|305|371|250|435|114|455|517|425|329|173|193|526|315|180|121|495|201|534|381|148|557|395|163|284|255|135|508|501|473|419|164|186|118|171 | 1.70E-58 |  | GO.0071310 | 5.465169514 | 0 |
| 263 | 79 | KEGG Pathways | | Cytokine-cytokine receptor interaction | 1.68E-54 | CX3CL1|RELT|TNFRSF1A|TGFB1|CCL1|CCL13|CXCL6|TGFB3|TNFSF10|TNFSF9|CCR7|LIF|PDGFRA|CCL21|PDGFRB|IL1A|IL1B|IL7|IL11|IL22RA1|ACKR3|IL9|TNFRSF10B|TNFSF4|CSF1R|IL17RB|TNFRSF13C|CXCR5|CXCL3|CXCL5|TNFRSF21|IL22RA2|TNFRSF11B|IL12A|IL13|IL7R|CXCL11|TNFRSF12A|CSF1|IFNLR1|IL25|CCR4|CXCL14|IL17F|IL21R|IFNAR2|CCR9|CCL20|TGFBR2|CCL28|CXCL9|TGFB2|IL20|IL6R|CXCR3|IL2RG|TGFBR1|TNFSF13B|TNFRSF1B|CCL7|IL6ST|TNFRSF19|IL17RE|CXCL12|TNFSF11|CMKBR6|CSF2RB|IL6|IL1R1|CXCR4|IL18R1|CXCR6|IL10|IL22|TNFRSF11A|CCL5|CCL16|TNFRSF9|CCL4 | 77 | 325|532|351|350|484|485|531|265|202|120|243|432|516|500|506|322|345|266|126|211|247|295|410|383|153|497|224|306|356|355|513|272|359|519|418|176|321|340|149|341|478|502|468|367|217|452|456|308|332|488|283|376|161|348|230|563|368|326|276|289|391|256|465|181|430|431|524|218|274|450|287|435|425|534|163|135|501|473|118 | 8.53E-57 |  | hsa04060 | 5.377469072 | 0 |
| 2672 | 205 | GO Process |  | cellular response to chemical stimulus | 2.26E-54 | CX3CL1|RELT|TNFRSF1A|TGFBR3|MAPK1|TGFB1|CCL1|CCL13|CXCL6|NFKB1|CDKN1B|IL1RL1|SOGA1|TGFB3|RHOQ|TNFSF9|CCR7|TRAF2|LIF|PIAS1|TXNDC17|PXDN|IRAK2|PDGFRA|HILPDA|IL36G|IL36B|CCL21|FDX1|PDGFRB|MAPK6|SMAD7|SMAD2|SMURF2|MAP3K8|NOX4|IL1A|IL1B|IL7|IL11|STAT3|PRDX5|IRF8|SOD1|IL22RA1|ACKR3|IL9|TNFRSF10B|PDCD4|TNFSF4|CSF1R|AIFM1|BAG4|IL17RB|SMAD6|TNFRSF13C|CXCR5|CXCL3|CXCL5|SNIP1|IL17RD|TNFRSF21|CREBRF|IL22RA2|TNFRSF11B|HIF1AN|AVPR1A|BCL2L1|IL16|IL12A|IL13|SOCS5|IL7R|CXCL11|PPARD|CFLAR|IL1RAP|TNIP1|PRKRA|TNFRSF12A|CSF1|IFNLR1|TGIF1|IL25|SOCS1|SOCS3|CCR4|SMAD3|CXCL14|IL17F|IL21R|SMAD4|LAMTOR4|GPR29|IFNAR2|MAP2K3|RBPJ|ATG7|IRF5|CCRL2|CCR9|CCL20|TGFBR2|MAP3K5|CCL28|GPX6|SMURF1|CXCL9|MAP3K3|TGFB2|IRF6|IL20|MAPKAPK2|TAB2|IL6R|MCL1|MAP3K7|IRAK1|TNFRSF6B|IFIT3|IFIT2|IL1RAPL2|AGO3|AGO1|AGO4|CXCR3|TLR4|IL2RG|TXNDC8|HLA-DQB1|TGFBR1|TNFSF13B|TNFRSF1B|HIF3A|CCL7|TAB3|BNIP3L|IRF4|IL6ST|IL33|JAK2|TNFRSF19|TXNRD3|IL17RE|LYST|IL17REL|TANK|PDCD10|IL36RN|BCL2L11|SOCS4|MAPK8|CXCL12|IRF9|SOCS6|BCL2|TNFSF11|MAPK4|TXNRD2|CMKBR6|CSF2RB|GATSL3|BCL6|PPARA|IL6|IL1R1|CXCR4|IL18R1|NFATC4|TLR6|IRAK4|MAPK9|CXCR6|HLA-B|NCOA2|DOCK4|OXR1|MAP2K4|IL10|TICAM2|ACKR2|SMAD1|IKBKB|TRAF6|BCLAF1|DICER1|HIF1A|SMAD5|IL22|SOD2|APAF1|TRAF3|LITAF|TNFRSF11A|PIAS2|MAP2K6|CCL5|NOS1|CCL16|TNFRSF9|SOCS2|TNFAIP3|SOCS7|CCL4|MAP3K14 | 77 | 325|532|351|447|469|350|484|485|531|529|285|336|317|265|552|120|243|444|432|554|140|543|258|516|462|561|558|500|353|506|235|195|200|362|165|364|322|345|266|126|334|208|398|281|211|247|295|410|311|383|153|482|409|497|369|224|306|356|355|337|136|513|296|272|359|228|222|372|385|519|418|226|176|321|227|133|199|538|196|340|149|341|143|478|460|246|502|241|468|367|217|470|188|448|452|528|438|119|160|134|456|308|332|335|488|388|464|283|299|376|487|161|268|184|348|278|358|434|481|404|394|252|520|239|240|230|342|563|238|109|368|326|276|510|289|206|499|169|391|346|309|256|263|465|555|479|314|316|536|234|523|282|181|492|244|475|430|231|476|431|524|288|310|491|218|274|450|287|249|305|371|250|435|114|455|544|144|517|425|329|173|193|526|315|180|121|495|201|534|381|148|557|395|163|284|255|135|508|501|473|419|164|186|118|171 | 2.16E-57 |  | GO.0070887 | 5.364589156 | 0 |
| 6212 | 312 | GO Process |  | cellular response to stimulus | 7.56E-54 | CX3CL1|MAP4K5|RELT|TNFRSF1A|TGFBR3|MAPK1|TGFB1|CCL1|CCL13|CXCL6|NFKB1|CDKN1B|IL1RL1|BCL9|SOGA1|TGFB3|RHOQ|TNFSF10|RASL11A|EEPD1|TNFSF9|TMX4|CCR7|ATG14|TRAF2|LIF|PIAS1|TXNDC17|BCL2L2|DOCK7|LILRA1|PXDN|MAP3K10|IRAK2|PDGFRA|HILPDA|DOCK10|IL36G|IL36B|CCL21|FDX1|BCL2L10|TRAF5|PDGFRB|MAPK6|FAF2|MMD|WDR59|SMAD7|SMAD2|RIMS2|TRAF4|SMURF2|MAP3K8|NOX4|IL1A|IL1B|IL7|MAP4K3|ATF2|IL11|STAT3|MAP3K13|PRDX5|CDK6|DOCK3|IGSF6|IRF8|SOD1|IL22RA1|ACKR3|IL9|DOCK11|TNFRSF10B|DOCK5|PDCD4|DOCK1|TNFSF4|ATG10|TXNDC11|DEPTOR|CSF1R|AIFM1|BAG4|IL17RB|SMAD6|TNFRSF13C|CXCR5|WDR24|TRAT1|CXCL3|CXCL5|SNIP1|IL17RD|RICTOR|TNFRSF21|CREBRF|IL22RA2|TNFRSF11B|DEFB1|HIF1AN|AVPR1A|TMX3|NFATC3|MAP2K1|BCL2L1|IL16|IL12A|DEFB4A|NKIRAS2|CTLA4|IL13|SOCS5|ALCAM|IL7R|CXCL11|MAP3K11|PPARD|OXSR1|CFLAR|IL1RAP|RHOJ|TNIP1|PRKRA|C8orf4|TNFRSF12A|CSF1|IFNLR1|TGIF1|TNFAIP8L3|PAWR|IL25|SOCS1|SOCS3|AEN|CCR4|SMAD3|LAMP1|BCL9L|CXCL14|IL17F|IL21R|BCL11A|KIR2DL4|MIOS|SMAD4|LAMTOR4|MAP4K4|GPR29|IFNAR2|MAP2K3|RBPJ|MAP3K15|ATG7|BCL11B|IRF5|CCRL2|CCR9|CCL20|TGFBR2|MAP3K5|MAPK10|CCL28|GPX6|SMURF1|CXCL9|MAP3K3|KIAA1804|TGFB2|IRF6|TAGAP|IL20|MAPKAPK2|TAB2|HIVEP2|THEMIS|IL6R|MCL1|ATG5|BAG3|PEX11B|MAP3K7|IGSF3|IRAK1BP1|IRAK1|TNFRSF6B|BCL10|IGSF1|IFIT3|IFIT2|IL1RAPL2|YWHAB|AGO3|AGO1|AGO4|CXCR3|TGIF2|TLR4|IL2RG|TXNDC8|HLA-DQB1|TGFBR1|ZAK|MAP3K19|TNFSF13B|TNFRSF1B|DOCK9|HIF3A|PLCXD3|CCL7|TAB3|TXNDC5|BNIP3L|IRF4|IL6ST|IL33|JAK2|TNFRSF19|TXNRD3|IL17RE|LYST|IL17REL|MAP3K4|TANK|PDCD10|IL36RN|BCL2L11|SOCS4|ULK2|MAPK8|CXCL12|NFATC2|IRF9|BLOC1S5|SOCS6|MAP2K7|BCL2|TNFSF11|MAP3K1|MAPK4|TXNRD2|CMKBR6|CSF2RB|GATSL3|BCL6|RASSF8|PPARA|IL6|LTBP1|IL1R1|CXCR4|IL18R1|MAP3K2|NFATC4|TLR6|IRAK4|TMX1|NKIRAS1|MAPK9|CXCR6|ITK|HLA-B|TNIK|NCOA2|DOCK4|OXR1|LAMP2|MAP2K4|BCL7B|IL10|TICAM2|ACKR2|LAT2|DEFB4B|SMAD1|CDKN2AIP|IKBKB|AMBRA1|ATG13|TRAF6|BCLAF1|DICER1|HIF1A|SMAD5|IL22|RASSF3|SOD2|APAF1|MAP3K12|MAPKAPK5|JKAMP|MAP3K9|MOAP1|TRAF3|NFAT5|LITAF|TXNIP|IKBKE|TNFRSF11A|PIAS2|ERC1|MAP2K6|CCL5|MR1|NOS1|CCL16|TNFRSF9|SOCS2|TNFAIP3|SOCS7|CCL4|MAP3K14 | 77 | 325|451|532|351|447|469|350|484|485|531|529|285|336|175|317|265|552|202|205|197|120|301|243|150|444|432|554|140|551|413|344|543|489|258|516|462|505|561|558|500|353|550|142|506|235|458|427|127|195|200|331|194|362|165|364|322|345|266|141|189|126|334|490|208|365|225|546|398|281|211|247|295|422|410|406|311|215|383|155|156|408|153|482|409|497|369|224|306|260|527|356|355|337|136|302|513|296|272|359|112|228|222|122|210|292|372|385|519|158|273|183|418|226|286|176|321|439|227|504|133|199|375|538|196|293|340|149|341|143|248|396|478|460|246|547|502|241|360|125|468|367|217|507|515|525|470|188|254|448|452|528|438|384|119|128|160|134|456|308|332|335|380|488|388|464|283|299|190|376|487|139|161|268|184|433|349|348|278|312|318|343|358|270|400|434|481|323|132|404|394|252|440|520|239|240|230|259|342|563|238|109|368|264|352|326|276|300|510|407|289|206|459|499|169|391|346|309|256|263|465|555|479|466|314|316|536|234|523|214|282|181|115|492|198|244|236|475|430|397|231|476|431|524|288|310|549|491|218|154|274|450|287|374|249|305|371|390|417|250|435|556|114|535|455|544|144|304|517|174|425|329|173|467|166|193|324|526|131|559|315|180|121|495|201|534|221|381|148|426|370|382|290|138|557|393|395|415|548|163|284|401|255|135|540|508|501|473|419|164|186|118|171 | 8.67E-57 |  | GO.0051716 | 5.31214782 | 0 |
| 3267 | 224 | GO Process |  | response to stress | 5.47E-53 | CX3CL1|MAP4K5|RELT|TNFRSF1A|TGFBR3|MAPK1|TGFB1|CCL1|CCL13|CXCL6|NFKB1|CDKN1B|LOX|BCL9|TGFB3|EEPD1|TMX4|CCR7|ATG14|TRAF2|BCL2L2|LILRA1|PXDN|MAP3K10|IRAK2|PDGFRA|HILPDA|IL36G|IL36B|CCL21|BCL2L10|ARG2|PDGFRB|FAF2|WDR59|SMAD7|SMAD2|MAP3K8|NOX4|IL1A|IL1B|MAP4K3|ATF2|STAT3|MAP3K13|PRDX5|HPS5|IRF8|SOD1|IL22RA1|IL9|DOCK11|TNFRSF10B|DOCK1|TNFSF4|ATG10|TXNDC11|CSF1R|AIFM1|IL17RB|SMAD6|WDR24|TRAT1|CXCL3|CXCL5|TNFRSF21|CREBRF|TNFRSF11B|DEFB1|HIF1AN|AVPR1A|HPS6|TMX3|NFATC3|MAP2K1|BCL2L1|IL12A|DEFB4A|CTLA4|IL13|SOCS5|CXCL11|MAP3K11|PPARD|OXSR1|CFLAR|IL1RAP|TNIP1|PRKRA|C8orf4|CSF1|IFNLR1|IL25|AEN|CCR4|SMAD3|IL17F|KIR2DL4|MIOS|SMAD4|GPR29|IFNAR2|MAP2K3|RBPJ|ATG7|IRF5|CCRL2|CCR9|CCL20|TGFBR2|MAP3K5|MAPK10|GPX6|SMURF1|CXCL9|MAP3K3|KIAA1804|TGFB2|IRF6|MAPKAPK2|TAB2|IL6R|MCL1|ATG5|BAG3|MAP3K7|IRAK1|TNFRSF6B|BCL10|IFI44L|IFIT3|IFIT2|CXCR3|TLR4|TXNDC8|HLA-DQB1|TGFBR1|ZAK|MAP3K19|TNFRSF1B|DOCK9|HIF3A|CCL7|TAB3|TXNDC5|BNIP3L|IRF4|IL33|JAK2|TNFRSF19|TXNRD3|IL17RE|LOXL2|LYST|ATG16L1|TANK|PDCD10|IL36RN|BCL2L11|ULK2|MAPK8|CXCL12|NFATC2|IRF9|SOCS6|MAP2K7|BCL2|TNFSF11|MAP3K1|TXNRD2|CMKBR6|BCL6|PPARA|IL6|IL1R1|CXCR4|IL18R1|MAP3K2|NFATC4|TLR6|IRAK4|TMX1|MAPK9|CXCR6|ITK|HLA-B|TNIK|ILF3|OXR1|LAMP2|MAP2K4|IL10|TICAM2|ACKR2|DEFB4B|SMAD1|CDKN2AIP|IKBKB|AMBRA1|ATG13|TRAF6|BCLAF1|HIF1A|IL22|SOD2|APAF1|MAP3K12|MAPKAPK5|JKAMP|MAP3K9|MOAP1|TRAF3|TXNIP|IKBKE|TNFRSF11A|MAP2K6|CCL5|MR1|NOS1|CCL16|TNFRSF9|TNFAIP3|CCL4|MAP3K14 | 77 | 325|451|532|351|447|469|350|484|485|531|529|285|168|175|265|197|301|243|150|444|551|344|543|489|258|516|462|561|558|500|550|518|506|458|127|195|200|165|364|322|345|141|189|334|490|208|366|398|281|211|295|422|410|215|383|155|156|153|482|497|369|260|527|356|355|513|296|359|112|228|222|209|122|210|292|372|519|158|183|418|226|321|439|227|504|133|199|538|196|293|149|341|478|547|502|241|367|515|525|470|448|452|528|438|119|160|134|456|308|332|335|380|388|464|283|299|190|376|487|268|184|348|278|312|318|358|434|481|323|177|404|394|230|342|238|109|368|264|352|276|300|510|289|206|459|499|169|346|309|256|263|465|480|555|223|314|316|536|234|214|282|181|115|492|244|236|475|430|397|476|431|310|491|218|274|450|287|374|249|305|371|390|250|435|556|114|535|110|144|304|517|425|329|173|166|193|324|526|131|559|315|180|495|534|381|148|426|370|382|290|138|557|415|548|163|255|135|540|508|501|473|164|118|171 | 7.32E-56 |  | GO.0006950 | 5.226201267 | 0 |
| 4738 | 270 | GO Process |  | signal transduction | 8.69E-53 | CX3CL1|MAP4K5|RELT|TNFRSF1A|TGFBR3|MAPK1|TGFB1|CCL1|CCL13|CXCL6|NFKB1|CDKN1B|IL1RL1|BCL9|SOGA1|TGFB3|RHOQ|TNFSF10|RASL11A|TNFSF9|CCR7|TRAF2|LIF|PIAS1|TXNDC17|BCL2L2|DOCK7|LILRA1|MAP3K10|IRAK2|PDGFRA|DOCK10|IL36G|IL36B|CCL21|BCL2L10|TRAF5|PDGFRB|MAPK6|MMD|SMAD7|SMAD2|RIMS2|TRAF4|SMURF2|MAP3K8|IL1A|IL1B|IL7|MAP4K3|IL11|STAT3|MAP3K13|CDK6|DOCK3|IGSF6|IRF8|SOD1|IL22RA1|ACKR3|IL9|DOCK11|TNFRSF10B|DOCK5|PDCD4|DOCK1|TNFSF4|ATG10|DEPTOR|CSF1R|AIFM1|BAG4|IL17RB|SMAD6|TNFRSF13C|CXCR5|TRAT1|CXCL3|CXCL5|SNIP1|IL17RD|RICTOR|TNFRSF21|CREBRF|IL22RA2|TNFRSF11B|DEFB1|AVPR1A|NFATC3|MAP2K1|BCL2L1|IL16|IL12A|DEFB4A|NKIRAS2|CTLA4|IL13|SOCS5|ALCAM|IL7R|CXCL11|MAP3K11|PPARD|OXSR1|IL1RAP|RHOJ|TNIP1|TNFRSF12A|CSF1|IFNLR1|TNFAIP8L3|PAWR|IL25|SOCS1|SOCS3|AEN|CCR4|SMAD3|LAMP1|BCL9L|CXCL14|IL17F|IL21R|BCL11A|KIR2DL4|SMAD4|MAP4K4|GPR29|IFNAR2|MAP2K3|RBPJ|MAP3K15|BCL11B|IRF5|CCRL2|CCR9|CCL20|TGFBR2|MAP3K5|MAPK10|CCL28|SMURF1|CXCL9|MAP3K3|KIAA1804|TGFB2|IRF6|TAGAP|IL20|MAPKAPK2|TAB2|HIVEP2|THEMIS|IL6R|MCL1|BAG3|PEX11B|MAP3K7|IGSF3|IRAK1BP1|IRAK1|TNFRSF6B|BCL10|IGSF1|IFIT3|IFIT2|IL1RAPL2|YWHAB|AGO3|AGO1|AGO4|CXCR3|TGIF2|TLR4|IL2RG|HLA-DQB1|TGFBR1|ZAK|MAP3K19|TNFSF13B|TNFRSF1B|DOCK9|PLCXD3|CCL7|TAB3|BNIP3L|IRF4|IL6ST|IL33|JAK2|TNFRSF19|IL17RE|IL17REL|MAP3K4|TANK|PDCD10|IL36RN|BCL2L11|SOCS4|ULK2|MAPK8|CXCL12|NFATC2|IRF9|SOCS6|MAP2K7|BCL2|TNFSF11|MAP3K1|MAPK4|CMKBR6|CSF2RB|BCL6|RASSF8|PPARA|IL6|LTBP1|IL1R1|CXCR4|IL18R1|MAP3K2|NFATC4|TLR6|IRAK4|NKIRAS1|MAPK9|CXCR6|ITK|HLA-B|TNIK|NCOA2|DOCK4|MAP2K4|BCL7B|IL10|TICAM2|ACKR2|LAT2|DEFB4B|SMAD1|IKBKB|TRAF6|HIF1A|SMAD5|IL22|RASSF3|SOD2|APAF1|MAP3K12|MAPKAPK5|MAP3K9|MOAP1|TRAF3|NFAT5|TXNIP|IKBKE|TNFRSF11A|PIAS2|ERC1|MAP2K6|CCL5|MR1|NOS1|CCL16|TNFRSF9|SOCS2|TNFAIP3|SOCS7|CCL4|MAP3K14 | 77 | 325|451|532|351|447|469|350|484|485|531|529|285|336|175|317|265|552|202|205|120|243|444|432|554|140|551|413|344|489|258|516|505|561|558|500|550|142|506|235|427|195|200|331|194|362|165|322|345|266|141|126|334|490|365|225|546|398|281|211|247|295|422|410|406|311|215|383|155|408|153|482|409|497|369|224|306|527|356|355|337|136|302|513|296|272|359|112|222|210|292|372|385|519|158|273|183|418|226|286|176|321|439|227|504|199|375|538|340|149|341|248|396|478|460|246|547|502|241|360|125|468|367|217|507|515|470|254|448|452|528|438|384|128|160|134|456|308|332|335|380|488|464|283|299|190|376|487|139|161|268|184|433|349|348|278|318|343|358|270|400|434|481|323|132|404|394|252|440|520|239|240|230|259|342|563|109|368|264|352|326|276|300|407|289|206|499|169|391|346|309|256|465|479|466|314|316|536|234|523|214|282|181|115|492|244|236|475|430|397|231|431|524|310|549|491|218|154|274|450|287|374|249|305|371|417|250|435|556|114|535|455|544|517|174|425|329|173|467|166|193|526|315|495|201|534|221|381|148|426|370|290|138|557|393|415|548|163|284|401|255|135|540|508|501|473|419|164|186|118|171 | 1.33E-55 |  | GO.0007165 | 5.206098022 | 0 |
| 2198 | 184 | GO Process |  | cell surface receptor signaling pathway | 8.80E-53 | CX3CL1|RELT|TNFRSF1A|TGFBR3|MAPK1|TGFB1|CCL1|CCL13|CXCL6|NFKB1|CDKN1B|IL1RL1|BCL9|SOGA1|TGFB3|RHOQ|TNFSF10|TNFSF9|CCR7|TRAF2|LIF|PIAS1|TXNDC17|BCL2L2|LILRA1|MAP3K10|IRAK2|PDGFRA|IL36G|IL36B|CCL21|BCL2L10|PDGFRB|SMAD7|SMAD2|SMURF2|MAP3K8|IL1A|IL1B|IL7|IL11|STAT3|IGSF6|IRF8|SOD1|IL22RA1|ACKR3|IL9|TNFRSF10B|PDCD4|DOCK1|TNFSF4|CSF1R|BAG4|IL17RB|SMAD6|TNFRSF13C|CXCR5|TRAT1|CXCL3|CXCL5|IL17RD|TNFRSF21|IL22RA2|TNFRSF11B|NFATC3|BCL2L1|IL16|IL12A|CTLA4|IL13|SOCS5|IL7R|CXCL11|IL1RAP|TNFRSF12A|CSF1|IFNLR1|IL25|SOCS1|SOCS3|CCR4|SMAD3|BCL9L|IL17F|IL21R|SMAD4|GPR29|IFNAR2|RBPJ|IRF5|CCRL2|CCR9|CCL20|TGFBR2|MAPK10|SMURF1|CXCL9|MAP3K3|TGFB2|IRF6|IL20|MAPKAPK2|TAB2|THEMIS|IL6R|MCL1|BAG3|MAP3K7|IGSF3|IRAK1|TNFRSF6B|BCL10|IFIT3|IFIT2|IL1RAPL2|AGO3|AGO1|AGO4|CXCR3|TGIF2|TLR4|IL2RG|HLA-DQB1|TGFBR1|TNFSF13B|TNFRSF1B|CCL7|TAB3|IRF4|IL6ST|IL33|JAK2|TNFRSF19|IL17RE|IL17REL|IL36RN|BCL2L11|SOCS4|MAPK8|CXCL12|NFATC2|IRF9|SOCS6|BCL2|TNFSF11|MAP3K1|CMKBR6|CSF2RB|BCL6|IL6|LTBP1|IL1R1|CXCR4|IL18R1|IRAK4|MAPK9|CXCR6|ITK|HLA-B|TNIK|BCL7B|IL10|ACKR2|LAT2|SMAD1|IKBKB|TRAF6|HIF1A|SMAD5|IL22|SOD2|MAPKAPK5|MOAP1|TRAF3|TXNIP|TNFRSF11A|CCL5|CCL16|TNFRSF9|SOCS2|SOCS7|CCL4|MAP3K14 | 77 | 325|532|351|447|469|350|484|485|531|529|285|336|175|317|265|552|202|120|243|444|432|554|140|551|344|489|258|516|561|558|500|550|506|195|200|362|165|322|345|266|126|334|546|398|281|211|247|295|410|311|215|383|153|409|497|369|224|306|527|356|355|136|513|272|359|210|372|385|519|183|418|226|176|321|199|340|149|341|478|460|246|502|241|125|367|217|470|448|452|438|160|134|456|308|332|380|464|283|299|376|487|161|268|184|349|348|278|318|358|270|434|481|323|404|394|252|520|239|240|230|259|342|563|109|368|326|276|289|206|169|391|346|309|256|465|479|536|234|523|282|181|115|492|244|475|430|397|431|524|310|218|154|274|450|287|371|250|435|556|114|535|174|425|173|467|193|526|315|495|201|534|381|370|138|557|415|163|135|501|473|419|186|118|171 | 1.51E-55 |  | GO.0007166 | 5.205551733 | 0 |
| 5219 | 283 | GO Process |  | cell communication | 1.98E-52 | CX3CL1|MAP4K5|RELT|TNFRSF1A|TGFBR3|MAPK1|TGFB1|CCL1|CCL13|CXCL6|NFKB1|CDKN1B|IL1RL1|BCL9|SOGA1|TGFB3|RHOQ|TNFSF10|RASL11A|TNFSF9|CCR7|ATG14|TRAF2|LIF|PIAS1|TXNDC17|BCL2L2|DOCK7|LILRA1|MAP3K10|EXOC4|IRAK2|PDGFRA|HILPDA|DOCK10|IL36G|IL36B|CCL21|BCL2L10|TRAF5|PDGFRB|MAPK6|MMD|WDR59|SMAD7|SMAD2|RIMS2|TRAF4|SMURF2|MAP3K8|IL1A|IL1B|IL7|MAP4K3|IL11|STAT3|MAP3K13|CDK6|DOCK3|IGSF6|IRF8|SOD1|IL22RA1|ILDR2|ACKR3|IL9|DOCK11|TNFRSF10B|DOCK5|PDCD4|DOCK1|TNFSF4|ATG10|DEPTOR|CSF1R|AIFM1|BAG4|IL17RB|SMAD6|TNFRSF13C|CXCR5|WDR24|TRAT1|CXCL3|CXCL5|SNIP1|IL17RD|RICTOR|TNFRSF21|CREBRF|IL22RA2|TNFRSF11B|DEFB1|AVPR1A|NFATC3|MAP2K1|BCL2L1|IL16|IL12A|DEFB4A|NKIRAS2|CTLA4|IL13|SOCS5|ALCAM|IL7R|CXCL11|MAP3K11|PPARD|OXSR1|IL1RAP|RHOJ|TNIP1|TNFRSF12A|CSF1|IFNLR1|TNFAIP8L3|PAWR|IL25|SOCS1|SOCS3|AEN|CCR4|SMAD3|LAMP1|BCL9L|CXCL14|IL17F|IL21R|BCL11A|KIR2DL4|MIOS|SMAD4|MAP4K4|GPR29|IFNAR2|MAP2K3|RBPJ|MAP3K15|ATG7|BCL11B|IRF5|CCRL2|CCR9|CCL20|TGFBR2|MAP3K5|MAPK10|CPLX2|CCL28|SMURF1|CXCL9|MAP3K3|KIAA1804|TGFB2|IRF6|TAGAP|IL20|MAPKAPK2|TAB2|HIVEP2|THEMIS|IL6R|MCL1|ATG5|BAG3|PEX11B|MAP3K7|IGSF3|IRAK1BP1|IRAK1|TNFRSF6B|BCL10|IGSF1|IFIT3|IFIT2|IL1RAPL2|YWHAB|AGO3|AGO1|AGO4|CXCR3|TGIF2|TLR4|IL2RG|HLA-DQB1|TGFBR1|ZAK|MAP3K19|TNFSF13B|TNFRSF1B|DOCK9|PLCXD3|CCL7|TAB3|BNIP3L|IRF4|IL6ST|IL33|JAK2|TNFRSF19|IL17RE|IL17REL|MAP3K4|TANK|PDCD10|IL36RN|BCL2L11|SOCS4|ULK2|MAPK8|CXCL12|NFATC2|IRF9|SOCS6|MAP2K7|BCL2|TNFSF11|MAP3K1|MAPK4|CMKBR6|CSF2RB|GATSL3|BCL6|RASSF8|PPARA|IL6|LTBP1|IL1R1|CXCR4|IL18R1|MAP3K2|NFATC4|TLR6|IRAK4|NKIRAS1|MAPK9|CXCR6|ITK|HLA-B|TNIK|NCOA2|DOCK4|LAMP2|MAP2K4|BCL7B|IL10|TICAM2|ACKR2|LAT2|DEFB4B|SMAD1|IKBKB|AMBRA1|TRAF6|HIF1A|SMAD5|IL22|RASSF3|SOD2|APAF1|MAP3K12|MAPKAPK5|MAP3K9|MOAP1|TRAF3|NFAT5|TXNIP|IKBKE|TNFRSF11A|PIAS2|ERC1|MAP2K6|CCL5|MR1|NOS1|CCL16|TNFRSF9|SOCS2|TNFAIP3|SOCS7|CCL4|MAP3K14 | 77 | 325|451|532|351|447|469|350|484|485|531|529|285|336|175|317|265|552|202|205|120|243|150|444|432|554|140|551|413|344|489|159|258|516|462|505|561|558|500|550|142|506|235|427|127|195|200|331|194|362|165|322|345|266|141|126|334|490|365|225|546|398|281|211|237|247|295|422|410|406|311|215|383|155|408|153|482|409|497|369|224|306|260|527|356|355|337|136|302|513|296|272|359|112|222|210|292|372|385|519|158|273|183|418|226|286|176|321|439|227|504|199|375|538|340|149|341|248|396|478|460|246|547|502|241|360|125|468|367|217|507|515|525|470|254|448|452|528|438|384|119|128|160|134|456|308|332|335|380|216|488|464|283|299|190|376|487|139|161|268|184|433|349|348|278|312|318|343|358|270|400|434|481|323|132|404|394|252|440|520|239|240|230|259|342|563|109|368|264|352|326|276|300|407|289|206|499|169|391|346|309|256|465|479|466|314|316|536|234|523|214|282|181|115|492|244|236|475|430|397|231|431|524|288|310|549|491|218|154|274|450|287|374|249|305|371|417|250|435|556|114|535|455|544|304|517|174|425|329|173|467|166|193|526|131|315|495|201|534|221|381|148|426|370|290|138|557|393|415|548|163|284|401|255|135|540|508|501|473|419|164|186|118|171 | 3.78E-55 |  | GO.0007154 | 5.170333481 | 0 |
| 2815 | 203 | GO Process |  | response to organic substance | 7.96E-50 | CX3CL1|RELT|TNFRSF1A|TGFBR3|MAPK1|TGFB1|CCL1|CCL13|CXCL6|NFKB1|CDKN1B|LOX|IL1RL1|SOGA1|TGFB3|RHOQ|TNFSF10|TNFSF9|CCR7|TRAF2|LIF|PIAS1|TXNDC17|IRAK2|PDGFRA|IL36G|IL36B|CCL21|FDX1|PDGFRB|MAPK6|LOXL1|FAF2|SMAD7|SMAD2|SMURF2|MAP3K8|NOX4|IL1A|IL1B|IL7|MAP4K3|IL11|STAT3|LAMP3|IRF8|SOD1|IL22RA1|ILDR2|ACKR3|IL9|TNFRSF10B|PDCD4|TNFSF4|CSF1R|AIFM1|BAG4|IL17RB|SMAD6|TNFRSF13C|CXCR5|CXCL3|CXCL5|SNIP1|IL17RD|TNFRSF21|CREBRF|IL22RA2|TNFRSF11B|AVPR1A|BCL2L1|IL16|IL12A|IL13|SOCS5|IL7R|CXCL11|PPARD|CFLAR|IL1RAP|TNIP1|PRKRA|TNFRSF12A|CSF1|IFNLR1|TGIF1|IL25|SOCS1|SOCS3|CCR4|SMAD3|IL17F|IL21R|SMAD4|LAMTOR4|GPR29|IFNAR2|MAP2K3|ATG7|IRF5|CCRL2|CCR9|CCL20|TGFBR2|MAP3K5|SMURF1|CXCL9|MAP3K3|TGFB2|IRF6|IL20|MAPKAPK2|TAB2|IL6R|MCL1|MAP3K7|IRAK1|TNFRSF6B|BCL10|IFIT3|IFIT2|IL1RAPL2|AGO3|AGO1|AGO4|CXCR3|TLR4|IL2RG|HLA-DQB1|TGFBR1|TNFSF13B|TNFRSF1B|CCL7|TAB3|IRF4|IL6ST|IL33|JAK2|TNFRSF19|IL17RE|IL17REL|TANK|PDCD10|IL36RN|BCL2L11|SOCS4|MAPK8|CXCL12|IRF9|SOCS6|MAP2K7|BCL2|TNFSF11|MAPK4|CMKBR6|CSF2RB|GATSL3|BCL6|PPARA|IL6|IL1R1|CXCR4|IL18R1|NFATC4|TLR6|IRAK4|MAPK9|CXCR6|HLA-B|NCOA2|MAP2K4|IL10|TICAM2|ACKR2|SMAD1|IKBKB|TRAF6|BCLAF1|DICER1|HIF1A|SMAD5|IL22|TET2|SOD2|APAF1|JKAMP|TRAF3|LITAF|TXNIP|IKBKE|TNFRSF11A|IER2|PIAS2|MAP2K6|CCL5|NOS1|CCL16|TNFRSF9|SOCS2|TNFAIP3|SOCS7|CCL4|MAP3K14 | 77 | 325|532|351|447|469|350|484|485|531|529|285|168|336|317|265|552|202|120|243|444|432|554|140|258|516|561|558|500|353|506|235|498|458|195|200|362|165|364|322|345|266|141|126|334|377|398|281|211|237|247|295|410|311|383|153|482|409|497|369|224|306|356|355|337|136|513|296|272|359|222|372|385|519|418|226|176|321|227|133|199|538|196|340|149|341|143|478|460|246|502|241|367|217|470|188|448|452|528|119|160|134|456|308|332|335|464|283|299|376|487|161|268|184|348|278|358|434|481|323|404|394|252|520|239|240|230|342|563|109|368|326|276|289|206|169|391|346|309|256|465|479|314|316|536|234|523|282|181|492|244|236|475|430|231|431|524|288|310|491|218|274|450|287|249|305|371|250|435|114|455|517|425|329|173|193|526|315|180|121|495|201|534|294|381|148|382|557|395|415|548|163|354|284|255|135|508|501|473|419|164|186|118|171 | 1.67E-52 |  | GO.0010033 | 4.909908693 | 0 |
| 5108 | 274 | GO Process |  | signaling | 8.13E-49 | CX3CL1|MAP4K5|RELT|TNFRSF1A|TGFBR3|MAPK1|TGFB1|CCL1|CCL13|CXCL6|NFKB1|CDKN1B|IL1RL1|BCL9|SOGA1|TGFB3|RHOQ|TNFSF10|RASL11A|TNFSF9|CCR7|TRAF2|LIF|PIAS1|TXNDC17|BCL2L2|DOCK7|LILRA1|MAP3K10|EXOC4|IRAK2|PDGFRA|HILPDA|DOCK10|IL36G|IL36B|CCL21|BCL2L10|TRAF5|PDGFRB|MAPK6|MMD|SMAD7|SMAD2|RIMS2|TRAF4|SMURF2|MAP3K8|IL1A|IL1B|IL7|MAP4K3|IL11|STAT3|MAP3K13|CDK6|DOCK3|IGSF6|IRF8|SOD1|IL22RA1|ILDR2|ACKR3|IL9|DOCK11|TNFRSF10B|DOCK5|PDCD4|DOCK1|TNFSF4|ATG10|DEPTOR|CSF1R|AIFM1|BAG4|IL17RB|SMAD6|TNFRSF13C|CXCR5|TRAT1|CXCL3|CXCL5|SNIP1|IL17RD|RICTOR|TNFRSF21|CREBRF|IL22RA2|TNFRSF11B|DEFB1|AVPR1A|NFATC3|MAP2K1|BCL2L1|IL16|IL12A|DEFB4A|NKIRAS2|CTLA4|IL13|SOCS5|ALCAM|IL7R|CXCL11|MAP3K11|PPARD|OXSR1|IL1RAP|RHOJ|TNIP1|TNFRSF12A|CSF1|IFNLR1|TNFAIP8L3|PAWR|IL25|SOCS1|SOCS3|AEN|CCR4|SMAD3|LAMP1|BCL9L|CXCL14|IL17F|IL21R|BCL11A|KIR2DL4|SMAD4|MAP4K4|GPR29|IFNAR2|MAP2K3|RBPJ|MAP3K15|BCL11B|IRF5|CCRL2|CCR9|CCL20|TGFBR2|MAP3K5|MAPK10|CPLX2|CCL28|SMURF1|CXCL9|MAP3K3|KIAA1804|TGFB2|IRF6|TAGAP|IL20|MAPKAPK2|TAB2|HIVEP2|THEMIS|IL6R|MCL1|BAG3|PEX11B|MAP3K7|IGSF3|IRAK1BP1|IRAK1|TNFRSF6B|BCL10|IGSF1|IFIT3|IFIT2|IL1RAPL2|YWHAB|AGO3|AGO1|AGO4|CXCR3|TGIF2|TLR4|IL2RG|HLA-DQB1|TGFBR1|ZAK|MAP3K19|TNFSF13B|TNFRSF1B|DOCK9|PLCXD3|CCL7|TAB3|BNIP3L|IRF4|IL6ST|IL33|JAK2|TNFRSF19|IL17RE|IL17REL|MAP3K4|TANK|PDCD10|IL36RN|BCL2L11|SOCS4|ULK2|MAPK8|CXCL12|NFATC2|IRF9|SOCS6|MAP2K7|BCL2|TNFSF11|MAP3K1|MAPK4|CMKBR6|CSF2RB|BCL6|RASSF8|PPARA|IL6|LTBP1|IL1R1|CXCR4|IL18R1|MAP3K2|NFATC4|TLR6|IRAK4|NKIRAS1|MAPK9|CXCR6|ITK|HLA-B|TNIK|NCOA2|DOCK4|MAP2K4|BCL7B|IL10|TICAM2|ACKR2|LAT2|DEFB4B|SMAD1|IKBKB|TRAF6|HIF1A|SMAD5|IL22|RASSF3|SOD2|APAF1|MAP3K12|MAPKAPK5|MAP3K9|MOAP1|TRAF3|NFAT5|TXNIP|IKBKE|TNFRSF11A|PIAS2|ERC1|MAP2K6|CCL5|MR1|NOS1|CCL16|TNFRSF9|SOCS2|TNFAIP3|SOCS7|CCL4|MAP3K14 | 77 | 325|451|532|351|447|469|350|484|485|531|529|285|336|175|317|265|552|202|205|120|243|444|432|554|140|551|413|344|489|159|258|516|462|505|561|558|500|550|142|506|235|427|195|200|331|194|362|165|322|345|266|141|126|334|490|365|225|546|398|281|211|237|247|295|422|410|406|311|215|383|155|408|153|482|409|497|369|224|306|527|356|355|337|136|302|513|296|272|359|112|222|210|292|372|385|519|158|273|183|418|226|286|176|321|439|227|504|199|375|538|340|149|341|248|396|478|460|246|547|502|241|360|125|468|367|217|507|515|470|254|448|452|528|438|384|128|160|134|456|308|332|335|380|216|488|464|283|299|190|376|487|139|161|268|184|433|349|348|278|318|343|358|270|400|434|481|323|132|404|394|252|440|520|239|240|230|259|342|563|109|368|264|352|326|276|300|407|289|206|499|169|391|346|309|256|465|479|466|314|316|536|234|523|214|282|181|115|492|244|236|475|430|397|231|431|524|310|549|491|218|154|274|450|287|374|249|305|371|417|250|435|556|114|535|455|544|517|174|425|329|173|467|166|193|526|315|495|201|534|221|381|148|426|370|290|138|557|393|415|548|163|284|401|255|135|540|508|501|473|419|164|186|118|171 | 1.86E-51 |  | GO.0023052 | 4.808990945 | 0 |
| 654 | 103 | Reactome Pathways | | Cytokine Signaling in Immune system | 3.32E-48 | TNFRSF1A|MAPK1|TGFB1|NFKB1|IL1RL1|TNFSF9|TRAF2|LIF|PIAS1|IRAK2|IL36G|IL36B|MAP3K8|IL1A|IL1B|IL7|ATF2|IL11|STAT3|IRF8|SOD1|IL22RA1|IL9|PDCD4|TNFSF4|CSF1R|IL17RB|TNFRSF13C|IL22RA2|TNFRSF11B|MAP2K1|BCL2L1|IL16|IL12A|NKIRAS2|IL13|SOCS5|IL7R|TNFRSF12A|CSF1|IFNLR1|IL25|SOCS1|SOCS3|SMAD3|IL17F|IL21R|MAP2K3|IRF5|CCL20|MAPK10|MAP3K3|IRF6|IL20|MAPKAPK2|TAB2|IL6R|MCL1|MAP3K7|IRAK1|IFIT3|IFIT2|IL2RG|HLA-DQB1|TNFSF13B|TNFRSF1B|TAB3|IRF4|IL6ST|IL33|JAK2|IL17RE|IL36RN|MAPK8|IRF9|MAP2K7|BCL2|TNFSF11|CSF2RB|BCL6|IL6|IL1R1|IL18R1|IRAK4|EIF4E3|NKIRAS1|MAPK9|HLA-B|MAP2K4|IL10|IKBKB|TRAF6|HIF1A|IL22|SOD2|TRAF3|TNFRSF11A|MAP2K6|CCL5|TNFRSF9|SOCS2|CCL4|MAP3K14 | 77 | 351|469|350|529|336|120|444|432|554|258|561|558|165|322|345|266|189|126|334|398|281|211|295|311|383|153|497|224|272|359|292|372|385|519|273|418|226|176|340|149|341|478|460|246|241|367|217|528|160|308|380|299|487|161|268|184|348|278|358|434|404|394|563|109|326|276|206|169|391|346|309|465|536|282|492|236|475|430|524|310|218|274|287|371|403|417|250|114|517|425|526|315|495|534|381|557|163|255|135|473|419|118|171 | 4.92E-51 |  | HSA-1280215 | 4.747886192 | 0 |
| 7824 | 341 | GO Process |  | response to stimulus | 5.14E-48 | CX3CL1|MAP4K5|RELT|NOX3|TNFRSF1A|TGFBR3|MAPK1|TGFB1|CCL1|CCL13|TNFAIP1|CXCL6|NFKB1|CRTAM|CDKN1B|LOX|IL1RL1|BCL9|SOGA1|TGFB3|RHOQ|TNFSF10|RASL11A|EEPD1|TNFSF9|TMX4|CCR7|ATG14|TRAF2|LIF|PIAS1|TXNDC17|BCL2L2|DOCK7|LILRA1|PXDN|MAP3K10|IRAK2|PDGFRA|HILPDA|DOCK10|IL36G|IL36B|CCL21|FDX1|BCL2L10|TRAF5|ARG2|PDGFRB|MAPK6|LOXL1|FAF2|MMD|WDR59|SMAD7|SMAD2|RIMS2|TRAF4|SMURF2|MAP3K8|NOX4|IL1A|IL1B|IL7|MAP4K3|ATF2|IL11|STAT3|MAP3K13|PRDX5|LAMP3|CDK6|HPS5|DOCK3|IGSF6|IRF8|SOD1|IL22RA1|ILDR2|ACKR3|IL9|DOCK11|TNFRSF10B|DOCK5|PDCD4|DOCK1|TNFSF4|ATG10|TXNDC11|DEPTOR|CSF1R|AIFM1|BAG4|IL17RB|SMAD6|TNFRSF13C|CXCR5|WDR24|TRAT1|CXCL3|CXCL5|SNIP1|IL17RD|RICTOR|TNFRSF21|CREBRF|IL22RA2|TNFRSF11B|DEFB1|IKBIP|HIF1AN|AVPR1A|HPS6|TMX3|NFATC3|MAP2K1|BCL2L1|IL16|IL12A|DEFB4A|NKIRAS2|CTLA4|IL13|SOCS5|ALCAM|IL7R|CXCL11|MAP3K11|PPARD|OXSR1|CFLAR|IL1RAP|RHOJ|TNIP1|PRKRA|ICOS|C8orf4|TNFRSF12A|CSF1|IFNLR1|TGIF1|TNFAIP8L3|PAWR|IL25|SOCS1|SOCS3|AEN|CCR4|SMAD3|LAMP1|BTLA|PDCD1|BCL9L|CXCL14|IL17F|IL21R|BCL11A|KIR2DL4|MIOS|SMAD4|LAMTOR4|MAP4K4|GPR29|IFNAR2|MAP2K3|RBPJ|MAP3K15|ATG7|BCL11B|IRF5|CCRL2|CCR9|CCL20|TGFBR2|MAP3K5|MAPK10|CPLX2|CCL28|GPX6|SMURF1|CXCL9|MAP3K3|ILF2|KIAA1804|TGFB2|IRF6|TAGAP|IL20|MAPKAPK2|TAB2|HIVEP2|THEMIS|IL6R|MCL1|ATG5|BAG3|PEX11B|MAP3K7|IGSF3|IRAK1BP1|IRAK1|TNFRSF6B|BCL10|PI4K2A|IFI44|IFI44L|IGSF1|IFIT3|IFIT2|IL1RAPL2|YWHAB|AGO3|AGO1|AGO4|CXCR3|TGIF2|TLR4|IL2RG|TXNDC8|HLA-DQB1|TGFBR1|ZAK|MAP3K19|TNFSF13B|TNFRSF1B|DOCK9|HIF3A|PLCXD3|CCL7|TAB3|TXNDC5|BNIP3L|NFIB|IRF4|IL6ST|IL33|JAK2|TNFRSF19|TXNRD3|IL17RE|LOXL2|LYST|IL17REL|ATG16L1|MAP3K4|TANK|PDCD10|IL36RN|BCL2L11|SOCS4|ULK2|MAPK8|CXCL12|NFATC2|IRF9|BLOC1S5|PDCD1LG2|SOCS6|MAP2K7|BCL2|TNFSF11|MAP3K1|MAPK4|TXNRD2|CMKBR6|CSF2RB|GATSL3|BCL6|RASSF8|PPARA|IL6|LTBP1|IL1R1|CXCR4|IL18R1|MAP3K2|NFATC4|TLR6|IRAK4|TMX1|NKIRAS1|MAPK9|CXCR6|ITK|HLA-DMB|HLA-B|TNIK|NCOA2|ILF3|DOCK4|OXR1|LAMP2|MAP2K4|BCL7B|IL10|TICAM2|TGFBI|ACKR2|LAT2|DEFB4B|SMAD1|CDKN2AIP|IKBKB|AMBRA1|ATG13|TRAF6|BCLAF1|DICER1|HIF1A|SMAD5|IL22|TET2|RASSF3|SOD2|APAF1|MAP3K12|MAPKAPK5|JKAMP|MAP3K9|MOAP1|TRAF3|NFAT5|LITAF|TXNIP|IKBKE|TNFRSF11A|IER2|PIAS2|ERC1|MAP2K6|CCL5|MR1|NOS1|CCL16|TNFRSF9|ECSCR|SOCS2|TNFAIP3|SOCS7|CCL4|MAP3K14 | 77 | 325|451|532|203|351|447|469|350|484|485|280|531|529|151|285|168|336|175|317|265|552|202|205|197|120|301|243|150|444|432|554|140|551|413|344|543|489|258|516|462|505|561|558|500|353|550|142|518|506|235|498|458|427|127|195|200|331|194|362|165|364|322|345|266|141|189|126|334|490|208|377|365|366|225|546|398|281|211|237|247|295|422|410|406|311|215|383|155|156|408|153|482|409|497|369|224|306|260|527|356|355|337|136|302|513|296|272|359|112|232|228|222|209|122|210|292|372|385|519|158|273|183|418|226|286|176|321|439|227|504|133|199|375|538|196|167|293|340|149|341|143|248|396|478|460|246|547|502|241|360|449|537|125|468|367|217|507|515|525|470|188|254|448|452|528|438|384|119|128|160|134|456|308|332|335|380|216|488|388|464|283|299|443|190|376|487|139|161|268|184|433|349|348|278|312|318|343|358|270|400|434|481|323|399|179|177|132|404|394|252|440|520|239|240|230|259|342|563|238|109|368|264|352|326|276|300|510|407|289|206|459|499|170|169|391|346|309|256|263|465|480|555|479|223|466|314|316|536|234|523|214|282|181|115|492|198|373|244|236|475|430|397|231|476|431|524|288|310|549|491|218|154|274|450|287|374|249|305|371|390|417|250|435|556|553|114|535|455|110|544|144|304|517|174|425|329|157|173|467|166|193|324|526|131|559|315|180|121|495|201|534|294|221|381|148|426|370|382|290|138|557|393|395|415|548|163|354|284|401|255|135|540|508|501|473|253|419|164|186|118|171 | 1.28E-50 |  | GO.0050896 | 4.728903688 | 0 |
| 2054 | 171 | GO Process |  | positive regulation of response to stimulus | 5.39E-48 | CX3CL1|MAP4K5|TNFRSF1A|TGFBR3|MAPK1|TGFB1|CCL1|CCL13|CXCL6|NFKB1|CRTAM|IL1RL1|TGFB3|TNFSF10|CCR7|TRAF2|LIF|MAP3K10|IRAK2|PDGFRA|CCL21|TRAF5|ARG2|PDGFRB|WDR59|SMAD2|RIMS2|TRAF4|SMURF2|MAP3K8|NOX4|IL1B|IL7|MAP4K3|IL11|STAT3|MAP3K13|SOD1|ACKR3|TNFRSF10B|PDCD4|DOCK1|TNFSF4|CSF1R|BAG4|IL17RB|TNFRSF13C|WDR24|TGFA|TRAT1|CXCL3|CXCL5|RICTOR|TNFRSF21|CREBRF|MAP2K1|BCL2L1|IL16|IL12A|CTLA4|IL13|SOCS5|IL7R|CXCL11|MAP3K11|PPARD|CFLAR|TNIP1|PRKRA|ICOS|C8orf4|TNFRSF12A|CSF1|TNFAIP8L3|PAWR|SOCS3|CCR4|SMAD3|LAMP1|CXCL14|IL17F|MIOS|SMAD4|LAMTOR4|MAP4K4|GPR29|MAP2K3|RBPJ|MAP3K15|CCL20|TGFBR2|MAP3K5|MAPK10|CXCL9|MAP3K3|NOS1AP|KIAA1804|TGFB2|IL20|MAPKAPK2|TAB2|THEMIS|IL6R|MCL1|MAP3K7|IRAK1|BCL10|YWHAB|AGO3|AGO1|CXCR3|TLR4|HLA-DQB1|TGFBR1|ZAK|TNFSF13B|CCL7|TAB3|IL6ST|IL33|JAK2|TNFRSF19|MAP3K4|TANK|PDCD10|BCL2L11|MAPK8|CXCL12|NFATC2|MAP2K7|BCL2|TNFSF11|MAP3K1|CMKBR6|IL6|IL1R1|CXCR4|IL18R1|MAP3K2|NFATC4|TLR6|IRAK4|MAPK9|ITK|HLA-DMB|HLA-B|TNIK|MAP2K4|TICAM2|LAT2|CDKN2AIP|IKBKB|TRAF6|BCLAF1|HIF1A|APAF1|MAP3K12|MAPKAPK5|MAP3K9|MOAP1|TRAF3|IKBKE|TNFRSF11A|MAP2K6|CCL5|NOS1|CCL16|SOCS2|TNFAIP3|CCL4|MAP3K14 | 77 | 325|451|351|447|469|350|484|485|531|529|151|336|265|202|243|444|432|489|258|516|500|142|518|506|127|200|331|194|362|165|364|345|266|141|126|334|490|281|247|410|311|215|383|153|409|497|224|260|279|527|356|355|302|513|296|292|372|385|519|183|418|226|176|321|439|227|133|538|196|167|293|340|149|248|396|246|502|241|360|468|367|525|470|188|254|448|528|438|384|308|332|335|380|283|299|212|190|376|161|268|184|349|348|278|358|434|323|440|520|239|230|342|109|368|264|326|289|206|391|346|309|256|466|314|316|234|282|181|115|236|475|430|397|431|218|274|450|287|374|249|305|371|250|556|553|114|535|517|329|467|324|526|315|180|495|148|426|370|290|138|557|548|163|255|135|508|501|419|164|118|171 | 1.44E-50 |  | GO.0048584 | 4.726841123 | 0 |
| 3882 | 232 | GO Process |  | regulation of response to stimulus | 1.40E-45 | CX3CL1|MAP4K5|TNFRSF1A|TGFBR3|MAPK1|TGFB1|CCL1|CCL13|TNFAIP1|CXCL6|NFKB1|CRTAM|IL1RL1|TGFB3|RHOQ|TNFSF10|TNFSF9|CCR7|TRAF2|LIF|PIAS1|BCL2L2|LILRA1|PXDN|MAP3K10|IRAK2|PDGFRA|IL36G|IL36B|CCL21|IER3|BCL2L10|TRAF5|ARG2|PDGFRB|WDR59|SMAD7|SMAD2|RIMS2|TRAF4|SMURF2|MAP3K8|NOX4|IL1A|IL1B|IL7|MAP4K3|IL11|STAT3|MAP3K13|CDK6|SOD1|ACKR3|IL9|TNFRSF10B|PDCD4|DOCK1|TNFSF4|DEPTOR|CSF1R|BAG4|IL17RB|SMAD6|TNFRSF13C|WDR24|TGFA|TRAT1|CXCL3|CXCL5|RICTOR|TNFRSF21|CREBRF|IL22RA2|TNFRSF11B|HIF1AN|NFATC3|MAP2K1|BCL2L1|IL16|IL12A|CTLA4|IL13|SOCS5|IL7R|CXCL11|MAP3K11|PPARD|CFLAR|RHOJ|TNIP1|PRKRA|ICOS|C8orf4|TNFRSF12A|CSF1|IFNLR1|TNFAIP8L3|PAWR|IL25|SOCS1|SOCS3|CCR4|SMAD3|LAMP1|BCL9L|CXCL14|IL17F|BAG5|KIR2DL4|MIOS|SMAD4|NCR3LG1|LAMTOR4|MAP4K4|GPR29|IFNAR2|MAP2K3|RBPJ|MAP3K15|ATG7|CCL20|TGFBR2|MAP3K5|MAPK10|CCL28|SMURF1|CXCL9|MAP3K3|NOS1AP|KIAA1804|TGFB2|TAGAP|IL20|MAPKAPK2|TAB2|THEMIS|IL6R|MCL1|ATG5|BAG3|MAP3K7|IRAK1|BCL10|BAG2|IGSF1|YWHAB|AGO3|AGO1|TRAF3IP1|CXCR3|TLR4|HLA-DQB1|TGFBR1|ZAK|TNFSF13B|TNFRSF1B|CCL7|TAB3|IRF4|IL6ST|IL33|JAK2|TNFRSF19|MAP3K4|TANK|PDCD10|IL36RN|BCL2L11|SOCS4|MAPK8|CXCL12|NFATC2|SOCS6|MAP2K7|BCL2|TNFSF11|MAP3K1|CMKBR6|GATSL3|BCL6|PPARA|IL6|LTBP1|IL1R1|CXCR4|IL18R1|MAP3K2|NFATC4|TLR6|IRAK4|MAPKBP1|MAPK9|OSGIN2|ITK|HLA-DMB|HLA-B|TNIK|OXR1|MAP2K4|IL10|TICAM2|PEX5L|LAT2|CDKN2AIP|IKBKB|TRAF6|BCLAF1|HIF1A|IL22|TNFAIP8L1|SOD2|APAF1|MAP3K12|MAPKAPK5|MAP3K9|MOAP1|TRAF3|NFAT5|LITAF|IKBKE|TNFRSF11A|PIAS2|MAP2K6|CCL5|NOS1|CCL16|SOCS2|TNFAIP3|SOCS7|CCL4|MAP3K14|GATSL2 | 77 | 325|451|351|447|469|350|484|485|280|531|529|151|336|265|552|202|120|243|444|432|554|551|344|543|489|258|516|561|558|500|461|550|142|518|506|127|195|200|331|194|362|165|364|322|345|266|141|126|334|490|365|281|247|295|410|311|215|383|408|153|409|497|369|224|260|279|527|356|355|302|513|296|272|359|228|210|292|372|385|519|183|418|226|176|321|439|227|133|375|538|196|167|293|340|149|341|248|396|478|460|246|502|241|360|125|468|367|124|515|525|470|313|188|254|448|452|528|438|384|119|308|332|335|380|488|464|283|299|212|190|376|139|161|268|184|349|348|278|312|318|358|434|323|116|132|440|520|239|474|230|342|109|368|264|326|276|289|206|169|391|346|309|256|466|314|316|536|234|523|282|181|115|244|236|475|430|397|431|288|310|491|218|154|274|450|287|374|249|305|371|298|250|446|556|553|114|535|144|517|425|329|328|467|324|526|315|180|495|534|453|381|148|426|370|290|138|557|393|395|548|163|284|255|135|508|501|419|164|186|118|171|402 | 4.00E-48 |  | GO.0048583 | 4.485387196 | 0 |
| 3033 | 200 | GO Process |  | regulation of signal transduction | 3.41E-43 | CX3CL1|MAP4K5|TNFRSF1A|TGFBR3|MAPK1|TGFB1|CCL1|CCL13|TNFAIP1|CXCL6|NFKB1|IL1RL1|TGFB3|RHOQ|TNFSF10|TNFSF9|CCR7|TRAF2|LIF|PIAS1|BCL2L2|PXDN|MAP3K10|IRAK2|PDGFRA|IL36G|IL36B|CCL21|IER3|BCL2L10|TRAF5|PDGFRB|WDR59|SMAD7|SMAD2|RIMS2|TRAF4|SMURF2|MAP3K8|NOX4|IL1A|IL1B|IL7|MAP4K3|IL11|STAT3|MAP3K13|SOD1|ACKR3|IL9|TNFRSF10B|PDCD4|TNFSF4|DEPTOR|CSF1R|BAG4|SMAD6|WDR24|TGFA|TRAT1|CXCL3|CXCL5|RICTOR|CREBRF|IL22RA2|TNFRSF11B|HIF1AN|MAP2K1|BCL2L1|IL16|IL12A|IL13|SOCS5|IL7R|CXCL11|MAP3K11|PPARD|CFLAR|RHOJ|TNIP1|PRKRA|ICOS|C8orf4|TNFRSF12A|CSF1|TNFAIP8L3|PAWR|IL25|SOCS1|SOCS3|SMAD3|BCL9L|CXCL14|IL17F|BAG5|MIOS|SMAD4|LAMTOR4|MAP4K4|IFNAR2|MAP2K3|RBPJ|MAP3K15|CCL20|TGFBR2|MAP3K5|MAPK10|CCL28|SMURF1|CXCL9|MAP3K3|NOS1AP|KIAA1804|TGFB2|TAGAP|IL20|MAPKAPK2|TAB2|IL6R|MCL1|MAP3K7|IRAK1|BCL10|IGSF1|YWHAB|AGO3|AGO1|TRAF3IP1|CXCR3|TLR4|TGFBR1|ZAK|TNFSF13B|CCL7|TAB3|IRF4|IL6ST|IL33|JAK2|TNFRSF19|MAP3K4|TANK|PDCD10|IL36RN|BCL2L11|SOCS4|MAPK8|CXCL12|SOCS6|MAP2K7|BCL2|TNFSF11|MAP3K1|GATSL3|BCL6|IL6|LTBP1|IL1R1|CXCR4|IL18R1|MAP3K2|NFATC4|TLR6|IRAK4|MAPKBP1|MAPK9|OSGIN2|TNIK|MAP2K4|IL10|TICAM2|PEX5L|CDKN2AIP|IKBKB|TRAF6|BCLAF1|HIF1A|IL22|TNFAIP8L1|SOD2|APAF1|MAP3K12|MAPKAPK5|MAP3K9|MOAP1|NFAT5|LITAF|IKBKE|TNFRSF11A|PIAS2|MAP2K6|CCL5|NOS1|CCL16|SOCS2|TNFAIP3|SOCS7|CCL4|MAP3K14|GATSL2 | 77 | 325|451|351|447|469|350|484|485|280|531|529|336|265|552|202|120|243|444|432|554|551|543|489|258|516|561|558|500|461|550|142|506|127|195|200|331|194|362|165|364|322|345|266|141|126|334|490|281|247|295|410|311|383|408|153|409|369|260|279|527|356|355|302|296|272|359|228|292|372|385|519|418|226|176|321|439|227|133|375|538|196|167|293|340|149|248|396|478|460|246|241|125|468|367|124|525|470|188|254|452|528|438|384|308|332|335|380|488|464|283|299|212|190|376|139|161|268|184|348|278|358|434|323|132|440|520|239|474|230|342|368|264|326|289|206|169|391|346|309|256|466|314|316|536|234|523|282|181|244|236|475|430|397|288|310|218|154|274|450|287|374|249|305|371|298|250|446|535|517|425|329|328|324|526|315|180|495|534|453|381|148|426|370|290|138|393|395|548|163|284|255|135|508|501|419|164|186|118|171|402 | 1.04E-45 |  | GO.0009966 | 4.246724562 | 0 |
| 1501 | 140 | GO Process |  | regulation of apoptotic process | 6.21E-43 | CX3CL1|MAP4K5|RELT|TNFRSF1A|TGFB1|NFKB1|CDKN1B|TGFB3|TNFSF10|CCR7|TRAF2|PIAS1|BCL2L2|MAP3K10|CCL21|IER3|BCL2L10|TRAF5|ARG2|PDGFRB|TRAF4|MAP3K8|NOX4|IL1A|IL1B|IL7|MAP4K3|ATF2|STAT3|PRDX5|LAMP3|BNIP2|SOD1|ACKR3|TNFRSF10B|PDCD4|DEPTOR|CSF1R|AIFM1|BAG4|SMAD6|TNFRSF21|TNFRSF11B|MAP2K1|BCL2L1|MAEA|IL12A|CTLA4|IL13|IL7R|AVEN|MAP3K11|PPARD|OXSR1|CFLAR|PRKRA|C8orf4|TNFRSF12A|TNFAIP8L3|PAWR|SOCS3|SMAD3|PDCD1|BAG5|MAP4K4|IKZF3|MAP2K3|ATG7|AREL1|BCL11B|IRF5|MAP3K5|MAP3K3|TGFB2|MCL1|ATG5|BAG3|IRAK1|TNFRSF6B|BCL10|IFIT3|IFIT2|YWHAB|AGO4|CXCR3|TGFBR1|ZAK|MAP3K19|TNFRSF1B|TXNDC5|CAAP1|BNIP3L|IL6ST|JAK2|MAP3K4|PDCD10|BCL2L11|BCL2L15|CIAPIN1|MAPK8|CXCL12|MAP2K7|BCL2|MAP3K1|BCL6|IL6|MAP3K2|NFATC4|MAPK9|TNIK|OXR1|MAP2K4|IL10|PXT1|TNFAIP8|IKBKB|API5|AMBRA1|TRAF6|BCLAF1|HIF1A|RASSF3|TNFAIP8L1|SOD2|APAF1|MAP3K12|MAP3K9|MOAP1|TRAF3|TXNIP|TNFRSF11A|ERC1|MAP2K6|CCL5|TNFRSF9|ECSCR|PANO|SOCS2|TNFAIP3|MAP3K14 | 77 | 325|451|532|351|350|529|285|265|202|243|444|554|551|489|500|461|550|142|518|506|194|165|364|322|345|266|141|189|334|208|377|187|281|247|410|311|408|153|482|409|369|513|359|292|372|207|519|183|418|176|145|439|227|504|133|196|293|340|248|396|246|241|537|124|254|392|528|119|437|128|160|335|299|376|278|312|318|434|481|323|404|394|440|240|230|368|264|352|276|459|428|499|391|309|466|316|234|320|472|282|181|236|475|397|310|218|374|249|250|535|144|517|425|363|185|526|463|131|315|180|495|221|453|381|148|426|290|138|557|415|163|401|255|135|473|253|172|419|164|171 | 2.02E-45 |  | GO.0042981 | 4.22069084 | 0 |
| 5459 | 273 | GO Process |  | positive regulation of biological process | 1.29E-42 | CX3CL1|MAP4K5|TNFRSF1A|TGFBR3|MAPK1|TGFB1|CCL1|CCL13|TNFAIP1|CXCL6|NFKB1|CRTAM|CDKN1B|IL1RL1|BCL9|TGFB3|RHOQ|TNFSF10|RASL11A|TNFSF9|CCR7|ATG14|TRAF2|LIF|PIAS1|DOCK7|MAP3K10|XPO4|IRAK2|PDGFRA|HILPDA|TBRG4|CCL21|BCL2L10|TRAF5|ARG2|PDGFRB|MAPK6|MMD|MSR1|WDR59|SMAD7|SMAD2|RIMS2|TRAF4|SMURF2|MAP3K8|NOX4|IL1A|IL1B|IL7|MAP4K3|ATF2|IL11|STAT3|MAP3K13|PRDX5|LAMP3|CDK6|CCAR1|DOCK3|BNIP2|IRF8|SOD1|ACKR3|IL9|DOCK11|TNFRSF10B|DOCK5|PDCD4|DOCK1|TNFSF4|ATG10|CSF1R|AIFM1|BAG4|IL17RB|SMAD6|TNFRSF13C|CXCR5|WDR24|TGFA|TRAT1|CXCL3|CXCL5|RICTOR|TNFRSF21|CREBRF|DEFB1|HIF1AN|AVPR1A|NFATC3|LTBP3|MAP2K1|BCL2L1|IL16|IL12A|CTLA4|IL13|SOCS5|IL7R|CXCL11|MAP3K11|PPARD|OXSR1|CFLAR|IL1RAP|RHOJ|TNIP1|PRKRA|ICOS|C8orf4|NFATC2IP|TNFRSF12A|CSF1|TNFAIP8L3|PAWR|IL25|SOCS1|SOCS3|IKZF1|CCR4|SMAD3|LAMP1|BTLA|PDCD1|BCL9L|CXCL14|IL17F|BCL11A|MIOS|SMAD4|LAMTOR4|MAP4K4|GPR29|IKZF3|MAP2K3|RBPJ|MAP3K15|ATG7|BCL11B|IRF5|FXR1|CCL20|TGFBR2|MAP3K5|MAPK10|CCL28|SMURF1|CXCL9|MAP3K3|ILF2|NOS1AP|KIAA1804|TGFB2|IRF6|IL20|MAPKAPK2|TAB2|THEMIS|IL6R|MCL1|ATG5|BAG3|CACUL1|MAP3K7|MTCP1|IRAK1|BCL10|BAG2|IFIT2|YWHAB|AGO3|AGO1|CXCR3|TGIF2|TLR4|HLA-DQB1|TGFBR1|ZAK|MAP3K19|TNFSF13B|TNFRSF1B|HIF3A|CCL7|TAB3|BNIP3L|NFIB|IRF4|IL6ST|IL33|JAK2|TNFRSF19|LOXL2|MAP3K4|TANK|PDCD10|BCL2L11|PRC1|AKTIP|SOCS4|MAPK8|CXCL12|NFATC2|BLOC1S5|PDCD1LG2|MAP2K7|BCL2|TNFSF11|MAP3K1|CMKBR6|BCL6|PPARA|IL6|IL1R1|CXCR4|IL18R1|MAP3K2|NFATC4|TLR6|IRAK4|MAPK9|ITK|HLA-DMB|HLA-B|TNIK|NCOA2|ILF3|DOCK4|MAP2K4|PDCD6IP|IL10|IKZF2|TICAM2|PXT1|LAT2|SMAD1|CDKN2AIP|TNFAIP8|IKBKB|AMBRA1|TRAF6|BCLAF1|DICER1|HIF1A|SMAD5|NKRF|TET2|SOD2|APAF1|MAP3K12|MAPKAPK5|MAP3K9|MOAP1|TRAF3|NFAT5|LITAF|TXNIP|IKBKE|TNFRSF11A|IER2|PIAS2|ERC1|MAP2K6|CCL5|NOS1|CCL16|ECSCR|PANO|SOCS2|TNFAIP3|CCL4|MAP3K14 | 77 | 325|451|351|447|469|350|484|485|280|531|529|151|285|336|175|265|552|202|205|120|243|150|444|432|554|413|489|386|258|516|462|496|500|550|142|518|506|235|427|147|127|195|200|331|194|362|165|364|322|345|266|141|189|126|334|490|208|377|365|111|225|187|398|281|247|295|422|410|406|311|215|383|155|153|482|409|497|369|224|306|260|279|527|356|355|302|513|296|112|228|222|210|441|292|372|385|519|183|418|226|176|321|439|227|504|133|199|375|538|196|167|293|347|340|149|248|396|478|460|246|220|502|241|360|449|537|125|468|367|507|525|470|188|254|448|392|528|438|384|119|128|160|245|308|332|335|380|488|464|283|299|443|212|190|376|487|161|268|184|349|348|278|312|318|522|358|162|434|323|116|394|440|520|239|230|259|342|109|368|264|352|326|276|510|289|206|499|170|169|391|346|309|256|480|466|314|316|234|511|424|523|282|181|115|198|373|236|475|430|397|431|310|491|218|274|450|287|374|249|305|371|250|556|553|114|535|455|110|544|517|307|425|486|329|363|467|193|324|185|526|131|315|180|121|495|201|291|294|381|148|426|370|290|138|557|393|395|415|548|163|354|284|401|255|135|508|501|253|172|419|164|118|171 | 4.45E-45 |  | GO.0048518 | 4.188941029 | 0 |
| 4898 | 257 | GO Process |  | positive regulation of cellular process | 1.79E-42 | CX3CL1|MAP4K5|TNFRSF1A|TGFBR3|MAPK1|TGFB1|CCL1|CCL13|TNFAIP1|CXCL6|NFKB1|CRTAM|CDKN1B|IL1RL1|BCL9|TGFB3|RHOQ|TNFSF10|RASL11A|TNFSF9|CCR7|ATG14|TRAF2|LIF|PIAS1|DOCK7|MAP3K10|IRAK2|PDGFRA|HILPDA|TBRG4|CCL21|BCL2L10|TRAF5|ARG2|PDGFRB|MAPK6|MMD|MSR1|WDR59|SMAD7|SMAD2|RIMS2|TRAF4|SMURF2|MAP3K8|NOX4|IL1A|IL1B|IL7|MAP4K3|ATF2|IL11|STAT3|MAP3K13|CDK6|CCAR1|DOCK3|BNIP2|IRF8|SOD1|ACKR3|IL9|DOCK11|TNFRSF10B|DOCK5|PDCD4|DOCK1|TNFSF4|ATG10|CSF1R|AIFM1|BAG4|IL17RB|SMAD6|TNFRSF13C|CXCR5|WDR24|TGFA|TRAT1|CXCL3|CXCL5|RICTOR|CREBRF|DEFB1|HIF1AN|AVPR1A|NFATC3|LTBP3|MAP2K1|BCL2L1|IL12A|CTLA4|IL13|SOCS5|IL7R|CXCL11|MAP3K11|PPARD|OXSR1|CFLAR|IL1RAP|RHOJ|TNIP1|PRKRA|ICOS|C8orf4|NFATC2IP|TNFRSF12A|CSF1|TNFAIP8L3|PAWR|IL25|SOCS1|SOCS3|IKZF1|SMAD3|LAMP1|BTLA|PDCD1|BCL9L|CXCL14|IL17F|BCL11A|MIOS|SMAD4|LAMTOR4|MAP4K4|GPR29|IKZF3|MAP2K3|RBPJ|MAP3K15|ATG7|BCL11B|IRF5|FXR1|CCL20|TGFBR2|MAP3K5|MAPK10|SMURF1|CXCL9|MAP3K3|ILF2|NOS1AP|KIAA1804|TGFB2|IRF6|IL20|MAPKAPK2|TAB2|IL6R|MCL1|CACUL1|MAP3K7|MTCP1|IRAK1|BCL10|BAG2|IFIT2|YWHAB|AGO3|AGO1|CXCR3|TGIF2|TLR4|TGFBR1|ZAK|MAP3K19|TNFSF13B|TNFRSF1B|HIF3A|CCL7|TAB3|BNIP3L|NFIB|IRF4|IL6ST|IL33|JAK2|TNFRSF19|LOXL2|MAP3K4|TANK|PDCD10|BCL2L11|PRC1|AKTIP|SOCS4|MAPK8|CXCL12|NFATC2|BLOC1S5|PDCD1LG2|MAP2K7|BCL2|TNFSF11|MAP3K1|CMKBR6|BCL6|PPARA|IL6|IL1R1|CXCR4|IL18R1|MAP3K2|NFATC4|TLR6|IRAK4|MAPK9|HLA-DMB|TNIK|NCOA2|ILF3|DOCK4|MAP2K4|PDCD6IP|IL10|IKZF2|TICAM2|PXT1|SMAD1|CDKN2AIP|TNFAIP8|IKBKB|AMBRA1|TRAF6|BCLAF1|DICER1|HIF1A|SMAD5|NKRF|TET2|SOD2|APAF1|MAP3K12|MAPKAPK5|MAP3K9|MOAP1|NFAT5|LITAF|TXNIP|IKBKE|TNFRSF11A|IER2|PIAS2|MAP2K6|CCL5|NOS1|CCL16|ECSCR|PANO|SOCS2|TNFAIP3|CCL4|MAP3K14 | 77 | 325|451|351|447|469|350|484|485|280|531|529|151|285|336|175|265|552|202|205|120|243|150|444|432|554|413|489|258|516|462|496|500|550|142|518|506|235|427|147|127|195|200|331|194|362|165|364|322|345|266|141|189|126|334|490|365|111|225|187|398|281|247|295|422|410|406|311|215|383|155|153|482|409|497|369|224|306|260|279|527|356|355|302|296|112|228|222|210|441|292|372|519|183|418|226|176|321|439|227|504|133|199|375|538|196|167|293|347|340|149|248|396|478|460|246|220|241|360|449|537|125|468|367|507|525|470|188|254|448|392|528|438|384|119|128|160|245|308|332|335|380|464|283|299|443|212|190|376|487|161|268|184|348|278|522|358|162|434|323|116|394|440|520|239|230|259|342|368|264|352|326|276|510|289|206|499|170|169|391|346|309|256|480|466|314|316|234|511|424|523|282|181|115|198|373|236|475|430|397|431|310|491|218|274|450|287|374|249|305|371|250|553|535|455|110|544|517|307|425|486|329|363|193|324|185|526|131|315|180|121|495|201|291|294|381|148|426|370|290|138|393|395|415|548|163|354|284|255|135|508|501|253|172|419|164|118|171 | 6.84E-45 |  | GO.0048522 | 4.174714697 | 0 |
| 959 | 113 | GO Process |  | positive regulation of intracellular signal transduction | 1.96E-42 | CX3CL1|MAP4K5|TNFRSF1A|MAPK1|TGFB1|CCL1|CCL13|TGFB3|TNFSF10|CCR7|TRAF2|LIF|MAP3K10|IRAK2|PDGFRA|CCL21|TRAF5|PDGFRB|WDR59|TRAF4|MAP3K8|NOX4|IL1B|MAP4K3|IL11|STAT3|MAP3K13|SOD1|ACKR3|TNFRSF10B|PDCD4|CSF1R|BAG4|WDR24|TGFA|TRAT1|RICTOR|MAP2K1|BCL2L1|IL12A|IL13|IL7R|CXCL11|MAP3K11|PPARD|CFLAR|PRKRA|ICOS|C8orf4|CSF1|TNFAIP8L3|SOCS3|MIOS|LAMTOR4|MAP4K4|MAP2K3|MAP3K15|CCL20|MAP3K5|MAPK10|CXCL9|MAP3K3|NOS1AP|KIAA1804|TGFB2|IL20|MAPKAPK2|TAB2|IL6R|MCL1|MAP3K7|IRAK1|BCL10|AGO3|AGO1|CXCR3|TLR4|TGFBR1|ZAK|CCL7|TAB3|IL6ST|JAK2|TNFRSF19|MAP3K4|PDCD10|BCL2L11|MAP2K7|BCL2|TNFSF11|MAP3K1|IL6|CXCR4|IL18R1|MAP3K2|TLR6|IRAK4|TNIK|MAP2K4|IKBKB|TRAF6|BCLAF1|MAP3K12|MAPKAPK5|MAP3K9|IKBKE|TNFRSF11A|MAP2K6|CCL5|NOS1|CCL16|CCL4|MAP3K14 | 77 | 325|451|351|469|350|484|485|265|202|243|444|432|489|258|516|500|142|506|127|194|165|364|345|141|126|334|490|281|247|410|311|153|409|260|279|527|302|292|372|519|418|176|321|439|227|133|196|167|293|149|248|246|525|188|254|528|384|308|335|380|283|299|212|190|376|161|268|184|348|278|358|434|323|520|239|230|342|368|264|289|206|391|309|256|466|316|234|236|475|430|397|218|450|287|374|305|371|535|517|526|315|180|426|370|290|548|163|255|135|508|501|118|171 | 7.84E-45 |  | GO.1902533 | 4.170774393 | 0 |
| 439 | 81 | Reactome Pathways | | Signaling by Interleukins | 9.05E-42 | TNFRSF1A|MAPK1|TGFB1|NFKB1|IL1RL1|LIF|IRAK2|IL36G|IL36B|MAP3K8|IL1A|IL1B|IL7|ATF2|IL11|STAT3|SOD1|IL22RA1|IL9|PDCD4|CSF1R|IL17RB|IL22RA2|MAP2K1|BCL2L1|IL16|IL12A|NKIRAS2|IL13|SOCS5|IL7R|CSF1|IFNLR1|IL25|SOCS1|SOCS3|SMAD3|IL17F|IL21R|MAP2K3|CCL20|MAPK10|MAP3K3|IL20|MAPKAPK2|TAB2|IL6R|MCL1|MAP3K7|IRAK1|IL2RG|TNFRSF1B|TAB3|IRF4|IL6ST|IL33|JAK2|IL17RE|IL36RN|MAPK8|MAP2K7|BCL2|CSF2RB|BCL6|IL6|IL1R1|IL18R1|IRAK4|NKIRAS1|MAPK9|MAP2K4|IL10|IKBKB|TRAF6|HIF1A|IL22|SOD2|MAP2K6|CCL5|SOCS2|CCL4 | 77 | 351|469|350|529|336|432|258|561|558|165|322|345|266|189|126|334|281|211|295|311|153|497|272|292|372|385|519|273|418|226|176|149|341|478|460|246|241|367|217|528|308|380|299|161|268|184|348|278|358|434|563|276|206|169|391|346|309|465|536|282|236|475|524|310|218|274|287|371|417|250|517|425|526|315|495|534|381|255|135|419|118 | 2.68E-44 |  | HSA-449147 | 4.104335142 | 0 |
| 1638 | 144 | GO Process |  | regulation of cell death | 1.17E-41 | CX3CL1|MAP4K5|RELT|TNFRSF1A|TGFB1|NFKB1|CDKN1B|TGFB3|TNFSF10|CCR7|TRAF2|PIAS1|BCL2L2|MAP3K10|CCL21|IER3|BCL2L10|TRAF5|ARG2|PDGFRB|TRAF4|MAP3K8|NOX4|IL1A|IL1B|IL7|MAP4K3|ATF2|STAT3|PRDX5|LAMP3|BNIP2|SOD1|ACKR3|TNFRSF10B|PDCD4|DEPTOR|CSF1R|AIFM1|BAG4|SMAD6|TNFRSF21|TNFRSF11B|MAP2K1|BCL2L1|MAEA|IL12A|CTLA4|IL13|IL7R|AVEN|MAP3K11|PPARD|OXSR1|CFLAR|PRKRA|C8orf4|TNFRSF12A|TNFAIP8L3|PAWR|SOCS3|SMAD3|PDCD1|BAG5|SMAD4|MAP4K4|IKZF3|MAP2K3|ATG7|AREL1|BCL11B|IRF5|MAP3K5|MAP3K3|TGFB2|MCL1|ATG5|BAG3|IRAK1|TNFRSF6B|BCL10|IFIT3|IFIT2|YWHAB|AGO4|CXCR3|TLR4|TGFBR1|ZAK|MAP3K19|TNFRSF1B|TXNDC5|CAAP1|BNIP3L|IL6ST|JAK2|MAP3K4|PDCD10|BCL2L11|BCL2L15|CIAPIN1|MAPK8|CXCL12|MAP2K7|BCL2|MAP3K1|BCL6|PPARA|IL6|MAP3K2|NFATC4|TLR6|MAPK9|TNIK|OXR1|MAP2K4|IL10|PXT1|TNFAIP8|IKBKB|API5|AMBRA1|TRAF6|BCLAF1|HIF1A|RASSF3|TNFAIP8L1|SOD2|APAF1|MAP3K12|MAP3K9|MOAP1|TRAF3|TXNIP|TNFRSF11A|ERC1|MAP2K6|CCL5|TNFRSF9|ECSCR|PANO|SOCS2|TNFAIP3|MAP3K14 | 77 | 325|451|532|351|350|529|285|265|202|243|444|554|551|489|500|461|550|142|518|506|194|165|364|322|345|266|141|189|334|208|377|187|281|247|410|311|408|153|482|409|369|513|359|292|372|207|519|183|418|176|145|439|227|504|133|196|293|340|248|396|246|241|537|124|470|254|392|528|119|437|128|160|335|299|376|278|312|318|434|481|323|404|394|440|240|230|342|368|264|352|276|459|428|499|391|309|466|316|234|320|472|282|181|236|475|397|310|491|218|374|249|305|250|535|144|517|425|363|185|526|463|131|315|180|495|221|453|381|148|426|290|138|557|415|163|401|255|135|473|253|172|419|164|171 | 4.91E-44 |  | GO.0010941 | 4.093181414 | 0 |
| 272 | 67 | GO Function | | cytokine receptor binding | 1.40E-40 | CX3CL1|TGFBR3|TGFB1|CCL1|CCL13|CXCL6|TGFB3|TNFSF10|TNFSF9|TRAF2|LIF|PXDN|IL36G|IL36B|CCL21|TRAF5|SMAD7|SMAD2|TRAF4|SMURF2|IL1A|IL1B|IL7|IL11|STAT3|IL9|TNFSF4|SMAD6|CXCL3|CXCL5|DEFB1|IL12A|DEFB4A|IL13|CXCL11|CFLAR|CSF1|IL25|SMAD3|CXCL14|IL17F|CCRL2|CCL20|TGFBR2|CCL28|CXCL9|TGFB2|IL20|TGFBR1|TNFSF13B|CCL7|IL6ST|JAK2|IL36RN|CXCL12|TNFSF11|IL6|IRAK4|IL10|DEFB4B|TRAF6|IL22|TRAF3|CCL5|CCL16|SOCS2|CCL4 | 77 | 325|447|350|484|485|531|265|202|120|444|432|543|561|558|500|142|195|200|194|362|322|345|266|126|334|295|383|369|356|355|112|519|158|418|321|133|149|478|241|468|367|134|308|332|488|283|376|161|368|326|289|391|309|536|181|430|218|371|425|166|315|534|557|135|501|419|118 | 2.18E-43 |  | GO.0005126 | 3.985387196 | 0 |
| 4153 | 231 | GO Process |  | response to chemical | 2.98E-40 | CX3CL1|RELT|TNFRSF1A|TGFBR3|MAPK1|TGFB1|CCL1|CCL13|CXCL6|NFKB1|CDKN1B|LOX|IL1RL1|SOGA1|TGFB3|RHOQ|TNFSF10|TNFSF9|CCR7|TRAF2|LIF|PIAS1|TXNDC17|PXDN|IRAK2|PDGFRA|HILPDA|IL36G|IL36B|CCL21|FDX1|PDGFRB|MAPK6|LOXL1|FAF2|SMAD7|SMAD2|SMURF2|MAP3K8|NOX4|IL1A|IL1B|IL7|MAP4K3|ATF2|IL11|STAT3|PRDX5|LAMP3|IRF8|SOD1|IL22RA1|ILDR2|ACKR3|IL9|TNFRSF10B|PDCD4|TNFSF4|CSF1R|AIFM1|BAG4|IL17RB|SMAD6|TNFRSF13C|CXCR5|CXCL3|CXCL5|SNIP1|IL17RD|TNFRSF21|CREBRF|IL22RA2|TNFRSF11B|DEFB1|HIF1AN|AVPR1A|MAP2K1|BCL2L1|IL16|IL12A|DEFB4A|IL13|SOCS5|ALCAM|IL7R|CXCL11|PPARD|CFLAR|IL1RAP|TNIP1|PRKRA|TNFRSF12A|CSF1|IFNLR1|TGIF1|IL25|SOCS1|SOCS3|CCR4|SMAD3|CXCL14|IL17F|IL21R|SMAD4|LAMTOR4|GPR29|IFNAR2|MAP2K3|RBPJ|ATG7|BCL11B|IRF5|CCRL2|CCR9|CCL20|TGFBR2|MAP3K5|CCL28|GPX6|SMURF1|CXCL9|MAP3K3|TGFB2|IRF6|IL20|MAPKAPK2|TAB2|IL6R|MCL1|ATG5|MAP3K7|IRAK1|TNFRSF6B|BCL10|IFIT3|IFIT2|IL1RAPL2|AGO3|AGO1|AGO4|CXCR3|TLR4|IL2RG|TXNDC8|HLA-DQB1|TGFBR1|TNFSF13B|TNFRSF1B|HIF3A|CCL7|TAB3|BNIP3L|NFIB|IRF4|IL6ST|IL33|JAK2|TNFRSF19|TXNRD3|IL17RE|LOXL2|LYST|IL17REL|TANK|PDCD10|IL36RN|BCL2L11|SOCS4|MAPK8|CXCL12|NFATC2|IRF9|SOCS6|MAP2K7|BCL2|TNFSF11|MAPK4|TXNRD2|CMKBR6|CSF2RB|GATSL3|BCL6|PPARA|IL6|IL1R1|CXCR4|IL18R1|NFATC4|TLR6|IRAK4|MAPK9|CXCR6|HLA-B|NCOA2|DOCK4|OXR1|MAP2K4|IL10|TICAM2|ACKR2|DEFB4B|SMAD1|IKBKB|TRAF6|BCLAF1|DICER1|HIF1A|SMAD5|IL22|TET2|SOD2|APAF1|JKAMP|TRAF3|LITAF|TXNIP|IKBKE|TNFRSF11A|IER2|PIAS2|MAP2K6|CCL5|NOS1|CCL16|TNFRSF9|ECSCR|SOCS2|TNFAIP3|SOCS7|CCL4|MAP3K14 | 77 | 325|532|351|447|469|350|484|485|531|529|285|168|336|317|265|552|202|120|243|444|432|554|140|543|258|516|462|561|558|500|353|506|235|498|458|195|200|362|165|364|322|345|266|141|189|126|334|208|377|398|281|211|237|247|295|410|311|383|153|482|409|497|369|224|306|356|355|337|136|513|296|272|359|112|228|222|292|372|385|519|158|418|226|286|176|321|227|133|199|538|196|340|149|341|143|478|460|246|502|241|468|367|217|470|188|448|452|528|438|119|128|160|134|456|308|332|335|488|388|464|283|299|376|487|161|268|184|348|278|312|358|434|481|323|404|394|252|520|239|240|230|342|563|238|109|368|326|276|510|289|206|499|170|169|391|346|309|256|263|465|480|555|479|314|316|536|234|523|282|181|115|492|244|236|475|430|231|476|431|524|288|310|491|218|274|450|287|249|305|371|250|435|114|455|544|144|517|425|329|173|166|193|526|315|180|121|495|201|534|294|381|148|382|557|395|415|548|163|354|284|255|135|508|501|473|253|419|164|186|118|171 | 1.31E-42 |  | GO.0042221 | 3.952578374 | 0 |
| 1493 | 135 | GO Process |  | positive regulation of signal transduction | 7.01E-40 | CX3CL1|MAP4K5|TNFRSF1A|TGFBR3|MAPK1|TGFB1|CCL1|CCL13|NFKB1|TGFB3|TNFSF10|CCR7|TRAF2|LIF|MAP3K10|IRAK2|PDGFRA|CCL21|TRAF5|PDGFRB|WDR59|SMAD2|RIMS2|TRAF4|SMURF2|MAP3K8|NOX4|IL1B|IL7|MAP4K3|IL11|STAT3|MAP3K13|SOD1|ACKR3|TNFRSF10B|PDCD4|CSF1R|BAG4|WDR24|TGFA|TRAT1|RICTOR|CREBRF|MAP2K1|BCL2L1|IL12A|IL13|IL7R|CXCL11|MAP3K11|PPARD|CFLAR|PRKRA|ICOS|C8orf4|TNFRSF12A|CSF1|TNFAIP8L3|SOCS3|SMAD3|MIOS|SMAD4|LAMTOR4|MAP4K4|MAP2K3|RBPJ|MAP3K15|CCL20|MAP3K5|MAPK10|CXCL9|MAP3K3|NOS1AP|KIAA1804|TGFB2|IL20|MAPKAPK2|TAB2|IL6R|MCL1|MAP3K7|IRAK1|BCL10|YWHAB|AGO3|AGO1|CXCR3|TLR4|TGFBR1|ZAK|CCL7|TAB3|IL6ST|JAK2|TNFRSF19|MAP3K4|PDCD10|BCL2L11|MAPK8|MAP2K7|BCL2|TNFSF11|MAP3K1|IL6|IL1R1|CXCR4|IL18R1|MAP3K2|NFATC4|TLR6|IRAK4|MAPK9|TNIK|MAP2K4|TICAM2|CDKN2AIP|IKBKB|TRAF6|BCLAF1|HIF1A|APAF1|MAP3K12|MAPKAPK5|MAP3K9|MOAP1|IKBKE|TNFRSF11A|MAP2K6|CCL5|NOS1|CCL16|SOCS2|CCL4|MAP3K14 | 77 | 325|451|351|447|469|350|484|485|529|265|202|243|444|432|489|258|516|500|142|506|127|200|331|194|362|165|364|345|266|141|126|334|490|281|247|410|311|153|409|260|279|527|302|296|292|372|519|418|176|321|439|227|133|196|167|293|340|149|248|246|241|525|470|188|254|528|438|384|308|335|380|283|299|212|190|376|161|268|184|348|278|358|434|323|440|520|239|230|342|368|264|289|206|391|309|256|466|316|234|282|236|475|430|397|218|274|450|287|374|249|305|371|250|535|517|329|324|526|315|180|495|148|426|370|290|138|548|163|255|135|508|501|419|118|171 | 3.21E-42 |  | GO.0009967 | 3.915428198 | 0 |
| 2370 | 170 | GO Process |  | immune system process | 1.32E-39 | CX3CL1|RELT|TNFRSF1A|TGFBR3|MAPK1|TGFB1|CCL1|CCL13|TNFAIP1|CXCL6|NFKB1|CRTAM|IL1RL1|TNFSF10|TNFSF9|CCR7|LIF|LILRA1|PXDN|IRAK2|PDGFRA|DOCK10|IL36G|IL36B|CCL21|ARG2|FAF2|MAP3K8|IL1A|IL1B|IL7|IL11|STAT3|LAMP3|IGSF6|IRF8|SOD1|IL9|DOCK11|TNFRSF10B|DOCK1|TNFSF4|CSF1R|SMAD6|TNFRSF13C|CXCR5|TRAT1|CXCL3|CXCL5|TNFRSF21|TNFRSF11B|DEFB1|MAP2K1|MAEA|IL16|IL12A|DEFB4A|CTLA4|IL13|ALCAM|IL7R|CXCL11|IL1RAP|TNIP1|PRKRA|ICOS|C8orf4|CSF1|IFNLR1|IL25|IKZF1|CCR4|SMAD3|LAMP1|BTLA|PDCD1|CXCL14|IL21R|GPR29|IFNAR2|IKZF3|RBPJ|ATG7|BCL11B|IRF5|CCR9|CCL20|TGFBR2|MAP3K5|CPLX2|CCL28|CXCL9|ILF2|TGFB2|IRF6|MAPKAPK2|TAB2|THEMIS|IL6R|ATG5|MAP3K7|IRAK1BP1|IRAK1|TNFRSF6B|BCL10|PI4K2A|IFI44L|IFIT3|IFIT2|CXCR3|TLR4|IL2RG|HLA-DQB1|TGFBR1|TNFSF13B|TNFRSF1B|CCL7|TAB3|TXNDC5|BNIP3L|IRF4|IL33|JAK2|LYST|TANK|IL36RN|BCL2L11|CIAPIN1|CXCL12|NFATC2|IRF9|PDCD1LG2|BCL2|TNFSF11|MAP3K1|CMKBR6|BCL6|IL6|IL1R1|CXCR4|IL18R1|TLR6|IRAK4|ITK|HLA-DMB|HLA-B|ILF3|LAMP2|IL10|TICAM2|ACKR2|LAT2|DEFB4B|IKBKB|TRAF6|HIF1A|SMAD5|TET2|APAF1|TRAF3|IKBKE|TNFRSF11A|MAP2K6|CCL5|MR1|CCL16|TNFRSF9|TNFAIP3|CCL4|MAP3K14 | 77 | 325|532|351|447|469|350|484|485|280|531|529|151|336|202|120|243|432|344|543|258|516|505|561|558|500|518|458|165|322|345|266|126|334|377|546|398|281|295|422|410|215|383|153|369|224|306|527|356|355|513|359|112|292|207|385|519|158|183|418|286|176|321|199|538|196|167|293|149|341|478|220|502|241|360|449|537|468|217|448|452|392|438|119|128|160|456|308|332|335|216|488|283|443|376|487|268|184|349|348|312|358|400|434|481|323|399|177|404|394|230|342|563|109|368|326|276|289|206|459|499|169|346|309|555|314|536|234|472|181|115|492|373|475|430|397|431|310|218|274|450|287|305|371|556|553|114|110|304|425|329|173|467|166|526|315|495|201|294|148|557|548|163|255|135|540|501|473|164|118|171 | 6.30E-42 |  | GO.0002376 | 3.887942607 | 0 |
| 1764 | 145 | GO Process |  | regulation of intracellular signal transduction | 7.24E-39 | CX3CL1|MAP4K5|TNFRSF1A|MAPK1|TGFB1|CCL1|CCL13|TNFAIP1|IL1RL1|TGFB3|RHOQ|TNFSF10|CCR7|TRAF2|LIF|BCL2L2|MAP3K10|IRAK2|PDGFRA|CCL21|IER3|BCL2L10|TRAF5|PDGFRB|WDR59|TRAF4|MAP3K8|NOX4|IL1B|MAP4K3|IL11|STAT3|MAP3K13|SOD1|ACKR3|TNFRSF10B|PDCD4|DEPTOR|CSF1R|BAG4|WDR24|TGFA|TRAT1|RICTOR|IL22RA2|MAP2K1|BCL2L1|IL12A|IL13|SOCS5|IL7R|CXCL11|MAP3K11|PPARD|CFLAR|RHOJ|TNIP1|PRKRA|ICOS|C8orf4|CSF1|TNFAIP8L3|SOCS1|SOCS3|BAG5|MIOS|SMAD4|LAMTOR4|MAP4K4|MAP2K3|MAP3K15|CCL20|MAP3K5|MAPK10|CXCL9|MAP3K3|NOS1AP|KIAA1804|TGFB2|TAGAP|IL20|MAPKAPK2|TAB2|IL6R|MCL1|MAP3K7|IRAK1|BCL10|AGO3|AGO1|CXCR3|TLR4|TGFBR1|ZAK|CCL7|TAB3|IL6ST|JAK2|TNFRSF19|MAP3K4|TANK|PDCD10|BCL2L11|SOCS4|CXCL12|SOCS6|MAP2K7|BCL2|TNFSF11|MAP3K1|GATSL3|BCL6|IL6|CXCR4|IL18R1|MAP3K2|TLR6|IRAK4|MAPKBP1|TNIK|MAP2K4|PEX5L|IKBKB|TRAF6|BCLAF1|HIF1A|TNFAIP8L1|SOD2|MAP3K12|MAPKAPK5|MAP3K9|NFAT5|LITAF|IKBKE|TNFRSF11A|MAP2K6|CCL5|NOS1|CCL16|SOCS2|TNFAIP3|SOCS7|CCL4|MAP3K14|GATSL2 | 77 | 325|451|351|469|350|484|485|280|336|265|552|202|243|444|432|551|489|258|516|500|461|550|142|506|127|194|165|364|345|141|126|334|490|281|247|410|311|408|153|409|260|279|527|302|272|292|372|519|418|226|176|321|439|227|133|375|538|196|167|293|149|248|460|246|124|525|470|188|254|528|384|308|335|380|283|299|212|190|376|139|161|268|184|348|278|358|434|323|520|239|230|342|368|264|289|206|391|309|256|466|314|316|234|523|181|244|236|475|430|397|288|310|218|450|287|374|305|371|298|535|517|328|526|315|180|495|453|381|426|370|290|393|395|548|163|255|135|508|501|419|164|186|118|171|402 | 3.60E-41 |  | GO.1902531 | 3.814026143 | 0 |
| 3327 | 202 | GO Process |  | regulation of cell communication | 1.00E-38 | CX3CL1|MAP4K5|TNFRSF1A|TGFBR3|MAPK1|TGFB1|CCL1|CCL13|TNFAIP1|CXCL6|NFKB1|IL1RL1|TGFB3|RHOQ|TNFSF10|TNFSF9|CCR7|TRAF2|LIF|PIAS1|BCL2L2|PXDN|MAP3K10|IRAK2|PDGFRA|IL36G|IL36B|CCL21|IER3|BCL2L10|TRAF5|PDGFRB|WDR59|SMAD7|SMAD2|RIMS2|TRAF4|SMURF2|MAP3K8|NOX4|IL1A|IL1B|IL7|MAP4K3|IL11|STAT3|MAP3K13|SOD1|ACKR3|IL9|TNFRSF10B|PDCD4|TNFSF4|DEPTOR|CSF1R|BAG4|SMAD6|WDR24|TGFA|TRAT1|CXCL3|CXCL5|RICTOR|CREBRF|IL22RA2|TNFRSF11B|HIF1AN|AVPR1A|MAP2K1|BCL2L1|IL16|IL12A|IL13|SOCS5|IL7R|CXCL11|MAP3K11|PPARD|CFLAR|RHOJ|TNIP1|PRKRA|ICOS|C8orf4|TNFRSF12A|CSF1|TNFAIP8L3|PAWR|IL25|SOCS1|SOCS3|SMAD3|BCL9L|CXCL14|IL17F|BAG5|MIOS|SMAD4|LAMTOR4|MAP4K4|IFNAR2|MAP2K3|RBPJ|MAP3K15|CCL20|TGFBR2|MAP3K5|MAPK10|CPLX2|CCL28|SMURF1|CXCL9|MAP3K3|NOS1AP|KIAA1804|TGFB2|TAGAP|IL20|MAPKAPK2|TAB2|IL6R|MCL1|MAP3K7|IRAK1|BCL10|IGSF1|YWHAB|AGO3|AGO1|TRAF3IP1|CXCR3|TLR4|TGFBR1|ZAK|TNFSF13B|CCL7|TAB3|IRF4|IL6ST|IL33|JAK2|TNFRSF19|MAP3K4|TANK|PDCD10|IL36RN|BCL2L11|SOCS4|MAPK8|CXCL12|SOCS6|MAP2K7|BCL2|TNFSF11|MAP3K1|GATSL3|BCL6|IL6|LTBP1|IL1R1|CXCR4|IL18R1|MAP3K2|NFATC4|TLR6|IRAK4|MAPKBP1|MAPK9|OSGIN2|TNIK|MAP2K4|IL10|TICAM2|PEX5L|CDKN2AIP|IKBKB|TRAF6|BCLAF1|HIF1A|IL22|TNFAIP8L1|SOD2|APAF1|MAP3K12|MAPKAPK5|MAP3K9|MOAP1|NFAT5|LITAF|IKBKE|TNFRSF11A|PIAS2|MAP2K6|CCL5|NOS1|CCL16|SOCS2|TNFAIP3|SOCS7|CCL4|MAP3K14|GATSL2 | 77 | 325|451|351|447|469|350|484|485|280|531|529|336|265|552|202|120|243|444|432|554|551|543|489|258|516|561|558|500|461|550|142|506|127|195|200|331|194|362|165|364|322|345|266|141|126|334|490|281|247|295|410|311|383|408|153|409|369|260|279|527|356|355|302|296|272|359|228|222|292|372|385|519|418|226|176|321|439|227|133|375|538|196|167|293|340|149|248|396|478|460|246|241|125|468|367|124|525|470|188|254|452|528|438|384|308|332|335|380|216|488|464|283|299|212|190|376|139|161|268|184|348|278|358|434|323|132|440|520|239|474|230|342|368|264|326|289|206|169|391|346|309|256|466|314|316|536|234|523|282|181|244|236|475|430|397|288|310|218|154|274|450|287|374|249|305|371|298|250|446|535|517|425|329|328|324|526|315|180|495|534|453|381|148|426|370|290|138|393|395|548|163|284|255|135|508|501|419|164|186|118|171|402 | 5.18E-41 |  | GO.0010646 | 3.8 | 0 |
| 3360 | 202 | GO Process |  | regulation of signaling | 4.04E-38 | CX3CL1|MAP4K5|TNFRSF1A|TGFBR3|MAPK1|TGFB1|CCL1|CCL13|TNFAIP1|CXCL6|NFKB1|IL1RL1|TGFB3|RHOQ|TNFSF10|TNFSF9|CCR7|TRAF2|LIF|PIAS1|BCL2L2|PXDN|MAP3K10|IRAK2|PDGFRA|IL36G|IL36B|CCL21|IER3|BCL2L10|TRAF5|PDGFRB|WDR59|SMAD7|SMAD2|RIMS2|TRAF4|SMURF2|MAP3K8|NOX4|IL1A|IL1B|IL7|MAP4K3|IL11|STAT3|MAP3K13|SOD1|ACKR3|IL9|TNFRSF10B|PDCD4|TNFSF4|DEPTOR|CSF1R|BAG4|SMAD6|WDR24|TGFA|TRAT1|CXCL3|CXCL5|RICTOR|CREBRF|IL22RA2|TNFRSF11B|HIF1AN|AVPR1A|MAP2K1|BCL2L1|IL16|IL12A|IL13|SOCS5|IL7R|CXCL11|MAP3K11|PPARD|CFLAR|RHOJ|TNIP1|PRKRA|ICOS|C8orf4|TNFRSF12A|CSF1|TNFAIP8L3|PAWR|IL25|SOCS1|SOCS3|SMAD3|BCL9L|CXCL14|IL17F|BAG5|MIOS|SMAD4|LAMTOR4|MAP4K4|IFNAR2|MAP2K3|RBPJ|MAP3K15|CCL20|TGFBR2|MAP3K5|MAPK10|CPLX2|CCL28|SMURF1|CXCL9|MAP3K3|NOS1AP|KIAA1804|TGFB2|TAGAP|IL20|MAPKAPK2|TAB2|IL6R|MCL1|MAP3K7|IRAK1|BCL10|IGSF1|YWHAB|AGO3|AGO1|TRAF3IP1|CXCR3|TLR4|TGFBR1|ZAK|TNFSF13B|CCL7|TAB3|IRF4|IL6ST|IL33|JAK2|TNFRSF19|MAP3K4|TANK|PDCD10|IL36RN|BCL2L11|SOCS4|MAPK8|CXCL12|SOCS6|MAP2K7|BCL2|TNFSF11|MAP3K1|GATSL3|BCL6|IL6|LTBP1|IL1R1|CXCR4|IL18R1|MAP3K2|NFATC4|TLR6|IRAK4|MAPKBP1|MAPK9|OSGIN2|TNIK|MAP2K4|IL10|TICAM2|PEX5L|CDKN2AIP|IKBKB|TRAF6|BCLAF1|HIF1A|IL22|TNFAIP8L1|SOD2|APAF1|MAP3K12|MAPKAPK5|MAP3K9|MOAP1|NFAT5|LITAF|IKBKE|TNFRSF11A|PIAS2|MAP2K6|CCL5|NOS1|CCL16|SOCS2|TNFAIP3|SOCS7|CCL4|MAP3K14|GATSL2 | 77 | 325|451|351|447|469|350|484|485|280|531|529|336|265|552|202|120|243|444|432|554|551|543|489|258|516|561|558|500|461|550|142|506|127|195|200|331|194|362|165|364|322|345|266|141|126|334|490|281|247|295|410|311|383|408|153|409|369|260|279|527|356|355|302|296|272|359|228|222|292|372|385|519|418|226|176|321|439|227|133|375|538|196|167|293|340|149|248|396|478|460|246|241|125|468|367|124|525|470|188|254|452|528|438|384|308|332|335|380|216|488|464|283|299|212|190|376|139|161|268|184|348|278|358|434|323|132|440|520|239|474|230|342|368|264|326|289|206|169|391|346|309|256|466|314|316|536|234|523|282|181|244|236|475|430|397|288|310|218|154|274|450|287|374|249|305|371|298|250|446|535|517|425|329|328|324|526|315|180|495|534|453|381|148|426|370|290|138|393|395|548|163|284|255|135|508|501|419|164|186|118|171|402 | 2.16E-40 |  | GO.0023051 | 3.739361863 | 0 |
| 10484 | 379 | GO Process |  | regulation of cellular process | 9.75E-38 | CX3CL1|MAP4K5|RELT|TNFRSF1A|TGFBR3|MAPK1|TGFB1|CCL1|CCL13|TNFAIP1|CXCL6|NFKB1|CRTAM|CDKN1B|IL1RL1|BCL9|SOGA1|TGFB3|RHOQ|TNFSF10|RASL11A|TNFSF9|TMX4|CCR7|ATG14|TRAF2|LIF|PIAS1|TXNDC17|BCL2L2|DOCK7|LILRA1|PXDN|MAP3K10|EXOC4|IRAK2|PDGFRA|HILPDA|DOCK10|TBRG4|IL36G|IL36B|CCL21|IER3|BCL2L10|TRAF5|ARG2|PDGFRB|MAPK6|MMD|MSR1|WDR59|SMAD7|SMAD2|RIMS2|TRAF4|SMURF2|MAP3K8|NOX4|IL1A|IL1B|IL7|MAP4K3|ATF2|IL11|STAT3|MAP3K13|PRDX5|LAMP3|CDK6|CCAR1|DOCK3|BNIP2|IGSF6|IRF8|SOD1|IL22RA1|ACKR3|IL9|DOCK11|TNFRSF10B|DOCK5|PDCD4|DOCK1|TNFSF4|ATG10|TXNDC11|ATG3|DEPTOR|CSF1R|AIFM1|BAG4|GATAD1|IL17RB|SMAD6|TNFRSF13C|CXCR5|WDR24|TGFA|TRAT1|CXCL3|CXCL5|SNIP1|IL17RD|RICTOR|TNFRSF21|CREBRF|IL22RA2|TNFRSF11B|DEFB1|HIF1AN|AVPR1A|TMX3|NFATC3|LTBP3|MAP2K1|BCL2L1|MAEA|IL16|IL12A|DEFB4A|NKIRAS2|CTLA4|IL13|SOCS5|ALCAM|IL7R|AVEN|CXCL11|MAP3K11|PPARD|OXSR1|CFLAR|IL1RAP|RHOJ|TNIP1|PRKRA|ICOS|C8orf4|NFATC2IP|TNFRSF12A|CSF1|IFNLR1|TGIF1|TNFAIP8L3|PAWR|IL25|SOCS1|PIWIL3|SOCS3|IKZF1|AEN|CCR4|SMAD3|LAMP1|BTLA|PDCD1|BCL9L|CXCL14|IL17F|IL21R|BCL11A|BAG5|KIR2DL4|MIOS|SMAD4|LAMTOR4|MAP4K4|GPR29|IFNAR2|IKZF3|MAP2K3|RBPJ|MAP3K15|ATG7|AREL1|PEX2|BCL11B|IRF5|CCRL2|FXR1|CCR9|TXNDC15|CCL20|TGFBR2|MAP3K5|MAPK10|CPLX2|CCL28|SMURF1|CXCL9|MAP3K3|ILF2|NOS1AP|IRF2BP2|KIAA1804|TGFB2|IRF6|TAGAP|IL20|MAPKAPK2|TAB2|HIVEP2|THEMIS|IL6R|GATAD2B|IKZF5|MCL1|ATG5|BAG3|CACUL1|PEX11B|MAP3K7|MTCP1|IGSF3|IRAK1BP1|IRAK1|TNFRSF6B|BCL10|BAG2|EXOSC1|IGSF1|LCOR|IFIT3|IFIT2|IL1RAPL2|YWHAB|AGO3|AGO1|AGO4|TRAF3IP1|CXCR3|TGIF2|TLR4|IL2RG|TXNDC8|HLA-DQB1|TGFBR1|ZAK|MAP3K19|TNFSF13B|TNFRSF1B|DOCK9|HIF3A|PLCXD3|BCOR|CCL7|TAB3|TXNDC5|CAAP1|BNIP3L|NFIB|IRF4|IL6ST|IL33|JAK2|TNFRSF19|TXNRD3|IL17RE|LOXL2|EXOSC8|IL17REL|MAP3K4|TANK|PDCD10|IL36RN|BCL2L11|BCL2L15|PRC1|CIAPIN1|AKTIP|SOCS4|ULK2|MAPK8|CXCL12|NFATC2|IRF9|BLOC1S5|PDCD1LG2|SOCS6|MAP2K7|BCL2|TNFSF11|MAP3K1|MAPK4|TXNRD2|CMKBR6|CSF2RB|GATSL3|BCL6|RASSF8|PPARA|IL6|LTBP1|IL1R1|CXCR4|IL18R1|MAP3K2|NFATC4|TLR6|IRAK4|PEX5|MAPKBP1|TMX1|EIF4E3|NKIRAS1|MAPK9|CXCR6|OSGIN2|ITK|HLA-DMB|HLA-B|TNIK|NCOA2|ILF3|DOCK4|OXR1|LAMP2|TBRG1|MAP2K4|BCL7B|PDCD6IP|IL10|IKZF2|TICAM2|TGFBI|ACKR2|PXT1|PEX5L|LAT2|DEFB4B|SMAD1|CDKN2AIP|TNFAIP8|IKBKB|API5|AMBRA1|ATG13|TRAF6|BCLAF1|DICER1|BCORL1|HIF1A|SMAD5|NKRF|IL22|TET2|RASSF3|TNFAIP8L1|BCL7A|SOD2|APAF1|MAP3K12|MAPKAPK5|MAP3K9|MOAP1|TRAF3|NFAT5|LITAF|TXNIP|IKBKE|TNFRSF11A|IER2|PIAS2|ERC1|MAP2K6|CCL5|MR1|NOS1|CCL16|TNFRSF9|ECSCR|PANO|SOCS2|TNFAIP3|SOCS7|CCL4|MAP3K14|GATSL2 | 77 | 325|451|532|351|447|469|350|484|485|280|531|529|151|285|336|175|317|265|552|202|205|120|301|243|150|444|432|554|140|551|413|344|543|489|159|258|516|462|505|496|561|558|500|461|550|142|518|506|235|427|147|127|195|200|331|194|362|165|364|322|345|266|141|189|126|334|490|208|377|365|111|225|187|546|398|281|211|247|295|422|410|406|311|215|383|155|156|113|408|153|482|409|530|497|369|224|306|260|279|527|356|355|337|136|302|513|296|272|359|112|228|222|122|210|441|292|372|207|385|519|158|273|183|418|226|286|176|145|321|439|227|504|133|199|375|538|196|167|293|347|340|149|341|143|248|396|478|460|182|246|220|547|502|241|360|449|537|125|468|367|217|507|124|515|525|470|188|254|448|452|392|528|438|384|119|437|269|128|160|134|245|456|494|308|332|335|380|216|488|464|283|299|443|212|178|190|376|487|139|161|268|184|433|349|348|414|412|278|312|318|522|343|358|162|270|400|434|481|323|116|137|132|233|404|394|252|440|520|239|240|474|230|259|342|563|238|109|368|264|352|326|276|300|510|407|541|289|206|459|428|499|170|169|391|346|309|256|263|465|480|297|479|466|314|316|536|234|320|511|472|424|523|214|282|181|115|492|198|373|244|236|475|430|397|231|476|431|524|288|310|549|491|218|154|274|450|287|374|249|305|371|514|298|390|403|417|250|435|446|556|553|114|535|455|110|544|144|304|445|517|174|307|425|486|329|157|173|363|328|467|166|193|324|185|526|463|131|559|315|180|121|387|495|201|291|534|294|221|453|303|381|148|426|370|290|138|557|393|395|415|548|163|354|284|401|255|135|540|508|501|473|253|172|419|164|186|118|171|402 | 5.40E-40 |  | GO.0050794 | 3.701099538 | 0 |
| 1560 | 134 | GO Process |  | immune response | 2.33E-37 | CX3CL1|RELT|TNFRSF1A|TGFBR3|MAPK1|TGFB1|CCL1|CCL13|TNFAIP1|CXCL6|NFKB1|CRTAM|IL1RL1|TNFSF10|TNFSF9|CCR7|LIF|LILRA1|PXDN|DOCK10|IL36G|IL36B|CCL21|ARG2|FAF2|IL1A|IL1B|IL7|STAT3|LAMP3|IGSF6|IRF8|IL9|DOCK11|TNFRSF10B|TNFSF4|CSF1R|SMAD6|TNFRSF13C|CXCR5|TRAT1|CXCL3|CXCL5|TNFRSF21|TNFRSF11B|DEFB1|IL16|IL12A|DEFB4A|CTLA4|IL13|ALCAM|IL7R|CXCL11|IL1RAP|PRKRA|ICOS|C8orf4|CSF1|IFNLR1|IL25|CCR4|SMAD3|LAMP1|BTLA|PDCD1|CXCL14|GPR29|IFNAR2|RBPJ|ATG7|IRF5|CCR9|CCL20|MAP3K5|CPLX2|CCL28|CXCL9|ILF2|IRF6|THEMIS|IL6R|IRAK1BP1|IRAK1|TNFRSF6B|BCL10|PI4K2A|IFIT3|IFIT2|TLR4|IL2RG|HLA-DQB1|TNFSF13B|TNFRSF1B|CCL7|TXNDC5|IRF4|IL33|JAK2|LYST|IL36RN|CXCL12|IRF9|PDCD1LG2|BCL2|TNFSF11|CMKBR6|BCL6|IL6|IL1R1|IL18R1|TLR6|IRAK4|ITK|HLA-DMB|HLA-B|LAMP2|IL10|TICAM2|ACKR2|LAT2|DEFB4B|IKBKB|TRAF6|APAF1|TRAF3|IKBKE|TNFRSF11A|CCL5|MR1|CCL16|TNFRSF9|CCL4|MAP3K14 | 77 | 325|532|351|447|469|350|484|485|280|531|529|151|336|202|120|243|432|344|543|505|561|558|500|518|458|322|345|266|334|377|546|398|295|422|410|383|153|369|224|306|527|356|355|513|359|112|385|519|158|183|418|286|176|321|199|196|167|293|149|341|478|502|241|360|449|537|468|448|452|438|119|160|456|308|335|216|488|283|443|487|349|348|400|434|481|323|399|404|394|342|563|109|326|276|289|459|169|346|309|555|536|181|492|373|475|430|431|310|218|274|287|305|371|556|553|114|304|425|329|173|467|166|526|315|148|557|548|163|135|540|501|473|118|171 | 1.33E-39 |  | GO.0006955 | 3.663264408 | 0 |
| 1925 | 147 | Reactome Pathways | | Immune System | 5.90E-36 | TNFRSF1A|MAPK1|TGFB1|NFKB1|CRTAM|IL1RL1|TNFSF9|TRAF2|LIF|PIAS1|LILRA1|IRAK2|IL36G|IL36B|FAF2|SMURF2|MAP3K8|IL1A|IL1B|IL7|ATF2|IL11|STAT3|IRF8|SOD1|IL22RA1|IL9|PDCD4|DOCK1|TNFSF4|CSF1R|IL17RB|TNFRSF13C|TRAT1|RICTOR|IL22RA2|TNFRSF11B|DEFB1|NFATC3|MAP2K1|BCL2L1|IL16|IL12A|NKIRAS2|CTLA4|IL13|SOCS5|IL7R|EEA1|TNFRSF12A|CSF1|IFNLR1|IL25|SOCS1|SOCS3|SMAD3|LAMP1|BTLA|PDCD1|IL17F|IL21R|KIR2DL4|NCR3LG1|MAP2K3|ATG7|AREL1|IRF5|CCL20|MAPK10|SMURF1|MAP3K3|ILF2|IRF6|IL20|MAPKAPK2|TAB2|IL6R|MCL1|ATG5|MAP3K7|SIKE1|IRAK1|BCL10|IFIT3|IFIT2|YWHAB|TLR4|IL2RG|HLA-DQB1|TNFSF13B|TNFRSF1B|TAB3|TXNDC5|IRF4|IL6ST|IL33|JAK2|IL17RE|TANK|IL36RN|MAPK8|NFATC2|IRF9|PDCD1LG2|MAP2K7|BCL2|TNFSF11|MAP3K1|CMKBR6|CSF2RB|BCL6|IL6|IL1R1|IL18R1|TLR6|IRAK4|EIF4E3|NKIRAS1|MAPK9|ITK|HLA-DMB|HLA-B|LAMP2|MAP2K4|IL10|TICAM2|LAT2|DEFB4B|ATG12|IKBKB|TRAF6|HIF1A|IL22|SOD2|APAF1|TRAF3|TXNIP|IKBKE|TNFRSF11A|MAP2K6|CCL5|NOS1|TNFRSF9|SOCS2|TNFAIP3|CCL4|MAP3K14 | 77 | 351|469|350|529|151|336|120|444|432|554|344|258|561|558|458|362|165|322|345|266|189|126|334|398|281|211|295|311|215|383|153|497|224|527|302|272|359|112|210|292|372|385|519|273|183|418|226|176|146|340|149|341|478|460|246|241|360|449|537|367|217|515|313|528|119|437|160|308|380|464|299|443|487|161|268|184|348|278|312|358|338|434|323|404|394|440|342|563|109|326|276|206|459|169|391|346|309|465|314|536|282|115|492|373|236|475|430|397|431|524|310|218|274|287|305|371|403|417|250|556|553|114|304|517|425|329|467|166|421|526|315|495|534|381|148|557|415|548|163|255|135|508|473|419|164|118|171 | 2.63E-38 |  | HSA-168256 | 3.522914799 | 0 |
| 1528 | 128 | GO Process |  | intracellular signal transduction | 2.13E-34 | MAP4K5|TNFRSF1A|TGFBR3|MAPK1|TGFB1|NFKB1|CDKN1B|RHOQ|TRAF2|LIF|PIAS1|BCL2L2|DOCK7|MAP3K10|IRAK2|PDGFRA|DOCK10|BCL2L10|PDGFRB|MAPK6|SMAD2|RIMS2|TRAF4|MAP3K8|IL1B|MAP4K3|STAT3|MAP3K13|DOCK3|DOCK11|TNFRSF10B|DOCK5|DOCK1|DEPTOR|CSF1R|AIFM1|SNIP1|IL17RD|RICTOR|DEFB1|AVPR1A|MAP2K1|BCL2L1|NKIRAS2|SOCS5|MAP3K11|OXSR1|RHOJ|TNFAIP8L3|SOCS1|SOCS3|AEN|SMAD4|MAP4K4|GPR29|IFNAR2|MAP2K3|MAP3K15|CCL20|MAP3K5|MAPK10|MAP3K3|KIAA1804|MAPKAPK2|TAB2|MCL1|MAP3K7|IRAK1BP1|IRAK1|BCL10|YWHAB|CXCR3|TLR4|IL2RG|TGFBR1|ZAK|MAP3K19|TNFRSF1B|DOCK9|TAB3|JAK2|TNFRSF19|MAP3K4|TANK|PDCD10|BCL2L11|SOCS4|MAPK8|NFATC2|SOCS6|MAP2K7|BCL2|TNFSF11|MAP3K1|MAPK4|CMKBR6|CSF2RB|BCL6|CXCR4|MAP3K2|NFATC4|TLR6|IRAK4|NKIRAS1|MAPK9|ITK|TNIK|DOCK4|MAP2K4|TICAM2|LAT2|SMAD1|IKBKB|TRAF6|APAF1|MAP3K12|MAPKAPK5|MAP3K9|MOAP1|IKBKE|ERC1|MAP2K6|CCL5|NOS1|SOCS2|TNFAIP3|SOCS7|MAP3K14 | 77 | 451|351|447|469|350|529|285|552|444|432|554|551|413|489|258|516|505|550|506|235|200|331|194|165|345|141|334|490|225|422|410|406|215|408|153|482|337|136|302|112|222|292|372|273|226|439|504|375|248|460|246|547|470|254|448|452|528|384|308|335|380|299|190|268|184|278|358|400|434|323|440|230|342|563|368|264|352|276|300|206|309|256|466|314|316|234|523|282|115|244|236|475|430|397|231|431|524|310|450|374|249|305|371|417|250|556|535|544|517|329|467|193|526|315|148|426|370|290|138|548|401|255|135|508|419|164|186|171 | 1.34E-36 |  | GO.0035556 | 3.36716204 | 0 |
| 1234 | 114 | GO Process |  | defense response | 1.04E-33 | CX3CL1|RELT|TNFRSF1A|TGFB1|CCL1|CCL13|CXCL6|NFKB1|CCR7|LILRA1|IRAK2|IL36G|IL36B|CCL21|ARG2|NOX4|IL1A|IL1B|STAT3|PRDX5|IRF8|IL22RA1|IL9|TNFRSF10B|TNFSF4|CSF1R|IL17RB|TRAT1|CXCL3|CXCL5|TNFRSF21|TNFRSF11B|DEFB1|NFATC3|IL12A|DEFB4A|IL13|CXCL11|IL1RAP|TNIP1|CSF1|IFNLR1|IL25|CCR4|IL17F|KIR2DL4|GPR29|IFNAR2|MAP2K3|RBPJ|ATG7|IRF5|CCRL2|CCR9|CCL20|MAP3K5|CXCL9|IRF6|MAPKAPK2|IL6R|IRAK1|TNFRSF6B|BCL10|IFI44L|IFIT3|IFIT2|CXCR3|TLR4|HLA-DQB1|TNFRSF1B|CCL7|BNIP3L|IRF4|IL33|JAK2|IL17RE|LYST|ATG16L1|IL36RN|CXCL12|IRF9|SOCS6|BCL2|CMKBR6|BCL6|IL6|IL1R1|CXCR4|IL18R1|NFATC4|TLR6|IRAK4|CXCR6|ITK|HLA-B|ILF3|IL10|TICAM2|ACKR2|DEFB4B|SMAD1|IKBKB|HIF1A|IL22|TRAF3|IKBKE|TNFRSF11A|CCL5|MR1|CCL16|TNFRSF9|TNFAIP3|CCL4|MAP3K14 | 77 | 325|532|351|350|484|485|531|529|243|344|258|561|558|500|518|364|322|345|334|208|398|211|295|410|383|153|497|527|356|355|513|359|112|210|519|158|418|321|199|538|149|341|478|502|367|515|448|452|528|438|119|160|134|456|308|335|283|487|268|348|434|481|323|177|404|394|230|342|109|276|289|499|169|346|309|465|555|223|536|181|492|244|475|431|310|218|274|450|287|249|305|371|435|556|114|110|425|329|173|166|193|526|495|534|557|548|163|135|540|501|473|164|118|171 | 6.72E-36 |  | GO.0006952 | 3.298296666 | 0 |
| 11116 | 384 | GO Process |  | regulation of biological process | 1.25E-33 | CX3CL1|MAP4K5|RELT|TNFRSF1A|TGFBR3|MAPK1|TGFB1|CCL1|CCL13|TNFAIP1|CXCL6|NFKB1|CRTAM|CDKN1B|EXOC2|IL1RL1|BCL9|SOGA1|TGFB3|RHOQ|TNFSF10|RASL11A|TNFSF9|TMX4|CCR7|ATG14|TRAF2|LIF|PIAS1|TXNDC17|BCL2L2|DOCK7|LILRA1|PXDN|MAP3K10|EXOC4|XPO4|IRAK2|PDGFRA|HILPDA|DOCK10|TBRG4|IL36G|IL36B|CCL21|IER3|BCL2L10|TRAF5|ARG2|PDGFRB|MAPK6|MMD|MSR1|WDR59|SMAD7|SMAD2|RIMS2|TRAF4|SMURF2|MAP3K8|NOX4|IL1A|IL1B|IL7|MAP4K3|ATF2|IL11|STAT3|MAP3K13|PRDX5|LAMP3|CDK6|CCAR1|DOCK3|BNIP2|IGSF6|IRF8|SOD1|IL22RA1|ACKR3|IL9|DOCK11|TNFRSF10B|DOCK5|PDCD4|DOCK1|TNFSF4|ATG10|TXNDC11|ATG3|DEPTOR|CSF1R|AIFM1|BAG4|GATAD1|IL17RB|SMAD6|TNFRSF13C|CXCR5|WDR24|TGFA|FAM19A4|TRAT1|CXCL3|CXCL5|SNIP1|IL17RD|RICTOR|TNFRSF21|CREBRF|IL22RA2|TNFRSF11B|DEFB1|HIF1AN|AVPR1A|TMX3|NFATC3|LTBP3|MAP2K1|BCL2L1|MAEA|IL16|IL12A|DEFB4A|NKIRAS2|CTLA4|IL13|SOCS5|ALCAM|IL7R|AVEN|CXCL11|MAP3K11|PPARD|OXSR1|CFLAR|IL1RAP|RHOJ|TNIP1|PRKRA|ICOS|C8orf4|NFATC2IP|TNFRSF12A|CSF1|IFNLR1|TGIF1|TNFAIP8L3|PAWR|IL25|SOCS1|PIWIL3|SOCS3|IKZF1|AEN|CCR4|SMAD3|LAMP1|BTLA|PDCD1|BCL9L|CXCL14|IL17F|IL21R|BCL11A|BAG5|KIR2DL4|MIOS|SMAD4|NCR3LG1|LAMTOR4|MAP4K4|GPR29|IFNAR2|IKZF3|MAP2K3|RBPJ|MAP3K15|ATG7|AREL1|PEX2|BCL11B|IRF5|CCRL2|FXR1|CCR9|TXNDC15|CCL20|TGFBR2|MAP3K5|MAPK10|CPLX2|CCL28|SMURF1|CXCL9|MAP3K3|ILF2|NOS1AP|IRF2BP2|KIAA1804|TGFB2|IRF6|TAGAP|IL20|MAPKAPK2|TAB2|HIVEP2|THEMIS|IL6R|GATAD2B|IKZF5|MCL1|ATG5|BAG3|CACUL1|PEX11B|MAP3K7|MTCP1|IGSF3|IRAK1BP1|IRAK1|TNFRSF6B|BCL10|BAG2|EXOSC1|IGSF1|LCOR|IFIT3|IFIT2|IL1RAPL2|YWHAB|AGO3|AGO1|AGO4|TRAF3IP1|CXCR3|TGIF2|TLR4|IL2RG|TXNDC8|HLA-DQB1|TGFBR1|ZAK|MAP3K19|TNFSF13B|TNFRSF1B|DOCK9|HIF3A|PLCXD3|BCOR|CCL7|TAB3|TXNDC5|CAAP1|BNIP3L|NFIB|IRF4|IL6ST|IL33|JAK2|TNFRSF19|TXNRD3|IL17RE|LOXL2|EXOSC8|IL17REL|MAP3K4|TANK|PDCD10|IL36RN|BCL2L11|BCL2L15|IGSF11|PRC1|CIAPIN1|AKTIP|SOCS4|ULK2|MAPK8|CXCL12|NFATC2|IRF9|BLOC1S5|PDCD1LG2|SOCS6|MAP2K7|BCL2|TNFSF11|MAP3K1|MAPK4|TXNRD2|CMKBR6|CSF2RB|GATSL3|BCL6|RASSF8|PPARA|IL6|LTBP1|IL1R1|CXCR4|IL18R1|MAP3K2|NFATC4|TLR6|IRAK4|PEX5|MAPKBP1|TMX1|EIF4E3|NKIRAS1|MAPK9|CXCR6|OSGIN2|ITK|HLA-DMB|HLA-B|TNIK|NCOA2|ILF3|DOCK4|OXR1|LAMP2|TBRG1|MAP2K4|BCL7B|PDCD6IP|IL10|IKZF2|TICAM2|TGFBI|ACKR2|PXT1|PEX5L|LAT2|DEFB4B|SMAD1|CDKN2AIP|TNFAIP8|IKBKB|API5|AMBRA1|ATG13|TRAF6|BCLAF1|DICER1|BCORL1|HIF1A|SMAD5|NKRF|IL22|TET2|RASSF3|TNFAIP8L1|BCL7A|SOD2|APAF1|MAP3K12|MAPKAPK5|MAP3K9|MOAP1|TRAF3|NFAT5|LITAF|TXNIP|IKBKE|TNFRSF11A|IER2|PIAS2|ERC1|MAP2K6|CCL5|MR1|NOS1|CCL16|TNFRSF9|ECSCR|PANO|SOCS2|TNFAIP3|SOCS7|CCL4|MAP3K14|GATSL2 | 77 | 325|451|532|351|447|469|350|484|485|280|531|529|151|285|339|336|175|317|265|552|202|205|120|301|243|150|444|432|554|140|551|413|344|543|489|159|386|258|516|462|505|496|561|558|500|461|550|142|518|506|235|427|147|127|195|200|331|194|362|165|364|322|345|266|141|189|126|334|490|208|377|365|111|225|187|546|398|281|211|247|295|422|410|406|311|215|383|155|156|113|408|153|482|409|530|497|369|224|306|260|279|545|527|356|355|337|136|302|513|296|272|359|112|228|222|122|210|441|292|372|207|385|519|158|273|183|418|226|286|176|145|321|439|227|504|133|199|375|538|196|167|293|347|340|149|341|143|248|396|478|460|182|246|220|547|502|241|360|449|537|125|468|367|217|507|124|515|525|470|313|188|254|448|452|392|528|438|384|119|437|269|128|160|134|245|456|494|308|332|335|380|216|488|464|283|299|443|212|178|190|376|487|139|161|268|184|433|349|348|414|412|278|312|318|522|343|358|162|270|400|434|481|323|116|137|132|233|404|394|252|440|520|239|240|474|230|259|342|563|238|109|368|264|352|326|276|300|510|407|541|289|206|459|428|499|170|169|391|346|309|256|263|465|480|297|479|466|314|316|536|234|320|130|511|472|424|523|214|282|181|115|492|198|373|244|236|475|430|397|231|476|431|524|288|310|549|491|218|154|274|450|287|374|249|305|371|514|298|390|403|417|250|435|446|556|553|114|535|455|110|544|144|304|445|517|174|307|425|486|329|157|173|363|328|467|166|193|324|185|526|463|131|559|315|180|121|387|495|201|291|534|294|221|453|303|381|148|426|370|290|138|557|393|395|415|548|163|354|284|401|255|135|540|508|501|473|253|172|419|164|186|118|171|402 | 8.38E-36 |  | GO.0050789 | 3.290308999 | 0 |
| 1587 | 129 | GO Process |  | positive regulation of protein metabolic process | 1.71E-33 | CX3CL1|MAP4K5|TNFRSF1A|MAPK1|TGFB1|CCL1|CCL13|CDKN1B|TGFB3|TNFSF10|CCR7|ATG14|TRAF2|LIF|PIAS1|DOCK7|MAP3K10|IRAK2|PDGFRA|CCL21|BCL2L10|PDGFRB|MMD|SMAD7|TRAF4|MAP3K8|NOX4|IL1A|IL1B|MAP4K3|IL11|STAT3|MAP3K13|DOCK3|SOD1|ACKR3|IL9|TNFRSF10B|ATG10|CSF1R|AIFM1|BAG4|TNFRSF13C|TGFA|RICTOR|CREBRF|MAP2K1|IL12A|IL13|SOCS5|MAP3K11|OXSR1|CFLAR|TNIP1|C8orf4|CSF1|TNFAIP8L3|PAWR|SOCS3|SMAD3|SMAD4|MAP4K4|MAP2K3|MAP3K15|ATG7|CCL20|TGFBR2|MAP3K5|MAPK10|SMURF1|MAP3K3|NOS1AP|KIAA1804|TGFB2|IL20|MAPKAPK2|TAB2|IL6R|CACUL1|MAP3K7|MTCP1|IRAK1|BCL10|BAG2|TLR4|TGFBR1|ZAK|MAP3K19|TNFRSF1B|CCL7|TAB3|IRF4|IL6ST|IL33|JAK2|TNFRSF19|MAP3K4|TANK|PDCD10|BCL2L11|AKTIP|SOCS4|MAPK8|MAP2K7|BCL2|TNFSF11|MAP3K1|BCL6|IL6|CXCR4|MAP3K2|TLR6|MAPK9|TNIK|MAP2K4|TRAF6|APAF1|MAP3K12|MAPKAPK5|MAP3K9|TNFRSF11A|MAP2K6|CCL5|NOS1|CCL16|ECSCR|TNFAIP3|CCL4|MAP3K14 | 77 | 325|451|351|469|350|484|485|285|265|202|243|150|444|432|554|413|489|258|516|500|550|506|427|195|194|165|364|322|345|141|126|334|490|225|281|247|295|410|155|153|482|409|224|279|302|296|292|519|418|226|439|504|133|538|293|149|248|396|246|241|470|254|528|384|119|308|332|335|380|464|299|212|190|376|161|268|184|348|522|358|162|434|323|116|342|368|264|352|276|289|206|169|391|346|309|256|466|314|316|234|424|523|282|236|475|430|397|310|218|450|374|305|250|535|517|315|148|426|370|290|163|255|135|508|501|253|164|118|171 | 1.18E-35 |  | GO.0051247 | 3.276700389 | 0 |
| 482 | 73 | GO Process |  | inflammatory response | 1.19E-32 | CX3CL1|RELT|TNFRSF1A|TGFB1|CCL1|CCL13|CXCL6|NFKB1|CCR7|IRAK2|IL36G|IL36B|CCL21|NOX4|IL1A|IL1B|STAT3|PRDX5|IL9|TNFRSF10B|TNFSF4|CSF1R|CXCL3|CXCL5|TNFRSF21|TNFRSF11B|NFATC3|IL13|CXCL11|IL1RAP|TNIP1|CSF1|IL25|CCR4|IL17F|GPR29|MAP2K3|RBPJ|CCRL2|CCL20|CXCL9|MAPKAPK2|IL6R|TNFRSF6B|CXCR3|TLR4|TNFRSF1B|CCL7|IL33|IL17RE|IL36RN|CMKBR6|BCL6|IL6|IL1R1|CXCR4|IL18R1|NFATC4|TLR6|CXCR6|IL10|TICAM2|ACKR2|SMAD1|IKBKB|HIF1A|IL22|TNFRSF11A|CCL5|CCL16|TNFRSF9|TNFAIP3|CCL4 | 77 | 325|532|351|350|484|485|531|529|243|258|561|558|500|364|322|345|334|208|295|410|383|153|356|355|513|359|210|418|321|199|538|149|478|502|367|448|528|438|134|308|283|268|348|481|230|342|276|289|346|465|536|431|310|218|274|450|287|249|305|435|425|329|173|193|526|495|534|163|135|501|473|164|118 | 8.39E-35 |  | GO.0006954 | 3.192445304 | 0 |
| 915 | 97 | GO Process |  | apoptotic process | 1.55E-32 | RELT|TNFRSF1A|MAPK1|TGFB1|TNFAIP1|NFKB1|TNFSF10|TNFSF9|TRAF2|BCL2L2|MAP3K10|IER3|BCL2L10|TRAF5|TRAF4|IL1A|IL1B|ATF2|PRDX5|CCAR1|BNIP2|TNFRSF10B|PDCD4|DOCK1|ATG3|AIFM1|TNFRSF21|TNFRSF11B|BCL2L1|IL12A|AVEN|PPARD|ATG4D|CFLAR|C8orf4|TNFRSF12A|PAWR|AEN|SMAD3|LAMP1|PDCD1|AREL1|FXR1|TGFBR2|MAP3K5|TGFB2|IL6R|MCL1|ATG5|BAG3|MAP3K7|TNFRSF6B|BCL10|IFIT2|CXCR3|TLR4|TGFBR1|TNFRSF1B|HIF3A|BCAP29|CAAP1|BNIP3L|IL33|JAK2|TNFRSF19|PDCD10|BCL2L11|BCL2L15|CIAPIN1|AKTIP|MAP2K7|BCL2|IL6|CXCR4|NFATC4|OXR1|MAP2K4|BCL7B|PDCD6IP|IL10|TICAM2|TNFAIP8|API5|BCLAF1|DICER1|SOD2|APAF1|MAP3K9|MOAP1|TRAF3|IKBKE|TNFRSF11A|MAP2K6|TNFRSF9|ECSCR|PANO|TNFAIP3 | 77 | 532|351|469|350|280|529|202|120|444|551|489|461|550|142|194|322|345|189|208|111|187|410|311|215|113|482|513|359|372|519|145|227|213|133|293|340|396|547|241|360|537|437|245|332|335|376|348|278|312|318|358|481|323|394|230|342|368|276|510|129|428|499|346|309|256|316|234|320|472|424|236|475|218|450|249|144|517|174|307|425|329|185|463|180|121|381|148|290|138|557|548|163|255|473|253|172|164 | 1.12E-34 |  | GO.0006915 | 3.18096683 | 0 |
| 1149 | 108 | GO Process |  | positive regulation of protein modification process | 2.28E-32 | CX3CL1|MAP4K5|TNFRSF1A|MAPK1|TGFB1|CCL1|CCL13|CDKN1B|TGFB3|CCR7|ATG14|TRAF2|LIF|PIAS1|DOCK7|MAP3K10|IRAK2|PDGFRA|CCL21|PDGFRB|MMD|SMAD7|TRAF4|MAP3K8|NOX4|IL1B|MAP4K3|IL11|STAT3|MAP3K13|DOCK3|SOD1|ACKR3|TNFRSF10B|ATG10|CSF1R|BAG4|TGFA|RICTOR|MAP2K1|IL12A|IL13|MAP3K11|OXSR1|CFLAR|TNIP1|C8orf4|CSF1|TNFAIP8L3|SOCS3|SMAD4|MAP4K4|MAP2K3|MAP3K15|ATG7|CCL20|TGFBR2|MAP3K5|MAPK10|MAP3K3|NOS1AP|KIAA1804|TGFB2|IL20|MAPKAPK2|TAB2|IL6R|CACUL1|MAP3K7|MTCP1|IRAK1|BCL10|TLR4|TGFBR1|ZAK|MAP3K19|CCL7|TAB3|IL6ST|JAK2|TNFRSF19|MAP3K4|TANK|PDCD10|AKTIP|MAP2K7|BCL2|TNFSF11|MAP3K1|BCL6|IL6|CXCR4|MAP3K2|TLR6|MAPK9|TNIK|MAP2K4|TRAF6|MAP3K12|MAPKAPK5|MAP3K9|TNFRSF11A|MAP2K6|CCL5|NOS1|CCL16|CCL4|MAP3K14 | 77 | 325|451|351|469|350|484|485|285|265|243|150|444|432|554|413|489|258|516|500|506|427|195|194|165|364|345|141|126|334|490|225|281|247|410|155|153|409|279|302|292|519|418|439|504|133|538|293|149|248|246|470|254|528|384|119|308|332|335|380|299|212|190|376|161|268|184|348|522|358|162|434|323|342|368|264|352|289|206|391|309|256|466|314|316|424|236|475|430|397|310|218|450|374|305|250|535|517|315|426|370|290|163|255|135|508|501|118|171 | 1.70E-34 |  | GO.0031401 | 3.164206515 | 0 |
| 11740 | 393 | GO Process |  | biological regulation | 2.76E-32 | CX3CL1|MAP4K5|RELT|NOX3|TNFRSF1A|TGFBR3|MAPK1|TGFB1|CCL1|CCL13|TNFAIP1|CXCL6|NFKB1|CRTAM|CDKN1B|EXOC2|IL1RL1|BCL9|SOGA1|TGFB3|RHOQ|TNFSF10|RASL11A|TNFSF9|TMX4|CCR7|ATG14|TRAF2|LIF|PIAS1|TXNDC17|BCL2L2|DOCK7|LILRA1|PXDN|MAP3K10|EXOC4|XPO4|IRAK2|PDGFRA|HILPDA|DOCK10|TBRG4|IL36G|IL36B|CCL21|IER3|FDX1|BCL2L10|TRAF5|ARG2|PDGFRB|MAPK6|FAF2|MMD|MSR1|WDR59|SMAD7|SMAD2|RIMS2|TRAF4|SMURF2|MAP3K8|NOX4|IL1A|IL1B|IL7|MAP4K3|ATF2|IL11|STAT3|MAP3K13|PRDX5|LAMP3|CDK6|CCAR1|HPS5|DOCK3|BNIP2|IGSF6|IRF8|SOD1|IL22RA1|ILDR2|ACKR3|IL9|DOCK11|TNFRSF10B|DOCK5|PDCD4|DOCK1|TNFSF4|ATG10|TXNDC11|ATG3|DEPTOR|CSF1R|AIFM1|BAG4|GATAD1|IL17RB|SMAD6|TNFRSF13C|CXCR5|WDR24|TGFA|FAM19A4|TRAT1|CXCL3|CXCL5|SNIP1|IL17RD|RICTOR|TNFRSF21|CREBRF|IL22RA2|TNFRSF11B|DEFB1|HIF1AN|AVPR1A|HPS6|TMX3|NFATC3|LTBP3|MAP2K1|BCL2L1|MAEA|IL16|IL12A|DEFB4A|NKIRAS2|CTLA4|IL13|SOCS5|ALCAM|IL7R|AVEN|CXCL11|MAP3K11|PPARD|OXSR1|CFLAR|IL1RAP|RHOJ|TNIP1|PRKRA|ICOS|C8orf4|NFATC2IP|TNFRSF12A|CSF1|IFNLR1|TGIF1|TNFAIP8L3|PAWR|IL25|SOCS1|PIWIL3|SOCS3|IKZF1|AEN|CCR4|SMAD3|LAMP1|BTLA|PDCD1|BCL9L|CXCL14|IL17F|IL21R|BCL11A|BAG5|KIR2DL4|MIOS|SMAD4|NCR3LG1|LAMTOR4|MAP4K4|GPR29|IFNAR2|IKZF3|MAP2K3|RBPJ|MAP3K15|ATG7|AREL1|PEX2|BCL11B|IRF5|CCRL2|FXR1|CCR9|TXNDC15|CCL20|TGFBR2|MAP3K5|MAPK10|CPLX2|CCL28|SMURF1|CXCL9|MAP3K3|ILF2|NOS1AP|IRF2BP2|KIAA1804|TGFB2|IRF6|TAGAP|IL20|MAPKAPK2|TAB2|HIVEP2|PEX19|THEMIS|IL6R|GATAD2B|IKZF5|MCL1|ATG5|BAG3|CACUL1|PEX11B|MAP3K7|MTCP1|IGSF3|IRAK1BP1|IRAK1|TNFRSF6B|BCL10|BAG2|EXOSC1|IGSF1|LCOR|IFIT3|IFIT2|IL1RAPL2|YWHAB|AGO3|AGO1|AGO4|TRAF3IP1|CXCR3|TGIF2|TLR4|IL2RG|TXNDC8|HLA-DQB1|TGFBR1|ZAK|MAP3K19|TNFSF13B|TNFRSF1B|DOCK9|HIF3A|PLCXD3|BCOR|CCL7|TAB3|TXNDC5|CAAP1|BNIP3L|NFIB|IRF4|IL6ST|IL33|JAK2|TNFRSF19|TXNRD3|IL17RE|LOXL2|EXOSC8|IL17REL|MAP3K4|TANK|PDCD10|IL36RN|BCL2L11|BCL2L15|FDX1L|IGSF11|PRC1|CIAPIN1|AKTIP|SOCS4|ULK2|MAPK8|CXCL12|NFATC2|IRF9|BLOC1S5|PDCD1LG2|SOCS6|MAP2K7|BCL2|TNFSF11|MAP3K1|MAPK4|TXNRD2|CMKBR6|CSF2RB|GATSL3|BCL6|RASSF8|PPARA|IL6|LTBP1|IL1R1|CXCR4|IL18R1|MAP3K2|NFATC4|TLR6|IRAK4|PEX5|MAPKBP1|TMX1|EIF4E3|NKIRAS1|MAPK9|CXCR6|OSGIN2|ITK|HLA-DMB|HLA-B|TNIK|NCOA2|VSIG1|ILF3|DOCK4|OXR1|LAMP2|TBRG1|MAP2K4|BCL7B|PDCD6IP|IL10|IKZF2|TICAM2|TGFBI|ACKR2|PXT1|PEX5L|LAT2|DEFB4B|SMAD1|CDKN2AIP|TNFAIP8|IKBKB|API5|AMBRA1|ATG13|TRAF6|BCLAF1|DICER1|BCORL1|HIF1A|SMAD5|NKRF|IL22|TET2|RASSF3|TNFAIP8L1|BCL7A|SOD2|APAF1|MAP3K12|MAPKAPK5|MAP3K9|MOAP1|TRAF3|NFAT5|LITAF|TXNIP|IKBKE|TNFRSF11A|IER2|PIAS2|ERC1|MAP2K6|CCL5|MR1|NOS1|CCL16|TNFRSF9|ECSCR|PANO|SOCS2|TNFAIP3|SOCS7|CCL4|MAP3K14|GATSL2 | 77 | 325|451|532|203|351|447|469|350|484|485|280|531|529|151|285|339|336|175|317|265|552|202|205|120|301|243|150|444|432|554|140|551|413|344|543|489|159|386|258|516|462|505|496|561|558|500|461|353|550|142|518|506|235|458|427|147|127|195|200|331|194|362|165|364|322|345|266|141|189|126|334|490|208|377|365|111|366|225|187|546|398|281|211|237|247|295|422|410|406|311|215|383|155|156|113|408|153|482|409|530|497|369|224|306|260|279|545|527|356|355|337|136|302|513|296|272|359|112|228|222|209|122|210|441|292|372|207|385|519|158|273|183|418|226|286|176|145|321|439|227|504|133|199|375|538|196|167|293|347|340|149|341|143|248|396|478|460|182|246|220|547|502|241|360|449|537|125|468|367|217|507|124|515|525|470|313|188|254|448|452|392|528|438|384|119|437|269|128|160|134|245|456|494|308|332|335|380|216|488|464|283|299|443|212|178|190|376|487|139|161|268|184|433|521|349|348|414|412|278|312|318|522|343|358|162|270|400|434|481|323|116|137|132|233|404|394|252|440|520|239|240|474|230|259|342|563|238|109|368|264|352|326|276|300|510|407|541|289|206|459|428|499|170|169|391|346|309|256|263|465|480|297|479|466|314|316|536|234|320|512|130|511|472|424|523|214|282|181|115|492|198|373|244|236|475|430|397|231|476|431|524|288|310|549|491|218|154|274|450|287|374|249|305|371|514|298|390|403|417|250|435|446|556|553|114|535|455|378|110|544|144|304|445|517|174|307|425|486|329|157|173|363|328|467|166|193|324|185|526|463|131|559|315|180|121|387|495|201|291|534|294|221|453|303|381|148|426|370|290|138|557|393|395|415|548|163|354|284|401|255|135|540|508|501|473|253|172|419|164|186|118|171|402 | 2.11E-34 |  | GO.0065007 | 3.155909092 | 0 |
| 984 | 100 | GO Process |  | positive regulation of phosphorylation | 2.80E-32 | CX3CL1|MAP4K5|TNFRSF1A|MAPK1|TGFB1|CCL1|CCL13|CDKN1B|TGFB3|CCR7|ATG14|TRAF2|LIF|DOCK7|MAP3K10|IRAK2|PDGFRA|CCL21|PDGFRB|MMD|TRAF4|MAP3K8|NOX4|IL1B|MAP4K3|IL11|STAT3|MAP3K13|DOCK3|SOD1|ACKR3|TNFRSF10B|CSF1R|BAG4|TGFA|RICTOR|MAP2K1|IL12A|IL13|MAP3K11|OXSR1|CFLAR|C8orf4|CSF1|TNFAIP8L3|SOCS3|SMAD4|MAP4K4|MAP2K3|MAP3K15|CCL20|TGFBR2|MAP3K5|MAPK10|MAP3K3|KIAA1804|TGFB2|IL20|MAPKAPK2|TAB2|IL6R|CACUL1|MAP3K7|MTCP1|IRAK1|BCL10|TLR4|TGFBR1|ZAK|MAP3K19|CCL7|TAB3|IL6ST|JAK2|TNFRSF19|MAP3K4|PDCD10|AKTIP|MAP2K7|BCL2|TNFSF11|MAP3K1|IL6|CXCR4|MAP3K2|TLR6|TNIK|MAP2K4|AMBRA1|TRAF6|HIF1A|MAP3K12|MAPKAPK5|MAP3K9|TNFRSF11A|MAP2K6|CCL5|CCL16|CCL4|MAP3K14 | 77 | 325|451|351|469|350|484|485|285|265|243|150|444|432|413|489|258|516|500|506|427|194|165|364|345|141|126|334|490|225|281|247|410|153|409|279|302|292|519|418|439|504|133|293|149|248|246|470|254|528|384|308|332|335|380|299|190|376|161|268|184|348|522|358|162|434|323|342|368|264|352|289|206|391|309|256|466|316|424|236|475|430|397|218|450|374|305|535|517|131|315|495|426|370|290|163|255|135|501|118|171 | 2.19E-34 |  | GO.0042327 | 3.155284197 | 0 |
| 1391 | 118 | GO Process |  | regulation of immune system process | 7.16E-32 | MAPK1|TGFB1|CCL1|CXCL6|NFKB1|CRTAM|IL1RL1|TGFB3|TNFSF9|CCR7|TRAF2|LIF|PIAS1|LILRA1|IRAK2|CCL21|ARG2|SMAD7|MAP3K8|IL1A|IL1B|IL7|STAT3|CDK6|SOD1|DOCK1|TNFSF4|TNFRSF13C|TRAT1|CXCL3|CXCL5|TNFRSF21|NFATC3|IL12A|CTLA4|IL13|SOCS5|IL7R|CXCL11|TNIP1|ICOS|C8orf4|CSF1|IFNLR1|PAWR|SOCS1|SOCS3|SMAD3|LAMP1|BTLA|PDCD1|CXCL14|KIR2DL4|NCR3LG1|GPR29|IFNAR2|IKZF3|CCL20|TGFBR2|MAPK10|CCL28|CXCL9|TGFB2|IL20|MAPKAPK2|TAB2|THEMIS|IL6R|ATG5|MAP3K7|IRAK1|BCL10|AGO3|AGO1|AGO4|TRAF3IP1|CXCR3|TLR4|HLA-DQB1|TNFSF13B|CCL7|TAB3|IRF4|IL6ST|IL33|JAK2|TANK|MAPK8|CXCL12|NFATC2|PDCD1LG2|SOCS6|BCL2|TNFSF11|MAP3K1|CMKBR6|BCL6|IL6|IL1R1|IL18R1|TLR6|IRAK4|MAPK9|ITK|HLA-DMB|HLA-B|IL10|TICAM2|LAT2|IKBKB|TRAF6|HIF1A|TRAF3|IKBKE|MAP2K6|CCL5|TNFAIP3|CCL4 | 77 | 469|350|484|531|529|151|336|265|120|243|444|432|554|344|258|500|518|195|165|322|345|266|334|365|281|215|383|224|527|356|355|513|210|519|183|418|226|176|321|538|167|293|149|341|396|460|246|241|360|449|537|468|515|313|448|452|392|308|332|380|488|283|376|161|268|184|349|348|312|358|434|323|520|239|240|474|230|342|109|326|289|206|169|391|346|309|314|282|181|115|373|244|475|430|397|431|310|218|274|287|305|371|250|556|553|114|425|329|467|526|315|495|557|548|255|135|164|118 | 5.75E-34 |  | GO.0002682 | 3.114508698 | 0 |
| 941 | 97 | GO Process |  | positive regulation of protein phosphorylation | 1.11E-31 | CX3CL1|MAP4K5|TNFRSF1A|MAPK1|TGFB1|CCL1|CCL13|CDKN1B|TGFB3|CCR7|ATG14|TRAF2|LIF|DOCK7|MAP3K10|IRAK2|PDGFRA|CCL21|PDGFRB|MMD|TRAF4|MAP3K8|NOX4|IL1B|MAP4K3|IL11|STAT3|MAP3K13|DOCK3|SOD1|ACKR3|TNFRSF10B|CSF1R|BAG4|TGFA|RICTOR|MAP2K1|IL12A|IL13|MAP3K11|OXSR1|CFLAR|C8orf4|CSF1|TNFAIP8L3|SOCS3|SMAD4|MAP4K4|MAP2K3|MAP3K15|CCL20|TGFBR2|MAP3K5|MAPK10|MAP3K3|KIAA1804|TGFB2|IL20|MAPKAPK2|TAB2|IL6R|CACUL1|MAP3K7|MTCP1|IRAK1|TLR4|TGFBR1|ZAK|MAP3K19|CCL7|TAB3|IL6ST|JAK2|TNFRSF19|MAP3K4|PDCD10|AKTIP|MAP2K7|BCL2|TNFSF11|MAP3K1|IL6|CXCR4|MAP3K2|TLR6|TNIK|MAP2K4|TRAF6|MAP3K12|MAPKAPK5|MAP3K9|TNFRSF11A|MAP2K6|CCL5|CCL16|CCL4|MAP3K14 | 77 | 325|451|351|469|350|484|485|285|265|243|150|444|432|413|489|258|516|500|506|427|194|165|364|345|141|126|334|490|225|281|247|410|153|409|279|302|292|519|418|439|504|133|293|149|248|246|470|254|528|384|308|332|335|380|299|190|376|161|268|184|348|522|358|162|434|342|368|264|352|289|206|391|309|256|466|316|424|236|475|430|397|218|450|374|305|535|517|315|426|370|290|163|255|135|501|118|171 | 9.13E-34 |  | GO.0001934 | 3.095467702 | 0 |
| 1496 | 122 | GO Process |  | positive regulation of cellular protein metabolic process | 1.37E-31 | CX3CL1|MAP4K5|TNFRSF1A|MAPK1|TGFB1|CCL1|CCL13|CDKN1B|TGFB3|TNFSF10|CCR7|ATG14|TRAF2|LIF|PIAS1|DOCK7|MAP3K10|IRAK2|PDGFRA|CCL21|BCL2L10|PDGFRB|MMD|SMAD7|TRAF4|MAP3K8|NOX4|IL1B|MAP4K3|IL11|STAT3|MAP3K13|DOCK3|SOD1|ACKR3|TNFRSF10B|ATG10|CSF1R|AIFM1|BAG4|TGFA|RICTOR|MAP2K1|IL12A|IL13|SOCS5|MAP3K11|OXSR1|CFLAR|TNIP1|C8orf4|CSF1|TNFAIP8L3|SOCS3|SMAD3|SMAD4|MAP4K4|MAP2K3|MAP3K15|ATG7|CCL20|TGFBR2|MAP3K5|MAPK10|SMURF1|MAP3K3|NOS1AP|KIAA1804|TGFB2|IL20|MAPKAPK2|TAB2|IL6R|CACUL1|MAP3K7|MTCP1|IRAK1|BCL10|BAG2|TLR4|TGFBR1|ZAK|MAP3K19|TNFRSF1B|CCL7|TAB3|IL6ST|IL33|JAK2|TNFRSF19|MAP3K4|TANK|PDCD10|BCL2L11|AKTIP|SOCS4|MAP2K7|BCL2|TNFSF11|MAP3K1|BCL6|IL6|CXCR4|MAP3K2|TLR6|MAPK9|TNIK|MAP2K4|TRAF6|APAF1|MAP3K12|MAPKAPK5|MAP3K9|TNFRSF11A|MAP2K6|CCL5|NOS1|CCL16|ECSCR|TNFAIP3|CCL4|MAP3K14 | 77 | 325|451|351|469|350|484|485|285|265|202|243|150|444|432|554|413|489|258|516|500|550|506|427|195|194|165|364|345|141|126|334|490|225|281|247|410|155|153|482|409|279|302|292|519|418|226|439|504|133|538|293|149|248|246|241|470|254|528|384|119|308|332|335|380|464|299|212|190|376|161|268|184|348|522|358|162|434|323|116|342|368|264|352|276|289|206|391|346|309|256|466|314|316|234|424|523|236|475|430|397|310|218|450|374|305|250|535|517|315|148|426|370|290|163|255|135|508|501|253|164|118|171 | 1.15E-33 |  | GO.0032270 | 3.086327943 | 0 |
| 2946 | 176 | GO Process |  | positive regulation of nitrogen compound metabolic process | 1.39E-31 | CX3CL1|MAP4K5|TNFRSF1A|MAPK1|TGFB1|CCL1|CCL13|TNFAIP1|NFKB1|CDKN1B|BCL9|TGFB3|RHOQ|TNFSF10|RASL11A|CCR7|ATG14|TRAF2|LIF|PIAS1|DOCK7|MAP3K10|IRAK2|PDGFRA|CCL21|BCL2L10|PDGFRB|MMD|SMAD7|SMAD2|TRAF4|MAP3K8|NOX4|IL1A|IL1B|MAP4K3|ATF2|IL11|STAT3|MAP3K13|CCAR1|DOCK3|IRF8|SOD1|ACKR3|IL9|TNFRSF10B|TNFSF4|ATG10|CSF1R|AIFM1|BAG4|SMAD6|TNFRSF13C|TGFA|RICTOR|CREBRF|NFATC3|MAP2K1|IL12A|IL13|SOCS5|MAP3K11|PPARD|OXSR1|CFLAR|TNIP1|C8orf4|NFATC2IP|CSF1|TNFAIP8L3|PAWR|IL25|SOCS3|IKZF1|SMAD3|BCL9L|IL17F|BCL11A|SMAD4|MAP4K4|IKZF3|MAP2K3|RBPJ|MAP3K15|ATG7|BCL11B|IRF5|CCL20|TGFBR2|MAP3K5|MAPK10|SMURF1|MAP3K3|ILF2|NOS1AP|KIAA1804|TGFB2|IRF6|IL20|MAPKAPK2|TAB2|IL6R|CACUL1|MAP3K7|MTCP1|IRAK1|BCL10|BAG2|AGO1|CXCR3|TLR4|TGFBR1|ZAK|MAP3K19|TNFRSF1B|HIF3A|CCL7|TAB3|NFIB|IRF4|IL6ST|IL33|JAK2|TNFRSF19|MAP3K4|TANK|PDCD10|BCL2L11|AKTIP|SOCS4|MAPK8|NFATC2|MAP2K7|BCL2|TNFSF11|MAP3K1|BCL6|PPARA|IL6|CXCR4|MAP3K2|NFATC4|TLR6|MAPK9|TNIK|NCOA2|ILF3|MAP2K4|IL10|IKZF2|SMAD1|IKBKB|TRAF6|BCLAF1|HIF1A|SMAD5|NKRF|TET2|APAF1|MAP3K12|MAPKAPK5|MAP3K9|NFAT5|LITAF|TNFRSF11A|IER2|PIAS2|MAP2K6|CCL5|NOS1|CCL16|ECSCR|TNFAIP3|CCL4|MAP3K14 | 77 | 325|451|351|469|350|484|485|280|529|285|175|265|552|202|205|243|150|444|432|554|413|489|258|516|500|550|506|427|195|200|194|165|364|322|345|141|189|126|334|490|111|225|398|281|247|295|410|383|155|153|482|409|369|224|279|302|296|210|292|519|418|226|439|227|504|133|538|293|347|149|248|396|478|246|220|241|125|367|507|470|254|392|528|438|384|119|128|160|308|332|335|380|464|299|443|212|190|376|487|161|268|184|348|522|358|162|434|323|116|239|230|342|368|264|352|276|510|289|206|170|169|391|346|309|256|466|314|316|234|424|523|282|115|236|475|430|397|310|491|218|450|374|249|305|250|535|455|110|517|425|486|193|526|315|180|495|201|291|294|148|426|370|290|393|395|163|354|284|255|135|508|501|253|164|118|171 | 1.19E-33 |  | GO.0051173 | 3.08569852 | 0 |
| 1052 | 102 | GO Process |  | positive regulation of phosphate metabolic process | 1.85E-31 | CX3CL1|MAP4K5|TNFRSF1A|MAPK1|TGFB1|CCL1|CCL13|CDKN1B|TGFB3|CCR7|ATG14|TRAF2|LIF|DOCK7|MAP3K10|IRAK2|PDGFRA|CCL21|PDGFRB|MMD|TRAF4|MAP3K8|NOX4|IL1B|MAP4K3|IL11|STAT3|MAP3K13|DOCK3|SOD1|ACKR3|TNFRSF10B|CSF1R|BAG4|TGFA|RICTOR|MAP2K1|IL12A|IL13|MAP3K11|OXSR1|CFLAR|C8orf4|CSF1|TNFAIP8L3|SOCS3|SMAD3|SMAD4|MAP4K4|MAP2K3|MAP3K15|CCL20|TGFBR2|MAP3K5|MAPK10|MAP3K3|KIAA1804|TGFB2|IL20|MAPKAPK2|TAB2|IL6R|CACUL1|MAP3K7|MTCP1|IRAK1|BCL10|TLR4|TGFBR1|ZAK|MAP3K19|CCL7|TAB3|IL6ST|JAK2|TNFRSF19|MAP3K4|PDCD10|AKTIP|MAP2K7|BCL2|TNFSF11|MAP3K1|IL6|CXCR4|MAP3K2|TLR6|TNIK|MAP2K4|AMBRA1|TRAF6|HIF1A|MAP3K12|MAPKAPK5|MAP3K9|TNFRSF11A|MAP2K6|CCL5|NOS1|CCL16|CCL4|MAP3K14 | 77 | 325|451|351|469|350|484|485|285|265|243|150|444|432|413|489|258|516|500|506|427|194|165|364|345|141|126|334|490|225|281|247|410|153|409|279|302|292|519|418|439|504|133|293|149|248|246|241|470|254|528|384|308|332|335|380|299|190|376|161|268|184|348|522|358|162|434|323|342|368|264|352|289|206|391|309|256|466|316|424|236|475|430|397|218|450|374|305|535|517|131|315|495|426|370|290|163|255|135|508|501|118|171 | 1.63E-33 |  | GO.0045937 | 3.073282827 | 0 |
| 3322 | 188 | GO Process |  | regulation of molecular function | 2.14E-31 | CX3CL1|MAP4K5|TGFBR3|MAPK1|TGFB1|CCL1|CCL13|CXCL6|NFKB1|CDKN1B|TGFB3|TNFSF10|TNFSF9|CCR7|ATG14|TRAF2|LIF|DOCK7|PXDN|MAP3K10|IRAK2|PDGFRA|DOCK10|IL36G|IL36B|CCL21|BCL2L10|TRAF5|PDGFRB|FAF2|MMD|SMAD7|SMAD2|TRAF4|MAP3K8|NOX4|IL1A|IL1B|IL7|MAP4K3|ATF2|IL11|STAT3|MAP3K13|PRDX5|LAMP3|CDK6|DOCK3|BNIP2|SOD1|IL9|DOCK11|TNFRSF10B|DOCK5|PDCD4|DOCK1|TNFSF4|DEPTOR|CSF1R|AIFM1|BAG4|TGFA|CXCL3|CXCL5|RICTOR|TNFRSF11B|AVPR1A|MAP2K1|IL16|IL12A|IL13|SOCS5|CXCL11|MAP3K11|OXSR1|CFLAR|IL1RAP|PRKRA|C8orf4|CSF1|TNFAIP8L3|IL25|SOCS1|SOCS3|SMAD3|CXCL14|IL17F|BAG5|SMAD4|MAP4K4|MAP2K3|MAP3K15|CCL20|TGFBR2|MAP3K5|MAPK10|CCL28|CXCL9|MAP3K3|NOS1AP|KIAA1804|TGFB2|TAGAP|IL20|MAPKAPK2|TAB2|PEX19|IL6R|BAG3|CACUL1|MAP3K7|MTCP1|IRAK1|TNFRSF6B|BCL10|BAG2|IGSF1|IFIT2|YWHAB|TLR4|IL2RG|TGFBR1|ZAK|MAP3K19|TNFSF13B|TNFRSF1B|DOCK9|CCL7|TAB3|NFIB|IRF4|IL33|JAK2|MAP3K4|TANK|PDCD10|IL36RN|BCL2L11|AKTIP|SOCS4|MAPK8|CXCL12|SOCS6|MAP2K7|BCL2|TNFSF11|MAP3K1|CSF2RB|BCL6|PPARA|IL6|CXCR4|IL18R1|MAP3K2|NFATC4|TLR6|IRAK4|OSGIN2|ITK|TNIK|DOCK4|MAP2K4|IL10|TNFAIP8|IKBKB|AMBRA1|TRAF6|HIF1A|IL22|APAF1|MAP3K12|MAPKAPK5|MAP3K9|TRAF3|TXNIP|IKBKE|TNFRSF11A|PIAS2|ERC1|MAP2K6|CCL5|NOS1|CCL16|SOCS2|TNFAIP3|SOCS7|CCL4|MAP3K14 | 77 | 325|451|447|469|350|484|485|531|529|285|265|202|120|243|150|444|432|413|543|489|258|516|505|561|558|500|550|142|506|458|427|195|200|194|165|364|322|345|266|141|189|126|334|490|208|377|365|225|187|281|295|422|410|406|311|215|383|408|153|482|409|279|356|355|302|359|222|292|385|519|418|226|321|439|504|133|199|196|293|149|248|478|460|246|241|468|367|124|470|254|528|384|308|332|335|380|488|283|299|212|190|376|139|161|268|184|521|348|318|522|358|162|434|481|323|116|132|394|440|342|563|368|264|352|326|276|300|289|206|170|169|346|309|466|314|316|536|234|424|523|282|181|244|236|475|430|397|524|310|491|218|450|287|374|249|305|371|446|556|535|544|517|425|185|526|131|315|495|534|148|426|370|290|557|415|548|163|284|401|255|135|508|501|419|164|186|118|171 | 1.96E-33 |  | GO.0065009 | 3.066958623 | 0 |
| 3081 | 180 | GO Process |  | positive regulation of macromolecule metabolic process | 2.39E-31 | CX3CL1|MAP4K5|TNFRSF1A|MAPK1|TGFB1|CCL1|CCL13|TNFAIP1|NFKB1|CDKN1B|BCL9|TGFB3|RHOQ|TNFSF10|RASL11A|CCR7|ATG14|TRAF2|LIF|PIAS1|DOCK7|MAP3K10|IRAK2|PDGFRA|CCL21|BCL2L10|PDGFRB|MMD|SMAD7|SMAD2|RIMS2|TRAF4|MAP3K8|NOX4|IL1A|IL1B|MAP4K3|ATF2|IL11|STAT3|MAP3K13|LAMP3|CCAR1|DOCK3|IRF8|SOD1|ACKR3|IL9|TNFRSF10B|TNFSF4|ATG10|CSF1R|AIFM1|BAG4|SMAD6|TNFRSF13C|TGFA|RICTOR|CREBRF|NFATC3|MAP2K1|IL12A|IL13|SOCS5|IL7R|MAP3K11|PPARD|OXSR1|CFLAR|TNIP1|C8orf4|NFATC2IP|CSF1|TNFAIP8L3|PAWR|IL25|SOCS3|IKZF1|SMAD3|BCL9L|IL17F|BCL11A|SMAD4|MAP4K4|IKZF3|MAP2K3|RBPJ|MAP3K15|ATG7|BCL11B|IRF5|CCL20|TGFBR2|MAP3K5|MAPK10|SMURF1|MAP3K3|ILF2|NOS1AP|KIAA1804|TGFB2|IRF6|IL20|MAPKAPK2|TAB2|IL6R|CACUL1|MAP3K7|MTCP1|IRAK1|BCL10|BAG2|AGO3|AGO1|CXCR3|TLR4|TGFBR1|ZAK|MAP3K19|TNFRSF1B|HIF3A|CCL7|TAB3|NFIB|IRF4|IL6ST|IL33|JAK2|TNFRSF19|MAP3K4|TANK|PDCD10|BCL2L11|AKTIP|SOCS4|MAPK8|NFATC2|MAP2K7|BCL2|TNFSF11|MAP3K1|BCL6|PPARA|IL6|CXCR4|MAP3K2|NFATC4|TLR6|MAPK9|TNIK|NCOA2|ILF3|MAP2K4|IL10|IKZF2|SMAD1|IKBKB|TRAF6|BCLAF1|HIF1A|SMAD5|NKRF|TET2|APAF1|MAP3K12|MAPKAPK5|MAP3K9|NFAT5|LITAF|TNFRSF11A|IER2|PIAS2|MAP2K6|CCL5|NOS1|CCL16|ECSCR|TNFAIP3|CCL4|MAP3K14 | 77 | 325|451|351|469|350|484|485|280|529|285|175|265|552|202|205|243|150|444|432|554|413|489|258|516|500|550|506|427|195|200|331|194|165|364|322|345|141|189|126|334|490|377|111|225|398|281|247|295|410|383|155|153|482|409|369|224|279|302|296|210|292|519|418|226|176|439|227|504|133|538|293|347|149|248|396|478|246|220|241|125|367|507|470|254|392|528|438|384|119|128|160|308|332|335|380|464|299|443|212|190|376|487|161|268|184|348|522|358|162|434|323|116|520|239|230|342|368|264|352|276|510|289|206|170|169|391|346|309|256|466|314|316|234|424|523|282|115|236|475|430|397|310|491|218|450|374|249|305|250|535|455|110|517|425|486|193|526|315|180|495|201|291|294|148|426|370|290|393|395|163|354|284|255|135|508|501|253|164|118|171 | 2.23E-33 |  | GO.0010604 | 3.06216021 | 0 |
| 3060 | 178 | GO Process |  | positive regulation of cellular metabolic process | 1.14E-30 | CX3CL1|MAP4K5|TNFRSF1A|MAPK1|TGFB1|CCL1|CCL13|TNFAIP1|NFKB1|CDKN1B|BCL9|TGFB3|RHOQ|TNFSF10|RASL11A|CCR7|ATG14|TRAF2|LIF|PIAS1|DOCK7|MAP3K10|IRAK2|PDGFRA|CCL21|BCL2L10|PDGFRB|MMD|SMAD7|SMAD2|TRAF4|MAP3K8|NOX4|IL1A|IL1B|MAP4K3|ATF2|IL11|STAT3|MAP3K13|CCAR1|DOCK3|IRF8|SOD1|ACKR3|IL9|TNFRSF10B|TNFSF4|ATG10|CSF1R|AIFM1|BAG4|SMAD6|TNFRSF13C|TGFA|RICTOR|CREBRF|AVPR1A|NFATC3|MAP2K1|IL12A|IL13|SOCS5|MAP3K11|PPARD|OXSR1|CFLAR|TNIP1|C8orf4|NFATC2IP|CSF1|TNFAIP8L3|PAWR|IL25|SOCS3|IKZF1|SMAD3|BCL9L|IL17F|BCL11A|SMAD4|MAP4K4|IKZF3|MAP2K3|RBPJ|MAP3K15|ATG7|BCL11B|IRF5|CCL20|TGFBR2|MAP3K5|MAPK10|SMURF1|MAP3K3|ILF2|NOS1AP|KIAA1804|TGFB2|IRF6|IL20|MAPKAPK2|TAB2|IL6R|CACUL1|MAP3K7|MTCP1|IRAK1|BCL10|BAG2|AGO1|CXCR3|TLR4|TGFBR1|ZAK|MAP3K19|TNFRSF1B|HIF3A|CCL7|TAB3|BNIP3L|NFIB|IRF4|IL6ST|IL33|JAK2|TNFRSF19|MAP3K4|TANK|PDCD10|BCL2L11|AKTIP|SOCS4|NFATC2|MAP2K7|BCL2|TNFSF11|MAP3K1|BCL6|PPARA|IL6|CXCR4|MAP3K2|NFATC4|TLR6|MAPK9|TNIK|NCOA2|ILF3|MAP2K4|IL10|IKZF2|SMAD1|IKBKB|AMBRA1|TRAF6|BCLAF1|HIF1A|SMAD5|NKRF|TET2|APAF1|MAP3K12|MAPKAPK5|MAP3K9|NFAT5|LITAF|TNFRSF11A|IER2|PIAS2|MAP2K6|CCL5|NOS1|CCL16|ECSCR|TNFAIP3|CCL4|MAP3K14 | 77 | 325|451|351|469|350|484|485|280|529|285|175|265|552|202|205|243|150|444|432|554|413|489|258|516|500|550|506|427|195|200|194|165|364|322|345|141|189|126|334|490|111|225|398|281|247|295|410|383|155|153|482|409|369|224|279|302|296|222|210|292|519|418|226|439|227|504|133|538|293|347|149|248|396|478|246|220|241|125|367|507|470|254|392|528|438|384|119|128|160|308|332|335|380|464|299|443|212|190|376|487|161|268|184|348|522|358|162|434|323|116|239|230|342|368|264|352|276|510|289|206|499|170|169|391|346|309|256|466|314|316|234|424|523|115|236|475|430|397|310|491|218|450|374|249|305|250|535|455|110|517|425|486|193|526|131|315|180|495|201|291|294|148|426|370|290|393|395|163|354|284|255|135|508|501|253|164|118|171 | 1.08E-32 |  | GO.0031325 | 2.994309515 | 0 |
| 2152 | 146 | GO Process |  | response to external stimulus | 1.47E-30 | CX3CL1|RELT|NOX3|TNFRSF1A|MAPK1|TGFB1|CCL1|CCL13|CXCL6|NFKB1|CCR7|ATG14|IRAK2|PDGFRA|IL36G|IL36B|CCL21|ARG2|PDGFRB|LOXL1|WDR59|IL1B|CDK6|IRF8|SOD1|IL22RA1|ACKR3|TNFRSF10B|PDCD4|TNFSF4|CSF1R|AIFM1|CXCR5|WDR24|CXCL3|CXCL5|TNFRSF21|TNFRSF11B|DEFB1|AVPR1A|MAP2K1|BCL2L1|IL16|IL12A|DEFB4A|IL13|ALCAM|CXCL11|PPARD|IL1RAP|TNIP1|PRKRA|CSF1|IFNLR1|IL25|CCR4|SMAD3|CXCL14|MIOS|SMAD4|GPR29|IFNAR2|RBPJ|ATG7|BCL11B|IRF5|CCRL2|CCR9|CCL20|TGFBR2|MAP3K5|CCL28|CXCL9|TGFB2|IRF6|MAPKAPK2|IL6R|ATG5|BAG3|IRAK1|TNFRSF6B|BCL10|IFI44|IFI44L|IFIT3|IFIT2|CXCR3|TLR4|HLA-DQB1|TNFRSF1B|CCL7|BNIP3L|NFIB|IRF4|IL33|JAK2|LYST|ATG16L1|IL36RN|BCL2L11|ULK2|MAPK8|CXCL12|IRF9|BLOC1S5|BCL2|TNFSF11|MAP3K1|CMKBR6|CSF2RB|GATSL3|PPARA|IL6|CXCR4|MAP3K2|TLR6|IRAK4|MAPK9|CXCR6|ITK|HLA-B|ILF3|DOCK4|LAMP2|MAP2K4|IL10|TICAM2|ACKR2|DEFB4B|IKBKB|AMBRA1|TRAF6|APAF1|TRAF3|LITAF|TXNIP|IKBKE|TNFRSF11A|CCL5|MR1|CCL16|TNFRSF9|ECSCR|TNFAIP3|CCL4|MAP3K14 | 77 | 325|532|203|351|469|350|484|485|531|529|243|150|258|516|561|558|500|518|506|498|127|345|365|398|281|211|247|410|311|383|153|482|306|260|356|355|513|359|112|222|292|372|385|519|158|418|286|321|227|199|538|196|149|341|478|502|241|468|525|470|448|452|438|119|128|160|134|456|308|332|335|488|283|376|487|268|348|312|318|434|481|323|179|177|404|394|230|342|109|276|289|499|170|169|346|309|555|223|536|234|214|282|181|492|198|475|430|397|431|524|288|491|218|450|374|305|371|250|435|556|114|110|544|304|517|425|329|173|166|526|131|315|148|557|395|415|548|163|135|540|501|473|253|164|118|171 | 1.43E-32 |  | GO.0009605 | 2.983268267 | 0 |
| 512 | 72 | GO Process |  | positive regulation of MAPK cascade | 1.82E-30 | CX3CL1|MAP4K5|MAPK1|TGFB1|CCL1|CCL13|TGFB3|CCR7|TRAF2|LIF|MAP3K10|IRAK2|PDGFRA|CCL21|PDGFRB|TRAF4|MAP3K8|NOX4|IL1B|MAP4K3|IL11|MAP3K13|SOD1|ACKR3|CSF1R|TGFA|MAP2K1|MAP3K11|CFLAR|TNFAIP8L3|MAP4K4|MAP2K3|MAP3K15|CCL20|MAP3K5|MAPK10|MAP3K3|KIAA1804|TGFB2|MAPKAPK2|TAB2|IL6R|MAP3K7|IRAK1|TLR4|TGFBR1|ZAK|CCL7|TAB3|JAK2|TNFRSF19|MAP3K4|PDCD10|MAP2K7|TNFSF11|MAP3K1|IL6|CXCR4|MAP3K2|TLR6|TNIK|MAP2K4|TRAF6|MAP3K12|MAPKAPK5|MAP3K9|TNFRSF11A|MAP2K6|CCL5|CCL16|CCL4|MAP3K14 | 77 | 325|451|469|350|484|485|265|243|444|432|489|258|516|500|506|194|165|364|345|141|126|490|281|247|153|279|292|439|133|248|254|528|384|308|335|380|299|190|376|268|184|348|358|434|342|368|264|289|206|309|256|466|316|236|430|397|218|450|374|305|535|517|315|426|370|290|163|255|135|501|118|171 | 1.81E-32 |  | GO.0043410 | 2.973992861 | 0 |
| 1206 | 107 | GO Process |  | response to biotic stimulus | 3.85E-30 | CX3CL1|RELT|TNFRSF1A|MAPK1|TGFB1|CCL1|CCL13|CXCL6|NFKB1|CRTAM|CCR7|IRAK2|IL36G|IL36B|CCL21|ARG2|LOXL1|IL1B|CDK6|IRF8|IL22RA1|TNFRSF10B|PDCD4|TNFSF4|ATG10|CSF1R|CXCL3|CXCL5|TNFRSF21|TNFRSF11B|DEFB1|BCL2L1|IL12A|DEFB4A|IL13|CXCL11|PPARD|IL1RAP|TNIP1|PRKRA|CSF1|IFNLR1|IL25|CCR4|SMAD3|CXCL14|IFNAR2|RBPJ|ATG7|IRF5|CCL20|MAP3K5|CCL28|CXCL9|IRF6|MAPKAPK2|IL6R|ATG5|IRAK1|TNFRSF6B|BCL10|IFI44|IFI44L|IFIT3|IFIT2|TLR4|HLA-DQB1|TNFRSF1B|CCL7|BNIP3L|IRF4|IL33|JAK2|LYST|ATG16L1|IL36RN|BCL2L11|MAPK8|CXCL12|IRF9|BCL2|CSF2RB|IL6|CXCR4|TLR6|IRAK4|ITK|HLA-B|ILF3|IL10|TICAM2|DEFB4B|IKBKB|TRAF6|APAF1|TRAF3|LITAF|TXNIP|IKBKE|TNFRSF11A|CCL5|MR1|CCL16|TNFRSF9|TNFAIP3|CCL4|MAP3K14 | 77 | 325|532|351|469|350|484|485|531|529|151|243|258|561|558|500|518|498|345|365|398|211|410|311|383|155|153|356|355|513|359|112|372|519|158|418|321|227|199|538|196|149|341|478|502|241|468|452|438|119|160|308|335|488|283|487|268|348|312|434|481|323|179|177|404|394|342|109|276|289|499|169|346|309|555|223|536|234|282|181|492|475|524|218|450|305|371|556|114|110|425|329|166|526|315|148|557|395|415|548|163|135|540|501|473|164|118|171 | 3.90E-32 |  | GO.0009607 | 2.941453927 | 0 |
| 3280 | 184 | GO Process |  | positive regulation of metabolic process | 4.43E-30 | CX3CL1|MAP4K5|TNFRSF1A|MAPK1|TGFB1|CCL1|CCL13|TNFAIP1|NFKB1|CDKN1B|BCL9|TGFB3|RHOQ|TNFSF10|RASL11A|CCR7|ATG14|TRAF2|LIF|PIAS1|DOCK7|MAP3K10|IRAK2|PDGFRA|CCL21|BCL2L10|PDGFRB|MMD|SMAD7|SMAD2|RIMS2|TRAF4|MAP3K8|NOX4|IL1A|IL1B|MAP4K3|ATF2|IL11|STAT3|MAP3K13|PRDX5|LAMP3|CCAR1|DOCK3|IRF8|SOD1|ACKR3|IL9|TNFRSF10B|TNFSF4|ATG10|CSF1R|AIFM1|BAG4|SMAD6|TNFRSF13C|TGFA|RICTOR|CREBRF|AVPR1A|NFATC3|MAP2K1|IL12A|IL13|SOCS5|IL7R|MAP3K11|PPARD|OXSR1|CFLAR|TNIP1|C8orf4|NFATC2IP|CSF1|TNFAIP8L3|PAWR|IL25|SOCS3|IKZF1|SMAD3|BCL9L|IL17F|BCL11A|SMAD4|MAP4K4|IKZF3|MAP2K3|RBPJ|MAP3K15|ATG7|BCL11B|IRF5|CCL20|TGFBR2|MAP3K5|MAPK10|SMURF1|MAP3K3|ILF2|NOS1AP|KIAA1804|TGFB2|IRF6|IL20|MAPKAPK2|TAB2|IL6R|CACUL1|MAP3K7|MTCP1|IRAK1|BCL10|BAG2|AGO3|AGO1|CXCR3|TLR4|TGFBR1|ZAK|MAP3K19|TNFRSF1B|HIF3A|CCL7|TAB3|BNIP3L|NFIB|IRF4|IL6ST|IL33|JAK2|TNFRSF19|MAP3K4|TANK|PDCD10|BCL2L11|AKTIP|SOCS4|MAPK8|NFATC2|MAP2K7|BCL2|TNFSF11|MAP3K1|BCL6|PPARA|IL6|CXCR4|MAP3K2|NFATC4|TLR6|MAPK9|TNIK|NCOA2|ILF3|MAP2K4|IL10|IKZF2|SMAD1|IKBKB|AMBRA1|TRAF6|BCLAF1|HIF1A|SMAD5|NKRF|TET2|APAF1|MAP3K12|MAPKAPK5|MAP3K9|NFAT5|LITAF|TNFRSF11A|IER2|PIAS2|MAP2K6|CCL5|NOS1|CCL16|ECSCR|TNFAIP3|CCL4|MAP3K14 | 77 | 325|451|351|469|350|484|485|280|529|285|175|265|552|202|205|243|150|444|432|554|413|489|258|516|500|550|506|427|195|200|331|194|165|364|322|345|141|189|126|334|490|208|377|111|225|398|281|247|295|410|383|155|153|482|409|369|224|279|302|296|222|210|292|519|418|226|176|439|227|504|133|538|293|347|149|248|396|478|246|220|241|125|367|507|470|254|392|528|438|384|119|128|160|308|332|335|380|464|299|443|212|190|376|487|161|268|184|348|522|358|162|434|323|116|520|239|230|342|368|264|352|276|510|289|206|499|170|169|391|346|309|256|466|314|316|234|424|523|282|115|236|475|430|397|310|491|218|450|374|249|305|250|535|455|110|517|425|486|193|526|131|315|180|495|201|291|294|148|426|370|290|393|395|163|354|284|255|135|508|501|253|164|118|171 | 4.57E-32 |  | GO.0009893 | 2.935359627 | 0 |
| 1080 | 101 | GO Process |  | cell death | 5.46E-30 | RELT|TNFRSF1A|MAPK1|TGFB1|TNFAIP1|NFKB1|CDKN1B|TNFSF10|TNFSF9|TRAF2|BCL2L2|MAP3K10|IER3|BCL2L10|TRAF5|TRAF4|IL1A|IL1B|ATF2|PRDX5|CCAR1|BNIP2|TNFRSF10B|PDCD4|DOCK1|ATG3|AIFM1|TNFRSF21|TNFRSF11B|BCL2L1|IL12A|AVEN|MAP3K11|PPARD|ATG4D|CFLAR|C8orf4|TNFRSF12A|PAWR|AEN|SMAD3|LAMP1|PDCD1|BAG5|AREL1|FXR1|TGFBR2|MAP3K5|TGFB2|IL6R|MCL1|ATG5|BAG3|MAP3K7|TNFRSF6B|BCL10|IFIT2|CXCR3|TLR4|TGFBR1|ZAK|TNFRSF1B|HIF3A|BCAP29|CAAP1|BNIP3L|IL33|JAK2|TNFRSF19|PDCD10|BCL2L11|BCL2L15|CIAPIN1|AKTIP|MAP2K7|BCL2|IL6|CXCR4|NFATC4|OXR1|MAP2K4|BCL7B|PDCD6IP|IL10|TICAM2|TNFAIP8|API5|BCLAF1|DICER1|SOD2|APAF1|MAP3K9|MOAP1|TRAF3|IKBKE|TNFRSF11A|MAP2K6|TNFRSF9|ECSCR|PANO|TNFAIP3 | 77 | 532|351|469|350|280|529|285|202|120|444|551|489|461|550|142|194|322|345|189|208|111|187|410|311|215|113|482|513|359|372|519|145|439|227|213|133|293|340|396|547|241|360|537|124|437|245|332|335|376|348|278|312|318|358|481|323|394|230|342|368|264|276|510|129|428|499|346|309|256|316|234|320|472|424|236|475|218|450|249|144|517|174|307|425|329|185|463|180|121|381|148|290|138|557|548|163|255|473|253|172|164 | 5.73E-32 |  | GO.0008219 | 2.926280736 | 0 |
| 1747 | 129 | GO Process |  | regulation of protein modification process | 9.60E-30 | CX3CL1|MAP4K5|TNFRSF1A|MAPK1|TGFB1|CCL1|CCL13|CDKN1B|TGFB3|CCR7|ATG14|TRAF2|LIF|PIAS1|DOCK7|MAP3K10|IRAK2|PDGFRA|CCL21|PDGFRB|MMD|SMAD7|TRAF4|MAP3K8|NOX4|IL1B|MAP4K3|IL11|STAT3|MAP3K13|CDK6|DOCK3|SOD1|ACKR3|TNFRSF10B|PDCD4|ATG10|DEPTOR|CSF1R|BAG4|SMAD6|TGFA|RICTOR|IL22RA2|MAP2K1|IL12A|IL13|SOCS5|MAP3K11|OXSR1|CFLAR|TNIP1|C8orf4|CSF1|TNFAIP8L3|SOCS1|SOCS3|BAG5|SMAD4|MAP4K4|MAP2K3|MAP3K15|ATG7|CCL20|TGFBR2|MAP3K5|MAPK10|MAP3K3|NOS1AP|KIAA1804|TGFB2|IL20|MAPKAPK2|TAB2|IL6R|ATG5|CACUL1|MAP3K7|MTCP1|IRAK1|BCL10|BAG2|YWHAB|TRAF3IP1|TLR4|TGFBR1|ZAK|MAP3K19|BCOR|CCL7|TAB3|IL6ST|JAK2|TNFRSF19|MAP3K4|TANK|PDCD10|AKTIP|SOCS4|MAPK8|SOCS6|MAP2K7|BCL2|TNFSF11|MAP3K1|BCL6|IL6|CXCR4|MAP3K2|TLR6|MAPK9|TNIK|OXR1|MAP2K4|IKBKB|TRAF6|MAP3K12|MAPKAPK5|MAP3K9|TNFRSF11A|MAP2K6|CCL5|NOS1|CCL16|SOCS2|TNFAIP3|SOCS7|CCL4|MAP3K14 | 77 | 325|451|351|469|350|484|485|285|265|243|150|444|432|554|413|489|258|516|500|506|427|195|194|165|364|345|141|126|334|490|365|225|281|247|410|311|155|408|153|409|369|279|302|272|292|519|418|226|439|504|133|538|293|149|248|460|246|124|470|254|528|384|119|308|332|335|380|299|212|190|376|161|268|184|348|312|522|358|162|434|323|116|440|474|342|368|264|352|541|289|206|391|309|256|466|314|316|424|523|282|244|236|475|430|397|310|218|450|374|305|250|535|144|517|526|315|426|370|290|163|255|135|508|501|419|164|186|118|171 | 1.03E-31 |  | GO.0031399 | 2.901772877 | 0 |
| 1465 | 117 | GO Process |  | regulation of phosphorylation | 1.99E-29 | CX3CL1|MAP4K5|TNFRSF1A|MAPK1|TGFB1|CCL1|CCL13|CDKN1B|TGFB3|CCR7|ATG14|TRAF2|LIF|DOCK7|MAP3K10|IRAK2|PDGFRA|CCL21|PDGFRB|MMD|SMAD7|TRAF4|MAP3K8|NOX4|IL1B|MAP4K3|IL11|STAT3|MAP3K13|CDK6|DOCK3|SOD1|ACKR3|TNFRSF10B|PDCD4|DEPTOR|CSF1R|BAG4|SMAD6|TGFA|RICTOR|IL22RA2|MAP2K1|IL12A|IL13|SOCS5|MAP3K11|OXSR1|CFLAR|TNIP1|C8orf4|CSF1|TNFAIP8L3|SOCS1|SOCS3|SMAD4|MAP4K4|MAP2K3|MAP3K15|CCL20|TGFBR2|MAP3K5|MAPK10|MAP3K3|KIAA1804|TGFB2|IL20|MAPKAPK2|TAB2|IL6R|CACUL1|MAP3K7|MTCP1|IRAK1|BCL10|TRAF3IP1|TLR4|TGFBR1|ZAK|MAP3K19|CCL7|TAB3|IL6ST|JAK2|TNFRSF19|MAP3K4|PDCD10|AKTIP|SOCS4|SOCS6|MAP2K7|BCL2|TNFSF11|MAP3K1|PPARA|IL6|CXCR4|MAP3K2|TLR6|TNIK|MAP2K4|IKBKB|AMBRA1|TRAF6|HIF1A|MAP3K12|MAPKAPK5|MAP3K9|TNFRSF11A|MAP2K6|CCL5|CCL16|SOCS2|TNFAIP3|SOCS7|CCL4|MAP3K14 | 77 | 325|451|351|469|350|484|485|285|265|243|150|444|432|413|489|258|516|500|506|427|195|194|165|364|345|141|126|334|490|365|225|281|247|410|311|408|153|409|369|279|302|272|292|519|418|226|439|504|133|538|293|149|248|460|246|470|254|528|384|308|332|335|380|299|190|376|161|268|184|348|522|358|162|434|323|474|342|368|264|352|289|206|391|309|256|466|316|424|523|244|236|475|430|397|491|218|450|374|305|535|517|526|131|315|495|426|370|290|163|255|135|501|419|164|186|118|171 | 2.17E-31 |  | GO.0042325 | 2.870114692 | 0 |
| 553 | 73 | GO Process |  | positive regulation of kinase activity | 2.23E-29 | MAP4K5|MAPK1|TGFB1|CDKN1B|TGFB3|CCR7|ATG14|TRAF2|MAP3K10|IRAK2|PDGFRA|CCL21|PDGFRB|MMD|TRAF4|MAP3K8|NOX4|IL1B|MAP4K3|MAP3K13|DOCK3|SOD1|TNFRSF10B|CSF1R|TGFA|RICTOR|MAP2K1|MAP3K11|OXSR1|C8orf4|CSF1|TNFAIP8L3|MAP4K4|MAP2K3|MAP3K15|TGFBR2|MAP3K5|MAPK10|MAP3K3|KIAA1804|TGFB2|MAPKAPK2|TAB2|IL6R|CACUL1|MAP3K7|MTCP1|IRAK1|TLR4|TGFBR1|ZAK|MAP3K19|TAB3|JAK2|MAP3K4|PDCD10|MAP2K7|TNFSF11|MAP3K1|CXCR4|MAP3K2|TLR6|TNIK|MAP2K4|AMBRA1|TRAF6|MAP3K12|MAPKAPK5|MAP3K9|TNFRSF11A|MAP2K6|CCL5|MAP3K14 | 77 | 451|469|350|285|265|243|150|444|489|258|516|500|506|427|194|165|364|345|141|490|225|281|410|153|279|302|292|439|504|293|149|248|254|528|384|332|335|380|299|190|376|268|184|348|522|358|162|434|342|368|264|352|206|309|466|316|236|430|397|450|374|305|535|517|131|315|426|370|290|163|255|135|171 | 2.47E-31 |  | GO.0033674 | 2.865169514 | 0 |
| 1042 | 98 | GO Process |  | programmed cell death | 3.31E-29 | RELT|TNFRSF1A|MAPK1|TGFB1|TNFAIP1|NFKB1|CDKN1B|TNFSF10|TNFSF9|TRAF2|BCL2L2|MAP3K10|IER3|BCL2L10|TRAF5|TRAF4|IL1A|IL1B|ATF2|PRDX5|CCAR1|BNIP2|TNFRSF10B|PDCD4|DOCK1|ATG3|AIFM1|TNFRSF21|TNFRSF11B|BCL2L1|IL12A|AVEN|PPARD|ATG4D|CFLAR|C8orf4|TNFRSF12A|PAWR|AEN|SMAD3|LAMP1|PDCD1|AREL1|FXR1|TGFBR2|MAP3K5|TGFB2|IL6R|MCL1|ATG5|BAG3|MAP3K7|TNFRSF6B|BCL10|IFIT2|CXCR3|TLR4|TGFBR1|TNFRSF1B|HIF3A|BCAP29|CAAP1|BNIP3L|IL33|JAK2|TNFRSF19|PDCD10|BCL2L11|BCL2L15|CIAPIN1|AKTIP|MAP2K7|BCL2|IL6|CXCR4|NFATC4|OXR1|MAP2K4|BCL7B|PDCD6IP|IL10|TICAM2|TNFAIP8|API5|BCLAF1|DICER1|SOD2|APAF1|MAP3K9|MOAP1|TRAF3|IKBKE|TNFRSF11A|MAP2K6|TNFRSF9|ECSCR|PANO|TNFAIP3 | 77 | 532|351|469|350|280|529|285|202|120|444|551|489|461|550|142|194|322|345|189|208|111|187|410|311|215|113|482|513|359|372|519|145|227|213|133|293|340|396|547|241|360|537|437|245|332|335|376|348|278|312|318|358|481|323|394|230|342|368|276|510|129|428|499|346|309|256|316|234|320|472|424|236|475|218|450|249|144|517|174|307|425|329|185|463|180|121|381|148|290|138|557|548|163|255|473|253|172|164 | 3.73E-31 |  | GO.0012501 | 2.848017201 | 0 |
| 1370 | 112 | GO Process |  | regulation of protein phosphorylation | 7.69E-29 | CX3CL1|MAP4K5|TNFRSF1A|MAPK1|TGFB1|CCL1|CCL13|CDKN1B|TGFB3|CCR7|ATG14|TRAF2|LIF|DOCK7|MAP3K10|IRAK2|PDGFRA|CCL21|PDGFRB|MMD|SMAD7|TRAF4|MAP3K8|NOX4|IL1B|MAP4K3|IL11|STAT3|MAP3K13|CDK6|DOCK3|SOD1|ACKR3|TNFRSF10B|PDCD4|DEPTOR|CSF1R|BAG4|SMAD6|TGFA|RICTOR|IL22RA2|MAP2K1|IL12A|IL13|SOCS5|MAP3K11|OXSR1|CFLAR|TNIP1|C8orf4|CSF1|TNFAIP8L3|SOCS1|SOCS3|SMAD4|MAP4K4|MAP2K3|MAP3K15|CCL20|TGFBR2|MAP3K5|MAPK10|MAP3K3|KIAA1804|TGFB2|IL20|MAPKAPK2|TAB2|IL6R|CACUL1|MAP3K7|MTCP1|IRAK1|TRAF3IP1|TLR4|TGFBR1|ZAK|MAP3K19|CCL7|TAB3|IL6ST|JAK2|TNFRSF19|MAP3K4|PDCD10|AKTIP|SOCS4|SOCS6|MAP2K7|BCL2|TNFSF11|MAP3K1|IL6|CXCR4|MAP3K2|TLR6|TNIK|MAP2K4|TRAF6|MAP3K12|MAPKAPK5|MAP3K9|TNFRSF11A|MAP2K6|CCL5|CCL16|SOCS2|TNFAIP3|SOCS7|CCL4|MAP3K14 | 77 | 325|451|351|469|350|484|485|285|265|243|150|444|432|413|489|258|516|500|506|427|195|194|165|364|345|141|126|334|490|365|225|281|247|410|311|408|153|409|369|279|302|272|292|519|418|226|439|504|133|538|293|149|248|460|246|470|254|528|384|308|332|335|380|299|190|376|161|268|184|348|522|358|162|434|474|342|368|264|352|289|206|391|309|256|466|316|424|523|244|236|475|430|397|218|450|374|305|535|517|315|426|370|290|163|255|135|501|419|164|186|118|171 | 8.81E-31 |  | GO.0001932 | 2.811407366 | 0 |
| 2668 | 161 | GO Process |  | regulation of protein metabolic process | 8.79E-29 | CX3CL1|MAP4K5|TNFRSF1A|MAPK1|TGFB1|CCL1|CCL13|NFKB1|CDKN1B|TGFB3|TNFSF10|CCR7|ATG14|TRAF2|LIF|PIAS1|DOCK7|MAP3K10|IRAK2|PDGFRA|CCL21|BCL2L10|PDGFRB|MMD|SMAD7|TRAF4|MAP3K8|NOX4|IL1A|IL1B|MAP4K3|IL11|STAT3|MAP3K13|PRDX5|LAMP3|CDK6|DOCK3|SOD1|ACKR3|IL9|TNFRSF10B|PDCD4|ATG10|DEPTOR|CSF1R|AIFM1|BAG4|SMAD6|TNFRSF13C|TGFA|RICTOR|CREBRF|IL22RA2|MAP2K1|IL12A|IL13|SOCS5|MAP3K11|OXSR1|CFLAR|TNIP1|C8orf4|CSF1|TNFAIP8L3|PAWR|SOCS1|PIWIL3|SOCS3|SMAD3|IL17F|BAG5|SMAD4|MAP4K4|MAP2K3|MAP3K15|ATG7|FXR1|CCL20|TGFBR2|MAP3K5|MAPK10|SMURF1|MAP3K3|NOS1AP|KIAA1804|TGFB2|IL20|MAPKAPK2|TAB2|IL6R|ATG5|CACUL1|MAP3K7|MTCP1|IRAK1|TNFRSF6B|BCL10|BAG2|YWHAB|AGO3|AGO1|AGO4|TRAF3IP1|TLR4|TGFBR1|ZAK|MAP3K19|TNFRSF1B|BCOR|CCL7|TAB3|IRF4|IL6ST|IL33|JAK2|TNFRSF19|MAP3K4|TANK|PDCD10|BCL2L11|AKTIP|SOCS4|MAPK8|SOCS6|MAP2K7|BCL2|TNFSF11|MAP3K1|BCL6|IL6|CXCR4|MAP3K2|TLR6|EIF4E3|MAPK9|TNIK|ILF3|OXR1|MAP2K4|IL10|TNFAIP8|IKBKB|TRAF6|APAF1|MAP3K12|MAPKAPK5|MAP3K9|TRAF3|TNFRSF11A|MAP2K6|CCL5|NOS1|CCL16|ECSCR|PANO|SOCS2|TNFAIP3|SOCS7|CCL4|MAP3K14 | 77 | 325|451|351|469|350|484|485|529|285|265|202|243|150|444|432|554|413|489|258|516|500|550|506|427|195|194|165|364|322|345|141|126|334|490|208|377|365|225|281|247|295|410|311|155|408|153|482|409|369|224|279|302|296|272|292|519|418|226|439|504|133|538|293|149|248|396|460|182|246|241|367|124|470|254|528|384|119|245|308|332|335|380|464|299|212|190|376|161|268|184|348|312|522|358|162|434|481|323|116|440|520|239|240|474|342|368|264|352|276|541|289|206|169|391|346|309|256|466|314|316|234|424|523|282|244|236|475|430|397|310|218|450|374|305|403|250|535|110|144|517|425|185|526|315|148|426|370|290|557|163|255|135|508|501|253|172|419|164|186|118|171 | 1.02E-30 |  | GO.0051246 | 2.805601112 | 0 |
| 1173 | 103 | GO Process |  | response to other organism | 1.36E-28 | CX3CL1|RELT|TNFRSF1A|MAPK1|TGFB1|CCL1|CCL13|CXCL6|NFKB1|CCR7|IRAK2|IL36G|IL36B|CCL21|ARG2|LOXL1|IL1B|CDK6|IRF8|IL22RA1|TNFRSF10B|PDCD4|TNFSF4|CSF1R|CXCL3|CXCL5|TNFRSF21|TNFRSF11B|DEFB1|BCL2L1|IL12A|DEFB4A|IL13|CXCL11|PPARD|IL1RAP|TNIP1|PRKRA|CSF1|IFNLR1|IL25|CCR4|SMAD3|CXCL14|IFNAR2|RBPJ|ATG7|IRF5|CCL20|MAP3K5|CCL28|CXCL9|IRF6|MAPKAPK2|IL6R|ATG5|IRAK1|TNFRSF6B|BCL10|IFI44|IFI44L|IFIT3|IFIT2|TLR4|HLA-DQB1|TNFRSF1B|CCL7|BNIP3L|IRF4|IL33|JAK2|LYST|ATG16L1|IL36RN|BCL2L11|MAPK8|CXCL12|IRF9|BCL2|CSF2RB|IL6|CXCR4|TLR6|IRAK4|ITK|HLA-B|ILF3|IL10|TICAM2|DEFB4B|IKBKB|TRAF6|TRAF3|LITAF|IKBKE|TNFRSF11A|CCL5|MR1|CCL16|TNFRSF9|TNFAIP3|CCL4|MAP3K14 | 77 | 325|532|351|469|350|484|485|531|529|243|258|561|558|500|518|498|345|365|398|211|410|311|383|153|356|355|513|359|112|372|519|158|418|321|227|199|538|196|149|341|478|502|241|468|452|438|119|160|308|335|488|283|487|268|348|312|434|481|323|179|177|404|394|342|109|276|289|499|169|346|309|555|223|536|234|282|181|492|475|524|218|450|305|371|556|114|110|425|329|166|526|315|557|395|548|163|135|540|501|473|164|118|171 | 1.61E-30 |  | GO.0051707 | 2.786646109 | 0 |
| 517 | 69 | GO Process |  | positive regulation of protein kinase activity | 5.88E-28 | MAP4K5|MAPK1|TGFB1|CDKN1B|TGFB3|CCR7|TRAF2|MAP3K10|IRAK2|CCL21|PDGFRB|MMD|TRAF4|MAP3K8|NOX4|IL1B|MAP4K3|MAP3K13|DOCK3|SOD1|TNFRSF10B|CSF1R|TGFA|RICTOR|MAP2K1|MAP3K11|OXSR1|C8orf4|CSF1|MAP4K4|MAP2K3|MAP3K15|TGFBR2|MAP3K5|MAPK10|MAP3K3|KIAA1804|TGFB2|MAPKAPK2|TAB2|IL6R|CACUL1|MAP3K7|MTCP1|IRAK1|TLR4|TGFBR1|ZAK|MAP3K19|TAB3|JAK2|MAP3K4|PDCD10|MAP2K7|TNFSF11|MAP3K1|CXCR4|MAP3K2|TLR6|TNIK|MAP2K4|TRAF6|MAP3K12|MAPKAPK5|MAP3K9|TNFRSF11A|MAP2K6|CCL5|MAP3K14 | 77 | 451|469|350|285|265|243|444|489|258|500|506|427|194|165|364|345|141|490|225|281|410|153|279|302|292|439|504|293|149|254|528|384|332|335|380|299|190|376|268|184|348|522|358|162|434|342|368|264|352|206|309|466|316|236|430|397|450|374|305|535|517|315|426|370|290|163|255|135|171 | 7.19E-30 |  | GO.0045860 | 2.723062267 | 0 |
| 2486 | 153 | GO Process |  | regulation of cellular protein metabolic process | 6.34E-28 | CX3CL1|MAP4K5|TNFRSF1A|MAPK1|TGFB1|CCL1|CCL13|NFKB1|CDKN1B|TGFB3|TNFSF10|CCR7|ATG14|TRAF2|LIF|PIAS1|DOCK7|MAP3K10|IRAK2|PDGFRA|CCL21|BCL2L10|PDGFRB|MMD|SMAD7|TRAF4|MAP3K8|NOX4|IL1B|MAP4K3|IL11|STAT3|MAP3K13|PRDX5|LAMP3|CDK6|DOCK3|SOD1|ACKR3|TNFRSF10B|PDCD4|ATG10|DEPTOR|CSF1R|AIFM1|BAG4|SMAD6|TGFA|RICTOR|IL22RA2|MAP2K1|IL12A|IL13|SOCS5|MAP3K11|OXSR1|CFLAR|TNIP1|C8orf4|CSF1|TNFAIP8L3|SOCS1|PIWIL3|SOCS3|SMAD3|BAG5|SMAD4|MAP4K4|MAP2K3|MAP3K15|ATG7|FXR1|CCL20|TGFBR2|MAP3K5|MAPK10|SMURF1|MAP3K3|NOS1AP|KIAA1804|TGFB2|IL20|MAPKAPK2|TAB2|IL6R|ATG5|CACUL1|MAP3K7|MTCP1|IRAK1|TNFRSF6B|BCL10|BAG2|YWHAB|AGO3|AGO1|AGO4|TRAF3IP1|TLR4|TGFBR1|ZAK|MAP3K19|TNFRSF1B|BCOR|CCL7|TAB3|IL6ST|IL33|JAK2|TNFRSF19|MAP3K4|TANK|PDCD10|BCL2L11|AKTIP|SOCS4|MAPK8|SOCS6|MAP2K7|BCL2|TNFSF11|MAP3K1|BCL6|IL6|CXCR4|MAP3K2|TLR6|EIF4E3|MAPK9|TNIK|ILF3|OXR1|MAP2K4|IL10|TNFAIP8|IKBKB|TRAF6|APAF1|MAP3K12|MAPKAPK5|MAP3K9|TNFRSF11A|MAP2K6|CCL5|NOS1|CCL16|ECSCR|PANO|SOCS2|TNFAIP3|SOCS7|CCL4|MAP3K14 | 77 | 325|451|351|469|350|484|485|529|285|265|202|243|150|444|432|554|413|489|258|516|500|550|506|427|195|194|165|364|345|141|126|334|490|208|377|365|225|281|247|410|311|155|408|153|482|409|369|279|302|272|292|519|418|226|439|504|133|538|293|149|248|460|182|246|241|124|470|254|528|384|119|245|308|332|335|380|464|299|212|190|376|161|268|184|348|312|522|358|162|434|481|323|116|440|520|239|240|474|342|368|264|352|276|541|289|206|391|346|309|256|466|314|316|234|424|523|282|244|236|475|430|397|310|218|450|374|305|403|250|535|110|144|517|425|185|526|315|148|426|370|290|163|255|135|508|501|253|172|419|164|186|118|171 | 7.87E-30 |  | GO.0032268 | 2.719791074 | 0 |
| 882 | 88 | GO Process |  | positive regulation of immune system process | 1.13E-27 | MAPK1|TGFB1|CCL1|CXCL6|NFKB1|CRTAM|IL1RL1|TNFSF9|CCR7|TRAF2|LIF|IRAK2|CCL21|MAP3K8|IL1A|IL1B|IL7|STAT3|DOCK1|TNFSF4|TNFRSF13C|TRAT1|CXCL3|CXCL5|TNFRSF21|IL12A|CTLA4|IL13|SOCS5|IL7R|CXCL11|TNIP1|ICOS|CSF1|SOCS1|LAMP1|BTLA|PDCD1|CXCL14|GPR29|CCL20|TGFBR2|CXCL9|TGFB2|IL20|MAPKAPK2|TAB2|THEMIS|IL6R|MAP3K7|IRAK1|BCL10|TLR4|HLA-DQB1|TNFSF13B|CCL7|TAB3|IL6ST|IL33|TANK|CXCL12|NFATC2|PDCD1LG2|BCL2|TNFSF11|MAP3K1|CMKBR6|BCL6|IL6|IL1R1|IL18R1|TLR6|IRAK4|ITK|HLA-DMB|HLA-B|IL10|TICAM2|LAT2|IKBKB|TRAF6|HIF1A|TRAF3|IKBKE|MAP2K6|CCL5|TNFAIP3|CCL4 | 77 | 469|350|484|531|529|151|336|120|243|444|432|258|500|165|322|345|266|334|215|383|224|527|356|355|513|519|183|418|226|176|321|538|167|149|460|360|449|537|468|448|308|332|283|376|161|268|184|349|348|358|434|323|342|109|326|289|206|391|346|314|181|115|373|475|430|397|431|310|218|274|287|305|371|556|553|114|425|329|467|526|315|495|557|548|255|135|164|118 | 1.43E-29 |  | GO.0002684 | 2.694692156 | 0 |
| 1657 | 120 | GO Process |  | regulation of phosphate metabolic process | 1.11E-26 | CX3CL1|MAP4K5|TNFRSF1A|MAPK1|TGFB1|CCL1|CCL13|CDKN1B|TGFB3|CCR7|ATG14|TRAF2|LIF|DOCK7|MAP3K10|IRAK2|PDGFRA|CCL21|PDGFRB|MMD|SMAD7|TRAF4|MAP3K8|NOX4|IL1B|MAP4K3|IL11|STAT3|MAP3K13|CDK6|DOCK3|SOD1|ACKR3|TNFRSF10B|PDCD4|DEPTOR|CSF1R|BAG4|SMAD6|TGFA|RICTOR|IL22RA2|MAP2K1|IL12A|IL13|SOCS5|MAP3K11|OXSR1|CFLAR|TNIP1|C8orf4|CSF1|TNFAIP8L3|SOCS1|SOCS3|SMAD3|SMAD4|MAP4K4|MAP2K3|MAP3K15|CCL20|TGFBR2|MAP3K5|MAPK10|MAP3K3|KIAA1804|TGFB2|IL20|MAPKAPK2|TAB2|IL6R|CACUL1|MAP3K7|MTCP1|IRAK1|BCL10|YWHAB|TRAF3IP1|TLR4|TGFBR1|ZAK|MAP3K19|CCL7|TAB3|IL6ST|JAK2|TNFRSF19|MAP3K4|PDCD10|AKTIP|SOCS4|SOCS6|MAP2K7|BCL2|TNFSF11|MAP3K1|PPARA|IL6|CXCR4|MAP3K2|TLR6|TNIK|MAP2K4|IKBKB|AMBRA1|TRAF6|HIF1A|MAP3K12|MAPKAPK5|MAP3K9|TNFRSF11A|MAP2K6|CCL5|NOS1|CCL16|SOCS2|TNFAIP3|SOCS7|CCL4|MAP3K14 | 77 | 325|451|351|469|350|484|485|285|265|243|150|444|432|413|489|258|516|500|506|427|195|194|165|364|345|141|126|334|490|365|225|281|247|410|311|408|153|409|369|279|302|272|292|519|418|226|439|504|133|538|293|149|248|460|246|241|470|254|528|384|308|332|335|380|299|190|376|161|268|184|348|522|358|162|434|323|440|474|342|368|264|352|289|206|391|309|256|466|316|424|523|244|236|475|430|397|491|218|450|374|305|535|517|526|131|315|495|426|370|290|163|255|135|508|501|419|164|186|118|171 | 1.42E-28 |  | GO.0019220 | 2.595467702 | 0 |
| 108 | 37 | KEGG Pathways | | TNF signaling pathway | 1.67E-26 | CX3CL1|TNFRSF1A|MAPK1|NFKB1|TRAF2|LIF|TRAF5|MAP3K8|IL1B|ATF2|BAG4|CXCL3|CXCL5|MAP2K1|CFLAR|CSF1|SOCS3|MAP2K3|CCL20|MAP3K5|MAPK10|TAB2|MAP3K7|TNFRSF1B|TAB3|MAPK8|MAP2K7|IL6|IL18R1|MAPK9|MAP2K4|IKBKB|TRAF3|MAP2K6|CCL5|TNFAIP3|MAP3K14 | 77 | 325|351|469|529|444|432|142|165|345|189|409|356|355|292|133|149|246|528|308|335|380|184|358|276|206|282|236|218|287|250|517|526|557|255|135|164|171 | 1.70E-28 |  | hsa04668 | 2.577728353 | 0 |
| 1299 | 105 | GO Process |  | regulation of response to stress | 1.76E-26 | CX3CL1|MAP4K5|TNFRSF1A|MAPK1|NFKB1|CRTAM|IL1RL1|CCR7|TRAF2|PIAS1|MAP3K10|IRAK2|PDGFRA|CCL21|IER3|ARG2|TRAF4|IL1B|CDK6|SOD1|ACKR3|PDCD4|TNFSF4|BAG4|IL17RB|RICTOR|CREBRF|IL22RA2|MAP2K1|BCL2L1|IL12A|SOCS5|MAP3K11|PPARD|TNIP1|TNFRSF12A|IFNLR1|PAWR|SOCS1|SOCS3|SMAD3|LAMP1|IL17F|BAG5|MAP4K4|IFNAR2|ATG7|TGFBR2|MAP3K5|MAP3K3|KIAA1804|TGFB2|IL20|MAPKAPK2|TAB2|MCL1|BAG3|MAP3K7|IRAK1|BCL10|BAG2|TRAF3IP1|TLR4|ZAK|TNFRSF1B|TAB3|IRF4|IL6ST|IL33|JAK2|TNFRSF19|MAP3K4|TANK|PDCD10|BCL2L11|CXCL12|MAP2K7|TNFSF11|MAP3K1|BCL6|PPARA|IL6|IL1R1|MAP3K2|TLR6|IRAK4|MAPKBP1|HLA-B|TNIK|OXR1|MAP2K4|IL10|TICAM2|IKBKB|TRAF6|BCLAF1|HIF1A|SOD2|MAP3K9|TRAF3|IKBKE|TNFRSF11A|MAP2K6|CCL5|TNFAIP3 | 77 | 325|451|351|469|529|151|336|243|444|554|489|258|516|500|461|518|194|345|365|281|247|311|383|409|497|302|296|272|292|372|519|226|439|227|538|340|341|396|460|246|241|360|367|124|254|452|119|332|335|299|190|376|161|268|184|278|318|358|434|323|116|474|342|264|276|206|169|391|346|309|256|466|314|316|234|181|236|430|397|310|491|218|274|374|305|371|298|114|535|144|517|425|329|526|315|180|495|381|290|557|548|163|255|135|164 | 2.32E-28 |  | GO.0080134 | 2.575448733 | 0 |
| 849 | 84 | GO Process |  | regulation of kinase activity | 4.29E-26 | MAP4K5|MAPK1|TGFB1|CDKN1B|TGFB3|CCR7|ATG14|TRAF2|MAP3K10|IRAK2|PDGFRA|CCL21|PDGFRB|MMD|TRAF4|MAP3K8|NOX4|IL1B|MAP4K3|MAP3K13|CDK6|DOCK3|SOD1|TNFRSF10B|PDCD4|DEPTOR|CSF1R|TGFA|RICTOR|MAP2K1|SOCS5|MAP3K11|OXSR1|C8orf4|CSF1|TNFAIP8L3|SOCS1|SOCS3|MAP4K4|MAP2K3|MAP3K15|TGFBR2|MAP3K5|MAPK10|MAP3K3|KIAA1804|TGFB2|MAPKAPK2|TAB2|IL6R|CACUL1|MAP3K7|MTCP1|IRAK1|TLR4|TGFBR1|ZAK|MAP3K19|TAB3|JAK2|MAP3K4|PDCD10|SOCS4|SOCS6|MAP2K7|TNFSF11|MAP3K1|CXCR4|MAP3K2|TLR6|TNIK|MAP2K4|AMBRA1|TRAF6|MAP3K12|MAPKAPK5|MAP3K9|TNFRSF11A|MAP2K6|CCL5|SOCS2|TNFAIP3|SOCS7|MAP3K14 | 77 | 451|469|350|285|265|243|150|444|489|258|516|500|506|427|194|165|364|345|141|490|365|225|281|410|311|408|153|279|302|292|226|439|504|293|149|248|460|246|254|528|384|332|335|380|299|190|376|268|184|348|522|358|162|434|342|368|264|352|206|309|466|316|523|244|236|430|397|450|374|305|535|517|131|315|426|370|290|163|255|135|419|164|186|171 | 5.82E-28 |  | GO.0043549 | 2.536754271 | 0 |
| 349 | 56 | GO Process |  | signal transduction by protein phosphorylation | 5.39E-26 | MAP4K5|MAPK1|TGFB1|NFKB1|MAP3K10|IRAK2|PDGFRA|PDGFRB|MAPK6|MAP3K8|IL1B|MAP4K3|MAP3K13|IL17RD|MAP2K1|MAP3K11|OXSR1|MAP4K4|MAP2K3|MAP3K15|MAP3K5|MAPK10|MAP3K3|KIAA1804|MAPKAPK2|TAB2|MAP3K7|IRAK1|YWHAB|IL2RG|ZAK|MAP3K19|TAB3|JAK2|TNFRSF19|MAP3K4|MAPK8|MAP2K7|TNFSF11|MAP3K1|MAPK4|CSF2RB|MAP3K2|IRAK4|MAPK9|TNIK|MAP2K4|SMAD1|IKBKB|TRAF6|MAP3K12|MAPKAPK5|MAP3K9|MAP2K6|CCL5|MAP3K14 | 77 | 451|469|350|529|489|258|516|506|235|165|345|141|490|136|292|439|504|254|528|384|335|380|299|190|268|184|358|434|440|563|264|352|206|309|256|466|282|236|430|397|231|524|374|371|250|535|517|193|526|315|426|370|290|255|135|171 | 7.42E-28 |  | GO.0023014 | 2.526841123 | 0 |
| 264 | 50 | GO Process |  | positive regulation of MAP kinase activity | 5.65E-26 | MAP4K5|MAPK1|TGFB1|TGFB3|TRAF2|MAP3K10|IRAK2|PDGFRB|MAP3K8|NOX4|IL1B|MAP4K3|MAP3K13|SOD1|TGFA|MAP2K1|MAP3K11|MAP4K4|MAP2K3|MAP3K15|MAP3K5|MAPK10|MAP3K3|KIAA1804|MAPKAPK2|TAB2|MAP3K7|IRAK1|TLR4|TGFBR1|ZAK|TAB3|JAK2|MAP3K4|PDCD10|MAP2K7|TNFSF11|MAP3K1|CXCR4|MAP3K2|TLR6|TNIK|MAP2K4|TRAF6|MAP3K12|MAPKAPK5|MAP3K9|TNFRSF11A|MAP2K6|MAP3K14 | 77 | 451|469|350|265|444|489|258|506|165|364|345|141|490|281|279|292|439|254|528|384|335|380|299|190|268|184|358|434|342|368|264|206|309|466|316|236|430|397|450|374|305|535|517|315|426|370|290|163|255|171 | 7.87E-28 |  | GO.0043406 | 2.524795155 | 0 |
| 323 | 54 | GO Process |  | MAPK cascade | 8.40E-26 | MAP4K5|MAPK1|TGFB1|NFKB1|MAP3K10|IRAK2|PDGFRA|PDGFRB|MAPK6|MAP3K8|IL1B|MAP4K3|MAP3K13|IL17RD|MAP2K1|MAP3K11|MAP4K4|MAP2K3|MAP3K15|MAP3K5|MAPK10|MAP3K3|KIAA1804|MAPKAPK2|TAB2|MAP3K7|IRAK1|YWHAB|IL2RG|ZAK|TAB3|JAK2|TNFRSF19|MAP3K4|MAPK8|MAP2K7|TNFSF11|MAP3K1|MAPK4|CSF2RB|MAP3K2|IRAK4|MAPK9|TNIK|MAP2K4|SMAD1|IKBKB|TRAF6|MAP3K12|MAPKAPK5|MAP3K9|MAP2K6|CCL5|MAP3K14 | 77 | 451|469|350|529|489|258|516|506|235|165|345|141|490|136|292|439|254|528|384|335|380|299|190|268|184|358|434|440|563|264|206|309|256|466|282|236|430|397|231|524|374|371|250|535|517|193|526|315|426|370|290|255|135|171 | 1.19E-27 |  | GO.0000165 | 2.507572071 | 0 |
| 1381 | 107 | GO Process |  | positive regulation of catalytic activity | 1.16E-25 | CX3CL1|MAP4K5|MAPK1|TGFB1|CCL1|CCL13|CDKN1B|TGFB3|TNFSF10|CCR7|ATG14|TRAF2|DOCK7|MAP3K10|IRAK2|PDGFRA|DOCK10|CCL21|BCL2L10|PDGFRB|MMD|TRAF4|MAP3K8|NOX4|IL1B|MAP4K3|STAT3|MAP3K13|DOCK3|BNIP2|SOD1|DOCK11|TNFRSF10B|DOCK1|CSF1R|AIFM1|TGFA|RICTOR|AVPR1A|MAP2K1|MAP3K11|OXSR1|CFLAR|PRKRA|C8orf4|CSF1|TNFAIP8L3|SMAD3|MAP4K4|MAP2K3|MAP3K15|CCL20|TGFBR2|MAP3K5|MAPK10|MAP3K3|NOS1AP|KIAA1804|TGFB2|TAGAP|MAPKAPK2|TAB2|IL6R|CACUL1|MAP3K7|MTCP1|IRAK1|BCL10|YWHAB|TLR4|TGFBR1|ZAK|MAP3K19|DOCK9|CCL7|TAB3|JAK2|MAP3K4|TANK|PDCD10|BCL2L11|MAPK8|MAP2K7|BCL2|TNFSF11|MAP3K1|CXCR4|MAP3K2|TLR6|ITK|TNIK|DOCK4|MAP2K4|AMBRA1|TRAF6|HIF1A|APAF1|MAP3K12|MAPKAPK5|MAP3K9|TNFRSF11A|MAP2K6|CCL5|NOS1|CCL16|CCL4|MAP3K14 | 77 | 325|451|469|350|484|485|285|265|202|243|150|444|413|489|258|516|505|500|550|506|427|194|165|364|345|141|334|490|225|187|281|422|410|215|153|482|279|302|222|292|439|504|133|196|293|149|248|241|254|528|384|308|332|335|380|299|212|190|376|139|268|184|348|522|358|162|434|323|440|342|368|264|352|300|289|206|309|466|314|316|234|282|236|475|430|397|450|374|305|556|535|544|517|131|315|495|148|426|370|290|163|255|135|508|501|118|171 | 1.67E-27 |  | GO.0043085 | 2.493554201 | 0 |
| 788 | 80 | GO Process |  | regulation of protein kinase activity | 1.90E-25 | MAP4K5|MAPK1|TGFB1|CDKN1B|TGFB3|CCR7|TRAF2|MAP3K10|IRAK2|CCL21|PDGFRB|MMD|TRAF4|MAP3K8|NOX4|IL1B|MAP4K3|MAP3K13|CDK6|DOCK3|SOD1|TNFRSF10B|PDCD4|DEPTOR|CSF1R|TGFA|RICTOR|MAP2K1|SOCS5|MAP3K11|OXSR1|C8orf4|CSF1|SOCS1|SOCS3|MAP4K4|MAP2K3|MAP3K15|TGFBR2|MAP3K5|MAPK10|MAP3K3|KIAA1804|TGFB2|MAPKAPK2|TAB2|IL6R|CACUL1|MAP3K7|MTCP1|IRAK1|TLR4|TGFBR1|ZAK|MAP3K19|TAB3|JAK2|MAP3K4|PDCD10|SOCS4|SOCS6|MAP2K7|TNFSF11|MAP3K1|CXCR4|MAP3K2|TLR6|TNIK|MAP2K4|TRAF6|MAP3K12|MAPKAPK5|MAP3K9|TNFRSF11A|MAP2K6|CCL5|SOCS2|TNFAIP3|SOCS7|MAP3K14 | 77 | 451|469|350|285|265|243|444|489|258|500|506|427|194|165|364|345|141|490|365|225|281|410|311|408|153|279|302|292|226|439|504|293|149|460|246|254|528|384|332|335|380|299|190|376|268|184|348|522|358|162|434|342|368|264|352|206|309|466|316|523|244|236|430|397|450|374|305|535|517|315|426|370|290|163|255|135|419|164|186|171 | 2.76E-27 |  | GO.0045859 | 2.47212464 | 0 |
| 347 | 55 | GO Process |  | activation of protein kinase activity | 2.66E-25 | MAP4K5|MAPK1|TGFB1|TGFB3|TRAF2|MAP3K10|IRAK2|TRAF4|MAP3K8|IL1B|MAP4K3|MAP3K13|SOD1|TNFRSF10B|TGFA|RICTOR|MAP2K1|MAP3K11|OXSR1|MAP4K4|MAP2K3|MAP3K15|TGFBR2|MAP3K5|MAPK10|MAP3K3|KIAA1804|TGFB2|MAPKAPK2|TAB2|MAP3K7|MTCP1|IRAK1|TLR4|TGFBR1|ZAK|MAP3K19|TAB3|JAK2|MAP3K4|MAP2K7|TNFSF11|MAP3K1|CXCR4|MAP3K2|TLR6|TNIK|MAP2K4|TRAF6|MAP3K12|MAPKAPK5|MAP3K9|MAP2K6|CCL5|MAP3K14 | 77 | 451|469|350|265|444|489|258|194|165|345|141|490|281|410|279|302|292|439|504|254|528|384|332|335|380|299|190|376|268|184|358|162|434|342|368|264|352|206|309|466|236|430|397|450|374|305|535|517|315|426|370|290|255|135|171 | 3.92E-27 |  | GO.0032147 | 2.457511836 | 0 |
| 216 | 46 | GO Function | | cytokine activity | 4.75E-25 | CX3CL1|TGFB1|CCL1|CCL13|CXCL6|TGFB3|TNFSF10|TNFSF9|LIF|IL36G|IL36B|CCL21|IL1A|IL1B|IL7|IL11|IL9|TNFSF4|CXCL3|CXCL5|TNFRSF11B|IL16|IL12A|IL13|CXCL11|CSF1|IL25|CXCL14|IL17F|CCL20|CCL28|CXCL9|TGFB2|IL20|TNFSF13B|CCL7|IL33|IL36RN|CXCL12|TNFSF11|IL6|IL10|IL22|CCL5|CCL16|CCL4 | 77 | 325|350|484|485|531|265|202|120|432|561|558|500|322|345|266|126|295|383|356|355|359|385|519|418|321|149|478|468|367|308|488|283|376|161|326|289|346|536|181|430|218|425|534|135|501|118 | 1.48E-27 |  | GO.0005125 | 2.432330639 | 0 |
| 1713 | 119 | GO Process |  | positive regulation of molecular function | 5.72E-25 | CX3CL1|MAP4K5|MAPK1|TGFB1|CCL1|CCL13|NFKB1|CDKN1B|TGFB3|TNFSF10|CCR7|ATG14|TRAF2|DOCK7|MAP3K10|IRAK2|PDGFRA|DOCK10|CCL21|BCL2L10|TRAF5|PDGFRB|MMD|TRAF4|MAP3K8|NOX4|IL1B|MAP4K3|ATF2|STAT3|MAP3K13|DOCK3|BNIP2|SOD1|DOCK11|TNFRSF10B|DOCK1|CSF1R|AIFM1|TGFA|RICTOR|AVPR1A|MAP2K1|MAP3K11|OXSR1|CFLAR|IL1RAP|PRKRA|C8orf4|CSF1|TNFAIP8L3|SMAD3|MAP4K4|MAP2K3|MAP3K15|CCL20|TGFBR2|MAP3K5|MAPK10|MAP3K3|NOS1AP|KIAA1804|TGFB2|TAGAP|MAPKAPK2|TAB2|IL6R|CACUL1|MAP3K7|MTCP1|IRAK1|BCL10|YWHAB|TLR4|TGFBR1|ZAK|MAP3K19|DOCK9|CCL7|TAB3|IRF4|JAK2|MAP3K4|TANK|PDCD10|BCL2L11|AKTIP|MAPK8|MAP2K7|BCL2|TNFSF11|MAP3K1|IL6|CXCR4|IL18R1|MAP3K2|TLR6|IRAK4|ITK|TNIK|DOCK4|MAP2K4|IL10|IKBKB|AMBRA1|TRAF6|HIF1A|APAF1|MAP3K12|MAPKAPK5|MAP3K9|TNFRSF11A|ERC1|MAP2K6|CCL5|NOS1|CCL16|CCL4|MAP3K14 | 77 | 325|451|469|350|484|485|529|285|265|202|243|150|444|413|489|258|516|505|500|550|142|506|427|194|165|364|345|141|189|334|490|225|187|281|422|410|215|153|482|279|302|222|292|439|504|133|199|196|293|149|248|241|254|528|384|308|332|335|380|299|212|190|376|139|268|184|348|522|358|162|434|323|440|342|368|264|352|300|289|206|169|309|466|314|316|234|424|282|236|475|430|397|218|450|287|374|305|371|556|535|544|517|425|526|131|315|495|148|426|370|290|163|401|255|135|508|501|118|171 | 8.53E-27 |  | GO.0044093 | 2.424260397 | 0 |
| 340 | 54 | GO Process |  | positive regulation of protein serine/threonine kinase activity | 7.24E-25 | MAP4K5|MAPK1|TGFB1|CDKN1B|TGFB3|TRAF2|MAP3K10|IRAK2|PDGFRB|MAP3K8|NOX4|IL1B|MAP4K3|MAP3K13|SOD1|CSF1R|TGFA|MAP2K1|MAP3K11|C8orf4|MAP4K4|MAP2K3|MAP3K15|MAP3K5|MAPK10|MAP3K3|KIAA1804|MAPKAPK2|TAB2|MAP3K7|MTCP1|IRAK1|TLR4|TGFBR1|ZAK|TAB3|JAK2|MAP3K4|PDCD10|MAP2K7|TNFSF11|MAP3K1|CXCR4|MAP3K2|TLR6|TNIK|MAP2K4|TRAF6|MAP3K12|MAPKAPK5|MAP3K9|TNFRSF11A|MAP2K6|MAP3K14 | 77 | 451|469|350|285|265|444|489|258|506|165|364|345|141|490|281|153|279|292|439|293|254|528|384|335|380|299|190|268|184|358|162|434|342|368|264|206|309|466|316|236|430|397|450|374|305|535|517|315|426|370|290|163|255|171 | 1.09E-26 |  | GO.0071902 | 2.414026143 | 0 |
| 185 | 43 | UniProt Keywords | | Cytokine | 1.08E-24 | CX3CL1|CCL1|CCL13|CXCL6|TNFSF10|TNFSF9|LIF|IL36G|IL36B|CCL21|IL1A|IL1B|IL7|IL11|IL9|TNFSF4|CXCL3|CXCL5|IL16|IL12A|IL13|CXCL11|CSF1|IL25|CXCL14|IL17F|CCL20|CCL28|CXCL9|IL20|TNFSF13B|CCL7|IL33|IL36RN|CXCL12|TNFSF11|FAM19A5|IL6|IL10|IL22|CCL5|CCL16|CCL4 | 77 | 325|484|485|531|202|120|432|561|558|500|322|345|266|126|295|383|356|355|385|519|418|321|149|478|468|367|308|488|283|161|326|289|346|536|181|430|327|218|425|534|135|501|118 | 3.89E-27 |  | KW-0202 | 2.396657624 | 0 |
| 712 | 75 | GO Process |  | regulation of MAPK cascade | 1.16E-24 | CX3CL1|MAP4K5|MAPK1|TGFB1|CCL1|CCL13|TGFB3|CCR7|TRAF2|LIF|MAP3K10|IRAK2|PDGFRA|CCL21|PDGFRB|TRAF4|MAP3K8|NOX4|IL1B|MAP4K3|IL11|MAP3K13|SOD1|ACKR3|PDCD4|CSF1R|TGFA|MAP2K1|MAP3K11|CFLAR|TNIP1|TNFAIP8L3|SMAD4|MAP4K4|MAP2K3|MAP3K15|CCL20|MAP3K5|MAPK10|MAP3K3|KIAA1804|TGFB2|MAPKAPK2|TAB2|IL6R|MAP3K7|IRAK1|TLR4|TGFBR1|ZAK|CCL7|TAB3|JAK2|TNFRSF19|MAP3K4|PDCD10|MAP2K7|TNFSF11|MAP3K1|IL6|CXCR4|MAP3K2|TLR6|TNIK|MAP2K4|TRAF6|MAP3K12|MAPKAPK5|MAP3K9|TNFRSF11A|MAP2K6|CCL5|CCL16|CCL4|MAP3K14 | 77 | 325|451|469|350|484|485|265|243|444|432|489|258|516|500|506|194|165|364|345|141|126|490|281|247|311|153|279|292|439|133|538|248|470|254|528|384|308|335|380|299|190|376|268|184|348|358|434|342|368|264|289|206|309|256|466|316|236|430|397|218|450|374|305|535|517|315|426|370|290|163|255|135|501|118|171 | 1.77E-26 |  | GO.0043408 | 2.393554201 | 0 |
| 964 | 87 | GO Process |  | regulation of transferase activity | 1.45E-24 | MAP4K5|MAPK1|TGFB1|CDKN1B|TGFB3|CCR7|ATG14|TRAF2|MAP3K10|IRAK2|PDGFRA|CCL21|PDGFRB|MMD|SMAD7|TRAF4|MAP3K8|NOX4|IL1B|MAP4K3|MAP3K13|CDK6|DOCK3|SOD1|TNFRSF10B|PDCD4|DEPTOR|CSF1R|TGFA|RICTOR|MAP2K1|SOCS5|MAP3K11|OXSR1|C8orf4|CSF1|TNFAIP8L3|SOCS1|SOCS3|BAG5|MAP4K4|MAP2K3|MAP3K15|TGFBR2|MAP3K5|MAPK10|MAP3K3|KIAA1804|TGFB2|MAPKAPK2|TAB2|IL6R|CACUL1|MAP3K7|MTCP1|IRAK1|BAG2|TLR4|TGFBR1|ZAK|MAP3K19|TAB3|JAK2|MAP3K4|PDCD10|SOCS4|SOCS6|MAP2K7|TNFSF11|MAP3K1|CXCR4|MAP3K2|TLR6|TNIK|MAP2K4|AMBRA1|TRAF6|MAP3K12|MAPKAPK5|MAP3K9|TNFRSF11A|MAP2K6|CCL5|SOCS2|TNFAIP3|SOCS7|MAP3K14 | 77 | 451|469|350|285|265|243|150|444|489|258|516|500|506|427|195|194|165|364|345|141|490|365|225|281|410|311|408|153|279|302|292|226|439|504|293|149|248|460|246|124|254|528|384|332|335|380|299|190|376|268|184|348|522|358|162|434|116|342|368|264|352|206|309|466|316|523|244|236|430|397|450|374|305|535|517|131|315|426|370|290|163|255|135|419|164|186|171 | 2.24E-26 |  | GO.0051338 | 2.3838632 | 0 |
| 138 | 38 | GO Process |  | stress-activated protein kinase signaling cascade | 1.52E-24 | MAP4K5|MAPK1|NFKB1|MAP3K10|IRAK2|MAP3K8|MAP4K3|MAP3K13|MAP3K11|OXSR1|MAP2K3|MAP3K5|MAPK10|MAP3K3|KIAA1804|MAPKAPK2|TAB2|MAP3K7|IRAK1|ZAK|MAP3K19|TAB3|TNFRSF19|MAPK8|MAP2K7|TNFSF11|MAP3K1|MAP3K2|IRAK4|MAPK9|TNIK|MAP2K4|IKBKB|TRAF6|MAP3K12|MAP3K9|MAP2K6|MAP3K14 | 77 | 451|469|529|489|258|165|141|490|439|504|528|335|380|299|190|268|184|358|434|264|352|206|256|282|236|430|397|374|371|250|535|517|526|315|426|290|255|171 | 2.38E-26 |  | GO.0031098 | 2.381815641 | 0 |
| 6607 | 266 | GO Function | | protein binding | 4.07E-24 | CX3CL1|TNFRSF1A|TGFBR3|MAPK1|TGFB1|CCL1|CCL13|TNFAIP1|CXCL6|NFKB1|CRTAM|CDKN1B|EXOC2|IL1RL1|BCL9|TGFB3|RHOQ|TNFSF10|TNFSF9|CCR7|ATG14|TRAF2|LIF|PIAS1|BCL2L2|DOCK7|PXDN|MAP3K10|EXOC4|XPO4|IRAK2|PDGFRA|HILPDA|DOCK10|IL36G|IL36B|CCL21|BCL2L10|TRAF5|PDGFRB|MAPK6|FAF2|SMAD7|SMAD2|RIMS2|TRAF4|SMURF2|NOX4|IL1A|IL1B|ITPKC|IL7|ATF2|IL11|STAT3|MAP3K13|PECR|PRDX5|CDK6|DOCK3|BNIP2|SOD1|IL22RA1|ACKR3|IL9|DOCK11|TNFRSF10B|DOCK5|DOCK1|TNFSF4|ATG3|CSF1R|AIFM1|BAG4|SMAD6|TGFA|TRAT1|CXCL3|CXCL5|RICTOR|IL22RA2|TNFRSF11B|DEFB1|HIF1AN|AVPR1A|HPS6|LTBP3|MAP2K1|BCL2L1|MAEA|IL16|IL12A|DEFB4A|IL13|SOCS5|ALCAM|CXCL11|MAP3K11|PPARD|OXSR1|CFLAR|IL1RAP|TNIP1|EEA1|PRKRA|C8orf4|CSF1|TGIF1|PAWR|IL25|SOCS1|SOCS3|IKZF1|SMAD3|LAMP1|BCL9L|CXCL14|IL17F|BCL11A|C1QTNF6|BAG5|SMAD4|GPR29|IFNAR2|IKZF3|MAP2K3|RBPJ|ATG7|IRF5|CCRL2|FXR1|CCL20|TGFBR2|MAP3K5|CPLX2|CCL28|SMURF1|CXCL9|NOS1AP|KIAA1804|TGFB2|TAGAP|IL20|MAPKAPK2|TAB2|PEX3|PEX19|IL6R|IKZF5|MCL1|BAG3|CACUL1|PEX11B|MAP3K7|MTCP1|SIKE1|IRAK1|BCL10|BAG2|IGSF1|LCOR|IFIT3|YWHAB|AGO1|TRAF3IP1|CXCR3|TLR4|IL2RG|TGFBR1|TNFSF13B|TNFRSF1B|DOCK9|HIF3A|BCOR|CCL7|BNIP3L|IRF4|IL6ST|IL33|JAK2|EXOSC8|ATG16L1|TANK|PDCD10|IL36RN|BCL2L11|PRC1|AKTIP|MAPK8|CXCL12|NFATC2|MAP2K7|BCL2|TNFSF11|MAP3K1|MAPK4|CMKBR6|CSF2RB|GATSL3|BCL6|PPARA|IL6|LTBP1|IL1R1|CXCR4|IL18R1|MAP3K2|NFATC4|TLR6|IRAK4|PEX5|MAPK9|CXCR6|OSGIN2|HLA-B|NCOA2|DOCK4|LAMP2|MAP2K4|BCL7B|PDCD6IP|IL10|IKZF2|TGFBI|ACKR2|PEX5L|LAT2|DEFB4B|SMAD1|CDKN2AIP|IKBKB|API5|AMBRA1|ATG13|TRAF6|DICER1|HIF1A|SMAD5|IL22|RASSF3|SOD2|APAF1|MAP3K12|MAPKAPK5|JKAMP|MAP3K9|MOAP1|TRAF3|LITAF|TXNIP|IKBKE|TNFRSF11A|PIAS2|ERC1|MAP2K6|CCL5|NOS1|CCL16|TNFRSF9|JAKMIP2|SOCS2|TNFAIP3|SOCS7|CCL4|PEX12|GATSL2 | 77 | 325|351|447|469|350|484|485|280|531|529|151|285|339|336|175|265|552|202|120|243|150|444|432|554|551|413|543|489|159|386|258|516|462|505|561|558|500|550|142|506|235|458|195|200|331|194|362|364|322|345|420|266|189|126|334|490|436|208|365|225|187|281|211|247|295|422|410|406|215|383|113|153|482|409|369|279|527|356|355|302|272|359|112|228|222|209|441|292|372|207|385|519|158|418|226|286|321|439|227|504|133|199|538|146|196|293|149|143|396|478|460|246|220|241|360|125|468|367|507|123|124|470|448|452|392|528|438|119|160|134|245|308|332|335|216|488|464|283|212|190|376|139|161|268|184|423|521|348|412|278|318|522|343|358|162|338|434|323|116|132|233|404|440|239|474|230|342|563|368|326|276|300|510|541|289|499|169|391|346|309|297|223|314|316|536|234|511|424|282|181|115|236|475|430|397|231|431|524|288|310|491|218|154|274|450|287|374|249|305|371|514|250|435|446|114|455|544|304|517|174|307|425|486|157|173|328|467|166|193|324|526|463|131|559|315|121|495|201|534|221|381|148|426|370|382|290|138|557|395|415|548|163|284|401|255|135|508|501|473|251|419|164|186|118|229|402 | 1.90E-26 |  | GO.0005515 | 2.339040559 | 0 |
| 2249 | 136 | GO Process |  | regulation of catalytic activity | 1.41E-23 | CX3CL1|MAP4K5|MAPK1|TGFB1|CCL1|CCL13|NFKB1|CDKN1B|TGFB3|TNFSF10|CCR7|ATG14|TRAF2|DOCK7|MAP3K10|IRAK2|PDGFRA|DOCK10|CCL21|BCL2L10|PDGFRB|FAF2|MMD|SMAD7|TRAF4|MAP3K8|NOX4|IL1B|IL7|MAP4K3|STAT3|MAP3K13|PRDX5|LAMP3|CDK6|DOCK3|BNIP2|SOD1|DOCK11|TNFRSF10B|PDCD4|DOCK1|DEPTOR|CSF1R|AIFM1|BAG4|TGFA|RICTOR|AVPR1A|MAP2K1|IL13|SOCS5|MAP3K11|OXSR1|CFLAR|PRKRA|C8orf4|CSF1|TNFAIP8L3|SOCS1|SOCS3|SMAD3|BAG5|MAP4K4|MAP2K3|MAP3K15|CCL20|TGFBR2|MAP3K5|MAPK10|MAP3K3|NOS1AP|KIAA1804|TGFB2|TAGAP|MAPKAPK2|TAB2|IL6R|BAG3|CACUL1|MAP3K7|MTCP1|IRAK1|TNFRSF6B|BCL10|BAG2|YWHAB|TLR4|TGFBR1|ZAK|MAP3K19|TNFRSF1B|DOCK9|CCL7|TAB3|JAK2|MAP3K4|TANK|PDCD10|BCL2L11|SOCS4|MAPK8|SOCS6|MAP2K7|BCL2|TNFSF11|MAP3K1|BCL6|CXCR4|MAP3K2|TLR6|ITK|TNIK|DOCK4|MAP2K4|TNFAIP8|IKBKB|AMBRA1|TRAF6|HIF1A|APAF1|MAP3K12|MAPKAPK5|MAP3K9|TXNIP|IKBKE|TNFRSF11A|MAP2K6|CCL5|NOS1|CCL16|SOCS2|TNFAIP3|SOCS7|CCL4|MAP3K14 | 77 | 325|451|469|350|484|485|529|285|265|202|243|150|444|413|489|258|516|505|500|550|506|458|427|195|194|165|364|345|266|141|334|490|208|377|365|225|187|281|422|410|311|215|408|153|482|409|279|302|222|292|418|226|439|504|133|196|293|149|248|460|246|241|124|254|528|384|308|332|335|380|299|212|190|376|139|268|184|348|318|522|358|162|434|481|323|116|440|342|368|264|352|276|300|289|206|309|466|314|316|234|523|282|244|236|475|430|397|310|450|374|305|556|535|544|517|185|526|131|315|495|148|426|370|290|415|548|163|255|135|508|501|419|164|186|118|171 | 2.25E-25 |  | GO.0050790 | 2.285078089 | 0 |
| 955 | 85 | GO Process |  | regulation of response to external stimulus | 1.41E-23 | CX3CL1|TNFRSF1A|TGFB1|CCL1|CXCL6|NFKB1|CRTAM|IL1RL1|CCR7|PIAS1|IRAK2|PDGFRA|CCL21|ARG2|PDGFRB|IL1B|PDCD4|TNFSF4|IL17RB|CXCL3|CXCL5|RICTOR|IL22RA2|MAP2K1|IL16|IL12A|SOCS5|CXCL11|PPARD|TNIP1|CSF1|IFNLR1|SOCS1|SOCS3|CCR4|SMAD3|LAMP1|CXCL14|IL17F|GPR29|IFNAR2|TGFBR2|CXCL9|IL20|MAPKAPK2|TAB2|IL6R|MAP3K7|IRAK1|BCL10|TRAF3IP1|CXCR3|TLR4|TNFRSF1B|CCL7|TAB3|IRF4|IL6ST|IL33|JAK2|TANK|CXCL12|TNFSF11|MAP3K1|CMKBR6|BCL6|PPARA|IL6|IL1R1|CXCR4|TLR6|IRAK4|MAPKBP1|HLA-B|IL10|TICAM2|IKBKB|TRAF6|TRAF3|IKBKE|TNFRSF11A|MAP2K6|CCL5|TNFAIP3|CCL4 | 77 | 325|351|350|484|531|529|151|336|243|554|258|516|500|518|506|345|311|383|497|356|355|302|272|292|385|519|226|321|227|538|149|341|460|246|502|241|360|468|367|448|452|332|283|161|268|184|348|358|434|323|474|230|342|276|289|206|169|391|346|309|314|181|430|397|431|310|491|218|274|450|305|371|298|114|425|329|526|315|557|548|163|255|135|164|118 | 2.24E-25 |  | GO.0032101 | 2.285078089 | 0 |
| 1553 | 110 | GO Process |  | cellular response to stress | 1.76E-23 | MAP4K5|TNFRSF1A|MAPK1|NFKB1|CDKN1B|EEPD1|TMX4|ATG14|TRAF2|BCL2L2|MAP3K10|IRAK2|PDGFRA|HILPDA|BCL2L10|FAF2|WDR59|MAP3K8|NOX4|IL1A|MAP4K3|ATF2|MAP3K13|PRDX5|SOD1|TNFRSF10B|ATG10|TXNDC11|AIFM1|WDR24|CREBRF|HIF1AN|AVPR1A|TMX3|MAP2K1|BCL2L1|CTLA4|SOCS5|MAP3K11|PPARD|OXSR1|CFLAR|PRKRA|C8orf4|AEN|MIOS|MAP2K3|RBPJ|ATG7|MAP3K5|MAPK10|SMURF1|MAP3K3|KIAA1804|MAPKAPK2|TAB2|MCL1|ATG5|BAG3|MAP3K7|IRAK1|TXNDC8|ZAK|MAP3K19|TNFRSF1B|HIF3A|TAB3|TXNDC5|BNIP3L|JAK2|TNFRSF19|TANK|PDCD10|BCL2L11|MAPK8|NFATC2|MAP2K7|BCL2|TNFSF11|MAP3K1|TXNRD2|BCL6|IL6|MAP3K2|NFATC4|IRAK4|TMX1|MAPK9|TNIK|OXR1|LAMP2|MAP2K4|CDKN2AIP|IKBKB|AMBRA1|ATG13|TRAF6|BCLAF1|HIF1A|SOD2|APAF1|MAP3K12|MAPKAPK5|JKAMP|MAP3K9|MOAP1|IKBKE|MAP2K6|TNFAIP3|MAP3K14 | 77 | 451|351|469|529|285|197|301|150|444|551|489|258|516|462|550|458|127|165|364|322|141|189|490|208|281|410|155|156|482|260|296|228|222|122|292|372|183|226|439|227|504|133|196|293|547|525|528|438|119|335|380|464|299|190|268|184|278|312|318|358|434|238|264|352|276|510|206|459|499|309|256|314|316|234|282|115|236|475|430|397|476|310|218|374|249|371|390|250|535|144|304|517|324|526|131|559|315|180|495|381|148|426|370|382|290|138|548|255|164|171 | 2.85E-25 |  | GO.0033554 | 2.275448733 | 0 |
| 530 | 64 | UniProt Keywords | | Apoptosis | 2.17E-23 | RELT|TNFRSF1A|MAPK1|NFKB1|TNFSF10|TRAF2|BCL2L2|BCL2L10|TRAF5|TRAF4|CCAR1|BNIP2|TNFRSF10B|PDCD4|DOCK1|AIFM1|TNFRSF21|TNFRSF11B|BCL2L1|AVEN|ATG4D|CFLAR|C8orf4|TNFRSF12A|PAWR|AEN|PDCD1|AREL1|TGFBR2|MAP3K5|MCL1|ATG5|BAG3|MAP3K7|BCL10|IFIT2|CXCR3|TGFBR1|TNFRSF1B|HIF3A|BCAP29|CAAP1|BNIP3L|TNFRSF19|PDCD10|BCL2L11|BCL2L15|CIAPIN1|AKTIP|MAP2K7|BCL2|MAP2K4|BCL7B|PDCD6IP|TNFAIP8|API5|APAF1|MAP3K9|MOAP1|TRAF3|MAP2K6|ECSCR|PANO|TNFAIP3 | 77 | 532|351|469|529|202|444|551|550|142|194|111|187|410|311|215|482|513|359|372|145|213|133|293|340|396|547|537|437|332|335|278|312|318|358|323|394|230|368|276|510|129|428|499|256|316|234|320|472|424|236|475|517|174|307|185|463|148|290|138|557|255|253|172|164 | 1.56E-25 |  | KW-0053 | 2.266354027 | 0 |
| 859 | 80 | GO Process |  | negative regulation of apoptotic process | 3.04E-23 | CX3CL1|NFKB1|CDKN1B|TGFB3|TNFSF10|CCR7|TRAF2|PIAS1|BCL2L2|CCL21|IER3|BCL2L10|ARG2|PDGFRB|IL1A|IL1B|IL7|STAT3|PRDX5|LAMP3|BNIP2|SOD1|ACKR3|TNFRSF10B|PDCD4|CSF1R|BAG4|SMAD6|BCL2L1|MAEA|IL13|IL7R|AVEN|PPARD|CFLAR|C8orf4|SOCS3|SMAD3|PDCD1|BAG5|MAP4K4|AREL1|BCL11B|MCL1|ATG5|BAG3|IRAK1|TNFRSF6B|BCL10|IFIT3|AGO4|CXCR3|TGFBR1|TNFRSF1B|TXNDC5|BNIP3L|IL6ST|JAK2|PDCD10|CIAPIN1|MAPK8|CXCL12|BCL2|BCL6|IL6|OXR1|MAP2K4|IL10|TNFAIP8|IKBKB|API5|AMBRA1|TRAF6|HIF1A|SOD2|MAP3K12|ERC1|CCL5|SOCS2|TNFAIP3 | 77 | 325|529|285|265|202|243|444|554|551|500|461|550|518|506|322|345|266|334|208|377|187|281|247|410|311|153|409|369|372|207|418|176|145|227|133|293|246|241|537|124|254|437|128|278|312|318|434|481|323|404|240|230|368|276|459|499|391|309|316|472|282|181|475|310|218|144|517|425|185|526|463|131|315|495|381|426|401|135|419|164 | 4.99E-25 |  | GO.0043066 | 2.251712642 | 0 |
| 499 | 61 | GO Process |  | positive regulation of response to external stimulus | 7.87E-23 | CX3CL1|TNFRSF1A|TGFB1|CCL1|CXCL6|NFKB1|CRTAM|IL1RL1|CCR7|IRAK2|CCL21|PDGFRB|IL1B|PDCD4|TNFSF4|IL17RB|CXCL3|CXCL5|IL16|IL12A|CXCL11|TNIP1|CSF1|CCR4|SMAD3|LAMP1|CXCL14|IL17F|GPR29|CXCL9|MAPKAPK2|TAB2|IL6R|MAP3K7|IRAK1|BCL10|CXCR3|TLR4|CCL7|TAB3|IL6ST|IL33|JAK2|TANK|CXCL12|TNFSF11|MAP3K1|CMKBR6|IL6|TLR6|IRAK4|TICAM2|IKBKB|TRAF6|TRAF3|IKBKE|TNFRSF11A|MAP2K6|CCL5|TNFAIP3|CCL4 | 77 | 325|351|350|484|531|529|151|336|243|258|500|506|345|311|383|497|356|355|385|519|321|538|149|502|241|360|468|367|448|283|268|184|348|358|434|323|230|342|289|206|391|346|309|314|181|430|397|431|218|305|371|329|526|315|557|548|163|255|135|164|118 | 1.32E-24 |  | GO.0032103 | 2.210402527 | 0 |
| 337 | 51 | GO Process |  | regulation of MAP kinase activity | 1.28E-22 | MAP4K5|MAPK1|TGFB1|TGFB3|TRAF2|MAP3K10|IRAK2|PDGFRB|MAP3K8|NOX4|IL1B|MAP4K3|MAP3K13|SOD1|PDCD4|TGFA|MAP2K1|MAP3K11|MAP4K4|MAP2K3|MAP3K15|MAP3K5|MAPK10|MAP3K3|KIAA1804|MAPKAPK2|TAB2|MAP3K7|IRAK1|TLR4|TGFBR1|ZAK|TAB3|JAK2|MAP3K4|PDCD10|MAP2K7|TNFSF11|MAP3K1|CXCR4|MAP3K2|TLR6|TNIK|MAP2K4|TRAF6|MAP3K12|MAPKAPK5|MAP3K9|TNFRSF11A|MAP2K6|MAP3K14 | 77 | 451|469|350|265|444|489|258|506|165|364|345|141|490|281|311|279|292|439|254|528|384|335|380|299|190|268|184|358|434|342|368|264|206|309|466|316|236|430|397|450|374|305|535|517|315|426|370|290|163|255|171 | 2.18E-24 |  | GO.0043405 | 2.189279003 | 0 |
| 293 | 48 | KEGG Pathways | | MAPK signaling pathway | 1.69E-22 | TNFRSF1A|MAPK1|TGFB1|NFKB1|TGFB3|TRAF2|PDGFRA|PDGFRB|MAP3K8|IL1A|IL1B|MAP4K3|ATF2|MAP3K13|CSF1R|TGFA|NFATC3|MAP2K1|MAP3K11|CSF1|MAP4K4|MAP2K3|TGFBR2|MAP3K5|MAPK10|MAP3K3|TGFB2|MAPKAPK2|TAB2|MAP3K7|IRAK1|TGFBR1|ZAK|MAP3K4|MAPK8|MAP2K7|MAP3K1|IL1R1|MAP3K2|IRAK4|MAPK9|MAP2K4|IKBKB|TRAF6|MAP3K12|MAPKAPK5|MAP2K6|MAP3K14 | 77 | 351|469|350|529|265|444|516|506|165|322|345|141|189|490|153|279|210|292|439|149|254|528|332|335|380|299|376|268|184|358|434|368|264|466|282|236|397|274|374|371|250|517|526|315|426|370|255|171 | 2.57E-24 |  | hsa04010 | 2.17721133 | 0 |
| 4953 | 217 | GO Process |  | negative regulation of biological process | 1.80E-22 | CX3CL1|TNFRSF1A|TGFBR3|MAPK1|TGFB1|TNFAIP1|NFKB1|CDKN1B|IL1RL1|SOGA1|TGFB3|RHOQ|TNFSF10|CCR7|ATG14|TRAF2|LIF|PIAS1|BCL2L2|PXDN|MAP3K10|IRAK2|PDGFRA|TBRG4|CCL21|IER3|BCL2L10|ARG2|PDGFRB|MSR1|SMAD7|SMAD2|SMURF2|NOX4|IL1A|IL1B|IL7|ATF2|IL11|STAT3|PRDX5|LAMP3|CDK6|CCAR1|BNIP2|IRF8|SOD1|ACKR3|TNFRSF10B|DOCK5|PDCD4|TNFSF4|ATG3|DEPTOR|CSF1R|BAG4|SMAD6|TRAT1|SNIP1|TNFRSF21|CREBRF|IL22RA2|TNFRSF11B|HIF1AN|AVPR1A|NFATC3|MAP2K1|BCL2L1|MAEA|IL12A|CTLA4|IL13|SOCS5|IL7R|AVEN|PPARD|OXSR1|CFLAR|TNIP1|PRKRA|C8orf4|IFNLR1|TGIF1|PAWR|SOCS1|PIWIL3|SOCS3|IKZF1|SMAD3|PDCD1|BCL9L|CXCL14|IL17F|BCL11A|BAG5|SMAD4|LAMTOR4|MAP4K4|RBPJ|ATG7|AREL1|PEX2|BCL11B|FXR1|TGFBR2|CCL28|SMURF1|MAP3K3|TGFB2|IRF6|MAPKAPK2|TAB2|IL6R|IKZF5|MCL1|ATG5|BAG3|IRAK1|TNFRSF6B|BCL10|BAG2|EXOSC1|IGSF1|LCOR|IFIT3|YWHAB|AGO3|AGO1|AGO4|TRAF3IP1|CXCR3|TGIF2|TLR4|TGFBR1|ZAK|TNFRSF1B|HIF3A|BCOR|TAB3|TXNDC5|BNIP3L|NFIB|IRF4|IL6ST|IL33|JAK2|LOXL2|EXOSC8|TANK|PDCD10|IL36RN|CIAPIN1|SOCS4|ULK2|MAPK8|CXCL12|NFATC2|PDCD1LG2|SOCS6|BCL2|GATSL3|BCL6|PPARA|IL6|LTBP1|NFATC4|TLR6|PEX5|MAPKBP1|OSGIN2|HLA-B|NCOA2|ILF3|DOCK4|OXR1|LAMP2|TBRG1|MAP2K4|IL10|IKZF2|TICAM2|TGFBI|SMAD1|CDKN2AIP|TNFAIP8|IKBKB|API5|AMBRA1|TRAF6|BCLAF1|DICER1|HIF1A|SMAD5|NKRF|TNFAIP8L1|BCL7A|SOD2|APAF1|MAP3K12|MAPKAPK5|TRAF3|LITAF|TXNIP|IKBKE|PIAS2|ERC1|MAP2K6|CCL5|NOS1|TNFRSF9|ECSCR|PANO|SOCS2|TNFAIP3|SOCS7|CCL4|GATSL2 | 77 | 325|351|447|469|350|280|529|285|336|317|265|552|202|243|150|444|432|554|551|543|489|258|516|496|500|461|550|518|506|147|195|200|362|364|322|345|266|189|126|334|208|377|365|111|187|398|281|247|410|406|311|383|113|408|153|409|369|527|337|513|296|272|359|228|222|210|292|372|207|519|183|418|226|176|145|227|504|133|538|196|293|341|143|396|460|182|246|220|241|537|125|468|367|507|124|470|188|254|438|119|437|269|128|245|332|488|464|299|376|487|268|184|348|412|278|312|318|434|481|323|116|137|132|233|404|440|520|239|240|474|230|259|342|368|264|276|510|541|206|459|499|170|169|391|346|309|480|297|314|316|536|472|523|214|282|181|115|373|244|475|288|310|491|218|154|249|305|514|298|446|114|455|110|544|144|304|445|517|425|486|329|157|193|324|185|526|463|131|315|180|121|495|201|291|453|303|381|148|426|370|557|395|415|548|284|401|255|135|508|473|253|172|419|164|186|118|402 | 3.10E-24 |  | GO.0048519 | 2.174472749 | 0 |
| 953 | 83 | GO Process |  | negative regulation of cell death | 1.98E-22 | CX3CL1|NFKB1|CDKN1B|TGFB3|TNFSF10|CCR7|TRAF2|PIAS1|BCL2L2|CCL21|IER3|BCL2L10|ARG2|PDGFRB|IL1A|IL1B|IL7|STAT3|PRDX5|LAMP3|BNIP2|SOD1|ACKR3|TNFRSF10B|PDCD4|CSF1R|BAG4|SMAD6|BCL2L1|MAEA|IL13|IL7R|AVEN|PPARD|CFLAR|C8orf4|SOCS3|SMAD3|PDCD1|BAG5|SMAD4|MAP4K4|ATG7|AREL1|BCL11B|MCL1|ATG5|BAG3|IRAK1|TNFRSF6B|BCL10|IFIT3|AGO4|CXCR3|TGFBR1|TNFRSF1B|TXNDC5|BNIP3L|IL6ST|JAK2|PDCD10|CIAPIN1|MAPK8|CXCL12|BCL2|BCL6|PPARA|IL6|OXR1|MAP2K4|IL10|TNFAIP8|IKBKB|API5|AMBRA1|TRAF6|HIF1A|SOD2|MAP3K12|ERC1|CCL5|SOCS2|TNFAIP3 | 77 | 325|529|285|265|202|243|444|554|551|500|461|550|518|506|322|345|266|334|208|377|187|281|247|410|311|153|409|369|372|207|418|176|145|227|133|293|246|241|537|124|470|254|119|437|128|278|312|318|434|481|323|404|240|230|368|276|459|499|391|309|316|472|282|181|475|310|491|218|144|517|425|185|526|463|131|315|495|381|426|401|135|419|164 | 3.43E-24 |  | GO.0060548 | 2.170333481 | 0 |
| 6082 | 247 | GO Process |  | regulation of cellular metabolic process | 2.37E-22 | CX3CL1|MAP4K5|TNFRSF1A|TGFBR3|MAPK1|TGFB1|CCL1|CCL13|TNFAIP1|NFKB1|CDKN1B|BCL9|SOGA1|TGFB3|RHOQ|TNFSF10|RASL11A|CCR7|ATG14|TRAF2|LIF|PIAS1|DOCK7|MAP3K10|EXOC4|IRAK2|PDGFRA|TBRG4|CCL21|BCL2L10|TRAF5|ARG2|PDGFRB|MMD|SMAD7|SMAD2|TRAF4|SMURF2|MAP3K8|NOX4|IL1A|IL1B|MAP4K3|ATF2|IL11|STAT3|MAP3K13|PRDX5|LAMP3|CDK6|CCAR1|DOCK3|IRF8|SOD1|ACKR3|IL9|TNFRSF10B|PDCD4|TNFSF4|ATG10|DEPTOR|CSF1R|AIFM1|BAG4|GATAD1|SMAD6|TNFRSF13C|WDR24|TGFA|TRAT1|SNIP1|RICTOR|CREBRF|IL22RA2|HIF1AN|AVPR1A|NFATC3|MAP2K1|BCL2L1|MAEA|IL16|IL12A|IL13|SOCS5|IL7R|MAP3K11|PPARD|OXSR1|CFLAR|IL1RAP|TNIP1|C8orf4|NFATC2IP|CSF1|TGIF1|TNFAIP8L3|PAWR|IL25|SOCS1|PIWIL3|SOCS3|IKZF1|SMAD3|BCL9L|IL17F|BCL11A|BAG5|SMAD4|LAMTOR4|MAP4K4|IKZF3|MAP2K3|RBPJ|MAP3K15|ATG7|PEX2|BCL11B|IRF5|FXR1|CCL20|TGFBR2|MAP3K5|MAPK10|SMURF1|MAP3K3|ILF2|NOS1AP|IRF2BP2|KIAA1804|TGFB2|IRF6|IL20|MAPKAPK2|TAB2|HIVEP2|IL6R|GATAD2B|IKZF5|MCL1|ATG5|BAG3|CACUL1|MAP3K7|MTCP1|IRAK1BP1|IRAK1|TNFRSF6B|BCL10|BAG2|EXOSC1|IGSF1|LCOR|YWHAB|AGO3|AGO1|AGO4|TRAF3IP1|CXCR3|TGIF2|TLR4|TGFBR1|ZAK|MAP3K19|TNFRSF1B|HIF3A|BCOR|CCL7|TAB3|BNIP3L|NFIB|IRF4|IL6ST|IL33|JAK2|TNFRSF19|LOXL2|EXOSC8|MAP3K4|TANK|PDCD10|BCL2L11|AKTIP|SOCS4|MAPK8|NFATC2|IRF9|SOCS6|MAP2K7|BCL2|TNFSF11|MAP3K1|BCL6|PPARA|IL6|CXCR4|IL18R1|MAP3K2|NFATC4|TLR6|IRAK4|EIF4E3|MAPK9|TNIK|NCOA2|ILF3|OXR1|MAP2K4|IL10|IKZF2|SMAD1|TNFAIP8|IKBKB|AMBRA1|ATG13|TRAF6|BCLAF1|DICER1|BCORL1|HIF1A|SMAD5|NKRF|TET2|BCL7A|SOD2|APAF1|MAP3K12|MAPKAPK5|MAP3K9|TRAF3|NFAT5|LITAF|TXNIP|TNFRSF11A|IER2|PIAS2|ERC1|MAP2K6|CCL5|NOS1|CCL16|ECSCR|PANO|SOCS2|TNFAIP3|SOCS7|CCL4|MAP3K14 | 77 | 325|451|351|447|469|350|484|485|280|529|285|175|317|265|552|202|205|243|150|444|432|554|413|489|159|258|516|496|500|550|142|518|506|427|195|200|194|362|165|364|322|345|141|189|126|334|490|208|377|365|111|225|398|281|247|295|410|311|383|155|408|153|482|409|530|369|224|260|279|527|337|302|296|272|228|222|210|292|372|207|385|519|418|226|176|439|227|504|133|199|538|293|347|149|143|248|396|478|460|182|246|220|241|125|367|507|124|470|188|254|392|528|438|384|119|269|128|160|245|308|332|335|380|464|299|443|212|178|190|376|487|161|268|184|433|348|414|412|278|312|318|522|358|162|400|434|481|323|116|137|132|233|440|520|239|240|474|230|259|342|368|264|352|276|510|541|289|206|499|170|169|391|346|309|256|480|297|466|314|316|234|424|523|282|115|492|244|236|475|430|397|310|491|218|450|287|374|249|305|371|403|250|535|455|110|144|517|425|486|193|185|526|131|559|315|180|121|387|495|201|291|294|303|381|148|426|370|290|557|393|395|415|163|354|284|401|255|135|508|501|253|172|419|164|186|118|171 | 4.16E-24 |  | GO.0031323 | 2.162525165 | 0 |
| 92 | 31 | KEGG Pathways | | IL-17 signaling pathway | 3.51E-22 | MAPK1|CXCL6|NFKB1|TRAF2|TRAF5|MAPK6|TRAF4|IL1B|IL17RB|CXCL3|CXCL5|IL13|IL25|IL17F|CCL20|MAPK10|TAB2|MAP3K7|CCL7|TAB3|IL17RE|MAPK8|MAPK4|IL6|MAPK9|DEFB4B|IKBKB|TRAF6|TRAF3|IKBKE|TNFAIP3 | 77 | 469|531|529|444|142|235|194|345|497|356|355|418|478|367|308|380|184|358|289|206|465|282|231|218|250|166|526|315|557|548|164 | 7.12E-24 |  | hsa04657 | 2.145469288 | 0 |
| 93 | 31 | KEGG Pathways | | NF-kappa B signaling pathway | 3.66E-22 | TNFRSF1A|CCL13|NFKB1|TRAF2|CCL21|TRAF5|IL1B|TNFRSF13C|BCL2L1|CFLAR|TAB2|MAP3K7|IRAK1|BCL10|TLR4|TNFSF13B|TAB3|CXCL12|BCL2|TNFSF11|IL1R1|IRAK4|TICAM2|IKBKB|TRAF6|TRAF3|TNFRSF11A|ERC1|TNFAIP3|CCL4|MAP3K14 | 77 | 351|485|529|444|500|142|345|224|372|133|184|358|434|323|342|326|206|181|475|430|274|371|329|526|315|557|163|401|164|118|171 | 9.30E-24 |  | hsa04064 | 2.143651891 | 0 |
| 615 | 66 | GO Process |  | regulation of cytokine production | 5.77E-22 | CX3CL1|TGFB1|NFKB1|CRTAM|IL1RL1|TGFB3|CCR7|TRAF2|HILPDA|ARG2|SMAD7|IL1A|IL1B|IL7|ATF2|IRF8|SOD1|IL9|PDCD4|TNFSF4|CSF1R|IL17RB|TNFRSF13C|TNFRSF21|IL12A|IL13|SOCS5|IL1RAP|SMAD3|IL17F|SMAD4|MAP2K3|IRF5|CCL20|TGFB2|MAPKAPK2|IL6R|ATG5|MAP3K7|IRAK1|BCL10|TRAF3IP1|TLR4|IRF4|IL6ST|IL33|JAK2|IL36RN|PDCD1LG2|BCL6|IL6|IL1R1|IL18R1|NFATC4|TLR6|MAPKBP1|HLA-B|IL10|TICAM2|TRAF6|HIF1A|TRAF3|LITAF|IKBKE|TNFRSF9|TNFAIP3 | 77 | 325|350|529|151|336|265|243|444|462|518|195|322|345|266|189|398|281|295|311|383|153|497|224|513|519|418|226|199|241|367|470|528|160|308|376|268|348|312|358|434|323|474|342|169|391|346|309|536|373|310|218|274|287|249|305|298|114|425|329|315|495|557|395|548|473|164 | 1.02E-23 |  | GO.0001817 | 2.123882419 | 0 |
| 160 | 37 | KEGG Pathways | | Jak-STAT signaling pathway | 6.66E-22 | LIF|PIAS1|PDGFRA|PDGFRB|IL7|IL11|STAT3|IL22RA1|IL9|IL22RA2|BCL2L1|IL12A|IL13|SOCS5|IL7R|IFNLR1|SOCS1|SOCS3|IL21R|IFNAR2|IL20|IL6R|MCL1|IL2RG|IL6ST|JAK2|SOCS4|IRF9|SOCS6|BCL2|CSF2RB|IL6|IL10|IL22|PIAS2|SOCS2|SOCS7 | 77 | 432|554|516|506|266|126|334|211|295|272|372|519|418|226|176|341|460|246|217|452|161|348|278|563|391|309|523|492|244|475|524|218|425|534|284|419|186 | 2.03E-23 |  | hsa04630 | 2.117652577 | 0 |
| 91 | 31 | GO Function | | cytokine receptor activity | 8.74E-22 | IL1RL1|CCR7|IL22RA1|ACKR3|IL17RB|CXCR5|IL17RD|IL22RA2|IL7R|IL1RAP|IFNLR1|CCR4|IL21R|GPR29|IFNAR2|CCRL2|CCR9|IL6R|IL1RAPL2|CXCR3|IL2RG|IL6ST|IL17RE|IL17REL|CMKBR6|CSF2RB|IL1R1|CXCR4|IL18R1|CXCR6|ACKR2 | 77 | 336|243|211|247|497|306|136|272|176|199|341|502|217|448|452|134|456|348|252|230|563|391|465|479|431|524|274|450|287|435|173 | 5.44E-24 |  | GO.0004896 | 2.105848857 | 0 |
| 107 | 32 | GO Process |  | stress-activated MAPK cascade | 1.55E-21 | MAP4K5|MAPK1|NFKB1|MAP3K10|IRAK2|MAP3K8|MAP4K3|MAP3K13|MAP3K11|MAP2K3|MAP3K5|MAPK10|KIAA1804|MAPKAPK2|TAB2|MAP3K7|IRAK1|ZAK|TAB3|TNFRSF19|MAPK8|MAP2K7|TNFSF11|MAP3K2|IRAK4|MAPK9|TNIK|MAP2K4|IKBKB|TRAF6|MAP3K12|MAP3K9 | 77 | 451|469|529|489|258|165|141|490|439|528|335|380|190|268|184|358|434|264|206|256|282|236|430|374|371|250|535|517|526|315|426|290 | 2.78E-23 |  | GO.0051403 | 2.08096683 | 0 |
| 923 | 80 | GO Process |  | protein phosphorylation | 1.93E-21 | MAP4K5|TGFBR3|MAPK1|TGFB1|NFKB1|CDKN1B|LIF|MAP3K10|IRAK2|PDGFRA|TBRG4|PDGFRB|MAPK6|MMD|SMAD7|SMAD2|MAP3K8|IL1B|MAP4K3|MAP3K13|CDK6|CSF1R|IL17RD|RICTOR|MAP2K1|MAP3K11|OXSR1|PRKRA|AATK|MAP4K4|MAP2K3|MAP3K15|TGFBR2|MAP3K5|MAPK10|MAP3K3|KIAA1804|TGFB2|MAPKAPK2|TAB2|MAP3K7|IRAK1|YWHAB|TLR4|IL2RG|TGFBR1|ZAK|MAP3K19|TAB3|JAK2|TNFRSF19|MAP3K4|ULK2|MAPK8|MAP2K7|BCL2|TNFSF11|MAP3K1|MAPK4|CSF2RB|LTBP1|MAP3K2|IRAK4|MAPK9|ITK|TNIK|ILF3|MAP2K4|SMAD1|IKBKB|TRAF6|SMAD5|MAP3K12|MAPKAPK5|MAP3K9|IKBKE|ERC1|MAP2K6|CCL5|MAP3K14 | 77 | 451|447|469|350|529|285|432|489|258|516|496|506|235|427|195|200|165|345|141|490|365|153|136|302|292|439|504|196|560|254|528|384|332|335|380|299|190|376|268|184|358|434|440|342|563|368|264|352|206|309|256|466|214|282|236|475|430|397|231|524|154|374|371|250|556|535|110|517|193|526|315|201|426|370|290|548|401|255|135|171 | 3.50E-23 |  | GO.0006468 | 2.071444269 | 0 |
| 102 | 31 | KEGG Pathways | | Toll-like receptor signaling pathway | 2.58E-21 | MAPK1|NFKB1|MAP3K8|IL1B|MAP2K1|IL12A|CXCL11|IFNAR2|MAP2K3|IRF5|MAPK10|CXCL9|TAB2|MAP3K7|IRAK1|TLR4|MAPK8|MAP2K7|IL6|TLR6|IRAK4|MAPK9|MAP2K4|TICAM2|IKBKB|TRAF6|TRAF3|IKBKE|MAP2K6|CCL5|CCL4 | 77 | 469|529|165|345|292|519|321|452|528|160|380|283|184|358|434|342|282|236|218|305|371|250|517|329|526|315|557|548|255|135|118 | 9.16E-23 |  | hsa04620 | 2.058838029 | 0 |
| 124 | 33 | KEGG Pathways | | Osteoclast differentiation | 3.07E-21 | TNFRSF1A|MAPK1|TGFB1|NFKB1|TRAF2|LILRA1|IL1A|IL1B|CSF1R|TNFRSF11B|MAP2K1|CSF1|SOCS1|SOCS3|IFNAR2|TGFBR2|MAPK10|TGFB2|TAB2|MAP3K7|TGFBR1|MAPK8|NFATC2|IRF9|MAP2K7|TNFSF11|IL1R1|MAPK9|IKBKB|TRAF6|TNFRSF11A|MAP2K6|MAP3K14 | 77 | 351|469|350|529|444|344|322|345|153|359|292|149|460|246|452|332|380|376|184|358|368|282|115|492|236|430|274|250|526|315|163|255|171 | 1.25E-22 |  | hsa04380 | 2.051286162 | 0 |
| 99 | 31 | GO Function | | cytokine binding | 5.61E-21 | TNFRSF1A|TGFBR3|IL1RL1|TGFB3|CCR7|PXDN|IL22RA1|ACKR3|CSF1R|IL22RA2|LTBP3|IL12A|IL17F|GPR29|IFNAR2|TGFBR2|IL6R|CXCR3|IL2RG|TGFBR1|IL6ST|IL36RN|CMKBR6|LTBP1|IL1R1|CXCR4|IL18R1|CXCR6|ACKR2|TNFRSF11A|TNFRSF9 | 77 | 351|447|336|265|243|543|211|247|153|272|441|519|367|448|452|332|348|230|563|368|391|536|431|154|274|450|287|435|173|163|473 | 4.37E-23 |  | GO.0019955 | 2.025103714 | 0 |
| 4454 | 199 | GO Process |  | negative regulation of cellular process | 7.46E-21 | CX3CL1|TNFRSF1A|TGFBR3|MAPK1|TGFB1|TNFAIP1|NFKB1|CDKN1B|IL1RL1|SOGA1|TGFB3|TNFSF10|CCR7|ATG14|TRAF2|LIF|PIAS1|BCL2L2|PXDN|MAP3K10|PDGFRA|TBRG4|CCL21|IER3|BCL2L10|ARG2|PDGFRB|SMAD7|SMAD2|SMURF2|NOX4|IL1A|IL1B|IL7|ATF2|IL11|STAT3|PRDX5|LAMP3|CDK6|CCAR1|BNIP2|IRF8|SOD1|ACKR3|TNFRSF10B|PDCD4|TNFSF4|ATG3|DEPTOR|CSF1R|BAG4|SMAD6|TRAT1|TNFRSF21|CREBRF|HIF1AN|AVPR1A|NFATC3|MAP2K1|BCL2L1|MAEA|IL12A|CTLA4|IL13|SOCS5|IL7R|AVEN|PPARD|OXSR1|CFLAR|TNIP1|PRKRA|C8orf4|IFNLR1|TGIF1|PAWR|SOCS1|SOCS3|IKZF1|SMAD3|PDCD1|BCL9L|CXCL14|BCL11A|BAG5|SMAD4|LAMTOR4|MAP4K4|RBPJ|ATG7|AREL1|PEX2|BCL11B|FXR1|TGFBR2|CCL28|SMURF1|MAP3K3|TGFB2|IRF6|MAPKAPK2|TAB2|IKZF5|MCL1|ATG5|BAG3|IRAK1|TNFRSF6B|BCL10|BAG2|IGSF1|LCOR|IFIT3|YWHAB|AGO3|AGO1|AGO4|TRAF3IP1|CXCR3|TGIF2|TLR4|TGFBR1|ZAK|TNFRSF1B|HIF3A|BCOR|TAB3|TXNDC5|BNIP3L|NFIB|IRF4|IL6ST|IL33|JAK2|LOXL2|TANK|PDCD10|IL36RN|CIAPIN1|SOCS4|ULK2|MAPK8|CXCL12|NFATC2|PDCD1LG2|SOCS6|BCL2|GATSL3|BCL6|PPARA|IL6|LTBP1|NFATC4|TLR6|PEX5|MAPKBP1|OSGIN2|NCOA2|ILF3|OXR1|LAMP2|TBRG1|MAP2K4|IL10|IKZF2|TICAM2|TGFBI|SMAD1|CDKN2AIP|TNFAIP8|IKBKB|API5|AMBRA1|TRAF6|BCLAF1|DICER1|HIF1A|SMAD5|NKRF|TNFAIP8L1|BCL7A|SOD2|APAF1|MAP3K12|MAPKAPK5|LITAF|TXNIP|PIAS2|ERC1|MAP2K6|CCL5|NOS1|TNFRSF9|PANO|SOCS2|TNFAIP3|SOCS7|GATSL2 | 77 | 325|351|447|469|350|280|529|285|336|317|265|202|243|150|444|432|554|551|543|489|516|496|500|461|550|518|506|195|200|362|364|322|345|266|189|126|334|208|377|365|111|187|398|281|247|410|311|383|113|408|153|409|369|527|513|296|228|222|210|292|372|207|519|183|418|226|176|145|227|504|133|538|196|293|341|143|396|460|246|220|241|537|125|468|507|124|470|188|254|438|119|437|269|128|245|332|488|464|299|376|487|268|184|412|278|312|318|434|481|323|116|132|233|404|440|520|239|240|474|230|259|342|368|264|276|510|541|206|459|499|170|169|391|346|309|480|314|316|536|472|523|214|282|181|115|373|244|475|288|310|491|218|154|249|305|514|298|446|455|110|144|304|445|517|425|486|329|157|193|324|185|526|463|131|315|180|121|495|201|291|453|303|381|148|426|370|395|415|284|401|255|135|508|473|172|419|164|186|402 | 1.37E-22 |  | GO.0048523 | 2.012726117 | 0 |
| 2514 | 139 | GO Process |  | multi-organism process | 1.05E-20 | CX3CL1|RELT|TNFRSF1A|MAPK1|TGFB1|CCL1|CCL13|CXCL6|NFKB1|TGFB3|CCR7|LIF|PIAS1|BCL2L2|IRAK2|PDGFRA|IL36G|IL36B|CCL21|BCL2L10|ARG2|LOXL1|IL1B|STAT3|CDK6|IRF8|SOD1|IL22RA1|ACKR3|TNFRSF10B|PDCD4|TNFSF4|CSF1R|PEX13|CXCL3|CXCL5|TNFRSF21|TNFRSF11B|DEFB1|AVPR1A|BCL2L1|IL16|IL12A|DEFB4A|IL13|CXCL11|PPARD|PAAF1|CFLAR|IL1RAP|TNIP1|EEA1|PRKRA|CSF1|IFNLR1|IL25|PIWIL3|CCR4|SMAD3|LAMP1|CXCL14|SMAD4|GPR29|IFNAR2|RBPJ|ATG7|IRF5|CCL20|TGFBR2|MAP3K5|CCL28|CXCL9|IRF6|MAPKAPK2|IL6R|ATG5|MAP3K7|IRAK1|TNFRSF6B|BCL10|IFI44|IFI44L|IFIT3|IFIT2|YWHAB|AGO4|TLR4|IL2RG|TXNDC8|HLA-DQB1|TGFBR1|TNFRSF1B|CCL7|BNIP3L|IRF4|IL6ST|IL33|JAK2|TXNRD3|LYST|ATG16L1|TANK|IL36RN|BCL2L11|ULK2|MAPK8|CXCL12|IRF9|BCL2|CMKBR6|CSF2RB|BCL6|IL6|CXCR4|TLR6|IRAK4|CXCR6|ITK|HLA-B|ILF3|PDCD6IP|IL10|TICAM2|DEFB4B|SMAD1|IKBKB|TRAF6|SMAD5|TRAF3|LITAF|IKBKE|TNFRSF11A|CCL5|MR1|CCL16|TNFRSF9|TNFAIP3|CCL4|MAP3K14 | 77 | 325|532|351|469|350|484|485|531|529|265|243|432|554|551|258|516|561|558|500|550|518|498|345|334|365|398|281|211|247|410|311|383|153|152|356|355|513|359|112|222|372|385|519|158|418|321|227|411|133|199|538|146|196|149|341|478|182|502|241|360|468|470|448|452|438|119|160|308|332|335|488|283|487|268|348|312|358|434|481|323|179|177|404|394|440|240|342|563|238|109|368|276|289|499|169|391|346|309|263|555|223|314|536|234|214|282|181|492|475|431|524|310|218|450|305|371|435|556|114|110|307|425|329|166|193|526|315|201|557|395|548|163|135|540|501|473|164|118|171 | 1.95E-22 |  | GO.0051704 | 1.99788107 | 0 |
| 6516 | 254 | GO Process |  | regulation of metabolic process | 1.05E-20 | CX3CL1|MAP4K5|TNFRSF1A|TGFBR3|MAPK1|TGFB1|CCL1|CCL13|TNFAIP1|NFKB1|CDKN1B|BCL9|SOGA1|TGFB3|RHOQ|TNFSF10|RASL11A|CCR7|ATG14|TRAF2|LIF|PIAS1|DOCK7|MAP3K10|EXOC4|IRAK2|PDGFRA|TBRG4|CCL21|BCL2L10|TRAF5|ARG2|PDGFRB|MAPK6|MMD|MSR1|SMAD7|SMAD2|RIMS2|TRAF4|SMURF2|MAP3K8|NOX4|IL1A|IL1B|IL7|MAP4K3|ATF2|IL11|STAT3|MAP3K13|PRDX5|LAMP3|CDK6|CCAR1|DOCK3|IRF8|SOD1|ACKR3|IL9|TNFRSF10B|PDCD4|TNFSF4|ATG10|DEPTOR|CSF1R|AIFM1|BAG4|GATAD1|SMAD6|TNFRSF13C|WDR24|TGFA|TRAT1|SNIP1|RICTOR|TNFRSF21|CREBRF|IL22RA2|HIF1AN|AVPR1A|NFATC3|MAP2K1|BCL2L1|MAEA|IL16|IL12A|IL13|SOCS5|IL7R|MAP3K11|PPARD|OXSR1|CFLAR|IL1RAP|TNIP1|PRKRA|C8orf4|NFATC2IP|CSF1|TGIF1|TNFAIP8L3|PAWR|IL25|SOCS1|PIWIL3|SOCS3|IKZF1|SMAD3|BCL9L|IL17F|BCL11A|BAG5|SMAD4|LAMTOR4|MAP4K4|IKZF3|MAP2K3|RBPJ|MAP3K15|ATG7|PEX2|BCL11B|IRF5|FXR1|CCL20|TGFBR2|MAP3K5|MAPK10|SMURF1|MAP3K3|ILF2|NOS1AP|IRF2BP2|KIAA1804|TGFB2|IRF6|IL20|MAPKAPK2|TAB2|HIVEP2|IL6R|GATAD2B|IKZF5|MCL1|ATG5|BAG3|CACUL1|MAP3K7|MTCP1|IRAK1BP1|IRAK1|TNFRSF6B|BCL10|BAG2|EXOSC1|IGSF1|LCOR|YWHAB|AGO3|AGO1|AGO4|TRAF3IP1|CXCR3|TGIF2|TLR4|TGFBR1|ZAK|MAP3K19|TNFRSF1B|HIF3A|BCOR|CCL7|TAB3|BNIP3L|NFIB|IRF4|IL6ST|IL33|JAK2|TNFRSF19|LOXL2|EXOSC8|MAP3K4|TANK|PDCD10|BCL2L11|AKTIP|SOCS4|MAPK8|NFATC2|IRF9|SOCS6|MAP2K7|BCL2|TNFSF11|MAP3K1|MAPK4|BCL6|PPARA|IL6|CXCR4|IL18R1|MAP3K2|NFATC4|TLR6|IRAK4|EIF4E3|MAPK9|TNIK|NCOA2|ILF3|OXR1|MAP2K4|IL10|IKZF2|SMAD1|TNFAIP8|IKBKB|AMBRA1|ATG13|TRAF6|BCLAF1|DICER1|BCORL1|HIF1A|SMAD5|NKRF|TET2|BCL7A|SOD2|APAF1|MAP3K12|MAPKAPK5|MAP3K9|TRAF3|NFAT5|LITAF|TXNIP|TNFRSF11A|IER2|PIAS2|ERC1|MAP2K6|CCL5|NOS1|CCL16|ECSCR|PANO|SOCS2|TNFAIP3|SOCS7|CCL4|MAP3K14 | 77 | 325|451|351|447|469|350|484|485|280|529|285|175|317|265|552|202|205|243|150|444|432|554|413|489|159|258|516|496|500|550|142|518|506|235|427|147|195|200|331|194|362|165|364|322|345|266|141|189|126|334|490|208|377|365|111|225|398|281|247|295|410|311|383|155|408|153|482|409|530|369|224|260|279|527|337|302|513|296|272|228|222|210|292|372|207|385|519|418|226|176|439|227|504|133|199|538|196|293|347|149|143|248|396|478|460|182|246|220|241|125|367|507|124|470|188|254|392|528|438|384|119|269|128|160|245|308|332|335|380|464|299|443|212|178|190|376|487|161|268|184|433|348|414|412|278|312|318|522|358|162|400|434|481|323|116|137|132|233|440|520|239|240|474|230|259|342|368|264|352|276|510|541|289|206|499|170|169|391|346|309|256|480|297|466|314|316|234|424|523|282|115|492|244|236|475|430|397|231|310|491|218|450|287|374|249|305|371|403|250|535|455|110|144|517|425|486|193|185|526|131|559|315|180|121|387|495|201|291|294|303|381|148|426|370|290|557|393|395|415|163|354|284|401|255|135|508|501|253|172|419|164|186|118|171 | 1.95E-22 |  | GO.0019222 | 1.99788107 | 0 |
| 488 | 57 | GO Process |  | regulation of protein serine/threonine kinase activity | 2.00E-20 | MAP4K5|MAPK1|TGFB1|CDKN1B|TGFB3|TRAF2|MAP3K10|IRAK2|PDGFRB|MAP3K8|NOX4|IL1B|MAP4K3|MAP3K13|CDK6|SOD1|PDCD4|CSF1R|TGFA|MAP2K1|MAP3K11|C8orf4|MAP4K4|MAP2K3|MAP3K15|MAP3K5|MAPK10|MAP3K3|KIAA1804|MAPKAPK2|TAB2|MAP3K7|MTCP1|IRAK1|TLR4|TGFBR1|ZAK|TAB3|JAK2|MAP3K4|PDCD10|MAP2K7|TNFSF11|MAP3K1|CXCR4|MAP3K2|TLR6|TNIK|MAP2K4|TRAF6|MAP3K12|MAPKAPK5|MAP3K9|TNFRSF11A|MAP2K6|TNFAIP3|MAP3K14 | 77 | 451|469|350|285|265|444|489|258|506|165|364|345|141|490|365|281|311|153|279|292|439|293|254|528|384|335|380|299|190|268|184|358|162|434|342|368|264|206|309|466|316|236|430|397|450|374|305|535|517|315|426|370|290|163|255|164|171 | 3.77E-22 |  | GO.0071900 | 1.969897 | 0 |
| 873 | 76 | GO Process |  | regulation of immune response | 2.10E-20 | MAPK1|TGFB1|NFKB1|CRTAM|IL1RL1|TGFB3|CCR7|TRAF2|PIAS1|LILRA1|IRAK2|ARG2|SMAD7|IL1B|DOCK1|TNFSF4|TNFRSF13C|TRAT1|TNFRSF21|NFATC3|IL12A|CTLA4|IL13|SOCS5|IL7R|TNIP1|PAWR|SOCS1|SOCS3|SMAD3|LAMP1|KIR2DL4|NCR3LG1|IFNAR2|MAPK10|TGFB2|MAPKAPK2|TAB2|THEMIS|ATG5|MAP3K7|IRAK1|BCL10|TLR4|HLA-DQB1|TNFSF13B|TAB3|IRF4|IL6ST|IL33|JAK2|TANK|MAPK8|NFATC2|BCL2|MAP3K1|BCL6|IL6|IL1R1|IL18R1|TLR6|IRAK4|MAPK9|ITK|HLA-DMB|HLA-B|IL10|TICAM2|LAT2|IKBKB|TRAF6|TRAF3|IKBKE|MAP2K6|CCL5|TNFAIP3 | 77 | 469|350|529|151|336|265|243|444|554|344|258|518|195|345|215|383|224|527|513|210|519|183|418|226|176|538|396|460|246|241|360|515|313|452|380|376|268|184|349|312|358|434|323|342|109|326|206|169|391|346|309|314|282|115|475|397|310|218|274|287|305|371|250|556|553|114|425|329|467|526|315|557|548|255|135|164 | 4.01E-22 |  | GO.0050776 | 1.967778071 | 0 |
| 5982 | 239 | GO Process |  | regulation of primary metabolic process | 3.19E-20 | CX3CL1|MAP4K5|TNFRSF1A|TGFBR3|MAPK1|TGFB1|CCL1|CCL13|TNFAIP1|NFKB1|CDKN1B|BCL9|SOGA1|TGFB3|RHOQ|TNFSF10|RASL11A|CCR7|ATG14|TRAF2|LIF|PIAS1|DOCK7|MAP3K10|IRAK2|PDGFRA|TBRG4|CCL21|BCL2L10|TRAF5|PDGFRB|MMD|SMAD7|SMAD2|TRAF4|SMURF2|MAP3K8|NOX4|IL1A|IL1B|MAP4K3|ATF2|IL11|STAT3|MAP3K13|PRDX5|LAMP3|CDK6|CCAR1|DOCK3|IRF8|SOD1|ACKR3|IL9|TNFRSF10B|PDCD4|TNFSF4|ATG10|DEPTOR|CSF1R|AIFM1|BAG4|GATAD1|SMAD6|TNFRSF13C|TGFA|SNIP1|RICTOR|TNFRSF21|CREBRF|IL22RA2|HIF1AN|AVPR1A|NFATC3|MAP2K1|MAEA|IL16|IL12A|IL13|SOCS5|IL7R|MAP3K11|PPARD|OXSR1|CFLAR|IL1RAP|TNIP1|C8orf4|NFATC2IP|CSF1|TGIF1|TNFAIP8L3|PAWR|IL25|SOCS1|PIWIL3|SOCS3|IKZF1|SMAD3|BCL9L|IL17F|BCL11A|BAG5|SMAD4|MAP4K4|IKZF3|MAP2K3|RBPJ|MAP3K15|ATG7|PEX2|BCL11B|IRF5|FXR1|CCL20|TGFBR2|MAP3K5|MAPK10|SMURF1|MAP3K3|ILF2|NOS1AP|IRF2BP2|KIAA1804|TGFB2|IRF6|IL20|MAPKAPK2|TAB2|HIVEP2|IL6R|GATAD2B|IKZF5|ATG5|BAG3|CACUL1|MAP3K7|MTCP1|IRAK1BP1|IRAK1|TNFRSF6B|BCL10|BAG2|EXOSC1|IGSF1|LCOR|YWHAB|AGO3|AGO1|AGO4|TRAF3IP1|CXCR3|TGIF2|TLR4|TGFBR1|ZAK|MAP3K19|TNFRSF1B|HIF3A|BCOR|CCL7|TAB3|NFIB|IRF4|IL6ST|IL33|JAK2|TNFRSF19|LOXL2|EXOSC8|MAP3K4|TANK|PDCD10|BCL2L11|AKTIP|SOCS4|MAPK8|NFATC2|IRF9|SOCS6|MAP2K7|BCL2|TNFSF11|MAP3K1|BCL6|PPARA|IL6|CXCR4|IL18R1|MAP3K2|NFATC4|TLR6|IRAK4|EIF4E3|MAPK9|TNIK|NCOA2|ILF3|OXR1|MAP2K4|IL10|IKZF2|SMAD1|TNFAIP8|IKBKB|AMBRA1|TRAF6|BCLAF1|DICER1|BCORL1|HIF1A|SMAD5|NKRF|TET2|BCL7A|SOD2|APAF1|MAP3K12|MAPKAPK5|MAP3K9|TRAF3|NFAT5|LITAF|TXNIP|TNFRSF11A|IER2|PIAS2|ERC1|MAP2K6|CCL5|NOS1|CCL16|ECSCR|PANO|SOCS2|TNFAIP3|SOCS7|CCL4|MAP3K14 | 77 | 325|451|351|447|469|350|484|485|280|529|285|175|317|265|552|202|205|243|150|444|432|554|413|489|258|516|496|500|550|142|506|427|195|200|194|362|165|364|322|345|141|189|126|334|490|208|377|365|111|225|398|281|247|295|410|311|383|155|408|153|482|409|530|369|224|279|337|302|513|296|272|228|222|210|292|207|385|519|418|226|176|439|227|504|133|199|538|293|347|149|143|248|396|478|460|182|246|220|241|125|367|507|124|470|254|392|528|438|384|119|269|128|160|245|308|332|335|380|464|299|443|212|178|190|376|487|161|268|184|433|348|414|412|312|318|522|358|162|400|434|481|323|116|137|132|233|440|520|239|240|474|230|259|342|368|264|352|276|510|541|289|206|170|169|391|346|309|256|480|297|466|314|316|234|424|523|282|115|492|244|236|475|430|397|310|491|218|450|287|374|249|305|371|403|250|535|455|110|144|517|425|486|193|185|526|131|315|180|121|387|495|201|291|294|303|381|148|426|370|290|557|393|395|415|163|354|284|401|255|135|508|501|253|172|419|164|186|118|171 | 6.15E-22 |  | GO.0080090 | 1.949620932 | 0 |
| 6072 | 241 | GO Process |  | regulation of macromolecule metabolic process | 4.29E-20 | CX3CL1|MAP4K5|TNFRSF1A|TGFBR3|MAPK1|TGFB1|CCL1|CCL13|TNFAIP1|NFKB1|CDKN1B|BCL9|TGFB3|RHOQ|TNFSF10|RASL11A|CCR7|ATG14|TRAF2|LIF|PIAS1|DOCK7|MAP3K10|IRAK2|PDGFRA|TBRG4|CCL21|BCL2L10|TRAF5|PDGFRB|MAPK6|MMD|MSR1|SMAD7|SMAD2|RIMS2|TRAF4|SMURF2|MAP3K8|NOX4|IL1A|IL1B|IL7|MAP4K3|ATF2|IL11|STAT3|MAP3K13|PRDX5|LAMP3|CDK6|CCAR1|DOCK3|IRF8|SOD1|ACKR3|IL9|TNFRSF10B|PDCD4|TNFSF4|ATG10|DEPTOR|CSF1R|AIFM1|BAG4|GATAD1|SMAD6|TNFRSF13C|TGFA|TRAT1|SNIP1|RICTOR|CREBRF|IL22RA2|HIF1AN|NFATC3|MAP2K1|IL16|IL12A|IL13|SOCS5|IL7R|MAP3K11|PPARD|OXSR1|CFLAR|IL1RAP|TNIP1|PRKRA|C8orf4|NFATC2IP|CSF1|TGIF1|TNFAIP8L3|PAWR|IL25|SOCS1|PIWIL3|SOCS3|IKZF1|SMAD3|BCL9L|IL17F|BCL11A|BAG5|SMAD4|MAP4K4|IKZF3|MAP2K3|RBPJ|MAP3K15|ATG7|PEX2|BCL11B|IRF5|FXR1|CCL20|TGFBR2|MAP3K5|MAPK10|SMURF1|MAP3K3|ILF2|NOS1AP|IRF2BP2|KIAA1804|TGFB2|IRF6|IL20|MAPKAPK2|TAB2|HIVEP2|IL6R|GATAD2B|IKZF5|ATG5|BAG3|CACUL1|MAP3K7|MTCP1|IRAK1BP1|IRAK1|TNFRSF6B|BCL10|BAG2|EXOSC1|IGSF1|LCOR|YWHAB|AGO3|AGO1|AGO4|TRAF3IP1|CXCR3|TGIF2|TLR4|TGFBR1|ZAK|MAP3K19|TNFRSF1B|HIF3A|BCOR|CCL7|TAB3|NFIB|IRF4|IL6ST|IL33|JAK2|TNFRSF19|LOXL2|EXOSC8|MAP3K4|TANK|PDCD10|BCL2L11|AKTIP|SOCS4|MAPK8|NFATC2|IRF9|SOCS6|MAP2K7|BCL2|TNFSF11|MAP3K1|MAPK4|BCL6|PPARA|IL6|CXCR4|IL18R1|MAP3K2|NFATC4|TLR6|IRAK4|EIF4E3|MAPK9|TNIK|NCOA2|ILF3|OXR1|MAP2K4|IL10|IKZF2|SMAD1|TNFAIP8|IKBKB|TRAF6|BCLAF1|DICER1|BCORL1|HIF1A|SMAD5|NKRF|TET2|BCL7A|SOD2|APAF1|MAP3K12|MAPKAPK5|MAP3K9|TRAF3|NFAT5|LITAF|TXNIP|TNFRSF11A|IER2|PIAS2|ERC1|MAP2K6|CCL5|NOS1|CCL16|ECSCR|PANO|SOCS2|TNFAIP3|SOCS7|CCL4|MAP3K14 | 77 | 325|451|351|447|469|350|484|485|280|529|285|175|265|552|202|205|243|150|444|432|554|413|489|258|516|496|500|550|142|506|235|427|147|195|200|331|194|362|165|364|322|345|266|141|189|126|334|490|208|377|365|111|225|398|281|247|295|410|311|383|155|408|153|482|409|530|369|224|279|527|337|302|296|272|228|210|292|385|519|418|226|176|439|227|504|133|199|538|196|293|347|149|143|248|396|478|460|182|246|220|241|125|367|507|124|470|254|392|528|438|384|119|269|128|160|245|308|332|335|380|464|299|443|212|178|190|376|487|161|268|184|433|348|414|412|312|318|522|358|162|400|434|481|323|116|137|132|233|440|520|239|240|474|230|259|342|368|264|352|276|510|541|289|206|170|169|391|346|309|256|480|297|466|314|316|234|424|523|282|115|492|244|236|475|430|397|231|310|491|218|450|287|374|249|305|371|403|250|535|455|110|144|517|425|486|193|185|526|315|180|121|387|495|201|291|294|303|381|148|426|370|290|557|393|395|415|163|354|284|401|255|135|508|501|253|172|419|164|186|118|171 | 8.36E-22 |  | GO.0060255 | 1.936754271 | 0 |
| 5827 | 234 | GO Process |  | regulation of nitrogen compound metabolic process | 7.31E-20 | CX3CL1|MAP4K5|TNFRSF1A|TGFBR3|MAPK1|TGFB1|CCL1|CCL13|TNFAIP1|NFKB1|CDKN1B|BCL9|TGFB3|RHOQ|TNFSF10|RASL11A|CCR7|ATG14|TRAF2|LIF|PIAS1|DOCK7|MAP3K10|IRAK2|PDGFRA|TBRG4|CCL21|BCL2L10|TRAF5|PDGFRB|MMD|SMAD7|SMAD2|TRAF4|SMURF2|MAP3K8|NOX4|IL1A|IL1B|MAP4K3|ATF2|IL11|STAT3|MAP3K13|PRDX5|LAMP3|CDK6|CCAR1|DOCK3|IRF8|SOD1|ACKR3|IL9|TNFRSF10B|PDCD4|TNFSF4|ATG10|DEPTOR|CSF1R|AIFM1|BAG4|GATAD1|SMAD6|TNFRSF13C|TGFA|SNIP1|RICTOR|CREBRF|IL22RA2|HIF1AN|NFATC3|MAP2K1|IL16|IL12A|IL13|SOCS5|IL7R|MAP3K11|PPARD|OXSR1|CFLAR|IL1RAP|TNIP1|C8orf4|NFATC2IP|CSF1|TGIF1|TNFAIP8L3|PAWR|IL25|SOCS1|PIWIL3|SOCS3|IKZF1|SMAD3|BCL9L|IL17F|BCL11A|BAG5|SMAD4|MAP4K4|IKZF3|MAP2K3|RBPJ|MAP3K15|ATG7|PEX2|BCL11B|IRF5|FXR1|CCL20|TGFBR2|MAP3K5|MAPK10|SMURF1|MAP3K3|ILF2|NOS1AP|IRF2BP2|KIAA1804|TGFB2|IRF6|IL20|MAPKAPK2|TAB2|HIVEP2|IL6R|GATAD2B|IKZF5|ATG5|BAG3|CACUL1|MAP3K7|MTCP1|IRAK1BP1|IRAK1|TNFRSF6B|BCL10|BAG2|EXOSC1|IGSF1|LCOR|YWHAB|AGO3|AGO1|AGO4|TRAF3IP1|CXCR3|TGIF2|TLR4|TGFBR1|ZAK|MAP3K19|TNFRSF1B|HIF3A|BCOR|CCL7|TAB3|NFIB|IRF4|IL6ST|IL33|JAK2|TNFRSF19|LOXL2|EXOSC8|MAP3K4|TANK|PDCD10|BCL2L11|AKTIP|SOCS4|MAPK8|NFATC2|IRF9|SOCS6|MAP2K7|BCL2|TNFSF11|MAP3K1|BCL6|PPARA|IL6|CXCR4|IL18R1|MAP3K2|NFATC4|TLR6|IRAK4|EIF4E3|MAPK9|TNIK|NCOA2|ILF3|OXR1|MAP2K4|IL10|IKZF2|SMAD1|TNFAIP8|IKBKB|TRAF6|BCLAF1|DICER1|BCORL1|HIF1A|SMAD5|NKRF|TET2|BCL7A|SOD2|APAF1|MAP3K12|MAPKAPK5|MAP3K9|TRAF3|NFAT5|LITAF|TXNIP|TNFRSF11A|IER2|PIAS2|ERC1|MAP2K6|CCL5|NOS1|CCL16|ECSCR|PANO|SOCS2|TNFAIP3|SOCS7|CCL4|MAP3K14 | 77 | 325|451|351|447|469|350|484|485|280|529|285|175|265|552|202|205|243|150|444|432|554|413|489|258|516|496|500|550|142|506|427|195|200|194|362|165|364|322|345|141|189|126|334|490|208|377|365|111|225|398|281|247|295|410|311|383|155|408|153|482|409|530|369|224|279|337|302|296|272|228|210|292|385|519|418|226|176|439|227|504|133|199|538|293|347|149|143|248|396|478|460|182|246|220|241|125|367|507|124|470|254|392|528|438|384|119|269|128|160|245|308|332|335|380|464|299|443|212|178|190|376|487|161|268|184|433|348|414|412|312|318|522|358|162|400|434|481|323|116|137|132|233|440|520|239|240|474|230|259|342|368|264|352|276|510|541|289|206|170|169|391|346|309|256|480|297|466|314|316|234|424|523|282|115|492|244|236|475|430|397|310|491|218|450|287|374|249|305|371|403|250|535|455|110|144|517|425|486|193|185|526|315|180|121|387|495|201|291|294|303|381|148|426|370|290|557|393|395|415|163|354|284|401|255|135|508|501|253|172|419|164|186|118|171 | 1.44E-21 |  | GO.0051171 | 1.913608262 | 0 |
| 75 | 27 | GO Process |  | chemokine-mediated signaling pathway | 7.34E-20 | CX3CL1|CCL1|CCL13|CXCL6|CCR7|CCL21|ACKR3|CXCR5|CXCL3|CXCL5|CXCL11|CCR4|GPR29|CCRL2|CCR9|CCL20|CXCL9|CXCR3|CCL7|CXCL12|CMKBR6|CXCR4|CXCR6|ACKR2|CCL5|CCL16|CCL4 | 77 | 325|484|485|531|243|500|247|306|356|355|321|502|448|134|456|308|283|230|289|181|431|450|435|173|135|501|118 | 1.46E-21 |  | GO.0070098 | 1.913430394 | 0 |
| 48 | 24 | Reactome Pathways | | Chemokine receptors bind chemokines | 9.67E-20 | CX3CL1|CCL1|CCL13|CXCL6|CCR7|CCL21|ACKR3|CXCR5|CXCL3|CXCL5|CXCL11|CCR4|CCRL2|CCR9|CCL20|CCL28|CXCL9|CXCR3|CXCL12|CMKBR6|CXCR4|CXCR6|CCL5|CCL16 | 77 | 325|484|485|531|243|500|247|306|356|355|321|502|134|456|308|488|283|230|181|431|450|435|135|501 | 5.74E-22 |  | HSA-380108 | 1.901457353 | 0 |
| 2788 | 145 | GO Process |  | regulation of multicellular organismal process | 2.20E-19 | CX3CL1|TNFRSF1A|TGFBR3|MAPK1|TGFB1|NFKB1|CRTAM|CDKN1B|IL1RL1|TGFB3|TNFSF9|CCR7|TRAF2|LIF|DOCK7|PDGFRA|HILPDA|ARG2|PDGFRB|MAPK6|MMD|SMAD7|SMAD2|RIMS2|SMURF2|IL1A|IL1B|IL7|ATF2|STAT3|CDK6|IRF8|SOD1|IL9|DOCK5|PDCD4|DOCK1|TNFSF4|CSF1R|IL17RB|SMAD6|TNFRSF13C|FAM19A4|TNFRSF21|TNFRSF11B|HIF1AN|AVPR1A|NFATC3|MAP2K1|IL12A|CTLA4|IL13|SOCS5|IL7R|PPARD|CFLAR|IL1RAP|RHOJ|C8orf4|TNFRSF12A|CSF1|SOCS1|SMAD3|PDCD1|BCL9L|IL17F|BCL11A|BAG5|SMAD4|MAP4K4|GPR29|IKZF3|MAP2K3|RBPJ|BCL11B|IRF5|CCL20|TGFBR2|SMURF1|MAP3K3|NOS1AP|TGFB2|IL20|MAPKAPK2|IL6R|ATG5|MAP3K7|IRAK1|BCL10|AGO3|AGO1|AGO4|TRAF3IP1|CXCR3|TGIF2|TLR4|TGFBR1|TNFRSF1B|BCOR|NFIB|IRF4|IL6ST|IL33|JAK2|LOXL2|PDCD10|IL36RN|BCL2L11|ULK2|CXCL12|NFATC2|PDCD1LG2|BCL2|TNFSF11|CMKBR6|BCL6|IL6|IL1R1|CXCR4|IL18R1|NFATC4|TLR6|MAPKBP1|HLA-B|TNIK|DOCK4|IL10|TICAM2|SMAD1|IKBKB|TRAF6|DICER1|HIF1A|SMAD5|SOD2|TRAF3|LITAF|IKBKE|TNFRSF11A|PIAS2|NOS1|TNFRSF9|ECSCR|SOCS2|TNFAIP3 | 77 | 325|351|447|469|350|529|151|285|336|265|120|243|444|432|413|516|462|518|506|235|427|195|200|331|362|322|345|266|189|334|365|398|281|295|406|311|215|383|153|497|369|224|545|513|359|228|222|210|292|519|183|418|226|176|227|133|199|375|293|340|149|460|241|537|125|367|507|124|470|254|448|392|528|438|128|160|308|332|464|299|212|376|161|268|348|312|358|434|323|520|239|240|474|230|259|342|368|276|541|170|169|391|346|309|480|316|536|234|214|181|115|373|475|430|431|310|218|274|450|287|249|305|298|114|535|544|425|329|193|526|315|121|495|201|381|557|395|548|163|284|508|473|253|419|164 | 4.42E-21 |  | GO.0051239 | 1.865757732 | 0 |
| 1594 | 103 | GO Process |  | regulation of cell population proliferation | 4.52E-19 | RELT|TNFRSF1A|TGFBR3|MAPK1|TGFB1|CXCL6|CDKN1B|TGFB3|TNFSF9|LIF|PIAS1|PDGFRA|HILPDA|TBRG4|ARG2|PDGFRB|SMAD2|NOX4|IL1A|IL1B|IL7|ATF2|IL11|STAT3|CDK6|CCAR1|IL9|TNFRSF10B|PDCD4|TNFSF4|CSF1R|SMAD6|TNFRSF13C|TGFA|CXCL3|CXCL5|RICTOR|TNFRSF21|TNFRSF11B|AVPR1A|LTBP3|MAP2K1|BCL2L1|IL12A|CTLA4|IL13|IL7R|CXCL11|PPARD|CFLAR|PRKRA|CSF1|IFNLR1|PAWR|SMAD3|SMAD4|IKZF3|RBPJ|PEX2|BCL11B|TGFBR2|MAP3K5|CXCL9|MAP3K3|TGFB2|IRF6|IL6R|CACUL1|IRAK1|TNFRSF6B|IFIT3|AGO3|CXCR3|TGFBR1|TNFSF13B|TNFRSF1B|NFIB|IL6ST|JAK2|PDCD10|PRC1|CXCL12|NFATC2|PDCD1LG2|BCL2|BCL6|IL6|IRAK4|ITK|HLA-DMB|TBRG1|IL10|SMAD1|AMBRA1|TRAF6|DICER1|HIF1A|SOD2|TXNIP|TNFRSF11A|CCL5|TNFRSF9|TNFAIP3 | 77 | 532|351|447|469|350|531|285|265|120|432|554|516|462|496|518|506|200|364|322|345|266|189|126|334|365|111|295|410|311|383|153|369|224|279|356|355|302|513|359|222|441|292|372|519|183|418|176|321|227|133|196|149|341|396|241|470|392|438|269|128|332|335|283|299|376|487|348|522|434|481|404|520|230|368|326|276|170|391|309|316|511|181|115|373|475|310|218|371|556|553|445|425|193|131|315|121|495|381|415|163|135|473|164 | 9.33E-21 |  | GO.0042127 | 1.834486157 | 0 |
| 317 | 45 | GO Process |  | response to molecule of bacterial origin | 6.09E-19 | RELT|TNFRSF1A|MAPK1|TGFB1|CXCL6|NFKB1|CCR7|IRAK2|LOXL1|IL1B|IRF8|TNFRSF10B|PDCD4|TNFSF4|CXCL3|CXCL5|TNFRSF21|TNFRSF11B|IL12A|IL13|CXCL11|PPARD|TNIP1|IRF5|CCL20|CXCL9|MAPKAPK2|IRAK1|TNFRSF6B|BCL10|TLR4|TNFRSF1B|JAK2|MAPK8|CSF2RB|IL6|TLR6|IL10|TICAM2|TRAF6|LITAF|TNFRSF11A|CCL5|TNFRSF9|TNFAIP3 | 77 | 532|351|469|350|531|529|243|258|498|345|398|410|311|383|356|355|513|359|519|418|321|227|538|160|308|283|268|434|481|323|342|276|309|282|524|218|305|425|329|315|395|163|135|473|164 | 1.27E-20 |  | GO.0002237 | 1.821538271 | 0 |
| 217 | 38 | GO Process |  | response to tumor necrosis factor | 1.19E-18 | CX3CL1|RELT|TNFRSF1A|MAPK1|CCL1|CCL13|NFKB1|TNFSF9|TRAF2|TXNDC17|CCL21|MAP4K3|TNFRSF10B|TNFSF4|BAG4|TNFRSF13C|TNFRSF21|TNFRSF11B|TNFRSF12A|CCL20|MAP3K5|TNFRSF6B|TNFSF13B|TNFRSF1B|CCL7|JAK2|TNFRSF19|TANK|MAP2K7|TNFSF11|IKBKB|TRAF3|TNFRSF11A|CCL5|CCL16|TNFRSF9|CCL4|MAP3K14 | 77 | 325|532|351|469|484|485|529|120|444|140|500|141|410|383|409|224|513|359|340|308|335|481|326|276|289|309|256|314|236|430|526|557|163|135|501|473|118|171 | 2.51E-20 |  | GO.0034612 | 1.792445304 | 0 |
| 126 | 31 | Reactome Pathways | | Toll Like Receptor 4 (TLR4) Cascade | 2.39E-18 | MAPK1|NFKB1|IRAK2|MAP3K8|ATF2|MAP2K1|NKIRAS2|SOCS1|MAP2K3|MAPK10|MAPKAPK2|TAB2|MAP3K7|IRAK1|TLR4|TAB3|TANK|MAPK8|MAP2K7|MAP3K1|TLR6|IRAK4|NKIRAS1|MAPK9|MAP2K4|TICAM2|IKBKB|TRAF6|TRAF3|IKBKE|MAP2K6 | 77 | 469|529|258|165|189|292|273|460|528|380|268|184|358|434|342|206|314|282|236|397|305|371|417|250|517|329|526|315|557|548|255 | 1.78E-20 |  | HSA-166016 | 1.76216021 | 0 |
| 102 | 28 | KEGG Pathways | | Th17 cell differentiation | 2.41E-18 | MAPK1|TGFB1|NFKB1|SMAD2|IL1B|STAT3|NFATC3|SMAD3|IL17F|IL21R|SMAD4|TGFBR2|MAPK10|IL6R|IL2RG|TGFBR1|IRF4|IL6ST|JAK2|MAPK8|NFATC2|IL6|IL1R1|MAPK9|HLA-DMB|IKBKB|HIF1A|IL22 | 77 | 469|350|529|200|345|334|210|241|367|217|470|332|380|348|563|368|169|391|309|282|115|218|274|250|553|526|495|534 | 1.10E-19 |  | hsa04659 | 1.761798296 | 0 |
| 1513 | 99 | GO Function | | signaling receptor binding | 3.00E-18 | CX3CL1|TGFBR3|TGFB1|CCL1|CCL13|CXCL6|CRTAM|TGFB3|TNFSF10|TNFSF9|TRAF2|LIF|PIAS1|PXDN|PDGFRA|HILPDA|IL36G|IL36B|CCL21|TRAF5|PDGFRB|SMAD7|SMAD2|TRAF4|SMURF2|IL1A|IL1B|IL7|IL11|STAT3|PECR|PRDX5|IL9|TNFSF4|SMAD6|TGFA|TRAT1|CXCL3|CXCL5|TNFRSF11B|DEFB1|HIF1AN|AVPR1A|IL16|IL12A|DEFB4A|IL13|SOCS5|ALCAM|CXCL11|CFLAR|C8orf4|CSF1|IL25|SOCS1|SMAD3|CXCL14|IL17F|CCRL2|CCL20|TGFBR2|CCL28|CXCL9|TGFB2|IL20|MAP3K7|IGSF1|TLR4|TGFBR1|TNFSF13B|CCL7|IL6ST|IL33|JAK2|IL36RN|CXCL12|TNFSF11|IL6|IL1R1|NFATC4|TLR6|IRAK4|OSGIN2|HLA-B|NCOA2|DOCK4|PDCD6IP|IL10|TGFBI|DEFB4B|TRAF6|HIF1A|IL22|TRAF3|PIAS2|CCL5|CCL16|SOCS2|CCL4 | 77 | 325|447|350|484|485|531|151|265|202|120|444|432|554|543|516|462|561|558|500|142|506|195|200|194|362|322|345|266|126|334|436|208|295|383|369|279|527|356|355|359|112|228|222|385|519|158|418|226|286|321|133|293|149|478|460|241|468|367|134|308|332|488|283|376|161|358|132|342|368|326|289|391|346|309|536|181|430|218|274|249|305|371|446|114|455|544|307|425|157|166|315|495|534|557|284|135|501|419|118 | 2.80E-20 |  | GO.0005102 | 1.752287875 | 0 |
| 183 | 35 | GO Process |  | cell chemotaxis | 3.28E-18 | CX3CL1|CCL1|CCL13|CXCL6|CCR7|PDGFRA|CCL21|PDGFRB|IL1B|CXCR5|CXCL3|CXCL5|IL16|CXCL11|CXCL14|GPR29|CCL20|CCL28|CXCL9|TGFB2|IL6R|CXCR3|CCL7|LYST|CXCL12|TNFSF11|CMKBR6|IL6|CXCR4|DOCK4|IL10|TNFRSF11A|CCL5|CCL16|CCL4 | 77 | 325|484|485|531|243|516|500|506|345|306|356|355|385|321|468|448|308|488|283|376|348|230|289|555|181|430|431|218|450|544|425|163|135|501|118 | 6.94E-20 |  | GO.0060326 | 1.748412616 | 0 |
| 197 | 36 | GO Process |  | cellular response to tumor necrosis factor | 3.53E-18 | CX3CL1|RELT|TNFRSF1A|MAPK1|CCL1|CCL13|NFKB1|TNFSF9|TRAF2|TXNDC17|CCL21|TNFRSF10B|TNFSF4|BAG4|TNFRSF13C|TNFRSF21|TNFRSF11B|TNFRSF12A|CCL20|MAP3K5|TNFRSF6B|TNFSF13B|TNFRSF1B|CCL7|JAK2|TNFRSF19|TANK|TNFSF11|IKBKB|TRAF3|TNFRSF11A|CCL5|CCL16|TNFRSF9|CCL4|MAP3K14 | 77 | 325|532|351|469|484|485|529|120|444|140|500|410|383|409|224|513|359|340|308|335|481|326|276|289|309|256|314|430|526|557|163|135|501|473|118|171 | 7.54E-20 |  | GO.0071356 | 1.745222529 | 0 |
| 1236 | 87 | GO Process |  | phosphorylation | 5.68E-18 | MAP4K5|TGFBR3|MAPK1|TGFB1|NFKB1|CDKN1B|LIF|NDUFA10|MAP3K10|IRAK2|PDGFRA|TBRG4|PDGFRB|MAPK6|MMD|SMAD7|SMAD2|MAP3K8|IL1B|ITPKC|MAP4K3|STAT3|MAP3K13|CDK6|ETNK1|CSF1R|TRAT1|IL17RD|RICTOR|MAP2K1|MAP3K11|OXSR1|PRKRA|ICOS|AATK|MAP4K4|MAP2K3|MAP3K15|TGFBR2|MAP3K5|MAPK10|MAP3K3|KIAA1804|TGFB2|MAPKAPK2|TAB2|MAP3K7|IRAK1|PI4K2A|YWHAB|TLR4|IL2RG|TGFBR1|ZAK|MAP3K19|TAB3|JAK2|TNFRSF19|MAP3K4|ULK2|MAPK8|MAP2K7|BCL2|TNFSF11|MAP3K1|MAPK4|CSF2RB|LTBP1|MAP3K2|IRAK4|MAPK9|ITK|TNIK|ILF3|MAP2K4|SMAD1|IKBKB|TRAF6|SMAD5|MAP3K12|MAPKAPK5|MAP3K9|IKBKE|ERC1|MAP2K6|CCL5|MAP3K14 | 77 | 451|447|469|350|529|285|432|275|489|258|516|496|506|235|427|195|200|165|345|420|141|334|490|365|191|153|527|136|302|292|439|504|196|167|560|254|528|384|332|335|380|299|190|376|268|184|358|434|399|440|342|563|368|264|352|206|309|256|466|214|282|236|475|430|397|231|524|154|374|371|250|556|535|110|517|193|526|315|201|426|370|290|548|401|255|135|171 | 1.22E-19 |  | GO.0016310 | 1.724565166 | 0 |
| 1551 | 99 | GO Process |  | positive regulation of multicellular organismal process | 6.67E-18 | CX3CL1|TGFBR3|TGFB1|NFKB1|CRTAM|IL1RL1|TGFB3|TNFSF9|CCR7|TRAF2|LIF|HILPDA|PDGFRB|MAPK6|MMD|SMAD2|RIMS2|SMURF2|IL1A|IL1B|IL7|ATF2|STAT3|IRF8|SOD1|IL9|DOCK5|DOCK1|TNFSF4|CSF1R|IL17RB|TNFRSF13C|HIF1AN|AVPR1A|MAP2K1|IL12A|IL13|SOCS5|IL7R|CFLAR|IL1RAP|RHOJ|TNFRSF12A|CSF1|SOCS1|SMAD3|BCL9L|IL17F|BCL11A|SMAD4|GPR29|MAP2K3|RBPJ|IRF5|CCL20|TGFBR2|SMURF1|MAP3K3|NOS1AP|TGFB2|IL20|MAPKAPK2|IL6R|ATG5|MAP3K7|IRAK1|BCL10|CXCR3|TGIF2|TLR4|TGFBR1|IRF4|IL6ST|IL33|JAK2|LOXL2|CXCL12|BCL2|TNFSF11|CMKBR6|BCL6|IL6|IL1R1|CXCR4|IL18R1|NFATC4|TLR6|IL10|TICAM2|SMAD1|TRAF6|DICER1|HIF1A|SMAD5|SOD2|TNFRSF11A|NOS1|SOCS2|TNFAIP3 | 77 | 325|447|350|529|151|336|265|120|243|444|432|462|506|235|427|200|331|362|322|345|266|189|334|398|281|295|406|215|383|153|497|224|228|222|292|519|418|226|176|133|199|375|340|149|460|241|125|367|507|470|448|528|438|160|308|332|464|299|212|376|161|268|348|312|358|434|323|230|259|342|368|169|391|346|309|480|181|475|430|431|310|218|274|450|287|249|305|425|329|193|315|121|495|201|381|163|508|419|164 | 1.45E-19 |  | GO.0051240 | 1.717587417 | 0 |
| 663 | 62 | GO Process |  | positive regulation of cell death | 7.43E-18 | TNFRSF1A|TGFB1|CDKN1B|TGFB3|TNFSF10|TRAF2|MAP3K10|BCL2L10|PDGFRB|NOX4|ATF2|SOD1|TNFRSF10B|PDCD4|AIFM1|BCL2L1|IL12A|CTLA4|MAP3K11|PRKRA|TNFRSF12A|PAWR|SMAD3|PDCD1|ATG7|IRF5|MAP3K5|TGFB2|MCL1|BCL10|IFIT2|YWHAB|CXCR3|TLR4|TGFBR1|ZAK|TNFRSF1B|BNIP3L|JAK2|BCL2L11|MAPK8|BCL2|BCL6|IL6|NFATC4|TLR6|MAPK9|MAP2K4|IL10|PXT1|TNFAIP8|TRAF6|BCLAF1|SOD2|APAF1|MAP3K9|MOAP1|TXNIP|MAP2K6|CCL5|ECSCR|PANO | 77 | 351|350|285|265|202|444|489|550|506|364|189|281|410|311|482|372|519|183|439|196|340|396|241|537|119|160|335|376|278|323|394|440|230|342|368|264|276|499|309|234|282|475|310|218|249|305|250|517|425|363|185|315|180|381|148|290|138|415|255|135|253|172 | 1.63E-19 |  | GO.0010942 | 1.712901119 | 0 |
| 604 | 59 | GO Process |  | positive regulation of apoptotic process | 9.01E-18 | TNFRSF1A|TGFB1|TGFB3|TNFSF10|TRAF2|MAP3K10|BCL2L10|PDGFRB|NOX4|ATF2|SOD1|TNFRSF10B|PDCD4|AIFM1|BCL2L1|IL12A|CTLA4|MAP3K11|PRKRA|TNFRSF12A|PAWR|SMAD3|PDCD1|ATG7|IRF5|MAP3K5|TGFB2|MCL1|BCL10|IFIT2|YWHAB|CXCR3|TGFBR1|ZAK|TNFRSF1B|BNIP3L|JAK2|BCL2L11|MAPK8|BCL2|BCL6|IL6|NFATC4|MAPK9|MAP2K4|IL10|PXT1|TNFAIP8|TRAF6|BCLAF1|SOD2|APAF1|MAP3K9|MOAP1|TXNIP|MAP2K6|CCL5|ECSCR|PANO | 77 | 351|350|265|202|444|489|550|506|364|189|281|410|311|482|372|519|183|439|196|340|396|241|537|119|160|335|376|278|323|394|440|230|368|264|276|499|309|234|282|475|310|218|249|250|517|425|363|185|315|180|381|148|290|138|415|255|135|253|172 | 2.00E-19 |  | GO.0043065 | 1.704527521 | 0 |
| 295 | 42 | GO Process |  | apoptotic signaling pathway | 1.05E-17 | RELT|TNFRSF1A|TGFB1|TRAF2|BCL2L2|BCL2L10|IL1A|IL1B|TNFRSF10B|AIFM1|TNFRSF21|TNFRSF11B|BCL2L1|IL12A|PPARD|TNFRSF12A|PAWR|AEN|SMAD3|LAMP1|MAP3K5|TGFB2|IL6R|MCL1|BAG3|TNFRSF6B|TLR4|TNFRSF1B|BNIP3L|IL33|JAK2|PDCD10|BCL2L11|BCL2|NFATC4|TICAM2|SOD2|APAF1|MOAP1|IKBKE|TNFRSF11A|TNFRSF9 | 77 | 532|351|350|444|551|550|322|345|410|482|513|359|372|519|227|340|396|547|241|360|335|376|348|278|318|481|342|276|499|346|309|316|234|475|249|329|381|148|138|548|163|473 | 2.35E-19 |  | GO.0097190 | 1.69788107 | 0 |
| 85 | 26 | GO Process |  | JNK cascade | 1.06E-17 | MAP4K5|MAP3K10|IRAK2|MAP4K3|MAP3K13|MAP3K11|MAP3K5|MAPK10|KIAA1804|TAB2|MAP3K7|IRAK1|ZAK|TAB3|TNFRSF19|MAPK8|MAP2K7|TNFSF11|MAP3K2|IRAK4|MAPK9|TNIK|MAP2K4|TRAF6|MAP3K12|MAP3K9 | 77 | 451|489|258|141|490|439|335|380|190|184|358|434|264|206|256|282|236|430|374|371|250|535|517|315|426|290 | 2.40E-19 |  | GO.0007254 | 1.697469413 | 0 |
| 298 | 42 | GO Process |  | response to lipopolysaccharide | 1.44E-17 | RELT|TNFRSF1A|MAPK1|TGFB1|CXCL6|NFKB1|CCR7|IRAK2|LOXL1|IL1B|IRF8|TNFRSF10B|PDCD4|TNFSF4|CXCL3|CXCL5|TNFRSF21|TNFRSF11B|IL12A|IL13|CXCL11|PPARD|TNIP1|CCL20|CXCL9|MAPKAPK2|IRAK1|TNFRSF6B|TLR4|TNFRSF1B|JAK2|MAPK8|CSF2RB|IL6|IL10|TICAM2|TRAF6|LITAF|TNFRSF11A|CCL5|TNFRSF9|TNFAIP3 | 77 | 532|351|469|350|531|529|243|258|498|345|398|410|311|383|356|355|513|359|519|418|321|227|538|308|283|268|434|481|342|276|309|282|524|218|425|329|315|395|163|135|473|164 | 3.29E-19 |  | GO.0032496 | 1.684163751 | 0 |
| 555 | 56 | GO Process |  | response to bacterium | 2.00E-17 | RELT|TNFRSF1A|MAPK1|TGFB1|CXCL6|NFKB1|CCR7|IRAK2|LOXL1|IL1B|IRF8|IL22RA1|TNFRSF10B|PDCD4|TNFSF4|CXCL3|CXCL5|TNFRSF21|TNFRSF11B|DEFB1|IL12A|DEFB4A|IL13|CXCL11|PPARD|TNIP1|CCR4|RBPJ|IRF5|CCL20|CXCL9|MAPKAPK2|IL6R|IRAK1|TNFRSF6B|BCL10|IFI44|TLR4|TNFRSF1B|JAK2|LYST|MAPK8|CSF2RB|IL6|TLR6|HLA-B|IL10|TICAM2|DEFB4B|TRAF6|LITAF|TNFRSF11A|CCL5|MR1|TNFRSF9|TNFAIP3 | 77 | 532|351|469|350|531|529|243|258|498|345|398|211|410|311|383|356|355|513|359|112|519|158|418|321|227|538|502|438|160|308|283|268|348|434|481|323|179|342|276|309|555|282|524|218|305|114|425|329|166|315|395|163|135|540|473|164 | 4.63E-19 |  | GO.0009617 | 1.669897 | 0 |
| 96 | 27 | Reactome Pathways | | TRIF(TICAM1)-mediated TLR4 signaling | 2.11E-17 | MAPK1|NFKB1|IRAK2|MAP3K8|ATF2|MAP2K1|NKIRAS2|MAP2K3|MAPK10|MAPKAPK2|TAB2|MAP3K7|IRAK1|TLR4|TAB3|TANK|MAPK8|MAP2K7|NKIRAS1|MAPK9|MAP2K4|TICAM2|IKBKB|TRAF6|TRAF3|IKBKE|MAP2K6 | 77 | 469|529|258|165|189|292|273|528|380|268|184|358|434|342|206|314|282|236|417|250|517|329|526|315|557|548|255 | 2.97E-19 |  | HSA-937061 | 1.667571754 | 0 |
| 151 | 32 | Reactome Pathways | | Toll-like Receptor Cascades | 2.11E-17 | MAPK1|NFKB1|IRAK2|MAP3K8|ATF2|MAP2K1|NKIRAS2|EEA1|SOCS1|MAP2K3|MAPK10|MAPKAPK2|TAB2|MAP3K7|IRAK1|TLR4|TAB3|TANK|MAPK8|MAP2K7|MAP3K1|TLR6|IRAK4|NKIRAS1|MAPK9|MAP2K4|TICAM2|IKBKB|TRAF6|TRAF3|IKBKE|MAP2K6 | 77 | 469|529|258|165|189|292|273|146|460|528|380|268|184|358|434|342|206|314|282|236|397|305|371|417|250|517|329|526|315|557|548|255 | 1.99E-19 |  | HSA-168898 | 1.667571754 | 0 |
| 95 | 27 | Reactome Pathways | | Toll Like Receptor 3 (TLR3) Cascade | 2.11E-17 | MAPK1|NFKB1|IRAK2|MAP3K8|ATF2|MAP2K1|NKIRAS2|MAP2K3|MAPK10|MAPKAPK2|TAB2|MAP3K7|IRAK1|TLR4|TAB3|TANK|MAPK8|MAP2K7|NKIRAS1|MAPK9|MAP2K4|TICAM2|IKBKB|TRAF6|TRAF3|IKBKE|MAP2K6 | 77 | 469|529|258|165|189|292|273|528|380|268|184|358|434|342|206|314|282|236|417|250|517|329|526|315|557|548|255 | 2.37E-19 |  | HSA-168164 | 1.667571754 | 0 |
| 96 | 27 | Reactome Pathways | | Toll Like Receptor 9 (TLR9) Cascade | 2.11E-17 | MAPK1|NFKB1|IRAK2|MAP3K8|ATF2|MAP2K1|NKIRAS2|EEA1|MAP2K3|MAPK10|MAPKAPK2|TAB2|MAP3K7|IRAK1|TLR4|TAB3|MAPK8|MAP2K7|MAP3K1|IRAK4|NKIRAS1|MAPK9|MAP2K4|TICAM2|IKBKB|TRAF6|MAP2K6 | 77 | 469|529|258|165|189|292|273|146|528|380|268|184|358|434|342|206|282|236|397|371|417|250|517|329|526|315|255 | 2.97E-19 |  | HSA-168138 | 1.667571754 | 0 |
| 94 | 27 | Reactome Pathways | | MyD88:MAL(TIRAP) cascade initiated on plasma membrane | 2.11E-17 | MAPK1|NFKB1|IRAK2|MAP3K8|ATF2|MAP2K1|NKIRAS2|SOCS1|MAP2K3|MAPK10|MAPKAPK2|TAB2|MAP3K7|IRAK1|TLR4|TAB3|MAPK8|MAP2K7|MAP3K1|TLR6|IRAK4|NKIRAS1|MAPK9|MAP2K4|IKBKB|TRAF6|MAP2K6 | 77 | 469|529|258|165|189|292|273|460|528|380|268|184|358|434|342|206|282|236|397|305|371|417|250|517|526|315|255 | 1.88E-19 |  | HSA-166058 | 1.667571754 | 0 |
| 482 | 52 | GO Process |  | positive regulation of cellular component movement | 2.93E-17 | MAPK1|TGFB1|CCL1|CXCL6|CCR7|DOCK7|PDGFRA|CCL21|PDGFRB|SMURF2|NOX4|IL1A|IL1B|CCAR1|ACKR3|DOCK5|DOCK1|CSF1R|BAG4|CXCL3|CXCL5|DEFB1|IL12A|CXCL11|RHOJ|CSF1|SMAD3|CXCL14|GPR29|MAP2K3|CCL20|TGFBR2|CXCL9|MAP3K3|NOS1AP|TGFB2|IL6R|TGFBR1|CCL7|JAK2|PDCD10|CXCL12|BCL2|CMKBR6|BCL6|IL6|IL1R1|DOCK4|HIF1A|SOD2|CCL5|CCL4 | 77 | 469|350|484|531|243|413|516|500|506|362|364|322|345|111|247|406|215|153|409|356|355|112|519|321|375|149|241|468|448|528|308|332|283|299|212|376|348|368|289|309|316|181|475|431|310|218|274|544|495|381|135|118 | 6.82E-19 |  | GO.0051272 | 1.653313238 | 0 |
| 503 | 53 | GO Process |  | positive regulation of locomotion | 3.36E-17 | MAPK1|TGFB1|CCL1|CXCL6|CCR7|DOCK7|PDGFRA|CCL21|PDGFRB|SMURF2|NOX4|IL1A|IL1B|CCAR1|ACKR3|DOCK5|DOCK1|CSF1R|BAG4|CXCL3|CXCL5|DEFB1|IL16|IL12A|CXCL11|RHOJ|CSF1|CCR4|SMAD3|CXCL14|GPR29|MAP2K3|CCL20|TGFBR2|CXCL9|MAP3K3|TGFB2|IL6R|CXCR3|TGFBR1|CCL7|JAK2|PDCD10|CXCL12|BCL2|CMKBR6|IL6|IL1R1|DOCK4|HIF1A|SOD2|CCL5|CCL4 | 77 | 469|350|484|531|243|413|516|500|506|362|364|322|345|111|247|406|215|153|409|356|355|112|385|519|321|375|149|502|241|468|448|528|308|332|283|299|376|348|230|368|289|309|316|181|475|431|218|274|544|495|381|135|118 | 7.89E-19 |  | GO.0040017 | 1.647366072 | 0 |
| 71 | 24 | Reactome Pathways | | Interleukin-17 signaling | 3.92E-17 | MAPK1|NFKB1|IRAK2|MAP3K8|ATF2|IL17RB|MAP2K1|IL25|IL17F|MAP2K3|MAPK10|MAPKAPK2|TAB2|MAP3K7|IRAK1|TAB3|IL17RE|MAPK8|MAP2K7|MAPK9|MAP2K4|IKBKB|TRAF6|MAP2K6 | 77 | 469|529|258|165|189|497|292|478|367|528|380|268|184|358|434|206|465|282|236|250|517|526|315|255 | 8.73E-19 |  | HSA-448424 | 1.640671393 | 0 |
| 91 | 26 | Reactome Pathways | | TRAF6 mediated induction of NFkB and MAP kinases upon TLR7/8 or 9 activation | 4.18E-17 | MAPK1|NFKB1|IRAK2|MAP3K8|ATF2|MAP2K1|NKIRAS2|MAP2K3|MAPK10|MAPKAPK2|TAB2|MAP3K7|IRAK1|TLR4|TAB3|MAPK8|MAP2K7|MAP3K1|IRAK4|NKIRAS1|MAPK9|MAP2K4|TICAM2|IKBKB|TRAF6|MAP2K6 | 77 | 469|529|258|165|189|292|273|528|380|268|184|358|434|342|206|282|236|397|371|417|250|517|329|526|315|255 | 9.93E-19 |  | HSA-975138 | 1.637882372 | 0 |
| 491 | 52 | GO Process |  | chemotaxis | 5.98E-17 | CX3CL1|MAPK1|CCL1|CCL13|CXCL6|CCR7|PDGFRA|CCL21|PDGFRB|IL1B|ACKR3|CSF1R|CXCR5|CXCL3|CXCL5|DEFB1|MAP2K1|IL16|DEFB4A|ALCAM|CXCL11|CCR4|CXCL14|SMAD4|GPR29|BCL11B|CCRL2|CCR9|CCL20|CCL28|CXCL9|TGFB2|IL6R|CXCR3|CCL7|NFIB|LYST|CXCL12|TNFSF11|CMKBR6|IL6|CXCR4|CXCR6|DOCK4|IL10|ACKR2|DEFB4B|TNFRSF11A|CCL5|CCL16|ECSCR|CCL4 | 77 | 325|469|484|485|531|243|516|500|506|345|247|153|306|356|355|112|292|385|158|286|321|502|468|470|448|128|134|456|308|488|283|376|348|230|289|170|555|181|430|431|218|450|435|544|425|173|166|163|135|501|253|118 | 1.42E-18 |  | GO.0006935 | 1.622329882 | 0 |
| 676 | 61 | GO Process |  | regulation of defense response | 6.81E-17 | CX3CL1|TNFRSF1A|NFKB1|CRTAM|IL1RL1|CCR7|PIAS1|IRAK2|ARG2|IL1B|PDCD4|TNFSF4|IL17RB|RICTOR|IL22RA2|IL12A|SOCS5|PPARD|TNIP1|IFNLR1|SOCS1|SOCS3|SMAD3|LAMP1|IL17F|IFNAR2|IL20|MAPKAPK2|TAB2|MAP3K7|IRAK1|BCL10|TRAF3IP1|TLR4|TNFRSF1B|TAB3|IRF4|IL6ST|IL33|JAK2|TANK|TNFSF11|MAP3K1|BCL6|PPARA|IL6|IL1R1|TLR6|IRAK4|MAPKBP1|HLA-B|IL10|TICAM2|IKBKB|TRAF6|TRAF3|IKBKE|TNFRSF11A|MAP2K6|CCL5|TNFAIP3 | 77 | 325|351|529|151|336|243|554|258|518|345|311|383|497|302|272|519|226|227|538|341|460|246|241|360|367|452|161|268|184|358|434|323|474|342|276|206|169|391|346|309|314|430|397|310|491|218|274|305|371|298|114|425|329|526|315|557|548|163|255|135|164 | 1.64E-18 |  | GO.0031347 | 1.616685289 | 0 |
| 130 | 29 | GO Process |  | leukocyte chemotaxis | 1.27E-16 | CX3CL1|CCL1|CCL13|CXCL6|CCR7|CCL21|IL1B|CXCR5|CXCL3|IL16|CXCL11|GPR29|CCL20|CXCL9|TGFB2|IL6R|CXCR3|CCL7|LYST|CXCL12|TNFSF11|CMKBR6|IL6|CXCR4|IL10|TNFRSF11A|CCL5|CCL16|CCL4 | 77 | 325|484|485|531|243|500|345|306|356|385|321|448|308|283|376|348|230|289|555|181|430|431|218|450|425|163|135|501|118 | 3.07E-18 |  | GO.0030595 | 1.589619628 | 0 |
| 2416 | 126 | GO Process |  | regulation of developmental process | 1.67E-16 | CX3CL1|TNFRSF1A|TGFBR3|MAPK1|TGFB1|CCL13|NFKB1|CDKN1B|TGFB3|RHOQ|TNFSF9|LIF|PIAS1|DOCK7|PDGFRA|ARG2|PDGFRB|MAPK6|MMD|MSR1|SMAD7|SMAD2|RIMS2|SMURF2|IL1A|IL1B|IL7|ATF2|STAT3|CDK6|BNIP2|SOD1|DOCK5|PDCD4|DOCK1|TNFSF4|CSF1R|SMAD6|TNFRSF13C|TNFRSF21|TNFRSF11B|HIF1AN|NFATC3|LTBP3|MAP2K1|IL12A|CTLA4|IL13|SOCS5|IL7R|PPARD|CFLAR|IL1RAP|RHOJ|C8orf4|TNFRSF12A|CSF1|PAWR|SOCS1|SOCS3|SMAD3|PDCD1|BCL9L|CXCL14|IL17F|BCL11A|BAG5|SMAD4|MAP4K4|IKZF3|RBPJ|BCL11B|TGFBR2|MAP3K5|SMURF1|CXCL9|MAP3K3|TGFB2|IL20|IL6R|AGO3|AGO1|AGO4|TRAF3IP1|CXCR3|TGIF2|TLR4|TGFBR1|TNFSF13B|TNFRSF1B|BCOR|CCL7|NFIB|IRF4|IL6ST|JAK2|LOXL2|PDCD10|BCL2L11|ULK2|CXCL12|NFATC2|BLOC1S5|BCL2|TNFSF11|BCL6|PPARA|IL6|CXCR4|NFATC4|MAPK9|HLA-B|TNIK|IL10|SMAD1|IKBKB|TRAF6|DICER1|HIF1A|SMAD5|SOD2|PIAS2|NOS1|ECSCR|SOCS2|TNFAIP3 | 77 | 325|351|447|469|350|485|529|285|265|552|120|432|554|413|516|518|506|235|427|147|195|200|331|362|322|345|266|189|334|365|187|281|406|311|215|383|153|369|224|513|359|228|210|441|292|519|183|418|226|176|227|133|199|375|293|340|149|396|460|246|241|537|125|468|367|507|124|470|254|392|438|128|332|335|464|283|299|376|161|348|520|239|240|474|230|259|342|368|326|276|541|289|170|169|391|309|480|316|234|214|181|115|198|475|430|310|491|218|450|249|250|114|535|425|193|526|315|121|495|201|381|284|508|253|419|164 | 4.09E-18 |  | GO.0050793 | 1.577728353 | 0 |
| 101 | 26 | KEGG Pathways | | Chagas disease (American trypanosomiasis) | 1.73E-16 | TNFRSF1A|MAPK1|TGFB1|NFKB1|TGFB3|SMAD2|IL1B|IL12A|CFLAR|SMAD3|TGFBR2|MAPK10|TGFB2|IRAK1|TLR4|TGFBR1|MAPK8|IL6|TLR6|IRAK4|MAPK9|MAP2K4|IL10|IKBKB|TRAF6|CCL5 | 77 | 351|469|350|529|265|200|345|519|133|241|332|380|376|434|342|368|282|218|305|371|250|517|425|526|315|135 | 8.77E-18 |  | hsa05142 | 1.57619539 | 0 |
| 825 | 67 | GO Process |  | response to lipid | 1.86E-16 | RELT|TNFRSF1A|TGFBR3|MAPK1|TGFB1|CXCL6|NFKB1|CDKN1B|LOX|TGFB3|CCR7|PIAS1|IRAK2|CCL21|FDX1|PDGFRB|LOXL1|SMAD2|IL1B|STAT3|IRF8|TNFRSF10B|PDCD4|TNFSF4|AIFM1|CXCL3|CXCL5|TNFRSF21|TNFRSF11B|AVPR1A|IL12A|IL13|CXCL11|PPARD|CFLAR|TNIP1|CCL20|TGFBR2|CXCL9|TGFB2|MAPKAPK2|IRAK1|TNFRSF6B|TLR4|TGFBR1|TNFRSF1B|JAK2|BCL2L11|MAPK8|BCL2|CSF2RB|PPARA|IL6|NFATC4|NCOA2|IL10|TICAM2|TRAF6|IL22|LITAF|TXNIP|TNFRSF11A|PIAS2|CCL5|TNFRSF9|SOCS2|TNFAIP3 | 77 | 532|351|447|469|350|531|529|285|168|265|243|554|258|500|353|506|498|200|345|334|398|410|311|383|482|356|355|513|359|222|519|418|321|227|133|538|308|332|283|376|268|434|481|342|368|276|309|234|282|475|524|491|218|249|455|425|329|315|534|395|415|163|284|135|473|419|164 | 4.57E-18 |  | GO.0033993 | 1.573048706 | 0 |
| 469 | 50 | GO Process |  | positive regulation of cell motility | 2.14E-16 | MAPK1|TGFB1|CCL1|CXCL6|CCR7|DOCK7|PDGFRA|CCL21|PDGFRB|SMURF2|NOX4|IL1A|IL1B|CCAR1|ACKR3|DOCK5|DOCK1|CSF1R|BAG4|CXCL3|CXCL5|DEFB1|IL12A|CXCL11|RHOJ|CSF1|SMAD3|CXCL14|GPR29|MAP2K3|CCL20|TGFBR2|CXCL9|MAP3K3|TGFB2|IL6R|TGFBR1|CCL7|JAK2|PDCD10|CXCL12|BCL2|CMKBR6|IL6|IL1R1|DOCK4|HIF1A|SOD2|CCL5|CCL4 | 77 | 469|350|484|531|243|413|516|500|506|362|364|322|345|111|247|406|215|153|409|356|355|112|519|321|375|149|241|468|448|528|308|332|283|299|376|348|368|289|309|316|181|475|431|218|274|544|495|381|135|118 | 5.31E-18 |  | GO.2000147 | 1.566958623 | 0 |
| 134 | 29 | Reactome Pathways | | Interleukin-1 family signaling | 2.21E-16 | NFKB1|IL1RL1|IRAK2|IL36G|IL36B|MAP3K8|IL1A|IL1B|STAT3|MAP2K1|NKIRAS2|IL13|SMAD3|MAP3K3|TAB2|MAP3K7|IRAK1|TAB3|IL33|IL36RN|MAPK8|IL1R1|IL18R1|IRAK4|NKIRAS1|MAP2K4|IKBKB|TRAF6|MAP2K6 | 77 | 529|336|258|561|558|165|322|345|334|292|273|418|241|299|184|358|434|206|346|536|282|274|287|371|417|517|526|315|255 | 6.24E-18 |  | HSA-446652 | 1.565560773 | 0 |
| 452 | 49 | GO Process |  | positive regulation of cell migration | 2.51E-16 | MAPK1|TGFB1|CCL1|CXCL6|CCR7|DOCK7|PDGFRA|CCL21|PDGFRB|SMURF2|NOX4|IL1A|IL1B|CCAR1|ACKR3|DOCK5|DOCK1|CSF1R|BAG4|CXCL3|CXCL5|IL12A|CXCL11|RHOJ|CSF1|SMAD3|CXCL14|GPR29|MAP2K3|CCL20|TGFBR2|CXCL9|MAP3K3|TGFB2|IL6R|TGFBR1|CCL7|JAK2|PDCD10|CXCL12|BCL2|CMKBR6|IL6|IL1R1|DOCK4|HIF1A|SOD2|CCL5|CCL4 | 77 | 469|350|484|531|243|413|516|500|506|362|364|322|345|111|247|406|215|153|409|356|355|519|321|375|149|241|468|448|528|308|332|283|299|376|348|368|289|309|316|181|475|431|218|274|544|495|381|135|118 | 6.28E-18 |  | GO.0030335 | 1.560032628 | 0 |
| 149 | 30 | GO Process |  | activation of MAPK activity | 3.76E-16 | MAP4K5|MAPK1|TGFB3|MAP3K10|IRAK2|IL1B|SOD1|TGFA|MAP2K1|MAP3K11|MAP2K3|MAP3K5|MAPK10|KIAA1804|MAPKAPK2|TAB2|MAP3K7|IRAK1|TLR4|ZAK|TAB3|MAP2K7|TNFSF11|CXCR4|MAP3K2|MAP2K4|TRAF6|MAPKAPK5|MAP3K9|MAP2K6 | 77 | 451|469|265|489|258|345|281|279|292|439|528|335|380|190|268|184|358|434|342|264|206|236|430|450|374|517|315|370|290|255 | 9.47E-18 |  | GO.0000187 | 1.542481216 | 0 |
| 181 | 32 | KEGG Pathways | | Chemokine signaling pathway | 3.77E-16 | CX3CL1|MAPK1|CCL1|CCL13|CXCL6|NFKB1|CCR7|CCL21|STAT3|CXCR5|CXCL3|CXCL5|MAP2K1|CXCL11|CCR4|CXCL14|CCR9|CCL20|CCL28|CXCL9|CXCR3|CCL7|JAK2|CXCL12|CMKBR6|CXCR4|CXCR6|ITK|IKBKB|CCL5|CCL16|CCL4 | 77 | 325|469|484|485|531|529|243|500|334|306|356|355|292|321|502|468|456|308|488|283|230|289|309|181|431|450|435|556|526|135|501|118 | 2.11E-17 |  | hsa04062 | 1.542365865 | 0 |
| 142 | 29 | KEGG Pathways | | Hepatitis B | 3.97E-16 | MAPK1|TGFB1|NFKB1|CDKN1B|TGFB3|ATF2|STAT3|CDK6|NFATC3|MAP2K1|SMAD3|SMAD4|MAPK10|TGFB2|YWHAB|TLR4|TGFBR1|MAPK8|NFATC2|BCL2|MAP3K1|IL6|NFATC4|MAPK9|MAP2K4|TICAM2|IKBKB|APAF1|IKBKE | 77 | 469|350|529|285|265|189|334|365|210|292|241|470|380|376|440|342|368|282|115|475|397|218|249|250|517|329|526|148|548 | 2.42E-17 |  | hsa05161 | 1.540120949 | 0 |
| 109 | 26 | KEGG Pathways | | Toxoplasmosis | 6.55E-16 | TNFRSF1A|MAPK1|TGFB1|NFKB1|TGFB3|STAT3|BCL2L1|IL12A|SOCS1|MAP2K3|MAPK10|TGFB2|TAB2|MAP3K7|IRAK1|TLR4|JAK2|MAPK8|BCL2|IRAK4|MAPK9|HLA-DMB|IL10|IKBKB|TRAF6|MAP2K6 | 77 | 351|469|350|529|265|334|372|519|460|528|380|376|184|358|434|342|309|282|475|371|250|553|425|526|315|255 | 4.33E-17 |  | hsa05145 | 1.51837587 | 0 |
| 390 | 45 | GO Process |  | positive regulation of cytokine production | 7.11E-16 | CX3CL1|TGFB1|NFKB1|CRTAM|IL1RL1|CCR7|TRAF2|HILPDA|IL1A|IL1B|IL7|ATF2|IRF8|SOD1|IL9|TNFSF4|CSF1R|IL17RB|TNFRSF13C|IL12A|IL13|IL1RAP|SMAD3|IL17F|IRF5|CCL20|MAPKAPK2|IL6R|MAP3K7|IRAK1|BCL10|TLR4|IRF4|IL6ST|IL33|JAK2|IL6|IL1R1|IL18R1|NFATC4|TLR6|IL10|TICAM2|TRAF6|HIF1A | 77 | 325|350|529|151|336|243|444|462|322|345|266|189|398|281|295|383|153|497|224|519|418|199|241|367|160|308|268|348|358|434|323|342|169|391|346|309|218|274|287|249|305|425|329|315|495 | 1.81E-17 |  | GO.0001819 | 1.51481304 | 0 |
| 84 | 24 | Reactome Pathways | | MyD88 cascade initiated on plasma membrane | 7.38E-16 | MAPK1|NFKB1|IRAK2|MAP3K8|ATF2|MAP2K1|NKIRAS2|MAP2K3|MAPK10|MAPKAPK2|TAB2|MAP3K7|IRAK1|TAB3|MAPK8|MAP2K7|MAP3K1|IRAK4|NKIRAS1|MAPK9|MAP2K4|IKBKB|TRAF6|MAP2K6 | 77 | 469|529|258|165|189|292|273|528|380|268|184|358|434|206|282|236|397|371|417|250|517|526|315|255 | 2.19E-17 |  | HSA-975871 | 1.513194364 | 0 |
| 1427 | 90 | GO Process |  | response to oxygen-containing compound | 7.69E-16 | RELT|TNFRSF1A|TGFBR3|MAPK1|TGFB1|CXCL6|NFKB1|CDKN1B|SOGA1|TGFB3|RHOQ|TNFSF10|CCR7|TRAF2|IRAK2|PDGFRA|CCL21|FDX1|PDGFRB|LOXL1|SMAD2|NOX4|IL1B|ATF2|STAT3|PRDX5|IRF8|SOD1|ILDR2|TNFRSF10B|PDCD4|TNFSF4|AIFM1|CXCL3|CXCL5|TNFRSF21|TNFRSF11B|AVPR1A|BCL2L1|IL12A|IL13|CXCL11|PPARD|CFLAR|TNIP1|LAMTOR4|ATG7|IRF5|CCL20|TGFBR2|MAP3K5|CXCL9|TGFB2|MAPKAPK2|IRAK1|TNFRSF6B|TLR4|TGFBR1|TNFRSF1B|CCL7|JAK2|TXNRD3|PDCD10|MAPK8|CXCL12|BCL2|TXNRD2|CSF2RB|GATSL3|PPARA|IL6|NFATC4|TLR6|MAPK9|NCOA2|OXR1|MAP2K4|IL10|TICAM2|TRAF6|SOD2|LITAF|TXNIP|TNFRSF11A|MAP2K6|CCL5|TNFRSF9|SOCS2|TNFAIP3|SOCS7 | 77 | 532|351|447|469|350|531|529|285|317|265|552|202|243|444|258|516|500|353|506|498|200|364|345|189|334|208|398|281|237|410|311|383|482|356|355|513|359|222|372|519|418|321|227|133|538|188|119|160|308|332|335|283|376|268|434|481|342|368|276|289|309|263|316|282|181|475|476|524|288|491|218|249|305|250|455|144|517|425|329|315|381|395|415|163|255|135|473|419|164|186 | 1.97E-17 |  | GO.1901700 | 1.511407366 | 0 |
| 1483 | 92 | GO Process |  | negative regulation of response to stimulus | 8.37E-16 | CX3CL1|TNFRSF1A|TGFBR3|TGFB1|TNFAIP1|NFKB1|IL1RL1|TGFB3|TNFSF10|TRAF2|LIF|BCL2L2|PXDN|PDGFRA|BCL2L10|ARG2|SMAD7|SMAD2|SMURF2|IL1A|IL1B|IL7|CDK6|ACKR3|TNFRSF10B|PDCD4|TNFSF4|DEPTOR|SMAD6|CREBRF|IL22RA2|HIF1AN|BCL2L1|CTLA4|SOCS5|IL7R|PPARD|CFLAR|TNIP1|C8orf4|PAWR|SOCS1|SOCS3|SMAD3|BCL9L|BAG5|SMAD4|MAP4K4|ATG7|TGFBR2|SMURF1|MAP3K3|TGFB2|MCL1|IGSF1|YWHAB|TRAF3IP1|TLR4|TGFBR1|TNFRSF1B|IRF4|IL6ST|IL33|TANK|IL36RN|SOCS4|CXCL12|SOCS6|BCL2|GATSL3|BCL6|PPARA|IL6|LTBP1|NFATC4|TLR6|MAPKBP1|HLA-B|OXR1|IL10|TICAM2|HIF1A|TNFAIP8L1|SOD2|MAPKAPK5|LITAF|PIAS2|CCL5|SOCS2|TNFAIP3|SOCS7|GATSL2 | 77 | 325|351|447|350|280|529|336|265|202|444|432|551|543|516|550|518|195|200|362|322|345|266|365|247|410|311|383|408|369|296|272|228|372|183|226|176|227|133|538|293|396|460|246|241|125|124|470|254|119|332|464|299|376|278|132|440|474|342|368|276|169|391|346|314|536|523|181|244|475|288|310|491|218|154|249|305|298|114|144|425|329|495|453|381|370|395|284|135|419|164|186|402 | 2.16E-17 |  | GO.0048585 | 1.507727454 | 0 |
| 125 | 27 | KEGG Pathways | | Autophagy - animal | 1.30E-15 | MAPK1|ATG14|ATG10|ATG3|DEPTOR|MAP2K1|BCL2L1|ATG4D|CFLAR|LAMP1|ATG7|MAPK10|ATG2B|ATG5|MAP3K7|ATG16L1|ULK2|MAPK8|BCL2|ATG9A|MAPK9|LAMP2|ATG12|AMBRA1|ATG13|TRAF6|HIF1A | 77 | 469|150|155|113|408|292|372|213|133|360|119|380|389|312|358|223|214|282|475|257|250|304|421|131|559|315|495 | 9.21E-17 |  | hsa04140 | 1.488605665 | 0 |
| 14652 | 412 | GO Process |  | cellular process | 1.30E-15 | CX3CL1|MAP4K5|RELT|NOX3|TNFRSF1A|TGFBR3|MAPK1|TGFB1|CCL1|CCL13|TNFAIP1|CXCL6|NFKB1|CRTAM|CDKN1B|EXOC2|LOX|NKTR|IL1RL1|BCL9|SOGA1|TGFB3|RHOQ|TNFSF10|RASL11A|EEPD1|TNFSF9|TMX4|CCR7|ATG14|TRAF2|LIF|PIAS1|ISLR|TXNDC17|BCL2L2|DOCK7|LILRA1|NDUFA10|PXDN|MAP3K10|EXOC4|IRAK2|PDGFRA|HILPDA|DOCK10|TBRG4|IL36G|IL36B|CCL21|IER3|FDX1|BCL2L10|TRAF5|ARG2|PDGFRB|MAPK6|LOXL1|FAF2|MMD|MSR1|WDR59|SMAD7|SMAD2|RIMS2|TRAF4|SMURF2|MAP3K8|NOX4|IL1A|IL1B|ITPKC|IL7|MAP4K3|ATF2|IL11|STAT3|MAP3K13|PECR|PRDX5|CDK6|CCAR1|HPS5|DOCK3|ETNK1|BNIP2|IGSF6|IRF8|SOD1|IL22RA1|ILDR2|ACKR3|IL9|DOCK11|TNFRSF10B|DOCK5|ENDOD1|PDCD4|DOCK1|TNFSF4|EPG5|ATG10|TXNDC11|ATG3|DEPTOR|CSF1R|AIFM1|BAG4|GATAD1|IL17RB|SMAD6|TNFRSF13C|CXCR5|WDR24|PEX13|TGFA|TRAT1|CXCL3|CXCL5|SNIP1|IL17RD|RICTOR|TNFRSF21|CREBRF|IL22RA2|TNFRSF11B|DEFB1|HIF1AN|AVPR1A|HPS6|TMX3|NFATC3|MAP2K1|BCL2L1|MAEA|IL16|IL12A|DEFB4A|NKIRAS2|CTLA4|IL13|SOCS5|ALCAM|IL7R|AVEN|CXCL11|MAP3K11|PPARD|ATG4D|OXSR1|CFLAR|IL1RAP|RHOJ|TNIP1|EEA1|PRKRA|ICOS|C8orf4|ISG20L2|AATK|NFATC2IP|PRX|TNFRSF12A|CSF1|IFNLR1|TGIF1|TNFAIP8L3|PAWR|IL25|SOCS1|PIWIL3|SOCS3|IKZF1|AEN|CCR4|SMAD3|LAMP1|PDCD1|BCL9L|CXCL14|IL17F|IL21R|BCL11A|C1QTNF6|BAG5|KIR2DL4|MIOS|SMAD4|NECAP1|LAMTOR4|MAP4K4|GPR29|IFNAR2|IKZF3|MAP2K3|RBPJ|MAP3K15|ATG7|AREL1|PEX2|BCL11B|IRF5|CCRL2|FXR1|CCR9|TXNDC15|CCL20|TGFBR2|MAP3K5|MAPK10|CPLX2|ATG2B|CCL28|GPX6|SMURF1|CXCL9|MAP3K3|ILF2|IRF2BP2|KIAA1804|TGFB2|IRF6|TAGAP|IL20|MAPKAPK2|TAB2|PEX3|HIVEP2|PEX19|THEMIS|IL6R|GATAD2B|IKZF5|MCL1|ATG5|BAG3|CACUL1|PEX11B|MAP3K7|IGSF3|IRAK1BP1|IRAK1|TNFRSF6B|BCL10|PI4K2A|BAG2|EXOSC1|IGSF1|LCOR|IFIT3|IFIT2|IL1RAPL2|YWHAB|AGO3|AGO1|AGO4|TRAF3IP1|CXCR3|TGIF2|TLR4|IL2RG|TXNDC8|HLA-DQB1|TGFBR1|ZAK|MAP3K19|TNFSF13B|TNFRSF1B|DOCK9|HIF3A|PLCXD3|BCOR|CCL7|TAB3|BCAP29|TXNDC5|CAAP1|BNIP3L|NFIB|IRF4|IL6ST|IL33|JAK2|TNFRSF19|TXNRD3|IL17RE|LOXL2|EXOSC8|LYST|IL17REL|ATG16L1|MAP3K4|TANK|PDCD10|IL36RN|BCL2L11|BCL2L15|FDX1L|SUOX|PRC1|CIAPIN1|AKTIP|SOCS4|ULK2|MAPK8|CXCL12|NFATC2|PDXDC1|IRF9|BLOC1S5|SOCS6|MAP2K7|BCL2|TNFSF11|MAP3K1|MAPK4|TXNRD2|CMKBR6|CSF2RB|GATSL3|BCL6|RASSF8|PPARA|IL6|LTBP1|IL1R1|ATG9A|CXCR4|IL18R1|MAP3K2|NFATC4|TLR6|IRAK4|PEX5|MAPKBP1|TMX1|EIF4E3|NKIRAS1|MAPK9|CXCR6|OSGIN2|ITK|HLA-DMB|HLA-B|TNIK|NCOA2|VSIG1|ILF3|DOCK4|OXR1|LAMP2|TBRG1|MAP2K4|LMLN|BCL7B|PDCD6IP|IL10|IKZF2|TICAM2|TGFBI|ACKR2|PEX5L|LAT2|DEFB4B|ATG12|SMAD1|CDKN2AIP|TNFAIP8|IKBKB|API5|AMBRA1|ATG13|TRAF6|BCLAF1|DICER1|BCORL1|HIF1A|SMAD5|NKRF|IL22|TET2|RASSF3|SOD2|APAF1|MAP3K12|MAPKAPK5|JKAMP|MAP3K9|MOAP1|TRAF3|NFAT5|LITAF|TXNIP|IKBKE|TNFRSF11A|IER2|PIAS2|ERC1|MAP2K6|CCL5|MR1|NOS1|CCL16|TNFRSF9|ECSCR|PANO|SOCS2|TNFAIP3|SOCS7|CCL4|PEX12|MAP3K14 | 77 | 325|451|532|203|351|447|469|350|484|485|280|531|529|151|285|339|168|117|336|175|317|265|552|202|205|197|120|301|243|150|444|432|554|477|140|551|413|344|275|543|489|159|258|516|462|505|496|561|558|500|461|353|550|142|518|506|235|498|458|427|147|127|195|200|331|194|362|165|364|322|345|420|266|141|189|126|334|490|436|208|365|111|366|225|191|187|546|398|281|211|237|247|295|422|410|406|204|311|215|383|361|155|156|113|408|153|482|409|530|497|369|224|306|260|152|279|527|356|355|337|136|302|513|296|272|359|112|228|222|209|122|210|292|372|207|385|519|158|273|183|418|226|286|176|145|321|439|227|213|504|133|199|375|538|146|196|167|293|429|560|347|319|340|149|341|143|248|396|478|460|182|246|220|547|502|241|360|537|125|468|367|217|507|123|124|515|525|470|542|188|254|448|452|392|528|438|384|119|437|269|128|160|134|245|456|494|308|332|335|380|216|389|488|388|464|283|299|443|178|190|376|487|139|161|268|184|423|433|521|349|348|414|412|278|312|318|522|343|358|270|400|434|481|323|399|116|137|132|233|404|394|252|440|520|239|240|474|230|259|342|563|238|109|368|264|352|326|276|300|510|407|541|289|206|129|459|428|499|170|169|391|346|309|256|263|465|480|297|555|479|223|466|314|316|536|234|320|512|262|511|472|424|523|214|282|181|115|330|492|198|244|236|475|430|397|231|476|431|524|288|310|549|491|218|154|274|257|450|287|374|249|305|371|514|298|390|403|417|250|435|446|556|553|114|535|455|378|110|544|144|304|445|517|333|174|307|425|486|329|157|173|328|467|166|421|193|324|185|526|463|131|559|315|180|121|387|495|201|291|534|294|221|381|148|426|370|382|290|138|557|393|395|415|548|163|354|284|401|255|135|540|508|501|473|253|172|419|164|186|118|229|171 | 3.37E-17 |  | GO.0009987 | 1.488605665 | 0 |
| 63 | 22 | GO Function | | chemokine receptor binding | 1.31E-15 | CX3CL1|CCL1|CCL13|CXCL6|CCL21|STAT3|CXCL3|CXCL5|DEFB1|DEFB4A|CXCL11|CXCL14|CCRL2|CCL20|CCL28|CXCL9|CCL7|CXCL12|DEFB4B|CCL5|CCL16|CCL4 | 77 | 325|484|485|531|500|334|356|355|112|158|321|468|134|308|488|283|289|181|166|135|501|118 | 1.42E-17 |  | GO.0042379 | 1.48827287 | 0 |
| 483 | 50 | GO Function | | receptor regulator activity | 1.31E-15 | CX3CL1|TGFB1|CCL1|CCL13|CXCL6|TGFB3|TNFSF10|TNFSF9|LIF|PXDN|IL36G|IL36B|CCL21|IL1A|IL1B|IL7|IL11|IL9|TNFSF4|TGFA|CXCL3|CXCL5|TNFRSF11B|IL16|IL12A|IL13|CXCL11|CSF1|IL25|CXCL14|IL17F|CCL20|CCL28|CXCL9|TGFB2|IL20|IGSF1|TNFSF13B|CCL7|IL33|IL36RN|CXCL12|TNFSF11|IL6|OSGIN2|IL10|IL22|CCL5|CCL16|CCL4 | 77 | 325|350|484|485|531|265|202|120|432|543|561|558|500|322|345|266|126|295|383|279|356|355|359|385|519|418|321|149|478|468|367|308|488|283|376|161|132|326|289|346|536|181|430|218|446|425|534|135|501|118 | 1.61E-17 |  | GO.0030545 | 1.48827287 | 0 |
| 444 | 48 | GO Function | | protein serine/threonine kinase activity | 1.31E-15 | MAP4K5|TGFBR3|MAPK1|CDKN1B|MAP3K10|IRAK2|MAPK6|MAP3K8|MAP4K3|MAP3K13|CDK6|MAP2K1|MAP3K11|OXSR1|AATK|MAP4K4|MAP2K3|MAP3K15|TGFBR2|MAP3K5|MAPK10|MAP3K3|KIAA1804|MAPKAPK2|MAP3K7|IRAK1|TGFBR1|ZAK|MAP3K19|MAP3K4|ULK2|MAPK8|MAP2K7|MAP3K1|MAPK4|LTBP1|MAP3K2|IRAK4|MAPK9|TNIK|MAP2K4|IKBKB|MAP3K12|MAPKAPK5|MAP3K9|IKBKE|MAP2K6|MAP3K14 | 77 | 451|447|469|285|489|258|235|165|141|490|365|292|439|504|560|254|528|384|332|335|380|299|190|268|358|434|368|264|352|466|214|282|236|397|231|154|374|371|250|535|517|526|426|370|290|548|255|171 | 1.55E-17 |  | GO.0004674 | 1.48827287 | 0 |
| 166 | 30 | KEGG Pathways | | NOD-like receptor signaling pathway | 1.68E-15 | MAPK1|NFKB1|TRAF2|TRAF5|IL1B|CXCL3|BCL2L1|IFNAR2|MAPK10|TAB2|ATG5|MAP3K7|TLR4|TAB3|ATG16L1|TANK|MAPK8|IRF9|BCL2|IL6|IRAK4|MAPK9|ATG12|IKBKB|TRAF6|TRAF3|TXNIP|IKBKE|CCL5|TNFAIP3 | 77 | 469|529|444|142|345|356|372|452|380|184|312|358|342|206|223|314|282|492|475|218|371|250|421|526|315|557|415|548|135|164 | 1.28E-16 |  | hsa04621 | 1.477469072 | 0 |
| 635 | 57 | GO Function | | protein kinase activity | 1.95E-15 | MAP4K5|TGFBR3|MAPK1|CDKN1B|MAP3K10|IRAK2|PDGFRA|TBRG4|PDGFRB|MAPK6|MMD|MAP3K8|MAP4K3|MAP3K13|CDK6|CSF1R|MAP2K1|MAP3K11|OXSR1|AATK|MAP4K4|MAP2K3|MAP3K15|TGFBR2|MAP3K5|MAPK10|MAP3K3|KIAA1804|MAPKAPK2|MAP3K7|IRAK1|TGFBR1|ZAK|MAP3K19|JAK2|MAP3K4|ULK2|MAPK8|MAP2K7|MAP3K1|MAPK4|CSF2RB|LTBP1|MAP3K2|IRAK4|MAPK9|ITK|TNIK|MAP2K4|IKBKB|MAP3K12|MAPKAPK5|MAP3K9|IKBKE|MAP2K6|CCL5|MAP3K14 | 77 | 451|447|469|285|489|258|516|496|506|235|427|165|141|490|365|153|292|439|504|560|254|528|384|332|335|380|299|190|268|358|434|368|264|352|309|466|214|282|236|397|231|524|154|374|371|250|556|535|517|526|426|370|290|548|255|135|171 | 3.04E-17 |  | GO.0004672 | 1.470996539 | 0 |
| 183 | 31 | KEGG Pathways | | Kaposi's sarcoma-associated herpesvirus infection | 2.40E-15 | TNFRSF1A|MAPK1|NFKB1|ATG14|TRAF2|STAT3|CDK6|ATG3|CXCL3|NFATC3|MAP2K1|CCR4|IFNAR2|MAPK10|MAPKAPK2|IL6ST|JAK2|MAPK8|NFATC2|IRF9|MAP2K7|IL6|NFATC4|MAPK9|HLA-B|MAP2K4|IKBKB|HIF1A|TRAF3|IKBKE|MAP2K6 | 77 | 351|469|529|150|444|334|365|113|356|210|292|502|452|380|268|391|309|282|115|492|236|218|249|250|114|517|526|495|557|548|255 | 1.95E-16 |  | hsa05167 | 1.461978876 | 0 |
| 458 | 48 | GO Function | | receptor ligand activity | 2.79E-15 | CX3CL1|TGFB1|CCL1|CCL13|CXCL6|TGFB3|TNFSF10|TNFSF9|LIF|IL36G|IL36B|CCL21|IL1A|IL1B|IL7|IL11|IL9|TNFSF4|TGFA|CXCL3|CXCL5|TNFRSF11B|IL16|IL12A|IL13|CXCL11|CSF1|IL25|CXCL14|IL17F|CCL20|CCL28|CXCL9|TGFB2|IL20|TNFSF13B|CCL7|IL33|IL36RN|CXCL12|TNFSF11|IL6|OSGIN2|IL10|IL22|CCL5|CCL16|CCL4 | 77 | 325|350|484|485|531|265|202|120|432|561|558|500|322|345|266|126|295|383|279|356|355|359|385|519|418|321|149|478|468|367|308|488|283|376|161|326|289|346|536|181|430|218|446|425|534|135|501|118 | 4.78E-17 |  | GO.0048018 | 1.45543958 | 0 |
| 81 | 23 | GO Process |  | tumor necrosis factor-mediated signaling pathway | 4.40E-15 | RELT|TNFRSF1A|TNFSF9|TRAF2|TXNDC17|TNFRSF10B|TNFSF4|BAG4|TNFRSF13C|TNFRSF21|TNFRSF11B|TNFRSF12A|TNFRSF6B|TNFSF13B|TNFRSF1B|JAK2|TNFRSF19|TNFSF11|IKBKB|TRAF3|TNFRSF11A|TNFRSF9|MAP3K14 | 77 | 532|351|120|444|140|410|383|409|224|513|359|340|481|326|276|309|256|430|526|557|163|473|171 | 1.15E-16 |  | GO.0033209 | 1.435654732 | 0 |
| 767 | 62 | GO Function | | phosphotransferase activity, alcohol group as acceptor | 6.15E-15 | MAP4K5|TGFBR3|MAPK1|CDKN1B|MAP3K10|IRAK2|PDGFRA|TBRG4|PDGFRB|MAPK6|MMD|MAP3K8|ITPKC|MAP4K3|MAP3K13|CDK6|ETNK1|CSF1R|TRAT1|MAP2K1|MAP3K11|OXSR1|ICOS|AATK|MAP4K4|MAP2K3|MAP3K15|TGFBR2|MAP3K5|MAPK10|MAP3K3|KIAA1804|MAPKAPK2|MAP3K7|IRAK1|PI4K2A|TGFBR1|ZAK|MAP3K19|JAK2|MAP3K4|ULK2|MAPK8|MAP2K7|MAP3K1|MAPK4|CSF2RB|LTBP1|MAP3K2|IRAK4|MAPK9|ITK|TNIK|MAP2K4|IKBKB|MAP3K12|MAPKAPK5|MAP3K9|IKBKE|MAP2K6|CCL5|MAP3K14 | 77 | 451|447|469|285|489|258|516|496|506|235|427|165|420|141|490|365|191|153|527|292|439|504|167|560|254|528|384|332|335|380|299|190|268|358|434|399|368|264|352|309|466|214|282|236|397|231|524|154|374|371|250|556|535|517|526|426|370|290|548|255|135|171 | 1.15E-16 |  | GO.0016773 | 1.421112488 | 0 |
| 387 | 44 | UniProt Keywords | | Serine/threonine-protein kinase | 6.40E-15 | MAP4K5|MAPK1|MAP3K10|MAPK6|MAP3K8|MAP4K3|MAP3K13|CDK6|MAP2K1|MAP3K11|OXSR1|AATK|MAP4K4|MAP2K3|MAP3K15|TGFBR2|MAP3K5|MAPK10|MAP3K3|KIAA1804|MAPKAPK2|MAP3K7|IRAK1|TGFBR1|ZAK|MAP3K19|MAP3K4|ULK2|MAPK8|MAP2K7|MAP3K1|MAPK4|MAP3K2|IRAK4|MAPK9|TNIK|MAP2K4|IKBKB|MAP3K12|MAPKAPK5|MAP3K9|IKBKE|MAP2K6|MAP3K14 | 77 | 451|469|489|235|165|141|490|365|292|439|504|560|254|528|384|332|335|380|299|190|268|358|434|368|264|352|466|214|282|236|397|231|374|371|250|535|517|526|426|370|290|548|255|171 | 6.91E-17 |  | KW-0723 | 1.419382003 | 0 |
| 308 | 39 | GO Process |  | positive regulation of cell activation | 6.51E-15 | TGFB1|IL1RL1|TNFSF9|CCR7|CCL21|PDGFRB|MAP3K8|IL1B|IL7|TNFSF4|TNFRSF13C|IL12A|CTLA4|IL13|SOCS5|IL7R|ICOS|SOCS1|LAMP1|BTLA|PDCD1|TGFBR2|BCL10|TLR4|TNFSF13B|IL6ST|IL33|JAK2|NFATC2|PDCD1LG2|BCL2|TNFSF11|BCL6|IL6|TLR6|HLA-DMB|IL10|TRAF6|CCL5 | 77 | 350|336|120|243|500|506|165|345|266|383|224|519|183|418|226|176|167|460|360|449|537|332|323|342|326|391|346|309|115|373|475|430|310|218|305|553|425|315|135 | 1.71E-16 |  | GO.0050867 | 1.418641901 | 0 |
| 278 | 37 | GO Process |  | regulation of leukocyte cell-cell adhesion | 9.45E-15 | TGFB1|TNFSF9|CCR7|CCL21|ARG2|SMAD7|MAP3K8|IL1B|IL7|TNFSF4|TNFRSF13C|TNFRSF21|IL12A|CTLA4|SOCS5|IL7R|ICOS|PAWR|SOCS1|BTLA|PDCD1|TGFBR2|CCL28|BCL10|TNFSF13B|IL6ST|CXCL12|PDCD1LG2|SOCS6|TNFSF11|BCL6|PPARA|IL6|HLA-DMB|IL10|TRAF6|CCL5 | 77 | 350|120|243|500|518|195|165|345|266|383|224|513|519|183|226|176|167|396|460|449|537|332|488|323|326|391|181|373|244|430|310|491|218|553|425|315|135 | 2.51E-16 |  | GO.1903037 | 1.402456819 | 0 |
| 484 | 50 | InterPro Domains | | Protein kinase domain | 1.03E-14 | MAP4K5|MAPK1|MAP3K10|IRAK2|PDGFRA|PDGFRB|MAPK6|MAP3K8|MAP4K3|MAP3K13|CDK6|CSF1R|MAP2K1|MAP3K11|OXSR1|AATK|MAP4K4|MAP2K3|MAP3K15|TGFBR2|MAP3K5|MAPK10|MAP3K3|KIAA1804|MAPKAPK2|MAP3K7|IRAK1|TGFBR1|ZAK|MAP3K19|JAK2|MAP3K4|ULK2|MAPK8|MAP2K7|MAP3K1|MAPK4|MAP3K2|IRAK4|MAPK9|ITK|TNIK|MAP2K4|IKBKB|MAP3K12|MAPKAPK5|MAP3K9|IKBKE|MAP2K6|MAP3K14 | 77 | 451|469|489|258|516|506|235|165|141|490|365|153|292|439|504|560|254|528|384|332|335|380|299|190|268|358|434|368|264|352|309|466|214|282|236|397|231|374|371|250|556|535|517|526|426|370|290|548|255|171 | 1.74E-17 |  | IPR000719 | 1.398716278 | 0 |
| 1695 | 97 | GO Process |  | regulation of cell differentiation | 1.35E-14 | CX3CL1|TNFRSF1A|MAPK1|TGFB1|NFKB1|CDKN1B|TGFB3|TNFSF9|LIF|PIAS1|DOCK7|PDGFRA|MAPK6|MMD|MSR1|SMAD7|SMAD2|RIMS2|IL1B|IL7|STAT3|CDK6|BNIP2|SOD1|DOCK5|PDCD4|DOCK1|TNFSF4|SMAD6|TNFRSF21|HIF1AN|NFATC3|LTBP3|MAP2K1|IL12A|CTLA4|IL13|SOCS5|IL7R|PPARD|CFLAR|IL1RAP|TNFRSF12A|CSF1|SOCS1|SOCS3|SMAD3|BCL9L|CXCL14|BCL11A|BAG5|SMAD4|MAP4K4|IKZF3|RBPJ|BCL11B|TGFBR2|MAP3K5|SMURF1|CXCL9|TGFB2|IL20|IL6R|AGO3|AGO1|AGO4|TGIF2|TLR4|TGFBR1|IRF4|IL6ST|JAK2|LOXL2|ULK2|CXCL12|NFATC2|BLOC1S5|BCL2|TNFSF11|BCL6|PPARA|IL6|CXCR4|NFATC4|MAPK9|HLA-B|TNIK|SMAD1|IKBKB|TRAF6|DICER1|HIF1A|SMAD5|SOD2|PIAS2|NOS1|SOCS2 | 77 | 325|351|469|350|529|285|265|120|432|554|413|516|235|427|147|195|200|331|345|266|334|365|187|281|406|311|215|383|369|513|228|210|441|292|519|183|418|226|176|227|133|199|340|149|460|246|241|125|468|507|124|470|254|392|438|128|332|335|464|283|376|161|348|520|239|240|259|342|368|169|391|309|480|214|181|115|198|475|430|310|491|218|450|249|250|114|535|193|526|315|121|495|201|381|284|508|419 | 3.61E-16 |  | GO.0045595 | 1.386966623 | 0 |
| 168 | 29 | KEGG Pathways | | Influenza A | 1.40E-14 | TNFRSF1A|MAPK1|NFKB1|TNFSF10|IL1A|IL1B|ATF2|TNFRSF10B|MAP2K1|IL12A|SOCS3|IFNAR2|MAP2K3|MAPK10|TLR4|IL33|JAK2|MAPK8|IRF9|MAP2K7|IL6|IRAK4|MAPK9|HLA-DMB|MAP2K4|IKBKB|IKBKE|MAP2K6|CCL5 | 77 | 351|469|529|202|322|345|189|410|292|519|246|452|528|380|342|346|309|282|492|236|218|371|250|553|517|526|548|255|135 | 1.21E-15 |  | hsa05164 | 1.385387196 | 0 |
| 506 | 49 | GO Process |  | regulation of cell activation | 1.47E-14 | TGFB1|IL1RL1|TNFSF9|CCR7|PDGFRA|CCL21|ARG2|PDGFRB|SMAD7|MAP3K8|IL1B|IL7|SOD1|TNFSF4|TNFRSF13C|TNFRSF21|IL12A|CTLA4|IL13|SOCS5|IL7R|ICOS|PAWR|SOCS1|LAMP1|BTLA|PDCD1|IKZF3|TGFBR2|BCL10|TLR4|TNFSF13B|IRF4|IL6ST|IL33|JAK2|NFATC2|PDCD1LG2|SOCS6|BCL2|TNFSF11|BCL6|IL6|TLR6|HLA-DMB|IL10|TRAF6|CCL5|TNFAIP3 | 77 | 350|336|120|243|516|500|518|506|195|165|345|266|281|383|224|513|519|183|418|226|176|167|396|460|360|449|537|392|332|323|342|326|169|391|346|309|115|373|244|475|430|310|218|305|553|425|315|135|164 | 3.99E-16 |  | GO.0050865 | 1.383268267 | 0 |
| 589 | 53 | GO Process |  | positive regulation of immune response | 1.47E-14 | MAPK1|TGFB1|NFKB1|CRTAM|CCR7|TRAF2|IRAK2|IL1B|DOCK1|TNFSF4|TNFRSF13C|TRAT1|TNFRSF21|IL12A|CTLA4|IL13|SOCS5|TNIP1|LAMP1|TGFB2|MAPKAPK2|TAB2|THEMIS|MAP3K7|IRAK1|BCL10|TLR4|HLA-DQB1|TNFSF13B|TAB3|IL6ST|IL33|TANK|NFATC2|BCL2|MAP3K1|IL6|IL1R1|IL18R1|TLR6|IRAK4|ITK|HLA-DMB|HLA-B|TICAM2|LAT2|IKBKB|TRAF6|TRAF3|IKBKE|MAP2K6|CCL5|TNFAIP3 | 77 | 469|350|529|151|243|444|258|345|215|383|224|527|513|519|183|418|226|538|360|376|268|184|349|358|434|323|342|109|326|206|391|346|314|115|475|397|218|274|287|305|371|556|553|114|329|467|526|315|557|548|255|135|164 | 3.97E-16 |  | GO.0050778 | 1.383268267 | 0 |
| 62 | 20 | KEGG Pathways | | Inflammatory bowel disease (IBD) | 1.66E-14 | TGFB1|NFKB1|TGFB3|SMAD2|IL1A|IL1B|STAT3|IL12A|IL13|SMAD3|IL17F|IL21R|TGFB2|TLR4|IL2RG|IL6|IL18R1|HLA-DMB|IL10|IL22 | 77 | 350|529|265|200|322|345|334|519|418|241|367|217|376|342|563|218|287|553|425|534 | 1.51E-15 |  | hsa05321 | 1.377989191 | 0 |
| 26 | 16 | GO Function | | MAP kinase kinase kinase activity | 1.87E-14 | MAP3K10|MAP3K8|MAP3K13|MAP3K11|MAP3K15|MAP3K5|MAP3K3|KIAA1804|MAP3K7|ZAK|MAP3K4|MAP3K1|MAP3K2|MAP3K12|MAP3K9|MAP3K14 | 77 | 489|165|490|439|384|335|299|190|358|264|466|397|374|426|290|171 | 3.78E-16 |  | GO.0004709 | 1.372815839 | 0 |
| 2197 | 114 | GO Function | | enzyme binding | 1.88E-14 | MAPK1|TGFB1|TNFAIP1|CDKN1B|EXOC2|ATG14|TRAF2|PIAS1|DOCK7|EXOC4|XPO4|PDGFRA|DOCK10|BCL2L10|TRAF5|PDGFRB|MAPK6|FAF2|SMAD7|SMAD2|RIMS2|TRAF4|NOX4|ATF2|STAT3|MAP3K13|DOCK3|SOD1|DOCK11|DOCK5|DOCK1|ATG3|CSF1R|BAG4|SMAD6|TRAT1|RICTOR|AVPR1A|HPS6|BCL2L1|SOCS5|MAP3K11|CFLAR|IL1RAP|TNIP1|PRKRA|PAWR|SOCS1|SMAD3|LAMP1|BAG5|IFNAR2|MAP2K3|TGFBR2|MAP3K5|NOS1AP|TAGAP|MAPKAPK2|PEX19|IL6R|CACUL1|MAP3K7|MTCP1|SIKE1|BCL10|BAG2|YWHAB|AGO1|IL2RG|TNFRSF1B|DOCK9|BCOR|JAK2|ATG16L1|TANK|PDCD10|BCL2L11|PRC1|AKTIP|MAPK8|NFATC2|MAP2K7|BCL2|MAP3K1|MAPK4|CSF2RB|PPARA|IL1R1|CXCR4|MAP3K2|PEX5|DOCK4|LAMP2|MAP2K4|PEX5L|SMAD1|IKBKB|AMBRA1|ATG13|TRAF6|HIF1A|SMAD5|MAP3K12|MAPKAPK5|JKAMP|MOAP1|TRAF3|TXNIP|IKBKE|PIAS2|ERC1|MAP2K6|JAKMIP2|TNFAIP3 | 77 | 469|350|280|285|339|150|444|554|413|159|386|516|505|550|142|506|235|458|195|200|331|194|364|189|334|490|225|281|422|406|215|113|153|409|369|527|302|222|209|372|226|439|133|199|538|196|396|460|241|360|124|452|528|332|335|212|139|268|521|348|522|358|162|338|323|116|440|239|563|276|300|541|309|223|314|316|234|511|424|282|115|236|475|397|231|524|491|274|450|374|514|544|304|517|328|193|526|131|559|315|495|201|426|370|382|138|557|415|548|284|401|255|251|164 | 4.09E-16 |  | GO.0019899 | 1.372584215 | 0 |
| 618 | 54 | GO Process |  | regulation of cellular response to stress | 2.36E-14 | MAP4K5|MAPK1|CCR7|TRAF2|MAP3K10|CCL21|IER3|ARG2|TRAF4|IL1B|CDK6|SOD1|ACKR3|PDCD4|BAG4|CREBRF|MAP2K1|BCL2L1|MAP3K11|PAWR|BAG5|MAP4K4|ATG7|MAP3K5|MAP3K3|KIAA1804|TGFB2|MAPKAPK2|MCL1|BAG3|MAP3K7|BAG2|TLR4|ZAK|TNFRSF19|MAP3K4|PDCD10|BCL2L11|CXCL12|MAP2K7|TNFSF11|BCL6|MAP3K2|TLR6|TNIK|OXR1|MAP2K4|IL10|TRAF6|BCLAF1|HIF1A|SOD2|MAP3K9|TNFRSF11A | 77 | 451|469|243|444|489|500|461|518|194|345|365|281|247|311|409|296|292|372|439|396|124|254|119|335|299|190|376|268|278|318|358|116|342|264|256|466|316|234|181|236|430|310|374|305|535|144|517|425|315|180|495|381|290|163 | 6.46E-16 |  | GO.0080135 | 1.3627088 | 0 |
| 124 | 26 | GO Process |  | cellular response to interleukin-1 | 2.36E-14 | CX3CL1|CCL1|CCL13|NFKB1|IRAK2|CCL21|MAP3K8|IL1A|IL1B|IL1RAP|CCL20|MAP3K3|TAB2|MAP3K7|IRAK1|CCL7|TAB3|TANK|IL1R1|IRAK4|IKBKB|TRAF6|HIF1A|CCL5|CCL16|CCL4 | 77 | 325|484|485|529|258|500|165|322|345|199|308|299|184|358|434|289|206|314|274|371|526|315|495|135|501|118 | 6.43E-16 |  | GO.0071347 | 1.3627088 | 0 |
| 577 | 52 | GO Process |  | regulation of signaling receptor activity | 2.63E-14 | CX3CL1|TGFB1|CCL1|CCL13|CXCL6|TGFB3|TNFSF10|TNFSF9|LIF|PXDN|IL36G|IL36B|CCL21|IL1A|IL1B|IL7|IL11|IL9|TNFSF4|TGFA|CXCL3|CXCL5|TNFRSF11B|IL16|IL12A|IL13|SOCS5|CXCL11|CSF1|IL25|CXCL14|IL17F|CCL20|CCL28|CXCL9|TGFB2|IL20|IGSF1|TNFSF13B|CCL7|IL33|IL36RN|SOCS4|CXCL12|TNFSF11|IL6|OSGIN2|IL10|IL22|CCL5|CCL16|CCL4 | 77 | 325|350|484|485|531|265|202|120|432|543|561|558|500|322|345|266|126|295|383|279|356|355|359|385|519|418|226|321|149|478|468|367|308|488|283|376|161|132|326|289|346|536|523|181|430|218|446|425|534|135|501|118 | 7.27E-16 |  | GO.0010469 | 1.358004425 | 0 |
| 459 | 46 | Reactome Pathways | | Cellular responses to external stimuli | 3.08E-14 | MAPK1|NFKB1|CDKN1B|ATG14|NOX4|IL1A|STAT3|CDK6|SOD1|ATG10|ATG3|BAG4|HIF1AN|ATG4D|BAG5|LAMTOR4|MAP4K4|MAP2K3|ATG7|MAP3K5|MAPK10|GPX6|MAPKAPK2|ATG5|BAG3|BAG2|AGO3|AGO1|AGO4|HIF3A|ATG16L1|MAPK8|MAP2K7|TXNRD2|IL6|ATG9A|MAPK9|TNIK|MAP2K4|ATG12|AMBRA1|ATG13|HIF1A|SOD2|MAPKAPK5|MAP2K6 | 77 | 469|529|285|150|364|322|334|365|281|155|113|409|228|213|124|188|254|528|119|335|380|388|268|312|318|116|520|239|240|510|223|282|236|476|218|257|250|535|517|421|131|559|495|381|370|255 | 1.05E-15 |  | HSA-8953897 | 1.351144928 | 0 |
| 529 | 51 | InterPro Domains | | Protein kinase-like domain superfamily | 3.34E-14 | MAP4K5|MAPK1|MAP3K10|IRAK2|PDGFRA|PDGFRB|MAPK6|MAP3K8|MAP4K3|MAP3K13|CDK6|ETNK1|CSF1R|MAP2K1|MAP3K11|OXSR1|AATK|MAP4K4|MAP2K3|MAP3K15|TGFBR2|MAP3K5|MAPK10|MAP3K3|KIAA1804|MAPKAPK2|MAP3K7|IRAK1|TGFBR1|ZAK|MAP3K19|JAK2|MAP3K4|ULK2|MAPK8|MAP2K7|MAP3K1|MAPK4|MAP3K2|IRAK4|MAPK9|ITK|TNIK|MAP2K4|IKBKB|MAP3K12|MAPKAPK5|MAP3K9|IKBKE|MAP2K6|MAP3K14 | 77 | 451|469|489|258|516|506|235|165|141|490|365|191|153|292|439|504|560|254|528|384|332|335|380|299|190|268|358|434|368|264|352|309|466|214|282|236|397|231|374|371|250|556|535|517|526|426|370|290|548|255|171 | 1.13E-16 |  | IPR011009 | 1.347625353 | 0 |
| 1286 | 81 | GO Process |  | positive regulation of developmental process | 4.23E-14 | CX3CL1|TNFRSF1A|TGFBR3|TGFB1|NFKB1|TGFB3|TNFSF9|LIF|PIAS1|ARG2|PDGFRB|MAPK6|MMD|MSR1|SMAD2|RIMS2|SMURF2|IL1A|IL1B|IL7|STAT3|BNIP2|DOCK5|DOCK1|TNFSF4|TNFRSF13C|HIF1AN|LTBP3|MAP2K1|IL12A|IL13|SOCS5|IL7R|PPARD|CFLAR|IL1RAP|RHOJ|TNFRSF12A|CSF1|PAWR|SOCS1|SOCS3|SMAD3|BCL9L|BCL11A|SMAD4|RBPJ|TGFBR2|MAP3K5|SMURF1|CXCL9|MAP3K3|TGFB2|IL20|IL6R|CXCR3|TGIF2|TGFBR1|TNFSF13B|TNFRSF1B|IL6ST|JAK2|LOXL2|CXCL12|NFATC2|BLOC1S5|BCL2|TNFSF11|BCL6|IL6|CXCR4|MAPK9|IL10|SMAD1|TRAF6|DICER1|HIF1A|SMAD5|SOD2|SOCS2|TNFAIP3 | 77 | 325|351|447|350|529|265|120|432|554|518|506|235|427|147|200|331|362|322|345|266|334|187|406|215|383|224|228|441|292|519|418|226|176|227|133|199|375|340|149|396|460|246|241|125|507|470|438|332|335|464|283|299|376|161|348|230|259|368|326|276|391|309|480|181|115|198|475|430|310|218|450|250|425|193|315|121|495|201|381|419|164 | 1.18E-15 |  | GO.0051094 | 1.337365963 | 0 |
| 52 | 19 | GO Process |  | activation of MAPKK activity | 4.26E-14 | MAP3K10|MAP3K8|MAP3K13|MAP3K11|MAP3K15|MAP3K5|MAP3K3|KIAA1804|MAP3K7|TGFBR1|ZAK|JAK2|MAP3K4|MAP3K1|MAP3K2|TNIK|MAP3K12|MAP3K9|MAP3K14 | 77 | 489|165|490|439|384|335|299|190|358|368|264|309|466|397|374|535|426|290|171 | 1.20E-15 |  | GO.0000186 | 1.33705904 | 0 |
| 859 | 64 | GO Process |  | defense response to other organism | 4.79E-14 | CX3CL1|TNFRSF1A|TGFB1|CCL1|CCL13|CXCL6|NFKB1|IL36G|IL36B|CCL21|ARG2|IRF8|IL22RA1|TNFSF4|CSF1R|DEFB1|IL12A|DEFB4A|IL1RAP|CSF1|IFNLR1|IFNAR2|RBPJ|ATG7|IRF5|CCL20|MAP3K5|CXCL9|IRF6|IL6R|IRAK1|BCL10|IFI44L|IFIT3|IFIT2|TLR4|HLA-DQB1|CCL7|BNIP3L|IRF4|IL33|JAK2|LYST|ATG16L1|IL36RN|IRF9|BCL2|IL6|TLR6|IRAK4|ITK|HLA-B|ILF3|IL10|TICAM2|DEFB4B|IKBKB|TRAF3|IKBKE|CCL5|MR1|CCL16|CCL4|MAP3K14 | 77 | 325|351|350|484|485|531|529|561|558|500|518|398|211|383|153|112|519|158|199|149|341|452|438|119|160|308|335|283|487|348|434|323|177|404|394|342|109|289|499|169|346|309|555|223|536|492|475|218|305|371|556|114|110|425|329|166|526|557|548|135|540|501|118|171 | 1.35E-15 |  | GO.0098542 | 1.331966449 | 0 |
| 908 | 66 | GO Process |  | positive regulation of cell differentiation | 4.97E-14 | CX3CL1|TGFB1|NFKB1|TGFB3|TNFSF9|LIF|PIAS1|MAPK6|MMD|MSR1|SMAD2|RIMS2|IL1B|IL7|STAT3|BNIP2|DOCK5|DOCK1|TNFSF4|HIF1AN|LTBP3|MAP2K1|IL12A|IL13|SOCS5|IL7R|PPARD|CFLAR|IL1RAP|TNFRSF12A|CSF1|SOCS1|SOCS3|SMAD3|BCL9L|BCL11A|SMAD4|RBPJ|TGFBR2|MAP3K5|SMURF1|CXCL9|TGFB2|IL20|IL6R|TGIF2|TGFBR1|IL6ST|JAK2|LOXL2|CXCL12|NFATC2|BLOC1S5|BCL2|TNFSF11|BCL6|IL6|CXCR4|MAPK9|SMAD1|TRAF6|DICER1|HIF1A|SMAD5|SOD2|SOCS2 | 77 | 325|350|529|265|120|432|554|235|427|147|200|331|345|266|334|187|406|215|383|228|441|292|519|418|226|176|227|133|199|340|149|460|246|241|125|507|470|438|332|335|464|283|376|161|348|259|368|391|309|480|181|115|198|475|430|310|218|450|250|193|315|121|495|201|381|419 | 1.41E-15 |  | GO.0045597 | 1.330364361 | 0 |
| 63 | 20 | Reactome Pathways | | MAP kinase activation | 5.49E-14 | MAPK1|NFKB1|IRAK2|MAP3K8|ATF2|MAP2K1|MAP2K3|MAPK10|MAPKAPK2|TAB2|MAP3K7|IRAK1|TAB3|MAPK8|MAP2K7|MAPK9|MAP2K4|IKBKB|TRAF6|MAP2K6 | 77 | 469|529|258|165|189|292|528|380|268|184|358|434|206|282|236|250|517|526|315|255 | 1.95E-15 |  | HSA-450294 | 1.326042766 | 0 |
| 298 | 37 | GO Process |  | positive regulation of leukocyte activation | 6.47E-14 | TGFB1|IL1RL1|TNFSF9|CCR7|CCL21|MAP3K8|IL1B|IL7|TNFSF4|TNFRSF13C|IL12A|CTLA4|IL13|SOCS5|IL7R|ICOS|SOCS1|LAMP1|BTLA|PDCD1|TGFBR2|BCL10|TLR4|TNFSF13B|IL6ST|IL33|NFATC2|PDCD1LG2|BCL2|TNFSF11|BCL6|IL6|TLR6|HLA-DMB|IL10|TRAF6|CCL5 | 77 | 350|336|120|243|500|165|345|266|383|224|519|183|418|226|176|167|460|360|449|537|332|323|342|326|391|346|115|373|475|430|310|218|305|553|425|315|135 | 1.85E-15 |  | GO.0002696 | 1.318909572 | 0 |
| 470 | 46 | GO Process |  | regulation of leukocyte activation | 8.20E-14 | TGFB1|IL1RL1|TNFSF9|CCR7|CCL21|ARG2|SMAD7|MAP3K8|IL1B|IL7|SOD1|TNFSF4|TNFRSF13C|TNFRSF21|IL12A|CTLA4|IL13|SOCS5|IL7R|ICOS|PAWR|SOCS1|LAMP1|BTLA|PDCD1|IKZF3|TGFBR2|BCL10|TLR4|TNFSF13B|IRF4|IL6ST|IL33|NFATC2|PDCD1LG2|SOCS6|BCL2|TNFSF11|BCL6|IL6|TLR6|HLA-DMB|IL10|TRAF6|CCL5|TNFAIP3 | 77 | 350|336|120|243|500|518|195|165|345|266|281|383|224|513|519|183|418|226|176|167|396|460|360|449|537|392|332|323|342|326|169|391|346|115|373|244|475|430|310|218|305|553|425|315|135|164 | 2.36E-15 |  | GO.0002694 | 1.308618615 | 0 |
| 302 | 37 | GO Process |  | regulation of T cell activation | 9.34E-14 | TGFB1|TNFSF9|CCR7|CCL21|ARG2|SMAD7|MAP3K8|IL1B|IL7|SOD1|TNFSF4|TNFRSF13C|TNFRSF21|IL12A|CTLA4|SOCS5|IL7R|ICOS|PAWR|SOCS1|BTLA|PDCD1|TGFBR2|BCL10|TNFSF13B|IRF4|IL6ST|NFATC2|PDCD1LG2|SOCS6|TNFSF11|BCL6|IL6|HLA-DMB|IL10|TRAF6|CCL5 | 77 | 350|120|243|500|518|195|165|345|266|281|383|224|513|519|183|226|176|167|396|460|449|537|332|323|326|169|391|115|373|244|430|310|218|553|425|315|135 | 2.71E-15 |  | GO.0050863 | 1.302965312 | 0 |
| 375 | 41 | GO Process |  | positive regulation of cell adhesion | 9.67E-14 | CX3CL1|TGFB1|TNFSF9|CCR7|CCL21|SMAD7|MAP3K8|IL1B|IL7|CDK6|DOCK5|DOCK1|TNFSF4|BAG4|TNFRSF13C|IL12A|CTLA4|SOCS5|IL7R|ICOS|CSF1|SOCS1|SMAD3|BTLA|PDCD1|TGFBR2|CCL28|TGFB2|BCL10|TNFSF13B|IL6ST|JAK2|CXCL12|PDCD1LG2|TNFSF11|BCL6|IL6|HLA-DMB|IL10|TRAF6|CCL5 | 77 | 325|350|120|243|500|195|165|345|266|365|406|215|383|409|224|519|183|226|176|167|149|460|241|449|537|332|488|376|323|326|391|309|181|373|430|310|218|553|425|315|135 | 2.82E-15 |  | GO.0045785 | 1.301457353 | 0 |
| 147 | 27 | GO Process |  | response to interleukin-1 | 1.06E-13 | CX3CL1|CCL1|CCL13|NFKB1|IRAK2|CCL21|MAP3K8|IL1A|IL1B|IL1RAP|CCL20|MAP3K3|TAB2|MAP3K7|IRAK1|CCL7|TAB3|TANK|IL1R1|IRAK4|IKBKB|TRAF6|HIF1A|TNFRSF11A|CCL5|CCL16|CCL4 | 77 | 325|484|485|529|258|500|165|322|345|199|308|299|184|358|434|289|206|314|274|371|526|315|495|163|135|501|118 | 3.11E-15 |  | GO.0070555 | 1.297469413 | 0 |
| 878 | 64 | GO Process |  | positive regulation of cell population proliferation | 1.20E-13 | TGFBR3|MAPK1|TGFB1|CDKN1B|TGFB3|TNFSF9|LIF|PDGFRA|HILPDA|TBRG4|PDGFRB|IL1B|IL7|ATF2|IL11|STAT3|CDK6|CCAR1|IL9|TNFSF4|CSF1R|TNFRSF13C|TGFA|CXCL5|RICTOR|AVPR1A|LTBP3|BCL2L1|IL12A|IL13|IL7R|PPARD|CFLAR|CSF1|SMAD4|RBPJ|TGFBR2|MAP3K5|MAP3K3|TGFB2|IL6R|CACUL1|IRAK1|CXCR3|TGFBR1|TNFSF13B|IL6ST|JAK2|PDCD10|PRC1|CXCL12|NFATC2|PDCD1LG2|BCL2|BCL6|IL6|IRAK4|HLA-DMB|IL10|TRAF6|HIF1A|TNFRSF11A|CCL5|TNFAIP3 | 77 | 447|469|350|285|265|120|432|516|462|496|506|345|266|189|126|334|365|111|295|383|153|224|279|355|302|222|441|372|519|418|176|227|133|149|470|438|332|335|299|376|348|522|434|230|368|326|391|309|316|511|181|115|373|475|310|218|371|553|425|315|495|163|135|164 | 3.56E-15 |  | GO.0008284 | 1.292081875 | 0 |
| 515 | 47 | KEGG Pathways | | Pathways in cancer | 1.34E-13 | MAPK1|TGFB1|NFKB1|CDKN1B|TGFB3|TRAF2|PDGFRA|TRAF5|PDGFRB|SMAD2|TRAF4|IL7|STAT3|CDK6|CSF1R|TGFA|MAP2K1|BCL2L1|IL12A|IL13|IL7R|PPARD|SMAD3|SMAD4|IFNAR2|TGFBR2|MAPK10|TGFB2|IL6R|IL2RG|TGFBR1|IL6ST|JAK2|BCL2L11|MAPK8|CXCL12|BCL2|TXNRD2|CSF2RB|IL6|CXCR4|MAPK9|IKBKB|TRAF6|HIF1A|APAF1|TRAF3 | 77 | 469|350|529|285|265|444|516|142|506|200|194|266|334|365|153|279|292|372|519|418|176|227|241|470|452|332|380|376|348|563|368|391|309|234|282|181|475|476|524|218|450|250|526|315|495|148|557 | 1.30E-14 |  | hsa05200 | 1.28728952 | 0 |
| 172 | 28 | KEGG Pathways | | Tuberculosis | 1.38E-13 | TNFRSF1A|MAPK1|TGFB1|NFKB1|TGFB3|IRAK2|IL1A|IL1B|IL12A|EEA1|LAMP1|MAPK10|TGFB2|IRAK1|BCL10|TLR4|JAK2|MAPK8|BCL2|IL6|TLR6|IRAK4|MAPK9|HLA-DMB|LAMP2|IL10|TRAF6|APAF1 | 77 | 351|469|350|529|265|258|322|345|519|146|360|380|376|434|323|342|309|282|475|218|305|371|250|553|304|425|315|148 | 1.40E-14 |  | hsa05152 | 1.286012091 | 0 |
| 401 | 42 | GO Process |  | regulation of lymphocyte activation | 1.67E-13 | TGFB1|TNFSF9|CCR7|CCL21|ARG2|SMAD7|MAP3K8|IL1B|IL7|SOD1|TNFSF4|TNFRSF13C|TNFRSF21|IL12A|CTLA4|IL13|SOCS5|IL7R|ICOS|PAWR|SOCS1|LAMP1|BTLA|PDCD1|IKZF3|TGFBR2|BCL10|TNFSF13B|IRF4|IL6ST|NFATC2|PDCD1LG2|SOCS6|BCL2|TNFSF11|BCL6|IL6|HLA-DMB|IL10|TRAF6|CCL5|TNFAIP3 | 77 | 350|120|243|500|518|195|165|345|266|281|383|224|513|519|183|418|226|176|167|396|460|360|449|537|392|332|323|326|169|391|115|373|244|475|430|310|218|553|425|315|135|164 | 4.97E-15 |  | GO.0051249 | 1.277728353 | 0 |
| 886 | 64 | GO Process |  | regulation of cellular component movement | 1.77E-13 | CX3CL1|TGFBR3|MAPK1|TGFB1|CCL1|CXCL6|CDKN1B|CCR7|DOCK7|PDGFRA|DOCK10|CCL21|PDGFRB|SMAD7|SMURF2|NOX4|IL1A|IL1B|STAT3|CDK6|CCAR1|ACKR3|DOCK5|DOCK1|CSF1R|BAG4|CXCL3|CXCL5|DEFB1|IL12A|CXCL11|PPARD|RHOJ|CSF1|SMAD3|LAMP1|CXCL14|GPR29|MAP2K3|CCL20|TGFBR2|CCL28|CXCL9|MAP3K3|NOS1AP|TGFB2|IL6R|CXCR3|TGFBR1|CCL7|IL33|JAK2|PDCD10|CXCL12|BCL2|CMKBR6|BCL6|IL6|IL1R1|DOCK4|HIF1A|SOD2|CCL5|CCL4 | 77 | 325|447|469|350|484|531|285|243|413|516|505|500|506|195|362|364|322|345|334|365|111|247|406|215|153|409|356|355|112|519|321|227|375|149|241|360|468|448|528|308|332|488|283|299|212|376|348|230|368|289|346|309|316|181|475|431|310|218|274|544|495|381|135|118 | 5.30E-15 |  | GO.0051270 | 1.275202673 | 0 |
| 365 | 40 | GO Process |  | positive regulation of defense response | 1.94E-13 | CX3CL1|TNFRSF1A|NFKB1|CRTAM|IL1RL1|CCR7|IRAK2|IL1B|PDCD4|TNFSF4|IL17RB|IL12A|TNIP1|LAMP1|IL17F|MAPKAPK2|TAB2|MAP3K7|IRAK1|BCL10|TLR4|TAB3|IL6ST|IL33|JAK2|TANK|TNFSF11|MAP3K1|IL6|TLR6|IRAK4|TICAM2|IKBKB|TRAF6|TRAF3|IKBKE|TNFRSF11A|MAP2K6|CCL5|TNFAIP3 | 77 | 325|351|529|151|336|243|258|345|311|383|497|519|538|360|367|268|184|358|434|323|342|206|391|346|309|314|430|397|218|305|371|329|526|315|557|548|163|255|135|164 | 5.84E-15 |  | GO.0031349 | 1.271219827 | 0 |
| 835 | 62 | GO Function | | kinase activity | 1.97E-13 | MAP4K5|TGFBR3|MAPK1|CDKN1B|MAP3K10|IRAK2|PDGFRA|TBRG4|PDGFRB|MAPK6|MMD|MAP3K8|ITPKC|MAP4K3|MAP3K13|CDK6|ETNK1|CSF1R|TRAT1|MAP2K1|MAP3K11|OXSR1|ICOS|AATK|MAP4K4|MAP2K3|MAP3K15|TGFBR2|MAP3K5|MAPK10|MAP3K3|KIAA1804|MAPKAPK2|MAP3K7|IRAK1|PI4K2A|TGFBR1|ZAK|MAP3K19|JAK2|MAP3K4|ULK2|MAPK8|MAP2K7|MAP3K1|MAPK4|CSF2RB|LTBP1|MAP3K2|IRAK4|MAPK9|ITK|TNIK|MAP2K4|IKBKB|MAP3K12|MAPKAPK5|MAP3K9|IKBKE|MAP2K6|CCL5|MAP3K14 | 77 | 451|447|469|285|489|258|516|496|506|235|427|165|420|141|490|365|191|153|527|292|439|504|167|560|254|528|384|332|335|380|299|190|268|358|434|399|368|264|352|309|466|214|282|236|397|231|524|154|374|371|250|556|535|517|526|426|370|290|548|255|135|171 | 4.61E-15 |  | GO.0016301 | 1.270553377 | 0 |
| 70 | 20 | GO Process |  | I-kappaB kinase/NF-kappaB signaling | 3.46E-13 | TNFRSF1A|NFKB1|TRAF2|IRAK2|SNIP1|NKIRAS2|TAB2|MAP3K7|IRAK1BP1|BCL10|TLR4|TAB3|TANK|NKIRAS1|TICAM2|IKBKB|TRAF6|IKBKE|ERC1|MAP3K14 | 77 | 351|529|444|258|337|273|184|358|400|323|342|206|314|417|329|526|315|548|401|171 | 1.05E-14 |  | GO.0007249 | 1.24609239 | 0 |
| 93 | 22 | GO Process |  | extrinsic apoptotic signaling pathway | 4.43E-13 | TNFRSF1A|TGFB1|TRAF2|BCL2L2|BCL2L10|IL1A|IL1B|TNFRSF10B|BCL2L1|IL12A|TNFRSF12A|SMAD3|TGFB2|IL6R|MCL1|BAG3|TNFRSF1B|IL33|JAK2|BCL2L11|BCL2|MOAP1 | 77 | 351|350|444|551|550|322|345|410|372|519|340|241|376|348|278|318|276|346|309|234|475|138 | 1.35E-14 |  | GO.0097191 | 1.235359627 | 0 |
| 106 | 23 | Reactome Pathways | | Interleukin-4 and Interleukin-13 signaling | 4.49E-13 | TGFB1|LIF|IL1A|IL1B|STAT3|BCL2L1|IL12A|IL13|SOCS5|SOCS1|SOCS3|IL17F|IL6R|MCL1|IL2RG|TNFRSF1B|IRF4|JAK2|BCL2|BCL6|IL6|IL10|HIF1A | 77 | 350|432|322|345|334|372|519|418|226|460|246|367|348|278|563|276|169|309|475|310|218|425|495 | 1.67E-14 |  | HSA-6785807 | 1.234775366 | 0 |
| 599 | 51 | GO Function | | protein kinase binding | 4.63E-13 | MAPK1|CDKN1B|EXOC2|TRAF2|PDGFRB|MAPK6|TRAF4|NOX4|ATF2|STAT3|MAP3K13|TRAT1|RICTOR|AVPR1A|BCL2L1|SOCS5|MAP3K11|IL1RAP|TNIP1|SOCS1|SMAD3|BAG5|IFNAR2|MAP2K3|TGFBR2|MAP3K5|MAPKAPK2|CACUL1|MAP3K7|MTCP1|SIKE1|BCL10|JAK2|PDCD10|BCL2L11|PRC1|MAP2K7|MAP3K1|MAPK4|MAP3K2|DOCK4|MAP2K4|SMAD1|IKBKB|ATG13|TRAF6|HIF1A|MAP3K12|MAPKAPK5|TRAF3|MAP2K6 | 77 | 469|285|339|444|506|235|194|364|189|334|490|527|302|222|372|226|439|199|538|460|241|124|452|528|332|335|268|522|358|162|338|323|309|316|234|511|236|397|231|374|544|517|193|526|559|315|495|426|370|557|255 | 1.15E-14 |  | GO.0019901 | 1.233441901 | 0 |
| 176 | 28 | GO Process |  | cellular response to biotic stimulus | 7.61E-13 | MAPK1|TGFB1|CXCL6|NFKB1|IRAK2|IL1B|IRF8|PDCD4|TNFSF4|ATG10|IL12A|PPARD|TNIP1|CCL20|IRAK1|TLR4|TNFRSF1B|MAPK8|IL6|TLR6|IL10|TICAM2|TRAF6|APAF1|LITAF|TXNIP|CCL5|TNFAIP3 | 77 | 469|350|531|529|258|345|398|311|383|155|519|227|538|308|434|342|276|282|218|305|425|329|315|148|395|415|135|164 | 2.34E-14 |  | GO.0071216 | 1.211861534 | 0 |
| 148 | 26 | GO Process |  | positive regulation of stress-activated MAPK cascade | 8.34E-13 | MAP4K5|CCR7|TRAF2|MAP3K10|CCL21|TRAF4|IL1B|MAP3K11|MAP3K5|MAP3K3|KIAA1804|TGFB2|MAP3K7|ZAK|TNFRSF19|MAP3K4|PDCD10|MAP2K7|TNFSF11|MAP3K2|TLR6|TNIK|MAP2K4|TRAF6|MAP3K9|TNFRSF11A | 77 | 451|243|444|489|500|194|345|439|335|299|190|376|358|264|256|466|316|236|430|374|305|535|517|315|290|163 | 2.58E-14 |  | GO.0032874 | 1.207883395 | 0 |
| 1098 | 71 | GO Process |  | negative regulation of multicellular organismal process | 8.73E-13 | TNFRSF1A|TGFB1|NFKB1|CDKN1B|IL1RL1|TGFB3|PDGFRA|ARG2|SMAD7|IL1B|ATF2|STAT3|CDK6|SOD1|DOCK5|PDCD4|TNFSF4|SMAD6|TNFRSF21|TNFRSF11B|AVPR1A|NFATC3|IL12A|CTLA4|IL13|SOCS5|SOCS1|SMAD3|PDCD1|IL17F|BCL11A|BAG5|SMAD4|MAP4K4|RBPJ|TGFBR2|SMURF1|TGFB2|IL6R|AGO1|TRAF3IP1|CXCR3|TLR4|TGFBR1|TNFRSF1B|BCOR|NFIB|IL33|JAK2|LOXL2|PDCD10|IL36RN|ULK2|NFATC2|PDCD1LG2|BCL2|BCL6|IL6|NFATC4|TLR6|MAPKBP1|DOCK4|IL10|TICAM2|DICER1|HIF1A|IKBKE|TNFRSF9|ECSCR|SOCS2|TNFAIP3 | 77 | 351|350|529|285|336|265|516|518|195|345|189|334|365|281|406|311|383|369|513|359|222|210|519|183|418|226|460|241|537|367|507|124|470|254|438|332|464|376|348|239|474|230|342|368|276|541|170|346|309|480|316|536|214|115|373|475|310|218|249|305|298|544|425|329|121|495|548|473|253|419|164 | 2.72E-14 |  | GO.0051241 | 1.205898576 | 0 |
| 208 | 30 | GO Process |  | regulation of stress-activated MAPK cascade | 8.85E-13 | MAP4K5|MAPK1|CCR7|TRAF2|MAP3K10|CCL21|TRAF4|IL1B|PDCD4|MAP2K1|MAP3K11|MAP4K4|MAP3K5|MAP3K3|KIAA1804|TGFB2|MAP3K7|ZAK|TNFRSF19|MAP3K4|PDCD10|MAP2K7|TNFSF11|MAP3K2|TLR6|TNIK|MAP2K4|TRAF6|MAP3K9|TNFRSF11A | 77 | 451|469|243|444|489|500|194|345|311|292|439|254|335|299|190|376|358|264|256|466|316|236|430|374|305|535|517|315|290|163 | 2.77E-14 |  | GO.0032872 | 1.205305673 | 0 |
| 1258 | 77 | GO Process |  | negative regulation of signaling | 9.03E-13 | CX3CL1|TGFBR3|TGFB1|TNFAIP1|IL1RL1|TGFB3|TNFSF10|TRAF2|LIF|BCL2L2|PXDN|BCL2L10|SMAD7|SMAD2|SMURF2|IL1A|IL1B|IL7|IL11|ACKR3|TNFRSF10B|PDCD4|DEPTOR|SMAD6|TRAT1|CREBRF|HIF1AN|AVPR1A|BCL2L1|SOCS5|CFLAR|TNIP1|C8orf4|PAWR|SOCS1|SOCS3|SMAD3|BCL9L|BAG5|SMAD4|MAP4K4|TGFBR2|SMURF1|TGFB2|MCL1|IGSF1|YWHAB|TRAF3IP1|TLR4|TGFBR1|IRF4|IL6ST|TANK|IL36RN|SOCS4|CXCL12|SOCS6|BCL2|GATSL3|BCL6|IL6|LTBP1|NFATC4|TLR6|MAPKBP1|TICAM2|HIF1A|TNFAIP8L1|SOD2|MAPKAPK5|LITAF|PIAS2|CCL5|SOCS2|TNFAIP3|SOCS7|GATSL2 | 77 | 325|447|350|280|336|265|202|444|432|551|543|550|195|200|362|322|345|266|126|247|410|311|408|369|527|296|228|222|372|226|133|538|293|396|460|246|241|125|124|470|254|332|464|376|278|132|440|474|342|368|169|391|314|536|523|181|244|475|288|310|218|154|249|305|298|329|495|453|381|370|395|284|135|419|164|186|402 | 2.84E-14 |  | GO.0023057 | 1.204431225 | 0 |
| 366 | 39 | GO Process |  | regulation of cell-cell adhesion | 9.36E-13 | CX3CL1|TGFB1|TNFSF9|CCR7|CCL21|ARG2|SMAD7|MAP3K8|IL1B|IL7|TNFSF4|TNFRSF13C|TNFRSF21|IL12A|CTLA4|SOCS5|IL7R|ICOS|PAWR|SOCS1|BTLA|PDCD1|TGFBR2|CCL28|BCL10|TNFSF13B|IL6ST|JAK2|CXCL12|PDCD1LG2|SOCS6|TNFSF11|BCL6|PPARA|IL6|HLA-DMB|IL10|TRAF6|CCL5 | 77 | 325|350|120|243|500|518|195|165|345|266|383|224|513|519|183|226|176|167|396|460|449|537|332|488|323|326|391|309|181|373|244|430|310|491|218|553|425|315|135 | 2.97E-14 |  | GO.0022407 | 1.202872415 | 0 |
| 130 | 24 | KEGG Pathways | | FoxO signaling pathway | 9.37E-13 | MAPK1|TGFB1|CDKN1B|TGFB3|TNFSF10|SMAD2|STAT3|MAP2K1|IL7R|SMAD3|SMAD4|TGFBR2|MAPK10|TGFB2|TGFBR1|BCL2L11|MAPK8|BCL6|IL6|MAPK9|IL10|ATG12|IKBKB|SOD2 | 77 | 469|350|285|265|202|200|334|292|176|241|470|332|380|376|368|234|282|310|218|250|425|421|526|381 | 9.99E-14 |  | hsa04068 | 1.202826041 | 0 |
| 753 | 57 | GO Process |  | regulation of cell migration | 9.63E-13 | CX3CL1|MAPK1|TGFB1|CCL1|CXCL6|CCR7|DOCK7|PDGFRA|DOCK10|CCL21|PDGFRB|SMAD7|SMURF2|NOX4|IL1A|IL1B|STAT3|CCAR1|ACKR3|DOCK5|DOCK1|CSF1R|BAG4|CXCL3|CXCL5|IL12A|CXCL11|PPARD|RHOJ|CSF1|SMAD3|CXCL14|GPR29|MAP2K3|CCL20|TGFBR2|CCL28|CXCL9|MAP3K3|TGFB2|IL6R|CXCR3|TGFBR1|CCL7|IL33|JAK2|PDCD10|CXCL12|BCL2|CMKBR6|IL6|IL1R1|DOCK4|HIF1A|SOD2|CCL5|CCL4 | 77 | 325|469|350|484|531|243|413|516|505|500|506|195|362|364|322|345|334|111|247|406|215|153|409|356|355|519|321|227|375|149|241|468|448|528|308|332|488|283|299|376|348|230|368|289|346|309|316|181|475|431|218|274|544|495|381|135|118 | 3.09E-14 |  | GO.0030334 | 1.201637371 | 0 |
| 1160 | 73 | GO Process |  | negative regulation of signal transduction | 1.25E-12 | CX3CL1|TGFBR3|TGFB1|TNFAIP1|IL1RL1|TGFB3|TNFSF10|TRAF2|LIF|BCL2L2|PXDN|BCL2L10|SMAD7|SMAD2|SMURF2|IL1A|IL1B|IL7|ACKR3|TNFRSF10B|PDCD4|DEPTOR|SMAD6|CREBRF|HIF1AN|BCL2L1|SOCS5|CFLAR|TNIP1|C8orf4|PAWR|SOCS1|SOCS3|SMAD3|BCL9L|BAG5|SMAD4|TGFBR2|SMURF1|TGFB2|MCL1|IGSF1|YWHAB|TRAF3IP1|TLR4|TGFBR1|IRF4|IL6ST|TANK|IL36RN|SOCS4|CXCL12|SOCS6|BCL2|GATSL3|BCL6|IL6|LTBP1|NFATC4|TLR6|MAPKBP1|TICAM2|HIF1A|TNFAIP8L1|SOD2|MAPKAPK5|LITAF|PIAS2|CCL5|SOCS2|TNFAIP3|SOCS7|GATSL2 | 77 | 325|447|350|280|336|265|202|444|432|551|543|550|195|200|362|322|345|266|247|410|311|408|369|296|228|372|226|133|538|293|396|460|246|241|125|124|470|332|464|376|278|132|440|474|342|368|169|391|314|536|523|181|244|475|288|310|218|154|249|305|298|329|495|453|381|370|395|284|135|419|164|186|402 | 4.04E-14 |  | GO.0009968 | 1.190308999 | 0 |
| 807 | 59 | GO Process |  | regulation of cell motility | 1.31E-12 | CX3CL1|MAPK1|TGFB1|CCL1|CXCL6|CCR7|DOCK7|PDGFRA|DOCK10|CCL21|PDGFRB|SMAD7|SMURF2|NOX4|IL1A|IL1B|STAT3|CDK6|CCAR1|ACKR3|DOCK5|DOCK1|CSF1R|BAG4|CXCL3|CXCL5|DEFB1|IL12A|CXCL11|PPARD|RHOJ|CSF1|SMAD3|CXCL14|GPR29|MAP2K3|CCL20|TGFBR2|CCL28|CXCL9|MAP3K3|TGFB2|IL6R|CXCR3|TGFBR1|CCL7|IL33|JAK2|PDCD10|CXCL12|BCL2|CMKBR6|IL6|IL1R1|DOCK4|HIF1A|SOD2|CCL5|CCL4 | 77 | 325|469|350|484|531|243|413|516|505|500|506|195|362|364|322|345|334|365|111|247|406|215|153|409|356|355|112|519|321|227|375|149|241|468|448|528|308|332|488|283|299|376|348|230|368|289|346|309|316|181|475|431|218|274|544|495|381|135|118 | 4.28E-14 |  | GO.2000145 | 1.18827287 | 0 |
| 138 | 25 | GO Process |  | positive regulation of chemotaxis | 1.35E-12 | TGFB1|CCL1|CXCL6|CCR7|CCL21|PDGFRB|CXCL3|CXCL5|IL16|IL12A|CXCL11|CSF1|CCR4|SMAD3|CXCL14|GPR29|CXCL9|IL6R|CXCR3|CCL7|CXCL12|CMKBR6|IL6|CCL5|CCL4 | 77 | 350|484|531|243|500|506|356|355|385|519|321|149|502|241|468|448|283|348|230|289|181|431|218|135|118 | 4.42E-14 |  | GO.0050921 | 1.186966623 | 0 |
| 881 | 62 | GO Process |  | regulation of locomotion | 1.36E-12 | CX3CL1|MAPK1|TGFB1|CCL1|CXCL6|CCR7|DOCK7|PDGFRA|DOCK10|CCL21|PDGFRB|SMAD7|SMURF2|NOX4|IL1A|IL1B|STAT3|CDK6|CCAR1|ACKR3|DOCK5|DOCK1|CSF1R|BAG4|CXCL3|CXCL5|DEFB1|IL16|IL12A|CXCL11|PPARD|RHOJ|CSF1|CCR4|SMAD3|CXCL14|GPR29|MAP2K3|CCL20|TGFBR2|CCL28|CXCL9|MAP3K3|TGFB2|IL6R|CXCR3|TGFBR1|CCL7|IL33|JAK2|PDCD10|CXCL12|BCL2|CMKBR6|IL6|IL1R1|CXCR4|DOCK4|HIF1A|SOD2|CCL5|CCL4 | 77 | 325|469|350|484|531|243|413|516|505|500|506|195|362|364|322|345|334|365|111|247|406|215|153|409|356|355|112|385|519|321|227|375|149|502|241|468|448|528|308|332|488|283|299|376|348|230|368|289|346|309|316|181|475|431|218|274|450|544|495|381|135|118 | 4.48E-14 |  | GO.0040012 | 1.186646109 | 0 |
| 631 | 52 | UniProt Keywords | | Kinase | 1.44E-12 | MAP4K5|MAPK1|MAP3K10|PDGFRA|PDGFRB|MAPK6|MAP3K8|ITPKC|MAP4K3|MAP3K13|CDK6|ETNK1|CSF1R|MAP2K1|MAP3K11|OXSR1|AATK|MAP4K4|MAP2K3|MAP3K15|TGFBR2|MAP3K5|MAPK10|MAP3K3|KIAA1804|MAPKAPK2|MAP3K7|IRAK1|PI4K2A|TGFBR1|ZAK|MAP3K19|JAK2|MAP3K4|ULK2|MAPK8|MAP2K7|MAP3K1|MAPK4|MAP3K2|IRAK4|MAPK9|ITK|TNIK|MAP2K4|IKBKB|MAP3K12|MAPKAPK5|MAP3K9|IKBKE|MAP2K6|MAP3K14 | 77 | 451|469|489|516|506|235|165|420|141|490|365|191|153|292|439|504|560|254|528|384|332|335|380|299|190|268|358|434|399|368|264|352|309|466|214|282|236|397|231|374|371|250|556|535|517|526|426|370|290|548|255|171 | 2.08E-14 |  | KW-0418 | 1.184163751 | 0 |
| 167 | 27 | GO Process |  | regulation of I-kappaB kinase/NF-kappaB signaling | 1.46E-12 | TNFRSF1A|IL1RL1|TNFSF10|CCR7|TRAF2|CCL21|TRAF5|IL1B|TNFRSF10B|CFLAR|TNIP1|MAP3K3|TAB2|MAP3K7|IRAK1|BCL10|TNFRSF19|TANK|TNFSF11|TLR6|IRAK4|MAPKBP1|IKBKB|TRAF6|IKBKE|TNFAIP3|MAP3K14 | 77 | 351|336|202|243|444|500|142|345|410|133|538|299|184|358|434|323|256|314|430|305|371|298|526|315|548|164|171 | 4.86E-14 |  | GO.0043122 | 1.183564714 | 0 |
| 601 | 50 | GO Process |  | immune system development | 1.47E-12 | TGFBR3|MAPK1|TGFB1|CCR7|PDGFRA|DOCK10|IL7|IL11|STAT3|IRF8|SOD1|DOCK11|CSF1R|CXCR5|MAP2K1|MAEA|IL7R|ICOS|CSF1|IL25|IKZF1|CCR4|SMAD3|GPR29|RBPJ|BCL11B|CCR9|TGFBR2|TGFB2|THEMIS|ATG5|TGFBR1|IRF4|JAK2|BCL2L11|CIAPIN1|BCL2|TNFSF11|CMKBR6|BCL6|IL6|IL18R1|ITK|IL10|TRAF6|HIF1A|SMAD5|TET2|TNFRSF11A|TNFAIP3 | 77 | 447|469|350|243|516|505|266|126|334|398|281|422|153|306|292|207|176|167|149|478|220|502|241|448|438|128|456|332|376|349|312|368|169|309|234|472|475|430|431|310|218|287|556|425|315|495|201|294|163|164 | 4.90E-14 |  | GO.0002520 | 1.183268267 | 0 |
| 1255 | 76 | GO Process |  | negative regulation of cell communication | 2.16E-12 | CX3CL1|TGFBR3|TGFB1|TNFAIP1|IL1RL1|TGFB3|TNFSF10|TRAF2|LIF|BCL2L2|PXDN|BCL2L10|SMAD7|SMAD2|SMURF2|IL1A|IL1B|IL7|IL11|ACKR3|TNFRSF10B|PDCD4|DEPTOR|SMAD6|CREBRF|HIF1AN|AVPR1A|BCL2L1|SOCS5|CFLAR|TNIP1|C8orf4|PAWR|SOCS1|SOCS3|SMAD3|BCL9L|BAG5|SMAD4|MAP4K4|TGFBR2|SMURF1|TGFB2|MCL1|IGSF1|YWHAB|TRAF3IP1|TLR4|TGFBR1|IRF4|IL6ST|TANK|IL36RN|SOCS4|CXCL12|SOCS6|BCL2|GATSL3|BCL6|IL6|LTBP1|NFATC4|TLR6|MAPKBP1|TICAM2|HIF1A|TNFAIP8L1|SOD2|MAPKAPK5|LITAF|PIAS2|CCL5|SOCS2|TNFAIP3|SOCS7|GATSL2 | 77 | 325|447|350|280|336|265|202|444|432|551|543|550|195|200|362|322|345|266|126|247|410|311|408|369|296|228|222|372|226|133|538|293|396|460|246|241|125|124|470|254|332|464|376|278|132|440|474|342|368|169|391|314|536|523|181|244|475|288|310|218|154|249|305|298|329|495|453|381|370|395|284|135|419|164|186|402 | 7.26E-14 |  | GO.0010648 | 1.166554625 | 0 |
| 48 | 17 | GO Function | | chemokine activity | 2.22E-12 | CX3CL1|CCL1|CCL13|CXCL6|CCL21|CXCL3|CXCL5|CXCL11|CXCL14|CCL20|CCL28|CXCL9|CCL7|CXCL12|CCL5|CCL16|CCL4 | 77 | 325|484|485|531|500|356|355|321|468|308|488|283|289|181|135|501|118 | 5.87E-14 |  | GO.0008009 | 1.165364703 | 0 |
| 74 | 19 | KEGG Pathways | | Pancreatic cancer | 2.24E-12 | MAPK1|TGFB1|NFKB1|TGFB3|SMAD2|STAT3|CDK6|TGFA|MAP2K1|BCL2L1|SMAD3|SMAD4|TGFBR2|MAPK10|TGFB2|TGFBR1|MAPK8|MAPK9|IKBKB | 77 | 469|350|529|265|200|334|365|279|292|372|241|470|332|380|376|368|282|250|526 | 2.50E-13 |  | hsa05212 | 1.164975198 | 0 |
| 98 | 21 | KEGG Pathways | | AGE-RAGE signaling pathway in diabetic complications | 2.47E-12 | MAPK1|TGFB1|NFKB1|CDKN1B|TGFB3|SMAD2|NOX4|IL1A|IL1B|STAT3|SMAD3|SMAD4|TGFBR2|MAPK10|TGFB2|TGFBR1|JAK2|MAPK8|BCL2|IL6|MAPK9 | 77 | 469|350|529|285|265|200|364|322|345|334|241|470|332|380|376|368|309|282|475|218|250 | 2.89E-13 |  | hsa04933 | 1.160730305 | 0 |
| 43 | 17 | InterPro Domains | | Chemokine interleukin-8-like superfamily | 2.66E-12 | CX3CL1|CCL1|CCL13|CXCL6|CCL21|CXCL3|CXCL5|CXCL11|CXCL14|CCL20|CCL28|CXCL9|CCL7|CXCL12|CCL5|CCL16|CCL4 | 77 | 325|484|485|531|500|356|355|321|468|308|488|283|289|181|135|501|118 | 1.35E-14 |  | IPR036048 | 1.157511836 | 0 |
| 43 | 17 | InterPro Domains | | Chemokine interleukin-8-like domain | 2.66E-12 | CX3CL1|CCL1|CCL13|CXCL6|CCL21|CXCL3|CXCL5|CXCL11|CXCL14|CCL20|CCL28|CXCL9|CCL7|CXCL12|CCL5|CCL16|CCL4 | 77 | 325|484|485|531|500|356|355|321|468|308|488|283|289|181|135|501|118 | 1.35E-14 |  | IPR001811 | 1.157511836 | 0 |
| 678 | 53 | GO Function | | kinase binding | 2.94E-12 | MAPK1|CDKN1B|EXOC2|TRAF2|PDGFRB|MAPK6|TRAF4|NOX4|ATF2|STAT3|MAP3K13|TRAT1|RICTOR|AVPR1A|BCL2L1|SOCS5|MAP3K11|IL1RAP|TNIP1|SOCS1|SMAD3|BAG5|IFNAR2|MAP2K3|TGFBR2|MAP3K5|MAPKAPK2|CACUL1|MAP3K7|MTCP1|SIKE1|BCL10|JAK2|PDCD10|BCL2L11|PRC1|MAP2K7|MAP3K1|MAPK4|MAP3K2|DOCK4|MAP2K4|SMAD1|IKBKB|ATG13|TRAF6|HIF1A|MAP3K12|MAPKAPK5|TRAF3|MAP2K6|JAKMIP2|TNFAIP3 | 77 | 469|285|339|444|506|235|194|364|189|334|490|527|302|222|372|226|439|199|538|460|241|124|452|528|332|335|268|522|358|162|338|323|309|316|234|511|236|397|231|374|544|517|193|526|559|315|495|426|370|557|255|251|164 | 8.22E-14 |  | GO.0019900 | 1.153165267 | 0 |
| 310 | 36 | InterPro Domains | | Serine/threonine-protein kinase, active site | 3.41E-12 | MAPK1|MAP3K10|MAPK6|MAP3K8|MAP3K13|CDK6|MAP2K1|MAP3K11|MAP4K4|MAP2K3|MAP3K15|TGFBR2|MAP3K5|MAPK10|KIAA1804|MAPKAPK2|MAP3K7|IRAK1|TGFBR1|ZAK|MAP3K19|MAP3K4|ULK2|MAPK8|MAP2K7|MAP3K1|MAPK4|MAPK9|TNIK|MAP2K4|IKBKB|MAP3K12|MAPKAPK5|MAP3K9|MAP2K6|MAP3K14 | 77 | 469|489|235|165|490|365|292|439|254|528|384|332|335|380|190|268|358|434|368|264|352|466|214|282|236|397|231|250|535|517|526|426|370|290|255|171 | 2.88E-14 |  | IPR008271 | 1.146724562 | 0 |
| 238 | 31 | GO Process |  | positive regulation of cell-cell adhesion | 3.57E-12 | CX3CL1|TGFB1|TNFSF9|CCR7|CCL21|SMAD7|MAP3K8|IL1B|IL7|TNFSF4|TNFRSF13C|IL12A|CTLA4|SOCS5|IL7R|ICOS|SOCS1|BTLA|PDCD1|TGFBR2|BCL10|TNFSF13B|IL6ST|PDCD1LG2|TNFSF11|BCL6|IL6|HLA-DMB|IL10|TRAF6|CCL5 | 77 | 325|350|120|243|500|195|165|345|266|383|224|519|183|226|176|167|460|449|537|332|323|326|391|373|430|310|218|553|425|315|135 | 1.21E-13 |  | GO.0022409 | 1.144733178 | 0 |
| 156 | 26 | UniProt Keywords | | Inflammatory response | 4.28E-12 | CCL13|IL36G|IL36B|CCL21|IL1A|IL1B|CSF1R|CXCL3|CXCL11|TNIP1|CSF1|CCL20|CXCL9|TLR4|CCL7|IL17RE|BCL6|IL1R1|IL18R1|TLR6|TICAM2|ACKR2|CCL5|CCL16|TNFAIP3|CCL4 | 77 | 485|561|558|500|322|345|153|356|321|538|149|308|283|342|289|465|310|274|287|305|329|173|135|501|164|118 | 7.69E-14 |  | KW-0395 | 1.136855623 | 0 |
| 1758 | 93 | GO Process |  | positive regulation of macromolecule biosynthetic process | 4.55E-12 | TNFRSF1A|MAPK1|TGFB1|TNFAIP1|NFKB1|BCL9|TGFB3|RHOQ|RASL11A|CCR7|LIF|PIAS1|CCL21|PDGFRB|SMAD7|SMAD2|NOX4|IL1A|IL1B|ATF2|IL11|STAT3|CCAR1|IRF8|IL9|SMAD6|TNFRSF13C|CREBRF|NFATC3|MAP2K1|PPARD|TNIP1|NFATC2IP|PAWR|IL25|IKZF1|SMAD3|BCL9L|IL17F|BCL11A|SMAD4|IKZF3|MAP2K3|RBPJ|BCL11B|IRF5|CCL20|MAP3K5|ILF2|TGFB2|IRF6|MAPKAPK2|IRAK1|BCL10|AGO1|CXCR3|TLR4|TGFBR1|HIF3A|NFIB|IRF4|IL33|JAK2|MAP3K4|NFATC2|MAP2K7|TNFSF11|PPARA|IL6|MAP3K2|NFATC4|TLR6|NCOA2|ILF3|MAP2K4|IL10|IKZF2|SMAD1|IKBKB|TRAF6|BCLAF1|HIF1A|SMAD5|NKRF|TET2|MAPKAPK5|NFAT5|LITAF|IER2|PIAS2|MAP2K6|CCL5|NOS1 | 77 | 351|469|350|280|529|175|265|552|205|243|432|554|500|506|195|200|364|322|345|189|126|334|111|398|295|369|224|296|210|292|227|538|347|396|478|220|241|125|367|507|470|392|528|438|128|160|308|335|443|376|487|268|434|323|239|230|342|368|510|170|169|346|309|466|115|236|430|491|218|374|249|305|455|110|517|425|486|193|526|315|180|495|201|291|294|370|393|395|354|284|255|135|508 | 1.55E-13 |  | GO.0010557 | 1.13419886 | 0 |
| 193 | 28 | GO Process |  | positive regulation of T cell activation | 5.27E-12 | TGFB1|TNFSF9|CCR7|CCL21|MAP3K8|IL1B|IL7|TNFSF4|TNFRSF13C|IL12A|CTLA4|SOCS5|IL7R|ICOS|SOCS1|BTLA|PDCD1|TGFBR2|BCL10|TNFSF13B|IL6ST|PDCD1LG2|TNFSF11|BCL6|IL6|HLA-DMB|TRAF6|CCL5 | 77 | 350|120|243|500|165|345|266|383|224|519|183|226|176|167|460|449|537|332|323|326|391|373|430|310|218|553|315|135 | 1.80E-13 |  | GO.0050870 | 1.127818938 | 0 |
| 260 | 32 | GO Process |  | positive regulation of lymphocyte activation | 5.69E-12 | TGFB1|TNFSF9|CCR7|CCL21|MAP3K8|IL1B|IL7|TNFSF4|TNFRSF13C|IL12A|CTLA4|IL13|SOCS5|IL7R|ICOS|SOCS1|LAMP1|BTLA|PDCD1|TGFBR2|BCL10|TNFSF13B|IL6ST|NFATC2|PDCD1LG2|BCL2|TNFSF11|BCL6|IL6|HLA-DMB|TRAF6|CCL5 | 77 | 350|120|243|500|165|345|266|383|224|519|183|418|226|176|167|460|360|449|537|332|323|326|391|115|373|475|430|310|218|553|315|135 | 1.96E-13 |  | GO.0051251 | 1.124488773 | 0 |
| 1793 | 94 | GO Function | | molecular function regulator | 6.21E-12 | CX3CL1|TGFB1|CCL1|CCL13|CXCL6|CDKN1B|TGFB3|TNFSF10|TNFSF9|LIF|DOCK7|PXDN|PDGFRA|DOCK10|IL36G|IL36B|CCL21|PDGFRB|FAF2|IL1A|IL1B|IL7|IL11|PRDX5|DOCK3|BNIP2|IL9|DOCK11|DOCK5|DOCK1|TNFSF4|BAG4|TGFA|CXCL3|CXCL5|RICTOR|TNFRSF11B|MAP2K1|IL16|IL12A|IL13|SOCS5|CXCL11|CFLAR|PRKRA|CSF1|IL25|SOCS1|SOCS3|CXCL14|IL17F|BAG5|CCL20|CCL28|CXCL9|TGFB2|TAGAP|IL20|BAG3|MTCP1|IRAK1|BAG2|IGSF1|IL2RG|TNFSF13B|DOCK9|CCL7|IL33|JAK2|TANK|IL36RN|SOCS4|MAPK8|CXCL12|SOCS6|BCL2|TNFSF11|CSF2RB|IL6|OSGIN2|DOCK4|IL10|TNFAIP8|IL22|APAF1|TXNIP|IKBKE|CCL5|NOS1|CCL16|SOCS2|SOCS7|CCL4|MAP3K14 | 77 | 325|350|484|485|531|285|265|202|120|432|413|543|516|505|561|558|500|506|458|322|345|266|126|208|225|187|295|422|406|215|383|409|279|356|355|302|359|292|385|519|418|226|321|133|196|149|478|460|246|468|367|124|308|488|283|376|139|161|318|162|434|116|132|563|326|300|289|346|309|314|536|523|282|181|244|475|430|524|218|446|544|425|185|534|148|415|548|135|508|501|419|186|118|171 | 1.84E-13 |  | GO.0098772 | 1.12069084 | 0 |
| 2999 | 132 | GO Process |  | cellular protein modification process | 6.39E-12 | MAP4K5|TGFBR3|MAPK1|TGFB1|TNFAIP1|NFKB1|CDKN1B|LOX|NKTR|TRAF2|LIF|PIAS1|MAP3K10|IRAK2|PDGFRA|TBRG4|PDGFRB|MAPK6|LOXL1|MMD|SMAD7|SMAD2|SMURF2|MAP3K8|IL1B|MAP4K3|ATF2|MAP3K13|CDK6|ATG10|ATG3|CSF1R|PEX13|IL17RD|RICTOR|HIF1AN|TMX3|MAP2K1|MAEA|SOCS5|MAP3K11|ATG4D|OXSR1|TNIP1|PRKRA|AATK|NFATC2IP|CSF1|SOCS1|SOCS3|SMAD3|BCL11A|SMAD4|MAP4K4|MAP2K3|MAP3K15|ATG7|AREL1|PEX2|TGFBR2|MAP3K5|MAPK10|SMURF1|MAP3K3|KIAA1804|TGFB2|MAPKAPK2|TAB2|ATG5|CACUL1|MAP3K7|IRAK1|BCL10|YWHAB|TLR4|IL2RG|TGFBR1|ZAK|MAP3K19|HIF3A|BCOR|TAB3|IRF4|IL33|JAK2|TNFRSF19|LOXL2|ATG16L1|MAP3K4|AKTIP|SOCS4|ULK2|MAPK8|SOCS6|MAP2K7|BCL2|TNFSF11|MAP3K1|MAPK4|CSF2RB|IL6|LTBP1|MAP3K2|IRAK4|PEX5|MAPK9|ITK|TNIK|ILF3|MAP2K4|ATG12|SMAD1|IKBKB|TRAF6|HIF1A|SMAD5|TET2|MAP3K12|MAPKAPK5|MAP3K9|TRAF3|IKBKE|PIAS2|ERC1|MAP2K6|CCL5|NOS1|SOCS2|TNFAIP3|SOCS7|PEX12|MAP3K14 | 77 | 451|447|469|350|280|529|285|168|117|444|432|554|489|258|516|496|506|235|498|427|195|200|362|165|345|141|189|490|365|155|113|153|152|136|302|228|122|292|207|226|439|213|504|538|196|560|347|149|460|246|241|507|470|254|528|384|119|437|269|332|335|380|464|299|190|376|268|184|312|522|358|434|323|440|342|563|368|264|352|510|541|206|169|346|309|256|480|223|466|424|523|214|282|244|236|475|430|397|231|524|218|154|374|371|514|250|556|535|110|517|421|193|526|315|495|201|294|426|370|290|557|548|284|401|255|135|508|419|164|186|229|171 | 2.21E-13 |  | GO.0006464 | 1.119449914 | 0 |
| 133 | 23 | KEGG Pathways | | Measles | 8.99E-12 | NFKB1|CDKN1B|TNFSF10|IL1A|IL1B|STAT3|CDK6|TNFRSF10B|IL12A|IL13|IFNAR2|TAB2|MAP3K7|IRAK1|TLR4|IL2RG|JAK2|IRF9|IL6|IRAK4|TRAF6|IKBKE|TNFAIP3 | 77 | 529|285|202|322|345|334|365|410|519|418|452|184|358|434|342|563|309|492|218|371|315|548|164 | 1.10E-12 |  | hsa05162 | 1.104624031 | 0 |
| 1876 | 96 | GO Process |  | positive regulation of biosynthetic process | 1.08E-11 | TNFRSF1A|MAPK1|TGFB1|TNFAIP1|NFKB1|BCL9|TGFB3|RHOQ|RASL11A|CCR7|LIF|PIAS1|CCL21|PDGFRB|SMAD7|SMAD2|NOX4|IL1A|IL1B|ATF2|IL11|STAT3|PRDX5|CCAR1|IRF8|IL9|SMAD6|TNFRSF13C|CREBRF|AVPR1A|NFATC3|MAP2K1|PPARD|TNIP1|NFATC2IP|PAWR|IL25|IKZF1|SMAD3|BCL9L|IL17F|BCL11A|SMAD4|IKZF3|MAP2K3|RBPJ|BCL11B|IRF5|CCL20|MAP3K5|ILF2|NOS1AP|TGFB2|IRF6|MAPKAPK2|IRAK1|BCL10|AGO1|CXCR3|TLR4|TGFBR1|HIF3A|NFIB|IRF4|IL33|JAK2|MAP3K4|NFATC2|MAP2K7|TNFSF11|PPARA|IL6|MAP3K2|NFATC4|TLR6|NCOA2|ILF3|MAP2K4|IL10|IKZF2|SMAD1|IKBKB|TRAF6|BCLAF1|HIF1A|SMAD5|NKRF|TET2|MAPKAPK5|NFAT5|LITAF|IER2|PIAS2|MAP2K6|CCL5|NOS1 | 77 | 351|469|350|280|529|175|265|552|205|243|432|554|500|506|195|200|364|322|345|189|126|334|208|111|398|295|369|224|296|222|210|292|227|538|347|396|478|220|241|125|367|507|470|392|528|438|128|160|308|335|443|212|376|487|268|434|323|239|230|342|368|510|170|169|346|309|466|115|236|430|491|218|374|249|305|455|110|517|425|486|193|526|315|180|495|201|291|294|370|393|395|354|284|255|135|508 | 3.76E-13 |  | GO.0009891 | 1.096657624 | 0 |
| 126 | 23 | GO Process |  | positive regulation of JNK cascade | 1.15E-11 | MAP4K5|CCR7|TRAF2|MAP3K10|CCL21|TRAF4|IL1B|MAP3K11|MAP3K5|KIAA1804|MAP3K7|ZAK|TNFRSF19|MAP3K4|MAP2K7|TNFSF11|MAP3K2|TLR6|TNIK|MAP2K4|TRAF6|MAP3K9|TNFRSF11A | 77 | 451|243|444|489|500|194|345|439|335|190|358|264|256|466|236|430|374|305|535|517|315|290|163 | 4.05E-13 |  | GO.0046330 | 1.093930216 | 0 |
| 127 | 23 | GO Process |  | positive regulation of leukocyte migration | 1.33E-11 | TGFB1|CCL1|CXCL6|CCR7|CCL21|IL1A|CXCL3|CXCL5|IL12A|CXCL11|CSF1|CXCL14|GPR29|CCL20|CXCL9|IL6R|CCL7|CXCL12|CMKBR6|IL6|IL1R1|CCL5|CCL4 | 77 | 350|484|531|243|500|322|356|355|519|321|149|468|448|308|283|348|289|181|431|218|274|135|118 | 4.69E-13 |  | GO.0002687 | 1.087614836 | 0 |
| 84 | 19 | KEGG Pathways | | Rheumatoid arthritis | 1.37E-11 | TGFB1|CXCL6|TGFB3|IL1A|IL1B|IL11|CXCL5|CTLA4|CSF1|CCL20|TGFB2|TLR4|TNFSF13B|CXCL12|TNFSF11|IL6|HLA-DMB|TNFRSF11A|CCL5 | 77 | 350|531|265|322|345|126|355|183|149|308|376|342|326|181|430|218|553|163|135 | 1.74E-12 |  | hsa05323 | 1.086327943 | 0 |
| 156 | 25 | GO Process |  | cellular response to molecule of bacterial origin | 1.43E-11 | MAPK1|TGFB1|CXCL6|NFKB1|IRAK2|IL1B|IRF8|PDCD4|TNFSF4|IL12A|PPARD|TNIP1|CCL20|IRAK1|TLR4|TNFRSF1B|MAPK8|IL6|TLR6|IL10|TICAM2|TRAF6|LITAF|CCL5|TNFAIP3 | 77 | 469|350|531|529|258|345|398|311|383|519|227|538|308|434|342|276|282|218|305|425|329|315|395|135|164 | 5.10E-13 |  | GO.0071219 | 1.084466396 | 0 |
| 1144 | 70 | GO Process |  | locomotion | 1.45E-11 | CX3CL1|TGFBR3|MAPK1|TGFB1|CCL1|CCL13|TNFAIP1|CXCL6|CCR7|PDGFRA|CCL21|PDGFRB|IL1B|ACKR3|TNFRSF10B|DOCK1|CSF1R|CXCR5|PEX13|CXCL3|CXCL5|DEFB1|MAP2K1|IL16|IL12A|DEFB4A|ALCAM|CXCL11|PPARD|TNFRSF12A|CCR4|CXCL14|SMAD4|GPR29|BCL11B|CCRL2|CCR9|CCL20|CCL28|CXCL9|TGFB2|IL6R|CXCR3|TGFBR1|CCL7|NFIB|JAK2|LOXL2|LYST|PDCD10|CXCL12|NFATC2|TNFSF11|CMKBR6|IL6|CXCR4|IRAK4|CXCR6|DOCK4|IL10|ACKR2|DEFB4B|HIF1A|TNFRSF11A|IER2|CCL5|CCL16|ECSCR|SOCS7|CCL4 | 77 | 325|447|469|350|484|485|280|531|243|516|500|506|345|247|410|215|153|306|152|356|355|112|292|385|519|158|286|321|227|340|502|468|470|448|128|134|456|308|488|283|376|348|230|368|289|170|309|480|555|316|181|115|430|431|218|450|371|435|544|425|173|166|495|163|354|135|501|253|186|118 | 5.21E-13 |  | GO.0040011 | 1.0838632 | 0 |
| 188 | 27 | GO Process |  | regulation of chemotaxis | 1.69E-11 | TGFB1|CCL1|CXCL6|CCR7|PDGFRA|CCL21|PDGFRB|CXCL3|CXCL5|IL16|IL12A|CXCL11|CSF1|CCR4|SMAD3|CXCL14|GPR29|CXCL9|IL6R|CXCR3|CCL7|CXCL12|CMKBR6|IL6|CXCR4|CCL5|CCL4 | 77 | 350|484|531|243|516|500|506|356|355|385|519|321|149|502|241|468|448|283|348|230|289|181|431|218|450|135|118 | 6.09E-13 |  | GO.0050920 | 1.07721133 | 0 |
| 42 | 16 | SMART Domains | | Intercrine alpha family (small cytokine C-X-C) (chemokine CXC). | 1.74E-11 | CX3CL1|CCL1|CCL13|CXCL6|CCL21|CXCL3|CXCL5|CXCL11|CCL20|CCL28|CXCL9|CCL7|CXCL12|CCL5|CCL16|CCL4 | 77 | 325|484|485|531|500|356|355|321|308|488|283|289|181|135|501|118 | 1.30E-13 |  | SM00199 | 1.075945075 | 0 |
| 74 | 18 | KEGG Pathways | | Pertussis | 1.78E-11 | MAPK1|CXCL6|NFKB1|IL1A|IL1B|IRF8|CXCL5|IL12A|MAPK10|IRAK1|TLR4|MAPK8|IL6|IRAK4|MAPK9|IL10|TICAM2|TRAF6 | 77 | 469|531|529|322|345|398|355|519|380|434|342|282|218|371|250|425|329|315 | 2.34E-12 |  | hsa05133 | 1.074958 | 0 |
| 2524 | 116 | GO Process |  | regulation of localization | 2.05E-11 | CX3CL1|TNFRSF1A|TGFBR3|MAPK1|TGFB1|CCL1|CXCL6|NFKB1|CRTAM|CDKN1B|IL1RL1|TGFB3|RHOQ|CCR7|TRAF2|LIF|DOCK7|XPO4|PDGFRA|HILPDA|DOCK10|CCL21|ARG2|PDGFRB|MMD|MSR1|SMAD7|RIMS2|SMURF2|NOX4|IL1A|IL1B|IL11|STAT3|CDK6|CCAR1|ACKR3|DOCK5|DOCK1|TNFSF4|ATG3|CSF1R|BAG4|IL17RB|TRAT1|CXCL3|CXCL5|TNFRSF21|CREBRF|DEFB1|AVPR1A|MAP2K1|BCL2L1|IL16|IL12A|IL13|CXCL11|PPARD|OXSR1|IL1RAP|RHOJ|CSF1|SMAD3|LAMP1|CXCL14|IL17F|SMAD4|MAP4K4|GPR29|MAP2K3|CCL20|TGFBR2|CPLX2|CCL28|CXCL9|MAP3K3|NOS1AP|TGFB2|IL6R|ATG5|BAG3|YWHAB|CXCR3|TLR4|TGFBR1|TNFRSF1B|CCL7|BNIP3L|IL33|JAK2|PDCD10|IL36RN|MAPK8|CXCL12|BCL2|TNFSF11|CMKBR6|BCL6|PPARA|IL6|IL1R1|TLR6|MAPKBP1|DOCK4|PDCD6IP|IL10|TRAF6|HIF1A|SOD2|IKBKE|TNFRSF11A|MAP2K6|CCL5|NOS1|TNFRSF9|CCL4 | 77 | 325|351|447|469|350|484|531|529|151|285|336|265|552|243|444|432|413|386|516|462|505|500|518|506|427|147|195|331|362|364|322|345|126|334|365|111|247|406|215|383|113|153|409|497|527|356|355|513|296|112|222|292|372|385|519|418|321|227|504|199|375|149|241|360|468|367|470|254|448|528|308|332|216|488|283|299|212|376|348|312|318|440|230|342|368|276|289|499|346|309|316|536|282|181|475|430|431|310|491|218|274|305|298|544|307|425|315|495|381|548|163|255|135|508|473|118 | 7.44E-13 |  | GO.0032879 | 1.068824614 | 0 |
| 15 | 12 | InterPro Domains | | SMAD-like domain superfamily | 2.07E-11 | SMAD7|SMAD2|IRF8|SMAD6|SMAD3|SMAD4|IRF5|IRF6|IRF4|IRF9|SMAD1|SMAD5 | 77 | 195|200|398|369|241|470|160|487|169|492|193|201 | 2.10E-13 |  | IPR017855 | 1.068402965 | 0 |
| 175 | 26 | GO Process |  | regulation of leukocyte migration | 2.25E-11 | TGFB1|CCL1|CXCL6|CCR7|CCL21|IL1A|CXCL3|CXCL5|IL12A|CXCL11|CSF1|CXCL14|GPR29|CCL20|CCL28|CXCL9|IL6R|CXCR3|CCL7|IL33|CXCL12|CMKBR6|IL6|IL1R1|CCL5|CCL4 | 77 | 350|484|531|243|500|322|356|355|519|321|149|468|448|308|488|283|348|230|289|346|181|431|218|274|135|118 | 8.22E-13 |  | GO.0002685 | 1.064781748 | 0 |
| 1876 | 95 | GO Process |  | regulation of multicellular organismal development | 2.52E-11 | CX3CL1|TNFRSF1A|TGFBR3|TGFB1|CDKN1B|TGFB3|TNFSF9|LIF|DOCK7|PDGFRB|MAPK6|MMD|SMAD7|SMAD2|RIMS2|SMURF2|IL1A|IL1B|IL7|ATF2|STAT3|CDK6|SOD1|PDCD4|TNFSF4|TNFRSF21|TNFRSF11B|HIF1AN|NFATC3|MAP2K1|IL12A|CTLA4|IL13|SOCS5|IL7R|PPARD|CFLAR|IL1RAP|RHOJ|C8orf4|TNFRSF12A|CSF1|SOCS1|SMAD3|PDCD1|BCL9L|IL17F|BCL11A|BAG5|SMAD4|MAP4K4|IKZF3|RBPJ|BCL11B|TGFBR2|SMURF1|MAP3K3|TGFB2|IL20|AGO3|AGO1|AGO4|TRAF3IP1|CXCR3|TGIF2|TLR4|TGFBR1|BCOR|NFIB|IRF4|IL6ST|LOXL2|PDCD10|ULK2|CXCL12|NFATC2|BCL2|TNFSF11|BCL6|IL6|CXCR4|NFATC4|HLA-B|TNIK|IL10|SMAD1|IKBKB|TRAF6|DICER1|HIF1A|SOD2|NOS1|ECSCR|SOCS2|TNFAIP3 | 77 | 325|351|447|350|285|265|120|432|413|506|235|427|195|200|331|362|322|345|266|189|334|365|281|311|383|513|359|228|210|292|519|183|418|226|176|227|133|199|375|293|340|149|460|241|537|125|367|507|124|470|254|392|438|128|332|464|299|376|161|520|239|240|474|230|259|342|368|541|170|169|391|480|316|214|181|115|475|430|310|218|450|249|114|535|425|193|526|315|121|495|381|508|253|419|164 | 9.25E-13 |  | GO.2000026 | 1.059859946 | 0 |
| 146 | 24 | GO Process |  | cellular response to lipopolysaccharide | 2.52E-11 | MAPK1|TGFB1|CXCL6|NFKB1|IRAK2|IL1B|IRF8|PDCD4|TNFSF4|IL12A|PPARD|TNIP1|CCL20|IRAK1|TLR4|TNFRSF1B|MAPK8|IL6|IL10|TICAM2|TRAF6|LITAF|CCL5|TNFAIP3 | 77 | 469|350|531|529|258|345|398|311|383|519|227|538|308|434|342|276|282|218|425|329|315|395|135|164 | 9.24E-13 |  | GO.0071222 | 1.059859946 | 0 |
| 1846 | 94 | GO Process |  | positive regulation of cellular biosynthetic process | 2.52E-11 | TNFRSF1A|MAPK1|TGFB1|TNFAIP1|NFKB1|BCL9|TGFB3|RHOQ|RASL11A|CCR7|LIF|PIAS1|CCL21|PDGFRB|SMAD7|SMAD2|NOX4|IL1A|IL1B|ATF2|IL11|STAT3|CCAR1|IRF8|IL9|SMAD6|TNFRSF13C|CREBRF|AVPR1A|NFATC3|MAP2K1|PPARD|TNIP1|NFATC2IP|PAWR|IL25|IKZF1|SMAD3|BCL9L|IL17F|BCL11A|SMAD4|IKZF3|MAP2K3|RBPJ|BCL11B|IRF5|CCL20|MAP3K5|ILF2|NOS1AP|TGFB2|IRF6|MAPKAPK2|IRAK1|BCL10|AGO1|CXCR3|TLR4|TGFBR1|HIF3A|NFIB|IRF4|IL33|JAK2|MAP3K4|NFATC2|MAP2K7|TNFSF11|PPARA|IL6|MAP3K2|NFATC4|TLR6|NCOA2|ILF3|MAP2K4|IL10|IKZF2|SMAD1|IKBKB|TRAF6|BCLAF1|HIF1A|SMAD5|NKRF|TET2|MAPKAPK5|NFAT5|LITAF|IER2|PIAS2|CCL5|NOS1 | 77 | 351|469|350|280|529|175|265|552|205|243|432|554|500|506|195|200|364|322|345|189|126|334|111|398|295|369|224|296|222|210|292|227|538|347|396|478|220|241|125|367|507|470|392|528|438|128|160|308|335|443|212|376|487|268|434|323|239|230|342|368|510|170|169|346|309|466|115|236|430|491|218|374|249|305|455|110|517|425|486|193|526|315|180|495|201|291|294|370|393|395|354|284|135|508 | 9.29E-13 |  | GO.0031328 | 1.059859946 | 0 |
| 71 | 18 | GO Process |  | positive regulation of JUN kinase activity | 3.46E-11 | MAP4K5|TRAF2|MAP3K10|MAP3K11|MAP3K5|KIAA1804|MAP3K7|ZAK|MAP3K4|MAP2K7|TNFSF11|MAP3K2|TLR6|TNIK|MAP2K4|TRAF6|MAP3K9|TNFRSF11A | 77 | 451|444|489|439|335|190|358|264|466|236|430|374|305|535|517|315|290|163 | 1.29E-12 |  | GO.0043507 | 1.04609239 | 0 |
| 122 | 22 | GO Process |  | positive regulation of I-kappaB kinase/NF-kappaB signaling | 4.38E-11 | TNFRSF1A|TNFSF10|CCR7|TRAF2|CCL21|TRAF5|IL1B|TNFRSF10B|CFLAR|MAP3K3|TAB2|MAP3K7|IRAK1|BCL10|TNFRSF19|TNFSF11|TLR6|IRAK4|IKBKB|TRAF6|IKBKE|MAP3K14 | 77 | 351|202|243|444|500|142|345|410|133|299|184|358|434|323|256|430|305|371|526|315|548|171 | 1.64E-12 |  | GO.0043123 | 1.035852589 | 0 |
| 573 | 46 | GO Process |  | hematopoietic or lymphoid organ development | 4.61E-11 | TGFBR3|MAPK1|TGFB1|CCR7|PDGFRA|DOCK10|IL7|IL11|STAT3|IRF8|SOD1|DOCK11|CSF1R|CXCR5|MAP2K1|MAEA|IL7R|CSF1|IL25|IKZF1|GPR29|RBPJ|BCL11B|CCR9|TGFBR2|TGFB2|THEMIS|ATG5|TGFBR1|IRF4|JAK2|BCL2L11|CIAPIN1|BCL2|TNFSF11|CMKBR6|BCL6|IL6|IL18R1|ITK|IL10|TRAF6|HIF1A|SMAD5|TET2|TNFRSF11A | 77 | 447|469|350|243|516|505|266|126|334|398|281|422|153|306|292|207|176|149|478|220|448|438|128|456|332|376|349|312|368|169|309|234|472|475|430|431|310|218|287|556|425|315|495|201|294|163 | 1.74E-12 |  | GO.0048534 | 1.033629907 | 0 |
| 194 | 26 | KEGG Pathways | | Epstein-Barr virus infection | 4.91E-11 | NFKB1|CDKN1B|TRAF2|TRAF5|ATF2|STAT3|MAP2K3|RBPJ|MAPK10|TAB2|MAP3K7|IRAK1|YWHAB|MAPK8|MAP2K7|BCL2|MAPK9|HLA-B|MAP2K4|IL10|IKBKB|TRAF6|TRAF3|MAP2K6|TNFAIP3|MAP3K14 | 77 | 529|285|444|142|189|334|528|438|380|184|358|434|440|282|236|475|250|114|517|425|526|315|557|255|164|171 | 6.73E-12 |  | hsa05169 | 1.030891851 | 0 |
| 669 | 50 | GO Process |  | negative regulation of cell population proliferation | 5.54E-11 | TGFBR3|TGFB1|CDKN1B|TGFB3|LIF|ARG2|SMAD2|NOX4|IL1A|IL1B|ATF2|STAT3|CDK6|PDCD4|CSF1R|SMAD6|TNFRSF21|MAP2K1|IL12A|CTLA4|PPARD|PRKRA|IFNLR1|PAWR|SMAD3|SMAD4|RBPJ|PEX2|BCL11B|TGFBR2|TGFB2|IRF6|IFIT3|CXCR3|TGFBR1|NFIB|JAK2|PDCD10|PDCD1LG2|BCL2|BCL6|IL6|TBRG1|IL10|SMAD1|AMBRA1|DICER1|SOD2|TNFRSF9|TNFAIP3 | 77 | 447|350|285|265|432|518|200|364|322|345|189|334|365|311|153|369|513|292|519|183|227|196|341|396|241|470|438|269|128|332|376|487|404|230|368|170|309|316|373|475|310|218|445|425|193|131|121|381|473|164 | 2.09E-12 |  | GO.0008285 | 1.025649024 | 0 |
| 94 | 20 | UniProt Keywords | | Chemotaxis | 5.56E-11 | CX3CL1|CCL1|CCL13|CXCL6|PDGFRA|CCL21|PDGFRB|CXCL3|IL16|CXCL11|CXCL14|CCL20|CCL28|CXCR3|CCL7|CXCL12|CCL5|CCL16|ECSCR|CCL4 | 77 | 325|484|485|531|516|500|506|356|385|321|468|308|488|230|289|181|135|501|253|118 | 1.20E-12 |  | KW-0145 | 1.025492521 | 0 |
| 69 | 17 | KEGG Pathways | | Prolactin signaling pathway | 5.68E-11 | MAPK1|NFKB1|STAT3|MAP2K1|SOCS5|SOCS1|SOCS3|MAPK10|JAK2|SOCS4|MAPK8|SOCS6|TNFSF11|MAPK9|TNFRSF11A|SOCS2|SOCS7 | 77 | 469|529|334|292|226|460|246|380|309|523|282|244|430|250|163|419|186 | 8.07E-12 |  | hsa04917 | 1.024565166 | 0 |
| 623 | 48 | GO Process |  | regulation of cell adhesion | 5.75E-11 | CX3CL1|TGFB1|TNFSF9|CCR7|CCL21|ARG2|SMAD7|MAP3K8|IL1B|IL7|CDK6|DOCK5|DOCK1|TNFSF4|BAG4|TNFRSF13C|TNFRSF21|IL12A|CTLA4|SOCS5|IL7R|ICOS|CSF1|PAWR|SOCS1|SMAD3|BTLA|PDCD1|TGFBR2|CCL28|TGFB2|BCL10|TNFSF13B|IL6ST|JAK2|CXCL12|PDCD1LG2|SOCS6|BCL2|TNFSF11|BCL6|PPARA|IL6|HLA-DMB|IL10|TGFBI|TRAF6|CCL5 | 77 | 325|350|120|243|500|518|195|165|345|266|365|406|215|383|409|224|513|519|183|226|176|167|149|396|460|241|449|537|332|488|376|323|326|391|309|181|373|244|475|430|310|491|218|553|425|157|315|135 | 2.19E-12 |  | GO.0030155 | 1.024033216 | 0 |
| 365 | 36 | GO Process |  | immune response-regulating signaling pathway | 6.35E-11 | MAPK1|NFKB1|IRAK2|DOCK1|TRAT1|TNFRSF21|NFATC3|CTLA4|TNIP1|MAPK10|MAPKAPK2|TAB2|THEMIS|MAP3K7|IRAK1|BCL10|TLR4|HLA-DQB1|TAB3|TANK|MAPK8|NFATC2|BCL2|MAP3K1|TLR6|IRAK4|MAPK9|ITK|TICAM2|LAT2|IKBKB|TRAF6|TRAF3|IKBKE|MAP2K6|TNFAIP3 | 77 | 469|529|258|215|527|513|210|183|538|380|268|184|349|358|434|323|342|109|206|314|282|115|475|397|305|371|250|556|329|467|526|315|557|548|255|164 | 2.42E-12 |  | GO.0002764 | 1.019722627 | 0 |
| 169 | 25 | GO Process |  | regulation of JNK cascade | 6.47E-11 | MAP4K5|CCR7|TRAF2|MAP3K10|CCL21|TRAF4|IL1B|PDCD4|MAP3K11|MAP4K4|MAP3K5|KIAA1804|MAP3K7|ZAK|TNFRSF19|MAP3K4|MAP2K7|TNFSF11|MAP3K2|TLR6|TNIK|MAP2K4|TRAF6|MAP3K9|TNFRSF11A | 77 | 451|243|444|489|500|194|345|311|439|254|335|190|358|264|256|466|236|430|374|305|535|517|315|290|163 | 2.48E-12 |  | GO.0046328 | 1.018909572 | 0 |
| 86 | 19 | GO Process |  | regulation of JUN kinase activity | 6.47E-11 | MAP4K5|TRAF2|MAP3K10|PDCD4|MAP3K11|MAP3K5|KIAA1804|MAP3K7|ZAK|MAP3K4|MAP2K7|TNFSF11|MAP3K2|TLR6|TNIK|MAP2K4|TRAF6|MAP3K9|TNFRSF11A | 77 | 451|444|489|311|439|335|190|358|264|466|236|430|374|305|535|517|315|290|163 | 2.49E-12 |  | GO.0043506 | 1.018909572 | 0 |
| 70 | 17 | KEGG Pathways | | Leishmaniasis | 6.67E-11 | MAPK1|TGFB1|NFKB1|TGFB3|IL1A|IL1B|IL12A|TGFB2|TAB2|MAP3K7|IRAK1|TLR4|JAK2|IRAK4|HLA-DMB|IL10|TRAF6 | 77 | 469|350|529|265|322|345|519|376|184|358|434|342|309|371|553|425|315 | 9.82E-12 |  | hsa05140 | 1.017587417 | 0 |
| 250 | 29 | KEGG Pathways | | HTLV-I infection | 6.68E-11 | TNFRSF1A|TGFB1|NFKB1|TGFB3|PDGFRA|PDGFRB|SMAD2|ATF2|TNFRSF13C|NFATC3|BCL2L1|SMAD3|SMAD4|TGFBR2|MAP3K3|TGFB2|IL2RG|TGFBR1|MAPK8|NFATC2|MAP3K1|IL6|IL1R1|NFATC4|HLA-DMB|HLA-B|MAP2K4|IKBKB|MAP3K14 | 77 | 351|350|529|265|516|506|200|189|224|210|372|241|470|332|299|376|563|368|282|115|397|218|274|249|553|114|517|526|171 | 1.02E-11 |  | hsa05166 | 1.017522354 | 0 |
| 1052 | 65 | GO Process |  | response to abiotic stimulus | 6.87E-11 | NOX3|TNFRSF1A|TGFBR3|TGFB1|NFKB1|CDKN1B|TGFB3|PIAS1|HILPDA|PDGFRB|NOX4|IL1A|IL1B|MAP4K3|ATF2|SOD1|TNFRSF10B|AIFM1|IKBIP|HIF1AN|AVPR1A|BCL2L1|IL12A|IL13|PPARD|OXSR1|CFLAR|C8orf4|AEN|CCR4|SMAD3|SMAD4|RBPJ|ATG7|TGFBR2|MAPK10|TGFB2|BAG3|IRAK1|BCL10|TLR4|ZAK|HIF3A|CCL7|BNIP3L|LOXL2|MAP3K4|TANK|MAPK8|CXCL12|MAP2K7|BCL2|MAP3K1|PPARA|CXCR4|MAP3K2|NFATC4|MAPK9|MAP2K4|HIF1A|APAF1|TXNIP|TNFRSF11A|NOS1|MAP3K14 | 77 | 203|351|447|350|529|285|265|554|462|506|364|322|345|141|189|281|410|482|232|228|222|372|519|418|227|504|133|293|547|502|241|470|438|119|332|380|376|318|434|323|342|264|510|289|499|480|466|314|282|181|236|475|397|491|450|374|249|250|517|495|148|415|163|508|171 | 2.66E-12 |  | GO.0009628 | 1.016304326 | 0 |
| 83 | 18 | KEGG Pathways | | TGF-beta signaling pathway | 7.84E-11 | MAPK1|TGFB1|TGFB3|SMAD7|SMAD2|SMURF2|SMAD6|TGIF1|SMAD3|SMAD4|TGFBR2|SMURF1|TGFB2|TGIF2|TGFBR1|LTBP1|SMAD1|SMAD5 | 77 | 469|350|265|195|200|362|369|143|241|470|332|464|376|259|368|154|193|201 | 1.23E-11 |  | hsa04350 | 1.010568394 | 0 |
| 142 | 23 | GO Process |  | positive regulation of NF-kappaB transcription factor activity | 9.33E-11 | TGFB1|NFKB1|TRAF2|IRAK2|TRAF5|IL1B|MAP3K13|CFLAR|IL1RAP|TAB2|MAP3K7|IRAK1|BCL10|TLR4|TAB3|TNFSF11|IL18R1|TLR6|IRAK4|IKBKB|TRAF6|TNFRSF11A|ERC1 | 77 | 350|529|444|258|142|345|490|133|199|184|358|434|323|342|206|430|287|305|371|526|315|163|401 | 3.63E-12 |  | GO.0051092 | 1.003011836 | 0 |
| 296 | 32 | GO Process |  | leukocyte migration | 1.19E-10 | CX3CL1|TGFB1|CCL1|CCL13|CXCL6|CCR7|CCL21|IL1B|TNFRSF10B|CXCR5|CXCL3|IL16|CXCL11|GPR29|CCL20|CXCL9|TGFB2|IL6R|CXCR3|CCL7|LYST|CXCL12|TNFSF11|CMKBR6|IL6|CXCR4|IRAK4|IL10|TNFRSF11A|CCL5|CCL16|CCL4 | 77 | 325|350|484|485|531|243|500|345|410|306|356|385|321|448|308|283|376|348|230|289|555|181|430|431|218|450|371|425|163|135|501|118 | 4.65E-12 |  | GO.0050900 | 0.992445304 | 0 |
| 380 | 37 | InterPro Domains | | Protein kinase, ATP binding site | 1.43E-10 | MAP4K5|MAPK1|MAP3K10|PDGFRA|PDGFRB|MAPK6|MAP4K3|CDK6|CSF1R|MAP2K1|MAP3K11|OXSR1|AATK|MAP4K4|MAP2K3|MAP3K15|TGFBR2|MAP3K5|KIAA1804|MAPKAPK2|MAP3K7|IRAK1|TGFBR1|MAP3K19|JAK2|MAP3K4|ULK2|MAP3K1|MAPK4|MAP3K2|ITK|TNIK|MAP2K4|MAP3K9|IKBKE|MAP2K6|MAP3K14 | 77 | 451|469|489|516|506|235|141|365|153|292|439|504|560|254|528|384|332|335|190|268|358|434|368|352|309|466|214|397|231|374|556|535|517|290|548|255|171 | 1.69E-12 |  | IPR017441 | 0.984466396 | 0 |
| 91 | 19 | GO Process |  | positive regulation of leukocyte chemotaxis | 1.50E-10 | CCL1|CXCL6|CCR7|CCL21|CXCL3|CXCL5|IL12A|CXCL11|CSF1|CXCL14|GPR29|CXCL9|IL6R|CCL7|CXCL12|CMKBR6|IL6|CCL5|CCL4 | 77 | 484|531|243|500|356|355|519|321|149|468|448|283|348|289|181|431|218|135|118 | 5.89E-12 |  | GO.0002690 | 0.982390874 | 0 |
| 11878 | 346 | GO Function | | binding | 1.64E-10 | CX3CL1|MAP4K5|TNFRSF1A|TGFBR3|MAPK1|TGFB1|CCL1|CCL13|TNFAIP1|CXCL6|NFKB1|CRTAM|CDKN1B|EXOC2|LOX|NKTR|IL1RL1|BCL9|TGFB3|RHOQ|TNFSF10|RASL11A|EEPD1|TNFSF9|CCR7|ATG14|TRAF2|LIF|PIAS1|BCL2L2|DOCK7|LILRA1|PXDN|MAP3K10|EXOC4|XPO4|IRAK2|PDGFRA|HILPDA|DOCK10|IL36G|IL36B|CCL21|FDX1|BCL2L10|LOXL4|TRAF5|ARG2|PDGFRB|MAPK6|LOXL1|FAF2|MSR1|SMAD7|SMAD2|RIMS2|TRAF4|SMURF2|MAP3K8|DGCR2|NOX4|IL1A|IL1B|ITPKC|IL7|MAP4K3|ATF2|IL11|STAT3|MAP3K13|PECR|PRDX5|CDK6|CCAR1|DOCK3|ETNK1|BNIP2|ADAMTS17|IRF8|SOD1|IL22RA1|ACKR3|IL9|DOCK11|TNFRSF10B|DOCK5|ENDOD1|PDCD4|DOCK1|TNFSF4|ATG3|CSF1R|AIFM1|BAG4|GATAD1|SMAD6|TGFA|TRAT1|CXCL3|CXCL5|SNIP1|RICTOR|CREBRF|IL22RA2|TNFRSF11B|DEFB1|HIF1AN|AVPR1A|HPS6|NFATC3|LTBP3|MAP2K1|BCL2L1|MAEA|IL16|IL12A|DEFB4A|NKIRAS2|IL13|SOCS5|ALCAM|IL7R|CXCL11|MAP3K11|PPARD|OXSR1|CFLAR|IL1RAP|RHOJ|TNIP1|EEA1|PRKRA|C8orf4|MICU3|AATK|CSF1|TGIF1|TNFAIP8L3|PAWR|IL25|SOCS1|PIWIL3|SOCS3|IKZF1|AEN|SMAD3|LAMP1|BCL9L|CXCL14|IL17F|BCL11A|C1QTNF6|BAG5|SMAD4|MAP4K4|GPR29|IFNAR2|IKZF3|MAP2K3|RBPJ|MAP3K15|ATG7|PEX2|BCL11B|IRF5|CCRL2|FXR1|CCL20|TGFBR2|MAP3K5|MAPK10|CPLX2|CCL28|SMURF1|CXCL9|MAP3K3|ILF2|NOS1AP|IRF2BP2|KIAA1804|TGFB2|IRF6|TAGAP|IL20|MAPKAPK2|TAB2|PEX3|HIVEP2|PEX19|IL6R|GATAD2B|IKZF5|MCL1|BAG3|CACUL1|PEX11B|MAP3K7|MTCP1|SIKE1|IRAK1|BCL10|PI4K2A|BAG2|IFI44L|EXOSC1|IGSF1|LCOR|IFIT3|YWHAB|AGO3|AGO1|AGO4|TRAF3IP1|CXCR3|TGIF2|TLR4|IL2RG|HLA-DQB1|TGFBR1|ZAK|MAP3K19|TNFSF13B|TNFRSF1B|DOCK9|HIF3A|BCOR|CCL7|TAB3|BNIP3L|NFIB|IRF4|IL6ST|IL33|JAK2|TXNRD3|LOXL2|EXOSC8|ATG16L1|MAP3K4|TANK|PDCD10|IL36RN|BCL2L11|FDX1L|SUOX|PRC1|CIAPIN1|AKTIP|ULK2|MAPK8|CXCL12|NFATC2|PDXDC1|IRF9|MAP2K7|BCL2|TNFSF11|MAP3K1|MAPK4|TXNRD2|CMKBR6|CSF2RB|GATSL3|BCL6|PPARA|IL6|LTBP1|IL1R1|CXCR4|IL18R1|MAP3K2|IRGQ|NFATC4|TLR6|IRAK4|PEX5|MAPKBP1|EIF4E3|NKIRAS1|MAPK9|CXCR6|OSGIN2|ITK|HLA-DMB|HLA-B|TNIK|NCOA2|ILF3|DOCK4|LAMP2|MAP2K4|LMLN|BCL7B|PDCD6IP|IL10|IKZF2|TICAM2|TGFBI|ACKR2|PEX5L|LAT2|DEFB4B|SMAD1|CDKN2AIP|IKBKB|API5|AMBRA1|ATG13|TRAF6|BCLAF1|DICER1|HIF1A|SMAD5|NKRF|IL22|TET2|RASSF3|SOD2|APAF1|MAP3K12|MAPKAPK5|JKAMP|MAP3K9|MOAP1|TRAF3|NFAT5|LITAF|TXNIP|IKBKE|TNFRSF11A|IER2|PIAS2|ERC1|MAP2K6|CCL5|NOS1|CCL16|TNFRSF9|JAKMIP2|SOCS2|TNFAIP3|SOCS7|CCL4|PEX12|MAP3K14|GATSL2 | 77 | 325|451|351|447|469|350|484|485|280|531|529|151|285|339|168|117|336|175|265|552|202|205|197|120|243|150|444|432|554|551|413|344|543|489|159|386|258|516|462|505|561|558|500|353|550|242|142|518|506|235|498|458|147|195|200|331|194|362|165|405|364|322|345|420|266|141|189|126|334|490|436|208|365|111|225|191|187|267|398|281|211|247|295|422|410|406|204|311|215|383|113|153|482|409|530|369|279|527|356|355|337|302|296|272|359|112|228|222|209|210|441|292|372|207|385|519|158|273|418|226|286|176|321|439|227|504|133|199|375|538|146|196|293|416|560|149|143|248|396|478|460|182|246|220|547|241|360|125|468|367|507|123|124|470|254|448|452|392|528|438|384|119|269|128|160|134|245|308|332|335|380|216|488|464|283|299|443|212|178|190|376|487|139|161|268|184|423|433|521|348|414|412|278|318|522|343|358|162|338|434|323|399|116|177|137|132|233|404|440|520|239|240|474|230|259|342|563|109|368|264|352|326|276|300|510|541|289|206|499|170|169|391|346|309|263|480|297|223|466|314|316|536|234|512|262|511|472|424|214|282|181|115|330|492|236|475|430|397|231|476|431|524|288|310|491|218|154|274|450|287|374|539|249|305|371|514|298|403|417|250|435|446|556|553|114|535|455|110|544|304|517|333|174|307|425|486|329|157|173|328|467|166|193|324|526|463|131|559|315|180|121|495|201|291|534|294|221|381|148|426|370|382|290|138|557|393|395|415|548|163|354|284|401|255|135|508|501|473|251|419|164|186|118|229|171|402 | 5.35E-12 |  | GO.0005488 | 0.978515615 | 0 |
| 213 | 27 | GO Process |  | regulation of leukocyte proliferation | 2.14E-10 | TGFB1|TNFSF9|ARG2|IL1B|IL7|TNFSF4|TNFRSF13C|TNFRSF21|IL12A|CTLA4|IL13|CSF1|PAWR|IKZF3|TGFBR2|TNFSF13B|IL6ST|NFATC2|PDCD1LG2|BCL2|BCL6|IL6|HLA-DMB|IL10|TRAF6|CCL5|TNFAIP3 | 77 | 350|120|518|345|266|383|224|513|519|183|418|149|396|392|332|326|391|115|373|475|310|218|553|425|315|135|164 | 8.47E-12 |  | GO.0070663 | 0.966958623 | 0 |
| 270 | 30 | GO Process |  | response to virus | 2.98E-10 | CDK6|TNFSF4|BCL2L1|IL12A|PRKRA|IFNLR1|IFNAR2|ATG7|IRF5|CXCL9|IFI44|IFI44L|IFIT3|IFIT2|BNIP3L|IL33|LYST|BCL2L11|CXCL12|IRF9|BCL2|IL6|CXCR4|ILF3|TICAM2|IKBKB|IKBKE|CCL5|CCL4|MAP3K14 | 77 | 365|383|372|519|196|341|452|119|160|283|179|177|404|394|499|346|555|234|181|492|475|218|450|110|329|526|548|135|118|171 | 1.19E-11 |  | GO.0009615 | 0.952578374 | 0 |
| 183 | 25 | Reactome Pathways | | Peptide ligand-binding receptors | 3.07E-10 | CX3CL1|CCL1|CCL13|CXCL6|CCR7|CCL21|ACKR3|CXCR5|CXCL3|CXCL5|AVPR1A|CXCL11|CCR4|CCRL2|CCR9|CCL20|CCL28|CXCL9|CXCR3|CXCL12|CMKBR6|CXCR4|CXCR6|CCL5|CCL16 | 77 | 325|484|485|531|243|500|247|306|356|355|222|321|502|134|456|308|488|283|230|181|431|450|435|135|501 | 1.19E-11 |  | HSA-375276 | 0.951286162 | 0 |
| 135 | 21 | KEGG Pathways | | Apoptosis | 3.85E-10 | TNFRSF1A|MAPK1|NFKB1|TNFSF10|TRAF2|TNFRSF10B|AIFM1|MAP2K1|BCL2L1|CFLAR|MAP3K5|MAPK10|MCL1|BCL2L11|MAPK8|BCL2|CSF2RB|MAPK9|IKBKB|APAF1|MAP3K14 | 77 | 351|469|529|202|444|410|482|292|372|133|335|380|278|234|282|475|524|250|526|148|171 | 6.25E-11 |  | hsa04210 | 0.941453927 | 0 |
| 51 | 15 | GO Process |  | interleukin-1-mediated signaling pathway | 4.13E-10 | NFKB1|IRAK2|MAP3K8|IL1A|IL1B|IL1RAP|MAP3K3|TAB2|MAP3K7|IRAK1|TAB3|IL1R1|IRAK4|IKBKB|TRAF6 | 77 | 529|258|165|322|345|199|299|184|358|434|206|274|371|526|315 | 1.65E-11 |  | GO.0070498 | 0.938404995 | 0 |
| 41 | 14 | GO Function | | CCR chemokine receptor binding | 4.37E-10 | CX3CL1|CCL1|CCL13|CCL21|STAT3|DEFB1|DEFB4A|CCRL2|CCL20|CCL7|DEFB4B|CCL5|CCL16|CCL4 | 77 | 325|484|485|500|334|112|158|134|308|289|166|135|501|118 | 1.50E-11 |  | GO.0048020 | 0.935951856 | 0 |
| 812 | 54 | GO Process |  | cell migration | 4.41E-10 | CX3CL1|TGFBR3|TGFB1|CCL1|CCL13|TNFAIP1|CXCL6|CCR7|PDGFRA|CCL21|PDGFRB|IL1B|TNFRSF10B|DOCK1|CXCR5|PEX13|CXCL3|CXCL5|IL16|IL12A|CXCL11|PPARD|TNFRSF12A|CCR4|CXCL14|GPR29|BCL11B|CCL20|CCL28|CXCL9|TGFB2|IL6R|CXCR3|TGFBR1|CCL7|JAK2|LOXL2|LYST|PDCD10|CXCL12|NFATC2|TNFSF11|CMKBR6|IL6|CXCR4|IRAK4|DOCK4|IL10|HIF1A|TNFRSF11A|CCL5|CCL16|SOCS7|CCL4 | 77 | 325|447|350|484|485|280|531|243|516|500|506|345|410|215|306|152|356|355|385|519|321|227|340|502|468|448|128|308|488|283|376|348|230|368|289|309|480|555|316|181|115|430|431|218|450|371|544|425|495|163|135|501|186|118 | 1.77E-11 |  | GO.0016477 | 0.935556141 | 0 |
| 98 | 19 | Reactome Pathways | | Interleukin-1 signaling | 4.56E-10 | NFKB1|IRAK2|MAP3K8|IL1A|IL1B|MAP2K1|NKIRAS2|MAP3K3|TAB2|MAP3K7|IRAK1|TAB3|IL1R1|IRAK4|NKIRAS1|MAP2K4|IKBKB|TRAF6|MAP2K6 | 77 | 529|258|165|322|345|292|273|299|184|358|434|206|274|371|417|517|526|315|255 | 1.83E-11 |  | HSA-9020702 | 0.934103516 | 0 |
| 1134 | 69 | GO Component | | extracellular space | 4.97E-10 | CX3CL1|TNFRSF1A|TGFB1|CCL1|CCL13|CXCL6|LOX|SOGA1|TGFB3|TNFSF9|LIF|PXDN|HILPDA|IL36G|IL36B|CCL21|LOXL1|MSR1|IL1A|IL1B|IL7|IL11|SOD1|IL9|TNFSF4|TGFA|CXCL3|CXCL5|IL22RA2|TNFRSF11B|DEFB1|IL16|IL12A|DEFB4A|IL13|CXCL11|CSF1|IL25|CXCL14|IL17F|C1QTNF6|IFNAR2|CCL20|CCL28|CXCL9|TGFB2|IL20|IL6R|TNFRSF6B|TNFSF13B|CCL7|IL6ST|IL33|LOXL2|IL36RN|TNFSF11|IL6|IRAK4|LAMP2|PDCD6IP|IL10|TGFBI|DEFB4B|DICER1|IL22|CCL5|CCL16|TNFRSF9|CCL4 | 77 | 325|351|350|484|485|531|168|317|265|120|432|543|462|561|558|500|498|147|322|345|266|126|281|295|383|279|356|355|272|359|112|385|519|158|418|321|149|478|468|367|123|452|308|488|283|376|161|348|481|326|289|391|346|480|536|430|218|371|304|307|425|157|166|121|534|135|501|473|118 | 1.00E-12 |  | GO.0005615 | 0.930364361 | 0 |
| 70 | 16 | KEGG Pathways | | RIG-I-like receptor signaling pathway | 5.23E-10 | NFKB1|TRAF2|IL12A|MAPK10|ATG5|MAP3K7|SIKE1|TANK|MAPK8|MAP3K1|MAPK9|ATG12|IKBKB|TRAF6|TRAF3|IKBKE | 77 | 529|444|519|380|312|358|338|314|282|397|250|421|526|315|557|548 | 8.76E-11 |  | hsa04622 | 0.928149831 | 0 |
| 3284 | 135 | UniProt Keywords | | Disulfide bond | 5.50E-10 | CX3CL1|RELT|TNFRSF1A|TGFBR3|TGFB1|CCL1|CCL13|CXCL6|CRTAM|LOX|IL1RL1|TGFB3|TMX4|CCR7|LIF|ISLR|TXNDC17|LILRA1|PXDN|PDGFRA|CCL21|LOXL4|PDGFRB|LOXL1|MSR1|DGCR2|NOX4|IL7|PRDX5|LAMP3|ADAMTS17|IGSF6|SOD1|IL22RA1|ILDR2|ACKR3|TNFRSF10B|TNFSF4|TXNDC11|CSF1R|TNFRSF13C|KIR3DL3|CXCR5|TGFA|TRAT1|CXCL3|CXCL5|TNFRSF21|IL22RA2|TNFRSF11B|DEFB1|AVPR1A|TMX3|LTBP3|IL12A|CTLA4|IL13|ALCAM|IL7R|CXCL11|ICOS|IGDCC4|TNFRSF12A|CSF1|IFNLR1|IL25|CCR4|IGDCC3|LAMP1|BTLA|PDCD1|CXCL14|IL17F|IL21R|KIR2DL4|NCR3LG1|IFNAR2|CCRL2|CCR9|CCL20|TGFBR2|CCL28|CXCL9|TGFB2|IL20|IL6R|IGSF3|CMC4|IGSF1|IL1RAPL2|CXCR3|TLR4|IL2RG|TXNDC8|HLA-DQB1|TGFBR1|TNFSF13B|TNFRSF1B|CCL7|TXNDC5|IL6ST|TNFRSF19|LOXL2|IL36RN|IGSF11|CXCL12|PDCD1LG2|TXNRD2|CMKBR6|CSF2RB|IL6|LTBP1|IL1R1|CXCR4|IL18R1|TLR6|TMX1|CXCR6|HLA-DMB|HLA-B|LY6H|VSIG1|LAMP2|IL10|TGFBI|ACKR2|DEFB4B|IL22|TXNIP|TNFRSF11A|CCL5|MR1|CCL16|TNFRSF9|CCL4 | 77 | 325|532|351|447|350|484|485|531|151|168|336|265|301|243|432|477|140|344|543|516|500|242|506|498|147|405|364|266|208|377|267|546|281|211|237|247|410|383|156|153|224|442|306|279|527|356|355|513|272|359|112|222|122|441|519|183|418|286|176|321|167|457|340|149|341|478|502|261|360|449|537|468|367|217|515|313|452|134|456|308|332|488|283|376|161|348|270|271|132|252|230|342|563|238|109|368|326|276|289|459|391|256|480|536|130|181|373|476|431|524|218|154|274|450|287|305|390|435|553|114|357|378|304|425|157|173|166|534|415|163|135|540|501|473|118 | 1.38E-11 |  | KW-1015 | 0.925963731 | 0 |
| 206 | 26 | GO Process |  | regulation of mononuclear cell proliferation | 5.59E-10 | TGFB1|TNFSF9|ARG2|IL1B|IL7|TNFSF4|TNFRSF13C|TNFRSF21|IL12A|CTLA4|IL13|CSF1|PAWR|IKZF3|TGFBR2|TNFSF13B|IL6ST|NFATC2|PDCD1LG2|BCL2|BCL6|IL6|HLA-DMB|IL10|TRAF6|CCL5 | 77 | 350|120|518|345|266|383|224|513|519|183|418|149|396|392|332|326|391|115|373|475|310|218|553|425|315|135 | 2.26E-11 |  | GO.0032944 | 0.925258819 | 0 |
| 87 | 18 | GO Process |  | toll-like receptor signaling pathway | 5.99E-10 | IRAK2|TNIP1|MAPKAPK2|TAB2|MAP3K7|IRAK1|BCL10|TLR4|TAB3|TANK|MAP3K1|TLR6|IRAK4|TICAM2|IKBKB|TRAF6|TRAF3|IKBKE | 77 | 258|538|268|184|358|434|323|342|206|314|397|305|371|329|526|315|557|548 | 2.44E-11 |  | GO.0002224 | 0.922257318 | 0 |
| 338 | 33 | GO Process |  | regulation of inflammatory response | 6.44E-10 | CX3CL1|TNFRSF1A|NFKB1|IL1RL1|CCR7|IL1B|PDCD4|TNFSF4|IL17RB|RICTOR|IL22RA2|SOCS5|PPARD|TNIP1|SOCS3|SMAD3|IL17F|IL20|TLR4|TNFRSF1B|IL6ST|IL33|JAK2|TNFSF11|BCL6|PPARA|IL6|IL1R1|TLR6|IL10|TNFRSF11A|CCL5|TNFAIP3 | 77 | 325|351|529|336|243|345|311|383|497|302|272|226|227|538|246|241|367|161|342|276|391|346|309|430|310|491|218|274|305|425|163|135|164 | 2.63E-11 |  | GO.0050727 | 0.919111413 | 0 |
| 114 | 20 | GO Process |  | pattern recognition receptor signaling pathway | 6.44E-10 | IRAK2|TNIP1|MAPKAPK2|TAB2|MAP3K7|IRAK1|BCL10|TLR4|TAB3|TANK|MAP3K1|TLR6|IRAK4|TICAM2|IKBKB|TRAF6|TRAF3|IKBKE|MAP2K6|TNFAIP3 | 77 | 258|538|268|184|358|434|323|342|206|314|397|305|371|329|526|315|557|548|255|164 | 2.65E-11 |  | GO.0002221 | 0.919111413 | 0 |
| 64 | 16 | GO Process |  | Fc-epsilon receptor signaling pathway | 6.71E-10 | MAPK1|NFKB1|NFATC3|MAPK10|TAB2|MAP3K7|BCL10|TAB3|MAPK8|NFATC2|MAP3K1|MAPK9|ITK|LAT2|IKBKB|TRAF6 | 77 | 469|529|210|380|184|358|323|206|282|115|397|250|556|467|526|315 | 2.77E-11 |  | GO.0038095 | 0.917327748 | 0 |
| 245 | 28 | GO Process |  | negative regulation of cytokine production | 7.74E-10 | TGFB1|NFKB1|IL1RL1|TGFB3|ARG2|SMAD7|PDCD4|TNFSF4|TNFRSF21|IL12A|IL13|SOCS5|TGFB2|IL6R|TRAF3IP1|TLR4|IL33|IL36RN|PDCD1LG2|BCL6|IL6|TLR6|MAPKBP1|IL10|TICAM2|IKBKE|TNFRSF9|TNFAIP3 | 77 | 350|529|336|265|518|195|311|383|513|519|418|226|376|348|474|342|346|536|373|310|218|305|298|425|329|548|473|164 | 3.21E-11 |  | GO.0001818 | 0.911125904 | 0 |
| 1012 | 61 | Reactome Pathways | | Innate Immune System | 8.58E-10 | MAPK1|NFKB1|TRAF2|IRAK2|FAF2|MAP3K8|IL1B|ATF2|DOCK1|DEFB1|NFATC3|MAP2K1|BCL2L1|NKIRAS2|EEA1|SOCS1|LAMP1|MAP2K3|ATG7|MAPK10|ILF2|MAPKAPK2|TAB2|ATG5|MAP3K7|SIKE1|IRAK1|BCL10|TLR4|TNFRSF1B|TAB3|TXNDC5|TANK|MAPK8|NFATC2|MAP2K7|BCL2|MAP3K1|CMKBR6|TLR6|IRAK4|NKIRAS1|MAPK9|ITK|HLA-B|LAMP2|MAP2K4|TICAM2|LAT2|DEFB4B|ATG12|IKBKB|TRAF6|APAF1|TRAF3|TXNIP|IKBKE|MAP2K6|NOS1|TNFAIP3|MAP3K14 | 77 | 469|529|444|258|458|165|345|189|215|112|210|292|372|273|146|460|360|528|119|380|443|268|184|312|358|338|434|323|342|276|206|459|314|282|115|236|475|397|431|305|371|417|250|556|114|304|517|329|467|166|421|526|315|148|557|415|548|255|508|164|171 | 3.57E-11 |  | HSA-168249 | 0.906651271 | 0 |
| 426 | 37 | GO Process |  | regulation of response to biotic stimulus | 8.83E-10 | NFKB1|CRTAM|PIAS1|IRAK2|ARG2|IL1B|IL12A|TNIP1|IFNLR1|SOCS1|SOCS3|LAMP1|IFNAR2|MAPKAPK2|TAB2|MAP3K7|IRAK1|BCL10|TRAF3IP1|TLR4|TAB3|IRF4|JAK2|TANK|MAP3K1|TLR6|IRAK4|MAPKBP1|HLA-B|TICAM2|IKBKB|TRAF6|TRAF3|IKBKE|MAP2K6|CCL5|TNFAIP3 | 77 | 529|151|554|258|518|345|519|538|341|460|246|360|452|268|184|358|434|323|474|342|206|169|309|314|397|305|371|298|114|329|526|315|557|548|255|135|164 | 3.68E-11 |  | GO.0002831 | 0.90540393 | 0 |
| 103 | 19 | GO Process |  | myeloid leukocyte migration | 9.31E-10 | CX3CL1|CCL1|CCL13|CXCL6|CCR7|CCL21|IL1B|CXCL3|CCL20|TGFB2|IL6R|CCL7|TNFSF11|IL6|IRAK4|TNFRSF11A|CCL5|CCL16|CCL4 | 77 | 325|484|485|531|243|500|345|356|308|376|348|289|430|218|371|163|135|501|118 | 3.89E-11 |  | GO.0097529 | 0.903105032 | 0 |
| 162 | 23 | GO Process |  | regulation of response to cytokine stimulus | 9.48E-10 | TNFRSF1A|TRAF2|PIAS1|PXDN|IRAK2|IL7|CREBRF|CSF1|SOCS1|SOCS3|IFNAR2|IRAK1|TLR4|IL6ST|JAK2|IL36RN|IL6|IL1R1|TICAM2|IKBKB|HIF1A|CCL5|TNFAIP3 | 77 | 351|444|554|543|258|266|296|149|460|246|452|434|342|391|309|536|218|274|329|526|495|135|164 | 3.98E-11 |  | GO.0060759 | 0.902319166 | 0 |
| 45 | 14 | Reactome Pathways | | Interleukin-10 signaling | 9.77E-10 | TNFRSF1A|LIF|IL1A|IL1B|STAT3|IL12A|CSF1|CCL20|TNFRSF1B|IL6|IL1R1|IL10|CCL5|CCL4 | 77 | 351|432|322|345|334|519|149|308|276|218|274|425|135|118 | 4.20E-11 |  | HSA-6783783 | 0.901010544 | 0 |
| 147 | 22 | GO Process |  | regulation of adaptive immune response | 1.01E-09 | TGFB1|IL1RL1|TRAF2|SMAD7|IL1B|TNFSF4|TNFRSF13C|IL12A|SOCS5|IL7R|MAP3K7|TNFSF13B|IL6ST|IL33|BCL6|IL6|IL1R1|IL18R1|HLA-B|IL10|TRAF6|TNFAIP3 | 77 | 350|336|444|195|345|383|224|519|226|176|358|326|391|346|310|218|274|287|114|425|315|164 | 4.27E-11 |  | GO.0002819 | 0.899567863 | 0 |
| 27 | 12 | GO Function | | chemokine receptor activity | 1.03E-09 | CCR7|ACKR3|CXCR5|CCR4|GPR29|CCRL2|CCR9|CXCR3|CMKBR6|CXCR4|CXCR6|ACKR2 | 77 | 243|247|306|502|448|134|456|230|431|450|435|173 | 3.69E-11 |  | GO.0004950 | 0.898716278 | 0 |
| 708 | 50 | InterPro Domains | | Immunoglobulin-like fold | 1.07E-09 | NFKB1|CRTAM|EXOC2|IL1RL1|ISLR|LILRA1|PXDN|PDGFRA|PDGFRB|IGSF6|IL22RA1|CSF1R|KIR3DL3|IL22RA2|NFATC3|CTLA4|ALCAM|IL7R|IL1RAP|ICOS|IGDCC4|IFNLR1|IGDCC3|BTLA|PDCD1|IL21R|KIR2DL4|NCR3LG1|IFNAR2|RBPJ|AREL1|IL6R|IGSF3|IGSF1|IL1RAPL2|IL2RG|HLA-DQB1|IL6ST|IGSF11|NFATC2|PDCD1LG2|CSF2RB|IL1R1|IL18R1|NFATC4|HLA-DMB|HLA-B|VSIG1|NFAT5|MR1 | 77 | 529|151|339|336|477|344|543|516|506|546|211|153|442|272|210|183|286|176|199|167|457|341|261|449|537|217|515|313|452|438|437|348|270|132|252|563|109|391|130|115|373|524|274|287|249|553|114|378|393|540 | 1.44E-11 |  | IPR013783 | 0.897061622 | 0 |
| 512 | 41 | UniProt Keywords | | Immunity | 1.13E-09 | CRTAM|LILRA1|IL36G|IL36B|ARG2|MAP3K8|LAMP3|CSF1R|TNFRSF13C|TRAT1|TNFRSF21|CTLA4|ALCAM|CSF1|BTLA|PDCD1|IRF5|MAP3K5|THEMIS|ATG5|IRAK1|BCL10|IFIT3|IFIT2|TLR4|HLA-DQB1|TNFSF13B|JAK2|IL36RN|PDCD1LG2|BCL6|TLR6|IRAK4|ITK|HLA-DMB|HLA-B|TICAM2|LAT2|TRAF6|TRAF3|MR1 | 77 | 151|344|561|558|518|165|377|153|224|527|513|183|286|149|449|537|160|335|349|312|434|323|404|394|342|109|326|309|536|373|310|305|371|556|553|114|329|467|315|557|540 | 3.24E-11 |  | KW-0391 | 0.894692156 | 0 |
| 388 | 35 | GO Process |  | regulation of apoptotic signaling pathway | 1.15E-09 | CX3CL1|TNFSF10|TRAF2|BCL2L2|BCL2L10|IL1A|IL1B|IL7|SOD1|ACKR3|TNFRSF10B|DEPTOR|BCL2L1|CFLAR|PRKRA|TNFRSF12A|SMAD3|BAG5|MCL1|BCL10|YWHAB|TGFBR1|JAK2|BCL2L11|MAPK8|CXCL12|BCL2|NFATC4|MAPK9|BCLAF1|HIF1A|SOD2|APAF1|MOAP1|TNFAIP3 | 77 | 325|202|444|551|550|322|345|266|281|247|410|408|372|133|196|340|241|124|278|323|440|368|309|234|282|181|475|249|250|180|495|381|148|138|164 | 4.89E-11 |  | GO.2001233 | 0.893930216 | 0 |
| 250 | 28 | GO Process |  | positive regulation of DNA-binding transcription factor activity | 1.16E-09 | TGFB1|NFKB1|TRAF2|IRAK2|TRAF5|IL1B|ATF2|MAP3K13|CFLAR|IL1RAP|SMAD3|TAB2|MAP3K7|IRAK1|BCL10|TLR4|TAB3|JAK2|TNFSF11|IL6|IL18R1|TLR6|IRAK4|IL10|IKBKB|TRAF6|TNFRSF11A|ERC1 | 77 | 350|529|444|258|142|345|189|490|133|199|241|184|358|434|323|342|206|309|430|218|287|305|371|425|526|315|163|401 | 4.93E-11 |  | GO.0051091 | 0.893554201 | 0 |
| 135 | 21 | GO Process |  | regulation of adaptive immune response based on somatic recombination of immune receptors built from immunoglobulin superfamily domains | 1.46E-09 | TGFB1|IL1RL1|TRAF2|SMAD7|IL1B|TNFSF4|TNFRSF13C|IL12A|SOCS5|IL7R|MAP3K7|TNFSF13B|IL33|BCL6|IL6|IL1R1|IL18R1|HLA-B|IL10|TRAF6|TNFAIP3 | 77 | 350|336|444|195|345|383|224|519|226|176|358|326|346|310|218|274|287|114|425|315|164 | 6.25E-11 |  | GO.0002822 | 0.883564714 | 0 |
| 151 | 22 | GO Process |  | regulation of cytokine-mediated signaling pathway | 1.58E-09 | TNFRSF1A|TRAF2|PIAS1|PXDN|IRAK2|IL7|CREBRF|CSF1|SOCS1|SOCS3|IFNAR2|IRAK1|IL6ST|JAK2|IL36RN|IL6|IL1R1|TICAM2|IKBKB|HIF1A|CCL5|TNFAIP3 | 77 | 351|444|554|543|258|266|296|149|460|246|452|434|391|309|536|218|274|329|526|495|135|164 | 6.80E-11 |  | GO.0001959 | 0.880134291 | 0 |
| 332 | 32 | GO Process |  | immune response-activating signal transduction | 1.67E-09 | MAPK1|NFKB1|IRAK2|DOCK1|TRAT1|TNFRSF21|CTLA4|TNIP1|MAPKAPK2|TAB2|THEMIS|MAP3K7|IRAK1|BCL10|TLR4|HLA-DQB1|TAB3|TANK|NFATC2|BCL2|MAP3K1|TLR6|IRAK4|ITK|TICAM2|LAT2|IKBKB|TRAF6|TRAF3|IKBKE|MAP2K6|TNFAIP3 | 77 | 469|529|258|215|527|513|183|538|268|184|349|358|434|323|342|109|206|314|115|475|397|305|371|556|329|467|526|315|557|548|255|164 | 7.21E-11 |  | GO.0002757 | 0.877728353 | 0 |
| 69 | 16 | GO Process |  | regulation of cytokine production involved in immune response | 1.68E-09 | TGFB1|TGFB3|TRAF2|SMAD7|IL1B|TNFSF4|TGFB2|ATG5|MAP3K7|BCL10|BCL6|IL6|IL1R1|IL18R1|IL10|TRAF6 | 77 | 350|265|444|195|345|383|376|312|358|323|310|218|274|287|425|315 | 7.28E-11 |  | GO.0002718 | 0.877469072 | 0 |
| 181 | 23 | KEGG Pathways | | Herpes simplex infection | 1.69E-09 | TNFRSF1A|NFKB1|TRAF2|TRAF5|IL1B|IL12A|SOCS3|IFNAR2|MAPK10|TAB2|MAP3K7|JAK2|MAPK8|IRF9|IL6|MAPK9|HLA-DMB|HLA-B|IKBKB|TRAF6|TRAF3|IKBKE|CCL5 | 77 | 351|529|444|142|345|519|246|452|380|184|358|309|282|492|218|250|553|114|526|315|557|548|135 | 2.91E-10 |  | hsa05168 | 0.87721133 | 0 |
| 137 | 21 | GO Process |  | cytokine production | 1.83E-09 | CCR7|IL1B|TNFSF4|LTBP3|IL12A|IL1RAP|NFATC2IP|PAWR|IL25|IL17F|RBPJ|BCL10|CXCR3|TLR4|NFATC2|TLR6|IRAK4|ITK|HIF1A|NFAT5|MR1 | 77 | 243|345|383|441|519|199|347|396|478|367|438|323|230|342|115|305|371|556|495|393|540 | 7.98E-11 |  | GO.0001816 | 0.873754891 | 0 |
| 22 | 11 | Reactome Pathways | | JNK (c-Jun kinases) phosphorylation and activation mediated by activated human TAK1 | 2.05E-09 | IRAK2|MAPK10|TAB2|MAP3K7|IRAK1|TAB3|MAPK8|MAP2K7|MAPK9|MAP2K4|TRAF6 | 77 | 258|380|184|358|434|206|282|236|250|517|315 | 9.13E-11 |  | HSA-450321 | 0.868824614 | 0 |
| 1826 | 88 | GO Process |  | positive regulation of gene expression | 2.17E-09 | TNFRSF1A|MAPK1|TGFB1|NFKB1|BCL9|TGFB3|RHOQ|RASL11A|LIF|PIAS1|SMAD7|SMAD2|RIMS2|IL1A|IL1B|ATF2|IL11|STAT3|LAMP3|IRF8|SMAD6|CREBRF|NFATC3|MAP2K1|IL7R|PPARD|TNIP1|NFATC2IP|CSF1|PAWR|IL25|IKZF1|SMAD3|BCL9L|IL17F|BCL11A|SMAD4|IKZF3|MAP2K3|RBPJ|BCL11B|IRF5|MAP3K5|ILF2|NOS1AP|TGFB2|IRF6|MAPKAPK2|IRAK1|BCL10|BAG2|AGO3|AGO1|CXCR3|TLR4|TGFBR1|HIF3A|NFIB|IRF4|IL33|PDCD10|MAPK8|NFATC2|TNFSF11|PPARA|IL6|MAP3K2|NFATC4|TLR6|MAPK9|NCOA2|ILF3|IL10|IKZF2|SMAD1|IKBKB|TRAF6|BCLAF1|HIF1A|SMAD5|NKRF|TET2|NFAT5|LITAF|IER2|PIAS2|CCL5|NOS1 | 77 | 351|469|350|529|175|265|552|205|432|554|195|200|331|322|345|189|126|334|377|398|369|296|210|292|176|227|538|347|149|396|478|220|241|125|367|507|470|392|528|438|128|160|335|443|212|376|487|268|434|323|116|520|239|230|342|368|510|170|169|346|316|282|115|430|491|218|374|249|305|250|455|110|425|486|193|526|315|180|495|201|291|294|393|395|354|284|135|508 | 9.51E-11 |  | GO.0010628 | 0.866354027 | 0 |
| 3559 | 140 | GO Process |  | regulation of biological quality | 2.42E-09 | NOX3|TGFBR3|MAPK1|TGFB1|CCL1|CCL13|NFKB1|RHOQ|TMX4|CCR7|ATG14|LIF|PDGFRA|HILPDA|DOCK10|TBRG4|CCL21|FDX1|ARG2|MSR1|SMAD7|SMAD2|RIMS2|NOX4|IL1A|IL1B|IL7|ATF2|IL11|STAT3|PRDX5|CDK6|HPS5|SOD1|ILDR2|DOCK11|DOCK5|DOCK1|TXNDC11|DEPTOR|CSF1R|BAG4|TNFRSF13C|FAM19A4|RICTOR|TNFRSF11B|AVPR1A|HPS6|TMX3|BCL2L1|MAEA|IL13|IL7R|CXCL11|PPARD|IL1RAP|RHOJ|PRKRA|TNFRSF12A|CSF1|IKZF1|CCR4|SMAD3|LAMP1|BCL11A|BAG5|SMAD4|LAMTOR4|MAP4K4|GPR29|PEX2|CCR9|TXNDC15|CPLX2|CCL28|CXCL9|NOS1AP|MAPKAPK2|PEX19|MCL1|ATG5|BAG3|PEX11B|BCL10|BAG2|EXOSC1|YWHAB|CXCR3|TLR4|TXNDC8|TNFSF13B|TNFRSF1B|DOCK9|CCL7|TXNDC5|BNIP3L|JAK2|TXNRD3|EXOSC8|MAP3K4|PDCD10|BCL2L11|FDX1L|ULK2|MAPK8|CXCL12|MAP2K7|BCL2|TNFSF11|TXNRD2|CMKBR6|BCL6|PPARA|IL6|LTBP1|CXCR4|NFATC4|TLR6|TMX1|VSIG1|DOCK4|LAMP2|TBRG1|PDCD6IP|IL10|SMAD1|CDKN2AIP|TRAF6|HIF1A|SMAD5|SOD2|MAPKAPK5|MOAP1|IKBKE|TNFRSF11A|CCL5|NOS1|PANO|SOCS2|TNFAIP3 | 77 | 203|447|469|350|484|485|529|552|301|243|150|432|516|462|505|496|500|353|518|147|195|200|331|364|322|345|266|189|126|334|208|365|366|281|237|422|406|215|156|408|153|409|224|545|302|359|222|209|122|372|207|418|176|321|227|199|375|196|340|149|220|502|241|360|507|124|470|188|254|448|269|456|494|216|488|283|212|268|521|278|312|318|343|323|116|137|440|230|342|238|326|276|300|289|459|499|309|263|297|466|316|234|512|214|282|181|236|475|430|476|431|310|491|218|154|450|249|305|390|378|544|304|445|307|425|193|324|315|495|201|381|370|138|548|163|135|508|172|419|164 | 1.06E-10 |  | GO.0065008 | 0.861618463 | 0 |
| 205 | 25 | GO Process |  | regulation of lymphocyte proliferation | 2.43E-09 | TGFB1|TNFSF9|ARG2|IL1B|IL7|TNFSF4|TNFRSF13C|TNFRSF21|IL12A|CTLA4|IL13|PAWR|IKZF3|TGFBR2|TNFSF13B|IL6ST|NFATC2|PDCD1LG2|BCL2|BCL6|IL6|HLA-DMB|IL10|TRAF6|CCL5 | 77 | 350|120|518|345|266|383|224|513|519|183|418|396|392|332|326|391|115|373|475|310|218|553|425|315|135 | 1.07E-10 |  | GO.0050670 | 0.861439373 | 0 |
| 653 | 46 | GO Process |  | regulation of multi-organism process | 2.45E-09 | NFKB1|CRTAM|EXOC2|PIAS1|IRAK2|ARG2|SMURF2|IL1B|LAMP3|IRF8|IL12A|TNIP1|IFNLR1|SOCS1|SOCS3|LAMP1|IFNAR2|MAPKAPK2|TAB2|MAP3K7|IRAK1|BCL10|TRAF3IP1|TLR4|TAB3|IRF4|JAK2|TANK|BCL2L11|BCL2|MAP3K1|TLR6|IRAK4|MAPKBP1|HLA-B|ILF3|IL10|TICAM2|IKBKB|TRAF6|TRAF3|IKBKE|MAP2K6|CCL5|TNFAIP3|CCL4 | 77 | 529|151|339|554|258|518|362|345|377|398|519|538|341|460|246|360|452|268|184|358|434|323|474|342|206|169|309|314|234|475|397|305|371|298|114|110|425|329|526|315|557|548|255|135|164|118 | 1.08E-10 |  | GO.0043900 | 0.861083392 | 0 |
| 403 | 35 | GO Process |  | regulation of DNA-binding transcription factor activity | 2.86E-09 | MAPK1|TGFB1|NFKB1|TRAF2|MAP3K10|IRAK2|TRAF5|SMAD7|IL1B|ATF2|MAP3K13|TNFSF4|CFLAR|IL1RAP|SMAD3|TAB2|MAP3K7|IRAK1|BCL10|TLR4|TAB3|JAK2|TNFSF11|IL6|IL18R1|TLR6|IRAK4|IL10|IKBKB|TRAF6|TRAF3|TNFRSF11A|PIAS2|ERC1|TNFAIP3 | 77 | 469|350|529|444|489|258|142|195|345|189|490|383|133|199|241|184|358|434|323|342|206|309|430|218|287|305|371|425|526|315|557|163|284|401|164 | 1.27E-10 |  | GO.0051090 | 0.854363397 | 0 |
| 914 | 56 | GO Process |  | cell motility | 3.11E-09 | CX3CL1|TGFBR3|TGFB1|CCL1|CCL13|TNFAIP1|CXCL6|CCR7|PDGFRA|CCL21|PDGFRB|IL1B|TNFRSF10B|DOCK1|CXCR5|PEX13|CXCL3|CXCL5|MAP2K1|IL16|IL12A|CXCL11|PPARD|TNFRSF12A|CCR4|CXCL14|GPR29|BCL11B|CCL20|CCL28|CXCL9|TGFB2|IL6R|CXCR3|TGFBR1|CCL7|JAK2|LOXL2|LYST|PDCD10|CXCL12|NFATC2|TNFSF11|CMKBR6|IL6|CXCR4|IRAK4|DOCK4|IL10|HIF1A|TNFRSF11A|IER2|CCL5|CCL16|SOCS7|CCL4 | 77 | 325|447|350|484|485|280|531|243|516|500|506|345|410|215|306|152|356|355|292|385|519|321|227|340|502|468|448|128|308|488|283|376|348|230|368|289|309|480|555|316|181|115|430|431|218|450|371|544|425|495|163|354|135|501|186|118 | 1.40E-10 |  | GO.0048870 | 0.850723961 | 0 |
| 384 | 34 | Reactome Pathways | | Cellular responses to stress | 3.21E-09 | MAPK1|NFKB1|CDKN1B|NOX4|IL1A|STAT3|CDK6|SOD1|BAG4|HIF1AN|BAG5|MAP4K4|MAP2K3|MAP3K5|MAPK10|GPX6|MAPKAPK2|BAG3|BAG2|AGO3|AGO1|AGO4|HIF3A|MAPK8|MAP2K7|TXNRD2|IL6|MAPK9|TNIK|MAP2K4|HIF1A|SOD2|MAPKAPK5|MAP2K6 | 77 | 469|529|285|364|322|334|365|281|409|228|124|254|528|335|380|388|268|318|116|520|239|240|510|282|236|476|218|250|535|517|495|381|370|255 | 1.48E-10 |  | HSA-2262752 | 0.849349497 | 0 |
| 155 | 21 | KEGG Pathways | | Necroptosis | 3.45E-09 | TNFRSF1A|TNFSF10|TRAF2|TRAF5|IL1A|IL1B|STAT3|TNFRSF10B|AIFM1|CFLAR|IFNAR2|MAPK10|TLR4|IL33|JAK2|MAPK8|IRF9|BCL2|MAPK9|TICAM2|TNFAIP3 | 77 | 351|202|444|142|322|345|334|410|482|133|452|380|342|346|309|282|492|475|250|329|164 | 6.12E-10 |  | hsa04217 | 0.84621809 | 0 |
| 156 | 21 | KEGG Pathways | | Cellular senescence | 3.72E-09 | MAPK1|TGFB1|NFKB1|TGFB3|SMAD2|IL1A|CDK6|NFATC3|MAP2K1|SMAD3|KIR2DL4|MAP2K3|TGFBR2|TGFB2|MAPKAPK2|TGFBR1|NFATC2|IL6|NFATC4|HLA-B|MAP2K6 | 77 | 469|350|529|265|200|322|365|210|292|241|515|528|332|376|268|368|115|218|249|114|255 | 6.80E-10 |  | hsa04218 | 0.842945706 | 0 |
| 193 | 24 | GO Process |  | homeostasis of number of cells | 3.92E-09 | TGFBR3|TGFB1|CCR7|DOCK10|IL7|PRDX5|SOD1|ILDR2|DOCK11|TNFRSF13C|MAEA|IL7R|CSF1|IKZF1|CCR4|TNFSF13B|JAK2|BCL2L11|BCL2|BCL6|IL6|HIF1A|SMAD5|TNFAIP3 | 77 | 447|350|243|505|266|208|281|237|422|224|207|176|149|220|502|326|309|234|475|310|218|495|201|164 | 1.77E-10 |  | GO.0048872 | 0.840671393 | 0 |
| 894 | 55 | GO Process |  | leukocyte activation | 3.96E-09 | CX3CL1|MAPK1|TGFB1|CXCL6|NFKB1|CCR7|DOCK10|CCL21|FAF2|IL7|IL11|STAT3|DOCK11|TNFSF4|CXCR5|IL12A|IL13|IL7R|CSF1|IKZF1|SMAD3|LAMP1|IL21R|GPR29|IKZF3|RBPJ|ATG7|BCL11B|CCR9|TGFBR2|CPLX2|ILF2|THEMIS|ATG5|PI4K2A|TLR4|TNFRSF1B|TXNDC5|IRF4|IL33|BCL2|CMKBR6|BCL6|IL6|IL18R1|TLR6|ITK|HLA-B|LAMP2|IL10|TICAM2|LAT2|TRAF6|APAF1|CCL5 | 77 | 325|469|350|531|529|243|505|500|458|266|126|334|422|383|306|519|418|176|149|220|241|360|217|448|392|438|119|128|456|332|216|443|349|312|399|342|276|459|169|346|475|431|310|218|287|305|556|114|304|425|329|467|315|148|135 | 1.80E-10 |  | GO.0045321 | 0.840230481 | 0 |
| 161 | 22 | Reactome Pathways | | Cellular Senescence | 4.33E-09 | MAPK1|NFKB1|CDKN1B|IL1A|STAT3|CDK6|MAP4K4|MAP2K3|MAP3K5|MAPK10|MAPKAPK2|AGO3|AGO1|AGO4|MAPK8|MAP2K7|IL6|MAPK9|TNIK|MAP2K4|MAPKAPK5|MAP2K6 | 77 | 469|529|285|322|334|365|254|528|335|380|268|520|239|240|282|236|218|250|535|517|370|255 | 2.06E-10 |  | HSA-2559583 | 0.83635121 | 0 |
| 1435 | 74 | GO Process |  | positive regulation of transcription, DNA-templated | 5.11E-09 | TNFRSF1A|MAPK1|TGFB1|NFKB1|BCL9|TGFB3|RHOQ|RASL11A|LIF|PIAS1|SMAD7|SMAD2|IL1A|IL1B|ATF2|IL11|STAT3|IRF8|SMAD6|CREBRF|NFATC3|MAP2K1|PPARD|TNIP1|NFATC2IP|IL25|IKZF1|SMAD3|BCL9L|IL17F|BCL11A|SMAD4|IKZF3|MAP2K3|RBPJ|BCL11B|IRF5|MAP3K5|ILF2|TGFB2|IRF6|IRAK1|BCL10|AGO1|CXCR3|TLR4|TGFBR1|HIF3A|NFIB|IRF4|IL33|NFATC2|TNFSF11|PPARA|IL6|MAP3K2|NFATC4|NCOA2|ILF3|IL10|IKZF2|SMAD1|IKBKB|TRAF6|BCLAF1|HIF1A|SMAD5|NKRF|TET2|NFAT5|LITAF|IER2|PIAS2|NOS1 | 77 | 351|469|350|529|175|265|552|205|432|554|195|200|322|345|189|126|334|398|369|296|210|292|227|538|347|478|220|241|125|367|507|470|392|528|438|128|160|335|443|376|487|434|323|239|230|342|368|510|170|169|346|115|430|491|218|374|249|455|110|425|486|193|526|315|180|495|201|291|294|393|395|354|284|508 | 2.33E-10 |  | GO.0045893 | 0.82915791 | 0 |
| 526 | 40 | GO Process |  | hemopoiesis | 5.24E-09 | TGFBR3|TGFB1|CCR7|PDGFRA|DOCK10|IL7|IL11|STAT3|IRF8|DOCK11|CSF1R|MAEA|IL7R|CSF1|IL25|IKZF1|GPR29|RBPJ|BCL11B|CCR9|TGFBR2|TGFB2|THEMIS|ATG5|IRF4|JAK2|CIAPIN1|BCL2|TNFSF11|CMKBR6|BCL6|IL6|IL18R1|ITK|IL10|TRAF6|HIF1A|SMAD5|TET2|TNFRSF11A | 77 | 447|350|243|516|505|266|126|334|398|422|153|207|176|149|478|220|448|438|128|456|332|376|349|312|169|309|472|475|430|431|310|218|287|556|425|315|495|201|294|163 | 2.40E-10 |  | GO.0030097 | 0.828066871 | 0 |
| 223 | 27 | GO Component | | external side of plasma membrane | 5.45E-09 | TGFBR3|IL1RL1|CCR7|PDGFRA|TNFRSF13C|CXCR5|CTLA4|IL13|ALCAM|IL7R|ICOS|CCR4|LAMP1|PDCD1|GPR29|TGFBR2|MAP3K5|CXCL9|CXCR3|TLR4|IL2RG|IL6ST|CXCL12|CMKBR6|IL1R1|TNFRSF11A|TNFRSF9 | 77 | 447|336|243|516|224|306|183|418|286|176|167|502|360|537|448|332|335|283|230|342|563|391|181|431|274|163|473 | 2.20E-11 |  | GO.0009897 | 0.82636035 | 0 |
| 85 | 16 | KEGG Pathways | | Colorectal cancer | 5.65E-09 | MAPK1|TGFB1|TGFB3|SMAD2|TGFA|MAP2K1|SMAD3|SMAD4|TGFBR2|MAPK10|TGFB2|TGFBR1|BCL2L11|MAPK8|BCL2|MAPK9 | 77 | 469|350|265|200|279|292|241|470|332|380|376|368|234|282|475|250 | 1.06E-09 |  | hsa05210 | 0.824795155 | 0 |
| 99 | 17 | KEGG Pathways | | T cell receptor signaling pathway | 5.74E-09 | MAPK1|NFKB1|MAP3K8|NFATC3|MAP2K1|CTLA4|ICOS|PDCD1|MAP3K7|BCL10|NFATC2|MAP2K7|MAPK9|ITK|IL10|IKBKB|MAP3K14 | 77 | 469|529|165|210|292|183|167|537|358|323|115|236|250|556|425|526|171 | 1.11E-09 |  | hsa04660 | 0.824108811 | 0 |
| 30 | 11 | KEGG Pathways | | Autophagy - other | 6.39E-09 | ATG10|ATG3|ATG4D|ATG7|ATG2B|ATG5|ATG16L1|ULK2|ATG9A|ATG12|ATG13 | 77 | 155|113|213|119|389|312|223|214|257|421|559 | 1.27E-09 |  | hsa04136 | 0.819449914 | 0 |
| 961 | 57 | GO Process |  | regulation of anatomical structure morphogenesis | 6.53E-09 | CX3CL1|TNFRSF1A|TGFB1|CCL13|RHOQ|LIF|RIMS2|SMURF2|IL1A|IL1B|ATF2|STAT3|DOCK5|DOCK1|CSF1R|TNFRSF13C|TNFRSF11B|HIF1AN|MAP2K1|CFLAR|RHOJ|TNFRSF12A|CSF1|BCL9L|IL17F|BCL11A|SMAD4|RBPJ|TGFBR2|SMURF1|CXCL9|MAP3K3|TGFB2|AGO1|AGO4|CXCR3|TGIF2|TGFBR1|TNFSF13B|TNFRSF1B|BCOR|CCL7|NFIB|PDCD10|ULK2|CXCL12|NFATC2|BCL2|BCL6|IL6|NFATC4|TNIK|IL10|SMAD1|HIF1A|ECSCR|TNFAIP3 | 77 | 325|351|350|485|552|432|331|362|322|345|189|334|406|215|153|224|359|228|292|133|375|340|149|125|367|507|470|438|332|464|283|299|376|239|240|230|259|368|326|276|541|289|170|316|214|181|115|475|310|218|249|535|425|193|495|253|164 | 3.01E-10 |  | GO.0022603 | 0.818508682 | 0 |
| 313 | 30 | GO Process |  | leukocyte differentiation | 7.04E-09 | TGFB1|CCR7|DOCK10|IL7|IL11|STAT3|DOCK11|CSF1R|IL7R|CSF1|IL25|IKZF1|GPR29|RBPJ|BCL11B|CCR9|TGFBR2|THEMIS|ATG5|IRF4|BCL2|TNFSF11|CMKBR6|BCL6|IL6|IL18R1|ITK|IL10|TRAF6|TNFRSF11A | 77 | 350|243|505|266|126|334|422|153|176|149|478|220|448|438|128|456|332|349|312|169|475|430|431|310|218|287|556|425|315|163 | 3.25E-10 |  | GO.0002521 | 0.815242734 | 0 |
| 92 | 17 | Reactome Pathways | | Oxidative Stress Induced Senescence | 8.36E-09 | MAPK1|CDK6|MAP4K4|MAP2K3|MAP3K5|MAPK10|MAPKAPK2|AGO3|AGO1|AGO4|MAPK8|MAP2K7|MAPK9|TNIK|MAP2K4|MAPKAPK5|MAP2K6 | 77 | 469|365|254|528|335|380|268|520|239|240|282|236|250|535|517|370|255 | 4.09E-10 |  | HSA-2559580 | 0.807779372 | 0 |
| 1024 | 59 | GO Process |  | cell activation | 8.79E-09 | CX3CL1|MAPK1|TGFB1|CXCL6|NFKB1|CCR7|PDGFRA|DOCK10|CCL21|FAF2|IL7|IL11|STAT3|DOCK11|TNFSF4|CXCR5|IL12A|IL13|IL7R|C8orf4|CSF1|IKZF1|SMAD3|LAMP1|IL21R|SMAD4|GPR29|IKZF3|RBPJ|ATG7|BCL11B|CCR9|TGFBR2|CPLX2|ILF2|THEMIS|ATG5|PI4K2A|TLR4|TGFBR1|TNFRSF1B|TXNDC5|IRF4|IL33|BCL2|CMKBR6|BCL6|IL6|IL18R1|TLR6|ITK|HLA-B|LAMP2|IL10|TICAM2|LAT2|TRAF6|APAF1|CCL5 | 77 | 325|469|350|531|529|243|516|505|500|458|266|126|334|422|383|306|519|418|176|293|149|220|241|360|217|470|448|392|438|119|128|456|332|216|443|349|312|399|342|368|276|459|169|346|475|431|310|218|287|305|556|114|304|425|329|467|315|148|135 | 4.08E-10 |  | GO.0001775 | 0.805601112 | 0 |
| 35 | 12 | GO Process |  | regulation of pri-miRNA transcription by RNA polymerase II | 8.96E-09 | TGFB1|STAT3|SMAD6|NFATC3|PPARD|SMAD3|TGFB2|NFIB|PPARA|NFATC4|SMAD1|HIF1A | 77 | 350|334|369|210|227|241|376|170|491|249|193|495 | 4.18E-10 |  | GO.1902893 | 0.804769199 | 0 |
| 1754 | 84 | GO Function | | identical protein binding | 9.70E-09 | TNFRSF1A|MAPK1|TGFB1|TNFAIP1|NFKB1|CRTAM|TGFB3|TNFSF10|TRAF2|BCL2L2|MAP3K10|IRAK2|PDGFRA|BCL2L10|TRAF5|SMAD2|TRAF4|SMURF2|STAT3|MAP3K13|BNIP2|SOD1|CSF1R|SMAD6|CXCL5|DEFB1|HIF1AN|BCL2L1|ALCAM|MAP3K11|OXSR1|TNIP1|EEA1|PRKRA|CSF1|SMAD3|IL17F|BCL11A|C1QTNF6|SMAD4|IKZF3|ATG7|IRF5|FXR1|MAP3K5|KIAA1804|TGFB2|IL6R|MCL1|PEX11B|MAP3K7|IRAK1|BCL10|BAG2|IFIT3|YWHAB|TLR4|BNIP3L|IL6ST|JAK2|EXOSC8|ATG16L1|PDCD10|PRC1|BCL2|MAPK4|GATSL3|BCL6|TLR6|PDCD6IP|IKZF2|SMAD1|IKBKB|TRAF6|RASSF3|SOD2|APAF1|MAP3K12|MAP3K9|MAP2K6|CCL5|TNFAIP3|CCL4|GATSL2 | 77 | 351|469|350|280|529|151|265|202|444|551|489|258|516|550|142|200|194|362|334|490|187|281|153|369|355|112|228|372|286|439|504|538|146|196|149|241|367|507|123|470|392|119|160|245|335|190|376|348|278|343|358|434|323|116|404|440|342|499|391|309|297|223|316|511|475|231|288|310|305|307|486|193|526|315|221|381|148|426|290|255|135|164|118|402 | 3.77E-10 |  | GO.0042802 | 0.801322827 | 0 |
| 3603 | 139 | GO Process |  | cellular protein metabolic process | 1.03E-08 | MAP4K5|TGFBR3|MAPK1|TGFB1|TNFAIP1|NFKB1|CDKN1B|LOX|NKTR|TRAF2|LIF|PIAS1|MAP3K10|IRAK2|PDGFRA|TBRG4|PDGFRB|MAPK6|LOXL1|FAF2|MMD|SMAD7|SMAD2|SMURF2|MAP3K8|IL1B|MAP4K3|ATF2|MAP3K13|CDK6|ATG10|ATG3|CSF1R|PEX13|IL17RD|RICTOR|HIF1AN|TMX3|MAP2K1|MAEA|SOCS5|MAP3K11|ATG4D|OXSR1|TNIP1|PRKRA|AATK|NFATC2IP|CSF1|SOCS1|SOCS3|SMAD3|BCL11A|SMAD4|MAP4K4|MAP2K3|MAP3K15|ATG7|AREL1|PEX2|TGFBR2|MAP3K5|MAPK10|SMURF1|MAP3K3|KIAA1804|TGFB2|MAPKAPK2|TAB2|ATG5|CACUL1|MAP3K7|IRAK1|BCL10|YWHAB|TLR4|IL2RG|TGFBR1|ZAK|MAP3K19|HIF3A|BCOR|TAB3|BNIP3L|IRF4|IL33|JAK2|TNFRSF19|LOXL2|ATG16L1|MAP3K4|AKTIP|SOCS4|ULK2|MAPK8|SOCS6|MAP2K7|BCL2|TNFSF11|MAP3K1|MAPK4|CSF2RB|IL6|LTBP1|MAP3K2|IRAK4|PEX5|EIF4E3|MAPK9|ITK|TNIK|ILF3|LAMP2|MAP2K4|PDCD6IP|TGFBI|ATG12|SMAD1|IKBKB|TRAF6|HIF1A|SMAD5|TET2|MAP3K12|MAPKAPK5|JKAMP|MAP3K9|TRAF3|IKBKE|PIAS2|ERC1|MAP2K6|CCL5|NOS1|SOCS2|TNFAIP3|SOCS7|PEX12|MAP3K14 | 77 | 451|447|469|350|280|529|285|168|117|444|432|554|489|258|516|496|506|235|498|458|427|195|200|362|165|345|141|189|490|365|155|113|153|152|136|302|228|122|292|207|226|439|213|504|538|196|560|347|149|460|246|241|507|470|254|528|384|119|437|269|332|335|380|464|299|190|376|268|184|312|522|358|434|323|440|342|563|368|264|352|510|541|206|499|169|346|309|256|480|223|466|424|523|214|282|244|236|475|430|397|231|524|218|154|374|371|514|403|250|556|535|110|304|517|307|157|421|193|526|315|495|201|294|426|370|382|290|557|548|284|401|255|135|508|419|164|186|229|171 | 4.81E-10 |  | GO.0044267 | 0.798716278 | 0 |
| 153 | 21 | GO Process |  | regulation of receptor signaling pathway via STAT | 1.05E-08 | TNFRSF1A|LIF|STAT3|CSF1R|IL22RA2|IL12A|IL13|SOCS5|IL7R|SOCS1|SOCS3|IL20|IL6R|IL6ST|JAK2|SOCS4|SOCS6|IL6|CCL5|SOCS2|SOCS7 | 77 | 351|432|334|153|272|519|418|226|176|460|246|161|348|391|309|523|244|218|135|419|186 | 4.95E-10 |  | GO.1904892 | 0.79788107 | 0 |
| 361 | 32 | GO Process |  | regulation of innate immune response | 1.08E-08 | NFKB1|CRTAM|PIAS1|IRAK2|IL12A|TNIP1|SOCS1|SOCS3|LAMP1|IFNAR2|MAPKAPK2|TAB2|MAP3K7|IRAK1|BCL10|TLR4|TAB3|IRF4|JAK2|TANK|MAP3K1|TLR6|IRAK4|HLA-B|TICAM2|IKBKB|TRAF6|TRAF3|IKBKE|MAP2K6|CCL5|TNFAIP3 | 77 | 529|151|554|258|519|538|460|246|360|452|268|184|358|434|323|342|206|169|309|314|397|305|371|114|329|526|315|557|548|255|135|164 | 5.08E-10 |  | GO.0045088 | 0.796657624 | 0 |
| 436 | 37 | GO Component | | side of membrane | 1.12E-08 | TGFBR3|IL1RL1|CCR7|TRAF2|PDGFRA|TRAF5|TNFRSF13C|CXCR5|CTLA4|IL13|ALCAM|IL7R|ICOS|CCR4|LAMP1|PDCD1|GPR29|TGFBR2|MAP3K5|CXCL9|CXCR3|TLR4|IL2RG|HLA-DQB1|IL6ST|JAK2|CXCL12|CMKBR6|IL1R1|ITK|HLA-B|IKBKB|TRAF6|TRAF3|LITAF|TNFRSF11A|TNFRSF9 | 77 | 447|336|243|444|516|142|224|306|183|418|286|176|167|502|360|537|448|332|335|283|230|342|563|109|391|309|181|431|274|556|114|526|315|557|395|163|473 | 6.80E-11 |  | GO.0098552 | 0.795078198 | 0 |
| 4958 | 179 | GO Component | | cytosol | 1.12E-08 | MAPK1|NFKB1|CDKN1B|EXOC2|NKTR|RHOQ|ATG14|TRAF2|LIF|TXNDC17|BCL2L2|EXOC4|XPO4|IRAK2|HILPDA|DOCK10|IER3|BCL2L10|TRAF5|MAPK6|SMAD7|SMAD2|SMURF2|MAP3K8|IL1A|IL1B|ITPKC|STAT3|PECR|PRDX5|CDK6|HPS5|DOCK3|ETNK1|BNIP2|IRF8|SOD1|DOCK11|DOCK5|ENDOD1|PDCD4|DOCK1|ATG10|ATG3|AIFM1|BAG4|SMAD6|SNIP1|RICTOR|IL22RA2|HIF1AN|HPS6|NFATC3|MAP2K1|BCL2L1|IL16|SOCS5|ATG4D|OXSR1|CFLAR|RHOJ|TNIP1|EEA1|PRKRA|C8orf4|TNFAIP8L3|SOCS1|SOCS3|SMAD3|LAMP1|BAG5|MIOS|SMAD4|NECAP1|IKZF3|MAP2K3|ATG7|AREL1|IRF5|FXR1|TGFBR2|MAP3K5|MAPK10|CPLX2|SMURF1|MAP3K3|NOS1AP|IRF6|TAGAP|MAPKAPK2|TAB2|PEX3|PEX19|MCL1|ATG5|BAG3|MAP3K7|SIKE1|IRAK1|BCL10|PI4K2A|BAG2|EXOSC1|IFIT3|IFIT2|YWHAB|AGO3|AGO1|AGO4|IL2RG|ZAK|DOCK9|HIF3A|TAB3|BNIP3L|IRF4|JAK2|EXOSC8|ATG16L1|TANK|PDCD10|BCL2L11|BCL2L15|PRC1|AKTIP|MAPK8|NFATC2|IRF9|BLOC1S5|SOCS6|MAP2K7|BCL2|MAP3K1|MAPK4|TXNRD2|GATSL3|MAP3K2|NFATC4|IRAK4|PEX5|EIF4E3|MAPK9|ITK|TNIK|DOCK4|MAP2K4|LMLN|PDCD6IP|ACKR2|PEX5L|ATG12|SMAD1|IKBKB|AMBRA1|ATG13|TRAF6|DICER1|HIF1A|SMAD5|RASSF3|APAF1|MAP3K12|MAPKAPK5|MOAP1|TRAF3|NFAT5|LITAF|TXNIP|IKBKE|TNFRSF11A|ERC1|MAP2K6|NOS1|ECSCR|SOCS2|TNFAIP3|SOCS7|MAP3K14|GATSL2 | 77 | 469|529|285|339|117|552|150|444|432|140|551|159|386|258|462|505|461|550|142|235|195|200|362|165|322|345|420|334|436|208|365|366|225|191|187|398|281|422|406|204|311|215|155|113|482|409|369|337|302|272|228|209|210|292|372|385|226|213|504|133|375|538|146|196|293|248|460|246|241|360|124|525|470|542|392|528|119|437|160|245|332|335|380|216|464|299|212|487|139|268|184|423|521|278|312|318|358|338|434|323|399|116|137|404|394|440|520|239|240|563|264|300|510|206|499|169|309|297|223|314|316|234|320|511|424|282|115|492|198|244|236|475|397|231|476|288|374|249|371|514|403|250|556|535|544|517|333|307|173|328|421|193|526|131|559|315|121|495|201|221|148|426|370|138|557|393|395|415|548|163|401|255|508|253|419|164|186|171|402 | 6.81E-11 |  | GO.0005829 | 0.795078198 | 0 |
| 280 | 28 | GO Process |  | adaptive immune response | 1.13E-08 | TGFB1|CRTAM|LILRA1|ARG2|STAT3|LAMP3|TNFRSF13C|TRAT1|TNFRSF21|IL12A|CTLA4|ALCAM|BTLA|GPR29|THEMIS|BCL10|TLR4|HLA-DQB1|IRF4|JAK2|CMKBR6|BCL6|IL6|IL18R1|ITK|LAT2|TRAF6|TNFRSF11A | 77 | 350|151|344|518|334|377|224|527|513|519|183|286|449|448|349|323|342|109|169|309|431|310|218|287|556|467|315|163 | 5.34E-10 |  | GO.0002250 | 0.794692156 | 0 |
| 4337 | 159 | GO Process |  | regulation of biosynthetic process | 1.19E-08 | TNFRSF1A|TGFBR3|MAPK1|TGFB1|TNFAIP1|NFKB1|CDKN1B|BCL9|SOGA1|TGFB3|RHOQ|RASL11A|CCR7|TRAF2|LIF|PIAS1|MAP3K10|IRAK2|CCL21|TRAF5|ARG2|PDGFRB|SMAD7|SMAD2|SMURF2|NOX4|IL1A|IL1B|ATF2|IL11|STAT3|MAP3K13|PRDX5|CCAR1|IRF8|SOD1|IL9|PDCD4|TNFSF4|GATAD1|SMAD6|TNFRSF13C|TGFA|SNIP1|CREBRF|HIF1AN|AVPR1A|NFATC3|MAP2K1|MAEA|IL16|PPARD|CFLAR|IL1RAP|TNIP1|C8orf4|NFATC2IP|TGIF1|PAWR|IL25|PIWIL3|IKZF1|SMAD3|BCL9L|IL17F|BCL11A|SMAD4|IKZF3|MAP2K3|RBPJ|ATG7|PEX2|BCL11B|IRF5|FXR1|CCL20|MAP3K5|SMURF1|ILF2|NOS1AP|IRF2BP2|TGFB2|IRF6|MAPKAPK2|TAB2|HIVEP2|IL6R|GATAD2B|IKZF5|BAG3|MAP3K7|IRAK1BP1|IRAK1|BCL10|IGSF1|LCOR|YWHAB|AGO3|AGO1|AGO4|CXCR3|TGIF2|TLR4|TGFBR1|HIF3A|BCOR|TAB3|NFIB|IRF4|IL33|JAK2|LOXL2|MAP3K4|NFATC2|IRF9|MAP2K7|BCL2|TNFSF11|BCL6|PPARA|IL6|IL18R1|MAP3K2|NFATC4|TLR6|IRAK4|EIF4E3|NCOA2|ILF3|MAP2K4|IL10|IKZF2|SMAD1|IKBKB|TRAF6|BCLAF1|DICER1|BCORL1|HIF1A|SMAD5|NKRF|TET2|BCL7A|SOD2|MAPKAPK5|MAP3K9|TRAF3|NFAT5|LITAF|TXNIP|TNFRSF11A|IER2|PIAS2|ERC1|MAP2K6|CCL5|NOS1|TNFAIP3|CCL4 | 77 | 351|447|469|350|280|529|285|175|317|265|552|205|243|444|432|554|489|258|500|142|518|506|195|200|362|364|322|345|189|126|334|490|208|111|398|281|295|311|383|530|369|224|279|337|296|228|222|210|292|207|385|227|133|199|538|293|347|143|396|478|182|220|241|125|367|507|470|392|528|438|119|269|128|160|245|308|335|464|443|212|178|376|487|268|184|433|348|414|412|318|358|400|434|323|132|233|440|520|239|240|230|259|342|368|510|541|206|170|169|346|309|480|466|115|492|236|475|430|310|491|218|287|374|249|305|371|403|455|110|517|425|486|193|526|315|180|121|387|495|201|291|294|303|381|370|290|557|393|395|415|163|354|284|401|255|135|508|164|118 | 5.67E-10 |  | GO.0009889 | 0.792445304 | 0 |
| 1770 | 84 | GO Process |  | positive regulation of nucleobase-containing compound metabolic process | 1.21E-08 | TNFRSF1A|MAPK1|TGFB1|TNFAIP1|NFKB1|BCL9|TGFB3|RHOQ|RASL11A|LIF|PIAS1|PDGFRB|SMAD7|SMAD2|NOX4|IL1A|IL1B|ATF2|IL11|STAT3|CCAR1|IRF8|TNFSF4|SMAD6|CREBRF|NFATC3|MAP2K1|PPARD|TNIP1|NFATC2IP|IL25|IKZF1|SMAD3|BCL9L|IL17F|BCL11A|SMAD4|IKZF3|MAP2K3|RBPJ|BCL11B|IRF5|MAP3K5|ILF2|TGFB2|IRF6|IRAK1|BCL10|AGO1|CXCR3|TLR4|TGFBR1|TNFRSF1B|HIF3A|NFIB|IRF4|IL33|MAP3K4|NFATC2|MAP2K7|TNFSF11|PPARA|IL6|MAP3K2|NFATC4|NCOA2|ILF3|MAP2K4|IL10|IKZF2|SMAD1|IKBKB|TRAF6|BCLAF1|HIF1A|SMAD5|NKRF|TET2|MAPKAPK5|NFAT5|LITAF|IER2|PIAS2|NOS1 | 77 | 351|469|350|280|529|175|265|552|205|432|554|506|195|200|364|322|345|189|126|334|111|398|383|369|296|210|292|227|538|347|478|220|241|125|367|507|470|392|528|438|128|160|335|443|376|487|434|323|239|230|342|368|276|510|170|169|346|466|115|236|430|491|218|374|249|455|110|517|425|486|193|526|315|180|495|201|291|294|370|393|395|354|284|508 | 5.75E-10 |  | GO.0045935 | 0.791721463 | 0 |
| 4533 | 164 | GO Process |  | regulation of gene expression | 1.42E-08 | TNFRSF1A|TGFBR3|MAPK1|TGFB1|NFKB1|CDKN1B|BCL9|TGFB3|RHOQ|RASL11A|ATG14|TRAF2|LIF|PIAS1|MAP3K10|IRAK2|PDGFRA|TBRG4|TRAF5|MAPK6|MSR1|SMAD7|SMAD2|RIMS2|SMURF2|IL1A|IL1B|IL7|ATF2|IL11|STAT3|MAP3K13|PRDX5|LAMP3|CDK6|CCAR1|IRF8|PDCD4|TNFSF4|GATAD1|SMAD6|TGFA|SNIP1|RICTOR|CREBRF|HIF1AN|NFATC3|MAP2K1|IL16|IL7R|PPARD|CFLAR|IL1RAP|TNIP1|PRKRA|C8orf4|NFATC2IP|CSF1|TGIF1|PAWR|IL25|PIWIL3|IKZF1|SMAD3|BCL9L|IL17F|BCL11A|SMAD4|IKZF3|MAP2K3|RBPJ|PEX2|BCL11B|IRF5|FXR1|TGFBR2|MAP3K5|MAPK10|SMURF1|ILF2|NOS1AP|IRF2BP2|TGFB2|IRF6|MAPKAPK2|TAB2|HIVEP2|GATAD2B|IKZF5|BAG3|MAP3K7|IRAK1BP1|IRAK1|BCL10|BAG2|EXOSC1|IGSF1|LCOR|YWHAB|AGO3|AGO1|AGO4|CXCR3|TGIF2|TLR4|TGFBR1|TNFRSF1B|HIF3A|BCOR|TAB3|NFIB|IRF4|IL33|JAK2|LOXL2|EXOSC8|MAP3K4|PDCD10|MAPK8|NFATC2|IRF9|BCL2|TNFSF11|MAPK4|BCL6|PPARA|IL6|IL18R1|MAP3K2|NFATC4|TLR6|IRAK4|EIF4E3|MAPK9|NCOA2|ILF3|IL10|IKZF2|SMAD1|IKBKB|TRAF6|BCLAF1|DICER1|BCORL1|HIF1A|SMAD5|NKRF|TET2|BCL7A|SOD2|MAPKAPK5|MAP3K9|TRAF3|NFAT5|LITAF|TXNIP|TNFRSF11A|IER2|PIAS2|ERC1|MAP2K6|CCL5|NOS1|TNFAIP3 | 77 | 351|447|469|350|529|285|175|265|552|205|150|444|432|554|489|258|516|496|142|235|147|195|200|331|362|322|345|266|189|126|334|490|208|377|365|111|398|311|383|530|369|279|337|302|296|228|210|292|385|176|227|133|199|538|196|293|347|149|143|396|478|182|220|241|125|367|507|470|392|528|438|269|128|160|245|332|335|380|464|443|212|178|376|487|268|184|433|414|412|318|358|400|434|323|116|137|132|233|440|520|239|240|230|259|342|368|276|510|541|206|170|169|346|309|480|297|466|316|282|115|492|475|430|231|310|491|218|287|374|249|305|371|403|250|455|110|425|486|193|526|315|180|121|387|495|201|291|294|303|381|370|290|557|393|395|415|163|354|284|401|255|135|508|164 | 6.79E-10 |  | GO.0010468 | 0.784771166 | 0 |
| 70 | 15 | GO Process |  | regulation of tyrosine phosphorylation of STAT protein | 1.54E-08 | TNFRSF1A|LIF|STAT3|CSF1R|IL22RA2|IL12A|IL13|SOCS1|SOCS3|IL20|IL6R|IL6ST|JAK2|IL6|CCL5 | 77 | 351|432|334|153|272|519|418|460|246|161|348|391|309|218|135 | 7.43E-10 |  | GO.0042509 | 0.781247928 | 0 |
| 305 | 29 | GO Process |  | cellular response to external stimulus | 1.59E-08 | TNFRSF1A|MAPK1|TGFB1|NFKB1|ATG14|WDR59|IL1B|SOD1|TNFRSF10B|AIFM1|WDR24|AVPR1A|IL13|MIOS|ATG7|MAP3K5|ATG5|BAG3|BCL10|TLR4|MAPK8|BCL2|MAP3K1|GATSL3|MAP3K2|LAMP2|MAP2K4|AMBRA1|MAP3K14 | 77 | 351|469|350|529|150|127|345|281|410|482|260|222|418|525|119|335|312|318|323|342|282|475|397|288|374|304|517|131|171 | 7.67E-10 |  | GO.0071496 | 0.779860288 | 0 |
| 126 | 19 | GO Process |  | regulation of smooth muscle cell proliferation | 1.69E-08 | CDKN1B|TGFB3|PDGFRB|PDCD4|IL12A|IL13|PPARD|TGFBR2|MAP3K5|IL6R|IRAK1|JAK2|IL6|IRAK4|IL10|TRAF6|SOD2|CCL5|TNFAIP3 | 77 | 285|265|506|311|519|418|227|332|335|348|434|309|218|371|425|315|381|135|164 | 8.19E-10 |  | GO.0048660 | 0.77721133 | 0 |
| 44 | 12 | KEGG Pathways | | Intestinal immune network for IgA production | 1.83E-08 | TGFB1|TNFRSF13C|ICOS|CCR9|CCL28|TNFSF13B|CXCL12|IL6|CXCR4|HLA-DMB|IL10|MAP3K14 | 77 | 350|224|167|456|488|326|181|218|450|553|425|171 | 3.71E-09 |  | hsa04672 | 0.773754891 | 0 |
| 690 | 47 | GO Component | | cell surface | 1.90E-08 | CX3CL1|TNFRSF1A|TGFBR3|TGFB1|IL1RL1|TGFB3|CCR7|PDGFRA|HILPDA|PDGFRB|ACKR3|TNFRSF10B|TNFSF4|CSF1R|IL17RB|TNFRSF13C|CXCR5|TGFA|TMX3|IL12A|CTLA4|IL13|ALCAM|IL7R|ICOS|TNFRSF12A|CCR4|LAMP1|PDCD1|GPR29|CCR9|TGFBR2|MAP3K5|CXCL9|IL6R|CXCR3|TLR4|IL2RG|TGFBR1|IL6ST|CXCL12|CMKBR6|IL1R1|CXCR4|HLA-B|TNFRSF11A|TNFRSF9 | 77 | 325|351|447|350|336|265|243|516|462|506|247|410|383|153|497|224|306|279|122|519|183|418|286|176|167|340|502|360|537|448|456|332|335|283|348|230|342|563|368|391|181|431|274|450|114|163|473 | 1.92E-10 |  | GO.0009986 | 0.77212464 | 0 |
| 112 | 18 | GO Process |  | regulation of interleukin-6 production | 1.90E-08 | IL1A|IL1B|TNFSF4|SOCS5|IL1RAP|IL17F|MAPKAPK2|IL6R|TLR4|IL33|IL36RN|IL6|TLR6|HLA-B|IL10|TICAM2|TRAF6|TNFAIP3 | 77 | 322|345|383|226|199|367|268|348|342|346|536|218|305|114|425|329|315|164 | 9.23E-10 |  | GO.0032675 | 0.77212464 | 0 |
| 4266 | 156 | GO Process |  | regulation of cellular biosynthetic process | 2.27E-08 | TNFRSF1A|TGFBR3|MAPK1|TGFB1|TNFAIP1|NFKB1|CDKN1B|BCL9|SOGA1|TGFB3|RHOQ|RASL11A|CCR7|TRAF2|LIF|PIAS1|MAP3K10|IRAK2|CCL21|TRAF5|PDGFRB|SMAD7|SMAD2|SMURF2|NOX4|IL1A|IL1B|ATF2|IL11|STAT3|MAP3K13|PRDX5|CCAR1|IRF8|IL9|PDCD4|TNFSF4|GATAD1|SMAD6|TNFRSF13C|TGFA|SNIP1|CREBRF|HIF1AN|AVPR1A|NFATC3|MAP2K1|MAEA|IL16|PPARD|CFLAR|IL1RAP|TNIP1|C8orf4|NFATC2IP|TGIF1|PAWR|IL25|PIWIL3|IKZF1|SMAD3|BCL9L|IL17F|BCL11A|SMAD4|IKZF3|MAP2K3|RBPJ|ATG7|PEX2|BCL11B|IRF5|FXR1|CCL20|MAP3K5|SMURF1|ILF2|NOS1AP|IRF2BP2|TGFB2|IRF6|MAPKAPK2|TAB2|HIVEP2|GATAD2B|IKZF5|BAG3|MAP3K7|IRAK1BP1|IRAK1|BCL10|IGSF1|LCOR|YWHAB|AGO3|AGO1|AGO4|CXCR3|TGIF2|TLR4|TGFBR1|HIF3A|BCOR|TAB3|NFIB|IRF4|IL33|JAK2|LOXL2|MAP3K4|NFATC2|IRF9|MAP2K7|BCL2|TNFSF11|BCL6|PPARA|IL6|IL18R1|MAP3K2|NFATC4|TLR6|IRAK4|EIF4E3|NCOA2|ILF3|MAP2K4|IL10|IKZF2|SMAD1|IKBKB|TRAF6|BCLAF1|DICER1|BCORL1|HIF1A|SMAD5|NKRF|TET2|BCL7A|SOD2|MAPKAPK5|MAP3K9|TRAF3|NFAT5|LITAF|TXNIP|TNFRSF11A|IER2|PIAS2|ERC1|MAP2K6|CCL5|NOS1|TNFAIP3|CCL4 | 77 | 351|447|469|350|280|529|285|175|317|265|552|205|243|444|432|554|489|258|500|142|506|195|200|362|364|322|345|189|126|334|490|208|111|398|295|311|383|530|369|224|279|337|296|228|222|210|292|207|385|227|133|199|538|293|347|143|396|478|182|220|241|125|367|507|470|392|528|438|119|269|128|160|245|308|335|464|443|212|178|376|487|268|184|433|414|412|318|358|400|434|323|132|233|440|520|239|240|230|259|342|368|510|541|206|170|169|346|309|480|466|115|492|236|475|430|310|491|218|287|374|249|305|371|403|455|110|517|425|486|193|526|315|180|121|387|495|201|291|294|303|381|370|290|557|393|395|415|163|354|284|401|255|135|508|164|118 | 1.11E-09 |  | GO.0031326 | 0.764397414 | 0 |
| 1520 | 75 | GO Process |  | positive regulation of nucleic acid-templated transcription | 2.34E-08 | TNFRSF1A|MAPK1|TGFB1|NFKB1|BCL9|TGFB3|RHOQ|RASL11A|LIF|PIAS1|SMAD7|SMAD2|IL1A|IL1B|ATF2|IL11|STAT3|CCAR1|IRF8|SMAD6|CREBRF|NFATC3|MAP2K1|PPARD|TNIP1|NFATC2IP|IL25|IKZF1|SMAD3|BCL9L|IL17F|BCL11A|SMAD4|IKZF3|MAP2K3|RBPJ|BCL11B|IRF5|MAP3K5|ILF2|TGFB2|IRF6|IRAK1|BCL10|AGO1|CXCR3|TLR4|TGFBR1|HIF3A|NFIB|IRF4|IL33|NFATC2|TNFSF11|PPARA|IL6|MAP3K2|NFATC4|NCOA2|ILF3|IL10|IKZF2|SMAD1|IKBKB|TRAF6|BCLAF1|HIF1A|SMAD5|NKRF|TET2|NFAT5|LITAF|IER2|PIAS2|NOS1 | 77 | 351|469|350|529|175|265|552|205|432|554|195|200|322|345|189|126|334|111|398|369|296|210|292|227|538|347|478|220|241|125|367|507|470|392|528|438|128|160|335|443|376|487|434|323|239|230|342|368|510|170|169|346|115|430|491|218|374|249|455|110|425|486|193|526|315|180|495|201|291|294|393|395|354|284|508 | 1.15E-09 |  | GO.1903508 | 0.763078414 | 0 |
| 311 | 29 | GO Function | | ubiquitin protein ligase binding | 2.87E-08 | TRAF2|PIAS1|TRAF5|FAF2|SMAD7|SMAD2|TRAF4|BAG4|SMAD6|SMAD3|BAG5|CACUL1|BCL10|BAG2|TNFRSF1B|TANK|AKTIP|BCL2|CXCR4|AMBRA1|TRAF6|HIF1A|SMAD5|JKAMP|MOAP1|TRAF3|TXNIP|IKBKE|PIAS2 | 77 | 444|554|142|458|195|200|194|409|369|241|124|522|323|116|276|314|424|475|450|131|315|495|201|382|138|557|415|548|284 | 1.16E-09 |  | GO.0031625 | 0.75421181 | 0 |
| 131 | 19 | GO Process |  | positive regulation of mononuclear cell proliferation | 2.97E-08 | TNFSF9|IL1B|IL7|TNFSF4|TNFRSF13C|IL12A|IL13|CSF1|TGFBR2|TNFSF13B|IL6ST|NFATC2|PDCD1LG2|BCL2|BCL6|IL6|HLA-DMB|TRAF6|CCL5 | 77 | 120|345|266|383|224|519|418|149|332|326|391|115|373|475|310|218|553|315|135 | 1.47E-09 |  | GO.0032946 | 0.752724355 | 0 |
| 358 | 31 | GO Process |  | lymphocyte activation | 3.24E-08 | TGFB1|CCR7|DOCK10|CCL21|IL7|IL11|STAT3|DOCK11|TNFSF4|CXCR5|IL12A|IL7R|IKZF1|SMAD3|IL21R|GPR29|IKZF3|RBPJ|BCL11B|CCR9|THEMIS|ATG5|IRF4|BCL2|CMKBR6|BCL6|IL6|IL18R1|ITK|IL10|LAT2 | 77 | 350|243|505|500|266|126|334|422|383|306|519|176|220|241|217|448|392|438|128|456|349|312|169|475|431|310|218|287|556|425|467 | 1.61E-09 |  | GO.0046649 | 0.748945499 | 0 |
| 4143 | 152 | GO Process |  | regulation of macromolecule biosynthetic process | 3.34E-08 | TNFRSF1A|TGFBR3|MAPK1|TGFB1|TNFAIP1|NFKB1|CDKN1B|BCL9|TGFB3|RHOQ|RASL11A|CCR7|TRAF2|LIF|PIAS1|MAP3K10|IRAK2|CCL21|TRAF5|PDGFRB|SMAD7|SMAD2|SMURF2|NOX4|IL1A|IL1B|ATF2|IL11|STAT3|MAP3K13|PRDX5|CCAR1|IRF8|IL9|PDCD4|TNFSF4|GATAD1|SMAD6|TNFRSF13C|TGFA|SNIP1|CREBRF|HIF1AN|NFATC3|MAP2K1|IL16|PPARD|CFLAR|IL1RAP|TNIP1|C8orf4|NFATC2IP|TGIF1|PAWR|IL25|PIWIL3|IKZF1|SMAD3|BCL9L|IL17F|BCL11A|SMAD4|IKZF3|MAP2K3|RBPJ|ATG7|PEX2|BCL11B|IRF5|FXR1|CCL20|MAP3K5|SMURF1|ILF2|IRF2BP2|TGFB2|IRF6|MAPKAPK2|TAB2|HIVEP2|GATAD2B|IKZF5|BAG3|MAP3K7|IRAK1BP1|IRAK1|BCL10|IGSF1|LCOR|YWHAB|AGO3|AGO1|AGO4|CXCR3|TGIF2|TLR4|TGFBR1|HIF3A|BCOR|TAB3|NFIB|IRF4|IL33|JAK2|LOXL2|MAP3K4|NFATC2|IRF9|MAP2K7|BCL2|TNFSF11|BCL6|PPARA|IL6|IL18R1|MAP3K2|NFATC4|TLR6|IRAK4|EIF4E3|NCOA2|ILF3|MAP2K4|IL10|IKZF2|SMAD1|IKBKB|TRAF6|BCLAF1|DICER1|BCORL1|HIF1A|SMAD5|NKRF|TET2|BCL7A|SOD2|MAPKAPK5|MAP3K9|TRAF3|NFAT5|LITAF|TXNIP|TNFRSF11A|IER2|PIAS2|ERC1|MAP2K6|CCL5|NOS1|TNFAIP3|CCL4 | 77 | 351|447|469|350|280|529|285|175|265|552|205|243|444|432|554|489|258|500|142|506|195|200|362|364|322|345|189|126|334|490|208|111|398|295|311|383|530|369|224|279|337|296|228|210|292|385|227|133|199|538|293|347|143|396|478|182|220|241|125|367|507|470|392|528|438|119|269|128|160|245|308|335|464|443|178|376|487|268|184|433|414|412|318|358|400|434|323|132|233|440|520|239|240|230|259|342|368|510|541|206|170|169|346|309|480|466|115|492|236|475|430|310|491|218|287|374|249|305|371|403|455|110|517|425|486|193|526|315|180|121|387|495|201|291|294|303|381|370|290|557|393|395|415|163|354|284|401|255|135|508|164|118 | 1.67E-09 |  | GO.0010556 | 0.747625353 | 0 |
| 15 | 9 | GO Function | | MAP kinase kinase activity | 3.44E-08 | MAPK1|MAP2K1|MAP2K3|MAPK10|MAP2K7|MAP2K4|MAPKAPK5|MAP3K9|MAP2K6 | 77 | 469|292|528|380|236|517|370|290|255 | 1.44E-09 |  | GO.0004708 | 0.746344156 | 0 |
| 41 | 12 | GO Process |  | monocyte chemotaxis | 3.77E-08 | CX3CL1|CCL13|CCL21|CCL20|IL6R|CCL7|TNFSF11|IL6|TNFRSF11A|CCL5|CCL16|CCL4 | 77 | 325|485|500|308|348|289|430|218|163|135|501|118 | 1.88E-09 |  | GO.0002548 | 0.742365865 | 0 |
| 151 | 20 | GO Process |  | regulation of receptor signaling pathway via JAK-STAT | 4.44E-08 | TNFRSF1A|LIF|STAT3|CSF1R|IL22RA2|IL12A|IL13|SOCS5|SOCS1|SOCS3|IL20|IL6R|IL6ST|JAK2|SOCS4|SOCS6|IL6|CCL5|SOCS2|SOCS7 | 77 | 351|432|334|153|272|519|418|226|460|246|161|348|391|309|523|244|218|135|419|186 | 2.24E-09 |  | GO.0046425 | 0.735261703 | 0 |
| 116 | 17 | KEGG Pathways | | Neurotrophin signaling pathway | 4.50E-08 | MAPK1|NFKB1|IRAK2|MAP2K1|MAP3K5|MAPK10|MAP3K3|MAPKAPK2|IRAK1|MAPK8|MAP2K7|BCL2|MAP3K1|IRAK4|MAPK9|IKBKB|TRAF6 | 77 | 469|529|258|292|335|380|299|268|434|282|236|475|397|371|250|526|315 | 9.37E-09 |  | hsa04722 | 0.734678749 | 0 |
| 168 | 21 | GO Process |  | innate immune response-activating signal transduction | 4.50E-08 | NFKB1|IRAK2|TNIP1|MAPKAPK2|TAB2|MAP3K7|IRAK1|BCL10|TLR4|TAB3|TANK|MAP3K1|TLR6|IRAK4|TICAM2|IKBKB|TRAF6|TRAF3|IKBKE|MAP2K6|TNFAIP3 | 77 | 529|258|538|268|184|358|434|323|342|206|314|397|305|371|329|526|315|557|548|255|164 | 2.28E-09 |  | GO.0002758 | 0.734678749 | 0 |
| 264 | 27 | SMART Domains | | Serine/Threonine protein kinases, catalytic domain | 4.66E-08 | MAP4K5|MAPK1|MAPK6|MAP4K3|CDK6|MAP2K1|OXSR1|MAP4K4|MAP2K3|MAP3K15|MAP3K5|MAPK10|MAP3K3|MAPKAPK2|MAP3K19|MAP3K4|ULK2|MAPK8|MAP2K7|MAP3K1|MAPK4|MAP3K2|MAPK9|TNIK|MAP2K4|MAPKAPK5|MAP2K6 | 77 | 451|469|235|141|365|292|504|254|528|384|335|380|299|268|352|466|214|282|236|397|231|374|250|535|517|370|255 | 6.96E-10 |  | SM00220 | 0.733161408 | 0 |
| 672 | 44 | GO Process |  | regulation of secretion by cell | 4.67E-08 | TNFRSF1A|TGFB1|CRTAM|IL1RL1|TGFB3|CCR7|TRAF2|LIF|ARG2|RIMS2|IL1A|IL1B|IL11|TNFSF4|CSF1R|IL17RB|TNFRSF21|AVPR1A|IL13|PPARD|IL1RAP|LAMP1|IL17F|SMAD4|MAP4K4|CPLX2|TGFB2|ATG5|TLR4|TNFRSF1B|IL33|JAK2|IL36RN|CXCL12|TNFSF11|IL6|TLR6|MAPKBP1|PDCD6IP|IL10|TRAF6|HIF1A|CCL5|TNFRSF9 | 77 | 351|350|151|336|265|243|444|432|518|331|322|345|126|383|153|497|513|222|418|227|199|360|367|470|254|216|376|312|342|276|346|309|536|181|430|218|305|298|307|425|315|495|135|473 | 2.38E-09 |  | GO.1903530 | 0.733068312 | 0 |
| 1104 | 60 | GO Process |  | positive regulation of transcription by RNA polymerase II | 4.67E-08 | TNFRSF1A|TGFB1|NFKB1|BCL9|TGFB3|RHOQ|LIF|SMAD7|SMAD2|IL1A|IL1B|ATF2|IL11|STAT3|IRF8|SMAD6|CREBRF|NFATC3|TNIP1|NFATC2IP|IL25|IKZF1|SMAD3|BCL9L|IL17F|BCL11A|SMAD4|IKZF3|RBPJ|BCL11B|IRF5|TGFB2|IRF6|AGO1|CXCR3|TLR4|HIF3A|NFIB|IRF4|IL33|NFATC2|TNFSF11|PPARA|IL6|NFATC4|NCOA2|IL10|IKZF2|SMAD1|IKBKB|TRAF6|HIF1A|SMAD5|NKRF|TET2|NFAT5|LITAF|IER2|PIAS2|NOS1 | 77 | 351|350|529|175|265|552|432|195|200|322|345|189|126|334|398|369|296|210|538|347|478|220|241|125|367|507|470|392|438|128|160|376|487|239|230|342|510|170|169|346|115|430|491|218|249|455|425|486|193|526|315|495|201|291|294|393|395|354|284|508 | 2.37E-09 |  | GO.0045944 | 0.733068312 | 0 |
| 120 | 18 | GO Process |  | positive regulation of inflammatory response | 4.82E-08 | CX3CL1|TNFRSF1A|IL1RL1|CCR7|IL1B|PDCD4|TNFSF4|IL17RB|TNIP1|IL17F|TLR4|IL6ST|IL33|JAK2|TNFSF11|IL6|TLR6|TNFRSF11A | 77 | 325|351|336|243|345|311|383|497|538|367|342|391|346|309|430|218|305|163 | 2.47E-09 |  | GO.0050729 | 0.731695296 | 0 |
| 4726 | 167 | GO Process |  | multicellular organism development | 4.91E-08 | CX3CL1|RELT|NOX3|TNFRSF1A|TGFBR3|MAPK1|TGFB1|CDKN1B|LOX|BCL9|TGFB3|TNFSF10|CCR7|LIF|BCL2L2|DOCK7|EXOC4|PDGFRA|DOCK10|ARG2|PDGFRB|MAPK6|LOXL1|MMD|SMAD7|SMAD2|RIMS2|TRAF4|DGCR2|NOX4|IL1B|IL7|ATF2|IL11|STAT3|CDK6|IRF8|SOD1|ILDR2|ACKR3|DOCK11|TNFRSF10B|PDCD4|CSF1R|AIFM1|SMAD6|CXCR5|PEX13|RICTOR|TNFRSF21|TNFRSF11B|AVPR1A|MAP2K1|BCL2L1|MAEA|ALCAM|IL7R|PPARD|CFLAR|IL1RAP|RHOJ|PRKRA|ICOS|PRX|TNFRSF12A|CSF1|TGIF1|IL25|PIWIL3|SOCS3|IKZF1|CCR4|SMAD3|PDCD1|BCL9L|CXCL14|IL17F|BCL11A|BAG5|SMAD4|MAP4K4|GPR29|RBPJ|BCL11B|FXR1|CCR9|TGFBR2|MAPK10|CPLX2|SMURF1|MAP3K3|TGFB2|IRF6|MAPKAPK2|TAB2|HIVEP2|THEMIS|IL6R|MCL1|ATG5|BAG3|IGSF3|TNFRSF6B|BCL10|IL1RAPL2|AGO4|TRAF3IP1|CXCR3|TGIF2|TXNDC8|TGFBR1|ZAK|TNFRSF1B|HIF3A|BCOR|NFIB|IRF4|IL6ST|IL33|JAK2|TNFRSF19|TXNRD3|LOXL2|MAP3K4|PDCD10|BCL2L11|CIAPIN1|ULK2|MAPK8|CXCL12|BLOC1S5|BCL2|TNFSF11|CMKBR6|BCL6|PPARA|IL6|LTBP1|CXCR4|IL18R1|NFATC4|MAPK9|ITK|LY6H|TNIK|MAP2K4|IL10|TGFBI|ACKR2|SMAD1|AMBRA1|TRAF6|DICER1|HIF1A|SMAD5|TET2|APAF1|TXNIP|TNFRSF11A|IER2|ERC1|NOS1|TNFRSF9|ECSCR|SOCS2|TNFAIP3|SOCS7 | 77 | 325|532|203|351|447|469|350|285|168|175|265|202|243|432|551|413|159|516|505|518|506|235|498|427|195|200|331|194|405|364|345|266|189|126|334|365|398|281|237|247|422|410|311|153|482|369|306|152|302|513|359|222|292|372|207|286|176|227|133|199|375|196|167|319|340|149|143|478|182|246|220|502|241|537|125|468|367|507|124|470|254|448|438|128|245|456|332|380|216|464|299|376|487|268|184|433|349|348|278|312|318|270|481|323|252|240|474|230|259|238|368|264|276|510|541|170|169|391|346|309|256|263|480|466|316|234|472|214|282|181|198|475|430|431|310|491|218|154|450|287|249|250|556|357|535|517|425|157|173|193|131|315|121|495|201|294|148|415|163|354|401|508|473|253|419|164|186 | 2.52E-09 |  | GO.0007275 | 0.730891851 | 0 |
| 676 | 44 | GO Process |  | innate immune response | 5.46E-08 | CX3CL1|TGFB1|CCL1|CCL13|NFKB1|IL36G|IL36B|CCL21|ARG2|IRF8|CSF1R|DEFB1|IL1RAP|CSF1|IFNLR1|IFNAR2|IRF5|CCL20|MAP3K5|IRF6|IRAK1|BCL10|IFIT3|IFIT2|TLR4|HLA-DQB1|CCL7|IRF4|JAK2|LYST|IL36RN|IRF9|TLR6|IRAK4|ITK|HLA-B|TICAM2|IKBKB|TRAF3|IKBKE|CCL5|MR1|CCL16|CCL4 | 77 | 325|350|484|485|529|561|558|500|518|398|153|112|199|149|341|452|160|308|335|487|434|323|404|394|342|109|289|169|309|555|536|492|305|371|556|114|329|526|557|548|135|540|501|118 | 2.82E-09 |  | GO.0045087 | 0.726280736 | 0 |
| 1814 | 84 | UniProt Keywords | | Secreted | 5.48E-08 | CX3CL1|TNFRSF1A|TGFBR3|TGFB1|CCL1|CCL13|CXCL6|LOX|IL1RL1|SOGA1|TGFB3|TNFSF10|LIF|ISLR|PXDN|HILPDA|IL36G|IL36B|CCL21|LOXL4|LOXL1|IL1A|IL1B|IL7|IL11|ADAMTS17|IL9|ENDOD1|IL17RB|LY6K|TGFA|FAM19A4|CXCL3|CXCL5|IL22RA2|TNFRSF11B|DEFB1|LTBP3|IL16|IL12A|IL13|ALCAM|IL7R|CXCL11|ICOS|CSF1|IL25|C1QTNF8|CXCL14|IL17F|C1QTNF6|IFNAR2|CCL20|CCL28|GPX6|CXCL9|TGFB2|IL20|IL6R|IGSF1|TNFSF13B|TNFRSF1B|CCL7|IL6ST|IL33|IL17RE|LOXL2|IL36RN|CXCL12|PDCD1LG2|TNFSF11|FAM19A5|IL6|LTBP1|IL1R1|PDCD6IP|IL10|TGFBI|DEFB4B|IL22|CCL5|MR1|CCL16|CCL4 | 77 | 325|351|447|350|484|485|531|168|336|317|265|202|432|477|543|462|561|558|500|242|498|322|345|266|126|267|295|204|497|503|279|545|356|355|272|359|112|441|385|519|418|286|176|321|167|149|478|471|468|367|123|452|308|488|388|283|376|161|348|132|326|276|289|391|346|465|480|536|181|373|430|327|218|154|274|307|425|157|166|534|135|540|501|118 | 1.78E-09 |  | KW-0964 | 0.726121944 | 0 |
| 728 | 46 | GO Process |  | regulation of secretion | 5.58E-08 | TNFRSF1A|TGFB1|CRTAM|IL1RL1|TGFB3|CCR7|TRAF2|LIF|ARG2|RIMS2|IL1A|IL1B|IL11|TNFSF4|CSF1R|IL17RB|TNFRSF21|AVPR1A|IL13|PPARD|IL1RAP|LAMP1|IL17F|SMAD4|MAP4K4|CPLX2|TGFB2|ATG5|TLR4|TNFRSF1B|IL33|JAK2|IL36RN|CXCL12|TNFSF11|IL6|TLR6|MAPKBP1|PDCD6IP|IL10|TRAF6|HIF1A|TNFRSF11A|MAP2K6|CCL5|TNFRSF9 | 77 | 351|350|151|336|265|243|444|432|518|331|322|345|126|383|153|497|513|222|418|227|199|360|367|470|254|216|376|312|342|276|346|309|536|181|430|218|305|298|307|425|315|495|163|255|135|473 | 2.89E-09 |  | GO.0051046 | 0.72533658 | 0 |
| 33 | 11 | GO Process |  | MyD88-dependent toll-like receptor signaling pathway | 5.58E-08 | IRAK2|TNIP1|TAB2|MAP3K7|IRAK1|TLR4|TAB3|MAP3K1|TLR6|IRAK4|TRAF6 | 77 | 258|538|184|358|434|342|206|397|305|371|315 | 2.89E-09 |  | GO.0002755 | 0.72533658 | 0 |
| 4979 | 174 | UniProt Keywords | | Cytoplasm | 5.82E-08 | MAP4K5|RELT|MAPK1|TNFAIP1|NFKB1|CDKN1B|RHOQ|ATG14|TRAF2|TXNDC17|XPO4|BIVM|DOCK10|TRAF5|MAPK6|FAF2|SMAD7|SMAD2|TRAF4|SMURF2|MAP3K8|IL1B|ITPKC|ATF2|STAT3|MAP3K13|PRDX5|CDK6|CCAR1|HPS5|DOCK3|ETNK1|BNIP2|IRF8|SOD1|ACKR3|DOCK5|PDCD4|DOCK1|EPG5|ATG10|ATG3|AIFM1|BAG4|LY6K|IL17RD|HIF1AN|HPS6|NFATC3|MAP2K1|BCL2L1|MAEA|IL16|NKIRAS2|MAP3K11|ATG4D|OXSR1|TNIP1|EEA1|PRKRA|C8orf4|AATK|NFATC2IP|PRX|TNFAIP8L3|PAWR|PIWIL3|IKZF1|SMAD3|BCL11A|SMAD4|MAP4K4|IKZF3|RBPJ|ATG7|AREL1|IRF5|FXR1|MAP3K5|MAPK10|CPLX2|SMURF1|ILF2|IRF2BP2|IRF6|MAPKAPK2|TAB2|PEX19|THEMIS|MCL1|ATG5|BAG3|MAP3K7|SIKE1|IRAK1BP1|IRAK1|BCL10|IFI44|IFI44L|EXOSC1|IFIT3|IFIT2|YWHAB|AGO3|AGO1|AGO4|TRAF3IP1|TXNDC8|ZAK|HIF3A|PLCXD3|JAK2|IL17RE|EXOSC8|LYST|ATG16L1|MAP3K4|TANK|PDCD10|PRC1|CIAPIN1|AKTIP|MAPK8|NFATC2|IRF9|MAP2K7|TNFSF11|MAPK4|GATSL3|MAP3K2|NFATC4|IRAK4|PEX5|MAPKBP1|NKIRAS1|MAPK9|ITK|TNIK|ILF3|DOCK4|MAP2K4|LMLN|PDCD6IP|TICAM2|PEX5L|ATG12|SMAD1|TNFAIP8|IKBKB|API5|ATG13|TRAF6|BCLAF1|DICER1|HIF1A|SMAD5|RASSF3|TNFAIP8L1|APAF1|MAP3K12|MAPKAPK5|TRAF3|NFAT5|LITAF|TXNIP|IKBKE|IER2|ERC1|MAP2K6|ECSCR|TNFAIP3|SOCS7|MAP3K14|GATSL2 | 77 | 451|532|469|280|529|285|552|150|444|140|386|219|505|142|235|458|195|200|194|362|165|345|420|189|334|490|208|365|111|366|225|191|187|398|281|247|406|311|215|361|155|113|482|409|503|136|228|209|210|292|372|207|385|273|439|213|504|538|146|196|293|560|347|319|248|396|182|220|241|507|470|254|392|438|119|437|160|245|335|380|216|464|443|178|487|268|184|521|349|278|312|318|358|338|400|434|323|179|177|137|404|394|440|520|239|240|474|238|264|510|407|309|465|297|555|223|466|314|316|511|472|424|282|115|492|236|430|231|288|374|249|371|514|298|417|250|556|535|110|544|517|333|307|329|328|421|193|185|526|463|559|315|180|121|495|201|221|453|148|426|370|557|393|395|415|548|354|401|255|253|164|186|171|402 | 2.09E-09 |  | KW-0963 | 0.723507702 | 0 |
| 4144 | 151 | GO Process |  | system development | 5.99E-08 | CX3CL1|NOX3|TNFRSF1A|TGFBR3|MAPK1|TGFB1|CDKN1B|LOX|BCL9|TGFB3|TNFSF10|CCR7|LIF|BCL2L2|DOCK7|EXOC4|PDGFRA|DOCK10|ARG2|PDGFRB|MAPK6|LOXL1|MMD|SMAD7|SMAD2|RIMS2|TRAF4|DGCR2|NOX4|IL7|ATF2|IL11|STAT3|CDK6|IRF8|SOD1|ILDR2|ACKR3|DOCK11|PDCD4|CSF1R|AIFM1|SMAD6|CXCR5|PEX13|TNFRSF21|TNFRSF11B|AVPR1A|MAP2K1|BCL2L1|MAEA|ALCAM|IL7R|PPARD|CFLAR|IL1RAP|RHOJ|PRKRA|ICOS|PRX|TNFRSF12A|CSF1|IL25|SOCS3|IKZF1|CCR4|SMAD3|BCL9L|CXCL14|IL17F|BCL11A|BAG5|SMAD4|MAP4K4|GPR29|RBPJ|BCL11B|FXR1|CCR9|TGFBR2|MAPK10|CPLX2|SMURF1|MAP3K3|TGFB2|IRF6|MAPKAPK2|TAB2|THEMIS|IL6R|ATG5|BAG3|IGSF3|BCL10|IL1RAPL2|AGO4|TRAF3IP1|CXCR3|TGIF2|TGFBR1|TNFRSF1B|HIF3A|BCOR|NFIB|IRF4|IL6ST|IL33|JAK2|TNFRSF19|LOXL2|MAP3K4|PDCD10|BCL2L11|CIAPIN1|ULK2|MAPK8|CXCL12|BLOC1S5|BCL2|TNFSF11|CMKBR6|BCL6|PPARA|IL6|LTBP1|CXCR4|IL18R1|NFATC4|MAPK9|ITK|LY6H|TNIK|MAP2K4|IL10|TGFBI|SMAD1|AMBRA1|TRAF6|DICER1|HIF1A|SMAD5|TET2|APAF1|TXNIP|TNFRSF11A|IER2|NOS1|ECSCR|SOCS2|TNFAIP3|SOCS7 | 77 | 325|203|351|447|469|350|285|168|175|265|202|243|432|551|413|159|516|505|518|506|235|498|427|195|200|331|194|405|364|266|189|126|334|365|398|281|237|247|422|311|153|482|369|306|152|513|359|222|292|372|207|286|176|227|133|199|375|196|167|319|340|149|478|246|220|502|241|125|468|367|507|124|470|254|448|438|128|245|456|332|380|216|464|299|376|487|268|184|349|348|312|318|270|323|252|240|474|230|259|368|276|510|541|170|169|391|346|309|256|480|466|316|234|472|214|282|181|198|475|430|431|310|491|218|154|450|287|249|250|556|357|535|517|425|157|193|131|315|121|495|201|294|148|415|163|354|508|253|419|164|186 | 3.13E-09 |  | GO.0048731 | 0.722257318 | 0 |
| 54 | 13 | Reactome Pathways | | Nucleotide-binding domain, leucine rich repeat containing receptor (NLR) signaling pathways | 5.99E-08 | NFKB1|IRAK2|BCL2L1|TAB2|MAP3K7|IRAK1|TAB3|BCL2|IKBKB|TRAF6|TXNIP|MAP2K6|TNFAIP3 | 77 | 529|258|372|184|358|434|206|475|526|315|415|255|164 | 3.02E-09 |  | HSA-168643 | 0.722257318 | 0 |
| 21 | 10 | InterPro Domains | | Chemokine receptor family | 6.34E-08 | CCR7|CXCR5|CCR4|CCRL2|CCR9|CXCR3|CMKBR6|CXCR4|CXCR6|ACKR2 | 77 | 243|306|502|134|456|230|431|450|435|173 | 9.64E-10 |  | IPR000355 | 0.719791074 | 0 |
| 2065 | 91 | GO Process |  | phosphate-containing compound metabolic process | 6.58E-08 | MAP4K5|TGFBR3|MAPK1|TGFB1|NFKB1|CDKN1B|RHOQ|LIF|NDUFA10|MAP3K10|IRAK2|PDGFRA|TBRG4|PDGFRB|MAPK6|MMD|SMAD7|SMAD2|MAP3K8|IL1B|ITPKC|MAP4K3|STAT3|MAP3K13|PRDX5|CDK6|ETNK1|CSF1R|TRAT1|IL17RD|RICTOR|MAP2K1|MAP3K11|PPARD|OXSR1|PRKRA|ICOS|AATK|TNFAIP8L3|MAP4K4|MAP2K3|MAP3K15|TGFBR2|MAP3K5|MAPK10|MAP3K3|KIAA1804|TGFB2|MAPKAPK2|TAB2|MAP3K7|IRAK1|PI4K2A|YWHAB|TLR4|IL2RG|TGFBR1|ZAK|MAP3K19|TAB3|JAK2|TNFRSF19|MAP3K4|ULK2|MAPK8|MAP2K7|BCL2|TNFSF11|MAP3K1|MAPK4|CSF2RB|LTBP1|MAP3K2|IRAK4|MAPK9|ITK|TNIK|ILF3|MAP2K4|SMAD1|IKBKB|TRAF6|SMAD5|MAP3K12|MAPKAPK5|MAP3K9|IKBKE|ERC1|MAP2K6|CCL5|MAP3K14 | 77 | 451|447|469|350|529|285|552|432|275|489|258|516|496|506|235|427|195|200|165|345|420|141|334|490|208|365|191|153|527|136|302|292|439|227|504|196|167|560|248|254|528|384|332|335|380|299|190|376|268|184|358|434|399|440|342|563|368|264|352|206|309|256|466|214|282|236|475|430|397|231|524|154|374|371|250|556|535|110|517|193|526|315|201|426|370|290|548|401|255|135|171 | 3.46E-09 |  | GO.0006796 | 0.718177411 | 0 |
| 76 | 14 | KEGG Pathways | | Chronic myeloid leukemia | 7.05E-08 | MAPK1|TGFB1|NFKB1|CDKN1B|TGFB3|CDK6|MAP2K1|BCL2L1|SMAD3|SMAD4|TGFBR2|TGFB2|TGFBR1|IKBKB | 77 | 469|350|529|285|265|365|292|372|241|470|332|376|368|526 | 1.50E-08 |  | hsa05220 | 0.715181088 | 0 |
| 1596 | 76 | GO Process |  | positive regulation of RNA metabolic process | 7.39E-08 | TNFRSF1A|MAPK1|TGFB1|NFKB1|BCL9|TGFB3|RHOQ|RASL11A|LIF|PIAS1|SMAD7|SMAD2|IL1A|IL1B|ATF2|IL11|STAT3|CCAR1|IRF8|SMAD6|CREBRF|NFATC3|MAP2K1|PPARD|TNIP1|NFATC2IP|IL25|IKZF1|SMAD3|BCL9L|IL17F|BCL11A|SMAD4|IKZF3|MAP2K3|RBPJ|BCL11B|IRF5|MAP3K5|ILF2|TGFB2|IRF6|IRAK1|BCL10|AGO1|CXCR3|TLR4|TGFBR1|TNFRSF1B|HIF3A|NFIB|IRF4|IL33|NFATC2|TNFSF11|PPARA|IL6|MAP3K2|NFATC4|NCOA2|ILF3|IL10|IKZF2|SMAD1|IKBKB|TRAF6|BCLAF1|HIF1A|SMAD5|NKRF|TET2|NFAT5|LITAF|IER2|PIAS2|NOS1 | 77 | 351|469|350|529|175|265|552|205|432|554|195|200|322|345|189|126|334|111|398|369|296|210|292|227|538|347|478|220|241|125|367|507|470|392|528|438|128|160|335|443|376|487|434|323|239|230|342|368|276|510|170|169|346|115|430|491|218|374|249|455|110|425|486|193|526|315|180|495|201|291|294|393|395|354|284|508 | 3.91E-09 |  | GO.0051254 | 0.713135556 | 0 |
| 896 | 52 | GO Process |  | cellular response to oxygen-containing compound | 7.40E-08 | MAPK1|TGFB1|CXCL6|NFKB1|SOGA1|RHOQ|TRAF2|IRAK2|PDGFRA|FDX1|NOX4|IL1B|STAT3|PRDX5|IRF8|SOD1|PDCD4|TNFSF4|AIFM1|AVPR1A|BCL2L1|IL12A|PPARD|CFLAR|TNIP1|LAMTOR4|CCL20|MAP3K5|IRAK1|TLR4|TNFRSF1B|CCL7|JAK2|PDCD10|MAPK8|GATSL3|IL6|NFATC4|TLR6|MAPK9|OXR1|MAP2K4|IL10|TICAM2|TRAF6|SOD2|LITAF|MAP2K6|CCL5|SOCS2|TNFAIP3|SOCS7 | 77 | 469|350|531|529|317|552|444|258|516|353|364|345|334|208|398|281|311|383|482|222|372|519|227|133|538|188|308|335|434|342|276|289|309|316|282|288|218|249|305|250|144|517|425|329|315|381|395|255|135|419|164|186 | 3.94E-09 |  | GO.1901701 | 0.713076828 | 0 |
| 288 | 27 | GO Process |  | regulation of neuron death | 7.40E-08 | TGFB3|TRAF2|ATF2|STAT3|SOD1|AIFM1|BCL2L1|IL13|MAP3K11|ATG7|MAP3K5|TGFB2|MCL1|TLR4|JAK2|BCL2L11|BCL2|PPARA|TLR6|OXR1|MAP2K4|IL10|AMBRA1|HIF1A|SOD2|MAP3K12|CCL5 | 77 | 265|444|189|334|281|482|372|418|439|119|335|376|278|342|309|234|475|491|305|144|517|425|131|495|381|426|135 | 3.95E-09 |  | GO.1901214 | 0.713076828 | 0 |
| 124 | 18 | GO Process |  | regulation of production of molecular mediator of immune response | 7.40E-08 | TGFB1|TGFB3|TRAF2|SMAD7|IL1B|TNFSF4|IL13|TGFB2|ATG5|MAP3K7|BCL10|IL33|BCL6|IL6|IL1R1|IL18R1|IL10|TRAF6 | 77 | 350|265|444|195|345|383|418|376|312|358|323|346|310|218|274|287|425|315 | 3.93E-09 |  | GO.0002700 | 0.713076828 | 0 |
| 25 | 10 | GO Function | | tumor necrosis factor-activated receptor activity | 8.15E-08 | RELT|TNFRSF1A|TNFRSF10B|TNFRSF21|TNFRSF11B|TNFRSF6B|TNFRSF1B|TNFRSF19|TNFRSF11A|TNFRSF9 | 77 | 532|351|410|513|359|481|276|256|163|473 | 3.68E-09 |  | GO.0005031 | 0.708884239 | 0 |
| 662 | 43 | GO Process |  | regulation of establishment of protein localization | 8.51E-08 | MAPK1|TGFB1|CRTAM|IL1RL1|TGFB3|CCR7|TRAF2|XPO4|ARG2|IL1A|IL1B|TNFSF4|CSF1R|BAG4|IL17RB|TNFRSF21|CREBRF|IL13|PPARD|IL1RAP|SMAD3|IL17F|MAP4K4|TGFB2|ATG5|BAG3|YWHAB|TLR4|BNIP3L|IL33|JAK2|PDCD10|IL36RN|MAPK8|BCL2|IL6|TLR6|MAPKBP1|IL10|TRAF6|HIF1A|CCL5|TNFRSF9 | 77 | 469|350|151|336|265|243|444|386|518|322|345|383|153|409|497|513|296|418|227|199|241|367|254|376|312|318|440|342|499|346|309|316|536|282|475|218|305|298|425|315|495|135|473 | 4.57E-09 |  | GO.0070201 | 0.707007044 | 0 |
| 96 | 16 | GO Process |  | regulation of cytokine biosynthetic process | 9.32E-08 | NFKB1|IL1A|IL1B|IL9|TNFRSF13C|IL17F|MAP2K3|CCL20|MAPKAPK2|BCL10|TLR4|IRF4|IL6|TLR6|IL10|TRAF6 | 77 | 529|322|345|295|224|367|528|308|268|323|342|169|218|305|425|315 | 5.02E-09 |  | GO.0042035 | 0.703058409 | 0 |
| 69 | 14 | GO Process |  | positive regulation of NIK/NF-kappaB signaling | 9.41E-08 | TRAF2|TRAF4|IL1B|TNFRSF10B|PDCD4|C8orf4|MAP3K7|IRAK1|AGO3|AGO1|TLR4|IL18R1|TLR6|TRAF6 | 77 | 444|194|345|410|311|293|358|434|520|239|342|287|305|315 | 5.08E-09 |  | GO.1901224 | 0.702641038 | 0 |
| 126 | 18 | Reactome Pathways | | Fc epsilon receptor (FCERI) signaling | 9.48E-08 | MAPK1|NFKB1|NFATC3|MAPK10|TAB2|MAP3K7|BCL10|TAB3|MAPK8|NFATC2|MAP2K7|MAP3K1|MAPK9|ITK|MAP2K4|LAT2|IKBKB|TRAF6 | 77 | 469|529|210|380|184|358|323|206|282|115|236|397|250|556|517|467|526|315 | 4.93E-09 |  | HSA-2454202 | 0.702319166 | 0 |
| 111 | 17 | GO Process |  | negative regulation of leukocyte cell-cell adhesion | 9.57E-08 | TGFB1|CCL21|ARG2|SMAD7|TNFSF4|TNFRSF21|CTLA4|SOCS5|PAWR|SOCS1|CCL28|CXCL12|PDCD1LG2|SOCS6|BCL6|PPARA|IL10 | 77 | 350|500|518|195|383|513|183|226|396|460|488|181|373|244|310|491|425 | 5.19E-09 |  | GO.1903038 | 0.701908806 | 0 |
| 5085 | 175 | GO Process |  | anatomical structure development | 1.08E-07 | CX3CL1|RELT|NOX3|TNFRSF1A|TGFBR3|MAPK1|TGFB1|CDKN1B|LOX|BCL9|TGFB3|TNFSF10|CCR7|LIF|BCL2L2|DOCK7|EXOC4|PDGFRA|DOCK10|CCL21|IER3|ARG2|PDGFRB|MAPK6|LOXL1|MMD|SMAD7|SMAD2|RIMS2|TRAF4|DGCR2|NOX4|IL1A|IL1B|IL7|ATF2|IL11|STAT3|CDK6|IRF8|SOD1|ILDR2|ACKR3|DOCK11|TNFRSF10B|PDCD4|CSF1R|AIFM1|SMAD6|CXCR5|PEX13|RICTOR|TNFRSF21|TNFRSF11B|DEFB1|AVPR1A|MAP2K1|BCL2L1|MAEA|ALCAM|IL7R|PPARD|CFLAR|IL1RAP|RHOJ|PRKRA|ICOS|PRX|TNFRSF12A|CSF1|TGIF1|IL25|PIWIL3|SOCS3|IKZF1|CCR4|SMAD3|PDCD1|BCL9L|CXCL14|IL17F|BCL11A|BAG5|SMAD4|MAP4K4|GPR29|IKZF3|RBPJ|BCL11B|FXR1|CCR9|TGFBR2|MAPK10|CPLX2|SMURF1|MAP3K3|TGFB2|IRF6|MAPKAPK2|TAB2|HIVEP2|THEMIS|IL6R|MCL1|ATG5|BAG3|IGSF3|TNFRSF6B|BCL10|IL1RAPL2|AGO4|TRAF3IP1|CXCR3|TGIF2|TXNDC8|TGFBR1|ZAK|TNFRSF1B|HIF3A|BCOR|NFIB|IRF4|IL6ST|IL33|JAK2|TNFRSF19|TXNRD3|LOXL2|MAP3K4|PDCD10|BCL2L11|CIAPIN1|ULK2|MAPK8|CXCL12|NFATC2|BLOC1S5|BCL2|TNFSF11|CMKBR6|BCL6|PPARA|IL6|LTBP1|CXCR4|IL18R1|NFATC4|MAPK9|ITK|LY6H|TNIK|VSIG1|MAP2K4|IL10|TGFBI|ACKR2|SMAD1|IKBKB|AMBRA1|TRAF6|DICER1|HIF1A|SMAD5|TET2|APAF1|TXNIP|TNFRSF11A|IER2|ERC1|NOS1|TNFRSF9|ECSCR|SOCS2|TNFAIP3|SOCS7 | 77 | 325|532|203|351|447|469|350|285|168|175|265|202|243|432|551|413|159|516|505|500|461|518|506|235|498|427|195|200|331|194|405|364|322|345|266|189|126|334|365|398|281|237|247|422|410|311|153|482|369|306|152|302|513|359|112|222|292|372|207|286|176|227|133|199|375|196|167|319|340|149|143|478|182|246|220|502|241|537|125|468|367|507|124|470|254|448|392|438|128|245|456|332|380|216|464|299|376|487|268|184|433|349|348|278|312|318|270|481|323|252|240|474|230|259|238|368|264|276|510|541|170|169|391|346|309|256|263|480|466|316|234|472|214|282|181|115|198|475|430|431|310|491|218|154|450|287|249|250|556|357|535|378|517|425|157|173|193|526|131|315|121|495|201|294|148|415|163|354|401|508|473|253|419|164|186 | 5.91E-09 |  | GO.0048856 | 0.696657624 | 0 |
| 274 | 26 | GO Process |  | regulation of ERK1 and ERK2 cascade | 1.12E-07 | CX3CL1|TGFB1|CCL1|CCL13|CCR7|LIF|PDGFRA|CCL21|PDGFRB|NOX4|IL1B|ACKR3|CSF1R|MAP2K1|CFLAR|TNIP1|TNFAIP8L3|SMAD4|CCL20|TLR4|CCL7|TNFSF11|TNFRSF11A|CCL5|CCL16|CCL4 | 77 | 325|350|484|485|243|432|516|500|506|364|345|247|153|292|133|538|248|470|308|342|289|430|163|135|501|118 | 6.11E-09 |  | GO.0070372 | 0.695078198 | 0 |
| 52 | 13 | InterPro Domains | | SMAD/FHA domain superfamily | 1.21E-07 | SMAD7|SMAD2|IRF8|SMAD6|SNIP1|SMAD3|SMAD4|IRF5|IRF6|IRF4|IRF9|SMAD1|SMAD5 | 77 | 195|200|398|369|337|241|470|160|487|169|492|193|201 | 2.04E-09 |  | IPR008984 | 0.691721463 | 0 |
| 5401 | 183 | GO Process |  | developmental process | 1.21E-07 | CX3CL1|RELT|NOX3|TNFRSF1A|TGFBR3|MAPK1|TGFB1|CDKN1B|LOX|BCL9|TGFB3|TNFSF10|CCR7|LIF|PIAS1|BCL2L2|DOCK7|EXOC4|PDGFRA|DOCK10|CCL21|IER3|ARG2|PDGFRB|MAPK6|LOXL1|MMD|SMAD7|SMAD2|RIMS2|TRAF4|DGCR2|NOX4|IL1A|IL1B|IL7|ATF2|IL11|STAT3|CDK6|IRF8|SOD1|ILDR2|ACKR3|DOCK11|TNFRSF10B|PDCD4|CSF1R|AIFM1|SMAD6|CXCR5|PEX13|RICTOR|TNFRSF21|TNFRSF11B|DEFB1|AVPR1A|HPS6|MAP2K1|BCL2L1|MAEA|ALCAM|IL7R|PPARD|CFLAR|IL1RAP|RHOJ|PRKRA|ICOS|PRX|TNFRSF12A|CSF1|TGIF1|IL25|PIWIL3|SOCS3|IKZF1|CCR4|SMAD3|PDCD1|BCL9L|CXCL14|IL17F|BCL11A|BAG5|SMAD4|MAP4K4|GPR29|IKZF3|RBPJ|ATG7|BCL11B|FXR1|CCR9|TGFBR2|MAPK10|CPLX2|SMURF1|MAP3K3|TGFB2|IRF6|MAPKAPK2|TAB2|HIVEP2|THEMIS|IL6R|MCL1|ATG5|BAG3|IGSF3|IRAK1|TNFRSF6B|BCL10|IL1RAPL2|AGO4|TRAF3IP1|CXCR3|TGIF2|TXNDC8|TGFBR1|ZAK|TNFRSF1B|HIF3A|BCOR|NFIB|IRF4|IL6ST|IL33|JAK2|TNFRSF19|TXNRD3|LOXL2|MAP3K4|PDCD10|BCL2L11|CIAPIN1|ULK2|MAPK8|CXCL12|NFATC2|BLOC1S5|BCL2|TNFSF11|CMKBR6|BCL6|PPARA|IL6|LTBP1|CXCR4|IL18R1|NFATC4|MAPK9|ITK|LY6H|TNIK|VSIG1|MAP2K4|BCL7B|IL10|TGFBI|ACKR2|SMAD1|IKBKB|AMBRA1|TRAF6|DICER1|HIF1A|SMAD5|TET2|SOD2|APAF1|MAPKAPK5|LITAF|TXNIP|TNFRSF11A|IER2|ERC1|NOS1|TNFRSF9|ECSCR|SOCS2|TNFAIP3|SOCS7 | 77 | 325|532|203|351|447|469|350|285|168|175|265|202|243|432|554|551|413|159|516|505|500|461|518|506|235|498|427|195|200|331|194|405|364|322|345|266|189|126|334|365|398|281|237|247|422|410|311|153|482|369|306|152|302|513|359|112|222|209|292|372|207|286|176|227|133|199|375|196|167|319|340|149|143|478|182|246|220|502|241|537|125|468|367|507|124|470|254|448|392|438|119|128|245|456|332|380|216|464|299|376|487|268|184|433|349|348|278|312|318|270|434|481|323|252|240|474|230|259|238|368|264|276|510|541|170|169|391|346|309|256|263|480|466|316|234|472|214|282|181|115|198|475|430|431|310|491|218|154|450|287|249|250|556|357|535|378|517|174|425|157|173|193|526|131|315|121|495|201|294|381|148|370|395|415|163|354|401|508|473|253|419|164|186 | 6.67E-09 |  | GO.0032502 | 0.691721463 | 0 |
| 724 | 45 | GO Process |  | interspecies interaction between organisms | 1.26E-07 | TNFRSF1A|MAPK1|TGFB1|CCL1|CCL13|PDGFRA|CCL21|STAT3|ACKR3|CXCL3|BCL2L1|IL16|DEFB4A|CXCL11|PAAF1|CFLAR|TNIP1|EEA1|SMAD3|LAMP1|CXCL14|ATG7|CCL20|MAP3K5|CCL28|CXCL9|ATG5|MAP3K7|IRAK1|YWHAB|IL2RG|BNIP3L|IL6ST|ATG16L1|TANK|BCL2L11|ULK2|CXCL12|CXCR4|CXCR6|HLA-B|PDCD6IP|DEFB4B|CCL5|CCL4 | 77 | 351|469|350|484|485|516|500|334|247|356|372|385|158|321|411|133|538|146|241|360|468|119|308|335|488|283|312|358|434|440|563|499|391|223|314|234|214|181|450|435|114|307|166|135|118 | 6.96E-09 |  | GO.0044419 | 0.689962945 | 0 |
| 4050 | 147 | GO Process |  | regulation of cellular macromolecule biosynthetic process | 1.36E-07 | TNFRSF1A|TGFBR3|MAPK1|TGFB1|TNFAIP1|NFKB1|CDKN1B|BCL9|TGFB3|RHOQ|RASL11A|CCR7|TRAF2|LIF|PIAS1|MAP3K10|IRAK2|CCL21|TRAF5|PDGFRB|SMAD7|SMAD2|SMURF2|NOX4|IL1A|IL1B|ATF2|IL11|STAT3|MAP3K13|PRDX5|CCAR1|IRF8|PDCD4|TNFSF4|GATAD1|SMAD6|TGFA|SNIP1|CREBRF|HIF1AN|NFATC3|MAP2K1|IL16|PPARD|CFLAR|IL1RAP|TNIP1|C8orf4|NFATC2IP|TGIF1|PAWR|IL25|PIWIL3|IKZF1|SMAD3|BCL9L|IL17F|BCL11A|SMAD4|IKZF3|MAP2K3|RBPJ|ATG7|PEX2|BCL11B|IRF5|FXR1|MAP3K5|SMURF1|ILF2|IRF2BP2|TGFB2|IRF6|TAB2|HIVEP2|GATAD2B|IKZF5|BAG3|MAP3K7|IRAK1BP1|IRAK1|BCL10|IGSF1|LCOR|YWHAB|AGO3|AGO1|AGO4|CXCR3|TGIF2|TLR4|TGFBR1|HIF3A|BCOR|TAB3|NFIB|IRF4|IL33|JAK2|LOXL2|MAP3K4|NFATC2|IRF9|MAP2K7|BCL2|TNFSF11|BCL6|PPARA|IL6|IL18R1|MAP3K2|NFATC4|TLR6|IRAK4|EIF4E3|NCOA2|ILF3|MAP2K4|IL10|IKZF2|SMAD1|IKBKB|TRAF6|BCLAF1|DICER1|BCORL1|HIF1A|SMAD5|NKRF|TET2|BCL7A|SOD2|MAPKAPK5|MAP3K9|TRAF3|NFAT5|LITAF|TXNIP|TNFRSF11A|IER2|PIAS2|ERC1|MAP2K6|CCL5|NOS1|TNFAIP3 | 77 | 351|447|469|350|280|529|285|175|265|552|205|243|444|432|554|489|258|500|142|506|195|200|362|364|322|345|189|126|334|490|208|111|398|311|383|530|369|279|337|296|228|210|292|385|227|133|199|538|293|347|143|396|478|182|220|241|125|367|507|470|392|528|438|119|269|128|160|245|335|464|443|178|376|487|184|433|414|412|318|358|400|434|323|132|233|440|520|239|240|230|259|342|368|510|541|206|170|169|346|309|480|466|115|492|236|475|430|310|491|218|287|374|249|305|371|403|455|110|517|425|486|193|526|315|180|121|387|495|201|291|294|303|381|370|290|557|393|395|415|163|354|284|401|255|135|508|164 | 7.51E-09 |  | GO.2000112 | 0.686646109 | 0 |
| 362 | 30 | GO Process |  | regulation of immune effector process | 1.36E-07 | TGFB1|CRTAM|TGFB3|TRAF2|SMAD7|IL1B|TNFSF4|IL12A|IL13|SOCS5|IL7R|IFNLR1|LAMP1|TGFB2|ATG5|MAP3K7|BCL10|TRAF3IP1|IRF4|IL33|BCL6|IL6|IL1R1|IL18R1|HLA-DMB|HLA-B|IL10|TRAF6|TRAF3|TNFAIP3 | 77 | 350|151|265|444|195|345|383|519|418|226|176|341|360|376|312|358|323|474|169|346|310|218|274|287|553|114|425|315|557|164 | 7.58E-09 |  | GO.0002697 | 0.686646109 | 0 |
| 130 | 18 | GO Process |  | positive regulation of lymphocyte proliferation | 1.37E-07 | TNFSF9|IL1B|IL7|TNFSF4|TNFRSF13C|IL12A|IL13|TGFBR2|TNFSF13B|IL6ST|NFATC2|PDCD1LG2|BCL2|BCL6|IL6|HLA-DMB|TRAF6|CCL5 | 77 | 120|345|266|383|224|519|418|332|326|391|115|373|475|310|218|553|315|135 | 7.66E-09 |  | GO.0050671 | 0.686327943 | 0 |
| 72 | 14 | GO Process |  | negative regulation of cellular response to transforming growth factor beta stimulus | 1.47E-07 | TGFBR3|TGFB1|TGFB3|SMAD7|SMAD2|SMURF2|SMAD6|CFLAR|SMAD3|BCL9L|TGFBR2|SMURF1|TGFBR1|LTBP1 | 77 | 447|350|265|195|200|362|369|133|241|125|332|464|368|154 | 8.20E-09 |  | GO.1903845 | 0.683268267 | 0 |
| 321 | 28 | GO Process |  | response to oxygen levels | 1.55E-07 | TGFBR3|TGFB1|CDKN1B|TGFB3|HILPDA|PDGFRB|NOX4|AIFM1|HIF1AN|PPARD|CFLAR|SMAD3|SMAD4|RBPJ|ATG7|TGFBR2|TGFB2|IRAK1|HIF3A|BNIP3L|LOXL2|CXCL12|BCL2|PPARA|CXCR4|HIF1A|APAF1|NOS1 | 77 | 447|350|285|265|462|506|364|482|228|227|133|241|470|438|119|332|376|434|510|499|480|181|475|491|450|495|148|508 | 8.72E-09 |  | GO.0070482 | 0.68096683 | 0 |
| 259 | 25 | GO Process |  | positive regulation of innate immune response | 1.56E-07 | NFKB1|CRTAM|IRAK2|IL12A|TNIP1|LAMP1|MAPKAPK2|TAB2|MAP3K7|IRAK1|BCL10|TLR4|TAB3|TANK|MAP3K1|TLR6|IRAK4|TICAM2|IKBKB|TRAF6|TRAF3|IKBKE|MAP2K6|CCL5|TNFAIP3 | 77 | 529|151|258|519|538|360|268|184|358|434|323|342|206|314|397|305|371|329|526|315|557|548|255|135|164 | 8.79E-09 |  | GO.0045089 | 0.68068754 | 0 |
| 60 | 13 | GO Process |  | positive regulation of tyrosine phosphorylation of STAT protein | 1.61E-07 | TNFRSF1A|LIF|STAT3|CSF1R|IL12A|IL13|SOCS3|IL20|IL6R|IL6ST|JAK2|IL6|CCL5 | 77 | 351|432|334|153|519|418|246|161|348|391|309|218|135 | 9.08E-09 |  | GO.0042531 | 0.679317412 | 0 |
| 38 | 11 | GO Process |  | activation of JUN kinase activity | 1.75E-07 | MAP4K5|MAP3K10|MAP3K11|MAP3K5|KIAA1804|ZAK|MAP2K7|TNFSF11|MAP3K2|MAP2K4|MAP3K9 | 77 | 451|489|439|335|190|264|236|430|374|517|290 | 9.91E-09 |  | GO.0007257 | 0.675696195 | 0 |
| 131 | 18 | GO Function | | growth factor receptor binding | 1.77E-07 | PDGFRA|IL36G|IL36B|PDGFRB|IL1A|IL1B|IL7|IL11|IL9|TGFA|IL12A|SOCS5|JAK2|IL36RN|IL6|IL1R1|IRAK4|IL10 | 77 | 516|561|558|506|322|345|266|126|295|279|519|226|309|536|218|274|371|425 | 8.53E-09 |  | GO.0070851 | 0.675202673 | 0 |
| 61 | 13 | GO Process |  | lymphocyte migration | 1.90E-07 | CX3CL1|CCL1|CCL13|CCR7|CCL21|CXCL11|GPR29|CCL20|CXCL9|CXCR3|CXCL12|CMKBR6|CCL16 | 77 | 325|484|485|243|500|321|448|308|283|230|181|431|501 | 1.08E-08 |  | GO.0072676 | 0.67212464 | 0 |
| 74 | 14 | GO Process |  | regulation of chemokine production | 1.95E-07 | IL1RL1|ARG2|IL1A|IL1B|IL7|CSF1R|SOCS5|IL6R|TLR4|IL33|IL6|IL10|TICAM2|HIF1A | 77 | 336|518|322|345|266|153|226|348|342|346|218|425|329|495 | 1.11E-08 |  | GO.0032642 | 0.670996539 | 0 |
| 11238 | 323 | GO Component | | cytoplasm | 2.00E-07 | MAP4K5|RELT|NOX3|TNFRSF1A|LAPTM4A|TGFBR3|MAPK1|TGFB1|TNFAIP1|NFKB1|CDKN1B|EXOC2|NKTR|BCL9|TGFB3|RHOQ|TMX4|LAMP5|CCR7|ATG14|TRAF2|LIF|ISLR|TXNDC17|BCL2L2|NDUFA10|PXDN|MAP3K10|EXOC4|XPO4|IRAK2|PDGFRA|BIVM|HILPDA|DOCK10|TBRG4|IER3|FDX1|BCL2L10|TRAF5|ARG2|PDGFRB|MAPK6|LOXL1|FAF2|MMD|MSR1|WDR59|SMAD7|SMAD2|TRAF4|SMURF2|MAP3K8|NOX4|IL1A|IL1B|ITPKC|MAP4K3|ATF2|IL11|STAT3|MAP3K13|PECR|PRDX5|LAMP3|CDK6|CCAR1|HPS5|DOCK3|ETNK1|BNIP2|IRF8|SOD1|ILDR2|ACKR3|DOCK11|DOCK5|ENDOD1|PDCD4|DOCK1|EPG5|ATG10|TXNDC11|ATG3|DEPTOR|AIFM1|BAG4|IL17RB|SMAD6|LY6K|WDR24|PEX13|TGFA|SNIP1|IL17RD|RICTOR|CREBRF|IL22RA2|DEFB1|IKBIP|HIF1AN|AVPR1A|HPS6|TMX3|NFATC3|MAP2K1|BCL2L1|MAEA|IL16|IL12A|DEFB4A|NKIRAS2|CTLA4|IL13|SOCS5|IL7R|MAP3K11|ATG4D|OXSR1|CFLAR|RHOJ|TNIP1|EEA1|PRKRA|C8orf4|MICU3|AATK|NFATC2IP|PRX|CSF1|TNFAIP8L3|PAWR|SOCS1|PIWIL3|SOCS3|IKZF1|SMAD3|LAMP1|CXCL14|BCL11A|BAG5|MIOS|TCAIM|SMAD4|NECAP1|LAMTOR4|MAP4K4|IKZF3|MAP2K3|RBPJ|ATG7|AREL1|PEX2|IRF5|FXR1|TGFBR2|MAP3K5|MAPK10|CPLX2|ATG2B|SMURF1|MAP3K3|ILF2|NOS1AP|IRF2BP2|KIAA1804|TGFB2|IRF6|TAGAP|MAPKAPK2|TAB2|PEX3|PEX19|THEMIS|MCL1|ATG5|BAG3|PEX11B|MAP3K7|CMC4|SIKE1|IRAK1BP1|IRAK1|BCL10|PI4K2A|BAG2|IFI44|IFI44L|EXOSC1|IFIT3|IFIT2|YWHAB|AGO3|AGO1|AGO4|TRAF3IP1|CXCR3|TLR4|IL2RG|TXNDC8|HLA-DQB1|TGFBR1|ZAK|MAP3K19|TNFSF13B|TNFRSF1B|DOCK9|HIF3A|PLCXD3|TAB3|BCAP29|TXNDC5|BNIP3L|IRF4|IL33|JAK2|TXNRD3|IL17RE|LOXL2|EXOSC8|LYST|ATG16L1|MAP3K4|TANK|PDCD10|BCL2L11|BCL2L15|FDX1L|SUOX|PRC1|CIAPIN1|AKTIP|SOCS4|ULK2|MAPK8|CXCL12|NFATC2|PDXDC1|IRF9|BLOC1S5|SOCS6|MAP2K7|BCL2|TNFSF11|MAP3K1|MAPK4|TXNRD2|GATSL3|BCL6|IL6|LTBP1|ATG9A|CXCR4|MAP3K2|NFATC4|TLR6|IRAK4|PEX5|MAPKBP1|TMX1|EIF4E3|NKIRAS1|MAPK9|ITK|HLA-DMB|HLA-B|TNIK|NCOA2|ILF3|DOCK4|OXR1|LAMP2|MAP2K4|LMLN|PDCD6IP|TICAM2|TGFBI|ACKR2|PXT1|PEX5L|LAT2|DEFB4B|ATG12|SMAD1|TNFAIP8|IKBKB|API5|AMBRA1|ATG13|TRAF6|BCLAF1|DICER1|HIF1A|SMAD5|RASSF3|TNFAIP8L1|SOD2|APAF1|MAP3K12|MAPKAPK5|JKAMP|MAP3K9|MOAP1|TRAF3|NFAT5|LITAF|TXNIP|IKBKE|TNFRSF11A|IER2|ERC1|MAP2K6|CCL5|MR1|NOS1|ECSCR|JAKMIP2|SOCS2|TNFAIP3|SOCS7|PEX12|MAP3K14|GATSL2 | 77 | 451|532|203|351|562|447|469|350|280|529|285|339|117|175|265|552|301|277|243|150|444|432|477|140|551|275|543|489|159|386|258|516|219|462|505|496|461|353|550|142|518|506|235|498|458|427|147|127|195|200|194|362|165|364|322|345|420|141|189|126|334|490|436|208|377|365|111|366|225|191|187|398|281|237|247|422|406|204|311|215|361|155|156|113|408|482|409|497|369|503|260|152|279|337|136|302|296|272|112|232|228|222|209|122|210|292|372|207|385|519|158|273|183|418|226|176|439|213|504|133|375|538|146|196|293|416|560|347|319|149|248|396|460|182|246|220|241|360|468|507|124|525|454|470|542|188|254|392|528|438|119|437|269|160|245|332|335|380|216|389|464|299|443|212|178|190|376|487|139|268|184|423|521|349|278|312|318|343|358|271|338|400|434|323|399|116|179|177|137|404|394|440|520|239|240|474|230|342|563|238|109|368|264|352|326|276|300|510|407|206|129|459|499|169|346|309|263|465|480|297|555|223|466|314|316|234|320|512|262|511|472|424|523|214|282|181|115|330|492|198|244|236|475|430|397|231|476|288|310|218|154|257|450|374|249|305|371|514|298|390|403|417|250|556|553|114|535|455|110|544|144|304|517|333|307|329|157|173|363|328|467|166|421|193|185|526|463|131|559|315|180|121|495|201|221|453|381|148|426|370|382|290|138|557|393|395|415|548|163|354|401|255|135|540|508|253|251|419|164|186|229|171|402 | 2.43E-09 |  | GO.0005737 | 0.669897 | 0 |
| 88 | 15 | GO Process |  | transforming growth factor beta receptor signaling pathway | 2.03E-07 | TGFBR3|TGFB1|TGFB3|SMAD7|SMAD2|SMAD6|SMAD3|SMAD4|TGFBR2|SMURF1|TGFB2|MAP3K7|TGFBR1|SMAD1|SMAD5 | 77 | 447|350|265|195|200|369|241|470|332|464|376|358|368|193|201 | 1.16E-08 |  | GO.0007179 | 0.669250396 | 0 |
| 1554 | 75 | GO Component | | whole membrane | 2.05E-07 | TNFRSF1A|LAPTM4A|MAPK1|RHOQ|LAMP5|ATG14|TRAF2|BCL2L2|IRAK2|BCL2L10|MMD|MSR1|WDR59|SMURF2|ATF2|PECR|LAMP3|LY6K|WDR24|PEX13|TGFA|HPS6|TMX3|BCL2L1|IL7R|CFLAR|EEA1|LAMP1|MIOS|NECAP1|LAMTOR4|PEX2|TGFBR2|ATG2B|NOS1AP|TAB2|PEX3|PEX19|MCL1|ATG5|PEX11B|MAP3K7|IRAK1|BCL10|PI4K2A|TLR4|HLA-DQB1|TGFBR1|TNFRSF1B|TAB3|BNIP3L|JAK2|ATG16L1|BCL2L11|ULK2|BCL2|ATG9A|TLR6|IRAK4|PEX5|HLA-DMB|HLA-B|LAMP2|TICAM2|PEX5L|LAT2|ATG12|IKBKB|AMBRA1|TRAF6|MOAP1|LITAF|IKBKE|NOS1|PEX12 | 77 | 351|562|469|552|277|150|444|551|258|550|427|147|127|362|189|436|377|503|260|152|279|209|122|372|176|133|146|360|525|542|188|269|332|389|212|184|423|521|278|312|343|358|434|323|399|342|109|368|276|206|499|309|223|234|214|475|257|305|371|514|553|114|304|329|328|467|421|526|131|315|138|395|548|508|229 | 2.90E-09 |  | GO.0098805 | 0.668824614 | 0 |
| 151 | 19 | GO Process |  | regulation of T cell proliferation | 2.09E-07 | TGFB1|TNFSF9|ARG2|IL1B|TNFSF4|TNFRSF13C|TNFRSF21|IL12A|CTLA4|PAWR|TGFBR2|TNFSF13B|IL6ST|PDCD1LG2|IL6|HLA-DMB|IL10|TRAF6|CCL5 | 77 | 350|120|518|345|383|224|513|519|183|396|332|326|391|373|218|553|425|315|135 | 1.20E-08 |  | GO.0042129 | 0.667985371 | 0 |
| 131 | 17 | KEGG Pathways | | Hepatitis C | 2.16E-07 | TNFRSF1A|MAPK1|NFKB1|TRAF2|PIAS1|STAT3|SOCS3|IFNAR2|MAPK10|MAPK8|IRF9|PPARA|MAPK9|IKBKB|TRAF6|TRAF3|IKBKE | 77 | 351|469|529|444|554|334|246|452|380|282|492|491|250|526|315|557|548 | 4.72E-08 |  | hsa05160 | 0.666554625 | 0 |
| 11 | 8 | InterPro Domains | | DHR-2 domain | 2.19E-07 | DOCK7|DOCK10|DOCK3|DOCK11|DOCK5|DOCK1|DOCK9|DOCK4 | 77 | 413|505|225|422|406|215|300|544 | 4.06E-09 |  | IPR027357 | 0.665955589 | 0 |
| 11 | 8 | InterPro Domains | | DHR-1 domain | 2.19E-07 | DOCK7|DOCK10|DOCK3|DOCK11|DOCK5|DOCK1|DOCK9|DOCK4 | 77 | 413|505|225|422|406|215|300|544 | 4.06E-09 |  | IPR027007 | 0.665955589 | 0 |
| 11 | 8 | InterPro Domains | | Dedicator of cytokinesis | 2.19E-07 | DOCK7|DOCK10|DOCK3|DOCK11|DOCK5|DOCK1|DOCK9|DOCK4 | 77 | 413|505|225|422|406|215|300|544 | 4.06E-09 |  | IPR026791 | 0.665955589 | 0 |
| 11 | 8 | InterPro Domains | | Dedicator of cytokinesis, C-terminal | 2.19E-07 | DOCK7|DOCK10|DOCK3|DOCK11|DOCK5|DOCK1|DOCK9|DOCK4 | 77 | 413|505|225|422|406|215|300|544 | 4.06E-09 |  | IPR010703 | 0.665955589 | 0 |
| 2762 | 110 | GO Process |  | negative regulation of metabolic process | 2.19E-07 | TGFB1|NFKB1|CDKN1B|SOGA1|ATG14|LIF|PIAS1|MAP3K10|MSR1|SMAD7|SMAD2|SMURF2|IL1B|ATF2|STAT3|PRDX5|LAMP3|CDK6|CCAR1|IRF8|SOD1|TNFRSF10B|PDCD4|TNFSF4|DEPTOR|BAG4|SMAD6|TRAT1|SNIP1|CREBRF|HIF1AN|NFATC3|MAP2K1|BCL2L1|MAEA|IL13|SOCS5|PPARD|CFLAR|TNIP1|PRKRA|TGIF1|PAWR|SOCS1|PIWIL3|SOCS3|IKZF1|SMAD3|BCL11A|BAG5|SMAD4|RBPJ|ATG7|PEX2|FXR1|TGFB2|MAPKAPK2|TAB2|IL6R|IKZF5|MCL1|ATG5|BAG3|TNFRSF6B|BAG2|EXOSC1|LCOR|YWHAB|AGO3|AGO1|AGO4|TRAF3IP1|TGIF2|TLR4|TNFRSF1B|HIF3A|BCOR|TAB3|NFIB|LOXL2|EXOSC8|PDCD10|SOCS4|NFATC2|SOCS6|BCL2|BCL6|PPARA|IL6|NFATC4|NCOA2|ILF3|OXR1|IL10|IKZF2|SMAD1|TNFAIP8|IKBKB|TRAF6|BCLAF1|DICER1|HIF1A|SMAD5|NKRF|BCL7A|TXNIP|PANO|SOCS2|TNFAIP3|SOCS7 | 77 | 350|529|285|317|150|432|554|489|147|195|200|362|345|189|334|208|377|365|111|398|281|410|311|383|408|409|369|527|337|296|228|210|292|372|207|418|226|227|133|538|196|143|396|460|182|246|220|241|507|124|470|438|119|269|245|376|268|184|348|412|278|312|318|481|116|137|233|440|520|239|240|474|259|342|276|510|541|206|170|480|297|316|523|115|244|475|310|491|218|249|455|110|144|425|486|193|185|526|315|180|121|495|201|291|303|415|172|419|164|186 | 1.27E-08 |  | GO.0009892 | 0.665955589 | 0 |
| 75 | 14 | GO Process |  | positive regulation of receptor signaling pathway via STAT | 2.23E-07 | TNFRSF1A|LIF|STAT3|CSF1R|IL12A|IL13|IL7R|SOCS3|IL20|IL6R|IL6ST|JAK2|IL6|CCL5 | 77 | 351|432|334|153|519|418|176|246|161|348|391|309|218|135 | 1.30E-08 |  | GO.1904894 | 0.665169514 | 0 |
| 12 | 8 | SMART Domains | | Domain A in dwarfin family proteins | 2.23E-07 | SMAD7|SMAD2|SMAD6|SMAD3|SMAD4|NFIB|SMAD1|SMAD5 | 77 | 195|200|369|241|470|170|193|201 | 6.63E-09 |  | SM00523 | 0.665169514 | 0 |
| 26 | 10 | SMART Domains | | Tumor necrosis factor receptor / nerve growth factor receptor repeats. | 2.23E-07 | RELT|TNFRSF1A|TNFRSF10B|TNFRSF21|TNFRSF11B|TNFRSF6B|TNFRSF1B|TNFRSF19|TNFRSF11A|TNFRSF9 | 77 | 532|351|410|513|359|481|276|256|163|473 | 4.99E-09 |  | SM00208 | 0.665169514 | 0 |
| 149 | 18 | KEGG Pathways | | Non-alcoholic fatty liver disease (NAFLD) | 2.30E-07 | TNFRSF1A|TGFB1|NFKB1|TRAF2|NDUFA10|IL1A|IL1B|MAP3K11|SOCS3|MAP3K5|MAPK10|IL6R|BCL2L11|MAPK8|PPARA|IL6|MAPK9|IKBKB | 77 | 351|350|529|444|275|322|345|439|246|335|380|348|234|282|491|218|250|526 | 5.14E-08 |  | hsa04932 | 0.663827216 | 0 |
| 226 | 23 | GO Process |  | lymphocyte differentiation | 2.43E-07 | TGFB1|CCR7|DOCK10|IL7|IL11|STAT3|DOCK11|IL7R|IKZF1|GPR29|RBPJ|BCL11B|CCR9|THEMIS|ATG5|IRF4|BCL2|CMKBR6|BCL6|IL6|IL18R1|ITK|IL10 | 77 | 350|243|505|266|126|334|422|176|220|448|438|128|456|349|312|169|475|431|310|218|287|556|425 | 1.41E-08 |  | GO.0030098 | 0.661439373 | 0 |
| 3236 | 124 | UniProt Keywords | | Signal | 2.46E-07 | CX3CL1|RELT|TNFRSF1A|TGFBR3|TGFB1|CCL1|CCL13|CXCL6|CRTAM|LOX|IL1RL1|TGFB3|TMX4|LAMP5|CCR7|LIF|ISLR|LILRA1|PXDN|PDGFRA|CCL21|LOXL4|PDGFRB|LOXL1|DGCR2|IL7|IL11|LAMP3|ADAMTS17|IGSF6|IL22RA1|ILDR2|IL9|TNFRSF10B|ENDOD1|CSF1R|IL17RB|KIR3DL3|TGFA|FAM19A4|CXCL3|CXCL5|IL17RD|TNFRSF21|IL22RA2|TNFRSF11B|DEFB1|TMX3|LTBP3|IL12A|CTLA4|IL13|ALCAM|IL7R|CXCL11|ICOS|IGDCC4|TNFRSF12A|CSF1|IFNLR1|IL25|C1QTNF8|IGDCC3|LAMP1|BTLA|PDCD1|CXCL14|IL17F|IL21R|C1QTNF6|KIR2DL4|NCR3LG1|IFNAR2|TXNDC15|CCL20|TGFBR2|MPEG1|CCL28|GPX6|CXCL9|TGFB2|IL20|IL6R|IGSF3|IGSF1|IL1RAPL2|TLR4|IL2RG|HLA-DQB1|TGFBR1|TNFRSF1B|CCL7|TXNDC5|IL6ST|TNFRSF19|IL17RE|LOXL2|IGSF11|CXCL12|PDCD1LG2|FAM19A5|CSF2RB|IL6|LTBP1|IL1R1|IL18R1|TLR6|TMX1|HLA-DMB|HLA-B|LY6H|VSIG1|LAMP2|IL10|TGFBI|DEFB4B|IL22|TNFRSF11A|CCL5|MR1|CCL16|TNFRSF9|ECSCR|CCL4 | 77 | 325|532|351|447|350|484|485|531|151|168|336|265|301|277|243|432|477|344|543|516|500|242|506|498|405|266|126|377|267|546|211|237|295|410|204|153|497|442|279|545|356|355|136|513|272|359|112|122|441|519|183|418|286|176|321|167|457|340|149|341|478|471|261|360|449|537|468|367|217|123|515|313|452|494|308|332|379|488|388|283|376|161|348|270|132|252|342|563|109|368|276|289|459|391|256|465|480|130|181|373|327|524|218|154|274|287|305|390|553|114|357|378|304|425|157|166|534|163|135|540|501|473|253|118 | 9.72E-09 |  | KW-0732 | 0.660906489 | 0 |
| 514 | 36 | GO Process |  | negative regulation of intracellular signal transduction | 2.52E-07 | TNFAIP1|IL1RL1|LIF|BCL2L2|BCL2L10|IL1B|ACKR3|PDCD4|DEPTOR|BCL2L1|SOCS5|TNIP1|SOCS1|SOCS3|BAG5|SMAD4|TGFB2|MCL1|TLR4|TANK|SOCS4|CXCL12|SOCS6|BCL2|GATSL3|BCL6|MAPKBP1|HIF1A|TNFAIP8L1|SOD2|MAPKAPK5|LITAF|SOCS2|TNFAIP3|SOCS7|GATSL2 | 77 | 280|336|432|551|550|345|247|311|408|372|226|538|460|246|124|470|376|278|342|314|523|181|244|475|288|310|298|495|453|381|370|395|419|164|186|402 | 1.47E-08 |  | GO.1902532 | 0.659859946 | 0 |
| 171 | 20 | GO Process |  | positive regulation of apoptotic signaling pathway | 2.61E-07 | TNFSF10|TRAF2|SOD1|BCL2L1|PRKRA|TNFRSF12A|SMAD3|MCL1|BCL10|YWHAB|TGFBR1|JAK2|BCL2L11|MAPK8|BCL2|NFATC4|MAPK9|BCLAF1|APAF1|MOAP1 | 77 | 202|444|281|372|196|340|241|278|323|440|368|309|234|282|475|249|250|180|148|138 | 1.53E-08 |  | GO.2001235 | 0.658335949 | 0 |
| 12 | 8 | InterPro Domains | | MAD homology 1, Dwarfin-type | 2.62E-07 | SMAD7|SMAD2|SMAD6|SMAD3|SMAD4|NFIB|SMAD1|SMAD5 | 77 | 195|200|369|241|470|170|193|201 | 6.63E-09 |  | IPR003619 | 0.658169871 | 0 |
| 105 | 16 | GO Process |  | regulation of cellular response to transforming growth factor beta stimulus | 2.66E-07 | TGFBR3|TGFB1|TGFB3|SMAD7|SMAD2|SMURF2|SMAD6|CFLAR|SMAD3|BCL9L|IL17F|SMAD4|TGFBR2|SMURF1|TGFBR1|LTBP1 | 77 | 447|350|265|195|200|362|369|133|241|125|367|470|332|464|368|154 | 1.57E-08 |  | GO.1903844 | 0.657511836 | 0 |
| 4133 | 148 | GO Process |  | regulation of nucleobase-containing compound metabolic process | 2.67E-07 | TNFRSF1A|TGFBR3|MAPK1|TGFB1|TNFAIP1|NFKB1|CDKN1B|BCL9|TGFB3|RHOQ|RASL11A|TRAF2|LIF|PIAS1|MAP3K10|IRAK2|TBRG4|TRAF5|PDGFRB|SMAD7|SMAD2|SMURF2|NOX4|IL1A|IL1B|ATF2|IL11|STAT3|MAP3K13|PRDX5|CCAR1|IRF8|PDCD4|TNFSF4|AIFM1|GATAD1|SMAD6|TGFA|SNIP1|CREBRF|HIF1AN|NFATC3|MAP2K1|IL16|IL7R|PPARD|CFLAR|IL1RAP|TNIP1|C8orf4|NFATC2IP|TGIF1|PAWR|IL25|IKZF1|SMAD3|BCL9L|IL17F|BCL11A|SMAD4|IKZF3|MAP2K3|RBPJ|ATG7|PEX2|BCL11B|IRF5|MAP3K5|SMURF1|ILF2|IRF2BP2|TGFB2|IRF6|MAPKAPK2|TAB2|HIVEP2|GATAD2B|IKZF5|BAG3|MAP3K7|IRAK1BP1|IRAK1|BCL10|EXOSC1|IGSF1|LCOR|YWHAB|AGO1|CXCR3|TGIF2|TLR4|TGFBR1|TNFRSF1B|HIF3A|BCOR|TAB3|NFIB|IRF4|IL33|JAK2|LOXL2|EXOSC8|MAP3K4|NFATC2|IRF9|MAP2K7|TNFSF11|BCL6|PPARA|IL6|IL18R1|MAP3K2|NFATC4|TLR6|IRAK4|NCOA2|ILF3|MAP2K4|IL10|IKZF2|SMAD1|IKBKB|TRAF6|BCLAF1|DICER1|BCORL1|HIF1A|SMAD5|NKRF|TET2|BCL7A|SOD2|APAF1|MAPKAPK5|MAP3K9|TRAF3|NFAT5|LITAF|TXNIP|TNFRSF11A|IER2|PIAS2|ERC1|MAP2K6|CCL5|NOS1|TNFAIP3|CCL4 | 77 | 351|447|469|350|280|529|285|175|265|552|205|444|432|554|489|258|496|142|506|195|200|362|364|322|345|189|126|334|490|208|111|398|311|383|482|530|369|279|337|296|228|210|292|385|176|227|133|199|538|293|347|143|396|478|220|241|125|367|507|470|392|528|438|119|269|128|160|335|464|443|178|376|487|268|184|433|414|412|318|358|400|434|323|137|132|233|440|239|230|259|342|368|276|510|541|206|170|169|346|309|480|297|466|115|492|236|430|310|491|218|287|374|249|305|371|455|110|517|425|486|193|526|315|180|121|387|495|201|291|294|303|381|148|370|290|557|393|395|415|163|354|284|401|255|135|508|164|118 | 1.58E-08 |  | GO.0019219 | 0.657348874 | 0 |
| 76 | 14 | Reactome Pathways | | DDX58/IFIH1-mediated induction of interferon-alpha/beta | 2.81E-07 | NFKB1|TRAF2|NKIRAS2|ATG5|SIKE1|TANK|MAP3K1|NKIRAS1|ATG12|IKBKB|TRAF6|TRAF3|IKBKE|TNFAIP3 | 77 | 529|444|273|312|338|314|397|417|421|526|315|557|548|164 | 1.50E-08 |  | HSA-168928 | 0.655129368 | 0 |
| 910 | 51 | GO Process |  | negative regulation of developmental process | 2.82E-07 | MAPK1|TGFB1|CDKN1B|SMAD7|ATF2|STAT3|CDK6|PDCD4|TNFSF4|SMAD6|TNFRSF21|TNFRSF11B|NFATC3|CTLA4|IL13|SOCS5|PPARD|CFLAR|SOCS1|SMAD3|PDCD1|CXCL14|IL17F|BCL11A|BAG5|SMAD4|MAP4K4|RBPJ|TGFBR2|MAP3K3|TGFB2|AGO1|TRAF3IP1|CXCR3|TLR4|TGFBR1|BCOR|NFIB|LOXL2|PDCD10|ULK2|NFATC2|BCL2|BCL6|PPARA|IL6|NFATC4|DICER1|HIF1A|ECSCR|SOCS2 | 77 | 469|350|285|195|189|334|365|311|383|369|513|359|210|183|418|226|227|133|460|241|537|468|367|507|124|470|254|438|332|299|376|239|474|230|342|368|541|170|480|316|214|115|475|310|491|218|249|121|495|253|419 | 1.67E-08 |  | GO.0051093 | 0.654975089 | 0 |
| 422 | 32 | GO Process |  | regulation of protein secretion | 2.89E-07 | TGFB1|CRTAM|IL1RL1|TGFB3|CCR7|TRAF2|ARG2|IL1A|IL1B|TNFSF4|CSF1R|IL17RB|TNFRSF21|IL13|PPARD|IL1RAP|IL17F|MAP4K4|TGFB2|ATG5|TLR4|IL33|JAK2|IL36RN|IL6|TLR6|MAPKBP1|IL10|TRAF6|HIF1A|CCL5|TNFRSF9 | 77 | 350|151|336|265|243|444|518|322|345|383|153|497|513|418|227|199|367|254|376|312|342|346|309|536|218|305|298|425|315|495|135|473 | 1.72E-08 |  | GO.0050708 | 0.653910216 | 0 |
| 2605 | 105 | Reactome Pathways | | Signal Transduction | 3.04E-07 | CX3CL1|NOX3|TNFRSF1A|MAPK1|TGFB1|CCL1|CCL13|CXCL6|NFKB1|CDKN1B|IL1RL1|BCL9|RHOQ|TNFSF10|CCR7|TRAF2|DOCK7|PDGFRA|CCL21|IER3|PDGFRB|MAPK6|SMAD7|SMAD2|SMURF2|ATF2|STAT3|DOCK3|ACKR3|TNFRSF10B|DOCK1|BAG4|SMAD6|CXCR5|TGFA|TRAT1|CXCL3|CXCL5|IL17RD|RICTOR|AVPR1A|MAP2K1|CXCL11|MAP3K11|PPARD|CFLAR|RHOJ|TGIF1|SOCS1|SOCS3|CCR4|SMAD3|BCL9L|SMAD4|LAMTOR4|RBPJ|CCRL2|CCR9|CCL20|TGFBR2|CCL28|SMURF1|CXCL9|TAGAP|MAPKAPK2|TAB2|IL6R|GATAD2B|MAP3K7|IRAK1|YWHAB|AGO3|AGO1|AGO4|CXCR3|TGIF2|IL2RG|TGFBR1|TAB3|IL33|JAK2|BCL2L11|PRC1|MAPK8|CXCL12|SOCS6|BCL2|MAPK4|CMKBR6|CSF2RB|IL6|CXCR4|IRAK4|CXCR6|NCOA2|SMAD1|IKBKB|TRAF6|HIF1A|SMAD5|MAPKAPK5|CCL5|CCL16|TNFAIP3|CCL4 | 77 | 325|203|351|469|350|484|485|531|529|285|336|175|552|202|243|444|413|516|500|461|506|235|195|200|362|189|334|225|247|410|215|409|369|306|279|527|356|355|136|302|222|292|321|439|227|133|375|143|460|246|502|241|125|470|188|438|134|456|308|332|488|464|283|139|268|184|348|414|358|434|440|520|239|240|230|259|563|368|206|346|309|234|511|282|181|244|475|231|431|524|218|450|371|435|455|193|526|315|495|201|370|135|501|164|118 | 1.67E-08 |  | HSA-162582 | 0.651712642 | 0 |
| 52 | 12 | GO Process |  | autophagosome assembly | 3.11E-07 | ATG14|ATG3|ATG4D|ATG7|ATG2B|SMURF1|ATG5|ATG16L1|ATG9A|ATG12|AMBRA1|ATG13 | 77 | 150|113|213|119|389|464|312|223|257|421|131|559 | 1.85E-08 |  | GO.0000045 | 0.650723961 | 0 |
| 31 | 10 | GO Process |  | extrinsic apoptotic signaling pathway in absence of ligand | 3.32E-07 | BCL2L2|BCL2L10|IL1A|IL1B|BCL2L1|MCL1|BAG3|BCL2L11|BCL2|MOAP1 | 77 | 551|550|322|345|372|278|318|234|475|138 | 1.99E-08 |  | GO.0097192 | 0.647886192 | 0 |
| 78 | 14 | GO Process |  | cellular response to mechanical stimulus | 3.34E-07 | TNFRSF1A|TGFB1|NFKB1|IL1B|TNFRSF10B|IL13|BAG3|BCL10|TLR4|MAPK8|MAP3K1|MAP3K2|MAP2K4|MAP3K14 | 77 | 351|350|529|345|410|418|318|323|342|282|397|374|517|171 | 2.01E-08 |  | GO.0071260 | 0.647625353 | 0 |
| 88 | 14 | KEGG Pathways | | Th1 and Th2 cell differentiation | 3.38E-07 | MAPK1|NFKB1|NFATC3|IL12A|IL13|RBPJ|MAPK10|IL2RG|JAK2|MAPK8|NFATC2|MAPK9|HLA-DMB|IKBKB | 77 | 469|529|210|519|418|438|380|563|309|282|115|250|553|526 | 7.71E-08 |  | hsa04658 | 0.64710833 | 0 |
| 622 | 40 | GO Process |  | regulation of protein transport | 3.49E-07 | MAPK1|TGFB1|CRTAM|IL1RL1|TGFB3|CCR7|TRAF2|XPO4|ARG2|IL1A|IL1B|TNFSF4|CSF1R|BAG4|IL17RB|TNFRSF21|CREBRF|IL13|PPARD|IL1RAP|SMAD3|IL17F|MAP4K4|TGFB2|ATG5|BAG3|TLR4|BNIP3L|IL33|JAK2|PDCD10|IL36RN|IL6|TLR6|MAPKBP1|IL10|TRAF6|HIF1A|CCL5|TNFRSF9 | 77 | 469|350|151|336|265|243|444|386|518|322|345|383|153|409|497|513|296|418|227|199|241|367|254|376|312|318|342|499|346|309|316|536|218|305|298|425|315|495|135|473 | 2.11E-08 |  | GO.0051223 | 0.645717457 | 0 |
| 252 | 24 | GO Process |  | humoral immune response | 3.66E-07 | CCL1|CCL13|CXCL6|CCL21|IL7|CXCL3|TNFRSF21|DEFB1|DEFB4A|CXCL11|PDCD1|CXCL14|GPR29|RBPJ|CCL20|CCL28|CXCL9|HLA-DQB1|IL36RN|CXCL12|BCL2|CMKBR6|IL6|DEFB4B | 77 | 484|485|531|500|266|356|513|112|158|321|537|468|448|438|308|488|283|109|536|181|475|431|218|166 | 2.22E-08 |  | GO.0006959 | 0.643651891 | 0 |
| 93 | 15 | GO Process |  | positive regulation of adaptive immune response | 3.70E-07 | TGFB1|TRAF2|IL1B|TNFSF4|TNFRSF13C|IL12A|SOCS5|MAP3K7|TNFSF13B|IL6ST|IL6|IL1R1|IL18R1|HLA-B|TRAF6 | 77 | 350|444|345|383|224|519|226|358|326|391|218|274|287|114|315 | 2.25E-08 |  | GO.0002821 | 0.643179828 | 0 |
| 4194 | 149 | GO Process |  | protein metabolic process | 3.77E-07 | MAP4K5|TGFBR3|MAPK1|TGFB1|TNFAIP1|NFKB1|CDKN1B|LOX|NKTR|TRAF2|LIF|PIAS1|MAP3K10|IRAK2|PDGFRA|TBRG4|PDGFRB|MAPK6|LOXL1|FAF2|MMD|SMAD7|SMAD2|SMURF2|MAP3K8|IL1B|MAP4K3|ATF2|MAP3K13|CDK6|ADAMTS17|ATG10|ATG3|CSF1R|PEX13|IL17RD|RICTOR|HIF1AN|TMX3|MAP2K1|MAEA|SOCS5|MAP3K11|PPARD|ATG4D|OXSR1|CFLAR|IL1RAP|TNIP1|PRKRA|AATK|NFATC2IP|CSF1|PAWR|SOCS1|SOCS3|SMAD3|IL17F|BCL11A|SMAD4|MAP4K4|MAP2K3|MAP3K15|ATG7|AREL1|PEX2|TGFBR2|MAP3K5|MAPK10|SMURF1|MAP3K3|KIAA1804|TGFB2|MAPKAPK2|TAB2|ATG5|CACUL1|MAP3K7|IRAK1|BCL10|BAG2|YWHAB|TLR4|IL2RG|TGFBR1|ZAK|MAP3K19|HIF3A|BCOR|TAB3|BNIP3L|IRF4|IL33|JAK2|TNFRSF19|LOXL2|ATG16L1|MAP3K4|TANK|AKTIP|SOCS4|ULK2|MAPK8|SOCS6|MAP2K7|BCL2|TNFSF11|MAP3K1|MAPK4|CSF2RB|PPARA|IL6|LTBP1|MAP3K2|IRAK4|PEX5|EIF4E3|MAPK9|ITK|TNIK|ILF3|LAMP2|MAP2K4|LMLN|PDCD6IP|TGFBI|ATG12|SMAD1|IKBKB|TRAF6|HIF1A|SMAD5|TET2|MAP3K12|MAPKAPK5|JKAMP|MAP3K9|TRAF3|IKBKE|PIAS2|ERC1|MAP2K6|CCL5|NOS1|SOCS2|TNFAIP3|SOCS7|PEX12|MAP3K14 | 77 | 451|447|469|350|280|529|285|168|117|444|432|554|489|258|516|496|506|235|498|458|427|195|200|362|165|345|141|189|490|365|267|155|113|153|152|136|302|228|122|292|207|226|439|227|213|504|133|199|538|196|560|347|149|396|460|246|241|367|507|470|254|528|384|119|437|269|332|335|380|464|299|190|376|268|184|312|522|358|434|323|116|440|342|563|368|264|352|510|541|206|499|169|346|309|256|480|223|466|314|424|523|214|282|244|236|475|430|397|231|524|491|218|154|374|371|514|403|250|556|535|110|304|517|333|307|157|421|193|526|315|495|201|294|426|370|382|290|557|548|284|401|255|135|508|419|164|186|229|171 | 2.30E-08 |  | GO.0019538 | 0.642365865 | 0 |
| 158 | 19 | GO Process |  | intrinsic apoptotic signaling pathway | 3.83E-07 | TNFRSF1A|TRAF2|BCL2L2|BCL2L10|TNFRSF10B|AIFM1|BCL2L1|AEN|MAP3K5|MCL1|TNFRSF1B|JAK2|PDCD10|BCL2L11|BCL2|NFATC4|APAF1|MOAP1|IKBKE | 77 | 351|444|551|550|410|482|372|547|335|278|276|309|316|234|475|249|148|138|548 | 2.34E-08 |  | GO.0097193 | 0.641680123 | 0 |
| 452 | 33 | GO Process |  | regulation of peptide secretion | 3.90E-07 | TGFB1|CRTAM|IL1RL1|TGFB3|CCR7|TRAF2|ARG2|IL1A|IL1B|TNFSF4|CSF1R|IL17RB|TNFRSF21|IL13|PPARD|IL1RAP|IL17F|MAP4K4|TGFB2|ATG5|TLR4|IL33|JAK2|IL36RN|TNFSF11|IL6|TLR6|MAPKBP1|IL10|TRAF6|HIF1A|CCL5|TNFRSF9 | 77 | 350|151|336|265|243|444|518|322|345|383|153|497|513|418|227|199|367|254|376|312|342|346|309|536|430|218|305|298|425|315|495|135|473 | 2.39E-08 |  | GO.0002791 | 0.640893539 | 0 |
| 31 | 10 | GO Function | | tumor necrosis factor receptor binding | 3.99E-07 | TNFSF10|TNFSF9|TRAF2|TRAF5|TRAF4|TNFSF4|TNFSF13B|TNFSF11|TRAF6|TRAF3 | 77 | 202|120|444|142|194|383|326|430|315|557 | 1.99E-08 |  | GO.0005164 | 0.63990271 | 0 |
| 272 | 25 | GO Function | | G protein-coupled receptor binding | 4.19E-07 | CX3CL1|CCL1|CCL13|CXCL6|CCL21|STAT3|CXCL3|CXCL5|DEFB1|AVPR1A|DEFB4A|CXCL11|CXCL14|CCRL2|CCL20|CCL28|CXCL9|CCL7|JAK2|CXCL12|PDCD6IP|DEFB4B|CCL5|CCL16|CCL4 | 77 | 325|484|485|531|500|334|356|355|112|222|158|321|468|134|308|488|283|289|309|181|307|166|135|501|118 | 2.15E-08 |  | GO.0001664 | 0.637778598 | 0 |
| 196 | 21 | GO Process |  | positive regulation of ERK1 and ERK2 cascade | 4.38E-07 | CX3CL1|TGFB1|CCL1|CCL13|CCR7|PDGFRA|CCL21|PDGFRB|NOX4|ACKR3|CSF1R|MAP2K1|CFLAR|TNFAIP8L3|CCL20|CCL7|TNFSF11|TNFRSF11A|CCL5|CCL16|CCL4 | 77 | 325|350|484|485|243|516|500|506|364|247|153|292|133|248|308|289|430|163|135|501|118 | 2.69E-08 |  | GO.0070374 | 0.635852589 | 0 |
| 654 | 41 | GO Process |  | regulation of peptide transport | 4.40E-07 | MAPK1|TGFB1|CRTAM|IL1RL1|TGFB3|CCR7|TRAF2|XPO4|ARG2|IL1A|IL1B|TNFSF4|CSF1R|BAG4|IL17RB|TNFRSF21|CREBRF|IL13|PPARD|IL1RAP|SMAD3|IL17F|MAP4K4|TGFB2|ATG5|BAG3|TLR4|BNIP3L|IL33|JAK2|PDCD10|IL36RN|TNFSF11|IL6|TLR6|MAPKBP1|IL10|TRAF6|HIF1A|CCL5|TNFRSF9 | 77 | 469|350|151|336|265|243|444|386|518|322|345|383|153|409|497|513|296|418|227|199|241|367|254|376|312|318|342|499|346|309|316|536|430|218|305|298|425|315|495|135|473 | 2.71E-08 |  | GO.0090087 | 0.635654732 | 0 |
| 8 | 7 | SMART Domains | | Domain B in dwarfin family proteins | 4.41E-07 | SMAD7|SMAD2|SMAD6|SMAD3|SMAD4|SMAD1|SMAD5 | 77 | 195|200|369|241|470|193|201 | 1.64E-08 |  | SM00524 | 0.635556141 | 0 |
| 32 | 10 | Reactome Pathways | | TAK1 activates NFkB by phosphorylation and activation of IKKs complex | 4.53E-07 | NFKB1|IRAK2|NKIRAS2|TAB2|MAP3K7|IRAK1|TAB3|NKIRAS1|IKBKB|TRAF6 | 77 | 529|258|273|184|358|434|206|417|526|315 | 2.56E-08 |  | HSA-445989 | 0.63439018 | 0 |
| 23 | 9 | Reactome Pathways | | activated TAK1 mediates p38 MAPK activation | 4.55E-07 | IRAK2|MAP2K3|MAPKAPK2|TAB2|MAP3K7|IRAK1|TAB3|TRAF6|MAP2K6 | 77 | 258|528|268|184|358|434|206|315|255 | 2.64E-08 |  | HSA-450302 | 0.63419886 | 0 |
| 126 | 17 | GO Process |  | Fc receptor signaling pathway | 4.56E-07 | MAPK1|NFKB1|DOCK1|NFATC3|MAPK10|TAB2|MAP3K7|BCL10|TAB3|MAPK8|NFATC2|MAP3K1|MAPK9|ITK|LAT2|IKBKB|TRAF6 | 77 | 469|529|215|210|380|184|358|323|206|282|115|397|250|556|467|526|315 | 2.82E-08 |  | GO.0038093 | 0.634103516 | 0 |
| 1483 | 70 | GO Function | | molecular transducer activity | 4.75E-07 | RELT|TNFRSF1A|TGFBR3|CDKN1B|IL1RL1|CCR7|LILRA1|PDGFRA|PDGFRB|MMD|SMAD7|SMAD2|STAT3|CDK6|IGSF6|IL22RA1|ACKR3|TNFRSF10B|CSF1R|IL17RB|SMAD6|CXCR5|IL17RD|TNFRSF21|IL22RA2|TNFRSF11B|AVPR1A|IL7R|PPARD|IL1RAP|IFNLR1|CCR4|SMAD3|IL21R|KIR2DL4|SMAD4|GPR29|IFNAR2|CCRL2|CCR9|TGFBR2|IL6R|TNFRSF6B|IGSF1|IL1RAPL2|CXCR3|TLR4|IL2RG|HLA-DQB1|TGFBR1|TNFRSF1B|IL6ST|TNFRSF19|IL17RE|IL17REL|CMKBR6|CSF2RB|PPARA|LTBP1|IL1R1|CXCR4|IL18R1|TLR6|CXCR6|ACKR2|SMAD1|SMAD5|TNFRSF11A|MR1|TNFRSF9 | 77 | 532|351|447|285|336|243|344|516|506|427|195|200|334|365|546|211|247|410|153|497|369|306|136|513|272|359|222|176|227|199|341|502|241|217|515|470|448|452|134|456|332|348|481|132|252|230|342|563|109|368|276|391|256|465|479|431|524|491|154|274|450|287|305|435|173|193|201|163|540|473 | 2.51E-08 |  | GO.0060089 | 0.632330639 | 0 |
| 298 | 26 | GO Process |  | response to decreased oxygen levels | 4.80E-07 | TGFBR3|TGFB1|CDKN1B|TGFB3|HILPDA|NOX4|AIFM1|HIF1AN|PPARD|CFLAR|SMAD3|SMAD4|RBPJ|TGFBR2|TGFB2|IRAK1|HIF3A|BNIP3L|LOXL2|CXCL12|BCL2|PPARA|CXCR4|HIF1A|APAF1|NOS1 | 77 | 447|350|285|265|462|364|482|228|227|133|241|470|438|332|376|434|510|499|480|181|475|491|450|495|148|508 | 2.98E-08 |  | GO.0036293 | 0.631875876 | 0 |
| 1491 | 70 | GO Process |  | homeostatic process | 4.93E-07 | NOX3|TGFBR3|MAPK1|TGFB1|CCL1|CCL13|TMX4|CCR7|PDGFRA|DOCK10|CCL21|SMAD7|NOX4|IL1A|IL1B|IL7|STAT3|PRDX5|SOD1|ILDR2|DOCK11|TXNDC11|TNFRSF13C|AVPR1A|TMX3|MAEA|IL13|IL7R|CXCL11|CSF1|IKZF1|CCR4|SMAD3|SMAD4|GPR29|CCR9|TXNDC15|CCL28|CXCL9|MCL1|ATG5|CXCR3|TLR4|TXNDC8|TNFSF13B|CCL7|TXNDC5|JAK2|TXNRD3|BCL2L11|CXCL12|BCL2|TNFSF11|TXNRD2|CMKBR6|BCL6|IL6|CXCR4|TMX1|VSIG1|LAMP2|SMAD1|TRAF6|HIF1A|SMAD5|SOD2|TNFRSF11A|CCL5|NOS1|TNFAIP3 | 77 | 203|447|469|350|484|485|301|243|516|505|500|195|364|322|345|266|334|208|281|237|422|156|224|222|122|207|418|176|321|149|220|502|241|470|448|456|494|488|283|278|312|230|342|238|326|289|459|309|263|234|181|475|430|476|431|310|218|450|390|378|304|193|315|495|201|381|163|135|508|164 | 3.08E-08 |  | GO.0042592 | 0.630715308 | 0 |
| 237 | 23 | GO Process |  | regulation of peptidyl-tyrosine phosphorylation | 5.07E-07 | TNFRSF1A|TGFB1|LIF|NOX4|IL11|STAT3|DOCK3|CSF1R|TGFA|RICTOR|IL22RA2|IL12A|IL13|SOCS5|SOCS1|SOCS3|IL20|IL6R|IL6ST|JAK2|SOCS4|IL6|CCL5 | 77 | 351|350|432|364|126|334|225|153|279|302|272|519|418|226|460|246|161|348|391|309|523|218|135 | 3.18E-08 |  | GO.0050730 | 0.629499204 | 0 |
| 68 | 13 | GO Process |  | neutrophil migration | 5.32E-07 | CX3CL1|CCL1|CCL13|CXCL6|IL1B|CXCL3|CCL20|TGFB2|CCL7|IRAK4|CCL5|CCL16|CCL4 | 77 | 325|484|485|531|345|356|308|376|289|371|135|501|118 | 3.35E-08 |  | GO.1990266 | 0.627408837 | 0 |
| 97 | 15 | GO Process |  | regulation of NIK/NF-kappaB signaling | 5.86E-07 | TRAF2|TRAF4|IL1B|TNFRSF10B|PDCD4|C8orf4|MAP3K7|IRAK1|AGO3|AGO1|TLR4|IL18R1|TLR6|TRAF6|LITAF | 77 | 444|194|345|410|311|293|358|434|520|239|342|287|305|315|395 | 3.71E-08 |  | GO.1901222 | 0.623210238 | 0 |
| 97 | 15 | GO Process |  | regulation of interferon-gamma production | 5.86E-07 | IL1RL1|CCR7|IL1B|IRF8|TNFSF4|TNFRSF13C|IL12A|TLR4|IL33|IL36RN|PDCD1LG2|IL1R1|IL18R1|IL10|TICAM2 | 77 | 336|243|345|398|383|224|519|342|346|536|373|274|287|425|329 | 3.71E-08 |  | GO.0032649 | 0.623210238 | 0 |
| 16 | 8 | Reactome Pathways | | TNF receptor superfamily (TNFSF) members mediating non-canonical NF-kB pathway | 6.03E-07 | TRAF2|TNFRSF13C|TNFRSF12A|TNFSF13B|TNFSF11|TRAF3|TNFRSF11A|MAP3K14 | 77 | 444|224|340|326|430|557|163|171 | 3.58E-08 |  | HSA-5676594 | 0.621968269 | 0 |
| 8 | 7 | InterPro Domains | | SMAD MH1 domain superfamily | 6.08E-07 | SMAD7|SMAD2|SMAD6|SMAD3|SMAD4|SMAD1|SMAD5 | 77 | 195|200|369|241|470|193|201 | 1.64E-08 |  | IPR036578 | 0.621609642 | 0 |
| 8 | 7 | InterPro Domains | | Dwarfin | 6.08E-07 | SMAD7|SMAD2|SMAD6|SMAD3|SMAD4|SMAD1|SMAD5 | 77 | 195|200|369|241|470|193|201 | 1.64E-08 |  | IPR013790 | 0.621609642 | 0 |
| 8 | 7 | InterPro Domains | | MAD homology, MH1 | 6.08E-07 | SMAD7|SMAD2|SMAD6|SMAD3|SMAD4|SMAD1|SMAD5 | 77 | 195|200|369|241|470|193|201 | 1.64E-08 |  | IPR013019 | 0.621609642 | 0 |
| 469 | 34 | InterPro Domains | | Immunoglobulin-like domain | 6.08E-07 | CRTAM|IL1RL1|ISLR|LILRA1|PXDN|PDGFRA|PDGFRB|IGSF6|ILDR2|CSF1R|KIR3DL3|CTLA4|ALCAM|IL1RAP|ICOS|IGDCC4|IGDCC3|BTLA|PDCD1|KIR2DL4|NCR3LG1|IL6R|IGSF3|IGSF1|IL1RAPL2|HLA-DQB1|IGSF11|PDCD1LG2|IL1R1|IL18R1|HLA-DMB|HLA-B|VSIG1|MR1 | 77 | 151|336|477|344|543|516|506|546|237|153|442|183|286|199|167|457|261|449|537|515|313|348|270|132|252|109|130|373|274|287|553|114|378|540 | 1.71E-08 |  | IPR007110 | 0.621609642 | 0 |
| 8 | 7 | InterPro Domains | | SMAD domain, Dwarfin-type | 6.08E-07 | SMAD7|SMAD2|SMAD6|SMAD3|SMAD4|SMAD1|SMAD5 | 77 | 195|200|369|241|470|193|201 | 1.64E-08 |  | IPR001132 | 0.621609642 | 0 |
| 182 | 20 | GO Process |  | modification of morphology or physiology of other organism | 6.20E-07 | TGFB1|CCL1|CCL13|CCL21|CXCL3|BCL2L1|DEFB4A|CXCL11|TNIP1|SMAD3|CXCL14|ATG7|CCL20|CCL28|CXCL9|BCL2L11|CXCL12|DEFB4B|CCL5|CCL4 | 77 | 350|484|485|500|356|372|158|321|538|241|468|119|308|488|283|234|181|166|135|118 | 3.94E-08 |  | GO.0035821 | 0.620760831 | 0 |
| 261 | 24 | GO Process |  | regulation of leukocyte differentiation | 6.43E-07 | TGFB1|TNFSF9|LIF|SMAD7|IL7|CDK6|SOD1|TNFSF4|IL12A|CTLA4|SOCS5|IL7R|CSF1|SOCS1|IKZF3|TGFBR2|IL20|TLR4|IRF4|NFATC2|TNFSF11|BCL6|HLA-B|TRAF6 | 77 | 350|120|432|195|266|365|281|383|519|183|226|176|149|460|392|332|161|342|169|115|430|310|114|315 | 4.10E-08 |  | GO.1902105 | 0.619178903 | 0 |
| 45 | 11 | GO Process |  | positive regulation of epithelial to mesenchymal transition | 6.86E-07 | TGFB1|TGFB3|SMAD2|IL1B|SMAD3|BCL9L|SMAD4|TGFBR2|TGFB2|TGFBR1|LOXL2 | 77 | 350|265|200|345|241|125|470|332|376|368|480 | 4.39E-08 |  | GO.0010718 | 0.616367588 | 0 |
| 222 | 22 | GO Process |  | cellular response to oxidative stress | 7.03E-07 | MAPK1|TRAF2|PDGFRA|NOX4|PRDX5|SOD1|AIFM1|CFLAR|PRKRA|MAP3K5|TXNDC8|JAK2|PDCD10|MAPK8|BCL2|TXNRD2|IL6|MAPK9|OXR1|HIF1A|SOD2|TNFAIP3 | 77 | 469|444|516|364|208|281|482|133|196|335|238|309|316|282|475|476|218|250|144|495|381|164 | 4.51E-08 |  | GO.0034599 | 0.615304467 | 0 |
| 70 | 13 | GO Process |  | negative regulation of transforming growth factor beta receptor signaling pathway | 7.04E-07 | TGFBR3|TGFB1|TGFB3|SMAD7|SMAD2|SMURF2|SMAD6|SMAD3|BCL9L|TGFBR2|SMURF1|TGFBR1|LTBP1 | 77 | 447|350|265|195|200|362|369|241|125|332|464|368|154 | 4.53E-08 |  | GO.0030512 | 0.615242734 | 0 |
| 305 | 26 | GO Process |  | regulation of vasculature development | 7.10E-07 | CX3CL1|IL1A|IL1B|ATF2|STAT3|PDCD4|HIF1AN|NFATC3|CFLAR|RHOJ|TNFRSF12A|IL17F|TGFBR2|MAP3K3|TGFB2|AGO1|CXCR3|PDCD10|NFATC2|IL6|IL10|SMAD1|HIF1A|SOD2|ECSCR|TNFAIP3 | 77 | 325|322|345|189|334|311|228|210|133|375|340|367|332|299|376|239|230|316|115|218|425|193|495|381|253|164 | 4.59E-08 |  | GO.1901342 | 0.614874165 | 0 |
| 373 | 29 | GO Process |  | response to oxidative stress | 7.64E-07 | MAPK1|CCR7|TRAF2|PXDN|PDGFRA|PDGFRB|NOX4|PRDX5|SOD1|AIFM1|OXSR1|CFLAR|PRKRA|MAP3K5|GPX6|TXNDC8|JAK2|TXNRD3|PDCD10|MAPK8|BCL2|TXNRD2|IL6|MAPK9|OXR1|HIF1A|SOD2|TXNIP|TNFAIP3 | 77 | 469|243|444|543|516|506|364|208|281|482|504|133|196|335|388|238|309|263|316|282|475|476|218|250|144|495|381|415|164 | 4.95E-08 |  | GO.0006979 | 0.611690664 | 0 |
| 35 | 10 | GO Process |  | NIK/NF-kappaB signaling | 8.03E-07 | NFKB1|TRAF2|TRAF4|TNFRSF10B|MAP3K7|IRAK1|TLR6|TRAF6|IKBKE|MAP3K14 | 77 | 529|444|194|410|358|434|305|315|548|171 | 5.21E-08 |  | GO.0038061 | 0.609528445 | 0 |
| 58 | 12 | GO Process |  | regulation of T cell mediated immunity | 8.14E-07 | TRAF2|SMAD7|IL1B|TNFSF4|IL12A|IL7R|MAP3K7|IL6|IL1R1|IL18R1|HLA-B|TRAF6 | 77 | 444|195|345|383|519|176|358|218|274|287|114|315 | 5.30E-08 |  | GO.0002709 | 0.60893756 | 0 |
| 807 | 46 | GO Process |  | circulatory system development | 8.49E-07 | CX3CL1|TNFRSF1A|TGFBR3|MAPK1|TGFB1|LOX|LIF|PDGFRA|PDGFRB|LOXL1|SMAD7|SMAD2|NOX4|ATF2|ACKR3|PDCD4|SMAD6|MAP2K1|PPARD|RHOJ|TNFRSF12A|SOCS3|SMAD3|SMAD4|RBPJ|TGFBR2|MAP3K3|TGFB2|TAB2|ATG5|TRAF3IP1|CXCR3|TGFBR1|TNFRSF1B|HIF3A|BCOR|LOXL2|PDCD10|PPARA|LTBP1|NFATC4|MAP2K4|TGFBI|SMAD1|HIF1A|ECSCR | 77 | 325|351|447|469|350|168|432|516|506|498|195|200|364|189|247|311|369|292|227|375|340|246|241|470|438|332|299|376|184|312|474|230|368|276|510|541|480|316|491|154|249|517|157|193|495|253 | 5.55E-08 |  | GO.0072359 | 0.607109231 | 0 |
| 133 | 17 | GO Process |  | regulation of lymphocyte mediated immunity | 8.80E-07 | TGFB1|CRTAM|TRAF2|SMAD7|IL1B|TNFSF4|IL12A|IL7R|LAMP1|MAP3K7|BCL6|IL6|IL1R1|IL18R1|HLA-B|IL10|TRAF6 | 77 | 350|151|444|195|345|383|519|176|360|358|310|218|274|287|114|425|315 | 5.77E-08 |  | GO.0002706 | 0.605551733 | 0 |
| 288 | 25 | GO Process |  | response to hypoxia | 9.19E-07 | TGFBR3|TGFB1|CDKN1B|TGFB3|HILPDA|NOX4|HIF1AN|PPARD|CFLAR|SMAD3|SMAD4|RBPJ|TGFBR2|TGFB2|IRAK1|HIF3A|BNIP3L|LOXL2|CXCL12|BCL2|PPARA|CXCR4|HIF1A|APAF1|NOS1 | 77 | 447|350|285|265|462|364|228|227|133|241|470|438|332|376|434|510|499|480|181|475|491|450|495|148|508 | 6.04E-08 |  | GO.0001666 | 0.603668449 | 0 |
| 59 | 12 | GO Process |  | neutrophil chemotaxis | 9.48E-07 | CX3CL1|CCL1|CCL13|CXCL6|IL1B|CXCL3|CCL20|TGFB2|CCL7|CCL5|CCL16|CCL4 | 77 | 325|484|485|531|345|356|308|376|289|135|501|118 | 6.25E-08 |  | GO.0030593 | 0.602319166 | 0 |
| 46 | 11 | GO Function | | tumor necrosis factor receptor superfamily binding | 9.79E-07 | TNFSF10|TNFSF9|TRAF2|TRAF5|TRAF4|TNFSF4|CFLAR|TNFSF13B|TNFSF11|TRAF6|TRAF3 | 77 | 202|120|444|142|194|383|133|326|430|315|557 | 5.33E-08 |  | GO.0032813 | 0.600921731 | 0 |
| 2381 | 97 | UniProt Keywords | | Ubl conjugation | 9.82E-07 | MAPK1|NFKB1|CDKN1B|NKTR|TRAF2|PIAS1|PDGFRA|PDGFRB|MAPK6|SMAD7|SMAD2|TRAF4|SMURF2|CCAR1|IRF8|SOD1|ACKR3|PDCD4|ATG3|DEPTOR|CSF1R|AIFM1|GATAD1|SMAD6|SNIP1|BCL2L1|MAEA|NFATC2IP|IKZF1|SMAD3|PDCD1|BCL9L|CXCL14|BCL11A|SMAD4|IKZF3|AREL1|BCL11B|IRF5|FXR1|MAP3K5|SMURF1|ILF2|IRF2BP2|IRF6|MAPKAPK2|TAB2|GATAD2B|IKZF5|MCL1|ATG5|BAG3|MAP3K7|IRAK1|PI4K2A|LCOR|YWHAB|TGFBR1|HIF3A|BCOR|TAB3|CAAP1|PDCD10|BCL2L11|NFATC2|BCL2|BCL6|CXCR4|MAP3K2|NFATC4|PEX5|ITK|HLA-B|NCOA2|ILF3|TBRG1|IKZF2|LAT2|SMAD1|CDKN2AIP|IKBKB|TRAF6|BCLAF1|BCORL1|HIF1A|NKRF|BCL7A|SOD2|JKAMP|MOAP1|TRAF3|NFAT5|TXNIP|IKBKE|PIAS2|NOS1|MAP3K14 | 77 | 469|529|285|117|444|554|516|506|235|195|200|194|362|111|398|281|247|311|113|408|153|482|530|369|337|372|207|347|220|241|537|125|468|507|470|392|437|128|160|245|335|464|443|178|487|268|184|414|412|278|312|318|358|434|399|233|440|368|510|541|206|428|316|234|115|475|310|450|374|249|514|556|114|455|110|445|486|467|193|324|526|315|180|387|495|291|303|381|382|138|557|393|415|548|284|508|171 | 4.24E-08 |  | KW-0832 | 0.600788851 | 0 |
| 425 | 31 | GO Process |  | negative regulation of immune system process | 9.93E-07 | TGFB1|IL1RL1|TGFB3|CCL21|ARG2|SMAD7|CDK6|TNFSF4|TNFRSF21|CTLA4|SOCS5|IL7R|PAWR|SOCS1|PDCD1|CCL28|TGFB2|BCL10|TRAF3IP1|TLR4|IRF4|IL33|CXCL12|PDCD1LG2|SOCS6|BCL6|TLR6|HLA-B|IL10|TICAM2|TNFAIP3 | 77 | 350|336|265|500|518|195|365|383|513|183|226|176|396|460|537|488|376|323|474|342|169|346|181|373|244|310|305|114|425|329|164 | 6.56E-08 |  | GO.0002683 | 0.600305075 | 0 |
| 118 | 16 | GO Process |  | regulation of B cell activation | 1.02E-06 | TGFB1|IL7|TNFSF4|TNFRSF13C|TNFRSF21|CTLA4|IL13|PAWR|IKZF3|TNFSF13B|NFATC2|BCL2|BCL6|IL6|IL10|TNFAIP3 | 77 | 350|266|383|224|513|183|418|396|392|326|115|475|310|218|425|164 | 6.79E-08 |  | GO.0050864 | 0.599139983 | 0 |
| 189 | 20 | GO Process |  | transmembrane receptor protein serine/threonine kinase signaling pathway | 1.05E-06 | TGFBR3|TGFB1|TGFB3|SMAD7|SMAD2|SMURF2|PDCD4|SMAD6|SMAD3|SMAD4|TGFBR2|SMURF1|TGFB2|MAP3K7|IRAK1|TGIF2|TGFBR1|LTBP1|SMAD1|SMAD5 | 77 | 447|350|265|195|200|362|311|369|241|470|332|464|376|358|434|259|368|154|193|201 | 6.96E-08 |  | GO.0007178 | 0.59788107 | 0 |
| 189 | 20 | GO Process |  | response to reactive oxygen species | 1.05E-06 | MAPK1|CCR7|TRAF2|PDGFRA|PDGFRB|PRDX5|SOD1|AIFM1|CFLAR|MAP3K5|TXNRD3|PDCD10|MAPK8|BCL2|TXNRD2|IL6|MAPK9|SOD2|TXNIP|TNFAIP3 | 77 | 469|243|444|516|506|208|281|482|133|335|263|316|282|475|476|218|250|381|415|164 | 6.96E-08 |  | GO.0000302 | 0.59788107 | 0 |
| 149 | 17 | KEGG Pathways | | MicroRNAs in cancer | 1.09E-06 | MAPK1|NFKB1|CDKN1B|BCL2L2|PDGFRA|PDGFRB|STAT3|CDK6|PDCD4|MAP2K1|SOCS1|TGFB2|MCL1|BCL2L11|BCL2|IKBKB|DICER1 | 77 | 469|529|285|551|516|506|334|365|311|292|460|376|278|234|475|526|121 | 2.54E-07 |  | hsa05206 | 0.59625735 | 0 |
| 103 | 15 | GO Process |  | regulation of transforming growth factor beta receptor signaling pathway | 1.12E-06 | TGFBR3|TGFB1|TGFB3|SMAD7|SMAD2|SMURF2|SMAD6|SMAD3|BCL9L|IL17F|SMAD4|TGFBR2|SMURF1|TGFBR1|LTBP1 | 77 | 447|350|265|195|200|362|369|241|125|367|470|332|464|368|154 | 7.54E-08 |  | GO.0017015 | 0.595078198 | 0 |
| 69 | 12 | KEGG Pathways | | Adipocytokine signaling pathway | 1.18E-06 | TNFRSF1A|NFKB1|TRAF2|STAT3|SOCS3|MAPK10|TNFRSF1B|JAK2|MAPK8|PPARA|MAPK9|IKBKB | 77 | 351|529|444|334|246|380|276|309|282|491|250|526 | 2.81E-07 |  | hsa04920 | 0.592811799 | 0 |
| 37 | 10 | GO Process |  | positive regulation of T cell mediated immunity | 1.21E-06 | TRAF2|IL1B|TNFSF4|IL12A|MAP3K7|IL6|IL1R1|IL18R1|HLA-B|TRAF6 | 77 | 444|345|383|519|358|218|274|287|114|315 | 8.12E-08 |  | GO.0002711 | 0.591721463 | 0 |
| 89 | 14 | GO Process |  | positive regulation of adaptive immune response based on somatic recombination of immune receptors built from immunoglobulin superfamily domains | 1.30E-06 | TGFB1|TRAF2|IL1B|TNFSF4|TNFRSF13C|IL12A|SOCS5|MAP3K7|TNFSF13B|IL6|IL1R1|IL18R1|HLA-B|TRAF6 | 77 | 350|444|345|383|224|519|226|358|326|218|274|287|114|315 | 8.74E-08 |  | GO.0002824 | 0.588605665 | 0 |
| 155 | 18 | GO Process |  | cellular response to interferon-gamma | 1.31E-06 | CX3CL1|CCL1|CCL13|CCL21|IRF8|IRF5|CCL20|IRF6|TLR4|HLA-DQB1|CCL7|IRF4|JAK2|IRF9|HLA-B|CCL5|CCL16|CCL4 | 77 | 325|484|485|500|398|160|308|487|342|109|289|169|309|492|114|135|501|118 | 8.87E-08 |  | GO.0071346 | 0.58827287 | 0 |
| 448 | 32 | UniProt Keywords | | Immunoglobulin domain | 1.35E-06 | CRTAM|IL1RL1|ISLR|LILRA1|PXDN|PDGFRA|PDGFRB|IGSF6|ILDR2|CSF1R|KIR3DL3|CTLA4|ALCAM|ICOS|IGDCC4|IGDCC3|BTLA|PDCD1|KIR2DL4|NCR3LG1|IL6R|IGSF3|IGSF1|IL1RAPL2|IL6ST|IGSF11|PDCD1LG2|IL1R1|IL18R1|HLA-DMB|VSIG1|MR1 | 77 | 151|336|477|344|543|516|506|546|237|153|442|183|286|167|457|261|449|537|515|313|348|270|132|252|391|130|373|274|287|553|378|540 | 6.29E-08 |  | KW-0393 | 0.586966623 | 0 |
| 11 | 7 | GO Function | | I-SMAD binding | 1.37E-06 | SMAD7|SMAD2|SMAD6|SMAD4|SMURF1|TGFBR1|SMAD1 | 77 | 195|200|369|470|464|368|193 | 7.66E-08 |  | GO.0070411 | 0.586327943 | 0 |
| 174 | 19 | GO Process |  | regulation of cytokine secretion | 1.39E-06 | CRTAM|IL1RL1|CCR7|ARG2|IL1A|IL1B|CSF1R|IL17RB|TNFRSF21|IL1RAP|IL17F|ATG5|TLR4|IL33|IL36RN|TLR6|MAPKBP1|IL10|TNFRSF9 | 77 | 151|336|243|518|322|345|153|497|513|199|367|312|342|346|536|305|298|425|473 | 9.45E-08 |  | GO.0050707 | 0.58569852 | 0 |
| 62 | 12 | GO Process |  | regulation of B cell proliferation | 1.48E-06 | IL7|TNFRSF13C|TNFRSF21|CTLA4|IL13|PAWR|IKZF3|TNFSF13B|NFATC2|BCL2|BCL6|IL10 | 77 | 266|224|513|183|418|396|392|326|115|475|310|425 | 1.01E-07 |  | GO.0030888 | 0.582973828 | 0 |
| 19 | 8 | GO Process |  | positive regulation of T cell cytokine production | 1.49E-06 | TRAF2|IL1B|TNFSF4|MAP3K7|IL6|IL1R1|IL18R1|TRAF6 | 77 | 444|345|383|358|218|274|287|315 | 1.02E-07 |  | GO.0002726 | 0.582681373 | 0 |
| 122 | 16 | GO Process |  | antigen receptor-mediated signaling pathway | 1.51E-06 | MAPK1|NFKB1|TRAT1|TNFRSF21|CTLA4|TAB2|THEMIS|MAP3K7|BCL10|HLA-DQB1|NFATC2|BCL2|ITK|LAT2|IKBKB|TRAF6 | 77 | 469|529|527|513|183|184|349|358|323|109|115|475|556|467|526|315 | 1.03E-07 |  | GO.0050851 | 0.582102305 | 0 |
| 663 | 40 | GO Process |  | regulation of growth | 1.54E-06 | TGFBR3|TGFB1|CDKN1B|RIMS2|IL7|STAT3|IRF8|SOD1|IL9|IL17RB|AVPR1A|BCL2L1|SOCS5|PPARD|TNFRSF12A|CSF1|SOCS1|SOCS3|SMAD3|BCL11A|SMAD4|RBPJ|TGFBR2|SMURF1|TGFB2|TGFBR1|BCL2L11|IGSF11|SOCS4|ULK2|CXCL12|SOCS6|BCL2|BCL6|OSGIN2|IL10|CDKN2AIP|HIF1A|SOCS2|SOCS7 | 77 | 447|350|285|331|266|334|398|281|295|497|222|372|226|227|340|149|460|246|241|507|470|438|332|464|376|368|234|130|523|214|181|244|475|310|446|425|324|495|419|186 | 1.06E-07 |  | GO.0040008 | 0.581247928 | 0 |
| 28 | 9 | GO Process |  | production of miRNAs involved in gene silencing by miRNA | 1.54E-06 | SMAD2|SNIP1|PRKRA|SMAD3|AGO3|AGO1|AGO4|SMAD1|DICER1 | 77 | 200|337|196|241|520|239|240|193|121 | 1.06E-07 |  | GO.0035196 | 0.581247928 | 0 |
| 2463 | 98 | GO Process |  | negative regulation of cellular metabolic process | 1.54E-06 | TGFB1|NFKB1|CDKN1B|SOGA1|ATG14|LIF|PIAS1|MAP3K10|SMAD7|SMAD2|SMURF2|IL1B|ATF2|STAT3|PRDX5|LAMP3|CDK6|CCAR1|IRF8|TNFRSF10B|PDCD4|TNFSF4|DEPTOR|BAG4|SMAD6|TRAT1|CREBRF|HIF1AN|NFATC3|BCL2L1|MAEA|SOCS5|PPARD|CFLAR|TNIP1|TGIF1|PAWR|SOCS1|SOCS3|IKZF1|SMAD3|BCL11A|BAG5|SMAD4|RBPJ|ATG7|PEX2|FXR1|TGFB2|MAPKAPK2|TAB2|IKZF5|MCL1|ATG5|BAG3|TNFRSF6B|BAG2|LCOR|YWHAB|AGO3|AGO1|AGO4|TRAF3IP1|TGIF2|TLR4|TNFRSF1B|HIF3A|BCOR|TAB3|NFIB|LOXL2|SOCS4|NFATC2|SOCS6|BCL2|BCL6|PPARA|IL6|NFATC4|NCOA2|ILF3|OXR1|IL10|IKZF2|TNFAIP8|IKBKB|TRAF6|BCLAF1|DICER1|HIF1A|SMAD5|NKRF|BCL7A|TXNIP|PANO|SOCS2|TNFAIP3|SOCS7 | 77 | 350|529|285|317|150|432|554|489|195|200|362|345|189|334|208|377|365|111|398|410|311|383|408|409|369|527|296|228|210|372|207|226|227|133|538|143|396|460|246|220|241|507|124|470|438|119|269|245|376|268|184|412|278|312|318|481|116|233|440|520|239|240|474|259|342|276|510|541|206|170|480|523|115|244|475|310|491|218|249|455|110|144|425|486|185|526|315|180|121|495|201|291|303|415|172|419|164|186 | 1.07E-07 |  | GO.0031324 | 0.581247928 | 0 |
| 1355 | 64 | GO Process |  | movement of cell or subcellular component | 1.54E-06 | CX3CL1|TGFBR3|MAPK1|TGFB1|CCL1|CCL13|TNFAIP1|CXCL6|CCR7|PDGFRA|CCL21|PDGFRB|IL1B|SOD1|TNFRSF10B|DOCK1|CSF1R|CXCR5|PEX13|CXCL3|CXCL5|MAP2K1|IL16|IL12A|ALCAM|CXCL11|PPARD|TNFRSF12A|CCR4|CXCL14|SMAD4|GPR29|BCL11B|CCL20|CCL28|CXCL9|TGFB2|IL6R|TRAF3IP1|CXCR3|TGFBR1|CCL7|NFIB|JAK2|LOXL2|LYST|PDCD10|CXCL12|NFATC2|BLOC1S5|TNFSF11|CMKBR6|IL6|CXCR4|IRAK4|DOCK4|IL10|HIF1A|TNFRSF11A|IER2|CCL5|CCL16|SOCS7|CCL4 | 77 | 325|447|469|350|484|485|280|531|243|516|500|506|345|281|410|215|153|306|152|356|355|292|385|519|286|321|227|340|502|468|470|448|128|308|488|283|376|348|474|230|368|289|170|309|480|555|316|181|115|198|430|431|218|450|371|544|425|495|163|354|135|501|186|118 | 1.07E-07 |  | GO.0006928 | 0.581247928 | 0 |
| 28 | 9 | GO Process |  | regulation of T cell cytokine production | 1.54E-06 | TRAF2|SMAD7|IL1B|TNFSF4|MAP3K7|IL6|IL1R1|IL18R1|TRAF6 | 77 | 444|195|345|383|358|218|274|287|315 | 1.06E-07 |  | GO.0002724 | 0.581247928 | 0 |
| 412 | 30 | GO Process |  | regulation of hemopoiesis | 1.60E-06 | TGFB1|TNFSF9|LIF|SMAD7|IL7|STAT3|CDK6|SOD1|TNFSF4|IL12A|CTLA4|SOCS5|IL7R|C8orf4|CSF1|SOCS1|IKZF3|TGFBR2|IL20|AGO3|AGO1|AGO4|TLR4|IRF4|NFATC2|TNFSF11|BCL6|HLA-B|TRAF6|HIF1A | 77 | 350|120|432|195|266|334|365|281|383|519|183|226|176|293|149|460|392|332|161|520|239|240|342|169|115|430|310|114|315|495 | 1.11E-07 |  | GO.1903706 | 0.579588002 | 0 |
| 140 | 17 | GO Process |  | cellular response to transforming growth factor beta stimulus | 1.62E-06 | TGFBR3|TGFB1|TGFB3|SMAD7|SMAD2|NOX4|SMAD6|SMAD3|SMAD4|TGFBR2|SMURF1|TGFB2|MAP3K7|TGFBR1|SMAD1|SMAD5|APAF1 | 77 | 447|350|265|195|200|364|369|241|470|332|464|376|358|368|193|201|148 | 1.13E-07 |  | GO.0071560 | 0.579048499 | 0 |
| 486 | 33 | GO Process |  | cellular response to lipid | 1.69E-06 | MAPK1|TGFB1|CXCL6|NFKB1|PIAS1|IRAK2|FDX1|IL1B|IRF8|PDCD4|TNFSF4|AIFM1|IL12A|PPARD|CFLAR|TNIP1|CCL20|IRAK1|TLR4|TNFRSF1B|JAK2|BCL2L11|MAPK8|PPARA|IL6|NFATC4|IL10|TICAM2|TRAF6|LITAF|PIAS2|CCL5|TNFAIP3 | 77 | 469|350|531|529|554|258|353|345|398|311|383|482|519|227|133|538|308|434|342|276|309|234|282|491|218|249|425|329|315|395|284|135|164 | 1.18E-07 |  | GO.0071396 | 0.57721133 | 0 |
| 26 | 9 | InterPro Domains | | Chemokine beta/gamma/delta | 1.76E-06 | CX3CL1|CCL1|CCL13|CCL21|CCL20|CCL7|CCL5|CCL16|CCL4 | 77 | 325|484|485|500|308|289|135|501|118 | 6.24E-08 |  | IPR039809 | 0.575448733 | 0 |
| 159 | 18 | GO Process |  | negative regulation of cell-cell adhesion | 1.79E-06 | TGFB1|CCL21|ARG2|SMAD7|TNFSF4|TNFRSF21|CTLA4|SOCS5|PAWR|SOCS1|CCL28|JAK2|CXCL12|PDCD1LG2|SOCS6|BCL6|PPARA|IL10 | 77 | 350|500|518|195|383|513|183|226|396|460|488|309|181|373|244|310|491|425 | 1.26E-07 |  | GO.0022408 | 0.574714697 | 0 |
| 6 | 6 | Reactome Pathways | | SMAD2/3 MH2 Domain Mutants in Cancer | 1.80E-06 | TGFB1|SMAD2|SMAD3|SMAD4|TGFBR2|TGFBR1 | 77 | 350|200|241|470|332|368 | 1.10E-07 |  | HSA-3315487 | 0.574472749 | 0 |
| 50 | 11 | GO Function | | transforming growth factor beta receptor binding | 1.93E-06 | TGFBR3|TGFB1|TGFB3|SMAD7|SMAD2|SMURF2|SMAD6|SMAD3|TGFBR2|TGFB2|TGFBR1 | 77 | 447|350|265|195|200|362|369|241|332|376|368 | 1.11E-07 |  | GO.0005160 | 0.571444269 | 0 |
| 393 | 29 | GO Process |  | positive regulation of secretion | 1.97E-06 | TGFB1|CRTAM|IL1RL1|TGFB3|IL1A|IL1B|TNFSF4|CSF1R|IL17RB|AVPR1A|IL13|PPARD|IL1RAP|LAMP1|IL17F|SMAD4|TGFB2|ATG5|TLR4|IL33|JAK2|CXCL12|TNFSF11|IL6|PDCD6IP|IL10|HIF1A|TNFRSF11A|MAP2K6 | 77 | 350|151|336|265|322|345|383|153|497|222|418|227|199|360|367|470|376|312|342|346|309|181|430|218|307|425|495|163|255 | 1.40E-07 |  | GO.0051047 | 0.570553377 | 0 |
| 88 | 13 | KEGG Pathways | | GnRH signaling pathway | 1.98E-06 | MAPK1|MAP2K1|MAP2K3|MAPK10|MAP3K3|MAP3K4|MAPK8|MAP2K7|MAP3K1|MAP3K2|MAPK9|MAP2K4|MAP2K6 | 77 | 469|292|528|380|299|466|282|236|397|374|250|517|255 | 4.82E-07 |  | hsa04912 | 0.570333481 | 0 |
| 348 | 26 | KEGG Pathways | | PI3K-Akt signaling pathway | 2.02E-06 | MAPK1|NFKB1|CDKN1B|PDGFRA|PDGFRB|IL7|ATF2|CDK6|CSF1R|TGFA|MAP2K1|BCL2L1|IL7R|CSF1|IFNAR2|IL6R|MCL1|MTCP1|YWHAB|TLR4|IL2RG|JAK2|BCL2L11|BCL2|IL6|IKBKB | 77 | 469|529|285|516|506|266|189|365|153|279|292|372|176|149|452|348|278|162|440|342|563|309|234|475|218|526 | 5.02E-07 |  | hsa04151 | 0.569464863 | 0 |
| 892 | 48 | GO Process |  | positive regulation of transport | 2.04E-06 | MAPK1|TGFB1|CRTAM|IL1RL1|TGFB3|RHOQ|XPO4|CCL21|PDGFRB|IL1A|IL1B|TNFSF4|CSF1R|IL17RB|CREBRF|AVPR1A|IL13|CXCL11|PPARD|IL1RAP|SMAD3|LAMP1|IL17F|SMAD4|CXCL9|NOS1AP|TGFB2|ATG5|BAG3|YWHAB|CXCR3|TLR4|IL33|JAK2|PDCD10|MAPK8|CXCL12|BCL2|TNFSF11|IL6|PDCD6IP|IL10|HIF1A|TNFRSF11A|MAP2K6|CCL5|NOS1|CCL4 | 77 | 469|350|151|336|265|552|386|500|506|322|345|383|153|497|296|222|418|321|227|199|241|360|367|470|283|212|376|312|318|440|230|342|346|309|316|282|181|475|430|218|307|425|495|163|255|135|508|118 | 1.45E-07 |  | GO.0051050 | 0.569036983 | 0 |
| 180 | 19 | GO Process |  | positive regulation of peptidyl-tyrosine phosphorylation | 2.15E-06 | TNFRSF1A|TGFB1|LIF|NOX4|IL11|STAT3|DOCK3|CSF1R|TGFA|RICTOR|IL12A|IL13|SOCS3|IL20|IL6R|IL6ST|JAK2|IL6|CCL5 | 77 | 351|350|432|364|126|334|225|153|279|302|519|418|246|161|348|391|309|218|135 | 1.53E-07 |  | GO.0050731 | 0.566756154 | 0 |
| 3691 | 132 | GO Process |  | regulation of RNA biosynthetic process | 2.18E-06 | TNFRSF1A|TGFBR3|MAPK1|TGFB1|NFKB1|CDKN1B|BCL9|TGFB3|RHOQ|RASL11A|TRAF2|LIF|PIAS1|MAP3K10|IRAK2|TRAF5|SMAD7|SMAD2|SMURF2|IL1A|IL1B|ATF2|IL11|STAT3|MAP3K13|PRDX5|CCAR1|IRF8|PDCD4|TNFSF4|GATAD1|SMAD6|TGFA|SNIP1|CREBRF|HIF1AN|NFATC3|MAP2K1|IL16|PPARD|CFLAR|IL1RAP|TNIP1|C8orf4|NFATC2IP|TGIF1|PAWR|IL25|IKZF1|SMAD3|BCL9L|IL17F|BCL11A|SMAD4|IKZF3|MAP2K3|RBPJ|PEX2|BCL11B|IRF5|MAP3K5|SMURF1|ILF2|IRF2BP2|TGFB2|IRF6|TAB2|HIVEP2|GATAD2B|IKZF5|BAG3|MAP3K7|IRAK1BP1|IRAK1|BCL10|IGSF1|LCOR|YWHAB|AGO1|CXCR3|TGIF2|TLR4|TGFBR1|HIF3A|BCOR|TAB3|NFIB|IRF4|IL33|JAK2|LOXL2|NFATC2|IRF9|TNFSF11|BCL6|PPARA|IL6|IL18R1|MAP3K2|NFATC4|TLR6|IRAK4|NCOA2|ILF3|IL10|IKZF2|SMAD1|IKBKB|TRAF6|BCLAF1|DICER1|BCORL1|HIF1A|SMAD5|NKRF|TET2|BCL7A|SOD2|MAP3K9|TRAF3|NFAT5|LITAF|TXNIP|TNFRSF11A|IER2|PIAS2|ERC1|MAP2K6|CCL5|NOS1|TNFAIP3|CCL4 | 77 | 351|447|469|350|529|285|175|265|552|205|444|432|554|489|258|142|195|200|362|322|345|189|126|334|490|208|111|398|311|383|530|369|279|337|296|228|210|292|385|227|133|199|538|293|347|143|396|478|220|241|125|367|507|470|392|528|438|269|128|160|335|464|443|178|376|487|184|433|414|412|318|358|400|434|323|132|233|440|239|230|259|342|368|510|541|206|170|169|346|309|480|115|492|430|310|491|218|287|374|249|305|371|455|110|425|486|193|526|315|180|121|387|495|201|291|294|303|381|290|557|393|395|415|163|354|284|401|255|135|508|164|118 | 1.56E-07 |  | GO.2001141 | 0.566154351 | 0 |
| 478 | 33 | InterPro Domains | | Immunoglobulin-like domain superfamily | 2.22E-06 | CRTAM|IL1RL1|ISLR|LILRA1|PXDN|PDGFRA|PDGFRB|IGSF6|ILDR2|CSF1R|KIR3DL3|CTLA4|ALCAM|IL1RAP|IGDCC4|IGDCC3|BTLA|PDCD1|KIR2DL4|NCR3LG1|IL6R|IGSF3|IGSF1|IL1RAPL2|HLA-DQB1|IGSF11|PDCD1LG2|IL1R1|IL18R1|HLA-DMB|HLA-B|VSIG1|MR1 | 77 | 151|336|477|344|543|516|506|546|237|153|442|183|286|199|457|261|449|537|515|313|348|270|132|252|109|130|373|274|287|553|114|378|540 | 8.24E-08 |  | IPR036179 | 0.565364703 | 0 |
| 181 | 19 | GO Process |  | regulation of ossification | 2.32E-06 | MAPK1|TGFB1|TGFB3|CDK6|SMAD6|CSF1|SMAD3|RBPJ|SMURF1|TGFB2|IL6R|BCOR|IL6ST|BCL2|IL6|SMAD1|HIF1A|SMAD5|PIAS2 | 77 | 469|350|265|365|369|149|241|438|464|376|348|541|391|475|218|193|495|201|284 | 1.66E-07 |  | GO.0030278 | 0.563451202 | 0 |
| 397 | 29 | GO Process |  | positive regulation of establishment of protein localization | 2.38E-06 | MAPK1|TGFB1|CRTAM|IL1RL1|TGFB3|XPO4|IL1A|IL1B|TNFSF4|CSF1R|IL17RB|CREBRF|IL13|PPARD|IL1RAP|SMAD3|IL17F|TGFB2|BAG3|YWHAB|TLR4|IL33|JAK2|PDCD10|MAPK8|BCL2|IL6|IL10|HIF1A | 77 | 469|350|151|336|265|386|322|345|383|153|497|296|418|227|199|241|367|376|318|440|342|346|309|316|282|475|218|425|495 | 1.71E-07 |  | GO.1904951 | 0.562342304 | 0 |
| 41 | 10 | GO Process |  | autophagy of mitochondrion | 2.56E-06 | ATG14|ATG3|ATG4D|ATG7|ATG2B|ATG5|ATG9A|ATG12|AMBRA1|ATG13 | 77 | 150|113|213|119|389|312|257|421|131|559 | 1.85E-07 |  | GO.0000422 | 0.559176003 | 0 |
| 142 | 17 | UniProt Keywords | | Autophagy | 2.70E-06 | ATG14|IRF8|EPG5|ATG10|ATG3|WDR24|ATG4D|ATG7|ATG2B|ATG5|ATG16L1|ULK2|ATG9A|LAMP2|ATG12|AMBRA1|ATG13 | 77 | 150|398|361|155|113|260|213|119|389|312|223|214|257|304|421|131|559 | 1.36E-07 |  | KW-0072 | 0.556863624 | 0 |
| 3890 | 137 | GO Process |  | regulation of RNA metabolic process | 2.70E-06 | TNFRSF1A|TGFBR3|MAPK1|TGFB1|NFKB1|CDKN1B|BCL9|TGFB3|RHOQ|RASL11A|TRAF2|LIF|PIAS1|MAP3K10|IRAK2|TBRG4|TRAF5|SMAD7|SMAD2|SMURF2|IL1A|IL1B|ATF2|IL11|STAT3|MAP3K13|PRDX5|CCAR1|IRF8|PDCD4|TNFSF4|GATAD1|SMAD6|TGFA|SNIP1|CREBRF|HIF1AN|NFATC3|MAP2K1|IL16|PPARD|CFLAR|IL1RAP|TNIP1|C8orf4|NFATC2IP|TGIF1|PAWR|IL25|IKZF1|SMAD3|BCL9L|IL17F|BCL11A|SMAD4|IKZF3|MAP2K3|RBPJ|PEX2|BCL11B|IRF5|MAP3K5|SMURF1|ILF2|IRF2BP2|TGFB2|IRF6|MAPKAPK2|TAB2|HIVEP2|GATAD2B|IKZF5|BAG3|MAP3K7|IRAK1BP1|IRAK1|BCL10|EXOSC1|IGSF1|LCOR|YWHAB|AGO1|CXCR3|TGIF2|TLR4|TGFBR1|TNFRSF1B|HIF3A|BCOR|TAB3|NFIB|IRF4|IL33|JAK2|LOXL2|EXOSC8|NFATC2|IRF9|TNFSF11|BCL6|PPARA|IL6|IL18R1|MAP3K2|NFATC4|TLR6|IRAK4|NCOA2|ILF3|IL10|IKZF2|SMAD1|IKBKB|TRAF6|BCLAF1|DICER1|BCORL1|HIF1A|SMAD5|NKRF|TET2|BCL7A|SOD2|MAP3K9|TRAF3|NFAT5|LITAF|TXNIP|TNFRSF11A|IER2|PIAS2|ERC1|MAP2K6|CCL5|NOS1|TNFAIP3|CCL4 | 77 | 351|447|469|350|529|285|175|265|552|205|444|432|554|489|258|496|142|195|200|362|322|345|189|126|334|490|208|111|398|311|383|530|369|279|337|296|228|210|292|385|227|133|199|538|293|347|143|396|478|220|241|125|367|507|470|392|528|438|269|128|160|335|464|443|178|376|487|268|184|433|414|412|318|358|400|434|323|137|132|233|440|239|230|259|342|368|276|510|541|206|170|169|346|309|480|297|115|492|430|310|491|218|287|374|249|305|371|455|110|425|486|193|526|315|180|121|387|495|201|291|294|303|381|290|557|393|395|415|163|354|284|401|255|135|508|164|118 | 1.96E-07 |  | GO.0051252 | 0.556863624 | 0 |
| 203 | 20 | GO Process |  | myeloid cell differentiation | 2.78E-06 | TGFBR3|TGFB1|CCR7|IL11|IRF8|CSF1R|MAEA|CSF1|IL25|IKZF1|RBPJ|TGFBR2|IRF4|JAK2|TNFSF11|BCL6|TRAF6|SMAD5|TET2|TNFRSF11A | 77 | 447|350|243|126|398|153|207|149|478|220|438|332|169|309|430|310|315|201|294|163 | 2.02E-07 |  | GO.0030099 | 0.55559552 | 0 |
| 66 | 12 | Reactome Pathways | | Macroautophagy | 2.88E-06 | ATG14|ATG10|ATG3|ATG4D|LAMTOR4|ATG7|ATG5|ATG16L1|ATG9A|ATG12|AMBRA1|ATG13 | 77 | 150|155|113|213|188|119|312|223|257|421|131|559 | 1.84E-07 |  | HSA-1632852 | 0.554060751 | 0 |
| 266 | 23 | GO Process |  | immune response-regulating cell surface receptor signaling pathway | 2.99E-06 | MAPK1|NFKB1|DOCK1|TRAT1|TNFRSF21|NFATC3|CTLA4|MAPK10|TAB2|THEMIS|MAP3K7|BCL10|HLA-DQB1|TAB3|MAPK8|NFATC2|BCL2|MAP3K1|MAPK9|ITK|LAT2|IKBKB|TRAF6 | 77 | 469|529|215|527|513|210|183|380|184|349|358|323|109|206|282|115|475|397|250|556|467|526|315 | 2.19E-07 |  | GO.0002768 | 0.552432881 | 0 |
| 96 | 14 | Reactome Pathways | | TNFR2 non-canonical NF-kB pathway | 3.06E-06 | TNFRSF1A|TNFSF9|TRAF2|TNFSF4|TNFRSF13C|TNFRSF11B|TNFRSF12A|TNFSF13B|TNFRSF1B|TNFSF11|TRAF3|TNFRSF11A|TNFRSF9|MAP3K14 | 77 | 351|120|444|383|224|359|340|326|276|430|557|163|473|171 | 2.02E-07 |  | HSA-5668541 | 0.551427857 | 0 |
| 31 | 9 | Reactome Pathways | | TGF-beta receptor signaling activates SMADs | 3.20E-06 | TGFB1|SMAD7|SMAD2|SMURF2|SMAD3|SMAD4|TGFBR2|SMURF1|TGFBR1 | 77 | 350|195|200|362|241|470|332|464|368 | 2.19E-07 |  | HSA-2173789 | 0.549485002 | 0 |
[truncated: 409,739 more chars]
